# GproDIA enables data-independent acquisition glycoproteomics with comprehensive statistical control

**DOI:** 10.1038/s41467-021-26246-3

**Published:** 2021-10-18

**Authors:** Yi Yang, Guoquan Yan, Siyuan Kong, Mengxi Wu, Pengyuan Yang, Weiqian Cao, Liang Qiao

**Affiliations:** 1 Department of Chemistry and Institutes of Biomedical Sciences, Fudan University, Shanghai, 200000 China; 2 The Shanghai Key Laboratory of Medical Epigenetics, Fudan University, Shanghai, 200000 China; 3 The International Co-laboratory of Medical Epigenetics and Metabolism (Ministry of Science and Technology), Fudan University, Shanghai, 200000 China; 4 NHC Key Laboratory of Glycoconjugates Research, Fudan University, Shanghai, 200000 China

**Keywords:** Glycobiology, Proteomics, Mass spectrometry, Proteome informatics

## Abstract

Large-scale profiling of intact glycopeptides is critical but challenging in glycoproteomics. Data independent acquisition (DIA) is an emerging technology with deep proteome coverage and accurate quantitative capability in proteomics studies, but is still in the early stage of development in the field of glycoproteomics. We propose GproDIA, a framework for the proteome-wide characterization of intact glycopeptides from DIA data with comprehensive statistical control by a 2-dimentional false discovery rate approach and a glycoform inference algorithm, enabling accurate identification of intact glycopeptides using wide isolation windows. We further utilize a semi-empirical spectrum prediction strategy to expand the coverage of spectral libraries of glycopeptides. We benchmark our method for N-glycopeptide profiling on DIA data of yeast and human serum samples, demonstrating that DIA with GproDIA outperforms the data-dependent acquisition-based methods for glycoproteomics in terms of capacity and data completeness of identification, as well as accuracy and precision of quantification. We expect that this work can provide a powerful tool for glycoproteomic studies.

## Abstract (web summary)

Data independent acquisition (DIA) proteomics provides deep coverage and high quantitative accuracy, but is not yet well established in glycoproteomics. Here, the authors develop a DIA-based glycoproteomics workflow with stringent statistical controls to enable accurate glycopeptide identification.

## Introduction

Protein glycosylation is one of the most abundant and heterogeneous post-translational modifications (PTMs) that provides great proteomic diversity and plays a key role in various biological processes^1–3^, even the host–pathogen interaction of the ongoing coronavirus disease 2019 pandemic^4^. Precise characterization of protein glycosylation is critical for understanding mechanism of diseases^5,6^, discovery of biomarkers for diagnosis^7^, and development of drugs and vaccines^8^. The high heterogeneity of glycans across glycosites results in an increased number of glycoproteoforms. Profiling of intact glycopeptide provides the opportunity of simultaneous analysis of glycans, glycosite occupancy and site-specific glycosylation on a proteome-wide scale^9^, and is an imperative but still challenging component to modern glycoproteomic studies^10^.

Currently, liquid chromatography coupled with tandem mass spectrometry (LC-MS/MS) is the method of choice widely used in proteomics and glycoproteomics^11,12^. Novel MS/MS fragmentation methods derived from higher-energy collisional dissociation (HCD) and electron transfer dissociation (ETD), such as stepped collision energy HCD (SCE-HCD)^13^ and ETD with supplemental HCD (EThcD)^14^, have been proven powerful for intact glycopeptides profiling. The most common strategy for glycopeptide profiling uses the data-dependent acquisition (DDA) approach, in which MS/MS (MS2) fragmentation is triggered by precursor ions observed in a full mass range survey scan (MS1). Various software tools^15^, such as Byonic^16^, pGlyco^17,18^, MSFragger-Glyco^19^, MetaMorpheus^20^, et al., have been developed for the interpretation of DDA data of intact glycopeptides. In pGlyco, comprehensive quality control has been developed with error rate evaluation on all levels of matches to glycans, peptides and glycopeptides^17,18^. However, a major bottleneck of the DDA approach is that the precursor selection constitutes a stochastic element^21^, resulting in the “missing value” problem.

To overcome this limitation, data-independent acquisition (DIA) methods have been proposed^22–24^, including a representative variant named sequential window acquisition of all theoretical mass spectra (SWATH-MS)^25^, where the instrument acquires fragmentation information of all precursor ions within defined isolation windows in a systematic manner. DIA has been reported to achieve deep proteome coverage with quantitative consistency and accuracy for large-scale proteomic studies^26^, and is now starting to be applied to the field of glycoproteomics^27^. Based on standard DIA protocols developed for proteomics, Zacchi et al. developed DIA analysis of intact glycopeptides to measure the pattern of glycosylation at eight N-glycosites in *Saccharomyces cerevisiae*^28^. In their protocol, spectral libraries of glycopeptides were generated using fragment ions from nonglycosylated counterparts of each peptide and precursor masses corresponding to various glycans. Sanda et al. reported detection of intact IgG glycoforms from human plasma, where spectral libraries were built by adding manually curated glycan fragment Y ions^29,30^. Pan et al. built a spectral library containing both peptide fragments and glycan Y ions, achieving site-specific N-glycosylation analysis of six glycosites in IgM^31^. Zhou et al. developed a SWATH-MS method with optimized variable windows for a set of target glycopeptides to allow accurate glycoform measurement^32^. These methods have achieved better sensitivity than DDA for targeted analysis of glycoforms of several or a dozen of glycoproteins. In 2019, Ye et al. proposed Glyco-DIA, a DIA-based strategy for O-glycoproteomics, enabling high-throughput quantitative O-GalNAc type glycoproteomic analysis in complex biological samples^33^.

Error rate control for glycopeptide identification is essential but particularly complicated in DIA analyses due to the increased complexity of DIA MS/MS spectra originated from multiple co-eluted precursors, especially when using wide isolation windows. In the case of HCD MS/MS, the same set of fragment ions could be generated for glycopeptides common in peptide sequence but different in glycan, resulting in a high level of misinterpretations of DIA data^27^. Although a few studies have elucidated error rate estimation for DDA-based glycopeptide analyses^17,34–36^, statistical control of DIA-based proteome-wide glycopeptides analyses, to the best of our knowledge, has not been properly addressed.

Herein, we propose GproDIA, a pipeline that applies the concept of peptide-centric DIA analysis to proteome-wide characterization of intact glycopeptides. GproDIA provides comprehensive statistical control by a 2-dimentional (2D) false discovery rate (FDR) approach and a glycoform inference algorithm, enabling accurate glycopeptide identification using wide isolation windows. We further utilize a semi-empirical spectrum prediction strategy to expand the coverage of spectral libraries for glycopeptides. We benchmarked GproDIA for N-glycopeptide profiling on DIA data of yeast samples, which only contain high-mannose-type glycans, as well as human serum samples with glycomes of much more complexity. The results demonstrate that DIA with GproDIA outperforms DDA in terms of capacity and data completeness of identification, as well as accuracy and precision of quantification.

## Results

### GproDIA enables characterization of intact glycopeptides from DIA data

**Fig. 1:**
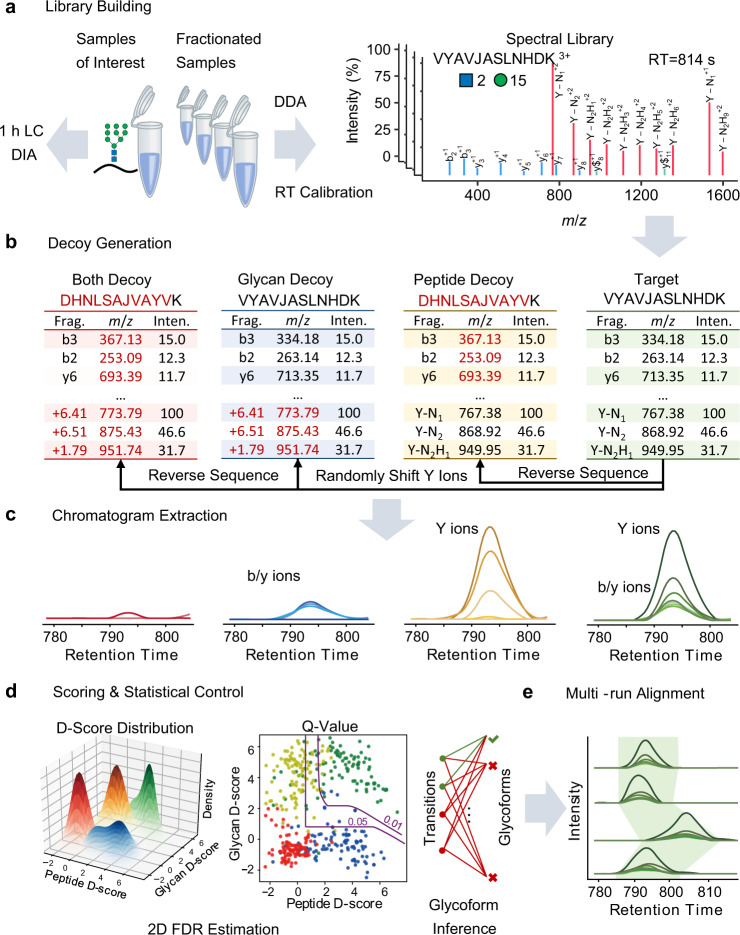
The workflow of GproDIA. **a** Building a spectral library of glycopeptides containing peptide ions (blue lines for b/y, and green lines for b/y with one HexNAc or its cross-ring fragment) and glycan Y ions (red lines) by DDA. “J” in peptide sequence indicates the N-glycosylation site. The glycan symbols are as follows: a green circle or “H” represents Hex; a blue square or “N” represents HexNAc. **b** Generating peptide decoys, glycan decoys and both decoys. **c** Extracting chromatogram features of the target glycopeptides and the decoys from the DIA data. **d** Scoring the extracted features and estimating error rates by a 2-dimentional FDR approach and a glycoform inference strategy. **e** Performing multi-run alignment to reduce missing values. **c–e** Green color indicates target peak groups, yellow indicates peptide decoy peak groups, blue indicates glycan decoy peak groups, and red indicates both decoy peak groups. Details on the glycoform inference strategy is illustrated in Fig. 3 and Supplementary Fig. 11.

GproDIA was developed based on the principle of peptide-centric analysis, which has been commonly used for the detection of peptides from DIA data^26^. The workflow is presented in Fig. 1. First, a spectral library of glycopeptides was built by DDA with pre-fractionation or using a long LC gradient. As LC conditions in this study were different from those used for non-glycosylated peptides, instead of using iRT^37^ as exogenous standards for retention time (RT) normalization, an extra DDA injection of the glycopeptides sample was performed with the same LC condition as that used for DIA experiments. The shared identifications between different LC conditions were used as internal standards to calibrate library RT to the gradient used in DIA. An example of RT calibration is shown in Supplementary Fig. 1. The spectral library contained the RT of glycopeptides, the precursor *m/z*, and *m/z* and intensities of annotated fragments in SCE-HCD MS/MS including peptide fragments (b/y, with or without one HexNAc residue and its cross-ring fragment) and intact peptide with glycan fragments (Y) (Fig. 1a). Next, three types of decoy libraries were generated by adding random mass shifts to glycan fragment peaks (glycan decoy), reversing the peptide sequences (peptide decoy), and performing the two operations successively (both decoy) (Fig. 1b). The idea of glycan decoy by random mass shift was initially reported in pGlyco^38^.

OpenSWATH^39^ was used to extract chromatogram data of the target glycopeptides and the decoys from the DIA data (Fig. 1c), and the extracted peak group features were scored using a semi-supervised learning approach implemented by PyProphet^40^. The peptide discriminant scores (D-scores) were computed using the target peak groups and the glycan decoy peak groups as “targets”, while the peptide decoy peak groups and the both decoy peak groups as “decoys”. Glycan D-scores were computed using the target peak groups and the peptide decoy peak groups as “targets”, while the glycan decoy peak groups and the both decoy peak groups as “decoys”. Error rates of identification were estimated using a 2D FDR approach (Fig. 1d), which extends the conventional FDR method in DIA for peptide identification^40^ to the 2D condition. Indeed, the idea of performing two-tier quality control on glycan and peptide levels has been reported in pGlyco for DDA using decoys generated for both the peptide and glycan parts of a glycopeptide^17,18^. Details of the DIA 2D FDR approach are described in the “Methods” section. In brief, the distributions of peptide and glycan D-scores of the targets and 3 types of decoys were fitted using a bivariate four-groups mixture model. The proportion of target peak groups for which the peptide and/or glycan part was incorrect was estimated from the D-score distributions of targets and 3 types of decoys. Local FDR (namely posterior error probability, PEP), which is the probability of each target peak group to be incorrect, was computed from the ratios of corresponding bivariate densities of the incorrect and total target peak groups with specified D-scores (Supplementary Fig. 2). Global FDR (q-value), which conveys the error introduced in the whole reported results if we accept a peak group with specified D-scores as a positive identification, was then derived from PEP by averaging PEPs of all the positive identifications. The 2D FDR approach using glycan decoys by random mass shift aims to indicate whether a glycan match is a random match and can rule out false identifications in less complex samples, e.g., yeast in this study. For complex samples such as sera, a glycoform inference strategy was further implemented to resolve glycopeptide precursors with the same peptide sequence but different glycans in one isolation window (Fig. 1d). Details of the glycoform inference strategy are described in the “**Inference of glycoforms in wide isolation windows**” subsection below. Finally, TRIC^41^ is used for multi-run alignment to reduce missing values (Fig. 1e).

### Benchmarking using data from yeast samples

**Fig. 2:**
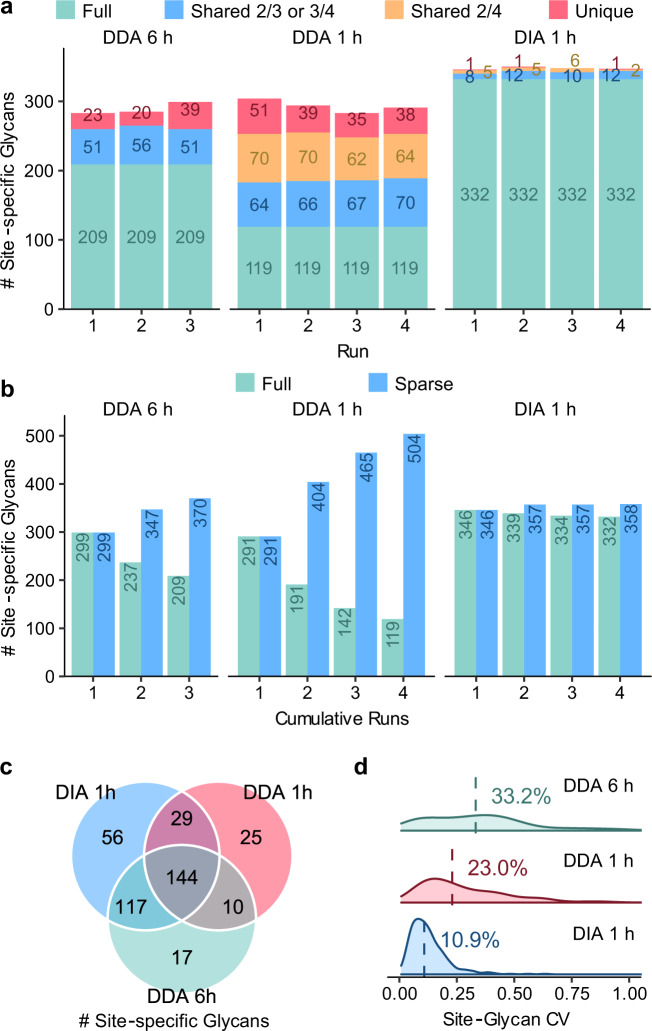
Performance comparison of DDA and DIA on the fission yeast sample at the site-specific glycan level. **a** Numbers of identifications per run. “Full” represents identifications observed in all the runs; “shared 2/3 or 3/4” represents identifications observed in 2 of the 3 runs or 3 of the 4 runs; “shared 2/4” represents identifications observed in 2 of the 4 runs; “unique” represents identifications observed in only 1 run. **b** Numbers of cumulative identifications across runs. “Full” represents identifications shared in the cumulative runs; “sparse” represents identifications observed in at least one run in the cumulative runs. **c** Comparison of numbers of identifications shared in at least 2/3 or 3/4 runs using DDA with an 1 h LC gradient, DDA with a 6 h LC gradient, and DIA with an 1 h LC gradient. **d** Coefficients of variation (CVs) of quantification results. Medians are indicated. Source data are provided as a Source Data file.

For benchmark purposes, we performed DDA and DIA experiments using an 1 h LC gradient with 4 technical replicates (repeat injections of the same sample), as well as a DDA using a 6 h LC gradient with 3 repeat injections, on a sample of fission yeast (*Schizosaccharomyces pombe*) glycopeptides. Glycopeptides were enriched using zwitterioic hydrophilic interaction liquid chromatography (ZIC-HILIC) method^17^. The DDA data were searched using pGlyco3^18^ against the Swiss-Prot *S. pombe* protein database and an embedded glycan database, considering asparagines (N) in N-X-S/T/C (X ≠ P) sequons as potential glycosites. Glycopeptide FDR cutoff at glycopeptide-spectrum match (GPSM) level was 1%. A sample-specific spectral library (fission yeast SSL, Supplementary Table 1 and Supplementary Data 1) was built using all the 6 h DDA data and one injection of the 1 h DDA data for DIA data analysis by GproDIA (detailed in the “Methods” section). For DIA, results with q-value <5% in each run and q-value < 1% in at least one run at peak group level, as well as q-value < 1% at glycopeptide level in the global context^40^, were reported. The detected glycopeptide precursors, site-specific glycans and protein glycosites are listed in Supplementary Data 2–4, and statistics of the results are shown in Fig. 2 and Supplementary Fig. 3–5. In average, 418 ± 2 (mean ± standard deviation, sic passim) glycopeptide precursors of 348 ± 2 site-specific glycans (corresponding to 153 ± 2 peptides) were detected per replicate run from the DIA data (Fig. 2a), more than those identified from the 1 h DDA (357 ± 16 precursors and 293 ± 9 site-specific glycans corresponding to 150 ± 8 peptides) and the 6 h DDA (351 ± 12 precursors and 289 ± 9 site-specific glycans corresponding to 127 ± 2 peptides). Notably, we use the term “site-specific glycan” referring glycans on specific glycosylation sites in a group of glycoproteins that are not distinguishable by protein inference^17^, rather than positions of glycans on peptide sequences (see the “Methods” section). Indeed, it is not very common that an N-glycopeptide has more than one potential glycosylation sites.

From the 4 DIA replicate runs, 433 glycopeptide precursors of 358 site-specific glycans on 142 protein glycosites (corresponding to 156 peptides of 79 proteins) were detected totally. Among them, 91% (392) precursors, 93% (332) site-specific glycans, and 96% (136) glycosites were shared in all the replicates (Fig. 2b), indicating much fewer missing values than those of 1 h DDA (122/666 = 18% at precursor level, 119/504 = 24% at site-specific glycan level, and 98/190 = 52% at glycosite level) and 6 h DDA (246/461 = 53% at precursor level, 209/370 = 56% at site-specific glycan level, and 102/139 = 73% at glycosite level). Considering identifications shared in >50% (2/3 or 3/4) replicate runs, DIA detected 21% more (418/346) glycopeptide precursors, 20% more (346/288) site-specific glycans, 19% more (139/117) protein glycosites, and 22% more (153/125) peptides than 6 h DDA, as well as 84% more (418/227) precursors, 66% more (346/208) site-specific glycans, 14% more (139/122) protein glycosites, and 20% more (153/128) peptides than 1 h DDA (Fig. 2c). It should be noticed that a less strict error rate control was applied on DDA results (only a GPSM-level FDR cutoff) than that on DIA results (peak group q-value and global glycopeptide q-value). Coefficients of variation (CVs) of glycopeptide precursor, site-specific glycan and protein glycosite quantification results were calculated among the technical replicates as shown in Fig. 2d. The median CVs were ~11% using DIA, much smaller than those using 1 h DDA (~17% at precursor level, ~23% at site-specific glycan level, and ~37% at protein glycosite level) and 6 h DDA (>32% at all levels). We also present the DIA results without multi-run alignment (1% peak group q-value and 1% global glycopeptide q-value) in Supplementary Fig. 6, wherein glycopeptides identification and quantification results close to the ones with multi-run alignment were obtained. The results indicate that the DIA workflow using GproDIA outperforms DDA not due to the multi-run alignment, but originated from the inherent feature of systematic and panoramic MS/MS recording in DIA that provides broadly informative data.

DIA analysis with 1 h gradient can measure more glycopeptides than an 1 h DDA, while it requires DDA runs with longer gradient to build sample-specific libraries. As an alternative to sample-specific libraries, community spectral libraries such as Pan-Human^42^ can be effectively used for peptide-centric DIA data analysis^43^. Such concept is also adequate for glycoproteomics. We tested the feasibility of using a lab repository-scale spectral library (fission yeast LRL, Supplementary Table 1 and Supplementary Data 1) generated by combining the SSL library and fission yeast data of previous projects in our labs. The results are presented in Supplementary Data 5 and Supplementary Fig. 7. Using LRL, 18% more (495/418) glycopeptide precursors, 14% more (394/346) site-specific glycans, and 3% more (143/139) protein glycosites were detected in at least 3 of the 4 replicate runs than using SSL, while the CVs (~11%) were very close to those using SSL.

DIA analysis was also performed on a budding yeast (*Saccharomyces cerevisiae*) sample with 3 repeat injections and an 1 h LC gradient, using a spectral library built from budding yeast DDA data (Supplementary Table 1 and Supplementary Data 1). Low levels of missing values and CVs were also observed (Supplementary Data 6 and Supplementary Fig. 8). All the results suggest that, within the tested condition, DIA with GproDIA improves the number of identifications and reproducibility of glycopeptide characterization compared to DDA-based workflows.

### Inference of glycoforms in wide isolation windows

We further compared DDA and DIA on a human serum sample acquired with 3 repeat injections using the 1 h LC gradient. The DDA results are shown in Supplementary Data 7 and Supplementary Fig. 9. The DIA data were analyzed using a sample-specific spectral library built by DDA with pre-fractionation (serum SSL, Supplementary Table 1 and Supplementary Data 8). Unlike high-mannose-type glycans of yeast, glycans in human serum have more complex compositions, presenting greater challenges for DIA analysis. For yeast samples, glycopeptides with the same peptide sequence and different glycans (HexNAc_2_Hex_*n*_) have mass differences of at least one hexose residue (162 Da), and are hardly co-fragmented in an isolation window (25 *m/z* in this study). For human serum samples, however, DIA analysis suffers from potential interference of glycopeptides with the same peptide sequence but different glycans (referred to as “glycoforms”) in the same isolation window. Therefore, although a large number of glycopeptides were reported by DIA (Supplementary Fig. 10), the results can have high error rates of identification in the glycan part.

**Fig. 3:**
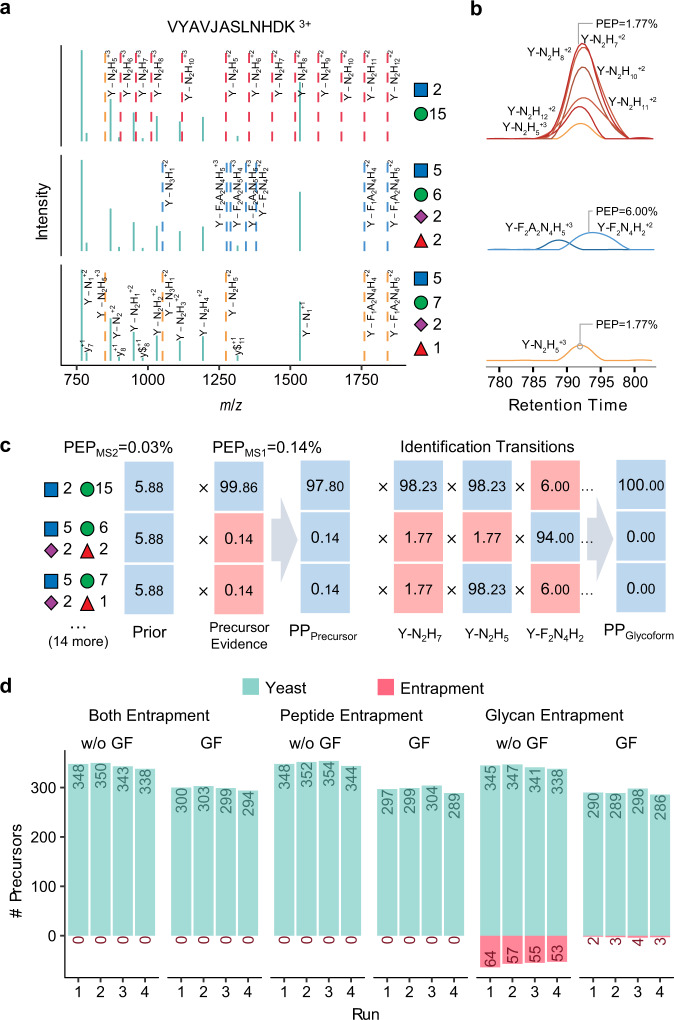
Inference of glycoforms. **a** Generating theoretical Y fragment ions as identification transitions (dashed lines) for the target glycopeptide and all potential glycoforms. “J” in peptide sequence indicates the N-glycosylation site. The glycan symbols are as follows: a green circle or “H” represents Hex; a blue square or “N” represents HexNAc; a red triangle or “F” represents Fuc; a purple diamond or “A” represents NeuAc. **b** Extracting chromatograms of identification transitions from the DIA data and performing transition-level scoring to get the PEPs of transitions. **c** Integrating the precursor and transition PPs to glycoform PPs using a Bayesian hierarchical model. **d** Numbers of identifications without (w/o GF) or with (GF) glycoform inference of the fission yeast sample using the entrapment libraries.

Inspired by IPF^44^, a DIA data analysis tool for peptides carrying PTMs, we further developed an algorithm to evaluate the global FDR (q-value) at glycoform level. The workflow is illustrated in Fig. 3a–c and Supplementary Fig. 11, and described in details in the “Methods” section. In brief, all theoretical Y fragment ions (called “identification transitions”) are generated in silico for each target glycopeptide precursor and the corresponding potential glycoforms within the isolation window when building the spectral library. The potential glycoforms were collected from the pGlyco 2.0 built-in glycan database^17^, containing 3065 glycan structures, and might not be present in the original library. Signals of precursors of target glycopeptides and identification transitions of the target glycopeptides/glycoforms are traced during chromatogram extraction from DIA data. The PEP of MS2 peak groups (PEP_MS2_), precursors (PEP_MS1_) and identification transitions (PEP_transition_) are integrated using a Bayesian hierarchical model (BHM), leading to a glycoform-level posterior probability (PP) for each detected peak group, from which the global FDR (q-value) at glycoform level can be derived.

The performance of the glycoform-level FDR control was tested on the fission yeast data using an entrapment strategy by adding glycopeptides with peptide sequences (peptide entrapment) or glycans (glycan entrapment) or both (both entrapment) from human serum SSL to the fission yeast SSL library. In all the analyses, we ensured that the entrapment glycans were different from the yeast glycans, and kept the number of entrapment glycopeptide precursors similar to that of the yeast library (Supplementary Table 1). Since we focus on investigating the performance of the glycoform-level error rate control, multi-run alignment was not performed, and no global glycopeptide-level q-value filter was applied. The DIA analyses results are presented in Fig. 3d and Supplementary Data 9. With the entrapment library containing human glycopeptides (both entrapment), fewer identifications were observed compared to those using the fission yeast library only, because the entrapment library contains a large fraction of “false targets” that are not detectable in the sample (referred to as π_0_^40^), compromising the detection sensitivity. Nevertheless, no entrapment identifications were observed at 1% peak group-level q-value. Similar results were obtained using the entrapment library containing glycopeptides with peptide sequences from human and glycans from yeast (peptide entrapment), suggesting satisfactory performance of error rate control in the peptide part. Using the entrapment library containing glycopeptides with peptide sequences from yeast and glycans from human (glycan entrapment), although 1% peak group-level q-value filter was applied, there were in average 14% of entrapment identifications (to all of those identified) remaining per run without glycoform inference. We optimized the search space of glycoform inference (Supplementary Note 1 and Supplementary Fig. 12), and the maximum number of potential glycoforms was set to 50 for a trade-off between accuracy and size of search space. After applying 1% glycoform-level q-value filter, despite a loss of 15% yeast identifications due to poor signals of precursors and/or glycoform-specific fragments, the entrapment percentage declined to ~1%.

To the best of our knowledge, the methods for statistical control of glycopeptide error rates in DIA analysis have not been well established. In previous studies, spectral libraries were generated from deglycosylated peptides^28,32^ or peptides with truncated glycans^33^ by shifting the precursor mass, and DIA data analysis of glycopeptides was performed using tools designed for peptide analysis. Therefore, as a baseline for comparison, we built another two glycan entrapment libraries using the peptide sequences from yeast and glycans from human, one without Y ions and the other including Y ions, to analyze the fission yeast sample using the peptide-only FDR control approach with peptide decoys only (Supplementary Note 2, Supplementary Data 9 and Supplementary Fig. 13). The results indicate that the peptide-only FDR approach for peptides cannot address error rate control for glycopeptides properly, which again stresses the significance of comprehensive statistical control by the 2D FDR and glycoform inference.

We further benchmarked the performance of GproDIA on DIA data of 14 synthetic glycopeptides with 7 peptide sequences and 2 sialylated glycans for each sequence (Supplementary Note 3 and Supplementary Table 2). Three fucosylated glycans were generated for each peptide sequence by replacing 1 NeuAc with 2 Fuc monosaccharides (with 1 Da mass difference) and used as entrapment (Supplementary Table 2). DIA was performed with 1 h LC gradient and 3 repeat injections, together with 3 repeat injections of DDA with 1 h LC gradient for library generation. After applying 1% peak group-level q-value and 1% glycoform-level q-value filter, 54 peak groups were reported from the DIA data, including 2 (~4%) entrapment peak groups. From the 3 DIA replicate runs, 13 of the 14 glycopeptides (93% recall) were detected, while 1 entrapment glycopeptide was reported (Supplementary Data 10 and Supplementary Fig. 14). The results demonstrate that the comprehensive statistical control by GproDIA can distinguish glycoforms with near identical masses in a large part.

### Improving glycoproteome coverage in human serum

**Fig. 4:**
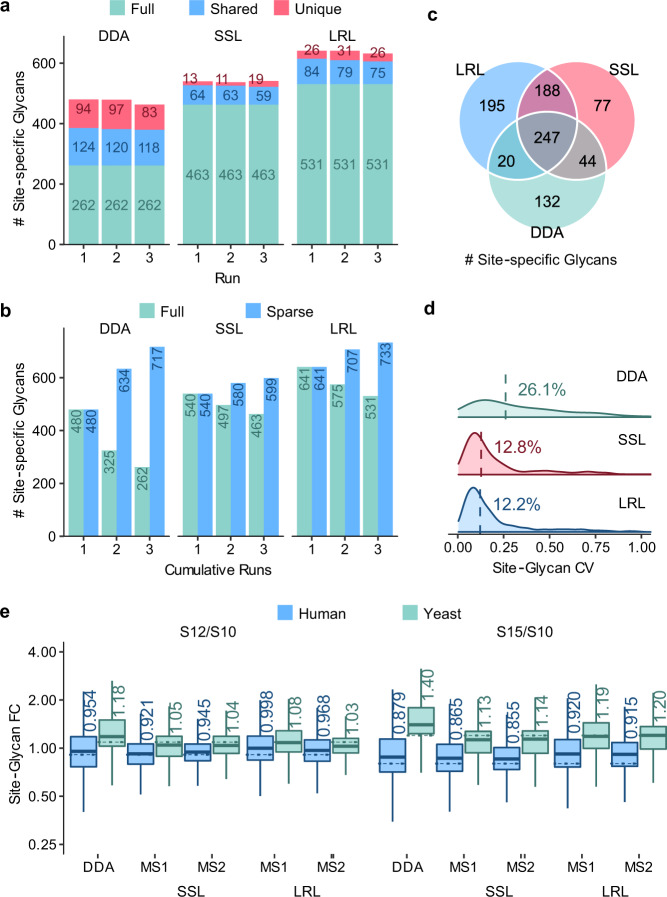
Performance comparison of DDA and DIA on the human serum and mixed-organism samples at the site-specific glycan level. **a** Numbers of identifications from the serum sample per run. “Full” represents identifications observed in all the runs; “shared” represents identifications observed in 2 runs; “unique” represents identifications observed in only 1 run. **b** Numbers of cumulative identifications from the serum sample across runs. “Full” represents identifications shared in the cumulative runs; “sparse” represents identifications observed in at least one run in the cumulative runs. **c** Comparison of numbers of identifications from the serum sample shared in 2/3 runs using DDA, DIA with the sample-specific library (SSL), and DIA with the lab repository-scale library (LRL). **d** Coefficients of variation (CVs) of quantification results. Medians are indicated. **e** Box plot visualization of fold change of the quantification results of the mixed-organism samples. Percent changes were calculated based on the mean quantities in three replicates of each sample. The medians are indicated. The boxes indicate the interquartile ranges (IQR), and whiskers indicate 1.5 × IQR values; no outliers are shown. The dashed lines indicate theoretical fold changes of the organisms (1:0.9:0.8 (S10:S12:S15) for human and 1:1.1:1.2 (S10:S12:S15) for yeast). Source data are provided as a Source Data file.

GproDIA was then tested on the human serum data with glycoform inference enabled. In addition to the sample-specific library (serum SSL), a lab repository-scale spectral library (serum LRL, Supplementary Table 1 and Supplementary Data 8) was also used for DIA data analysis. Global glycopeptide-level q-value cutoff was 1%. After multi-run alignment, results with glycoform-level q-value <5% in each run and <1% in at least one run were finally reported (Supplementary Data 11 and 12, as well as Supplementary Figs. 15 and 16). Comparison between DDA and DIA results is illustrated in Fig. 4a–d. At site-specific glycan level, compared to DDA, DIA using SSL and LRL brought 14% more (539/474) and 35% more (638/474) identifications, respectively, in average per run, whereas fewer missing values (463 shared in all replicates/559 in total using SSL, and 531/733 using LRL) by DIA were observed than DDA (262/717). Considering identifications shared in at least 2 of the 3 replicate runs, DIA using SSL and LRL detected 26% more (556/443) and 47% more (650/443) site-specific glycans, respectively, than DDA. CVs using DIA (12.8% with SSL and 12.2% with LRL) were significantly smaller than that using DDA (26.1%). The performance of statistical control was tested on the human serum data using the entrapment strategy by adding entrapment glycopeptides with peptide sequences from human and glycans from the model higher plant *Arabidopsis thaliana* to the human serum SSL library, where only the plant glycans containing a xylose monosaccharide were used as entrapment, and thus there was no overlap between the human and entrapment glycans. With 1% glycoform-level q-value cutoff, the entrapment percentage declined to 2.4%. Notably, the compositions of the entrapment glycans were very similar to the human glycans except for their core xylose, and this test was aimed at exploring the performance of the statistical control in a worst-case scenario that barely distinguishable glycome is queried against data of complex samples. (Supplementary Note 4, Supplementary Data 13 and Supplementary Fig. 17). We further examined the peaks of oxonium ions in the apex MS2 spectrum of each peak group in the DIA results, and the vast majority of identifications (2395/2398 by the SSL library and 2769/2774 by the LRL library) were supported by the presence of oxonium ions specific to the reported glycan (Supplementary Note 5).

From the human serum data, GproDIA with the serum SSL library detected extra 265 site-specific glycans (corresponding to 322 glycopeptide precursors) that were missed by DDA, considering the identifications shared in 2/3 runs (Fig. 4c). Among them, 236 precursors and 194 site-specific glycans (73%) were validated by targeted MS/MS experiments (Supplementary Note 6 and Supplementary Data 14), and they were distributed throughout the intensity range of DIA quantification results (Supplementary Fig. 18). It should be noticed that we used targeted MS/MS to support the identification results by DIA, which does not indicate that the glycopeptides not observed by targeted MS/MS were wrong.

GproDIA uniquely detected 25 new glycosites, including 11 from new glycoproteins, missed by DDA, by considering the identifications shared in 2/3 runs (Supplementary Fig. 16). Among them, 17 glycosites (9 from new glycoproteins) were supported by targeted MS/MS results, including glycosites on selenoprotein P, proteoglycan 4, plasma protease C1 inhibitor, and alpha-1-antitrypsin. Selenoprotein P is a selenium-containing protein that contributes to antioxidant-mediated protection in colitis-associated cancer^45^. Proteoglycan 4 can protect against the development of osteoarthritis^46^. It has been reported that C1 esterase inhibitor can protect from lung injury^47^, and alpha-1-antitrypsin can inhibit SARS-CoV-2 infection in cell lines and primary cells^48^. The results suggest promising prospects of GproDIA for improving glycoproteome coverage in human serum that boosts disease mechanism study and biomarker discovery.

### Evaluating quantitative accuracy on mixed-organism samples

The performance of GproDIA was further evaluated on data of mixed-organism samples containing glycopeptides from budding yeast and human serum with different abundance (S10, S12, and S15, see the “Methods” section). DIA analysis was performed using a combined library of the budding yeast library and the serum SSL library, as well as a combined library of the budding yeast library and the serum LRL library, respectively (Supplementary Table 1, Supplementary Data 15–17 and Supplementary Figs. 19–21). Based on the mean quantities in three replicates of each sample, fold changes of detected site-specific glycans of samples S12/S10 and S15/S10 were calculated and visualized in Fig. 4e. Fold changes of human and yeast glycopeptide abundance were closer to the theoretical values using DIA-based quantification at both MS1 and MS2 level with the yeast + serum SSL library than those using DDA. Using the yeast + serum LRL library, fold changes of human glycopeptide abundance were overestimated, while fold changes of yeast glycopeptides were measured accurately. In addition, the distribution of fold changes was less dispersed using DIA than that using DDA. All the results underline the high quality of DIA-based glycopeptide characterization using GproDIA that outperforms DDA-based workflows in terms of numbers of identifications, as well as accuracy and precision of quantification.

### Extending library coverage semi-empirically

**Fig. 5:**
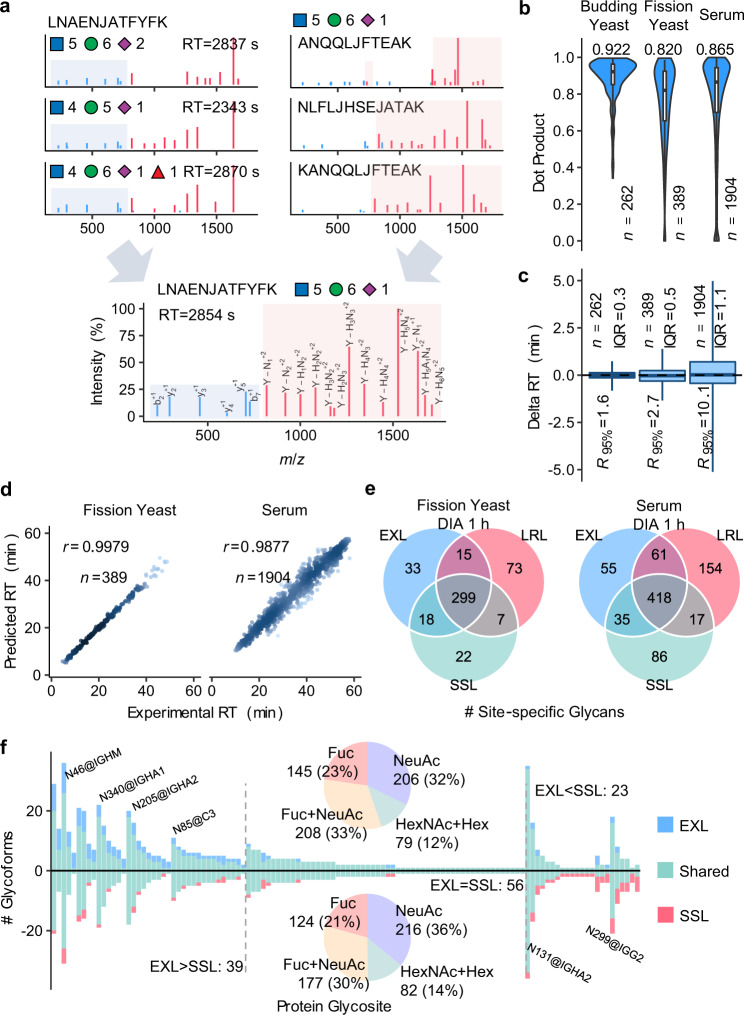
Generating and utilizing semi-empirical spectral libraries. **a** Generating semi-empirical MS/MS spectra by combining the peptide and glycan fragment peaks in the experimental spectra of different glycopeptides. “J” in peptide sequence indicates the N-glycosylation site. The glycan symbols are as follows: a green circle or “H” represents Hex; a blue square or “N” represents HexNAc; a red triangle or “F” represents Fuc; a purple diamond or “A” represents NeuAc. **b** The distributions of dot products computed between the predicted and experimental MS/MS peak intensities. **c** The differences between predicted and experimental retention times (RTs). In (**b**) and (**c**), the middle lines of the boxplots indicate the median, the lower/upper hinges correspond to the first/third quartiles, the lower/upper whiskers extend from the hinges to the 2.5%/97.5% percentiles, while data beyond the end of the whiskers are not shown. **d** Pearson correlation coefficients (*r*) between predicted and experimental RTs. In **b**–**d**, “*n*” indicates the number of generated MS2 spectra or RT values by the prediction method. **e** Comparison of numbers of identifications shared in >50% (2/3 or 3/4) runs using the sample-specific library (SSL), the lab repository-scale library (LRL), and the extended library by the semi-empirical approach (EXL). **f** Comparison of numbers of glycoforms per glycosite detected from serum using SSL and EXL. Proportions of glycan composition types are shown in the pie charts. Glycosites with high variabilities in glycoforms are indicated on the figure. Source data are provided as a Source Data file.

In peptide-centric DIA data analysis, the capability of detection is limited due to the incomplete coverage of spectral libraries. For this reason, we propose a computational approach to expand the coverage of spectral libraries of glycopeptides semi-empirically, wherein the MS2 spectra of predicted glycopeptides are generated by swapping and combining the peptide and glycan fragment peaks in experimental spectra of different glycopeptides using a *k*-nearest neighbor (KNN) strategy (Fig. 5a). The RT of the predicted glycopeptide is the weighted mean RT of experimentally identified glycopeptides with the same peptide sequences and close monosaccharide compositions by the KNN strategy. Details are described in the “Methods” section.

Cross validations of the prediction were conducted using the fission yeast LRL library and the budding yeast library, wherein the library entry of each glycopeptide precursor was pulled out from the library, and meanwhile the rest of the library entries were used to generate the predicted library entry of the glycopeptide precursor. Different numbers of nearest neighbors (*k*) were tested. Dot products (DPs) were computed between the predicted and experimental MS/MS intensities (Supplementary Figs. 22 and 23). With *k* = 3, the median DP was 0.820 for the fission yeast library and 0.922 for the budding yeast library, higher than those without KNN and with *k* = 2 or 4. Increasing *k* can lead to higher prediction accuracy, but fewer glycopeptides can be predicted because at least *k* neighboring entries for generating the peptide part and the glycan part are required in the experimental library. Therefore, we chose *k* = 3 to achieve the trade-off between prediction accuracy and library coverage. With *k* = 3, the interquartile ranges (IQRs) of the differences between predicted and experimental RTs were <0.6 min. Pearson correlation coefficients (*r*) of predicted and experimental RTs were >0.99 (Fig. 5d, Supplementary Fig. 22 and 23). The serum SSL library was also used for the cross validation with *k* = 3. The median DP was 0.865 (Fig. 5b), the IQR of RT differences was 1.1 min (Fig. 5c), and the *r* of RTs was >0.98 (Fig. 5d). The prediction on human serum glycopeptides is less accurate compared to yeast due to the high complexity of human serum glycopeptides. DPs, RT differences and *r* were also computed among replicate experimental spectra, which can be considered as possible upper limit of prediction accuracy (Supplementary Fig. 24), showing that there are still rooms for improvement. Nevertheless, due to the limit of current available data of glycopeptides and the highly complex isomeric glycan structures, more accurate prediction by machine learning and deep learning strategies can hardly be applied at this stage.

Next, we built a semi-empirical library based on the fission yeast SSL library, which was then merged with the SSL to form an extended library (fission yeast EXL, Supplementary Table 1 and Supplementary Data 1), containing 331 extra precursors and 288 site-specific glycans that are not present in the SSL (Supplementary Fig. 25). From the fission yeast DIA data, 5% more (365/346) site-specific glycans were detected in at least 3 of the 4 runs using EXL than those using SSL, while the CVs stayed unchanged approximately (Fig. 5e, Supplementary Fig. 26 and Supplementary Data 18). Performance of error rate control when using extended libraries was also evaluated using the entrapment strategy (Supplementary Fig. 27 and Supplementary Data 19).

An extended library (serum EXL, Supplementary Table 1 and Supplementary Data 8) was also generated from the serum SSL library. To avoid combinatorial explosion of peptides and glycans, we collected a list of glycopeptides combined from the serum LRL library and a publication on N-linked intact glycopeptides in human serum^49^, and only peptide-glycan combinations in the glycopeptide list were taken into consideration when generating the extended library. Consequently, the EXL library containing 1990 extra precursors and 927 site-specific glycans that are not present in the serum SSL (Supplementary Fig. 25). Combining all the 3 replicates, 118 protein glycosites were detected in total using both the SSL and EXL libraries. Among them, on 39 glycosites, more glycoforms were detected using EXL than using SSL, while on 23 glycosites, more glycoforms were detected using SSL (Fig. 5f, Supplementary Data 20). The increase in the detected glycoforms can be attributed to the greater proportion of fucosylated glycans (145 site-specific glycans with fucosylation and without sialylation, as well as 208 with both fucosylation and sialylation) detected using EXL than using SSL (124 with fucosylation and without sialylation, as well as 177 with both fucosylation and sialylation). It should be noted that the semi-empirical approach does not increase the protein glycosites. The CVs stayed unchanged approximately using the human serum EXL (Supplementary Fig. 28).

## Discussions

With more efficient usage of ions, DIA can provide a significant increase in the identification efficiency of glycopeptides compared to DDA^27,50^. We have benchmarked our DIA-based workflow against the DDA-based glycoproteomics, demonstrating that short-gradient DIA can outperform DDA under the same conditions or even with a much longer gradient in terms of detectable glycopeptides as well as measurement reproducibility. It has been reported that DIA copes well with shortening of the LC gradient length because the deterministic nature of the MS2 sampling in DIA attenuates the attrition in number of identifications for shorter separation gradients, while the number of acquired MS/MS spectra and identifications decrease proportionally with the gradient length in DDA mode^26^. As a less time-consuming approach for intact glycopeptides profiling, short-gradient DIA is favorable for large-scale quantitative glycoproteomic analyses.

Interference from other co-eluted and co-fragmented glycopeptides is the main challenge for DIA data analysis that may result in a high level of misinterpretations. In GproDIA, we implement a 2-dimensional FDR approach and a glycoform inference strategy, providing comprehensive statistical control for glycopeptide identification. For the peptide part of the 2-dimensional FDR approach, the reverse decoy approach was inherited from the DIA analysis method for non-glycosylated peptides. For the glycan part, random mass shifts were performed on the glycan fragment peaks, which was initially reported in pGlyco^38^. In the 2-dimensional FDR approach, sufficient numbers of training data are necessary for the semi-supervised algorithm. Also, the decoy distribution should match the true-negative part of the target distribution. If the spectral library is too small, these conditions may not be fulfilled, and the machine learning and FDR estimation may be biased (Supplementary Note 7). In this study, however, we used the entrapment strategy to assess the quality of statistical control. Even for the synthetic dataset, which is the smallest dataset in the study, the false positives were still well controlled. Glycoform inference can be used as an option to perform more strict assessment by utilizing signals of precursors and glycan-specific fragments to resolve interference from potential glycoforms. In this study, glycoform inference was enabled when multiple glycopeptide precursors with the same peptide sequence and different glycans are arranged to be fragmented in one isolation window. Despite the herein proposed strategies to control error rates when using wide isolation windows, we still recommend to design the isolation windows properly according to the mass distribution of glycopeptides^32^, if possible, for improving the detection sensitivity. Recent advances in ion mobility spectrometry (IMS) including high field asymmetric waveform ion mobility spectrometry (FAIMS)^51^ and parallel accumulation-serial fragmentation (diaPASEF)^52^ have achieved rapid improvements in the sensitivity of DIA analysis. We expect that DIA-based glycopeptides profiling can benefit from the enhanced separation of glycoforms by IMS.

Spectral library coverage determines the upper limit of the identification capacity by peptide-centric DIA analysis. To date, the majority of DIA studies have used DDA-based sample-specific spectral libraries, frequently with pre-fractionation (for the serum sample in this study), or sometimes by repeated DDA analysis of non-fractionated samples (for the fission yeast sample in this study)^26^. Other sources, such as previously assembled repositories shared by the community, can also be considered as a supplement for library completeness^43^. We achieved the best coverage by using repository-scale libraries integrating data from multiple previous projects in our lab. In the context of DIA data analysis, FDR control is performed on the features extracted from the DIA data, rather than entries in the spectral library. Despite the accumulation of false entries when combining multiple spectral libraries, FDR of the DIA results can still be controlled, unless it is in an extreme case of using very huge and heterogeneous spectral libraries. We envision that more “off-the-shelf” glycopeptide libraries will be built and published by the community, just like those for proteomic studies^42^, which can then be used for glycopeptide DIA data analysis.

Since repository-scale libraries may not always be available, we have built semi-empirical libraries as an attempt to extend the library coverage. The current proposed strategy facilitates detection of more glycoforms by DIA on protein glycosites that are observed by DDA, but cannot increase the coverage at glycosite level. However, deep learning-based tools such as Prosit^53^ and DeepDIA^54^ have been developed for generating in silico peptide spectral libraries directly from peptide sequences, and we anticipate that predicted libraries will break through the limitation on coverage of glycopeptide libraries by DDA in the future when large scale glycopeptide libraries are built by the community for training the deep neural network models.

Since our proposed method is based on the concept of peptide-centric analysis, it inherits the general limitation of working with very large-scale spectral library resources, e.g., proteome-wide predicted libraries. In such analyses, it is common that a significant fraction of glycopeptides are actually not present in the samples at a detectable level, and the large query space may increase the multiple testing burden and compromise detection sensitivity^26^. Therefore, instead of enumerating all the peptide-glycan combinations in a spectral library, we suggest researchers focusing on a subset of glycoproteins/glycoproteoforms of interest for their specific biological questions. Another issue is the localization of glycosite. Our proposed method is demonstrated here in the context of N-glycoproteomics, where it is not very common that more than one potential glycosylation site exist in a glycopeptide. Although the principle of comprehensive statistical control in GproDIA should also be applicable to O-glycopeptides for peptide sequence and glycan identification, O-glycopeptides generally have multiple serine and/or threonine residues that serve as potential glycosites, which calls for site-specific glycoproteome profiling. Unfortunately, HCD is not sufficient for glycosite localization for O-glycopeptides^10^. ETD-based DIA methods may solve the problems of glycosite localization, but could also suffer from limited fragments information for both peptide and glycan parts. We would expect development in instruments, experimental methods and data analysis approaches for site-specific O-glycopeptide analyses.

## Methods

### Term usage definition

Unless otherwise specified, when we use the term “glycan” in this study, we refer to monosaccharide compositions, ignoring isomeric glycan structures. When we use the term “glycopeptide precursor”, we refer to a precursor with specific peptide sequence, modifications (other than glycosylation), glycan composition and charge state, ignoring positions of the glycan on the peptide sequence. Indeed, it is not very common that an N-glycopeptide has more than one potential glycosylation sites. When we use the term “glycopeptide”, we refer to a group of glycopeptide precursors with the same peptide sequence and glycan composition. These precursors may contain different modifications (other than glycosylation) and distinct charge states. When we use the term “protein glycosite”, we refer to a group of glycoproteins with a corresponding glycosylation site on each protein. These glycoproteins are not distinguishable by protein inference in DDA database searching. We did not perform extra protein inference, and just used the protein glycosylation site groups determined by pGlyco3 when building spectral libraries. When we use the term “site-specific glycan”, we refer to a glycan composition on a “protein glycosite”, which contains a group of glycopeptide variants covering the same “protein glycosite” resulting from missed cleavages in protein digestion.

We use the term “q-value” to refer to global FDR, and “global q-value” to refer to q-value in the global context. Global FDR is a concept opposite to local FDR (PEP). Whereas local FDR is the probability of each target peak group to be incorrect, global FDR conveys the error introduced in the whole reported results. Q-value in the global context is a different concept. In contrast to the run-specific context that conducts separate error rate estimation for each run, the global context only considers the best-scoring peak group per analyte across the entire experiment^40^. Visualizations of these concepts are shown in Supplementary Fig. 29.

GproDIA uses DDA results by pGlyco3 for spectral library generation. For compatibility purposes, GproDIA uses the nomenclature of monosaccharides defined by the pGlyco series^17,18^, which is associated with but slightly different from the standard Symbol Nomenclature for Glycans (SNFG). The nomenclature is listed in Supplementary Table 3.

### Yeast sample preparation

*Saccharomyces cerevisiae* (budding yeast) and *Schizosaccharomyces pombe* (fission yeast) were cultured in yeast extract peptone dextrose (YPD) and yeast extract with supplements (YES) medium, respectively, at 30 °C until optical density at 600 nm (OD_600_) of 0.6 was reached. Then the yeasts were harvested by centrifugation at 1000 *g*, and washed twice with a 10 mM Tris/HCl buffer (pH = 7). Cells were then snap-frozen in liquid nitrogen, ground to a fine powder with mortar and pestle in liquid nitrogen, and stored at −80 °C until use. The grinding powder was processed to protein extraction. The powder was dissolved in fivefold-volume lysis buffer (4% sodium dodecyl sulfate (SDS), 0.1 M Tris/HCl, pH 8.0) with protease inhibitor (1 mM phenylmethanesulfonyl fluoride (PMSF), 1 mM cocktail), followed by boiling at 100 °C for 10 min, ultrasonication for 5 min and centrifugation at 12,000 × *g* at 18 °C for 30 min to collect protein extracts. The protein concentration was determined by bicinchoninic acid (BCA) method. A starting amount of 100 μg protein was used for one LC-MS/MS analysis, which was afterwards subjected to proteolysis and glycopeptide enrichment.

Proteins were reduced in 10 mM dithiothreitol (DTT) at 57 °C for 30 min, and then alkylated in dark by 20 mM iodoacetamide (IAA) at room temperature for 30 min. After carbamidomethylation, six volumes of acetone were added to precipitate the proteins at −20 °C for at least 3 h. The precipitates were dissolved in a denaturing buffer (8 M urea in 50 mM NH_4_HCO_3_) following a tenfold dilution with 50 mM NH_4_HCO_3_. Trypsin was added to a final enzyme-to-substrate ratio of 1:50 (wt/wt) and incubated at 37 °C overnight. The reaction was terminated by adding 0.5% trifluoroacetic acid (TFA). All digested samples were centrifuged at 16,000 × *g* for 10 min and the supernatants were desalted using the Sep-Pak C18 cartridges (Waters, USA). Briefly, the samples were loaded into the cartridge, which was preconditioned with 1 mL ACN once and 500 μL 0.1% TFA twice. Then, the Sep-Pak C18 cartridge was washed with 500 μL 0.1% TFA twice, and eluted with 250 μL 30% ACN containing 0.1% TFA and 250 μL 70% ACN containing 0.1% TFA, successively. The desalted peptides were then dried by vacuum centrifugation and used for glycopeptide enrichment.

### Human serum sample preparation

Human serum sampleswere collected under the consent of the donors. The research followed the tenetof the Declaration of Helsinki, and the protocol of blood collection, processingand MS analysis was approved by the Ethics Committee of Fudan University, andcomplied with all relevant laws and regulations of China.

A total of 40 human serum samples were collected. The collected blood samples were immediately placed on ice for 30 min and then centrifuged at 2000 × *g* for 15 min. The supernatant was collected and stored at −80 °C until use.

A pooled serum specimen mixed from equal volume of the above samples was used in this study. A volume of 20 μL of the pooled serum was added to 180 μL of 50 mM NH_4_HCO_3_, and placed in boiling water and ice 40 times alternately to denature the proteins. The proteins were then reduced with 10 mM DTT at 37 °C for 1 h and alkylated with 20 mM IAA for 0.5 h at room temperature in the dark. Then the proteins were digested into peptides and desalted using the same protocol as that for yeasts.

### Fractionation with high-pH reversed-phase chromatography

For spectra library building, the peptide digests from serum samples were fractionated by high pH reverse phase (RP) LC separation using a Waters UPLC system coupled with a C18 column (Waters BEH C18, 2.1×150 mm, 1.7 μm). The digests were re-dissolved in phase A (2% acetonitrile (ACN), 1% NH_3_·H_2_O, 97% H_2_O). The column flow rate was maintained at 0.2 mL min^−1^ and the column temperature was maintained at 45 °C. The gradient was as follow: 0% B (90% ACN, 1% NH_3_·H_2_O, 9% H_2_O) for 2 min, 0–5% B in 4 min, 5–10% B in 9 min, 10–25% B in 35 min, 25–35% B in 7 min, 35–50% B in 4 min, 50–90% B in 1 min, 90% B for 2 min, return to 0% in 0.1 min, and hold until the end of gradient. Twenty fractions were collected from the 3rd minute to the 64th minute within each 1 minute in turn as the following conditions: fraction 1 (3, 23, 43, 63); fraction 2 (4, 24, 44); … fraction 20 (22, 42, 62). Each fraction was dried in a vacuum concentrator and used for glycopeptide enrichment.

### Glycopeptide enrichment

Glycopeptides were enriched using a ZIC-HILIC method with minor modification^17^. The desalted peptides of 1 mg were resuspended in 300 μL loading buffer containing 80% ACN and 1% TFA and then loaded onto an in-house micro-column containing 50 mg of ZIC-HILIC particles (Merck Millipore, Darmstadt, Germany) packed onto a C8 disk. The flow through was collected and reloaded onto the column for additional four times. Then, the column was washed with 200 μL loading buffer for four times. Finally, the glycopeptides that have been enriched in the column were collected by elution with 600 μL 0.1% TFA and dried by vacuum centrifugation.

### Mixed-organism sample preparation

Glycopeptides enriched from human serum and budding yeast were resuspended in 0.1% formic acid (FA). Two mg serum proteins were used as starting material and finally resuspended in 80 μL 0.1% FA after digestion and ZIC-HILIC enrichment, while 3 mg yeast proteins in 120 μL 0.1% FA. Then the samples were mixed at definite ratios: (i) sample S10, 1:1 (16 μL:16 μL, human: yeast); (ii) sample S12, 1:1.2 (16 μL:19.2 μL, human: yeast); (iii) sample S15, 1:1.5 (16 μL:24 μL, human: yeast). Four μL of each mixture was subjected to LC-MS/MS analysis. Consequently, the final concentration ratio was 1:0.9:0.8 (S10:S12:S15) for human and 1:1.1:1.2 (S10:S12:S15) for yeast.

### LC-MS/MS analysis

Intact glycopeptides were detected using a nanospray LC-MS/MS on an Orbitrap Fusion Tribrid system (Thermo Fisher Scientific, Waltham, MA, USA) fitted with an EASY-nLC TM1100 system (Thermo Fisher Scientific, Waltham, MA, USA) that included a reverse-phase analytical column without the trap column. Samples were loaded onto a C18 column (50 cm × 75 μm i.d.) and separated at a flow rate of 300 nL min^−1^. Solvent A was a 0.1% FA aqueous solution. Solvent B was 80% ACN containing 0.1% FA. Two LC gradients were used in this study: (i) 1 h in total, 5–10% B in 3 min, 10–40% B in 42 min, 40–60% in 5 min, followed by an increase to 90% B in 3 min, hold for another 1.5 min, return to 5% B in 10 s and hold for the last 5 min; (ii) 6 h in total, 1–30% in 330 min, 30–45% B in 15 min, followed by an increase to 90% B in 1 min, hold for another 7 min, return to 1% B in 10 s and hold for the last minutes. For DDA, the fission yeast sample was analyzed using the 1 h and 6 h gradients, while the other samples were only analyzed using the 1 h gradient. For DIA, the 1 h gradient was always used.

Data collection was performed by Thermo Xcalibur (version 3.0.63). The parameters for DDA MS analysis was: (1) MS: scan range (*m/z*) = 700–2000; resolution = 120,000; AGC target = 500,000; maximum injection time = 50 ms; included charge state = 2–6; dynamic exclusion after *n* times, *n* = 1; dynamic exclusion duration = 15 s; the precursors were selected under the “top speed” mode and each selected precursor was subject to one HCD-MS/MS; (2) HCD-MS/MS: isolation window = 4; detector type = Orbitrap; resolution = 15,000; AGC target = 500,000; maximum injection time = 250 ms; collision energy = 30%; stepped collision mode on, energy difference of ±10% (10% as absolute value in the Orbitrap Fusion). The acquisition cycle time was set as 3 s, which was used to determine the frequency of full scans with the maximum number of data-dependent scans in the “top speed” mode.

DIA was performed with 40 isolation windows from 700 Da to 1636 Da (center) with 25 Da width and 1 Da overlap. The acquisition cycle time was set as 3 s, the maximum injection time was set as 50 ms, and the AGC target (the total number of ions) was set as 2.0e5. The other MS parameters were the same as those in DDA.

### Database searching of DDA data

The raw DDA data files were searched using pGlyco3^18^ (version 3.0.rc1). Mass tolerances for the precursors and fragment ions were set as ±4 ppm and ±20 ppm, respectively. Two missed cleavages were allowed for trypsin digestion. The fixed modification was carbamidomethylation of all cysteine residues (+57.02 Da). Variable modifications included oxidation of methionine (+15.99 Da) and acetylation on protein N-term (+42.01 Da). The protein databases downloaded from Swiss-Prot/UniProt (https://www.uniprot.org/, access date 2018-08) were used for each sample after replacing the “N” in the sequon N-X-S/T/C (X ≠ P) with “J” (as potential glycosites) in all protein sequences: (i) for the fission yeast sample, the protein database with species of *S. pombe* (5140 entries) was used; (ii) for the budding yeast sample, the protein database with species of *S. cerevisiae* (6721 entries) was used; for the human serum sample, the protein database with species of *Homo sapiens* (20,398 entries) was used; (iii) for the mixed organism samples, the protein databases with species of *S. cerevisiae* and *H. sapiens* were combined; (iv) for the synthetic glycopeptide sample, a protein database containing 11 entries from *H. sapiens* and *Mus musculus* corresponding to the synthetic peptide sequences was used. A glycan database containing 2922 entries (726 glycan compositions) embedded in pGlyco3 was used for the human, yeast and mixed organism samples. A self-defined glycan database containing 9 entries (5 glycan compositions) of the synthetic glycopeptides and entrapments was used for the synthetic data. GPSM-level FDR cutoff was applied at 1% for quality control.

### Spectral library building

For each GPSM, pGlyco3 reported a plausible glycan structure in canonical form. GPSMs of each identified glycopeptide may be annotated with different plausible glycan structures. For each plausible glycan structure, the identification scores (total scores in pGlyco3) of corresponding GPSMs were summed, and only the GPSMs annotated with the glycan structure with the highest sum identification score were considered as confident identifications.

For each of these confident identifications from DDA data, the corresponding MS/MS spectrum was matched with *m/z* of theoretical fragment ions of intact glycopeptides in SCE-HCD^17,38^: (i) naked peptide (Y_0_) with charge states 1+ to 3+; (ii) peptide backbone with one HexNAc attached (Y_1_) and its corresponding cross-ring fragmentation on the HexNAc residue (^0,2^X_0_, denoted as Y_$_ for simplicity) with charge states 1+ to 3+; (iii) other Y ions with charge states 1+ to 3+; (iv) naked peptide backbone b and y fragment ions with charge states 1+ and 2+; (v) b/y ions with one HexNAc (b-N_1_/y-N_1_) and cross-ring fragment of HexNAc (b_$_/y_$_) with charge states 1+ and 2+. The matched peak intensities were extracted and imported into a spectral library. Each library entry contained the identification (peptide sequence, glycan, charge state, other modifications if any), the retention time (RT), the precursor *m/z*, and the annotated fragment peak list.

The generated spectral library contained non-unique entries (replicates) resulting from multiple GPSMs of the same glycopeptide precursor. Where available, replicates were combined to create a “consensus” spectrum^55^ through a series of steps: (i) pairwise dot products (DPs) were calculated among replicates, and dissimilar replicates (with median DP < 0.5 to the other replicates) were discarded; (ii) only peaks present in >60% of the replicates were kept; (iii) the consensus *m/z* and intensities were calculated as averages of the corresponding peaks in the replicates weighted by the identification scores.

Transitions with fragment ion *m/z* out of the scan range or falling within the isolation window of their precursor *m/z*^56^ were excluded from the library. Up to 10 + 10 most intense peptide/glycan transitions per precursor were kept, respectively, and the top 6 + 6 were used for quantification. Precursors with <3 peptide transitions or <3 glycan transitions were excluded.

In each DDA run with long LC gradient and/or pre-fractionation, RT values of glycopeptides were calibrated to the single shot 1 h LC DDA run using the shared identifications as anchors. Locally weighted scatterplot smoothing (LOWESS) was used to scale the RT values into the 1 h LC gradient space. Transitions of the anchors were saved as TraML file, which was used by OpenSWATH in the chromatographic extraction step.

### Decoy generation

The decoy peptides were generated in silico from the target peptide list by reversing the amino acid sequence (while keeping the C-terminal arginine and lysine unmoved^26^). For glycans, a spectrum-based decoy method^38^ was used to generate decoy glycan spectra by adding a random mass shift ranging from 1 to 30 Da to the mass of each Y ion (except Y_0_ and Y_$_). A peptide decoy library containing glycopeptide precursors with decoy peptides and target glycans, a glycan decoy library containing precursors with target peptides and decoy glycans, and a both decoy library containing precursors with decoy peptides and decoy glycans were appended to the target library. The same number of decoys were generated as targets for each decoy type, and the size of the final combined library was 4 times the size of the target library.

### Chromatographic extraction from DIA data

The raw DIA data files were converted to mzML format using MSConvert from ProteoWizard (version 3.0.11537) with the peak picking algorithm set to vendor. Then the precursor and fragment ion chromatograms of the glycopeptides in the spectral library were extracted from the mzML files using OpenSWATH (version 2.6.0)^39^. The MS1 and MS2 *m/z* extraction windows were set to 10 ppm and 20 ppm, respectively. The anchor TraML file generated in the spectral library building step was used as reference glycopeptides for retention time normalization, which was similar to the iRT approach^37^. The RT alignment method was set to LOWESS.

### Peak group scoring and statistical control

Semi-supervised learning was conducted to compute discriminant scores (D-scores) of the extracted candidate peak groups using PyProphet (version 2.1.5)^40,57^. Peptide D-scores were computed using the target peak groups and the glycan decoy peak groups as “targets”, while the peptide decoy peak groups and the both decoy peak groups as “decoys”. Glycan D-scores were computed using the target peak groups and the peptide decoy peak groups as “targets”, while the glycan decoy peak groups and the both decoy peak groups as “decoys”. Linear discriminant analysis (LDA) was used to calculate a weighted linear combination of the peptide and glycan D-scores (combined D-scores) to separate targets and decoys. For each queried glycopeptide precursor, the peak group with the highest combined D-score was then considered as the most likely detected peak group.

Error rates of identification were estimated using a 2-dimentional FDR approach. The distributions of peptide and glycan D-scores (*s*_P_ and *s*_G_) were estimated using a bivariate four-groups mixture model^58^. The bivariate density of the targets (*f*_TT_) is given by

1 $${f}_{{{\mbox{TT}}}}\left({s}_{P},{s}_{G}\right)={\pi }_{00}{f}_{00}\left({s}_{{{{{{\rm{P}}}}}}},{s}_{{{{{{\rm{G}}}}}}}\right)+{\pi }_{01}{f}_{01}\left({s}_{{{{{{\rm{P}}}}}}},{s}_{{{{{{\rm{G}}}}}}}\right)+{\pi }_{10}{f}_{10}\left({s}_{{{{{{\rm{P}}}}}}},{s}_{G}\right)+{\pi }_{11}{f}_{11}\left({s}_{{{{{{\rm{P}}}}}}},{s}_{{{{{{\rm{G}}}}}}}\right)$$

where π_00_ is the proportion of target peak groups for which both peptide and glycan are null, π_01_ is the proportion where peptide is null and glycan is non-null, π_10_ is the proportion where peptide is non-null and glycan is null, π_11_ is the proportion where both peptide and glycan are non-null, and they satisfy that

2 $${\pi }_{00}+{\pi }_{01}+{\pi }_{10}+{\pi }_{11}=1$$

*f*_ij_ (i = 0 or 1, j = 0 or 1) is the corresponding null or non-null density. In the glycan decoys, all the glycans are null, and the proportion where peptide is null is π_00_ + π_01_. Thus, the bivariate density of the glycan decoys (*f*_TD_) is

3 $${f}_{{{\mbox{TD}}}}\left({s}_{{{{{{\rm{P}}}}}}},{s}_{{{{{{\rm{G}}}}}}}\right)=\left({\pi }_{00}+{\pi }_{01}\right){f}_{00}\left({s}_{{{{{{\rm{P}}}}}}},{s}_{{{{{{\rm{G}}}}}}}\right)+\left({\pi }_{10}+{\pi }_{11}\right){f}_{10}\left({s}_{{{{{{\rm{P}}}}}}},{s}_{{{{{{\rm{G}}}}}}}\right)$$

Similarly, the bivariate densities of the peptide decoys (*f*_DT_) and the both decoys (*f*_DD_) are

4 $${f}_{{{\mbox{DT}}}}\left({s}_{{{{{{\rm{P}}}}}}},{s}_{{{{{{\rm{G}}}}}}}\right)=\left({\pi }_{00}+{\pi }_{10}\right){f}_{00}\left({s}_{{{{{{\rm{P}}}}}}},{s}_{{{{{{\rm{G}}}}}}}\right)+\left({\pi }_{01}+{\pi }_{11}\right){f}_{01}\left({s}_{{{{{{\rm{P}}}}}}},{s}_{{{{{{\rm{G}}}}}}}\right)$$

5 $${f}_{{{\mbox{DD}}}}\left({s}_{{{{{{\rm{P}}}}}}},{s}_{{{{{{\rm{G}}}}}}}\right)={f}_{00}\left({s}_{{{{{{\rm{P}}}}}}},{s}_{{{{{{\rm{G}}}}}}}\right)$$

Therefore,

6 $${f}_{01}\left({s}_{P},{s}_{G}\right)=\frac{{f}_{{{\mbox{DT}}}}\left({s}_{{{{{{\rm{P}}}}}}},{s}_{{{{{{\rm{G}}}}}}}\right)-\left({\pi }_{00}+{\pi }_{10}\right){f}_{{{\mbox{DD}}}}\left({s}_{{{{{{\rm{P}}}}}}},{s}_{{{{{{\rm{G}}}}}}}\right)}{{\pi }_{01}+{\pi }_{11}}$$

7 $${f}_{10}\left({s}_{{{{{{\rm{P}}}}}}},{s}_{{{{{{\rm{G}}}}}}}\right)=\frac{{f}_{{{\mbox{TD}}}}\left({s}_{{{{{{\rm{P}}}}}}},{s}_{{{{{{\rm{G}}}}}}}\right)-\left({\pi }_{00}+{\pi }_{01}\right){f}_{{{\mbox{DD}}}}\left({s}_{{{{{{\rm{P}}}}}}},{s}_{{{{{{\rm{G}}}}}}}\right)}{{\pi }_{10}+{\pi }_{11}}$$

In the target peak groups, the proportion for which peptide is null is

8 $${\pi }_{{{{{{\rm{P}}}}}}}={\pi }_{00}+{\pi }_{01}$$

which can be estimated from the peptide D-score distributions by Storey’s method^59^, using the target peak groups and the glycan decoy peak groups as “targets”, while the peptide decoy peak groups and the both decoy peak groups as “decoys”. Similarly, the proportion for which glycan is null is

9 $${\pi }_{{{{{{\rm{G}}}}}}}={\pi }_{00}+{\pi }_{10}$$

which can be estimated from the glycan D-score distributions using the target peak groups and the peptide decoy peak groups as “targets”, as well as the glycan decoy peak groups and the both decoy peak groups as “decoys”. π_00_ can be estimated from the combined D-score distributions using the targets and the both decoys. With the estimated values of π_00_, π_01,_ π_10_ and π_11_, the *f*_ij_ of each target peak group can be derived by using Eqs. (1), (3), (4), (5) to calculate the local FDR, which is given by

10 $${{{{{{\rm{PEP}}}}}}}_{{{{{{\rm{P}}}}}}}\left({s}_{{{{{{\rm{P}}}}}}},{s}_{{{{{{\rm{G}}}}}}}\right)=\frac{{\pi }_{00}{f}_{00}\left({s}_{{{{{{\rm{P}}}}}}},{s}_{{{{{{\rm{G}}}}}}}\right)+{\pi }_{01}{f}_{01}\left({s}_{{{{{{\rm{P}}}}}}},{s}_{{{{{{\rm{G}}}}}}}\right)}{{f}_{{{\mbox{TT}}}}\left({s}_{{{{{{\rm{P}}}}}}},{s}_{{{{{{\rm{G}}}}}}}\right)}$$

where the peptide part of a target peak group is null,

11 $${{{{{{\rm{PEP}}}}}}}_{{{{{{\rm{G}}}}}}}\left({s}_{{{{{{\rm{P}}}}}}},{s}_{{{{{{\rm{G}}}}}}}\right)=\frac{{\pi }_{00}{f}_{00}\left({s}_{{{{{{\rm{P}}}}}}},{s}_{{{{{{\rm{G}}}}}}}\right)+{\pi }_{10}{f}_{10}\left({s}_{{{{{{\rm{P}}}}}}},{s}_{{{{{{\rm{G}}}}}}}\right)}{{f}_{{{\mbox{TT}}}}\left({s}_{{{{{{\rm{P}}}}}}},{s}_{{{{{{\rm{G}}}}}}}\right)}$$

where the glycan part of a target peak group is null, and

12 $${{{{{{\rm{PEP}}}}}}}_{{{{{{\rm{P}}}}}}\cap {{{{{\rm{G}}}}}}}\left({s}_{{{{{{\rm{P}}}}}}},{s}_{{{{{{\rm{G}}}}}}}\right)=\frac{{\pi }_{00}{f}_{00}\left({s}_{{{{{{\rm{P}}}}}}},{s}_{{{{{{\rm{G}}}}}}}\right)}{{f}_{{{\mbox{TT}}}}\left({s}_{{{{{{\rm{P}}}}}}},{s}_{{{{{{\rm{G}}}}}}}\right)}$$

where both the peptide and glycan parts of a target peak group are null. The total PEP or local FDR is

13 $${{{{{{\rm{PEP}}}}}}}_{{{{{{\rm{P}}}}}}\cup {{{{{\rm{G}}}}}}}\left({s}_{{{{{{\rm{P}}}}}}},{s}_{{{{{{\rm{G}}}}}}}\right) =\frac{{\pi }_{00}{f}_{00}\left({s}_{{{{{{\rm{P}}}}}}},{s}_{{{{{{\rm{G}}}}}}}\right)+{\pi }_{01}{f}_{01}\left({s}_{{{{{{\rm{P}}}}}}},{s}_{{{{{{\rm{G}}}}}}}\right)+{\pi }_{10}{f}_{10}\left({s}_{{{{{{\rm{P}}}}}}},{s}_{{{{{{\rm{G}}}}}}}\right)}{{f}_{{{\mbox{TT}}}}\left({s}_{{{{{{\rm{P}}}}}}},{s}_{{{{{{\rm{G}}}}}}}\right)}\\ ={{{{{{\rm{PEP}}}}}}}_{{{{{{\rm{P}}}}}}}\left({s}_{{{{{{\rm{P}}}}}}},{s}_{{{{{{\rm{G}}}}}}}\right)+{{{{{{\rm{PEP}}}}}}}_{{{{{{\rm{G}}}}}}}\left({s}_{{{{{{\rm{P}}}}}}},{s}_{{{{{{\rm{G}}}}}}}\right)-{{{{{{\rm{PEP}}}}}}}_{{{{{{\rm{P}}}}}}\cap {{{{{\rm{G}}}}}}}\left({s}_{{{{{{\rm{P}}}}}}},{s}_{{{{{{\rm{G}}}}}}}\right)$$

The estimated PEP is then monotonized so that it decreases with *s*_P_ and *s*_G_ increasing.

Given a PEP threshold *t*, a rejection region can be delineated in which all peak groups are reported as positive identifications (rejecting the null hypothesis). The global FDR (q-value) is the average of the local FDR (PEP)^60^:

14 $$q(t)=\frac{{\sum }_{i\in \{i|{{{{{{\rm{PEP}}}}}}}_{i}\le t\}}{{{{{{\rm{PEP}}}}}}}_{i}}{|\{i|{{{{{{\rm{PEP}}}}}}}_{i}\le t\}|}$$

where *i* is a peak group reported as a positive identification.

The strategy described above is applied to individual runs separately at peak group level. It can also be conducted at glycopeptide level in an “experiment-wide” fashion and in a “global” context^40^. In this study, a q-value cutoff of 1% was used at glycopeptide level in the global context. Besides, only peak groups with q-value < 5% were subjected to downstream multi-run alignment.

### Multi-run alignment

TRIC^41^ from msproteomicstools (version 0.11.0) was used to propagate identification and quantification across runs. The tree-based alignment strategy was used. The maximal RT shift was 90. A fixed q-value cutoff of 0.01 was used, which means only glycopeptide precursors with q-value < 1% in at least one run were reported.

### Glycopeptide and protein glycosite quantification

Median normalization was applied to the peak group intensity matrix for each run. The normalized intensities of peak groups were aggregated into glycopeptide-level intensities per sample by summing the 3 most intensive peak groups per glycopeptide. The 3 most intensive glycopeptides per site-specific glycan were summed to calculate the intensities at site-specific glycan level. The intensities at protein glycosite level were calculated by summing up all site-specific glycan intensities per glycosite.

### Glycoform inference

For glycoform inference, all theoretical Y fragment ions (called “identification transitions”) are generated in silico for each target glycopeptide precursor and the corresponding glycoforms with the same peptide sequence but different glycans that fall within the same precursor isolation window (called “background” glycoforms) when building the spectral library. The identification transitions are in silico generated even when the fragment ions are also observed experimentally. For the fission yeast entrapment libraries, human serum libraries and mixed-organism libraries, background glycoforms were selected from the pGlyco 2.0 built-in glycan database^17^, containing 3065 glycan structures consisted of HexNAc, Hex, NeuAc and Fuc. Since the DDA data analysis is performed using pGlyco, all the glycan structures in the original library are covered by the built-in glycan database. Isomeric glycan structures are combined if their theoretical Y fragment ions are exactly the same. The background glycan structures are sorted by Jaccard similarity coefficient, i.e., the ratio of the number of the theoretical Y fragment ions shared with those of the target glycan to the number of their union:

15 $$J(A,B)=\frac{|{{{{{{\rm{Frag}}}}}}}_{A}{\cap }^{}{{{{{{\rm{Frag}}}}}}}_{B}|}{|{{{{{{\rm{Frag}}}}}}}_{A}{\cup }^{}{{{{{{\rm{Frag}}}}}}}_{B}|}$$

where *A* and *B* are the target glycan and a background glycan structure, respectively. To reduce the search space, only the top *n*_bg_ background glycan structures were included in the spectral library. We tested different *n*_bg_ from 20 to 70, and chose 50 for a trade-off between accuracy and size of search space. Additionally, unfragmented precursor in MS2 can be used and added as identification transitions to support precursor detection as IPF does^44^. In this work, MS2 precursor detection was enabled.

Decoy identification transitions are generated for scoring chromatograms of individual identification transitions in subsequent steps. Instead of reversing the peptide sequence, a random sequence of the 20 amino acids with identical length as the target is generated, with all the PTMs kept, referring to the IPF^34^. Decoy identification transitions are generated using the decoy peptide sequence and the target glycan. To ensure that decoy identification transitions are not overlapping with the target identification transitions, the target and decoy peptide sequence must have at least 1 Da mass difference.

Chromatograms of identification transitions and precursors are extracted from DIA data by OpenSWATH and scored by PyProphet. For a given peak group, both target glycopeptides and the corresponding glycoforms are considered for the extraction of identification transitions. The PEP of MS2 peak groups (PEP_MS2_), precursors (PEP_MS1_) and identification transitions (PEP_transition_) are used by a Bayesian hierarchical model (BHM) and integrated to glycoform-level PPs. The priors for each candidate glycoform (hypothesis *A*_*i*_, *i* ≠ 0) or incorrect detection (hypothesis *A*_0_) of the precursor are derived from the PEP_MS2_:

16 $$P({A}_{i})=\left\{\begin{array}{c}\frac{1-{{{{{{\rm{PEP}}}}}}}_{{{{{{\rm{MS2}}}}}}}}{N}\\ {{{{{{\rm{PEP}}}}}}}_{{{{{{\rm{MS2}}}}}}}\end{array}\,\begin{array}{c}{{{{{\rm{if}}}}}}\,i\,\ne\, 0\\ {{{{{\rm{if}}}}}}\,i=0\end{array}\right.$$

with *N* representing the total number of potential glycoforms including target glycopeptides. The conditional probability for MS1 precursor signal is derived from the PEP_MS1_:

17 $$P({B}_{{{{{{\rm{MS1}}}}}}}|{A}_{i})=\left\{\begin{array}{c}1-{{{{{{\rm{PEP}}}}}}}_{{{{{{\rm{MS1}}}}}}}\\ {{{{{{\rm{PEP}}}}}}}_{{{{{{\rm{MS1}}}}}}}\end{array}\,\begin{array}{c}{{{{{\rm{if}}}}}}\,i\in M\\ {{{{{\rm{if}}}}}}\,i\,\notin\, M\end{array}\right.$$

with *M* representing the set of glycoforms whose precursor *m/z* is within the tolerance window (10 ppm in this study) of the target precursor *m/z*. When information of MS2-level unfragmented precursor of each candidate glycoform is available, the conditional probability of glycoform *i* for MS2 precursor signal *k* is

18 $$P({B}_{{{{{{\rm{MS2}}}}}},{{{{{\rm{Prec}}}}}}k}|{A}_{i})=\left\{\begin{array}{c}1-{{{{{{\rm{PEP}}}}}}}_{{{{{{\rm{MS2}}}}}},{{{{{\rm{Prec}}}}}}k}\\ {{{{{{\rm{PEP}}}}}}}_{{{{{{\rm{MS2}}}}}},{{{{{\rm{Prec}}}}}}k}\end{array}\,\begin{array}{c}{{{{{\rm{if}}}}}}\,k=i\\ {{{{{\rm{if}}}}}}\,k\,\ne\, i\end{array}\right.$$

The combined precursor conditional probability is

19 $$P(B|{A}_{i})=P({B}_{{{{{{\rm{MS1}}}}}}}|{A}_{i})\mathop{\prod}\limits_{k}P({B}_{{{{{{\rm{MS2}}}}}},{{{{{\rm{Prec}}}}}}k}|{A}_{i})$$

The PP of hypothesis *A*_*i*_ with precursor information can thus be defined as

20 $$P({G}_{i})=P({A}_{i}|B)=\frac{P(B|{A}_{i})P({A}_{i})}{{\sum }_{i}P(B|{A}_{i})P({A}_{i})}$$

The conditional probability for each transition *j* is derived from the transition-level PEP:

21 $$P({T}_{j}|{G}_{i})=\left\{\begin{array}{c}1-{{{{{{\rm{PEP}}}}}}}_{j}\\ {{{{{{\rm{PEP}}}}}}}_{j}\end{array}\,\begin{array}{c}{{{{{\rm{if}}}}}}\,j\in {{{{{\rm{transition}}}}}}(i)\\ {{{{{\rm{if}}}}}}\,j\,\notin\, {{{{{\rm{transition}}}}}}(i)\end{array}\right.$$

with transition(*i*) representing the set of identification transitions that can originate from the glycoform *i*. The PP of glycoform *i* (or incorrect MS2 peak group detection *i* = 0) is then defined as

22 $$P({G}_{i}|T)=\frac{({\prod }_{j}P({T}_{j}|{G}_{i}))P({G}_{i})}{{\sum }_{i}({\prod }_{j}P({T}_{j}|{G}_{i}))P({G}_{i})}$$

Isomeric glycan structures of the same monosaccharide composition may have different theoretical fragments. For each glycan composition *i*, the prior is equally divided for each isomeric glycan structure *l*, which is considered separately in the BHM. The PP of a glycan composition is the sum of PPs of its isomeric glycan structures:

23 $${{{{{{\rm{PP}}}}}}}_{{{{{{\rm{comp}}}}}}i}=\mathop{\sum} _{l\in i}P({G}_{{{{{{\rm{struct}}}}}}l}|T)$$

The global FDR (q-value) at glycoform level can be derived from PEP (i.e., 1 − PP), as described in the “**Peakgroup scoring and statistical control**” subsection.

The glycoform-level q-value can also be used for downstream multi-run alignment by TRIC. In this study, target peak groups (excluding background glycans) with glycoform-level q-value < 5% were subjected to downstream multi-run alignment, and glycopeptide precursors with glycoform-level q-value <1% in at least one run were reported finally.

### Entrapment strategy

An entrapment strategy was used to benchmark statistical control. Three entrapment libraries were built for the fission yeast: (i) by selecting glycopeptide precursors from the human serum library (both entrapment); (ii) using peptide sequences from the human serum library and glycans from the fission yeast library (peptide entrapment); (iii) using peptide sequences from the fission yeast library and glycans from the human serum library (glycan entrapment). Only the human glycans containing Fuc or NeuAc monosaccharides were used as entrapment, and thus there was no overlap between the yeast and entrapment glycans. The peptide and glycan entrapment libraries were generated using a semi-empirical approach (see below in Semi-empirical spectral library generation). The three entrapment libraries were randomly subsampled that they were of roughly equivalent size to the fission yeast library. The fission yeast library and each entrapment library were merged and used as the target library, respectively. The generation of decoy was on the whole target library including entrapment. Identification results were filtered by 1% q-value at peak group level by a target-decoy approach, and no glycopeptide-level q-value cutoff was applied in the global context. Glycoform-level q-value cutoff was 1%. Multi-run alignment was not performed. As we introduced the entrapment entries in the target database, the entrapment hits in filtered target hits were considered as false positive results, and thus the entrapment percentage (percentage of the number of entrapment hits to the target hits) can be used to evaluate the performance of error rate control. In a similar way, glycan entrapment libraries were built by adding entrapment glycopeptides with peptide sequences from human and glycans from the model higher plant *Arabidopsis thaliana* to the human serum SSL library. Only the plant glycans containing a xylose monosaccharide were used as entrapment, and thus there was no overlap between the human and entrapment glycans.

### Semi-empirical spectral library generation

A computational approach was used to expand the coverage of spectral libraries for glycopeptides by generating semi-empirical spectra combining the peptide and glycan fragment peaks in the experimental spectra of different glycopeptides using a *k*-nearest neighbor (KNN) strategy. To generate a semi-empirical spectrum of a glycopeptide precursor with peptide *P*, glycan *G*, and charge state *n*+, the algorithm looks up the experimental spectral library to find a set of spectra of *n*+ glycopeptide precursors with peptide *P* (*S*_*P*_) or glycan *G* (*S*_*G*_). Peptides are vectorized to amino acid composition (an array of numbers of the 20 amino acids) and glycan are vectorized to monosaccharide composition (an array of numbers of Hex, NexNAc, NeuAc, and Fuc). Experimental spectra in *S*_*P*_ are ranked by Euclidean distances between its glycan and the target glycan *G* to find the *k* nearest neighbors to *G* in *S*_*P*_, denoted as *S*_*Pk*_. Similarly, the *k* nearest neighbors to peptide *P* in *S*_*G*_ are denoted as *S*_*Gk*_. The peptide/glycan fragment peaks in each spectrum in *S*_*Pk*_/*S*_*Gk*_ are extracted and merged into a consensus peptide/glycan spectrum, respectively, using the strategy described in Spectral library building. The ratio (*r*) of the sum intensity of the peptide fragment peaks to that of the glycan fragment peaks is calculated for each spectrum in *S*_*Pk*_ and *S*_*Gk*_. The consensus peptide and glycan spectra are merged by the mean *r*, and the merged spectrum is used as a semi-empirical spectrum of the target glycopeptide precursor (*P*, *G*, *n*+).

Assuming that glycopeptides with the same peptide sequences will have similar RT, the weighted mean RT of the spectra in *S*_*Pk*_ is used as a semi-empirical RT of the target glycopeptide (*P*, *G*). The weight is a Gaussian function of the distance between *G* and the glycan of each experimental spectrum.

24 $${w}_{i}=\frac{1}{\sqrt{2\pi {\sigma }^{2}}}{{\exp }}\left(-\frac{{d}_{i}^{2}}{2{\sigma }^{2}}\right)$$

where *d*_*i*_ is the Euclidean distance between G and the glycan of the experimental spectrum *i* in *S*_*Pk*_, and *σ*2 is the mean of *d*_*i*_^2^ (*i* = 1, …, *k*). The predicted RT value is

25 $${{{{{{\rm{RT}}}}}}}_{\left(P,G\right)}=\frac{{\sum }_{i=1}^{k}{w}_{i}{{{{{{\rm{RT}}}}}}}_{i}}{{\sum }_{i=1}^{k}{w}_{i}}$$

Charge states are ignored when calculating semi-empirical RTs.

For comparison, we also predicted MS/MS and RTs without KNN, in which *S*_*P*_ was used as *S*_*Pk*_, and *S*_*G*_ was used as *S*_*Gk*_.

### Implementation, statistics and visualization

GproDIA was implemented in Python (version 3.5.6, Anaconda distribution version 4.2.0, https://www.anaconda.com/). Post-analysis statistics was conducted using R (version 3.5.1, Microsoft R Open, https://mran.microsoft.com/open). The Python package “matplotlib” (version 3.0.3), as well as the R packages “ggplot2” (version 3.0.0) and “VennDiagram” (version 1.6.20) were used for data visualization.

### Reporting summary

Further information on research design is available in the Nature Research Reporting Summary linked to this article.

## Supplementary information

Supplementary Information

Description of Additional Supplementary Files

Supplementary Data 1

Supplementary Data 2

Supplementary Data 3

Supplementary Data 4

Supplementary Data 5

Supplementary Data 6

Supplementary Data 7

Supplementary Data 8

Supplementary Data 9

Supplementary Data 10

Supplementary Data 11

Supplementary Data 12

Supplementary Data 13

Supplementary Data 14

Supplementary Data 15

Supplementary Data 16

Supplementary Data 17

Supplementary Data 18

Supplementary Data 19

Supplementary Data 20

Reporting Summary

## Data Availability

All raw mass spectrometry data, spectral libraries and search results generated in this study have been deposited in the ProteomeXchange via the iProX^61^ partner repository with the dataset identifiers PXD023980 or IPX0002792000. Swiss-Prot protein databases used in this study are available at UniProt (https://www.uniprot.org), and have also been deposited to the ProteomeXchange/iProX repository. Source data are provided with this paper.

## Source data

Source data file

## Code availability

GproDIA is open source and freely available on GitHub (https://github.com/lmsac/GproDIA) and Zenodo (https://zenodo.org/record/5496762)^62^.
