# Supplementary material for: GproDIA enables data-independent acquisition glycoproteomics with comprehensive statistical control
Source: Nat Commun. 2021 Oct 18;12:6073. doi: 10.1038/s41467-021-26246-3 (PMC8523693; doi:10.1038/s41467-021-26246-3)
Supplement: Supplementary file 1 — Supplementary Information [file 41467_2021_26246_MOESM1_ESM.pdf]

Supplementary Information for

GproDIA enables data-independent acquisition glycoproteomics with comprehensive statistical control

Yi Yang et al.

Supplementary Table 1. Spectral libraries used in this study.

Name	Description
Fission yeast SSL	A sample-specific spectral library of fission yeast generated from DDA data using a 6 h LC gradient with 3 repeat injections. An extra DDA injection with an 1 h LC gradient has been used for RT calibration. Data with the 1 h gradient have also been appended to the calibrated library.<br>Containing 502 precursors of 434 glycopeptides, 412 site-specific glycans, 156 protein glycosites (excluding decoys).
Fission yeast LRL	A lab repository-scale spectral library of fission yeast generated by combining the SSL library and fission yeast data of previous projects in our labs [a]. RTs have been calibrated to the 1 h LC gradient.<br>Containing 1044 precursors of 832 glycopeptides, 725 site-specific glycans, 235 protein glycosites (excluding decoys).
Fission yeast EXL	An extended spectral library of fission yeast generated by combining the SSL library and a semi-empirical library generated from the SSL library.<br>Containing 833 precursors of 733 glycopeptides, 700 site-specific glycans, 156 protein glycosites (excluding decoys).
Budding yeast	A lab repository-scale spectral library of budding yeast generated from budding yeast DDA data. RTs have been calibrated to the 1 h LC gradient using an extra injection of DDA.<br>Containing 850 precursors of 667 glycopeptides, 613 site-specific glycans, 241 protein glycosites (excluding decoys).
Serum SSL	A sample-specific spectral library of human serum generated from DDA data using an 1 h LC gradient with 20 fractions. An extra DDA injection with an 1 h LC gradient without fractionation has been used for RT calibration. Data with the 1 h gradient and without fractionation have also been appended to the calibrated library.<br>Containing 3518 precursors of 2402 glycopeptides, 2082 site-specific glycans, 396 protein glycosites (excluding decoys).
Serum LRL	A lab repository-scale spectral library of human serum generated by combining the SSL library and serum data of previous projects in our labs [a]. RTs have been calibrated to the 1 h LC gradient.<br>Containing 5734 precursors of 4011 glycopeptides, 3519 site-specific glycans, 571 protein glycosites (excluding decoys).
Serum EXL	An extended spectral library of human serum generated by combining the SSL library and a semi-empirical library generated from the SSL library.<br>Containing 5508 precursors of 3433 glycopeptides, 3009 site-specific glycans, 396 protein glycosites (excluding decoys).
Yeast + serum SSL	A spectral library generated by combining the budding yeast library and the serum SSL library.
Yeast + serum LRL	A spectral library generated by combining the budding yeast library and the serum LRL library.

Synthetic	A spectral library of 14 sialylated synthetic glycopeptides generated from DDA data using an 1 h LC gradient with 3 repeat injections. The DDA data and data of fucosylated glycopeptides collected from previous projects of our lab have been used to generate the semi-empirical entrapment glycopeptides, which have been appended to the library. Peptide sequences and glycans are listed in **Supplementary Table 2**.<br>Containing 25 + 41 precursors of 14 + 21 glycopeptides (synthetic + entrapment, excluding decoys).
Both entrapment	A spectral library generated by combining the fission yeast SSL library and 500 precursors randomly sampled from the serum SSL library.
Peptide entrapment	A spectral library generated by combining the fission yeast SSL library and 500 entrapment precursors [b]. The entrapment precursors have been generated semi-empirically using the fission yeast and serum SSL libraries, and then randomly subsampled to 500. The peptide sequences of the entrapment precursors are from human, while the glycans are from yeast.
Glycan entrapment	A spectral library generated by combining the fission yeast SSL library and 500 entrapment precursors [b]. The entrapment precursors have been generated semi-empirically using the fission yeast and serum SSL libraries, and then randomly subsampled to 500. The peptide sequences of the entrapment precursors are from yeast, while the glycans are from human.
Serum + plant glycan entrapment	A spectral library generated by combining the serum SSL library and 3500 entrapment precursors [b]. The entrapment precursors have been generated semi-empirically using the serum SSL libraries and data of *Arabidopsis thaliana* glycopeptides collected from previous projects of our lab, and then randomly subsampled to 3500. The peptide sequences of the entrapment precursors are from human, while the glycans are from *A. thaliana*.
Both entrapment EXL	A spectral library generated by combining the fission yeast EXL library and 850 precursors randomly sampled from the serum SSL library.
Peptide entrapment EXL	A spectral library generated by combining the fission yeast EXL library and 850 entrapment precursors [b]. The entrapment precursors have been generated semi-empirically using the fission yeast and serum SSL libraries, and then randomly subsampled to 850. The peptide sequences of the entrapment precursors are from human, while the glycans are from yeast.
Glycan entrapment EXL	A spectral library generated by combining the fission yeast EXL library and 850 entrapment precursors [b]. The entrapment precursors have been generated semi-empirically using the fission yeast and serum SSL libraries, and then randomly subsampled to 850. The peptide sequences of the entrapment precursors are from yeast, while the glycans are from human.

[a] The combination of libraries is performed using the approach of generating "consensus" spectrum when multiple spectra for a glycopeptide exist in different libraries. During the procedure, some glycopeptides can be eliminated if the consensus spectrum does not meet the criteria described in the **Spectral library building** in the **Methods**.

[b] As a special case, when generating entrapment libraries using the semi-empirical approach, variants with the same glycan monosaccharide compositions but different isomeric glycan structures are regarded as different precursors which can have different glycan fragment peaks. The same criteria are followed when counting entrapment identifications in DIA results.

Supplementary Table 2. Sequences and glycans of the synthetic glycopeptides.

<table>
<tr><th>Sequence</th><th colspan="2">Glycan</th></tr>
<tr><td>DLTHLJR</td><td colspan="2">$N_4H_5A_1$</td></tr>
<tr><td>EEQFJSTFR</td><td colspan="2">$N_4H_5A_2$</td></tr>
<tr><td>EEQYJSTYR</td><td>$N_4H_5F_2$</td><td rowspan="3">Entrapment in spectral library</td></tr>
<tr><td>GHTLTLJFTR</td><td>$N_4H_5A_1F_2$</td></tr>
<tr><td>LLNINPJK</td><td>$N_4H_5F_4$</td></tr>
<tr><td>RFJGSVSFFR</td><td rowspan="2" colspan="2"></td></tr>
<tr><td>VQPFJVTQGK</td></tr>
</table>

"N" replaced by "J" denotes the glycosylation site. A glycan composition is represented in the form of "N-H-A-F", where "H" stands for Hex, "N" stands for HexNAc, "A" stands for NeuAc, and "F" stands for Fuc.

Supplementary Table 3. Nomenclature of monosaccharides used in this study.

Name	Abbr.	Char	Symbol	SNFG
hexose	Hex	H	green circle	white circle
N-acetylhexosamine	HexNAc	N	blue square	white square
N-acetylneuraminic acid	NeuAc	A	purple diamond	purple diamond
fucose	Fuc	F	red triangle	red triangle
xylose	Xyl	X	orange star	orange star

SNFG: Symbol Nomenclature for Glycans

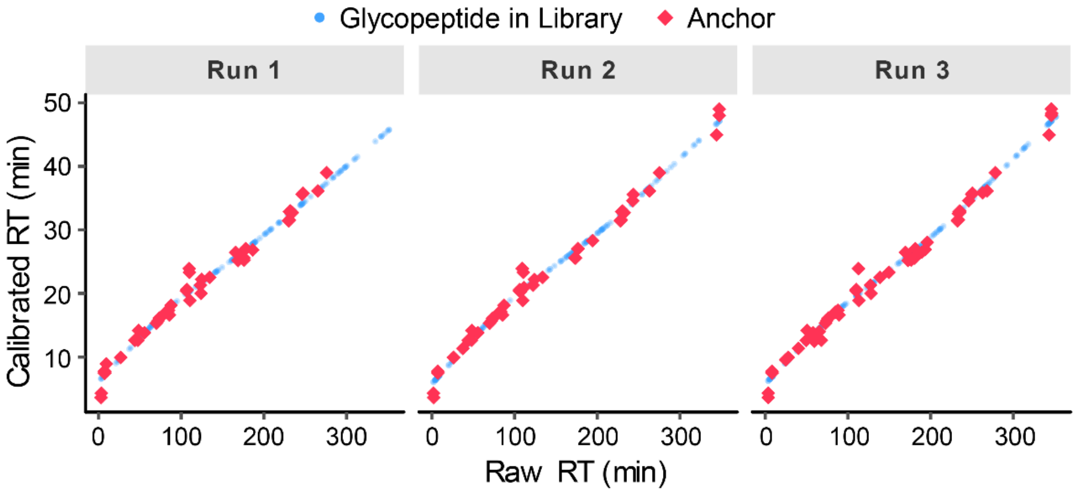

Supplementary Fig. 1. RT calibration for the fission yeast SSL library. RTs of glycopeptides in a 6 h LC gradient were transformed into an 1 h LC gradient. The shared identifications between the 6 h and the 1 h LC gradients were used as anchors. Source data are provided as a Source Data file.

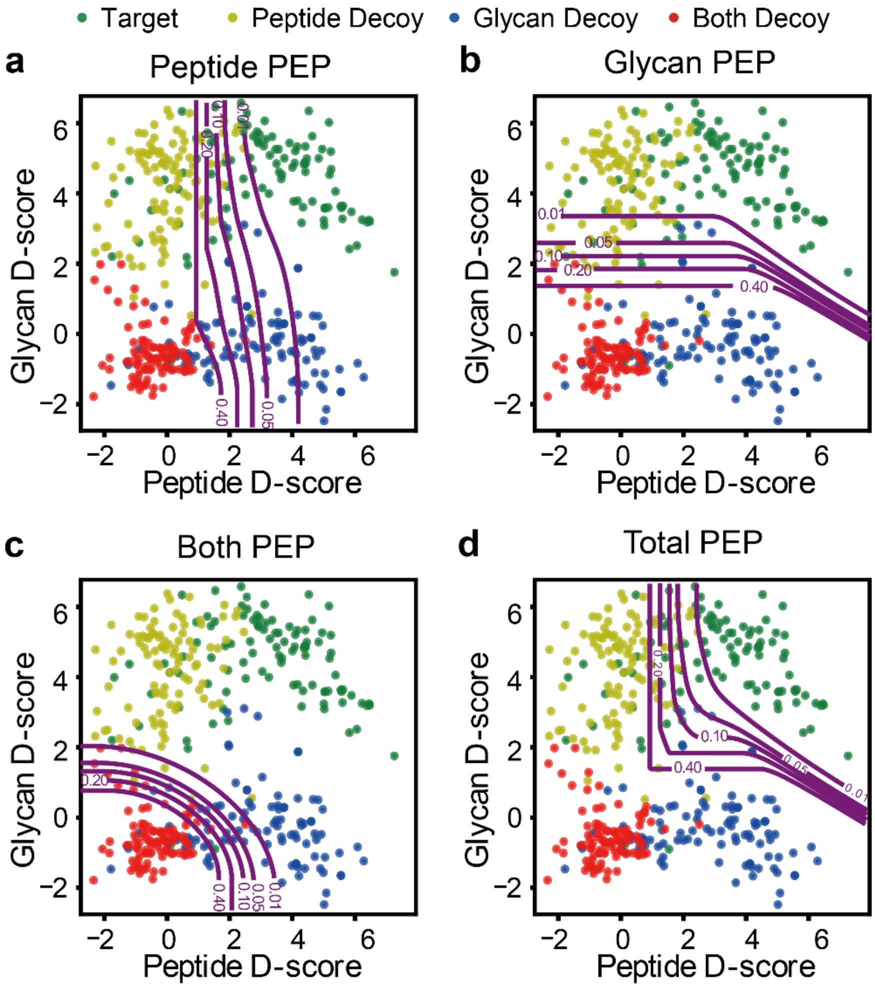

Supplementary Fig. 2. Contour plots of posterior error probability (PEP) of peak groups extracted from the fission yeast DIA data using the SSL library. (**a**) PEP that the peptide part of a peak group is a false identification. (**b**) PEP that the glycan part of a peak group is a false identification. (**c**) PEP that both the peptide and the glycan parts of a peak group are false identifications. (**d**) PEP that both/either the peptide and/or glycan parts of a peak group are false identifications. Green color indicates target peak groups, yellow indicates peptide decoy peak groups, blue indicates glycan decoy peak groups, and red indicates both decoy peak groups. Source data are provided as a Source Data file.

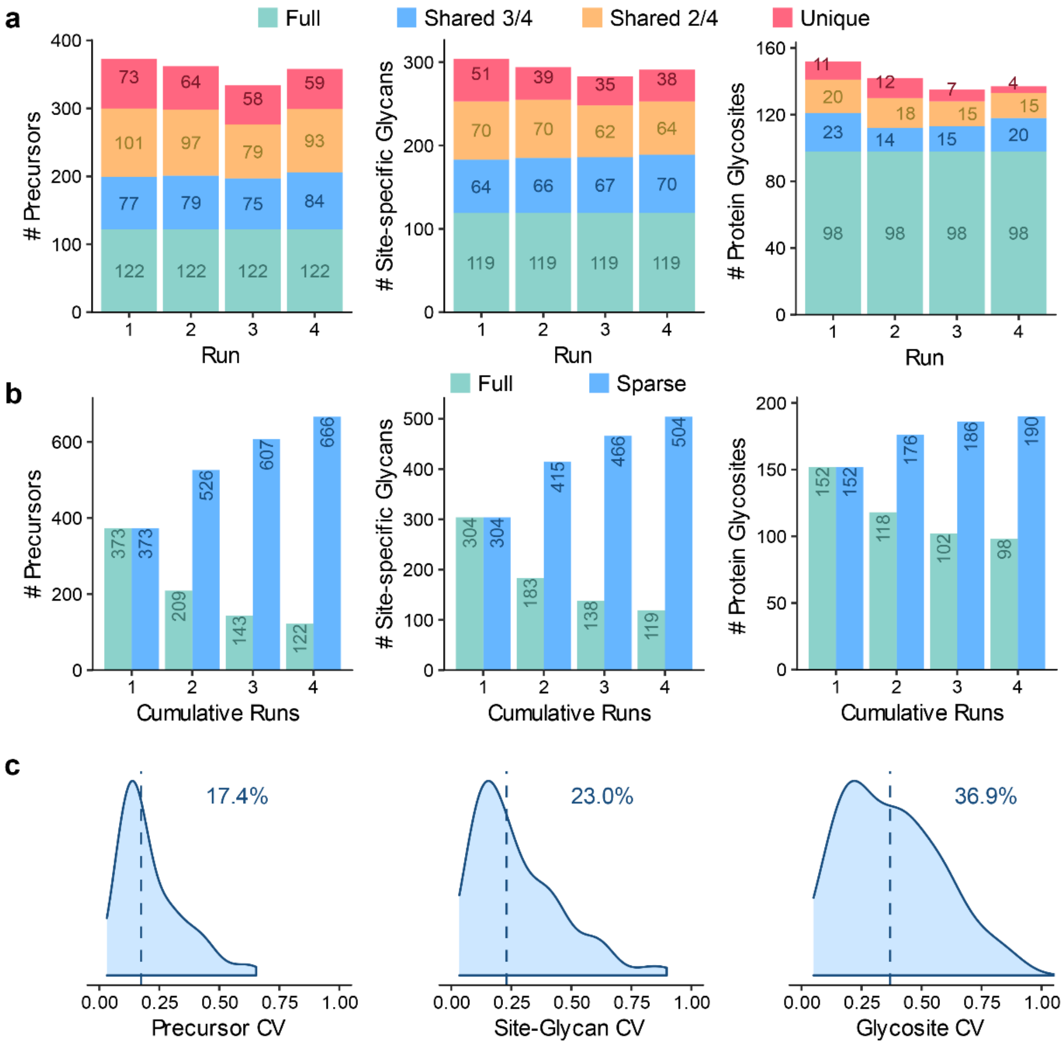

Supplementary Fig. 3. DDA results of the fission yeast sample with an 1 h LC gradient at the level of precursor, site-specific glycan and protein glycosite. (**a**) Numbers of identifications per run. "Full" represents identifications observed in all the runs; "shared 3/4" represents identifications observed in 3 runs; "shared 2/4" represents identifications observed in 2 runs; "unique" represents identifications observed in only 1 run. (**b**) Numbers of cumulative identifications from run 1 to 4. "Full" represents identifications shared in the cumulative runs; "sparse" represents identifications observed in at least one run in the cumulative runs. (**c**) Coefficients of variation (CVs) of quantification results. Medians are indicated. Source data are provided as a Source Data file.

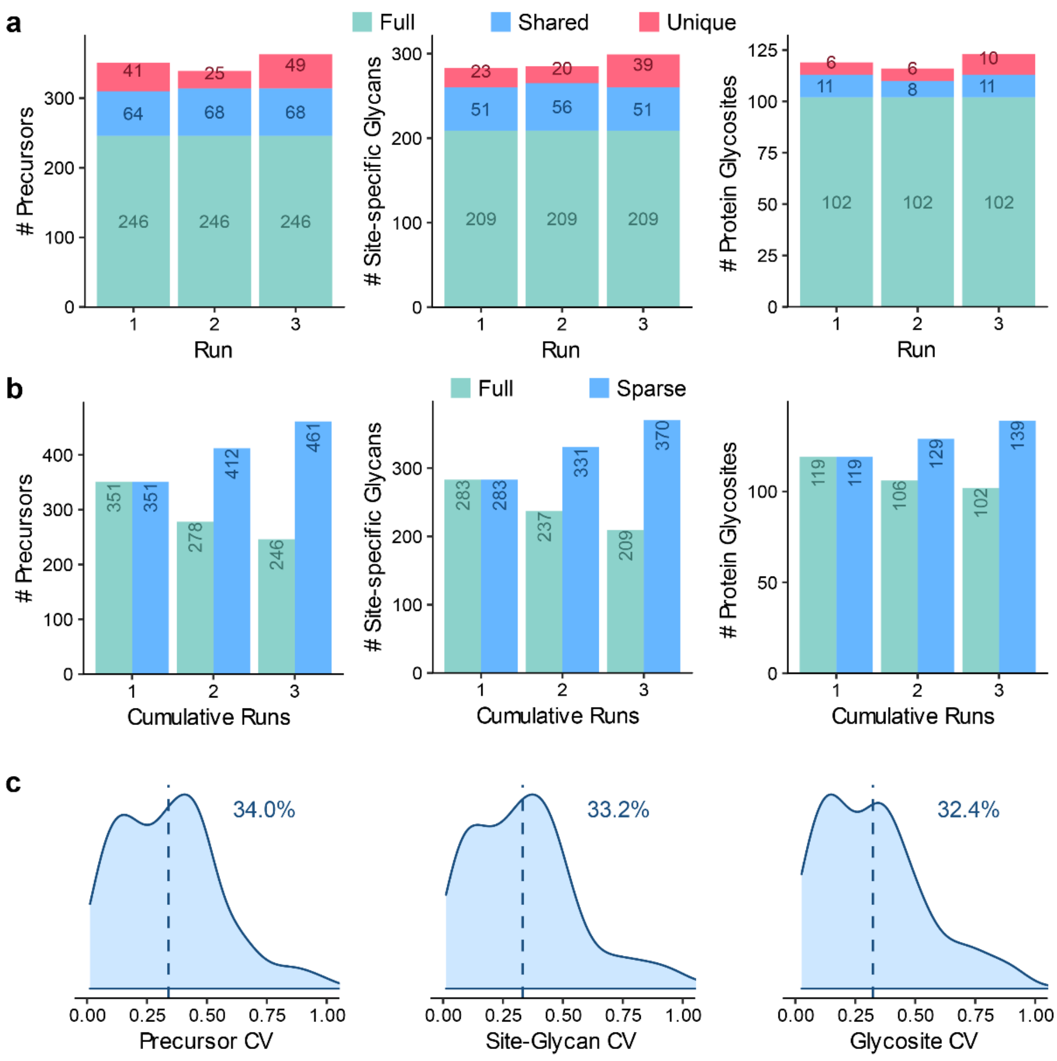

Supplementary Fig. 4. DDA results of the fission yeast sample with a 6 h LC gradient at the level of precursor, site-specific glycan and protein glycosite. (**a**) Numbers of identifications per run. "Full" represents identifications observed in all the runs; "shared" represents identifications observed in 2 runs; "unique" represents identifications observed in only 1 run. (**b**) Numbers of cumulative identifications from run 1 to 3. "Full" represents identifications shared in the cumulative runs; "sparse" represents identifications observed in at least one run in the cumulative runs. (**c**) Coefficients of variation (CVs) of quantification results. Medians are indicated. Source data are provided as a Source Data file.

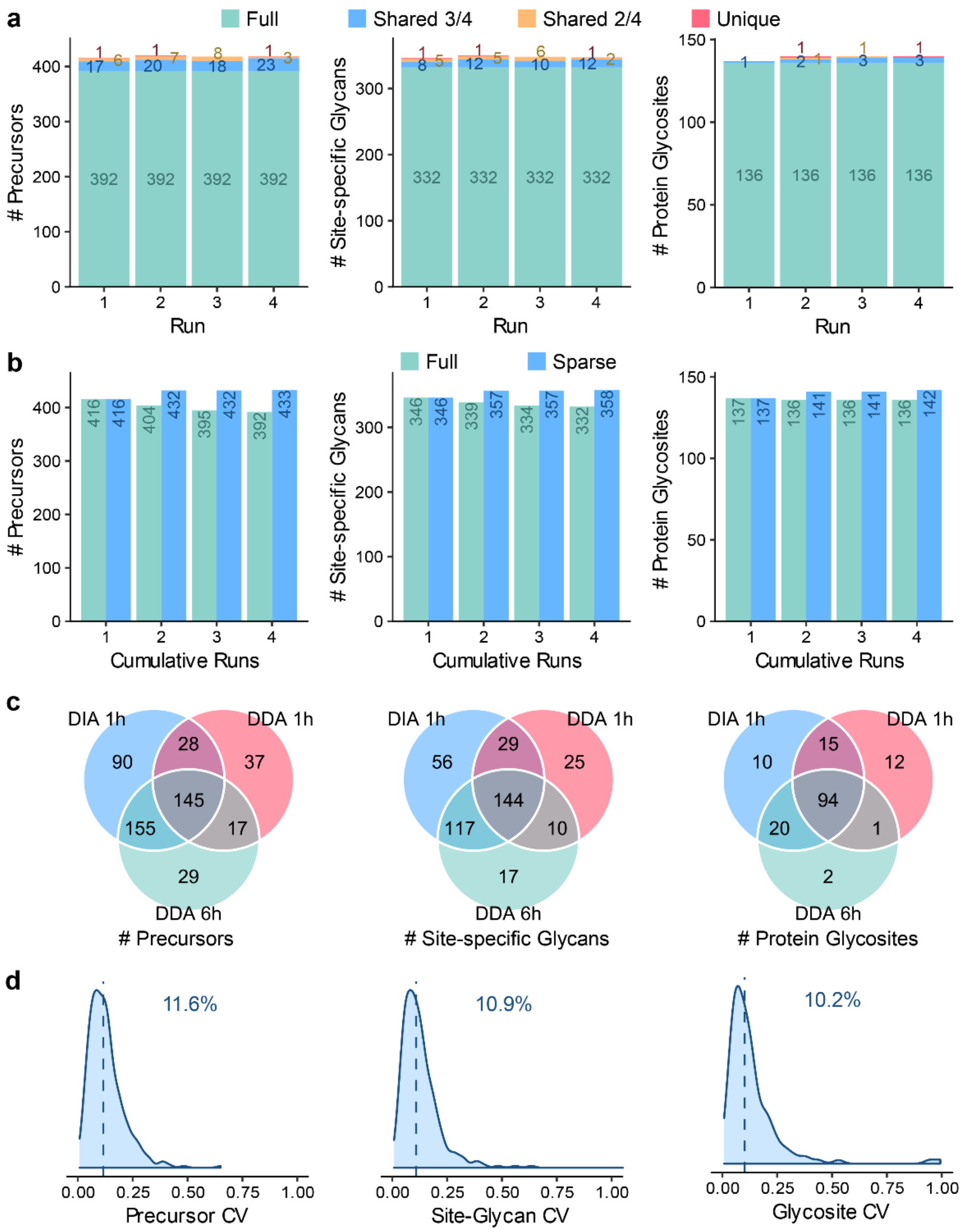

Supplementary Fig. 5. DIA results of the fission yeast sample using the sample-specific library at the level of precursor, site-specific glycan and protein glycosite. (**a**) Numbers of identifications per run. "Full" represents identifications observed in all the runs; "shared 3/4" represents identifications observed in 3 runs; "shared 2/4" represents identifications observed in 2 runs; "unique" represents identifications observed in only 1 run. (**b**) Numbers of cumulative identifications from run 1 to 4. "Full" represents identifications shared in the cumulative runs; "sparse" represents identifications observed in at least one run in the cumulative runs. (**c**) Comparison of numbers of identifications shared in >50% runs using DDA with an 1 h LC gradient, DDA with a 6 h LC gradient, and DIA with an 1 h LC gradient. (**d**) Coefficients of variation (CVs) of quantification results by DIA. Medians are indicated. Source data are provided as a Source Data file.

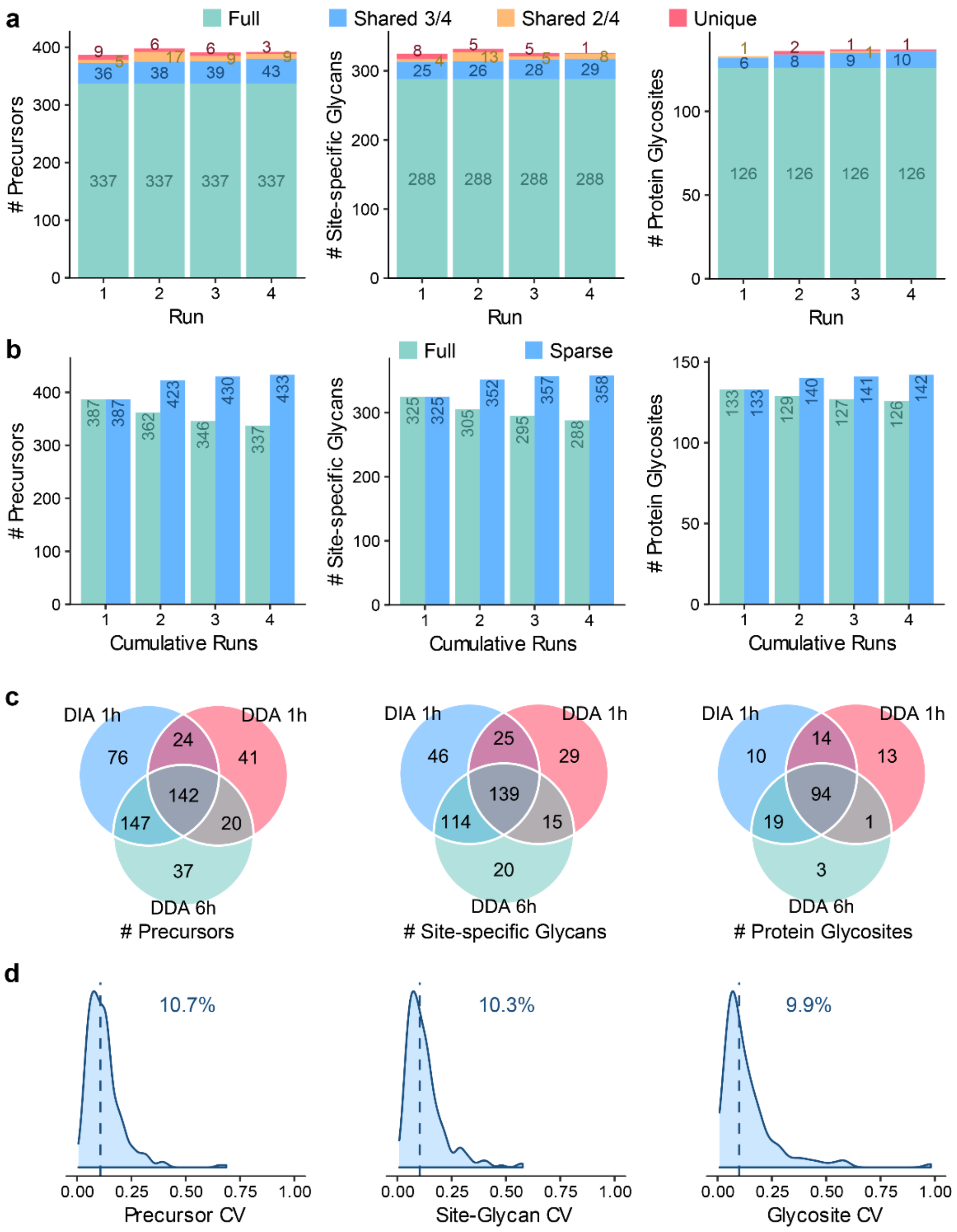

Supplementary Fig. 6. DIA results without multi-run alignment of the fission yeast sample using the sample-specific library at the level of precursor, site-specific glycan and protein glycosite. (**a**) Numbers of identifications per run. "Full" represents identifications observed in all the runs; "shared 3/4" represents identifications observed in 3 runs; "shared 2/4" represents identifications observed in 2 runs; "unique" represents identifications observed in only 1 run. (**b**) Numbers of cumulative identifications from run 1 to 4. "Full" represents identifications shared in the cumulative runs; "sparse" represents identifications observed in at least one run in the cumulative runs. (**c**) Comparison of numbers of identifications shared in >50% runs using DDA with an 1 h LC gradient, DDA with a 6 h LC gradient, and DIA with an 1 h LC gradient. (**d**) Coefficients of variation (CVs) of quantification results by DIA. Medians are indicated. Source data are provided as a Source Data file.

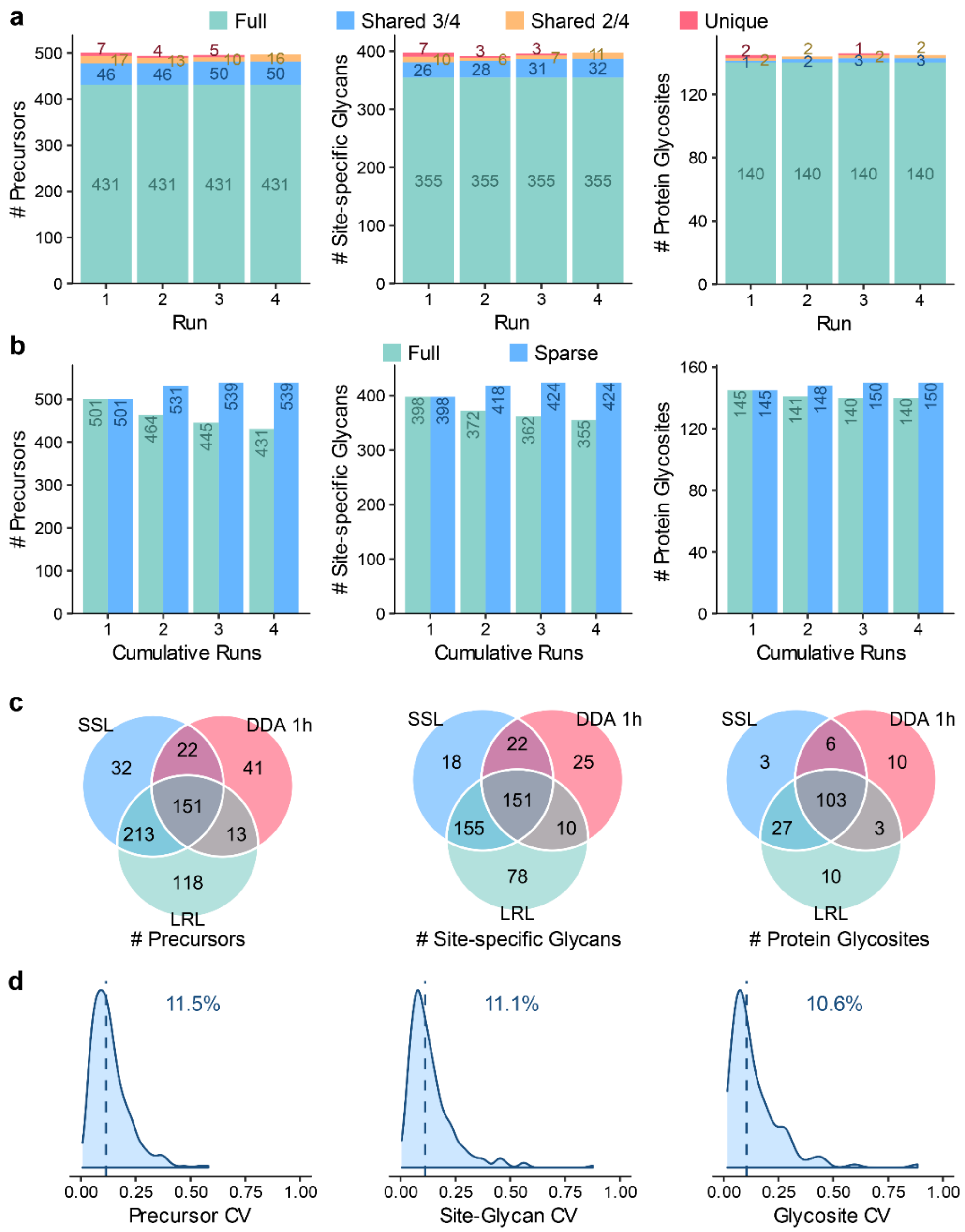

Supplementary Fig. 7. DIA results of the fission yeast sample using the lab repository-scale library at the level of precursor, site-specific glycan and protein glycosite. (**a**) Numbers of identifications per run. "Full" represents identifications observed in all the runs; "shared 3/4" represents identifications observed in 3 runs; "shared 2/4" represents identifications observed in 2 runs; "unique" represents identifications observed in only 1 run. (**b**) Numbers of cumulative identifications from run 1 to 4. "Full" represents identifications shared in the cumulative runs; "sparse" represents identifications observed in at least one run in the cumulative runs. (**c**) Comparison of numbers of identifications shared in 3/4 runs using DDA with an 1 h LC gradient, DIA with the sample-specific library (SSL), and DIA with the lab repository-scale library (LRL). (**d**) Coefficients of variation (CVs) of quantification results by DIA with LRL. Medians are indicated. Source data are provided as a Source Data file.

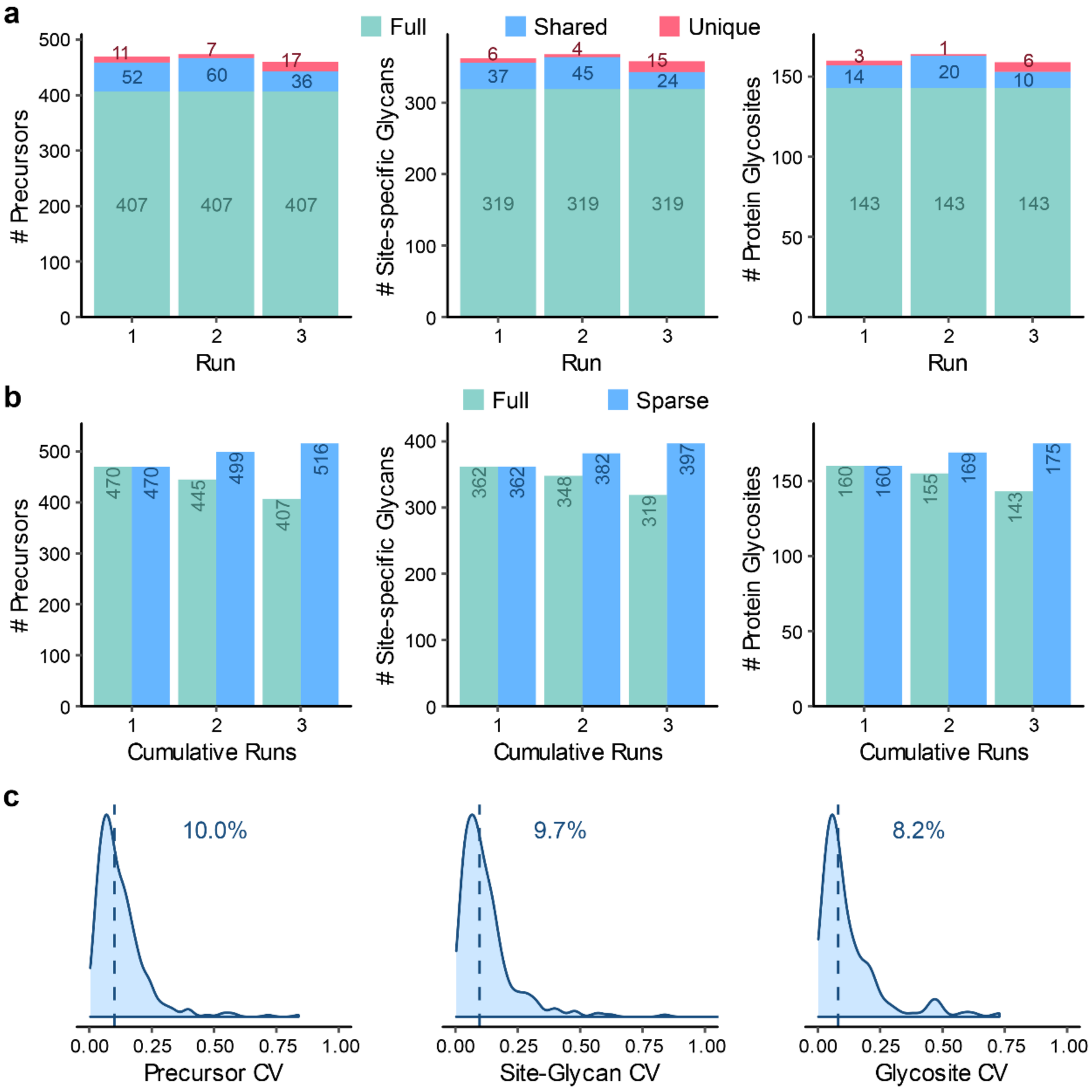

Supplementary Fig. 8. DIA results of the budding yeast sample at the level of precursor, site-specific glycan and protein glycosite. (**a**) Numbers of identifications per run. "Full" represents identifications observed in all the runs; "shared" represents identifications observed in 2 runs; "unique" represents identifications observed in only 1 run. (**b**) Numbers of cumulative identifications from run 1 to 3. "Full" represents identifications shared in the cumulative runs; "sparse" represents identifications observed in at least one run in the cumulative runs. (**c**) Coefficients of variation (CVs) of quantification results. Medians are indicated. Source data are provided as a Source Data file.

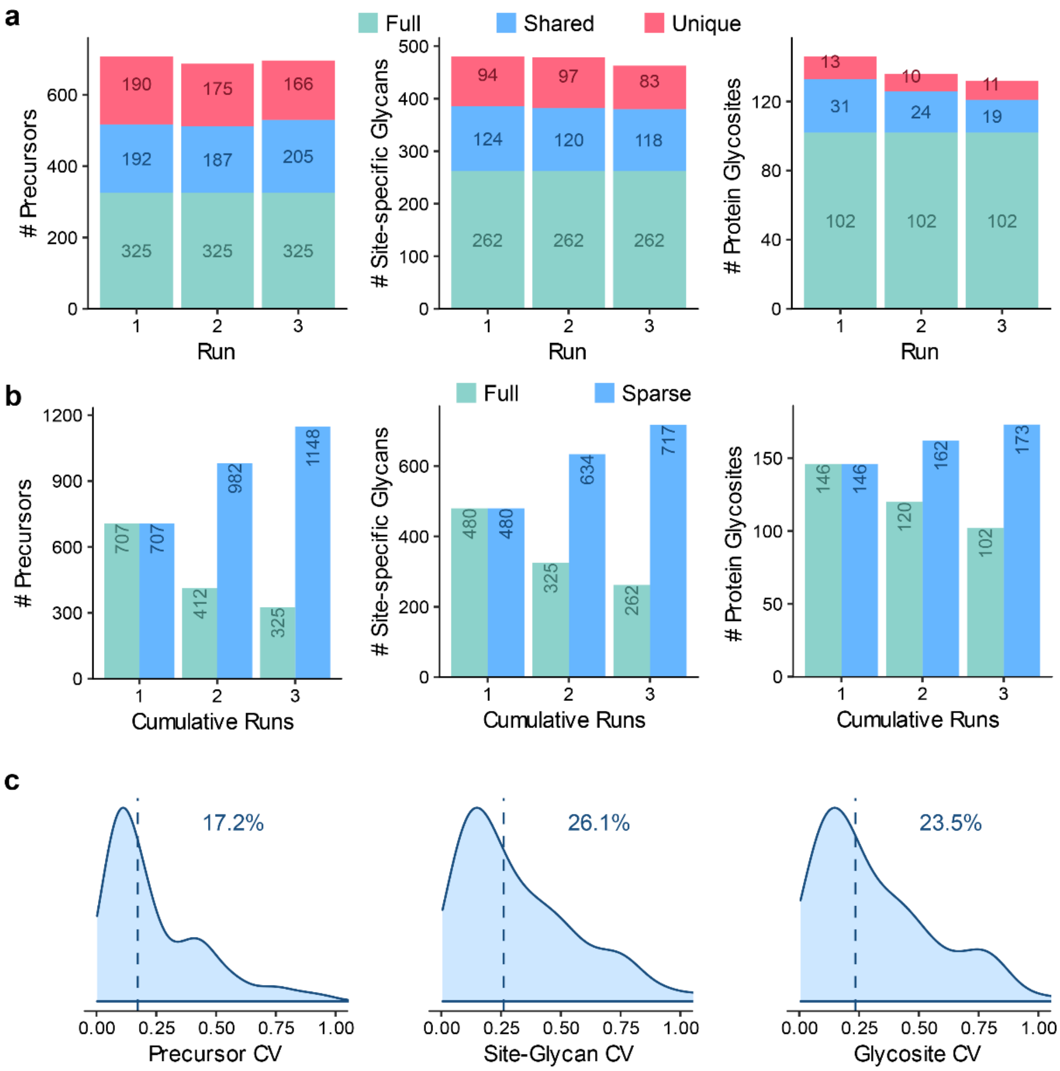

Supplementary Fig. 9. DDA results of the human serum sample at the level of precursor, site-specific glycan and protein glycosite. (**a**) Numbers of identifications per run. "Full" represents identifications observed in all the runs; "shared" represents identifications observed in 2 runs; "unique" represents identifications observed in only 1 run. (**b**) Numbers of cumulative identifications from run 1 to 3. "Full" represents identifications shared in the cumulative runs; "sparse" represents identifications observed in at least one run in the cumulative runs. (**c**) Coefficients of variation (CVs) of quantification results. Medians are indicated. Source data are provided as a Source Data file.

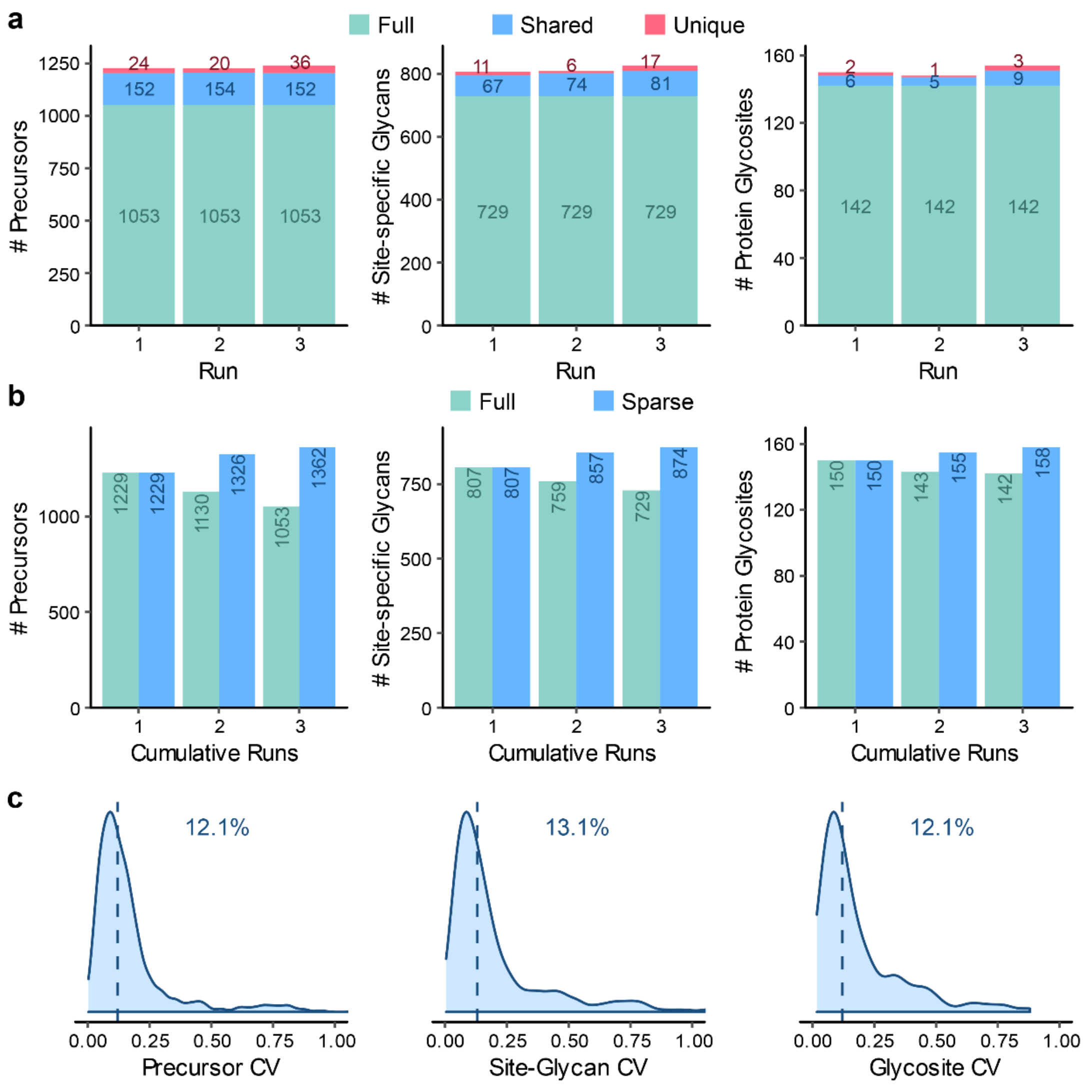

Supplementary Fig. 10. DIA results without glycoform inference of the human serum sample using the sample-specific library at the level of precursor, site-specific glycan and protein glycosite. (**a**) Numbers of identifications per run. “Full” represents identifications observed in all the runs; “shared” represents identifications observed in 2 runs; “unique” represents identifications observed in only 1 run. (**b**) Numbers of cumulative identifications from run 1 to 3. “Full” represents identifications shared in the cumulative runs; “sparse” represents identifications observed in at least one run in the cumulative runs. (**c**) Coefficients of variation (CVs) of quantification results. Medians are indicated. Source data are provided as a Source Data file.

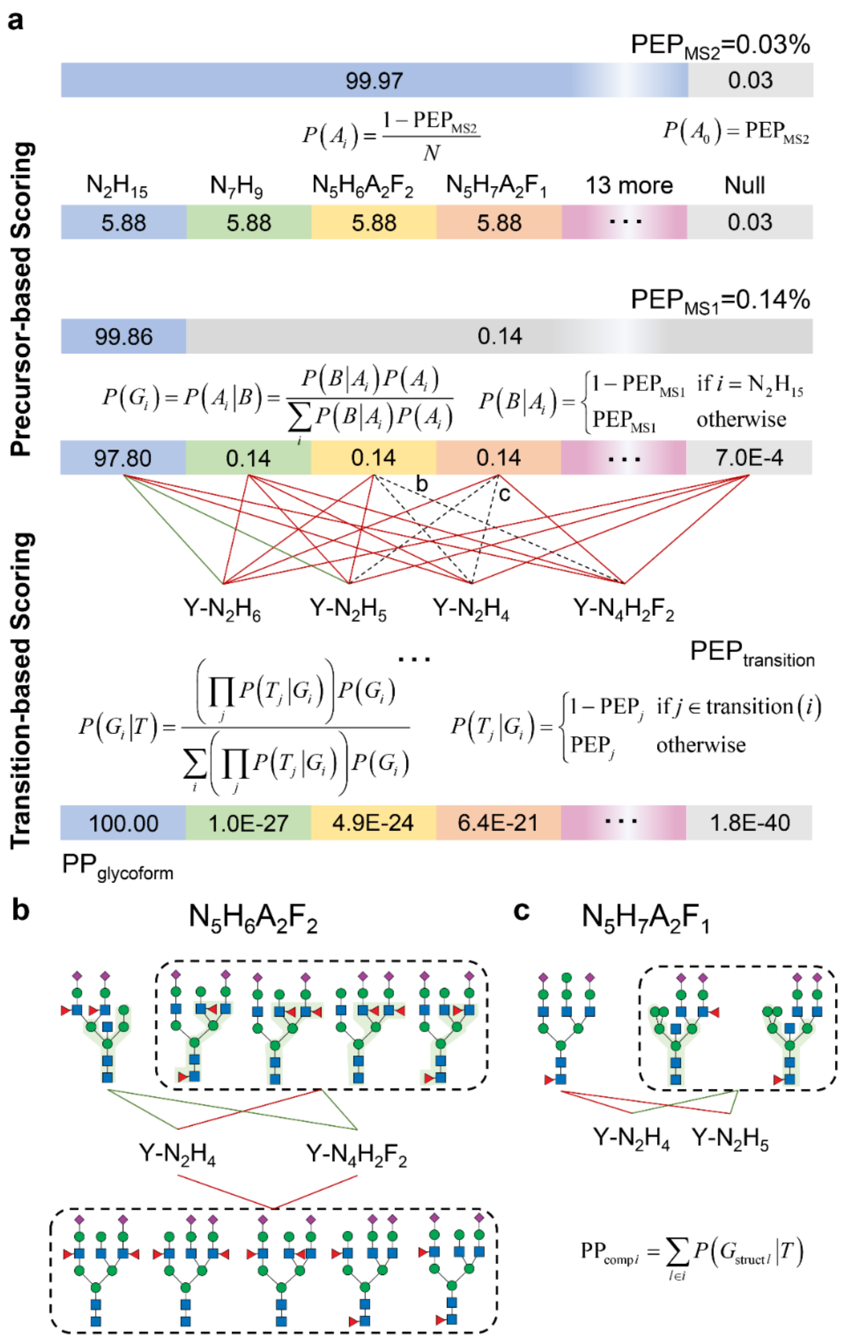

Supplementary Fig. 11. The Bayesian hierarchical model for glycoform inference. (**a**) Integrating the precursor and transition posterior probabilities (PPs) to calculate glycoform PP. (**b**) Isomeric glycan structures of $HexNAc_5Hex_6NeuAc_2Fuc_2$. (**c**) Isomeric glycan structures of $HexNAc_5Hex_7NeuAc_2Fuc_1$. The glycan symbols are as follows: a green circle or "H" represents Hex; a blue square or "N" represents HexNAc; a red triangle or "F" represents Fuc; a purple diamond or "A" represents NeuAc. A green line indicates a Y ion can be originated from a glycoform (glycan composition or structure); otherwise, they are connected with a red line. The information for the dashed lines is expanded in (**b**) and (**c**) because different isomeric glycan structures of the corresponding glycoforms can generate different fragment ions. PEP: posterior error probabilities.

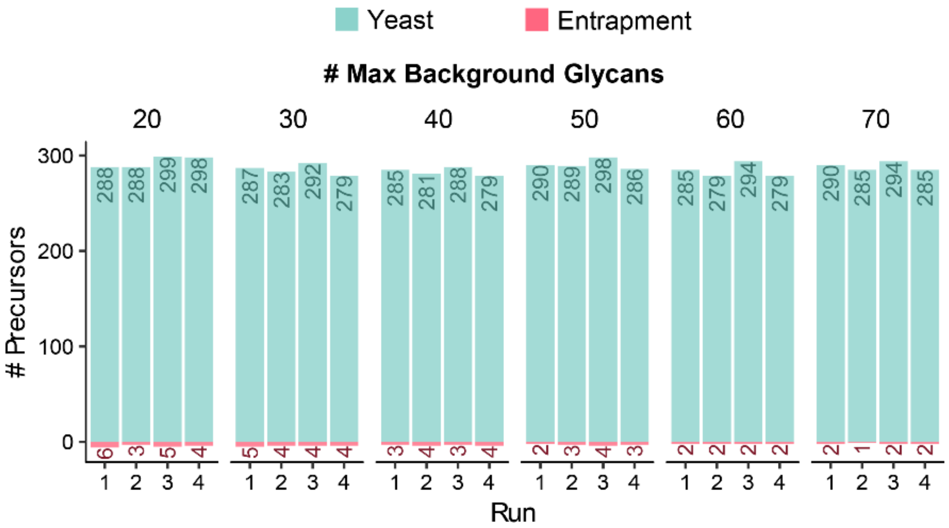

Supplementary Fig. 12. Numbers of identifications from the fission yeast sample using the glycan entrapment libraries with different search spaces of glycoform inference.

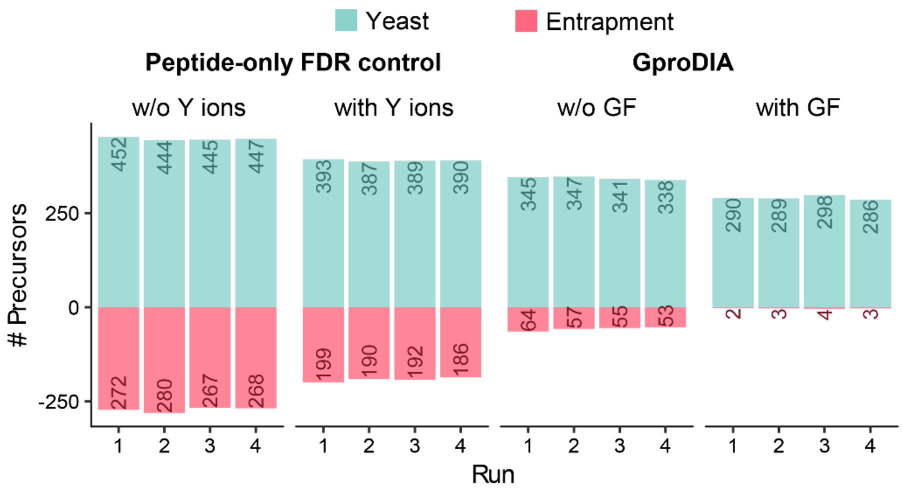

Supplementary Fig. 13. Numbers of identifications from the fission yeast sample using the glycan entrapment libraries (containing glycopeptides with peptide sequences from yeast and glycans from human) by the peptide-only FDR approach and the GproDIA considering 2D FDR control without (w/o GF) or with (with GF) glycoform inference.

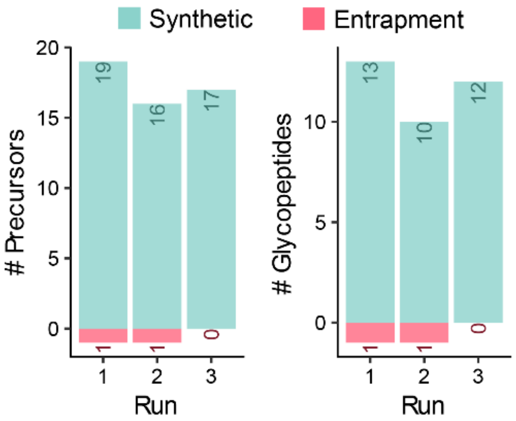

Supplementary Fig. 14. Numbers of identifications from the synthetic glycopeptide sample.

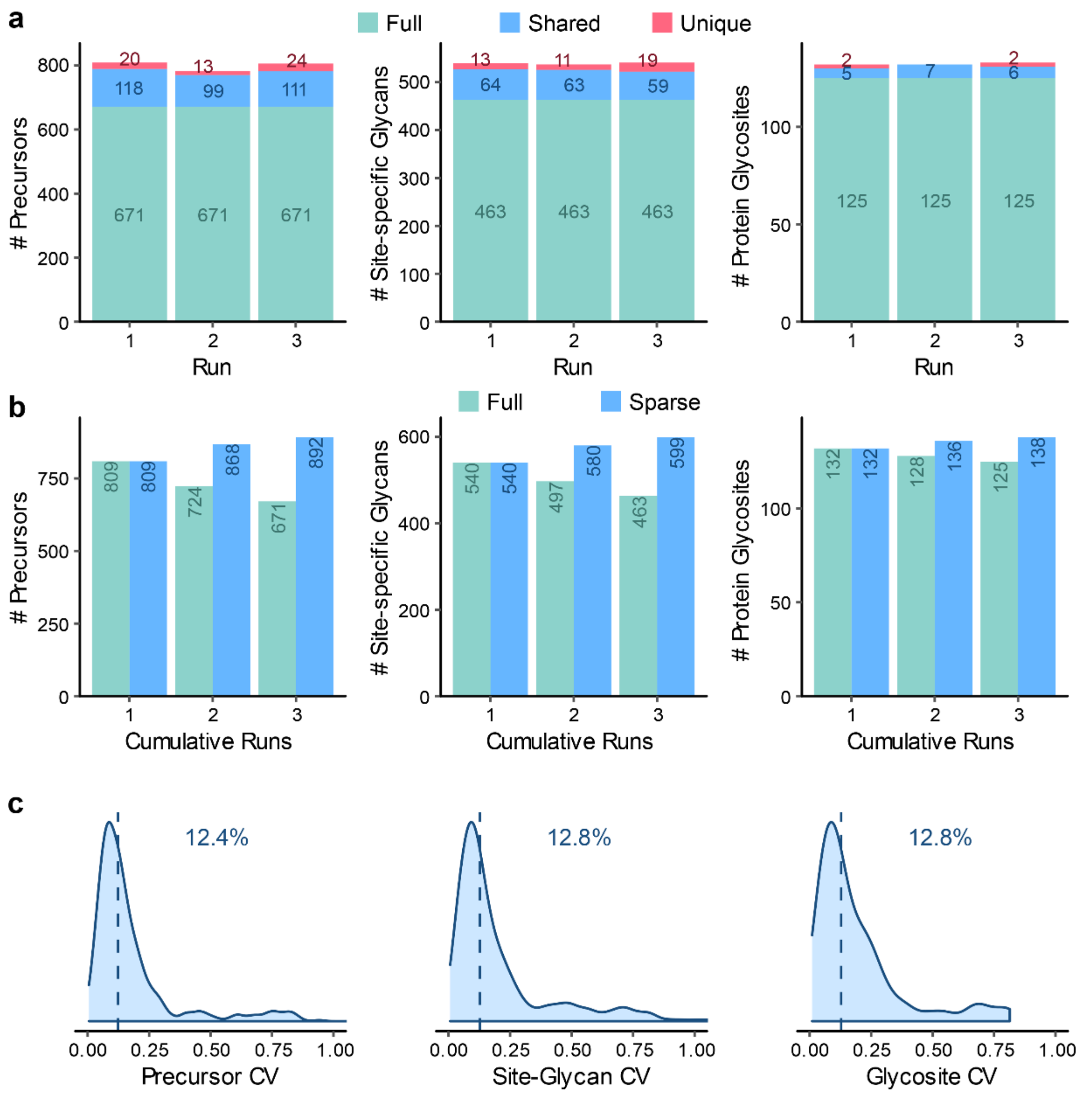

Supplementary Fig. 15. DIA results with glycoform inference of the human serum sample using the sample-specific library at the level of precursor, site-specific glycan and protein glycosite. (**a**) Numbers of identifications per run. "Full" represents identifications observed in all the runs; "shared" represents identifications observed in 2 runs; "unique" represents identifications observed in only 1 run. (**b**) Numbers of cumulative identifications from run 1 to 3. "Full" represents identifications shared in the cumulative runs; "sparse" represents identifications observed in at least one run in the cumulative runs. (**c**) Coefficients of variation (CVs) of quantification results. Medians are indicated. Source data are provided as a Source Data file.

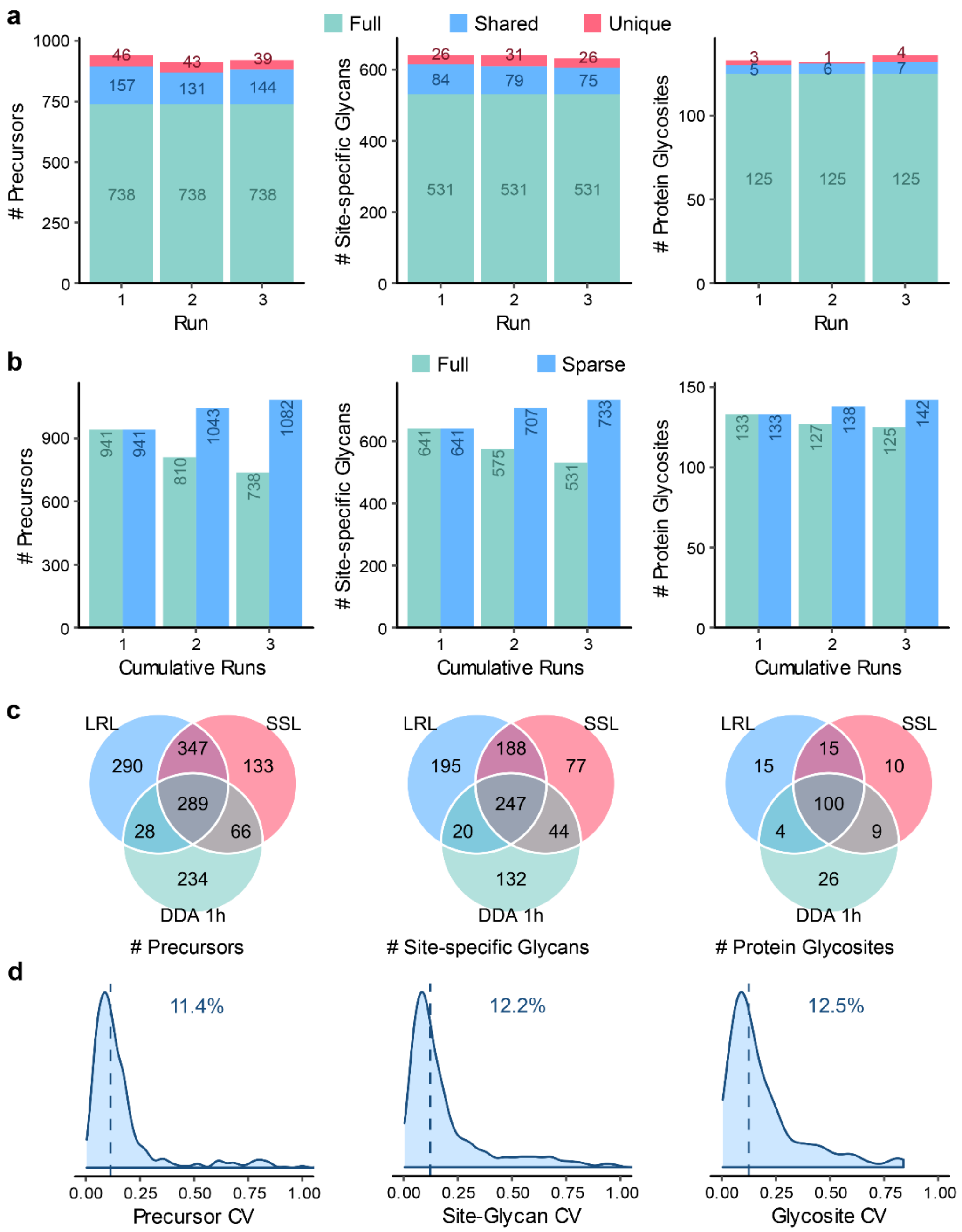

Supplementary Fig. 16. DIA results with glycoform inference of the human serum sample using the lab repository-scale library at the level of precursor, site-specific glycan and protein glycosite. (**a**) Numbers of identifications per run. "Full" represents identifications observed in all the runs; "shared" represents identifications observed in 2 runs; "unique" represents identifications observed in only 1 run. (**b**) Numbers of cumulative identifications from run 1 to 3. "Full" represents identifications shared in the cumulative runs; "sparse" represents identifications observed in at least one run in the cumulative runs. (**c**) Comparison of numbers of identifications shared in 2/3 runs using DDA, DIA with the sample-specific library (SSL), and DIA with the lab repository-scale library (LRL). (**d**) Coefficients of variation (CVs) of quantification results by DIA with LRL. Medians are indicated. Source data are provided as a Source Data file.

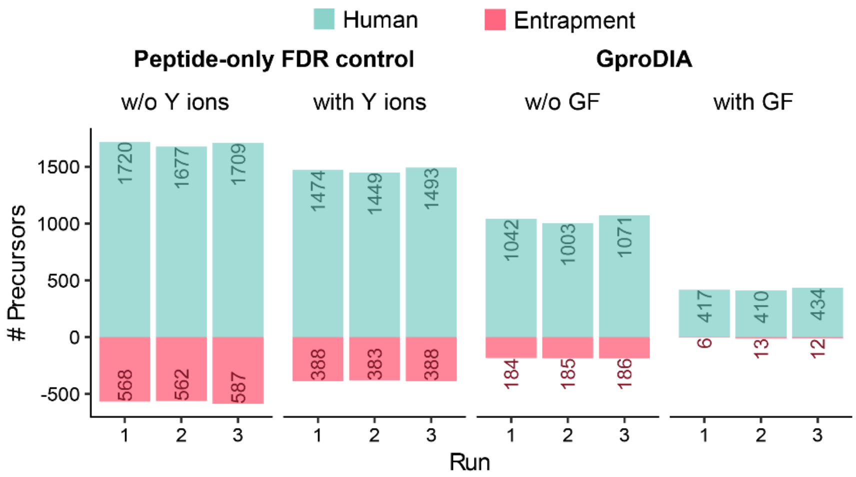

Supplementary Fig. 17. Numbers of identifications from the human serum sample using the serum + plant glycan entrapment library by the peptide-only FDR approach and GproDIA without (w/o GF) or with (with GF) glycoform inference.

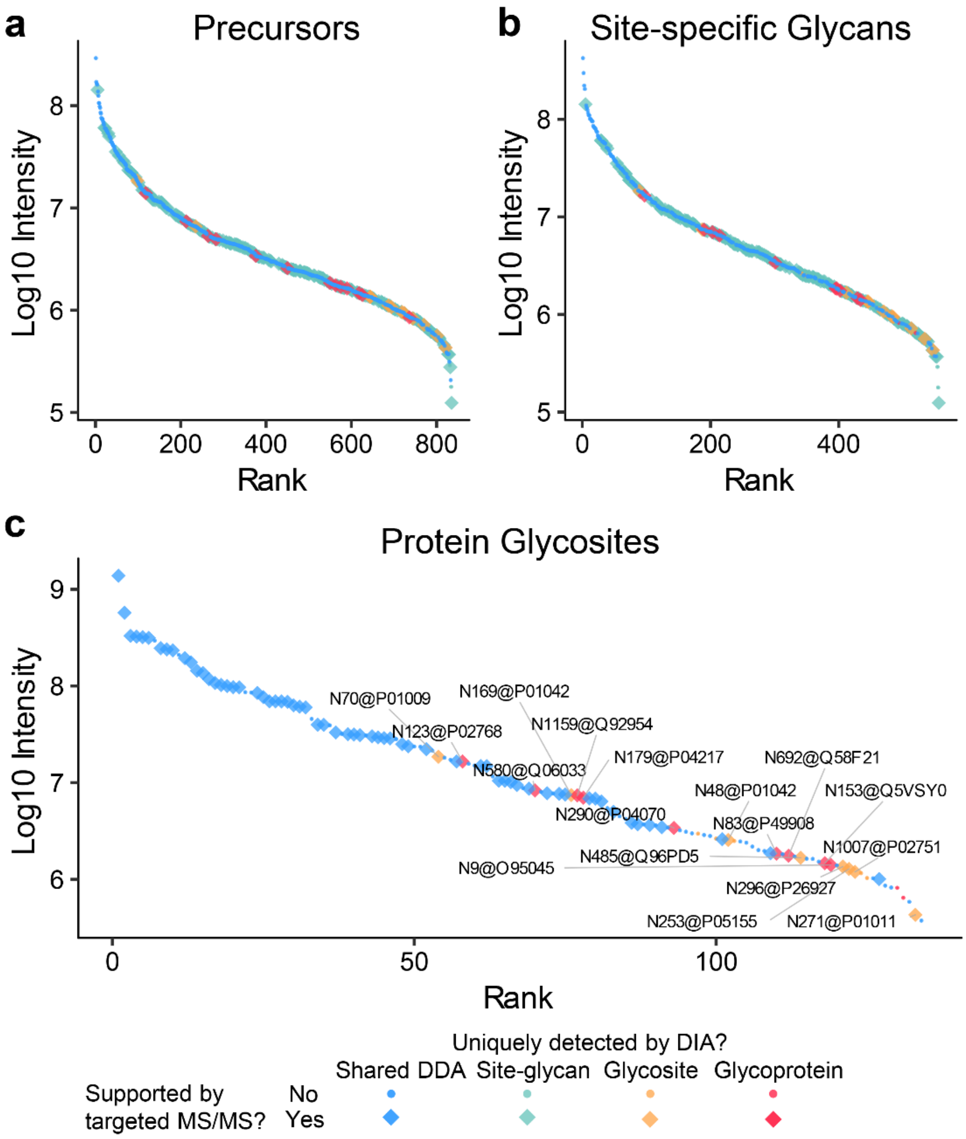

Supplementary Fig. 18. Plots of (**a**) precursor, (**b**) site-specific glycan, and (**c**) protein glycosite quantity ($\log_{10}$) against rank by DIA quantification results of the human serum sample using the SSL library. Only the records shared in at least 2 of the 3 runs are considered, and the mean intensity is used as quantity for each analyte. A small cycle represents a record in DIA result not supported by target MS/MS, and a large diamond represents a record supported by target MS/MS. Records shared with DDA result are in blue; records corresponding to new site-specific glycans uniquely detected by DIA and missed by DDA are in green; records corresponding to new glycosites (except those on new glycoproteins) are in orange; records corresponding to new glycoproteins are in red. Source data are provided as a Source Data file.

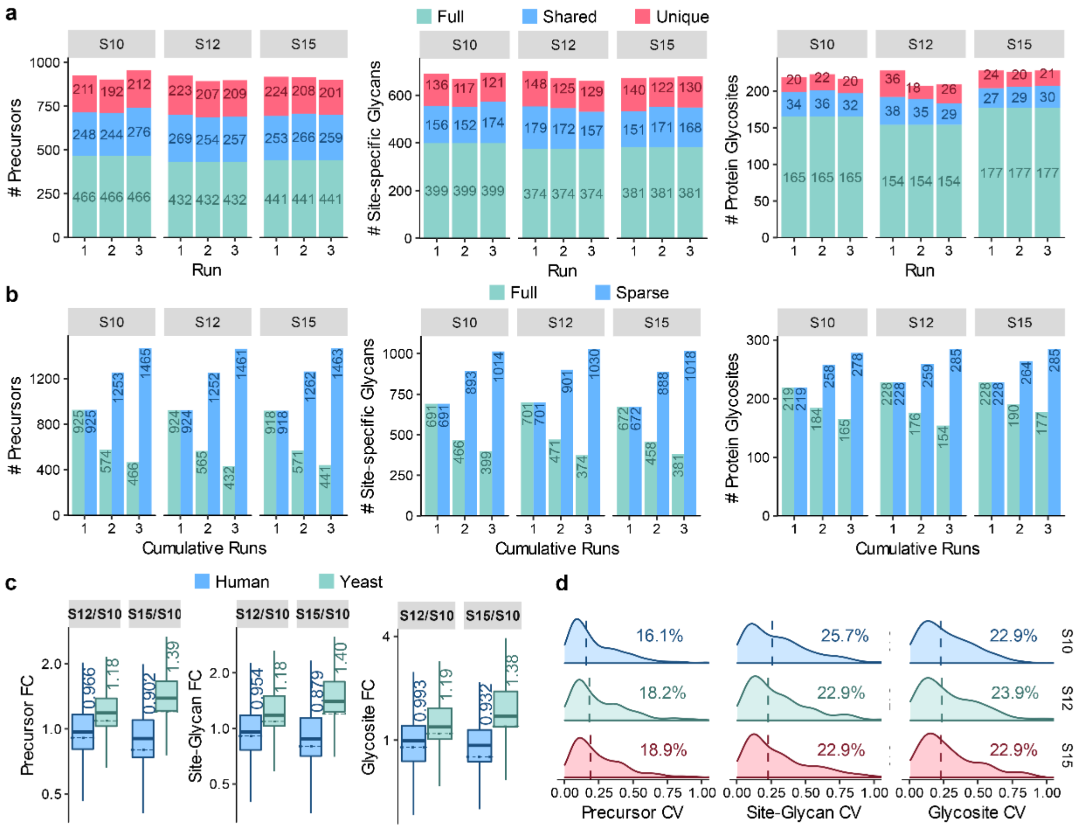

Supplementary Fig. 19. DDA results of the mixed-organism samples at the level of precursor, site-specific glycan and protein glycosite. (**a**) Numbers of identifications per run of each sample. “Full” represents identifications observed in all the runs; “shared” represents identifications observed in 2 runs; “unique” represents identifications observed in only 1 run. (**b**) Numbers of cumulative identifications from run 1 to 3 of each sample. “Full” represents identifications shared in the cumulative runs; “sparse” represents identifications observed in at least one run in the cumulative runs. (**c**) Box plot visualization of fold change of the quantification results of the mixed-organism samples. Percent changes were calculated based on the mean quantities in three replicates of each sample. The medians are indicated. The boxes indicate the interquartile ranges (IQR), and whiskers indicate 1.5 × IQR values; no outliers are shown. The dashed lines indicate theoretical fold changes of the organisms (1:0.9:0.8 (S10:S12:S15) for human and 1:1.1:1.2 (S10:S12:S15) for yeast). (**d**) Coefficients of variation (CVs) of quantification results of each sample. Medians are indicated. Source data are provided as a Source Data file.

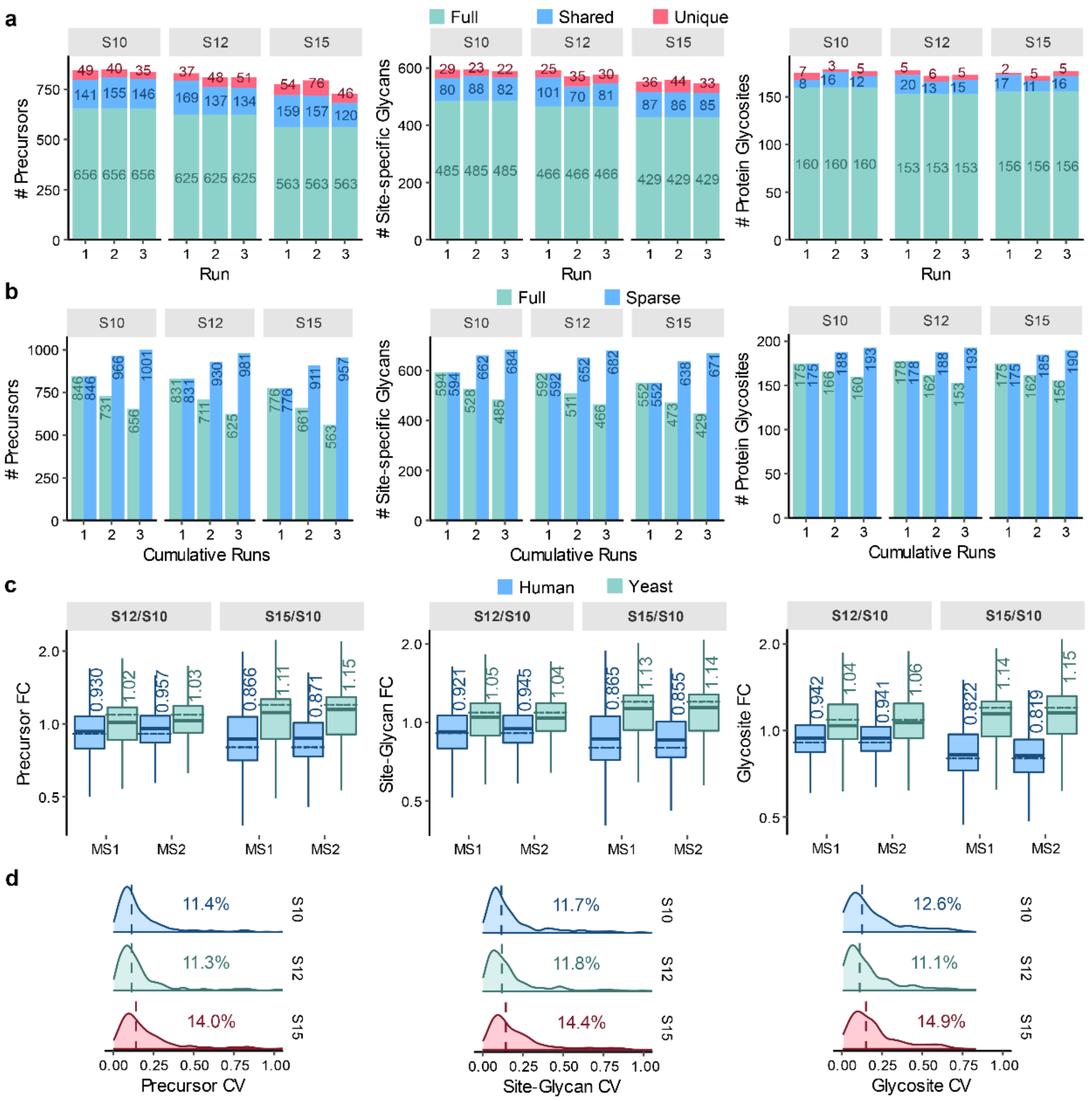

Supplementary Fig. 20. DIA results of the mixed-organism samples using a combined library of the budding yeast library and the serum sample-specific library (SSL) at the level of precursor, site-specific glycan and protein glycosite. (**a**) Numbers of identifications per run of each sample. "Full" represents identifications observed in all the runs; "shared" represents identifications observed in 2 runs; "unique" represents identifications observed in only 1 run. (**b**) Numbers of cumulative identifications from run 1 to 3 of each sample. "Full" represents identifications shared in the cumulative runs; "sparse" represents identifications observed in at least one run in the cumulative runs. (**c**) Box plot visualization of fold change of the MS1 and MS2-level quantification results of the mixed-organism samples. Percent changes were calculated based on the mean quantities in three replicates of each sample. The medians are indicated. The boxes indicate the interquartile ranges (IQR), and whiskers indicate 1.5 × IQR values; no outliers are shown. The dashed lines indicate theoretical fold changes of the organisms (1:0.9:0.8 (S10:S12:S15) for human and 1:1.1:1.2 (S10:S12:S15) for yeast). (**d**) Coefficients of variation (CVs) of quantification results of each sample. Medians are indicated. Source data are provided as a Source Data file.

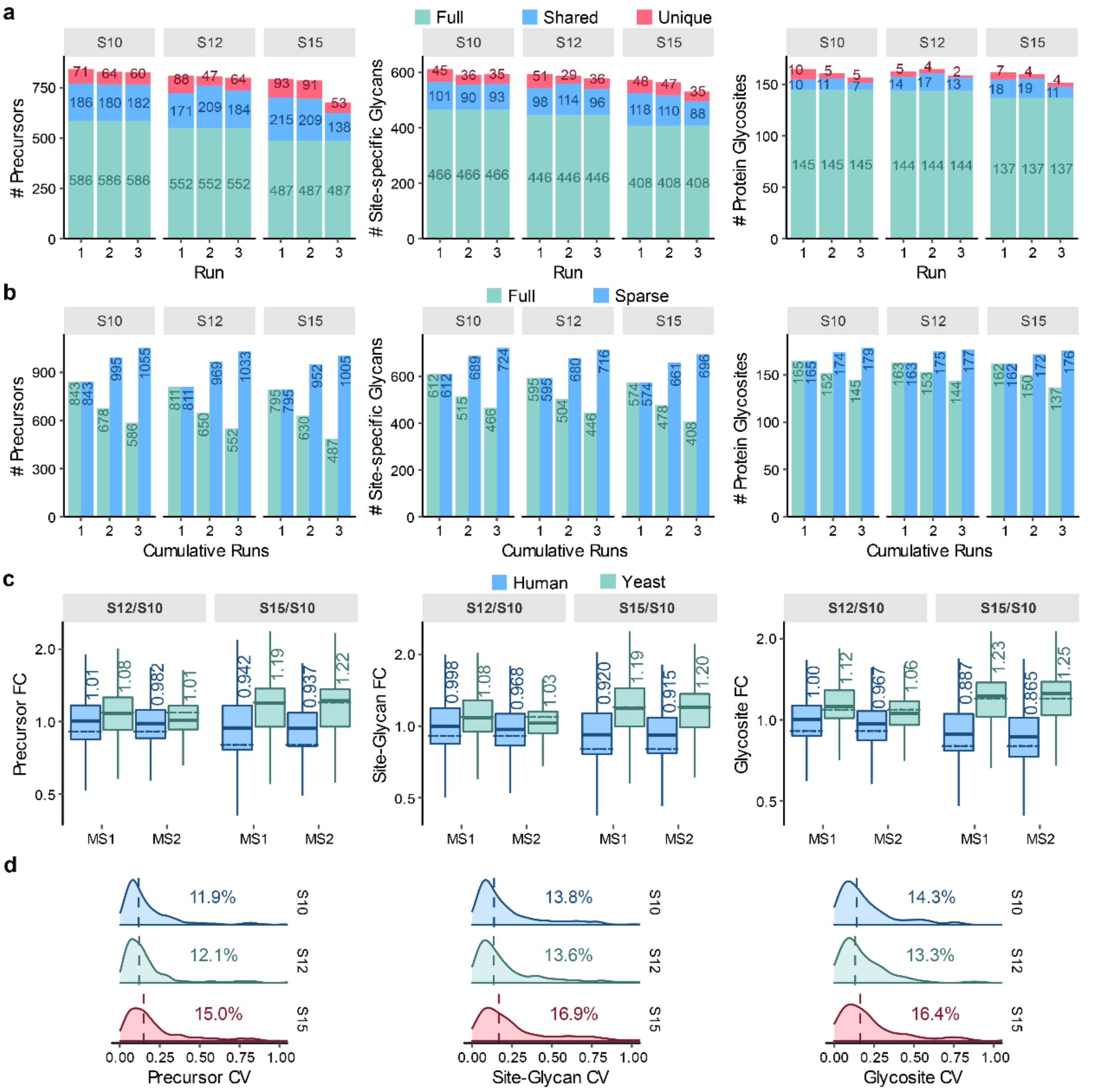

Supplementary Fig. 21. DIA results of the mixed-organism samples using a combined library of the budding yeast library and the serum lab repository-scale library (LRL) at the level of precursor, site-specific glycan and protein glycosite. (**a**) Numbers of identifications per run of each sample. "Full" represents identifications observed in all the runs; "shared" represents identifications observed in 2 runs; "unique" represents identifications observed in only 1 run. (**b**) Numbers of cumulative identifications from run 1 to 3 of each sample. "Full" represents identifications shared in the cumulative runs; "sparse" represents identifications observed in at least one run in the cumulative runs. (**c**) Box plot visualization of fold change of the MS1 and MS2-level quantification results of the mixed-organism samples. Percent changes were calculated based on the mean quantities in three replicates of each sample. The medians are indicated. The boxes indicate the interquartile ranges (IQR), and whiskers indicate 1.5 × IQR values; no outliers are shown. The dashed lines indicate theoretical fold changes of the organisms (1:0.9:0.8 (S10:S12:S15) for human and 1:1.1:1.2 (S10:S12:S15) for yeast). (**d**) Coefficients of variation (CVs) of quantification results of each sample. Medians are indicated. Source data are provided as a Source Data file.

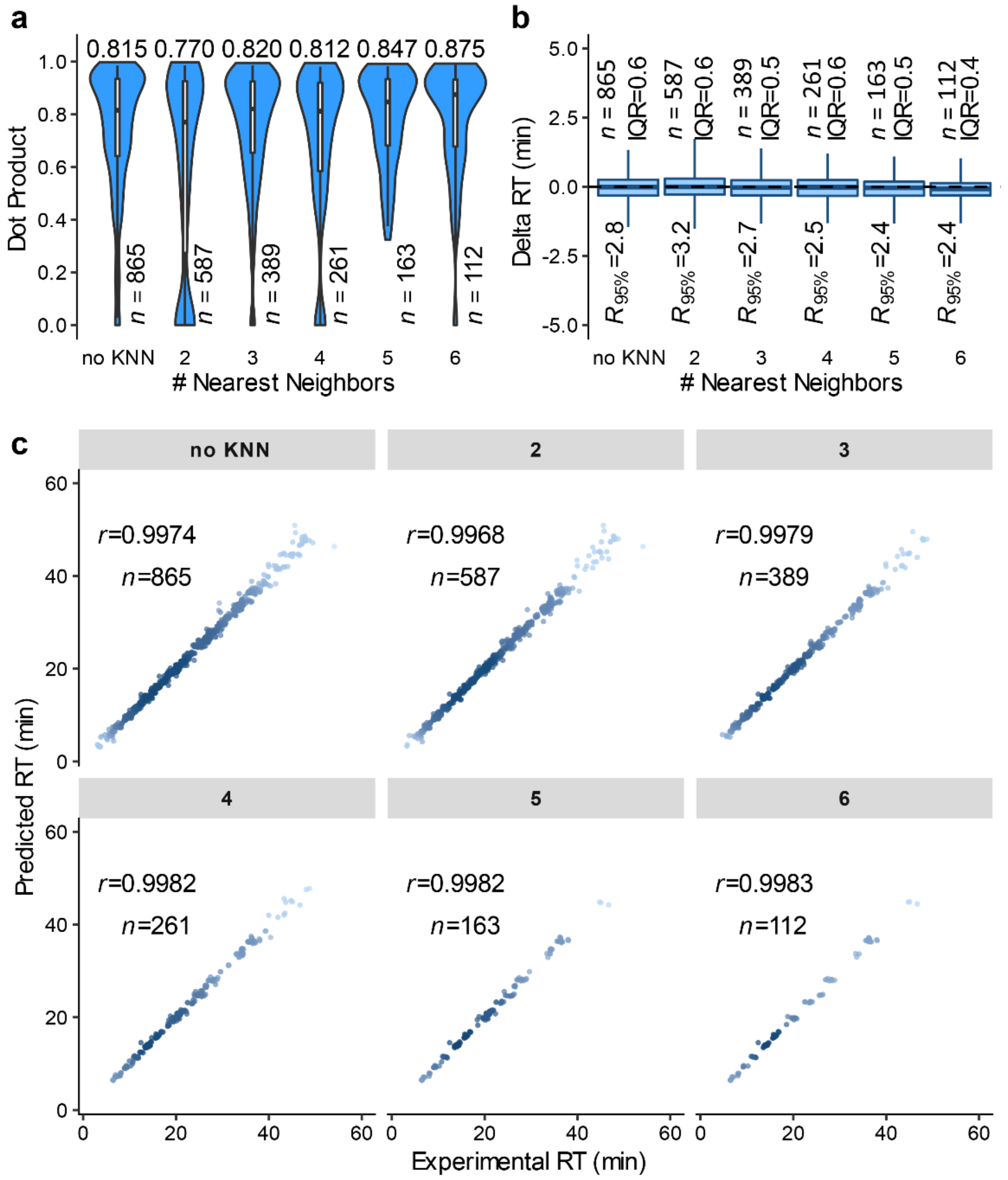

Supplementary Fig. 22. Cross validations of semi-empirical spectral library generation using the fission yeast lab repository-scale library (LRL), tested with different numbers of nearest neighbors *k* or without KNN. (**a**) The distributions of dot products computed between predicted and experimental MS/MS peak intensities. (**b**) The differences between predicted and experimental retention times (RTs). In **a** and **b**, the middle lines of the boxplots indicate the median, the lower/upper hinges correspond to the first/third quartiles, the lower/upper whiskers extend from the hinges to the 2.5%/97.5% percentiles, while data beyond the end of the whiskers are not shown. (**c**) Pearson correlation coefficients (*r*) between predicted and experimental RTs. "*n*" indicates the number of generated MS2 spectra or RT values by the prediction method. Source data are provided as a Source Data file.

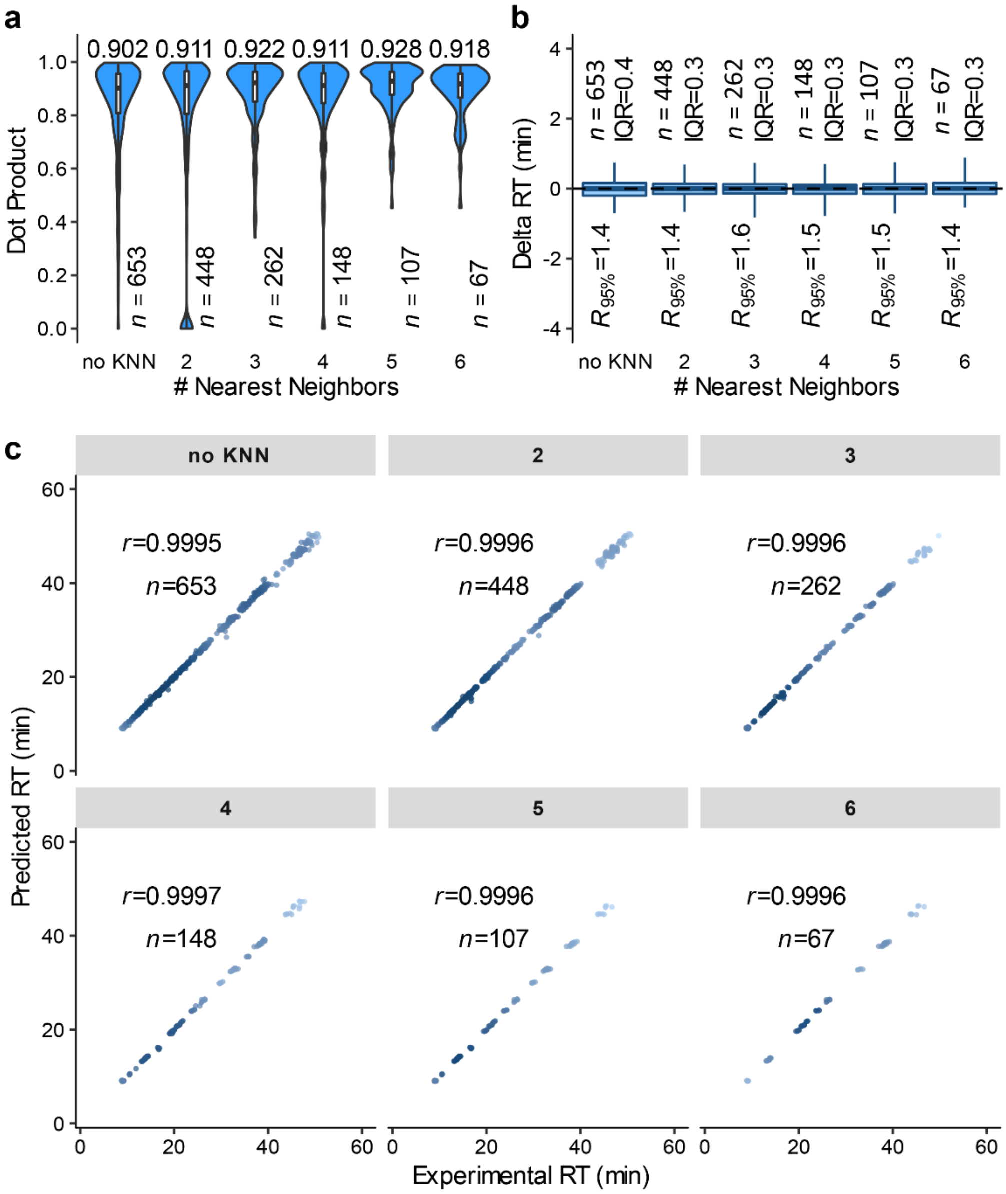

Supplementary Fig. 23. Cross validations of semi-empirical spectral library generation using the budding yeast library, tested with different numbers of nearest neighbors *k* or without KNN. (**a**) The distributions of dot products computed between predicted and experimental MS/MS peak intensities. (**b**) The differences between predicted and experimental retention times (RTs). In **a** and **b**, the middle lines of the boxplots indicate the median, the lower/upper hinges correspond to the first/third quartiles, the lower/upper whiskers extend from the hinges to the 2.5%/97.5% percentiles, while data beyond the end of the whiskers are not shown. (**c**) Pearson correlation coefficients (*r*) between predicted and experimental RTs. "*n*" indicates the number of generated MS2 spectra or RT values by the prediction method. Source data are provided as a Source Data file.

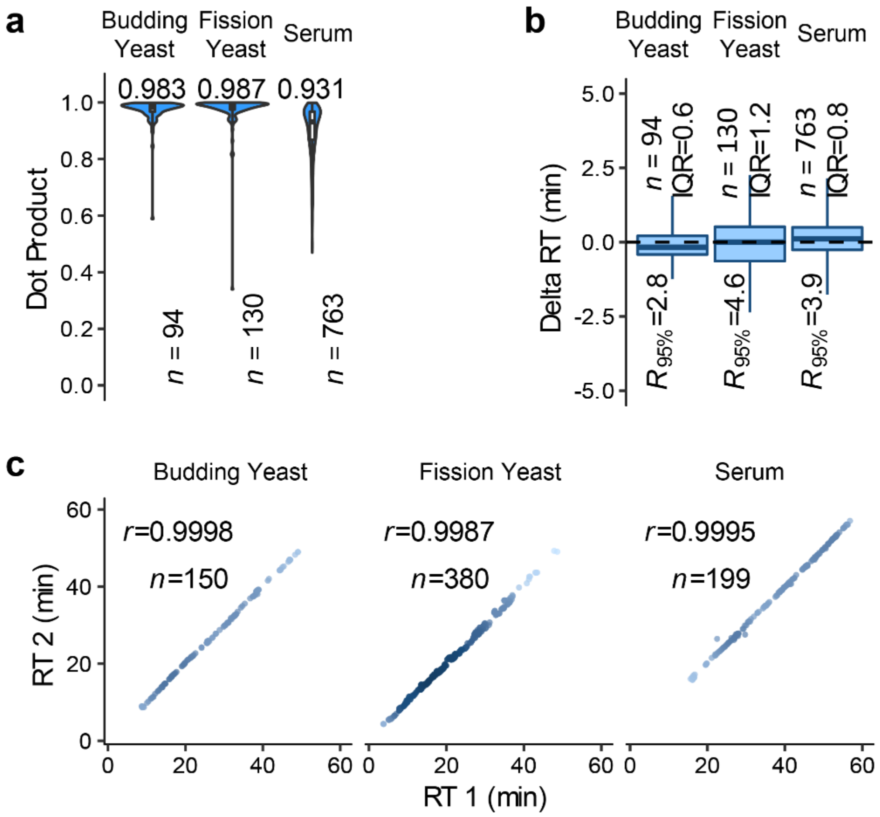

Supplementary Fig. 24. Statistics of replicate experimental spectra in the budding yeast, fission yeast, and serum library. Replicates within each run were combined into consensus spectra, and statistics were computed between the consensus spectra pairwise across runs. For each glycopeptide precursor, the minimum of the paired DPs and the maximum (absolute value) of the paired retention time (RT) differences were kept. (**a**) The distributions of dot products computed between MS/MS peak intensities of consensus replicate spectra. (**b**) The differences between retention times (RTs) of consensus replicate spectra. In **a** and **b**, the middle lines of the boxplots indicate the median, the lower/upper hinges correspond to the first/third quartiles, the lower/upper whiskers extend from the hinges to the 2.5%/97.5% percentiles, while data beyond the end of the whiskers are not shown. (**c**) Pearson correlation coefficients (*r*) between RTs of consensus replicate spectra in two randomly chosen runs. “*n*” indicates the number of paired experimental MS2 spectra or RT values. Source data are provided as a Source Data file.

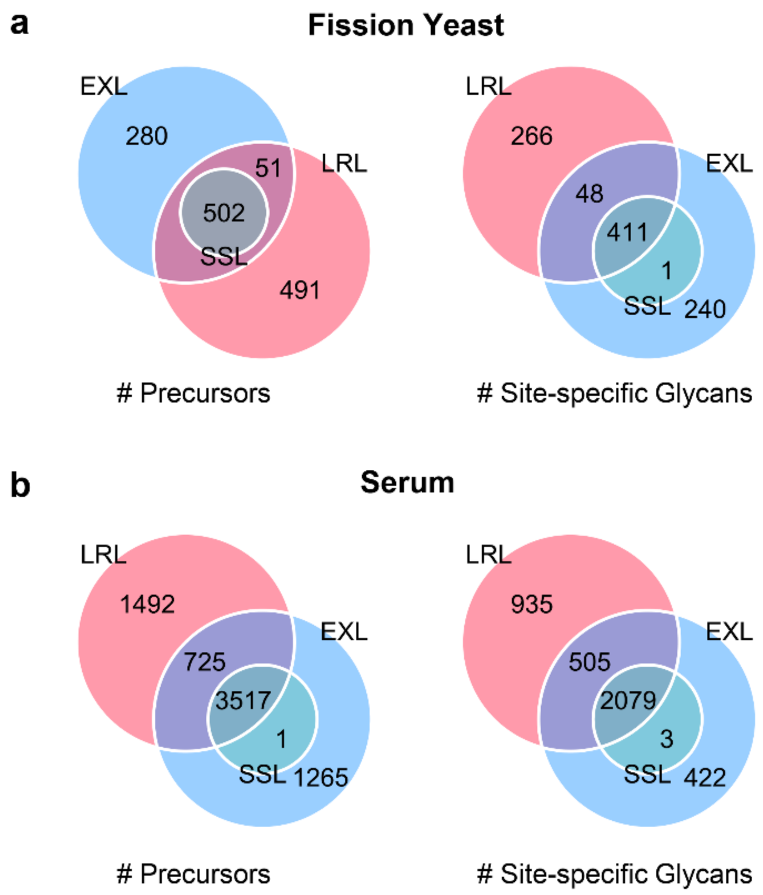

Supplementary Fig. 25. Coverage comparison of the sample-specific library (SSL), the lab repository-scale library (LRL), and the extended library by the semi-empirical approach (EXL). (**a**) Libraries for the fission yeast sample. (**b**) Libraries for the human serum sample.

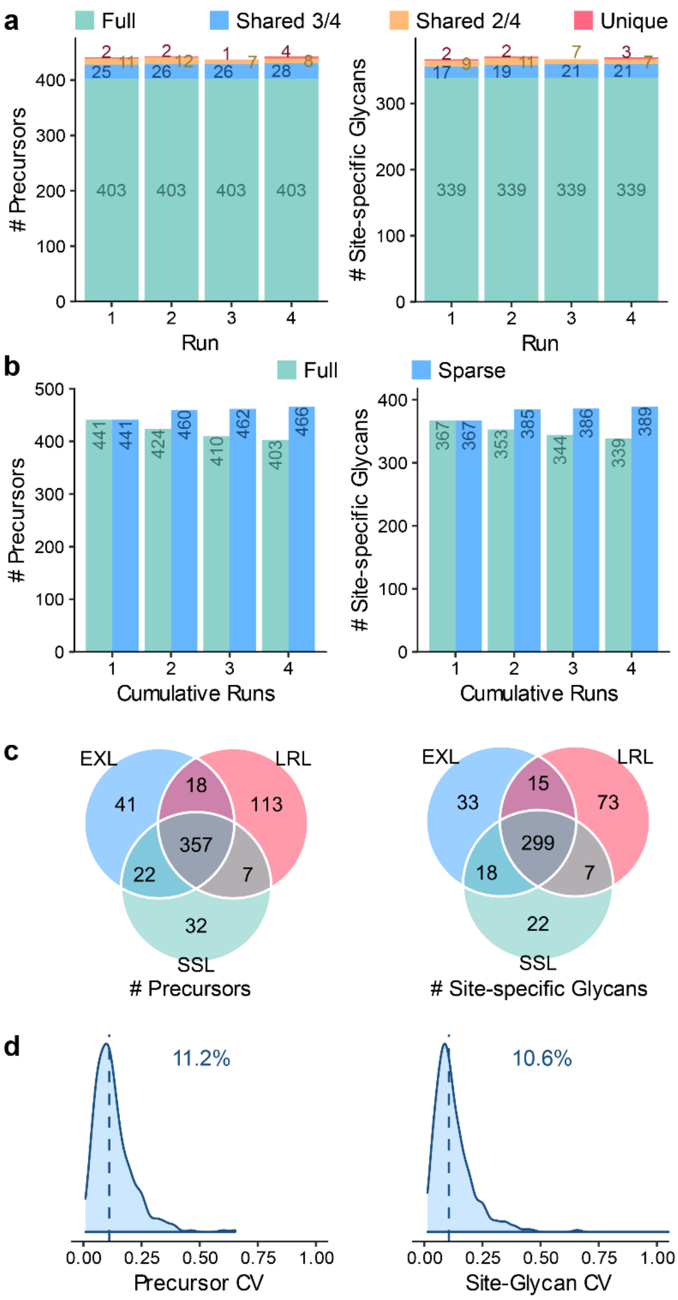

Supplementary Fig. 26. DIA results of the fission yeast sample using the extended library at the level of precursor and site-specific glycan. (**a**) Numbers of identifications per run. "Full" represents identifications observed in all the runs; "shared >50%" represents identifications observed in 3 runs; "shared ≤50%" represents identifications observed in 2 runs; "unique" represents identifications observed in only 1 run. (**b**) Numbers of cumulative identifications from run 1 to 4. "Full" represents identifications shared in the cumulative runs; "sparse" represents identifications observed in at least one run in the cumulative runs. (**c**) Comparison of numbers of identifications shared in >50% runs using the sample-specific library (SSL), the lab repository-scale library (LRL), and the extended library (EXL). (**d**) Coefficients of variation (CVs) of quantification results by DIA with EXL. Medians are indicated. Source data are provided as a Source Data file.

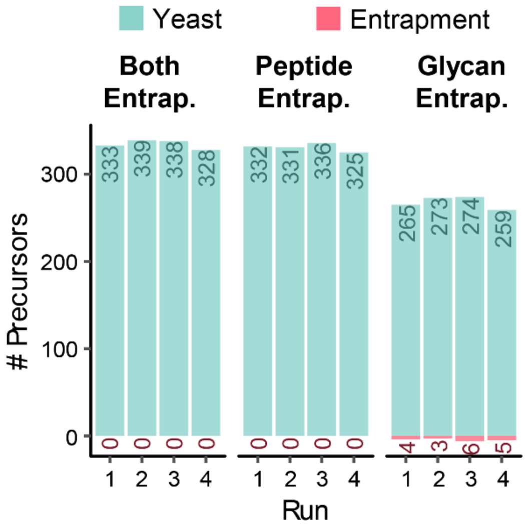

Supplementary Fig. 27. Numbers of identifications from the fission yeast sample using the extend libraries with entrapment glycopeptides.

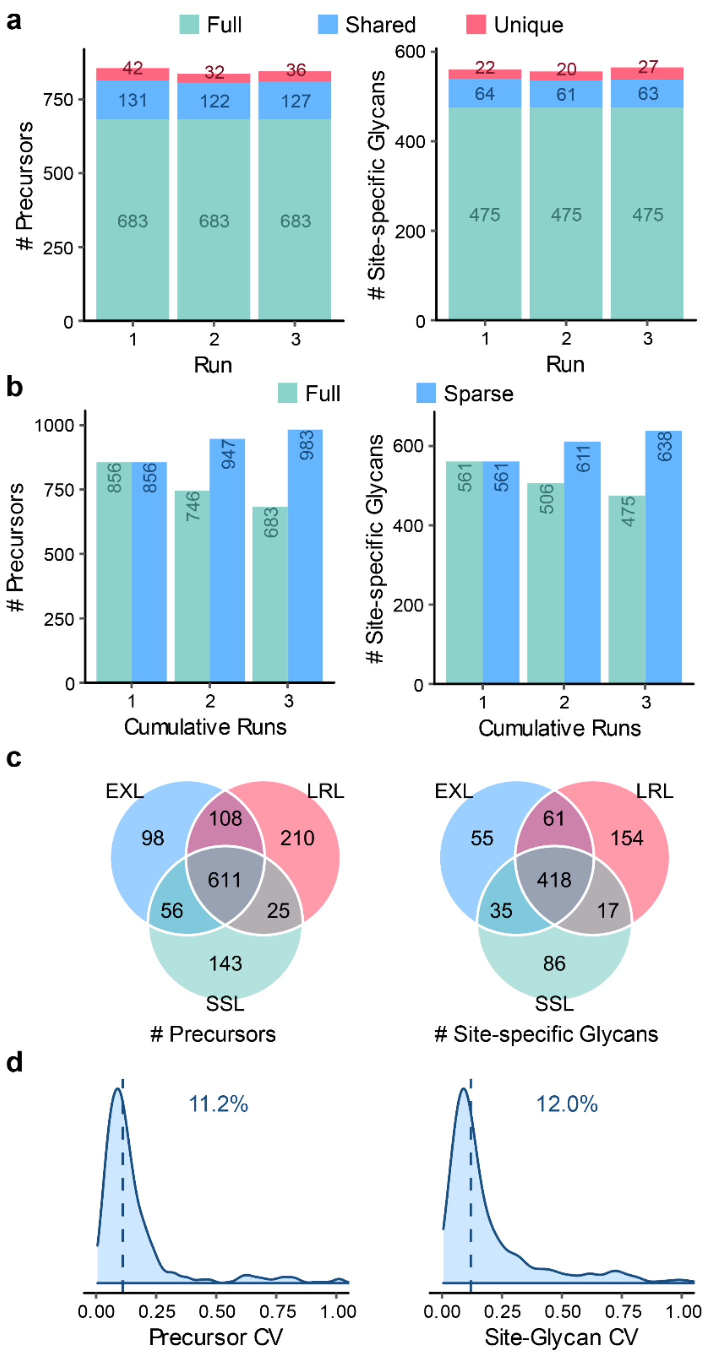

Supplementary Fig. 28. DIA results of the human serum sample using the extended library at the level of precursor and site-specific glycan. (**a**) Numbers of identifications per run. "Full" represents identifications observed in all the runs; "shared" represents identifications observed in 2 runs; "unique" represents identifications observed in only 1 run. (**b**) Numbers of cumulative identifications from run 1 to 3. "Full" represents identifications shared in the cumulative runs; "sparse" represents identifications observed in at least one run in the cumulative runs. (**c**) Comparison of numbers of identifications shared in >50% runs using the sample-specific library (SSL), the lab repository-scale library (LRL), and the extended library (EXL). (**d**) Coefficients of variation (CVs) of quantification results by DIA with EXL. Medians are indicated. Source data are provided as a Source Data file.

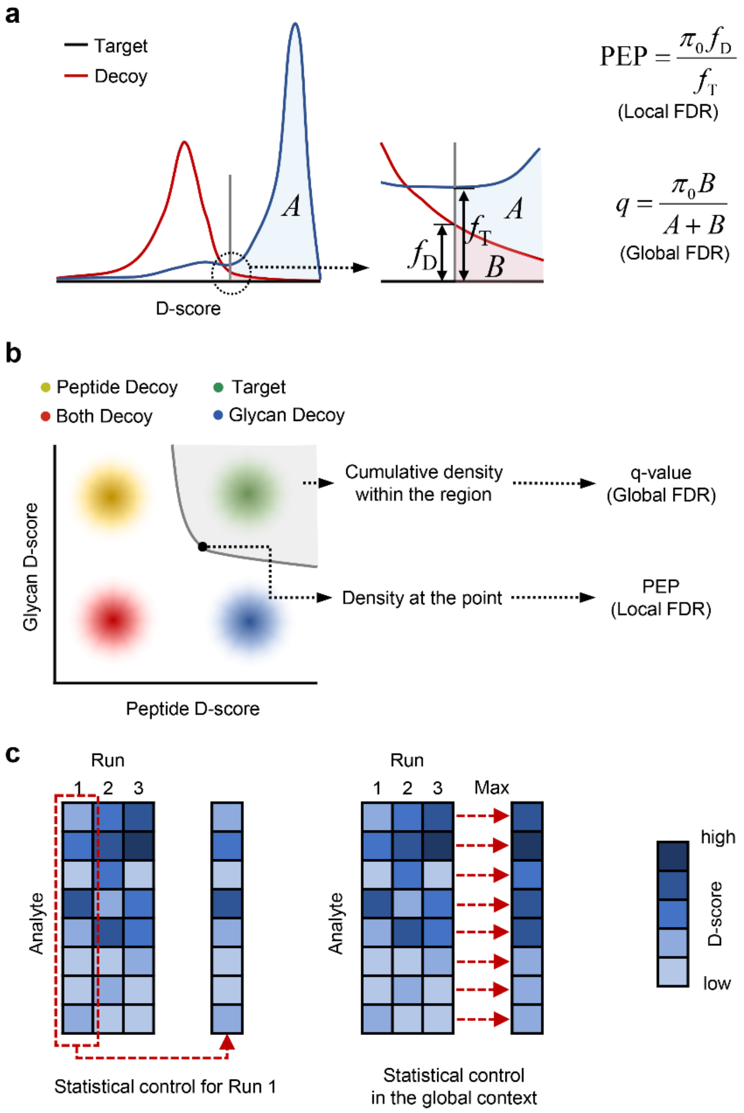

Supplementary Fig. 29. Schematic illustration of the concept of local FDR (PEP) and global FDR (q-value), as well as q-value in the global context. (**a**) Local FDR and global FDR in the one-dimensional condition. Local FDR is calculated from the density of the target and decoy distributions at a specific point (f_T and f_D), whereas global FDR is calculated from the area of the target and decoy distributions with the reported region (A+B and B). (**b**) Local FDR and global FDR in the two-dimensional condition. For simplicity, the bivariate distribution is shown in the top view, and color gradient indicates the density relatively. (**c**) Statistical control in the run-specific context (left) and in the global context (right). The run-specific context conducts separate error rate estimation for each run, whereas the global context only considers the best-scoring peak group per analyte across the entire experiment. Color gradient indicates the D-score relatively.

Supplementary Note 1. Optimization of the search space of glycoform inference

When generating identification transitions for glycoform inference, only the top n_{bg} background glycan structures were included in the spectral library to limit the search space. We tested different n_{bg} from 20 to 70 on the fission yeast data by the entrapment approach. The results using glycan entrapment libraries are presented in **Supplementary Fig. 12**. After applying 1% glycoform-level q-value filter, the entrapment percentage declined from 1.5% to 0.6% with the increase of n_{bg} from 20 to 70. When n_{bg} = 50, the entrapment percentage was ~1%. Therefore, we chose 50 for a trade-off between accuracy and size of search space.

Supplementary Note 2. Performance comparison of comprehensive statistical control by GproDIA with the peptide-only FDR control

We compared the statistical control of glycopeptide error rates by GproDIA with the peptide-only FDR control approach designed for peptide DIA analysis on the fission yeast data using the entrapment strategy. Two glycan entrapment libraries were built: (i) one only contains peptide b/y, b-N_1/y-N_1 and $b_\$$/$y_\$$ fragments, excluding all Y ions to follow the reported strategies where spectral libraries were generated from deglycosylated peptides[1,2] or peptides with truncated glycans[3]; (ii) the other contains both peptide fragments and Y ions. The entrapment glycopeptides were built with peptide sequences from yeast and glycans from human. Only peptide decoys were generated and appended to the entrapment libraries, while no glycan decoys were used. The fission yeast data were analyzed using the OpenSWATH-PyProphet pipeline[4,5] for peptide analysis. When scoring the peak groups using PyProphet, both MS1 and MS2 features were considered by setting the parameter "--level=ms1ms2". Peak group q-value cutoff was 1%. Peptide/protein inference and subsequent steps were not performed. The results are presented in **Supplementary Fig. 13**. The peptide-only FDR approach for peptides cannot address error rate control for glycopeptides properly, which again stresses the significance of comprehensive statistical control by the 2D FDR and glycoform inference.

Supplementary Note 3. Benchmarking using synthetic glycopeptides.

We further benchmarked the performance of GproDIA to differentiate glycoforms with near identical masses on data of a synthetic glycopeptide sample. The sample contained 14 synthetic glycopeptides of 7 peptide sequences and 2 sialylated glycans for each sequence (**Supplementary Table 2**). DDA with 1 h LC gradient and 3 repeat injections was used to build a spectral library. Three entrapment glycans were generated for each peptide sequence by replacing 1 NeuAc with 2 Fuc monosaccharides, where the mass difference between 1 NeuAc and 2 Fuc is ~1 Da. The entrapment library was generated using the semi-empirical approach. DDA data of fucosylated glycopeptides with the 3 entrapment glycans were collected from previous projects of our lab, and then used to generate the semi-empirical entrapment library entries with the DDA data of the synthetic peptides. For coverage completeness of the semi-empirical entrapment entries, the number of nearest neighbors (*k*) was set to 1. When generating the spectral library for glycoform inference, 9 glycan structures of the 2 synthetic and 3 entrapment glycans were appended to the background glycan database for human serum, and the whole background glycan database contained 3071 glycan structures with duplicates removed. The spectral library finally contained 25 precursors of the 14 synthetic glycopeptides and 41 precursors of 21 entrapment glycopeptides (**Supplementary Table 1**).

DIA was performed with 1 h LC gradient and 3 repeat injections. Since the same LC condition was used for DDA and DIA, retention time normalization was not performed and no anchor TraML file was specified. Multi-run alignment was not performed, and no global glycopeptide-level q-value filter was applied. After applying 1% peak group-level q-value and 1% glycoform-level q-value filter, 54 peak groups were reported, including 2 (~4%) entrapment peak groups. From the 3 DIA replicate runs, 13 of the 14 glycopeptides (93% recall) were detected totally, while 1 entrapment glycopeptides were reported (**Supplementary Data 10** and **Supplementary Fig. 14**). The results demonstrate that comprehensive statistical control by GproDIA can distinguish glycoforms with near identical masses in a large part. It should be noted that in the peptide centric DIA approach, a high-quality sample specific library by DDA can largely rule out the false identification between NeuAc containing glycan and Fuc

containing glycan. Together with the statistical control by GproDIA, the false identification between NeuAc and Fuc in the final DIA identification results should not be significant.

Supplementary Note 4. Benchmarking statistical control on human serum sample using the entrapment strategy

The performance of statistical control was further tested on the human serum data using the entrapment strategy. Glycan entrapment libraries were built by adding entrapment glycopeptides with peptide sequences from human and glycans from the model higher plant *Arabidopsis thaliana* to the human serum SSL library (**Supplementary Table 1**). LC-MS/MS data of *A. thaliana* glycopeptides were collected from previous projects of our lab. Only the plant glycans containing a xylose monosaccharide were used as entrapment, and thus there was no overlap between the human and entrapment glycans. The human serum DIA data were analyzed using GproDIA without or with glycoform inference, respectively. When generated the spectral library for glycoform inference, 289 glycan structures of *A. thaliana* from the pGlyco3 plant glycan database[6] were appended to the background glycan database for human serum, and the whole background glycan database contained 3274 glycan structures with duplicates removed. As the human entrapment library was much larger than the fission yeast entrapment libraries and required a large amount of computing resources for DIA data analysis, the maximum number of potential glycoforms was set to 30 to reduce the search space of glycoform inference. The peptide-only FDR control approach with peptide decoys only was also conducted for comparison purpose (**Supplementary Note 2**).

The DIA analyses results are presented in **Supplementary Data 13** and **Supplementary Fig. 17**. In comparison with the peptide-only FDR control approach, comprehensive statistical control by GproDIA greatly improved the performance error rate control. With 1% glycoform-level q-value cutoff, the entrapment percentage declined to 2.4%. Notably, the compositions of the entrapment glycans were very similar to the human glycans except for their core xylose, and this test was aimed at exploring the performance of statistical control in a worst-case scenario that barely distinguishable glycome is queried against data of complex samples.

Supplementary Note 5. Examination of oxonium ions in DIA data

In DDA data analysis of glycopeptides, glycan oxonium ions are usually utilized as diagnostic peaks and aid in identification of different types of glycans[7]. In DIA data, however, oxonium ions are shared by glycopeptides with different glycans in the same isolation window, resulting in highly convoluted elution profiles that are not efficient to determine the glycans. Therefore, instead of utilizing oxonium ions as transitions for feature scoring, we examined the presence of oxonium ions in the apex MS2 spectrum of each scored feature (peak group) in the DIA results after statistical control. The following oxonium ions were considered[7]: $[M+H]^+$ *m/z* = 109.03 ($C_6H_4O_2$), 115.04 ($C_5H_6O_3$), 127.04 ($C_6H_6O_3$, Hex−$2H_2O$), and 163.06 ($C_6H_{10}O_5$, Hex) originated from Hex; 126.05 ($C_6H_7NO_2$), 138.05 ($C_7H_7NO_2$), 144.06 ($C_6H_9NO_3$), 168.06 ($C_8H_9NO_3$, HexNAc−$2H_2O$), 186.07 ($C_8H_{11}NO_4$, HexNAc−H_2O), and 204.08 ($C_8H_{13}NO_5$, HexNAc) originated from HexNAc; 274.08 ($C_{11}H_{15}NO_7$, NeuAc−H_2O) and 292.10 ($C_{11}H_{17}NO_8$, NeuAc) originated from NeuAc; 366.13 ($C_{14}H_{23}NO_{10}$, Hex+HexNAc) originated from Hex and HexNAc. For each peak group in the DIA results, the presence of oxonium ions specific to every type of monosaccharides in the reported glycan can support the glycan identification, while the presence of oxonium ions specific to other types of monosaccharides does not indicate a false identification due to the possible co-fragmentation of precursors in DIA. In the human serum DIA results, the vast majority of identifications were supported by oxonium ions, except for only 3 out of the 2398 peak groups detected using the SSL library and 5 out of the 2774 peak groups detected using the LRL library, where oxonium ions were not observed for at least one type of monosaccharides (**Supplementary Data 11** and **12**).

Supplementary Note 6. Validation of the DIA identification results by targeted MS/MS

Among the DIA identification results of the human serum data using the serum SSL library with glycoform inference enabled, there were 265 site-specific glycans (corresponding to 322 glycopeptide precursors) missed by DDA, considering the identifications shared in 2/3 runs (**Fig. 4c**). Targeted MS/MS experiments were performed to validate these glycopeptide precursors. The 322 precursors were divided into 16 groups, i.e., 14 groups containing 20 precursors and the other 2 groups containing 21 precursors per group. Each group was analyzed with one LC-MS/MS injection. The mass spectrometer altered between a MS1 scan and a sequence of MS2 scans for the target precursors, and the cycle was repeated throughout the whole LC gradient (1 h). The isolation window was 4 Da width. The other parameters were the same as those in DDA.

The raw data files were converted to Mascot generic format (MGF) files using MSConvert from ProteoWizard (version 3.0.11537)[8] with the peak picking algorithm set to vendor. The MS2 spectra were annotated with gLabel in pGlyco3[6] and manually examined. The results are shown in **Supplementary Data 14**. Among the 322 target precursors and 265 site-specific glycans, 236 precursors and 194 site-specific glycans (73%) were validated with MS2 evidences observed, and they were distributed throughout the intensity range of DIA quantification results (**Supplementary Fig. 18**). It should be noticed that we used the targeted MS/MS to support the identification results by DIA, which does not indicate that the glycopeptides not observed by the targeted MS/MS were wrong.

Supplementary Note 7. Limitations of the 2-dimensional FDR approach for small datasets.

For the peptide part of the 2-dimensional FDR approach, the reverse decoy approach was inherited from the DIA analysis method for non-glycosylated peptides. For the glycan part, random mass shifts were performed on the glycan fragment peaks, which was initially reported in pGlyco[7]. Typically, the peptide FDR approach based on reverse decoys is applied for large datasets, and may be biased for small datasets.

The debate on how the decoy peptides should be assembled has been around for more than ten years. In 2009, Shen et al. benchmarked different decoy strategies for DDA database searching on a mixture of 19 model proteins, which actually was not a large dataset, demonstrating that the target-decoy strategy with decoy being the reversed version of the target database is a practically simple and reasonably accurate strategy for the estimation of false positives, which could also improve sensitivity[9]. Lam et al. extended the target-decoy approach to spectral library searching, where decoy spectra were generated instead of decoy sequences[10]. Zhang et al. compared different decoy strategies for spectral library searching, showing that the reverse method yielded slightly fewer identifications than the random method, but the differences in these methods were rather small[11]. Many DIA analysis software tools inherit the decoy strategies for spectral library searching. For example, OpenSWATH provides options to use the simplest reverse method or the shuffle method by Lam et al. to create the decoy transitions (https://abibuilder.informatik.unituebingen.de/archive/openms/Documentation/release/latest/html/TOPP_OpenSwathDecoyGenerator.html).

The principle of decoy transitions in peptide-centric DIA analyses is conceptually related to but different from the decoy database approach used for DDA database searching. In contrast to the decoy database approach in DDA that operates on the database searching level, the decoy transitions in DIA are introduced at the measurement level[12]. Notably, the decoy strategy in DIA analyses was initially proposed for selected reaction monitoring (SRM) feature scoring. In our study, the spectral libraries (except the synthetic dataset) contain hundreds to thousands of

glycopeptide precursors (more than 1000 if including entrapments), which is not much fewer than the peptide assay in an ordinary SRM experiment.

In the statistical control step of DIA analyses, a semi-supervised learning algorithm is executed iteratively to optimize the weights of individual score combinations to separate targets and decoys[13]. Sufficient numbers of training data are normally necessary for the semi-supervised algorithm. Also, the decoy distribution should match the true-negative part of the target distribution. If the spectral library is too small, these conditions may not be fulfilled, and the machine learning and FDR estimation may be biased. In this study, however, we used the entrapment strategy to assess the quality of statistical control. Even for the synthetic dataset, which is the smallest dataset in the study, the false positives in the peptide part were still well controlled.

In summary, the current peptide FDR approach has limitation for small datasets. However, it is, to the best of our knowledge, the only choice for peptide-centric DIA analyses at present. Indeed, the emphasis of this work is on algorithms for glycopeptide identification, especially in the glycan part, and the performance of the current method is still acceptable with the validations by different methods, i.e. entrapment library and targeted MS/MS analysis.

Supplementary References

1. Zacchi, L. F. & Schulz, B. L. SWATH-MS glycoproteomics reveals consequences of defects in the glycosylation machinery. *Mol. Cell. Proteomics* **15**, 2435-2447 (2016).
2. Zhou, C. & Schulz, B. L. Glycopeptide variable window SWATH for improved data independent acquisition glycoprotein analysis. *Anal. Biochem.* **597**, 113667 (2020).
3. Ye, Z., Mao, Y., Clausen, H. & Vakhrushev, S. Y. Glyco-DIA: A method for quantitative O-glycoproteomics with in silico-boosted glycopeptide libraries. *Nat. Methods* **16**, 902-910 (2019).
4. Röst, H. L. et al. OpenSWATH enables automated, targeted analysis of data-independent acquisition MS data. *Nat. Biotechnol.* **32**, 219-223 (2014).
5. Rosenberger, G. et al. Statistical control of peptide and protein error rates in large-scale targeted data-independent acquisition analyses. *Nat. Methods* **14**, 921-927 (2017).
6. Zeng, W.-F., Cao, W.-Q., Liu, M.-Q., He, S.-M. & Yang, P.-Y. Precise, Fast and Comprehensive Analysis of Intact Glycopeptides and Monosaccharide-Modifications with pGlyco3. Preprint at http://biorxiv.org/content/early/2021/02/08/2021.02.06.430063 (2021).
7. Zeng, W.-F. et al. pGlyco: A pipeline for the identification of intact N-glycopeptides by using HCD- and CID-MS/MS and MS3. *Sci. Rep.* **6**, 25102 (2016).
8. Chambers, M. C. et al. A cross-platform toolkit for mass spectrometry and proteomics. *Nat. Biotechnol.* **30**, 918-920 (2012).
9. Shen, C. et al. On the estimation of false positives in peptide identifications using decoy search strategy. *Proteomics* **9**, 194-204 (2009).
10. Lam, H., Deutsch, E. W. & Aebersold, R. Artificial decoy spectral libraries for false discovery rate estimation in spectral library searching in proteomics. *J. Proteome Res.* **9**, 605-610 (2010).
11. Zhang, Z. et al. Reverse and random decoy methods for false discovery rate estimation in high mass accuracy peptide spectral library searches. *J. Proteome Res.* **17**, 846-857 (2018).
12. Reiter, L. et al. mProphet: automated data processing and statistical validation for large-scale SRM experiments. *Nat. Methods* **8**, 430-435 (2011).
13. Ludwig, C. et al. Data-independent acquisition-based SWATH-MS for quantitative proteomics: a tutorial. *Mol. Syst. Bio.* **14**, e8126 (2018).
