# Supplementary material for: GproDIA enables data-independent acquisition glycoproteomics with comprehensive statistical control
Source: Nat Commun. 2021 Oct 18;12:6073. doi: 10.1038/s41467-021-26246-3 (PMC8523693; doi:10.1038/s41467-021-26246-3)
Supplement: Supplementary file 2 — Description of Additional Supplementary Files [file 41467_2021_26246_MOESM2_ESM.docx]

File Name: Supplementary information PDF file

Description: Supplementary Tables, Figs. and Notes.

File Name: Supplementary Data 1

Description: Entries in the spectral libraries for the fission yeast and budding yeast samples.

File Name: Supplementary Data 2

Description: DDA results of the fission yeast sample with an 1 h LC gradient.

File Name: Supplementary Data 3

Description: DDA results of the fission yeast sample with a 6 h LC gradient.

File Name: Supplementary Data 4

Description: DIA results of the fission yeast sample using the sample-specific library.

File Name: Supplementary Data 5

Description: DIA results of the fission yeast sample using the lab repository-scale library.

File Name: Supplementary Data 6

Description: DIA results of the budding yeast sample.

File Name: Supplementary Data 7

Description: DDA results of the human serum sample.

File Name: Supplementary Data 8

Description: Entries in the spectral libraries for the human serum sample.

File Name: Supplementary Data 9

Description: DIA results of the fission yeast sample using the entrapment libraries.

File Name: Supplementary Data 10

Description: Entries in the spectral library for the synthetic glycopeptide sample and DIA results with glycoform inference.

File Name: Supplementary Data 11

Description: DIA results with glycoform inference of the human serum sample using the sample-specific library.

File Name: Supplementary Data 12

Description: DIA results with glycoform inference of the human serum sample using the lab repository-scale library.

File Name: Supplementary Data 13

Description: DIA results of the human serum sample using the entrapment libraries.

File Name: Supplementary Data 14

Description: Targeted MS/MS results of the human serum sample.

File Name: Supplementary Data 15

Description: DDA results of the mixed-organism samples.

File Name: Supplementary Data 16

Description: DIA results of the mixed-organism samples using a combined library of the budding yeast library and the serum sample-specific library.

File Name: Supplementary Data 17

Description: DIA results of the mixed-organism samples using a combined library of the budding yeast library and the serum lab repository-scale library.

File Name: Supplementary Data 18

Description: DIA results of the fission yeast sample using the extended library.

File Name: Supplementary Data 19

Description: DIA results of the fission yeast sample using the extended libraries with entrapment glycopeptides.

File Name: Supplementary Data 20

Description: DIA results of the human serum sample using the extended library.
