# Supplementary material for: GproDIA enables data-independent acquisition glycoproteomics with comprehensive statistical control
Source: Nat Commun. 2021 Oct 18;12:6073. doi: 10.1038/s41467-021-26246-3 (PMC8523693; doi:10.1038/s41467-021-26246-3)
Supplement: Supplementary file 16 — Supplementary Data 14 [file 41467_2021_26246_MOESM16_ESM.zip › SData14_targeted_MS2/SData14_targeted_MS2.pdf]

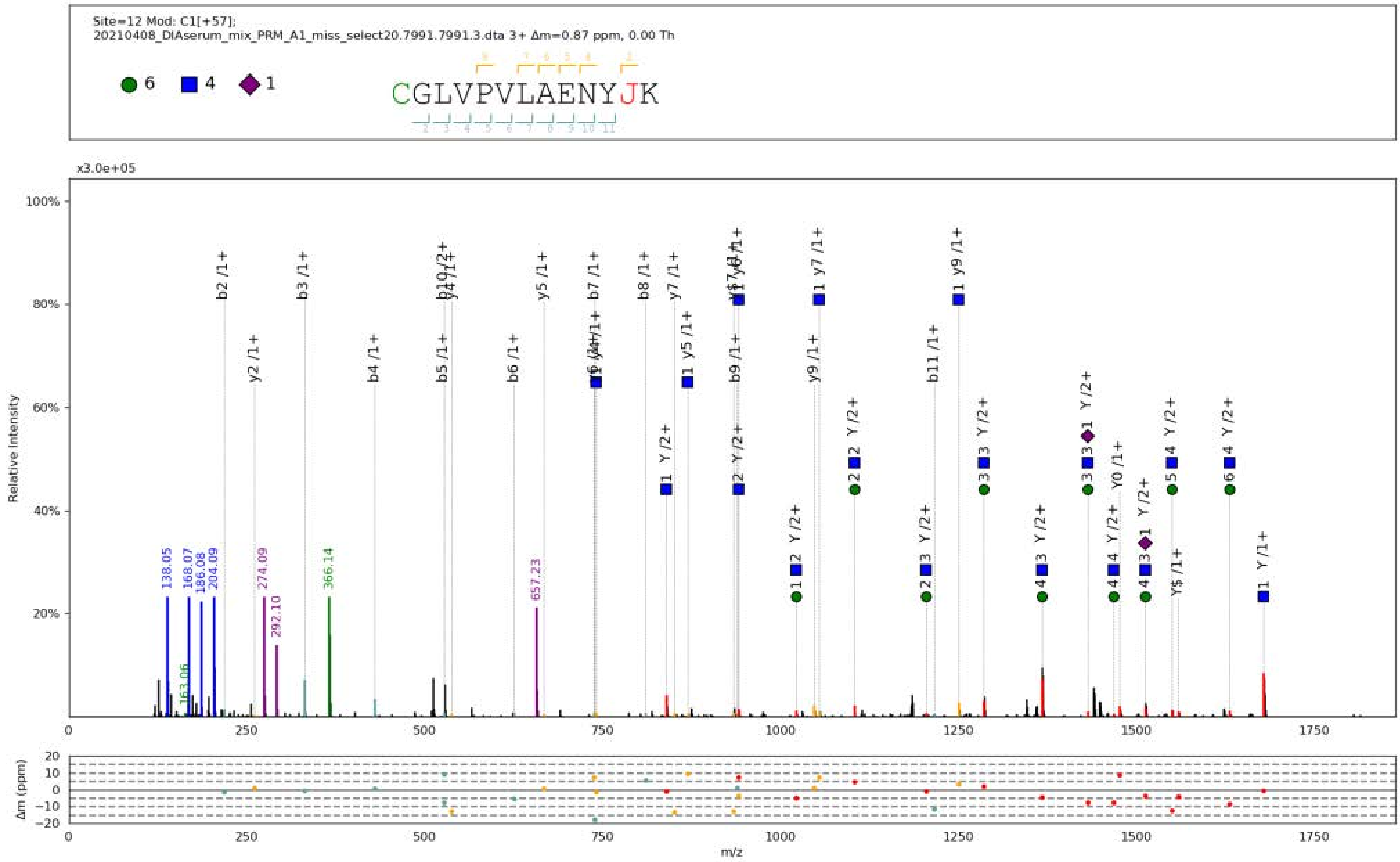

Site=12 Mod: C1[+57];
20210408_DIAserum_mix_PRM_A1_miss_select20.7991.7991.3.dta 3+ Δm=0.87 ppm, 0.00 Th
6
4
1
CGLVPVLAENYJK
x3.0e+05
Relative Intensity
100%
80%
60%
40%
20%
138.05
168.07
186.08
204.09
163.06
274.09
292.10
366.14
657.23
b2 /1+
y2 /1+
b3 /1+
b4 /1+
b5 /1+
b6 /1+
y5 /1+
b7 /1+
b8 /1+
y7 /1+
b9 /1+
y9 /1+
b11 /1+
Y0 /1+
Y /2+
Y /1+
m/z
Δm (ppm)
0
250
500
750
1000
1250
1500
1750

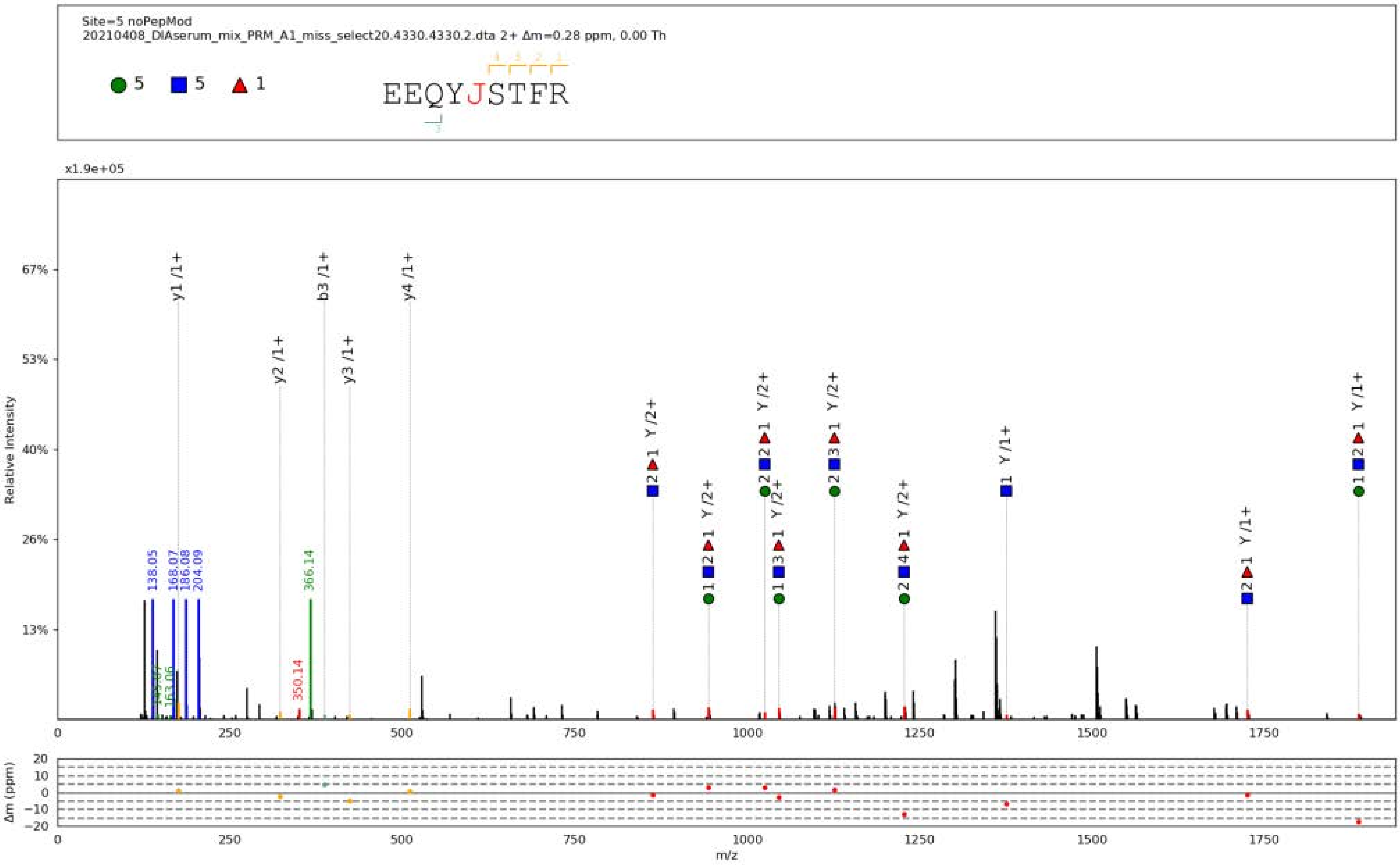

Site=5 noPepMod
20210408_DIAserum_mix_PRM_A1_miss_select20.4330.4330.2.dta 2+ Δm=0.28 ppm, 0.00 Th
5
5
1
EEQYJSTFR
x1.9e+05
Relative Intensity
y1 /1+
b3 /1+
y4 /1+
y2 /1+
y3 /1+
138.05
168.07
186.08
204.09
366.14
350.14
163.06
Y /2+
Y /1+
m/z
Δm (ppm)

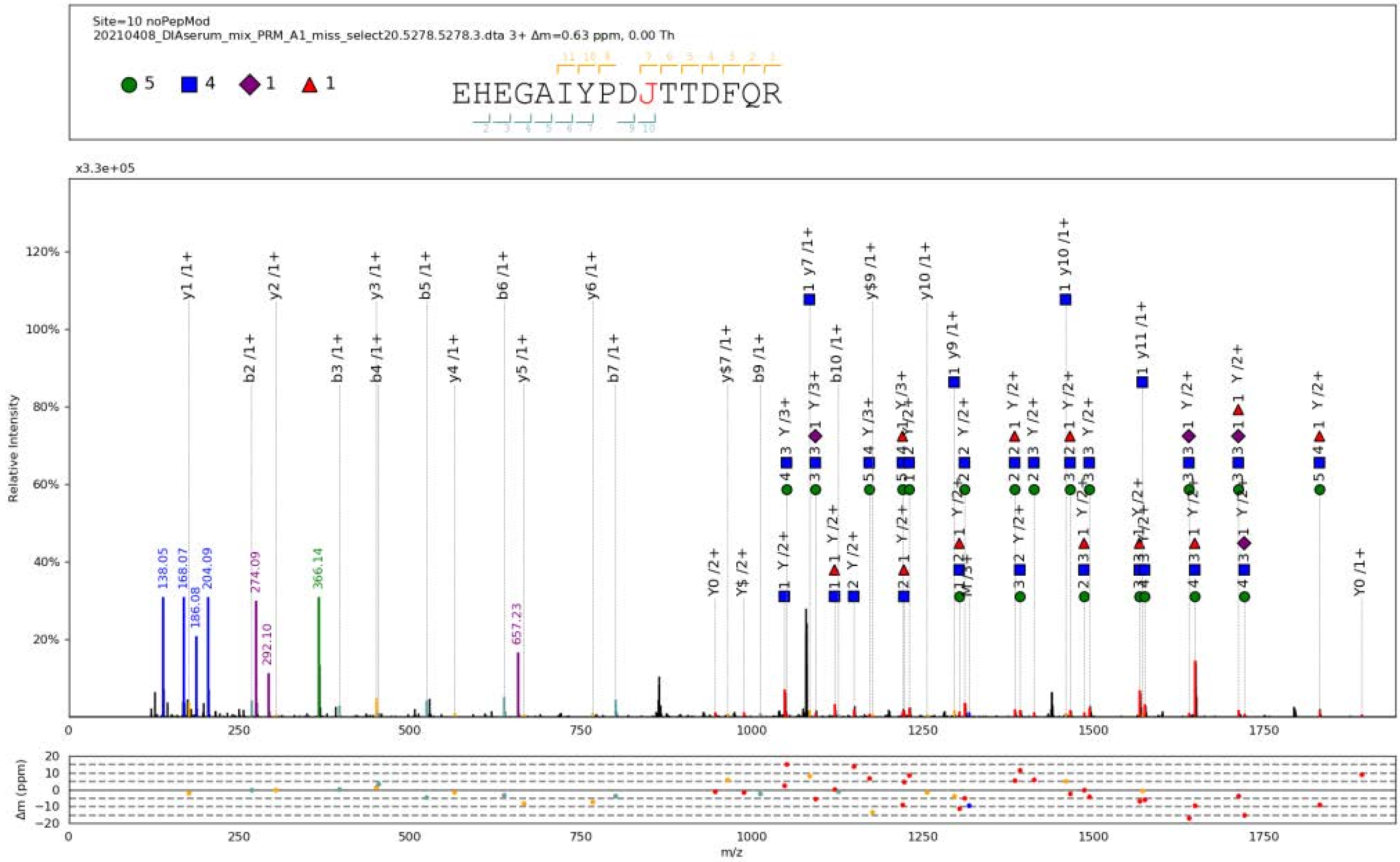
Site=10 noPepMod
20210408_DIAserum_mix_PRM_A1_miss_select20.5278.5278.3.dta 3+ Δm=0.63 ppm, 0.00 Th
EHEGAIYPDJTTDFQR
x3.3e+05
Relative Intensity
Δm (ppm)
m/z

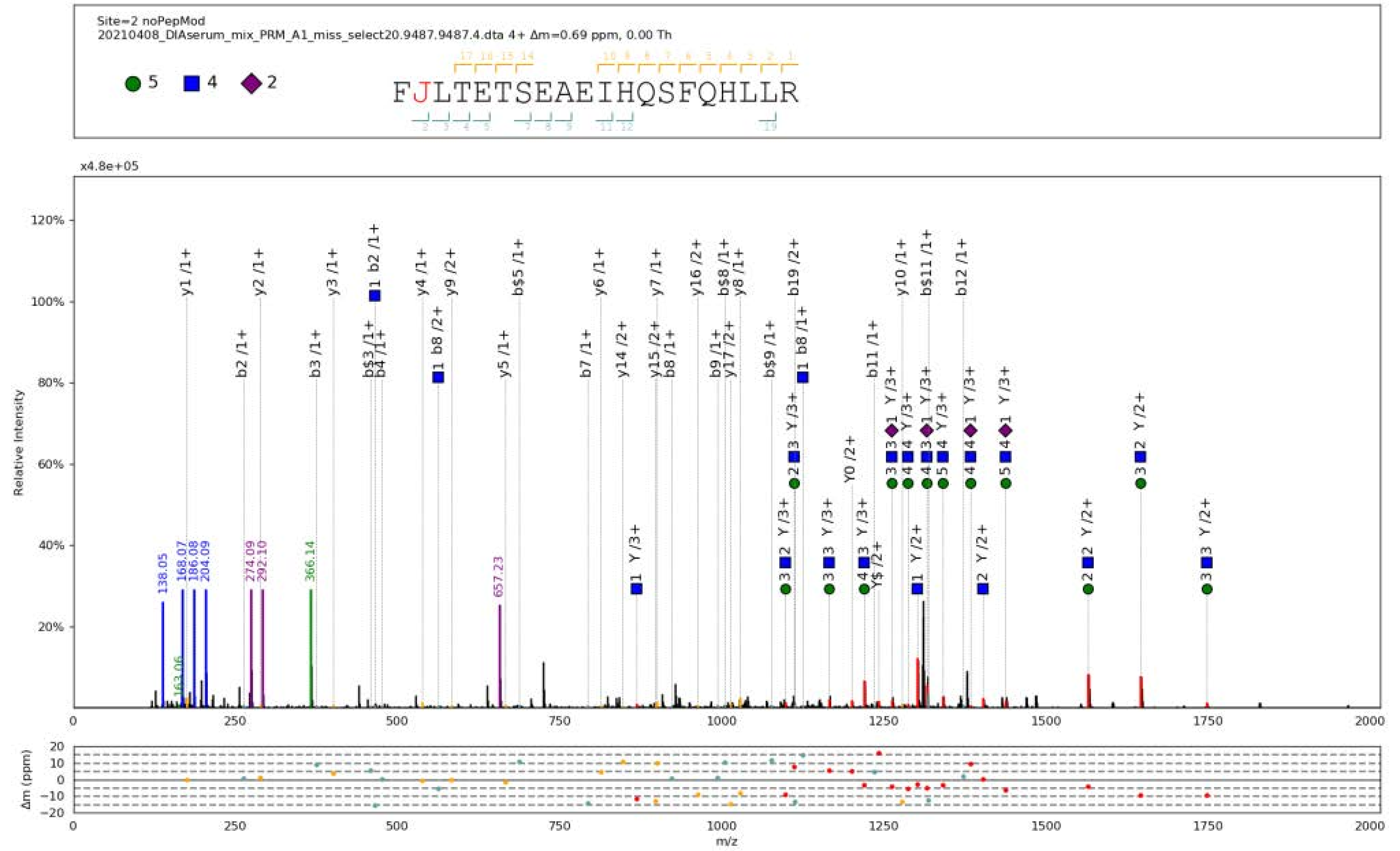

Site=2 noPepMod
20210408_DIAserum_mix_PRM_A1_miss_select20.9487.9487.4.dta 4+ Δm=0.69 ppm, 0.00 Th
5
4
2
FJLTETSEAEIHQSFQHLLR
x4.8e+05
Relative Intensity
y1 /1+
y2 /1+
b2 /1+
b3 /1+
y3 /1+
b$3 /1+
b4 /1+
1 b2 /1+
y4 /1+
1 b8 /2+
y9 /2+
y5 /1+
b$5 /1+
b7 /1+
y6 /1+
y14 /2+
y15 /2+
b8 /1+
y7 /1+
y16 /2+
b9 /1+
y17 /2+
b$8 /1+
y8 /1+
b$9 /1+
b19 /2+
1 b8 /1+
Y0 /2+
b11 /1+
y10 /1+
b$11 /1+
b12 /1+
138.05
168.07
186.08
204.09
163.06
274.09
292.10
366.14
657.23
m/z
Δm (ppm)

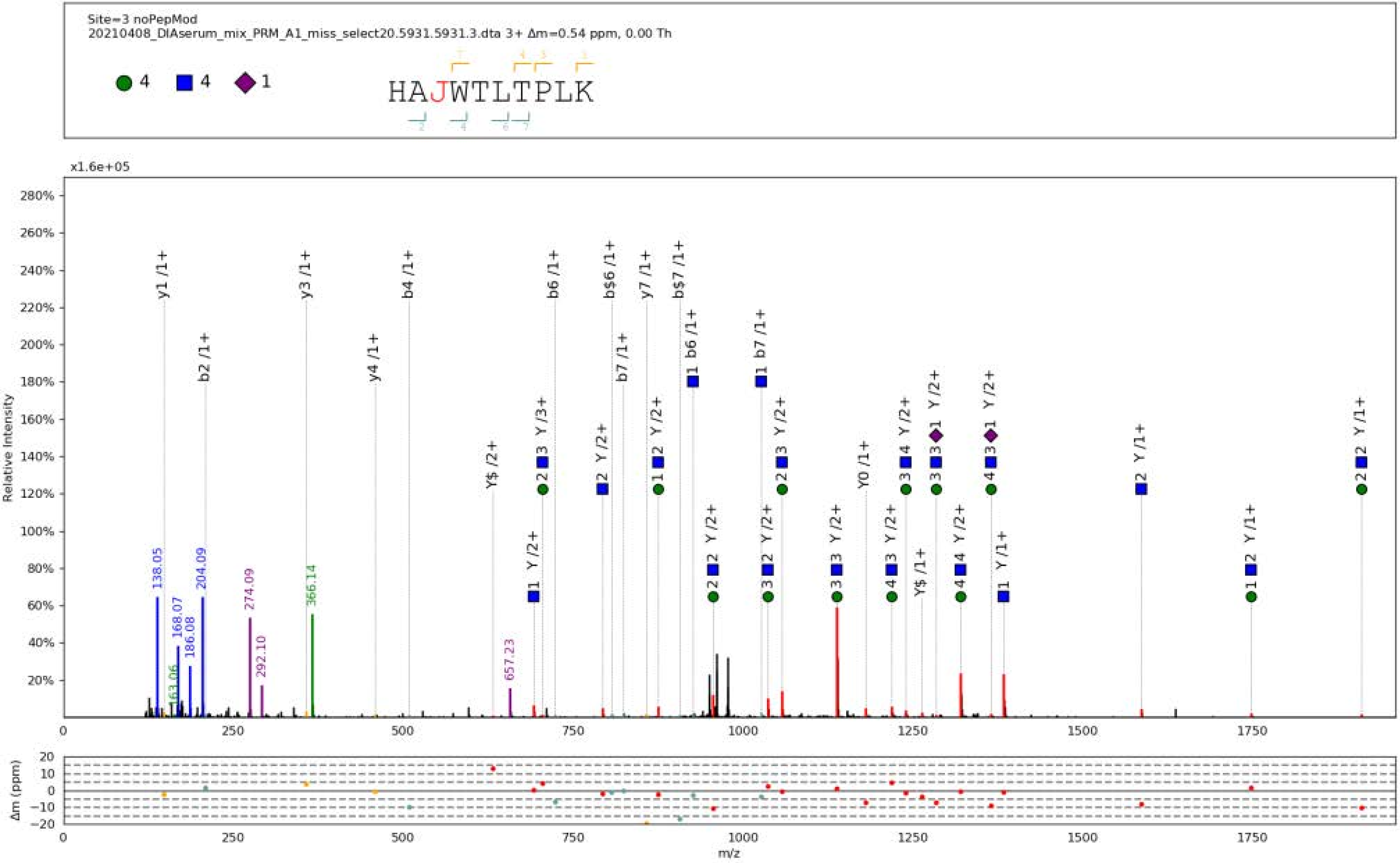
Site=3 noPepMod
20210408_DIAserum_mix_PRM_A1_miss_select20.5931.5931.3.dta 3+ Δm=0.54 ppm, 0.00 Th
HAJWTLTPLK
x1.6e+05
Relative Intensity
Δm (ppm)
m/z

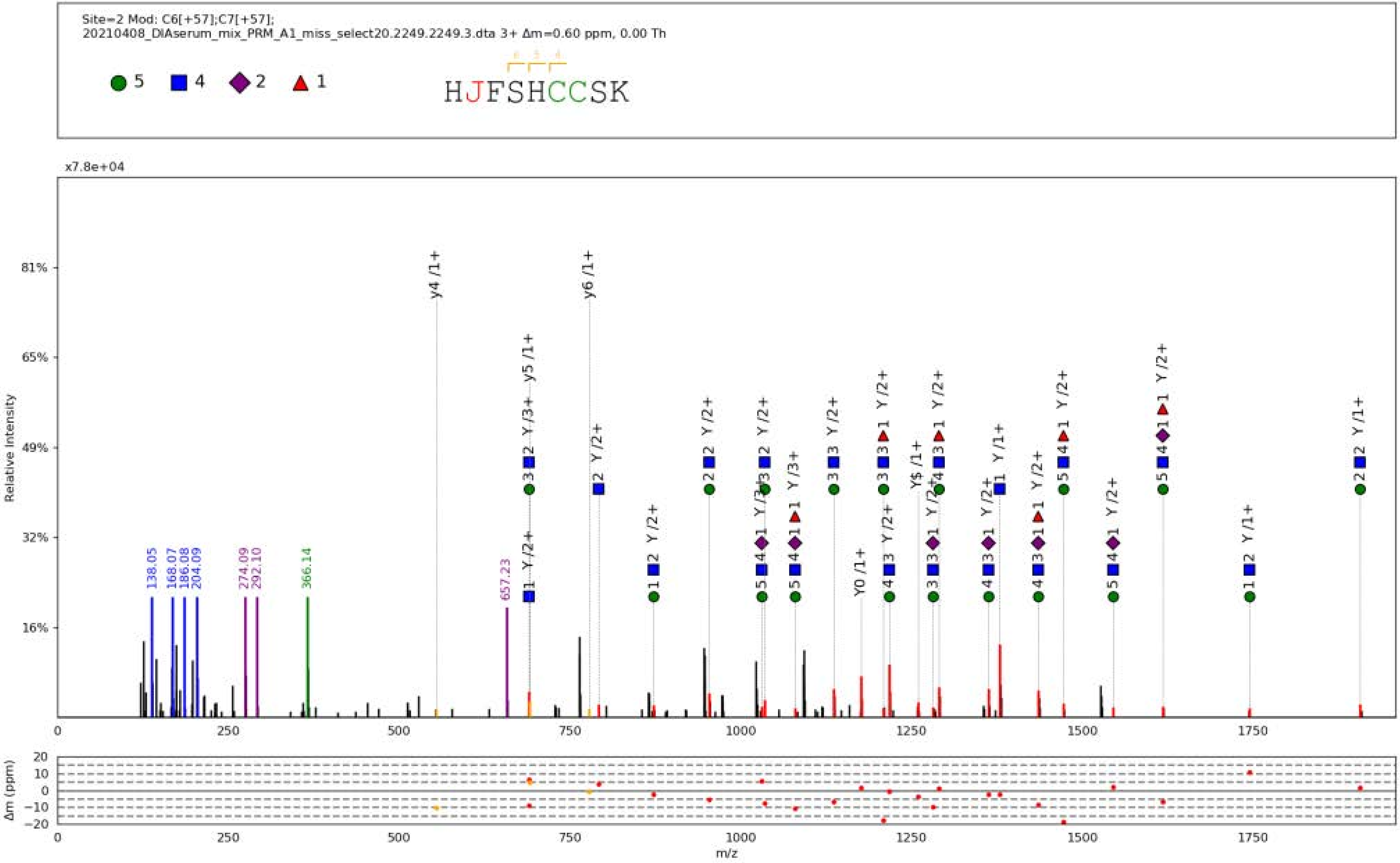
Site=2 Mod: C6[+57];C7[+57];
20210408_DIAserum_mix_PRM_A1_miss_select20.2249.2249.3.dta 3+ Δm=0.60 ppm, 0.00 Th
5
4
2
1
HJFSHCCSK
x7.8e+04
Relative Intensity
81%
65%
49%
32%
16%
138.05
168.07
186.08
204.09
274.09
292.10
366.14
657.23
y4 /1+
y6 /1+
y5 /1+
Y0 /1+
Y$ /1+
Y /1+
Y /2+
Y /3+
m/z
Δm (ppm)
0
250
500
750
1000
1250
1500
1750
20
10
0
−10
−20

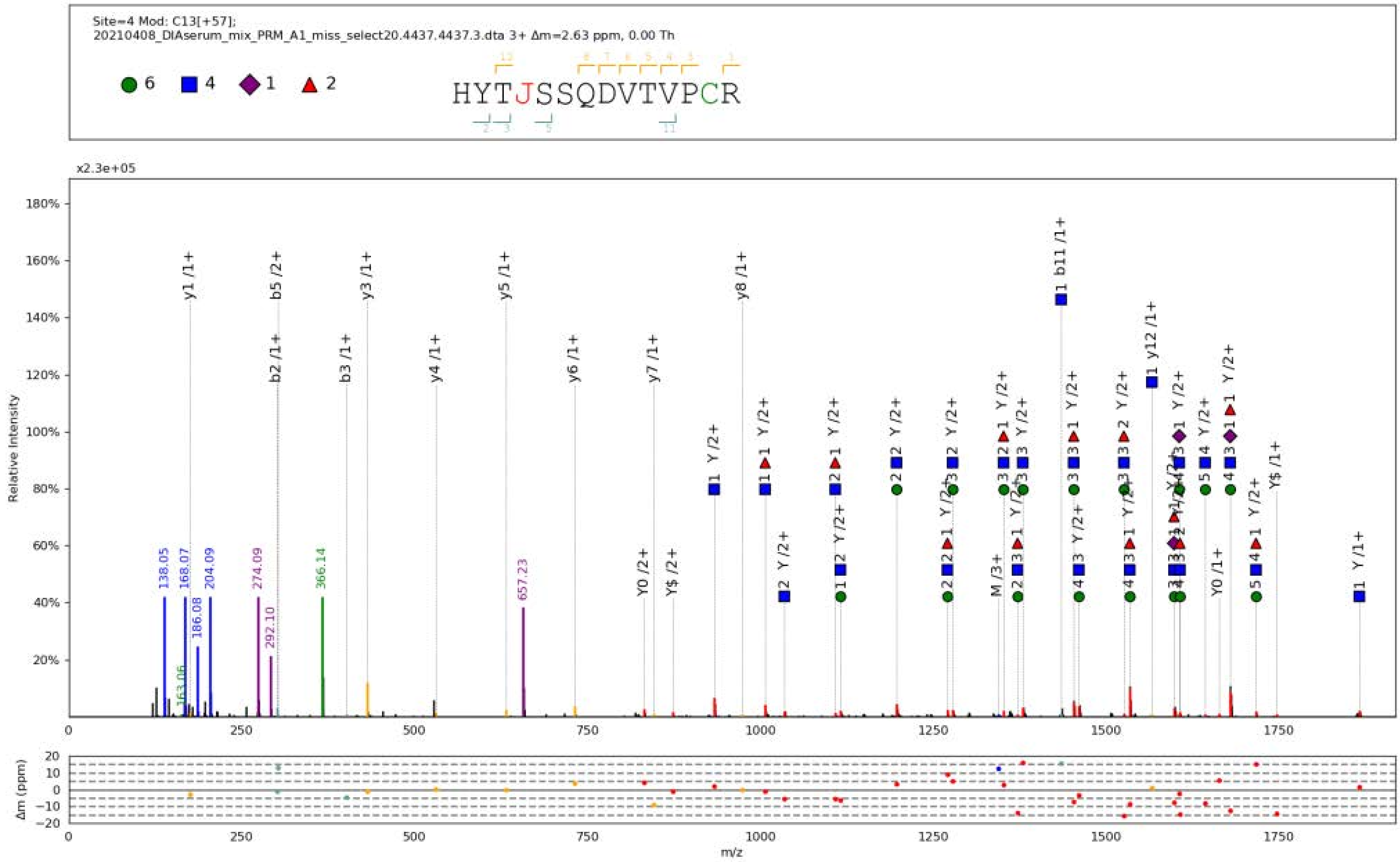

Site=4 Mod: C13[+57];
20210408_DIAserum_mix_PRM_A1_miss_select20.4437.4437.3.dta 3+ Δm=2.63 ppm, 0.00 Th
6 4 1 2
HYTJSSQDVTVPCR
x2.3e+05
Relative Intensity
Δm (ppm)
m/z

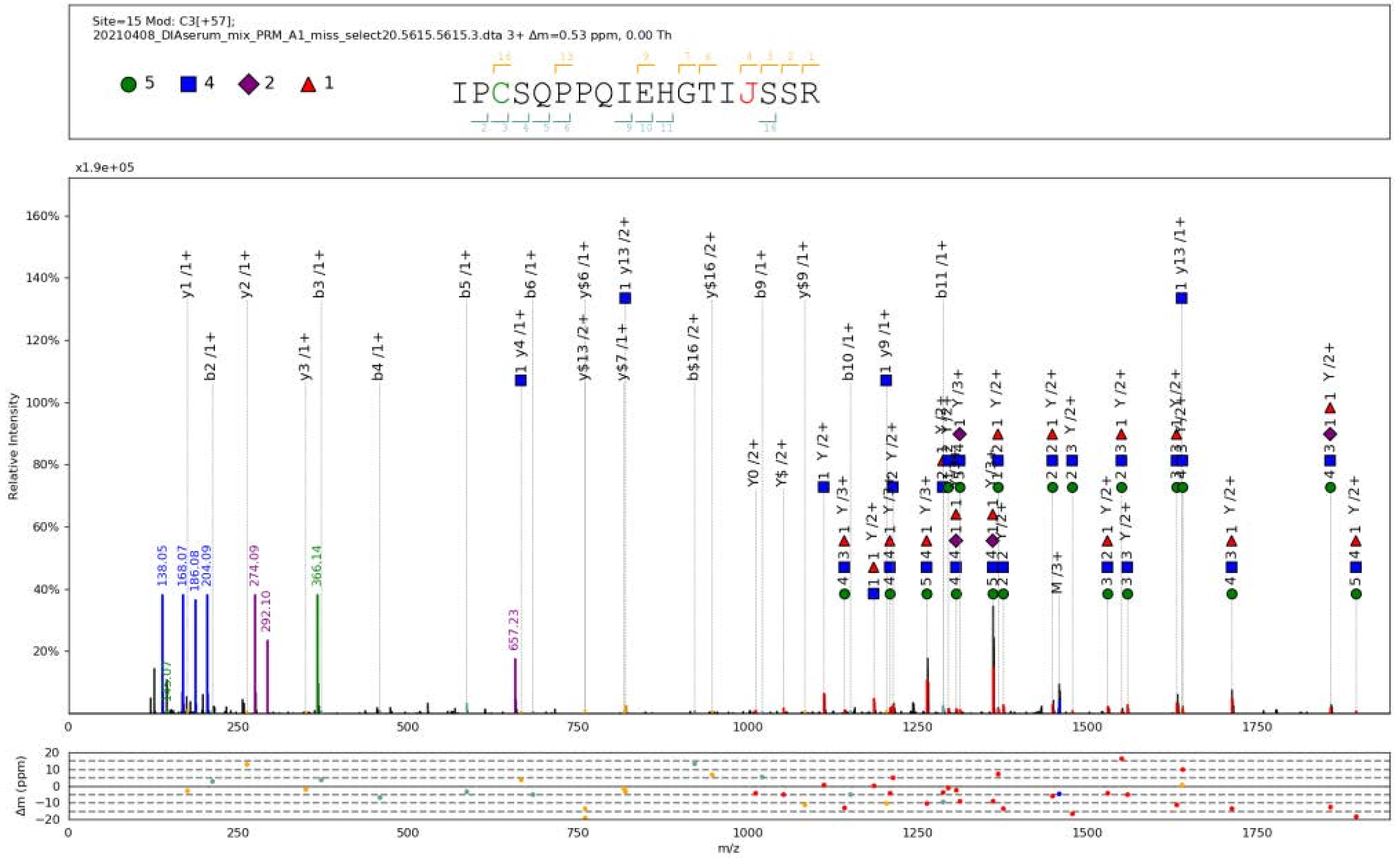
Site=15 Mod: C3[+57];
20210408_DIAserum_mix_PRM_A1_miss_select20.5615.5615.3.dta 3+ Δm=0.53 ppm, 0.00 Th
5 4 2 1
IPCSQPPQIEHGTIJSSR
x1.9e+05
Relative Intensity
m/z
Δm (ppm)
y1 /1+
b2 /1+
y2 /1+
y3 /1+
b3 /1+
b4 /1+
b5 /1+
y4 /1+
b6 /1+
y$13 /2+
y$6 /1+
y$7 /1+
y13 /2+
b$16 /2+
y$16 /2+
Y0 /2+
b9 /1+
Y$ /2+
y$9 /1+
b10 /1+
y9 /1+
b11 /1+
y13 /1+
M /3+
138.05
168.07
186.08
204.09
274.09
292.10
366.14
657.23

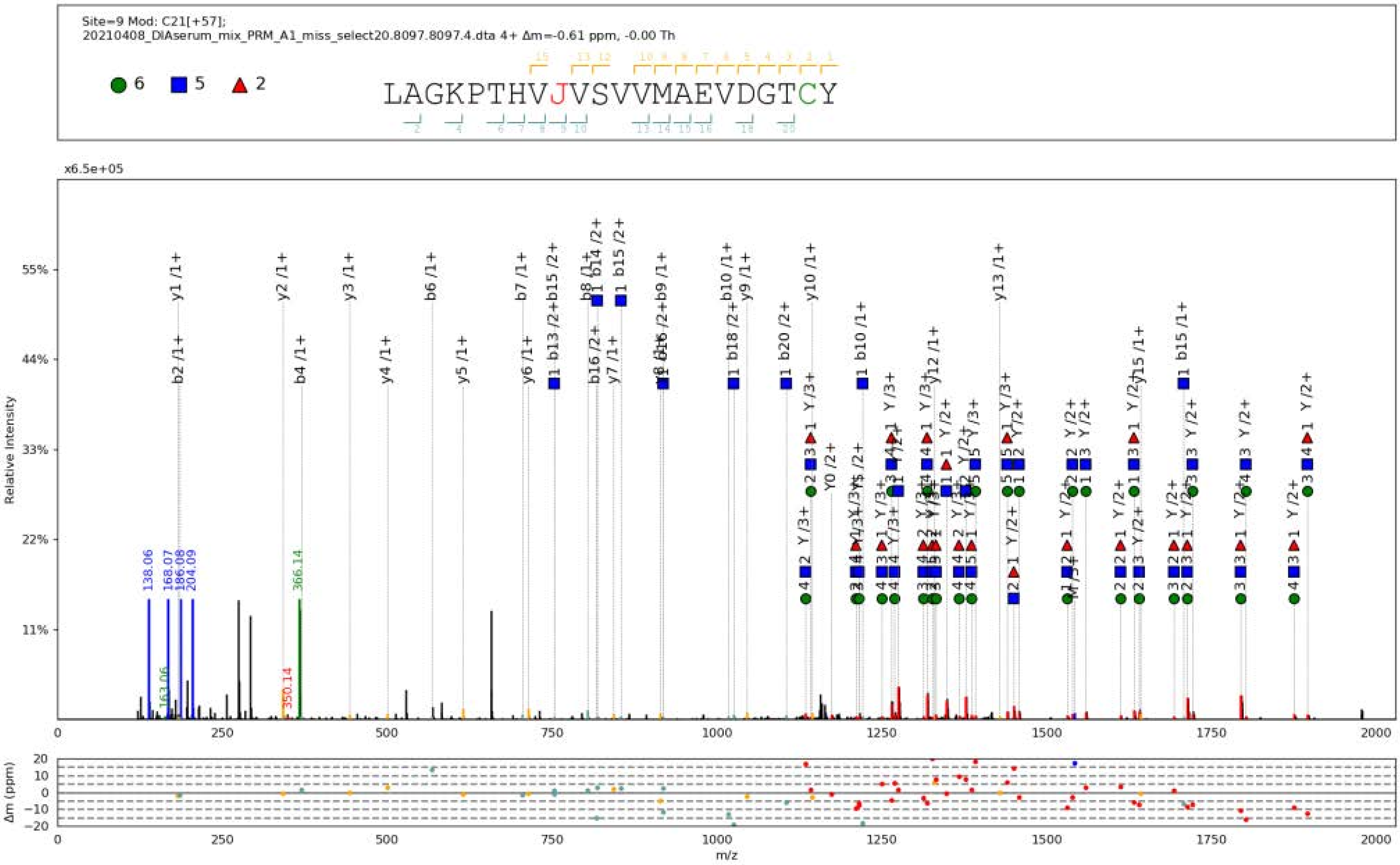

Site=9 Mod: C21[+57];
20210408_DIAserum_mix_PRM_A1_miss_select20.8097.8097.4.dta 4+ Δm=-0.61 ppm, -0.00 Th
6 5 2
LAGKPTHVJVSVVMAEVDGTCY
x6.5e+05
Relative Intensity
55%
44%
33%
22%
11%
m/z
Δm (ppm)

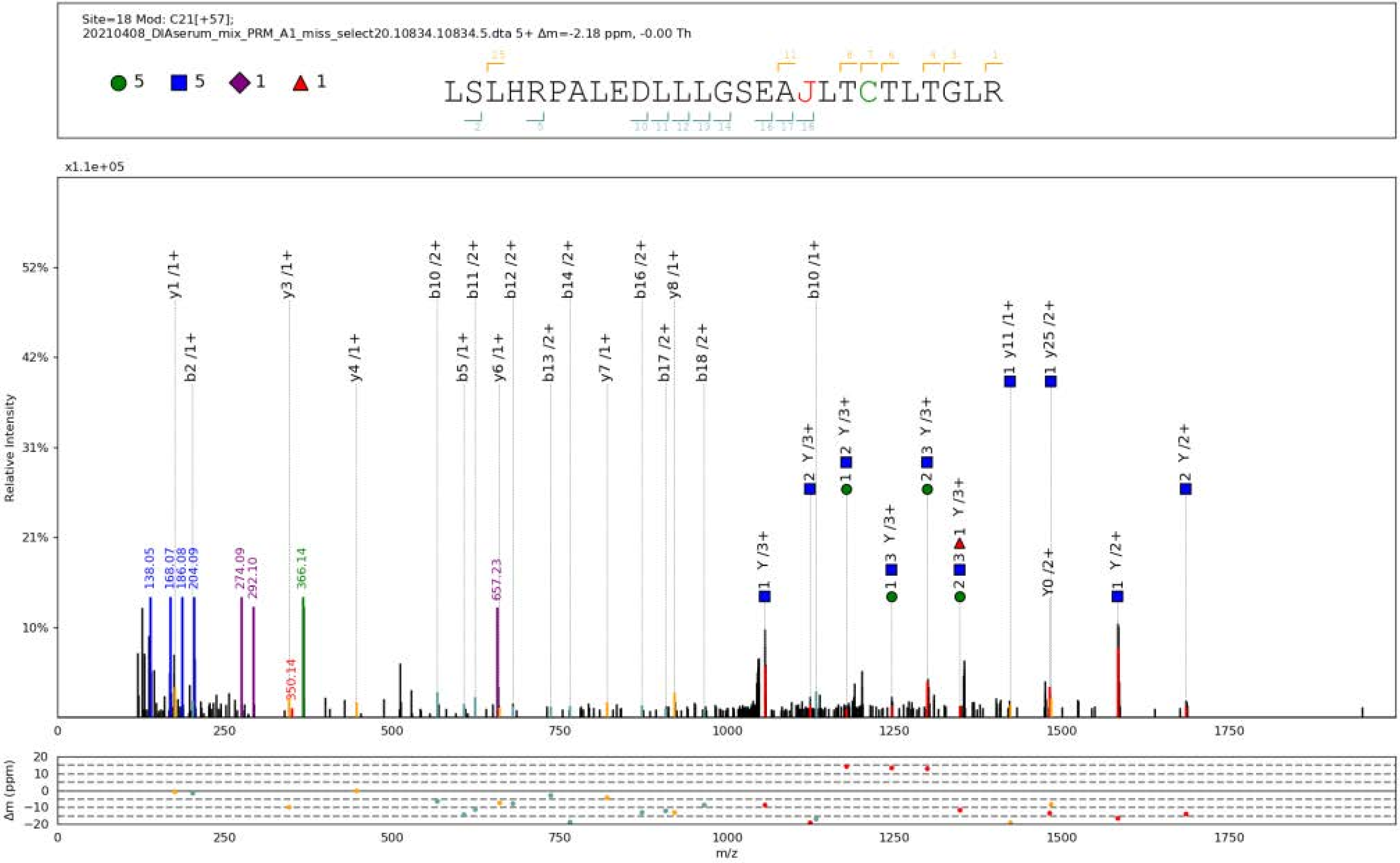

Site=18 Mod: C21[+57];
20210408_DIAserum_mix_PRM_A1_miss_select20.10834.10834.5.dta 5+ Δm=-2.18 ppm, -0.00 Th
5 5 1 1
LSLHRPALEDLLLGSEAJLTCTLTGLR
x1.1e+05
Relative Intensity
52%
42%
31%
21%
10%
y1 /1+
b2 /1+
y3 /1+
y4 /1+
b10 /2+
b5 /1+
b11 /2+
y6 /1+
b12 /2+
b13 /2+
b14 /2+
y7 /1+
b16 /2+
b17 /2+
y8 /1+
b18 /2+
b10 /1+
y11 /1+
y25 /2+
Y0 /2+
138.05
168.07
186.08
204.09
274.09
292.10
366.14
657.23
350.14
m/z
Δm (ppm)

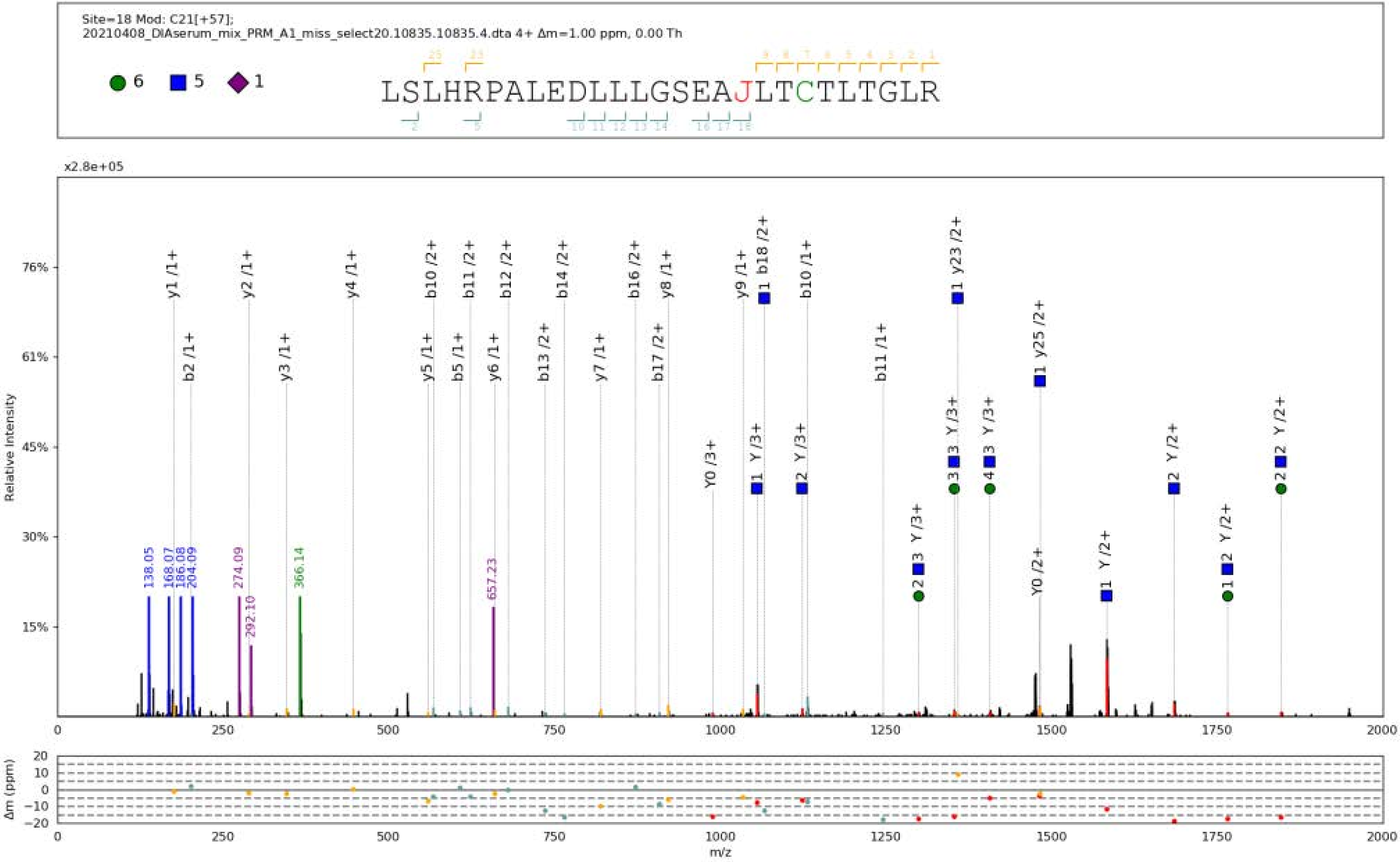

Site=18 Mod: C21[+57];
20210408_DIAserum_mix_PRM_A1_miss_select20.10835.10835.4.dta 4+ Δm=1.00 ppm, 0.00 Th
6
5
1
LSLHRPALEDLLLGSEAJLTCTLTGLR
x2.8e+05
Relative Intensity
76%
61%
45%
30%
15%
y1 /1+
b2 /1+
y2 /1+
y3 /1+
y4 /1+
y5 /1+
b10 /2+
b5 /1+
b11 /2+
y6 /1+
b12 /2+
b13 /2+
b14 /2+
y7 /1+
b16 /2+
b17 /2+
y8 /1+
Y0 /3+
y9 /1+
1 b18 /2+
1 Y /3+
2 Y /3+
b10 /1+
b11 /1+
2 3 Y /3+
3 3 Y /3+
1 y23 /2+
4 3 Y /3+
1 y25 /2+
Y0 /2+
1 Y /2+
2 Y /2+
1 2 Y /2+
2 2 Y /2+
138.05
168.07
186.08
204.09
274.09
292.10
366.14
657.23
0
250
500
750
1000
1250
1500
1750
2000
Δm (ppm)
20
10
0
-10
-20
m/z

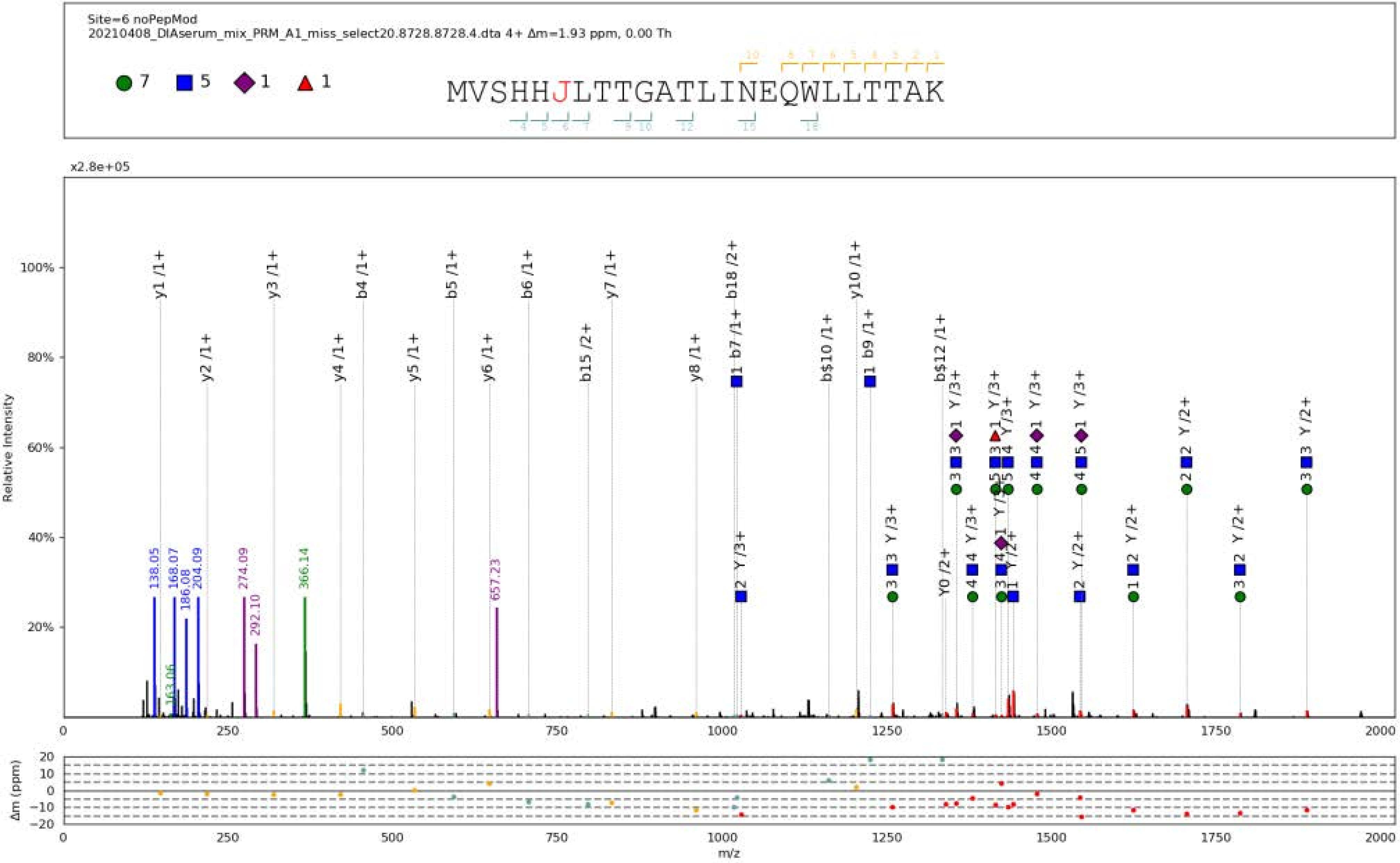

Site=6 noPepMod
20210408_DIAserum_mix_PRM_A1_miss_select20.8728.8728.4.dta 4+ Δm=1.93 ppm, 0.00 Th
7 5 1 1
MVSHHJLTTGATLINEQWLLTTAK
x2.8e+05
Relative Intensity
y1 /1+
y2 /1+
y3 /1+
y4 /1+
b4 /1+
y5 /1+
b5 /1+
y6 /1+
b6 /1+
b15 /2+
y7 /1+
y8 /1+
b7 /1+
b18 /2+
b$10 /1+
y10 /1+
b9 /1+
b$12 /1+
Y0 /2+
138.05
168.07
186.08
204.09
163.06
274.09
292.10
366.14
657.23
m/z
Δm (ppm)

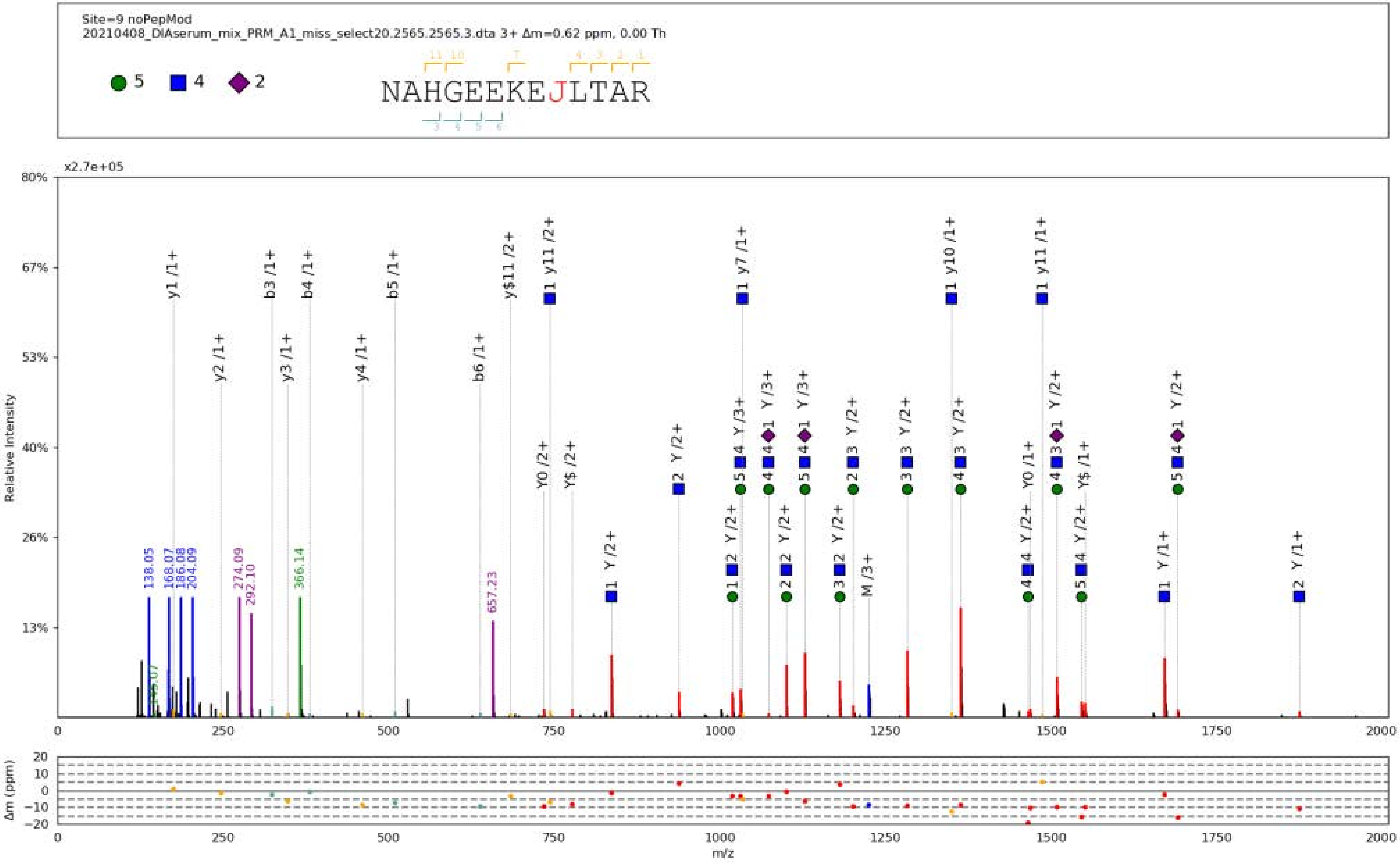

Site=9 noPepMod
20210408_DIAserum_mix_PRM_A1_miss_select20.2565.2565.3.dta 3+ Δm=0.62 ppm, 0.00 Th
NAHGEEKEJLTAR
x2.7e+05
Relative Intensity
Δm (ppm)
m/z

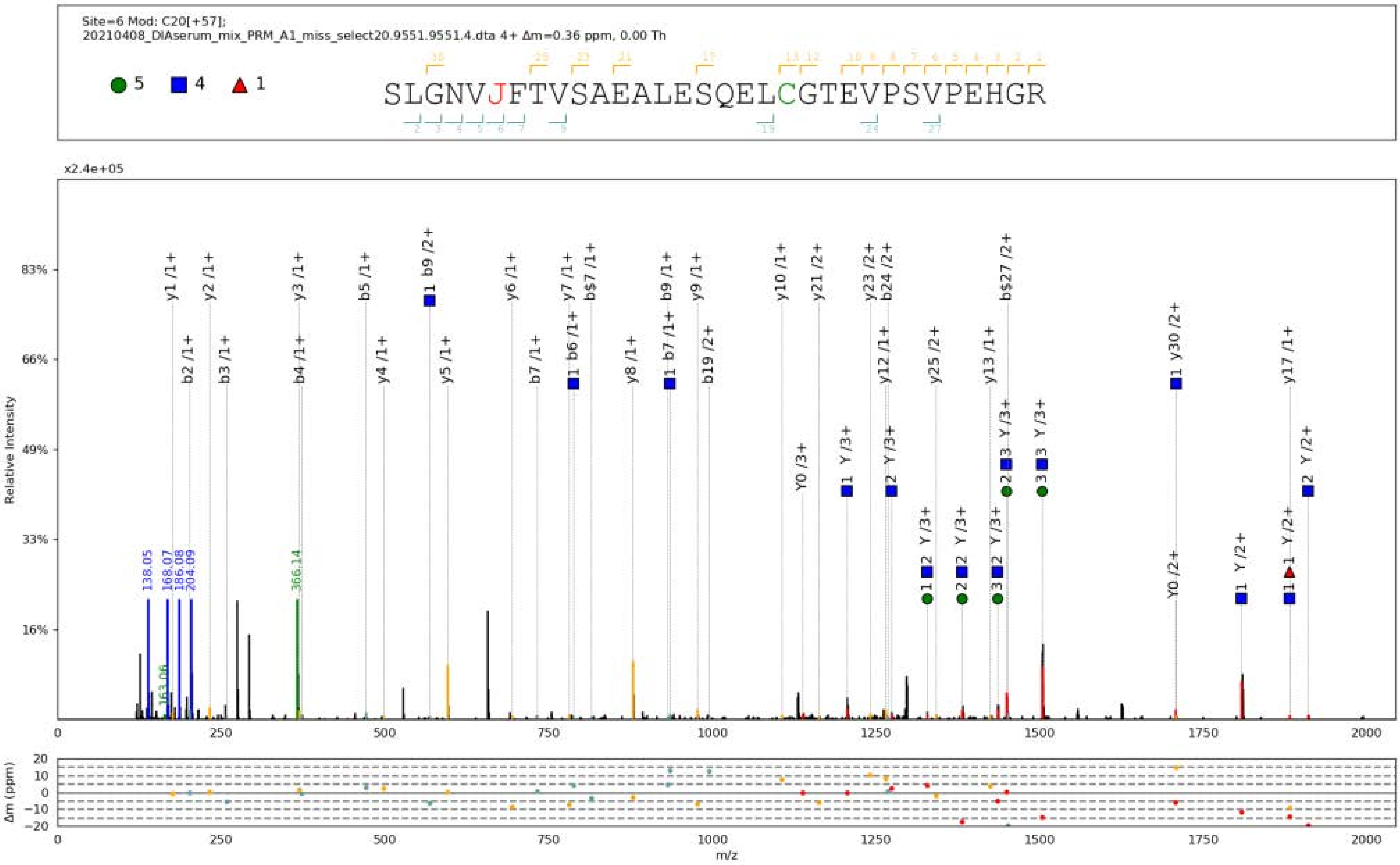

Site=6 Mod: C20[+57];
20210408_DIAserum_mix_PRM_A1_miss_select20.9551.9551.4.dta 4+ Δm=0.36 ppm, 0.00 Th
5
4
1
SLGNVJFTVSAEALESQELCGTEVPSVPEHGR
x2.4e+05
Relative Intensity
83%
66%
49%
33%
16%
138.05
168.07
186.08
204.09
163.06
366.14
y1 /1+
b2 /1+
y2 /1+
b3 /1+
y3 /1+
b4 /1+
b5 /1+
y4 /1+
b9 /2+
y5 /1+
y6 /1+
b7 /1+
y7 /1+
b6 /1+
b$7 /1+
y8 /1+
b9 /1+
b7 /1+
y9 /1+
b19 /2+
y10 /1+
Y0 /3+
y21 /2+
y23 /2+
b24 /2+
y12 /1+
y25 /2+
y13 /1+
b$27 /2+
y30 /2+
Y0 /2+
y17 /1+
m/z
Δm (ppm)

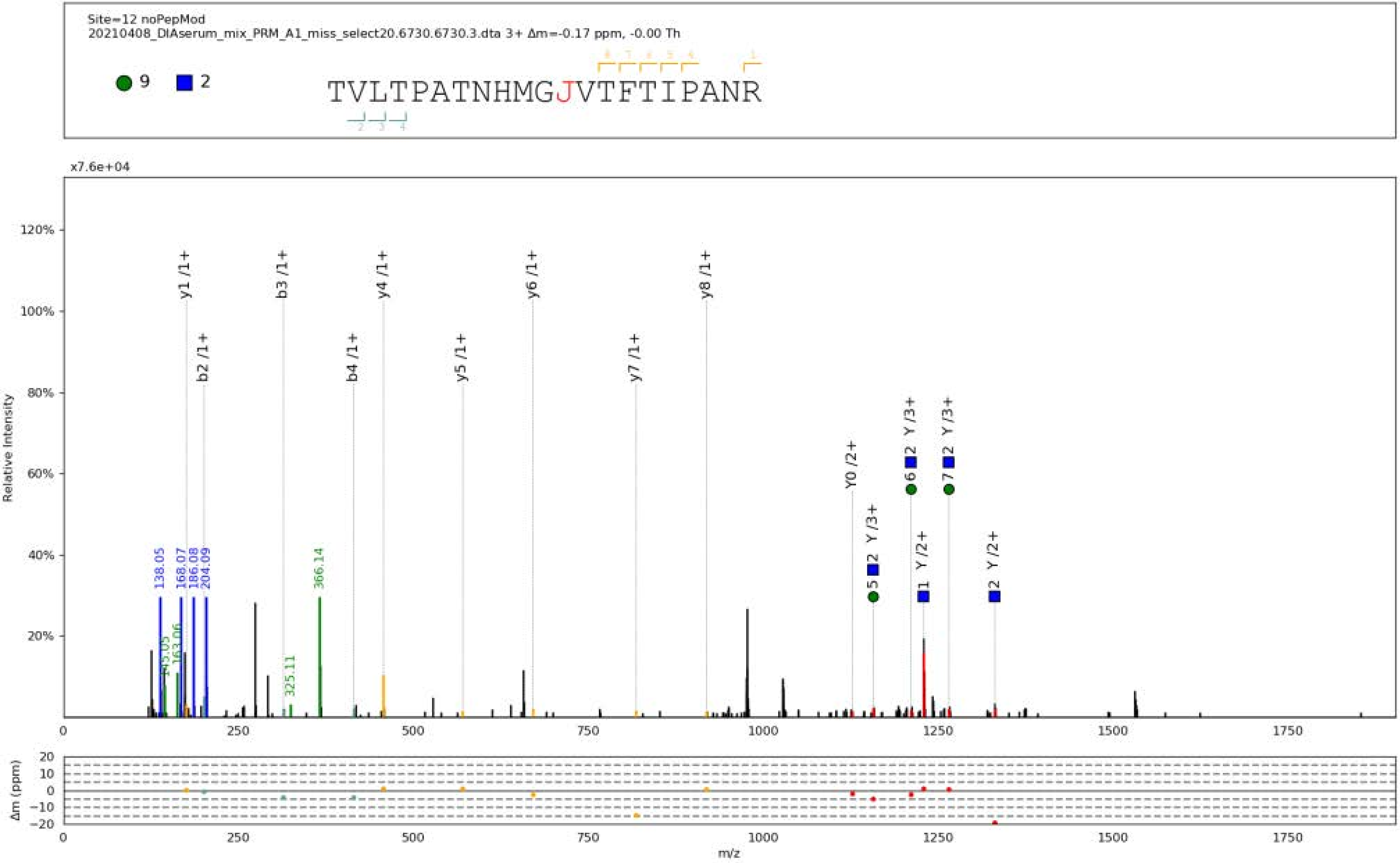

Site=12 noPepMod
20210408_DIAserum_mix_PRM_A1_miss_select20.6730.6730.3.dta 3+ Δm=-0.17 ppm, -0.00 Th
9
2
TVLTPATNHMGJVTFTIPANR
x7.6e+04
Relative Intensity
y1 /1+
b2 /1+
b3 /1+
b4 /1+
y4 /1+
y5 /1+
y6 /1+
y7 /1+
y8 /1+
Y0 /2+
138.05
168.07
186.08
204.09
366.14
145.05
163.06
325.11
m/z
Δm (ppm)

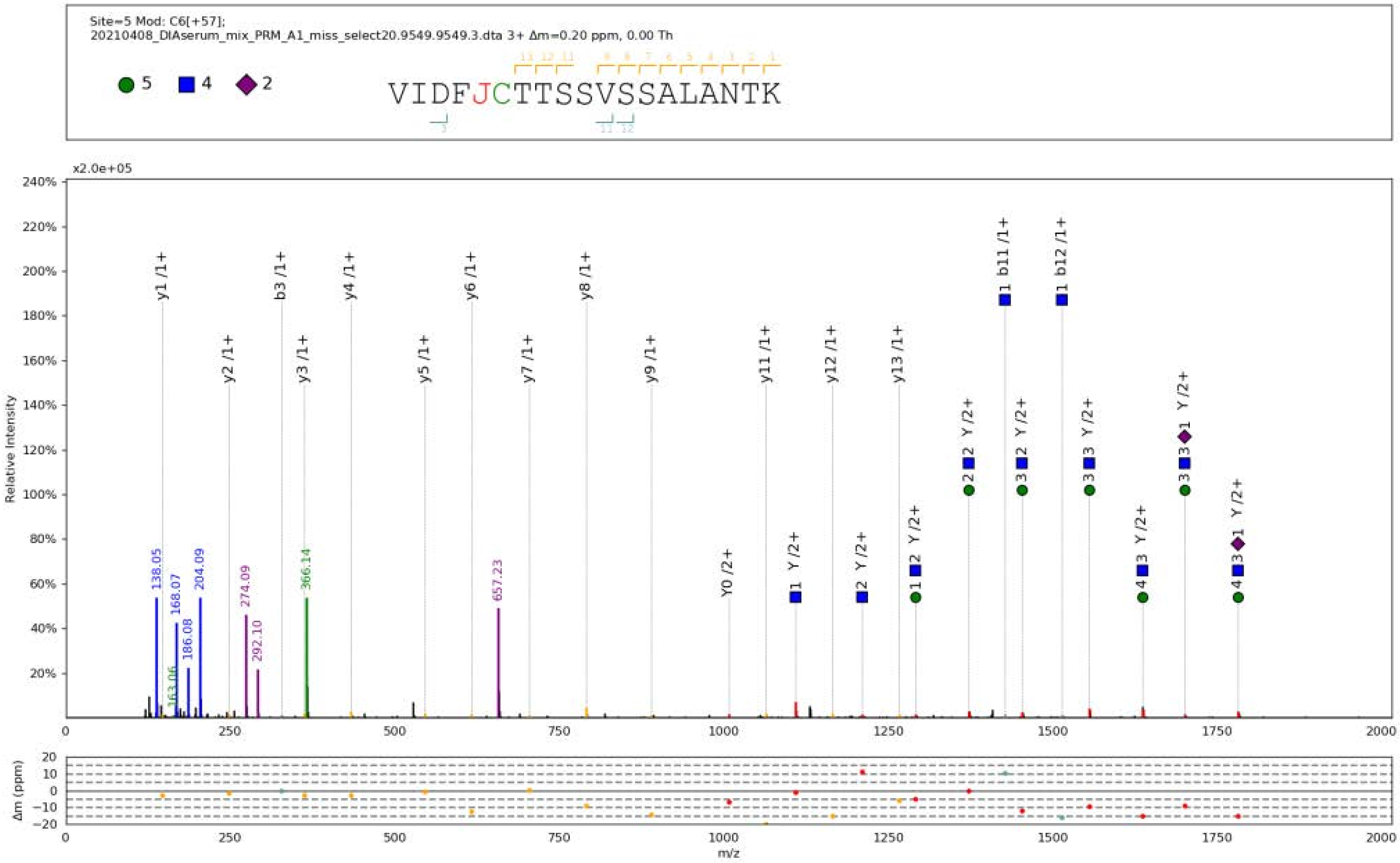
Site=5 Mod: C6[+57];
20210408_DIAserum_mix_PRM_A1_miss_select20.9549.9549.3.dta 3+ Δm=0.20 ppm, 0.00 Th
5
4
2
VIDFJCTTSSVSSALANTK
x2.0e+05
Relative Intensity
y1 /1+
b3 /1+
y4 /1+
y6 /1+
y8 /1+
y2 /1+
y3 /1+
y5 /1+
y7 /1+
y9 /1+
y11 /1+
y12 /1+
y13 /1+
1 b11 /1+
1 b12 /1+
Y0 /2+
138.05
168.07
186.08
204.09
163.06
274.09
292.10
366.14
657.23
m/z
Δm (ppm)

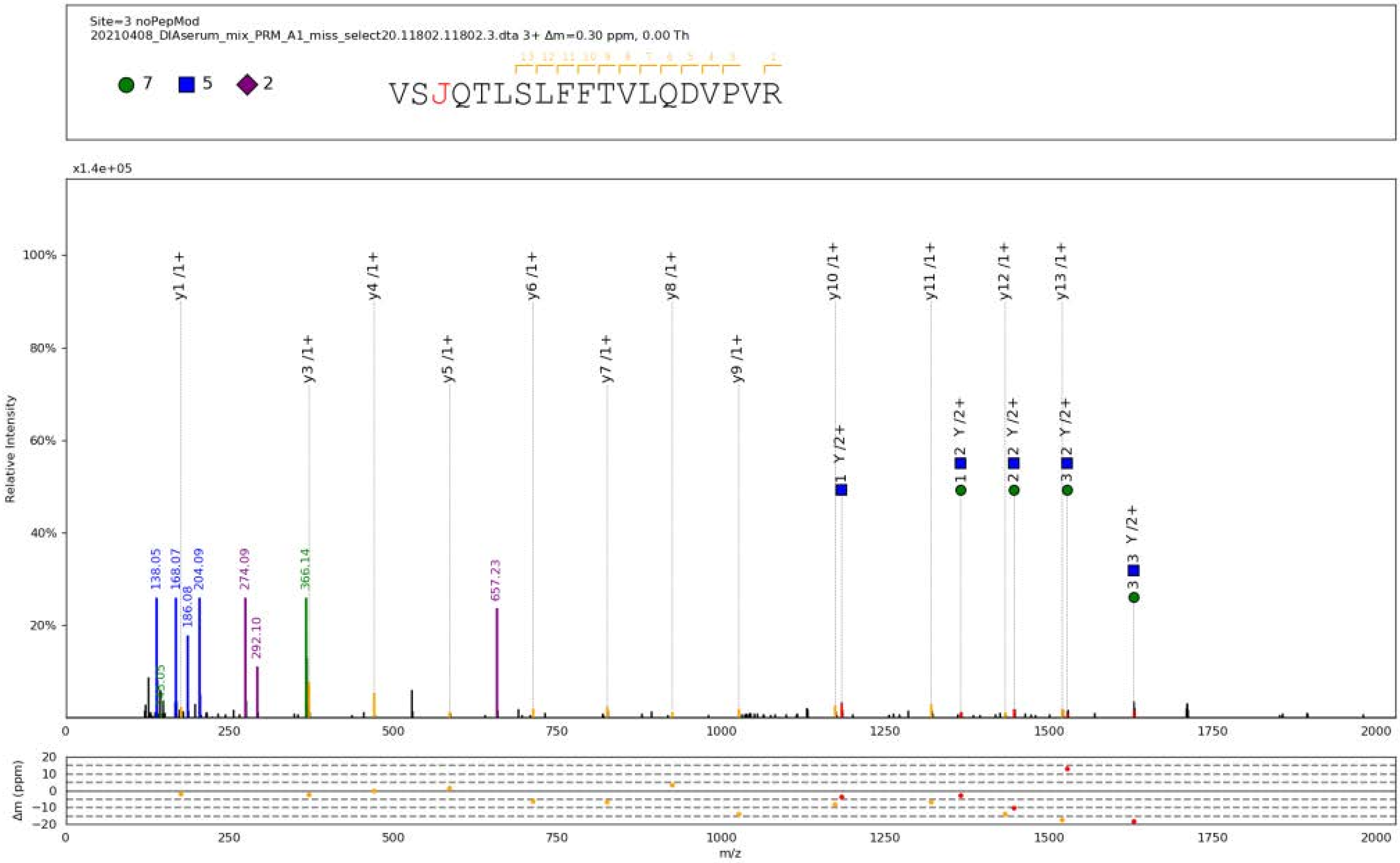
Site=3 noPepMod
20210408_DIAserum_mix_PRM_A1_miss_select20.11802.11802.3.dta 3+ Δm=0.30 ppm, 0.00 Th
7 5 2
VSJQTLSLFFTVLQDVPVR
x1.4e+05
Relative Intensity
y1 /1+
y3 /1+
y4 /1+
y5 /1+
y6 /1+
y7 /1+
y8 /1+
y9 /1+
y10 /1+
y11 /1+
y12 /1+
y13 /1+
138.05
168.07
186.08
204.09
274.09
292.10
366.14
657.23
Δm (ppm)
m/z

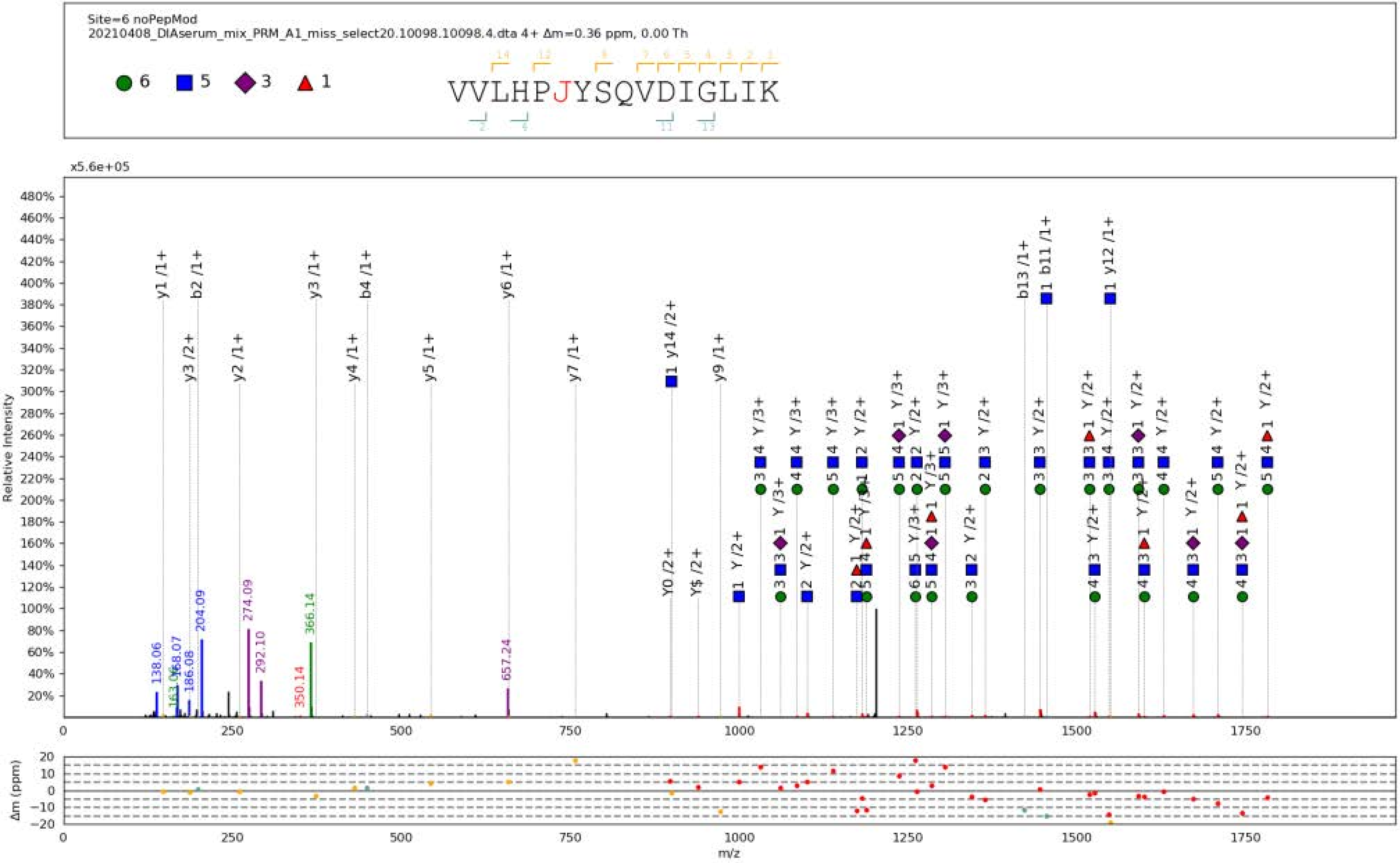
Site=6 noPepMod
20210408_DIAserum_mix_PRM_A1_miss_select20.10098.10098.4.dta 4+ Δm=0.36 ppm, 0.00 Th
6
5
3
1
VVLHPJYSQVDIGLIK
x5.6e+05
Relative Intensity
y1 /1+
b2 /1+
y3 /2+
y2 /1+
y3 /1+
y4 /1+
b4 /1+
y5 /1+
y6 /1+
y7 /1+
y14 /2+
y9 /1+
b13 /1+
b11 /1+
y12 /1+
Y0 /2+
Y$ /2+
138.06
163.06
168.07
186.08
204.09
274.09
292.10
350.14
366.14
657.24
m/z
Δm (ppm)

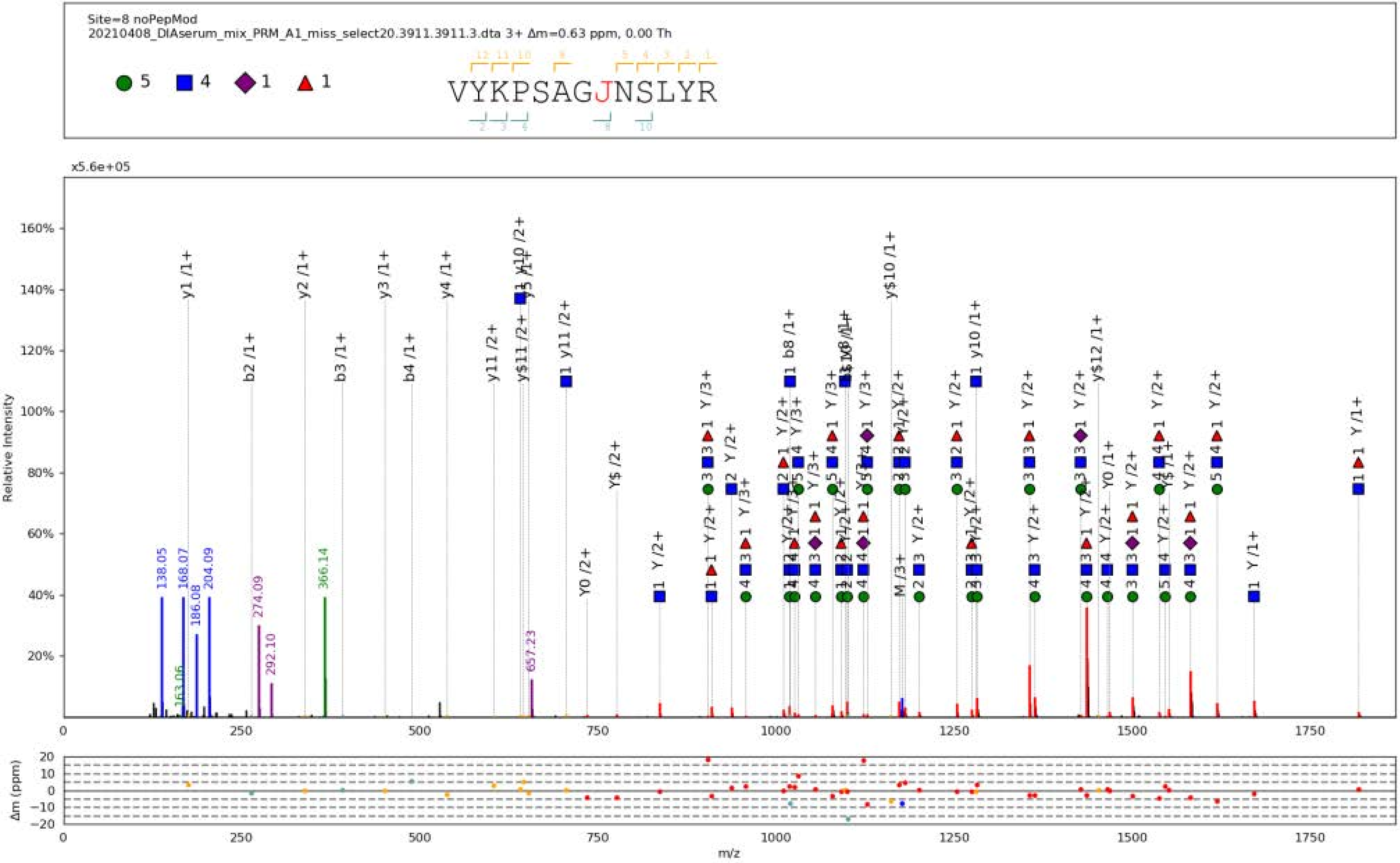
Site=8 noPepMod
20210408_DIAserum_mix_PRM_A1_miss_select20.3911.3911.3.dta 3+ Δm=0.63 ppm, 0.00 Th
VYKPSAGJNSLYR
x5.6e+05
Relative Intensity
y1 /1+
y2 /1+
y3 /1+
y4 /1+
b2 /1+
b3 /1+
b4 /1+
y11 /2+
y$11 /2+
y$10 /1+
y10 /1+
y$12 /1+
b8 /1+
Y0 /2+
Y0 /1+
Y$ /2+
M /3+
138.05
168.07
186.08
204.09
163.06
274.09
292.10
366.14
657.23
m/z
Δm (ppm)

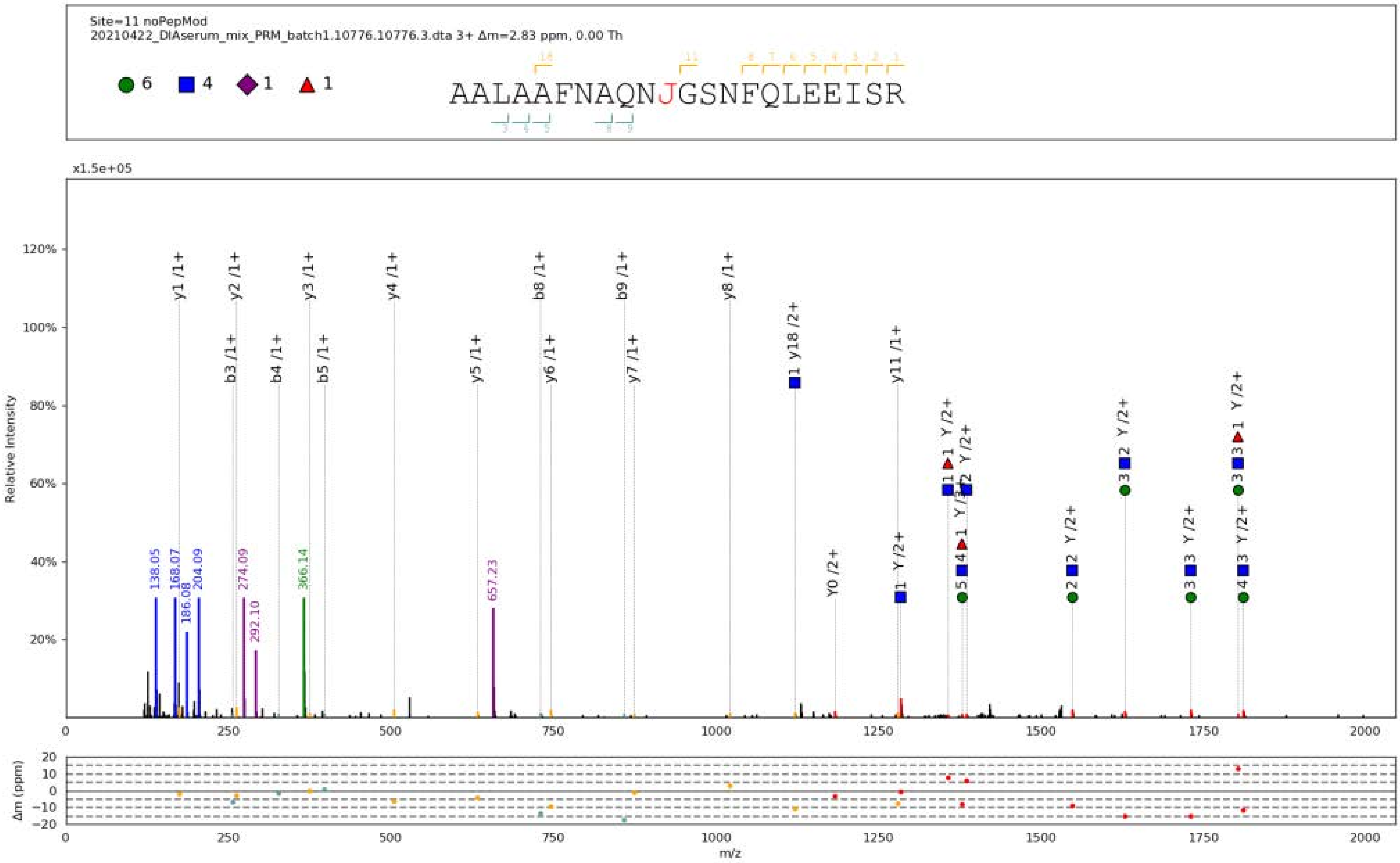

Site=11 noPepMod
20210422_DIAserum_mix_PRM_batch1.10776.10776.3.dta 3+ Δm=2.83 ppm, 0.00 Th
6 4 1 1
AALAAFNAQNJGSNFQLEEISR
x1.5e+05
Relative Intensity
y1 /1+
y2 /1+
b3 /1+
b4 /1+
y3 /1+
b5 /1+
y4 /1+
y5 /1+
b8 /1+
y6 /1+
b9 /1+
y7 /1+
y8 /1+
y18 /2+
Y0 /2+
y11 /1+
Y /2+
138.05
168.07
186.08
204.09
274.09
292.10
366.14
657.23
m/z
Δm (ppm)

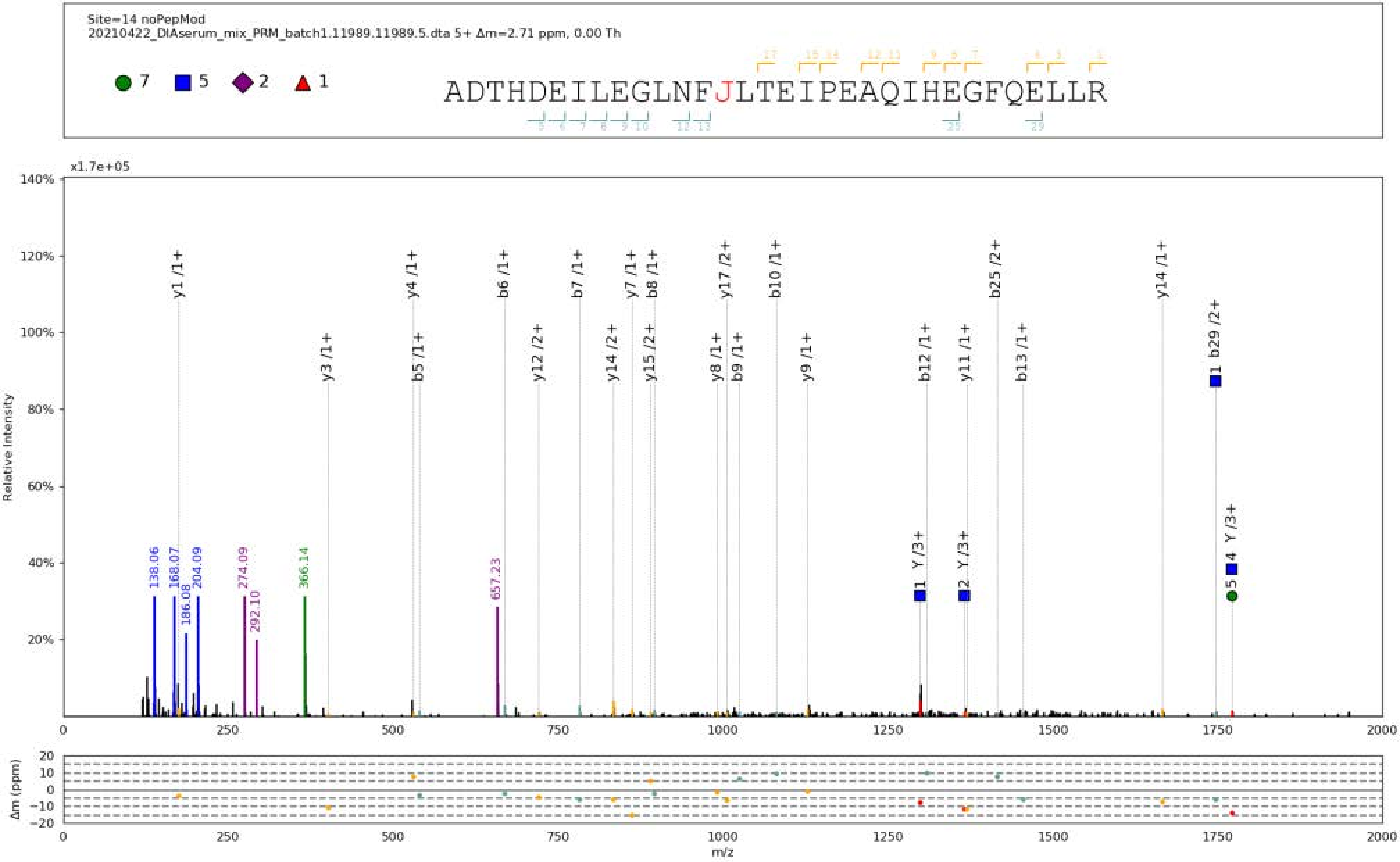
Site=14 noPepMod
20210422_DIAserum_mix_PRM_batch1.11989.11989.5.dta 5+ Δm=2.71 ppm, 0.00 Th
7
5
2
1
ADTHDEILEGLNFJLTEIPEAQIHEGFQELLR
x1.7e+05
Relative Intensity
m/z
Δm (ppm)
y1 /1+
y3 /1+
y4 /1+
b5 /1+
b6 /1+
y12 /2+
b7 /1+
y14 /2+
y7 /1+
y15 /2+
b8 /1+
y8 /1+
y17 /2+
b9 /1+
b10 /1+
y9 /1+
b12 /1+
y11 /1+
b25 /2+
b13 /1+
y14 /1+
b29 /2+
1 Y /3+
2 Y /3+
5 4 Y /3+
138.06
168.07
186.08
204.09
274.09
292.10
366.14
657.23

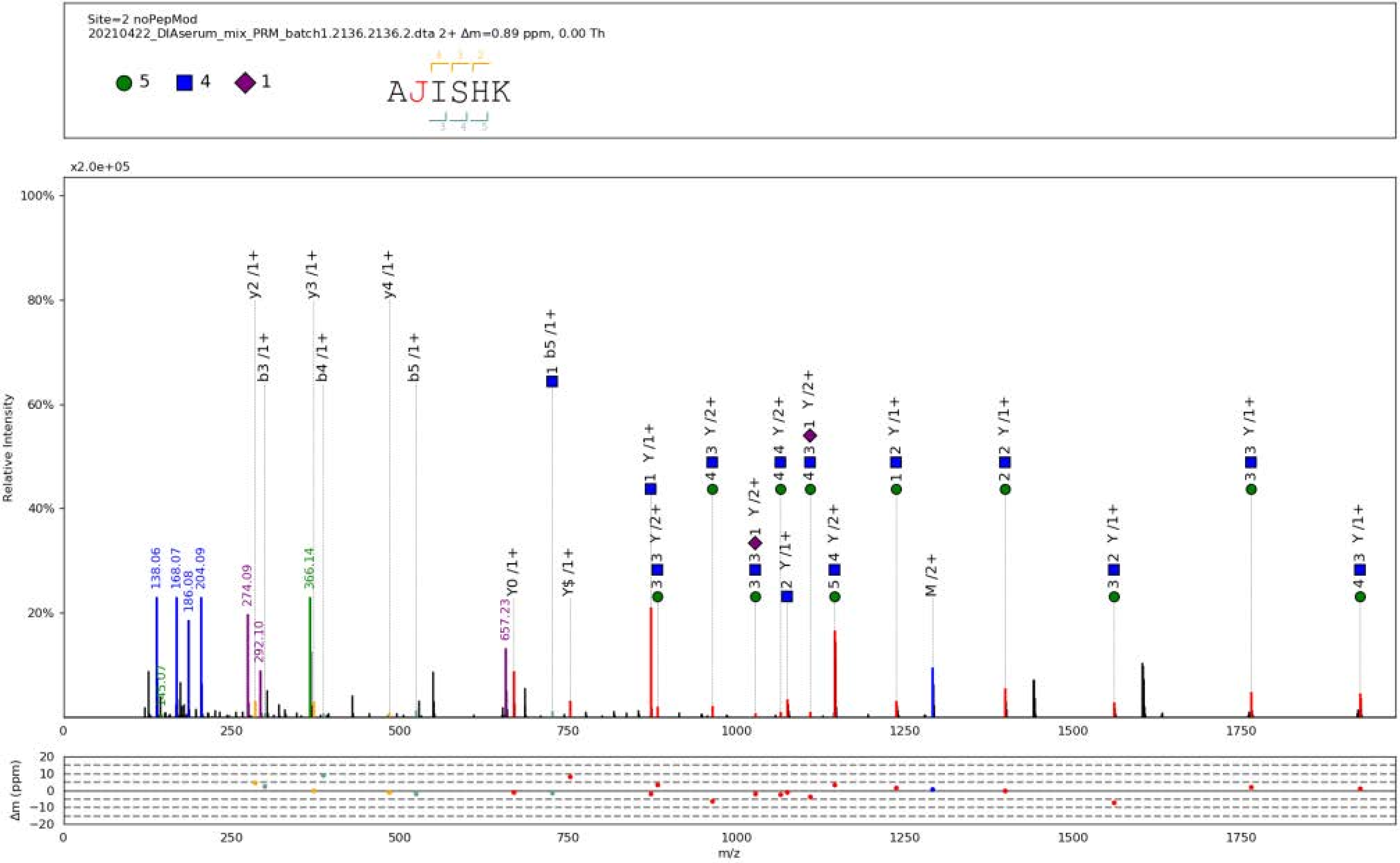

Site=2 noPepMod
20210422_DIAserum_mix_PRM_batch1.2136.2136.2.dta 2+ Δm=0.89 ppm, 0.00 Th
5
4
1
AJISHK
x2.0e+05
Relative Intensity
m/z
Δm (ppm)
y2 /1+
b3 /1+
y3 /1+
b4 /1+
y4 /1+
b5 /1+
1 b5 /1+
Y0 /1+
Y$ /1+
M /2+
138.06
168.07
186.08
204.09
145.07
274.09
292.10
366.14
657.23

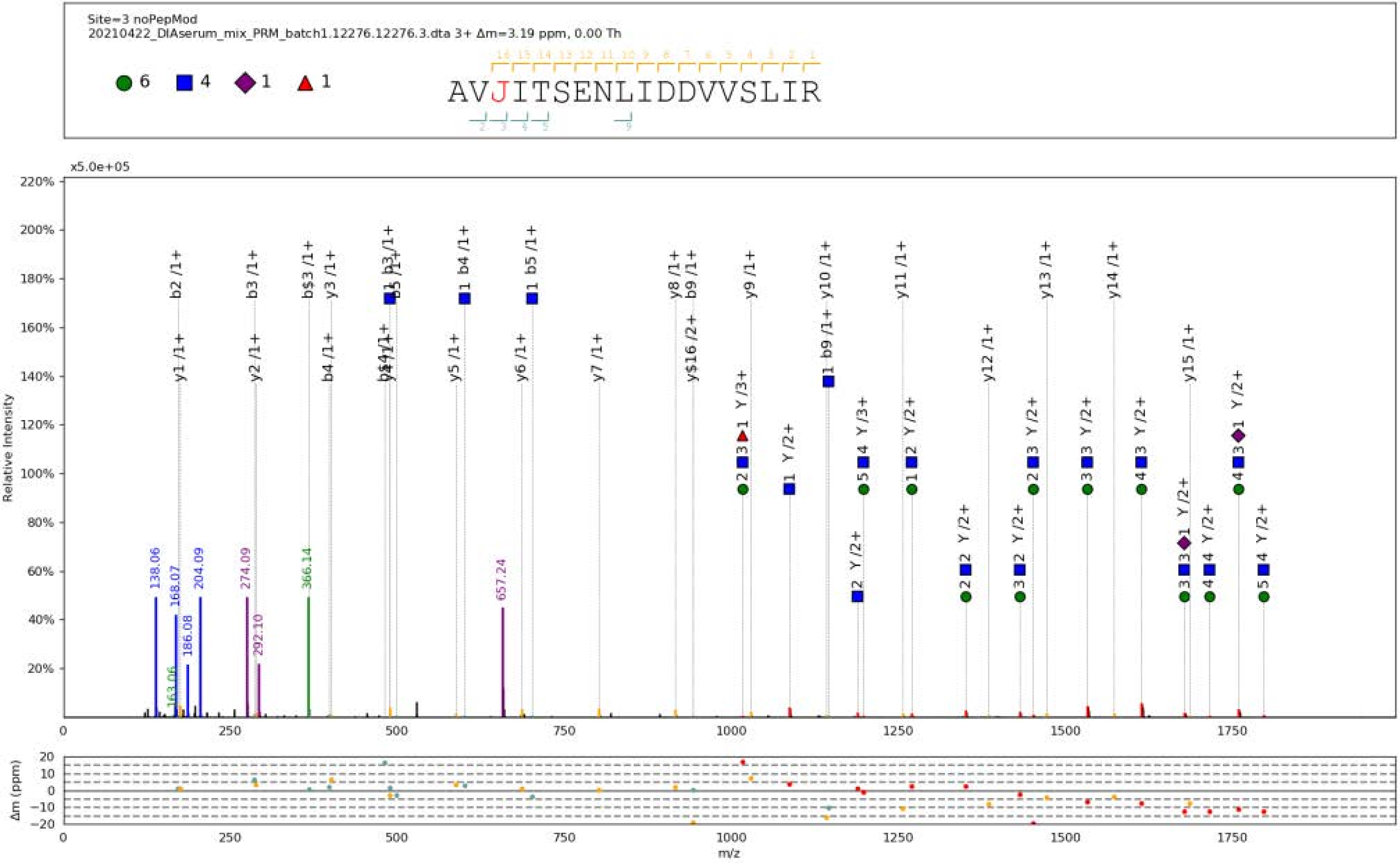
Site=3 noPepMod
20210422_DIAserum_mix_PRM_batch1.12276.12276.3.dta 3+ Δm=3.19 ppm, 0.00 Th
6
4
1
1
AVJITSENLIDDVVSLIR
x5.0e+05
Relative Intensity
b2 /1+
y1 /1+
b3 /1+
y2 /1+
b$3 /1+
y3 /1+
b4 /1+
b1 b3 /1+
b5 /1+
y4 /1+
b4 /1+
1 b4 /1+
y5 /1+
1 b5 /1+
y6 /1+
y7 /1+
y8 /1+
b9 /1+
y$16 /2+
y9 /1+
2 3 1 Y /3+
1 Y /2+
y10 /1+
1 b9 /1+
2 Y /2+
5 4 Y /3+
y11 /1+
1 2 Y /2+
2 2 Y /2+
y12 /1+
3 2 Y /2+
2 3 Y /2+
y13 /1+
3 3 Y /2+
y14 /1+
4 3 Y /2+
3 3 1 Y /2+
y15 /1+
4 4 Y /2+
4 3 1 Y /2+
5 4 Y /2+
138.06
163.06
168.07
186.08
204.09
274.09
292.10
366.14
657.24
Δm (ppm)
m/z

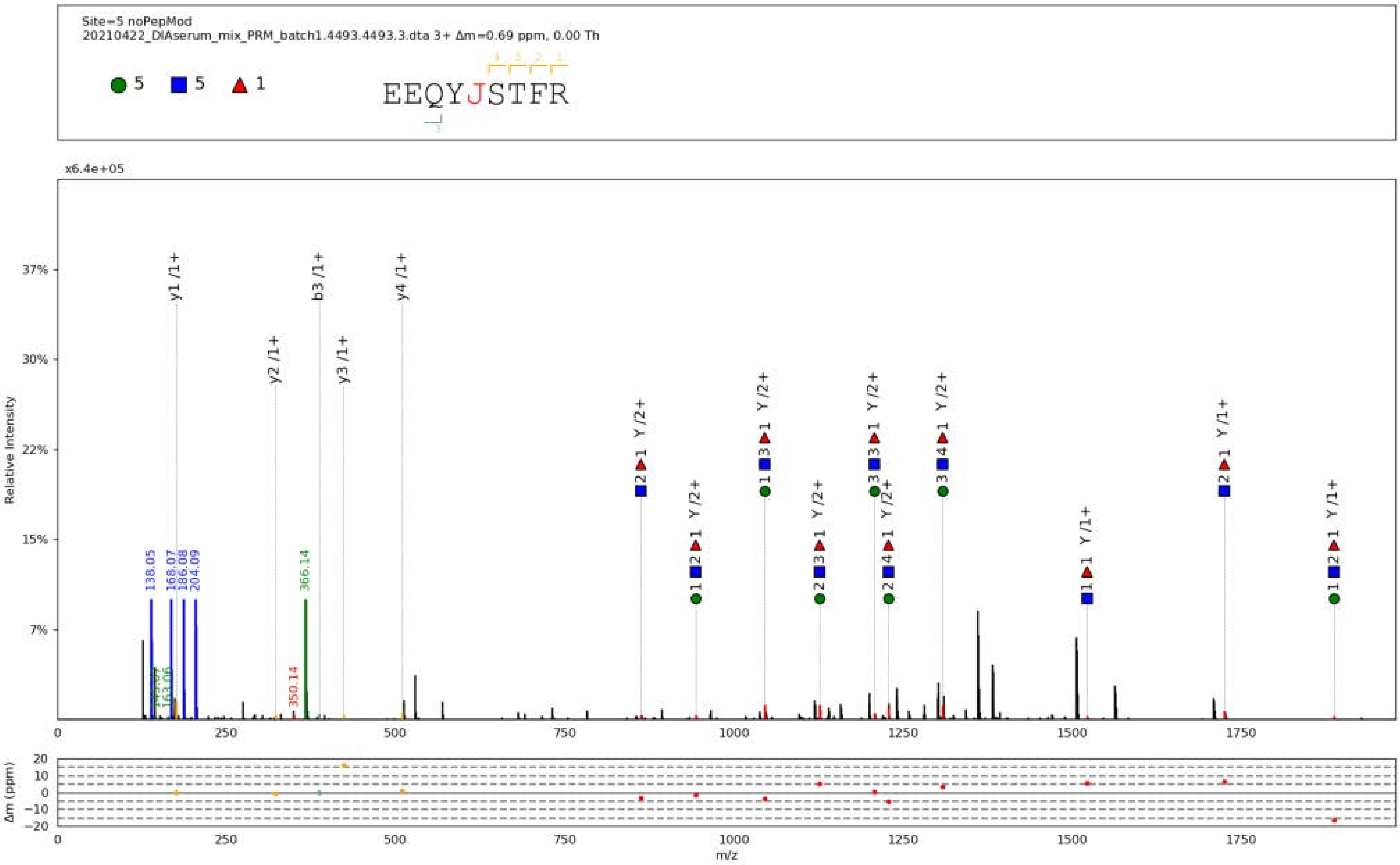
Site=5 noPepMod
20210422_DIAserum_mix_PRM_batch1.4493.4493.3.dta 3+ Δm=0.69 ppm, 0.00 Th
EEQYJSTFR
x6.4e+05
Relative Intensity
m/z
Δm (ppm)

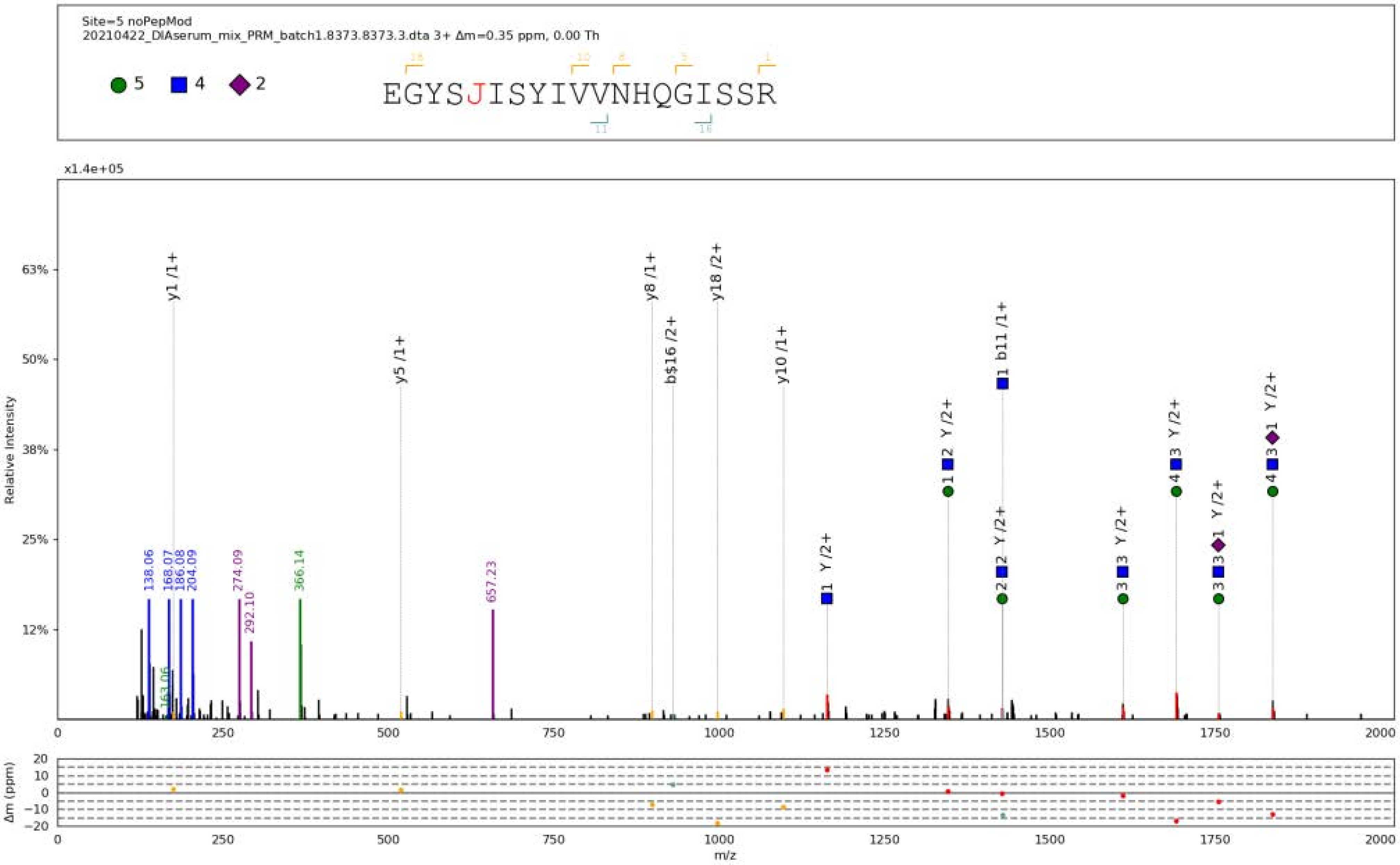
Site=5 noPepMod
20210422_DIAserum_mix_PRM_batch1.8373.8373.3.dta 3+ Δm=0.35 ppm, 0.00 Th
EGYSJISYIVVNHQGISSR
x1.4e+05
Relative Intensity
y1 /1+
y8 /1+
y18 /2+
b$16 /2+
y5 /1+
y10 /1+
b11 /1+
138.06
168.07
186.08
204.09
274.09
292.10
366.14
657.23
163.06
Δm (ppm)
m/z

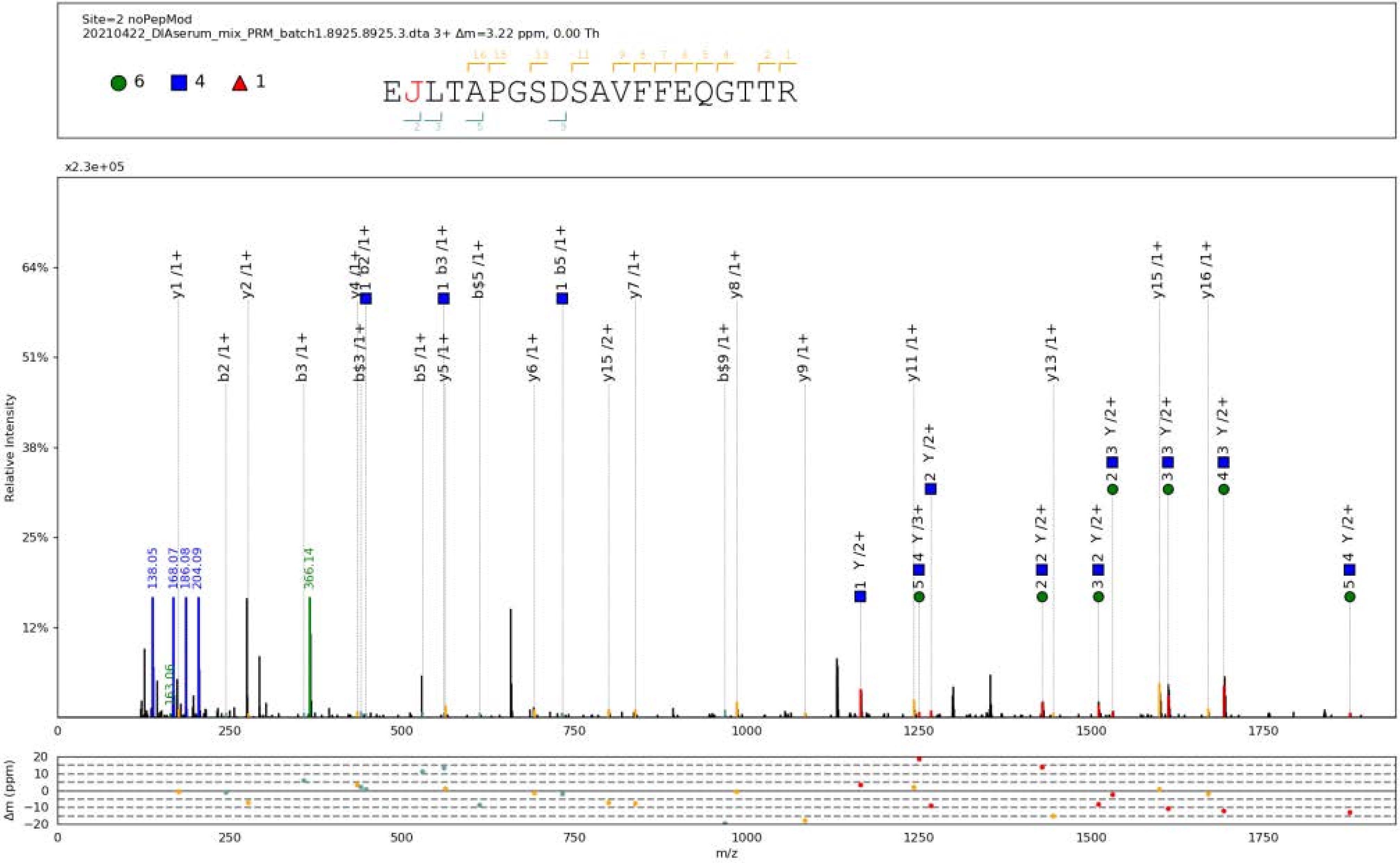

Site=2 noPepMod
20210422_DIAserum_mix_PRM_batch1.8925.8925.3.dta 3+ Δm=3.22 ppm, 0.00 Th
6
4
1
EJLTAPGSDSAVFFEQGTTR
x2.3e+05
Relative Intensity
64%
51%
38%
25%
12%
y1 /1+
y2 /1+
b2 /1+
b3 /1+
b$3 /1+
y4 /1+
b5 /1+
y5 /1+
b$5 /1+
y6 /1+
y15 /2+
y7 /1+
b$9 /1+
y8 /1+
y9 /1+
y11 /1+
y13 /1+
y15 /1+
y16 /1+
138.05
168.07
186.08
204.09
163.06
366.14
m/z
Δm (ppm)

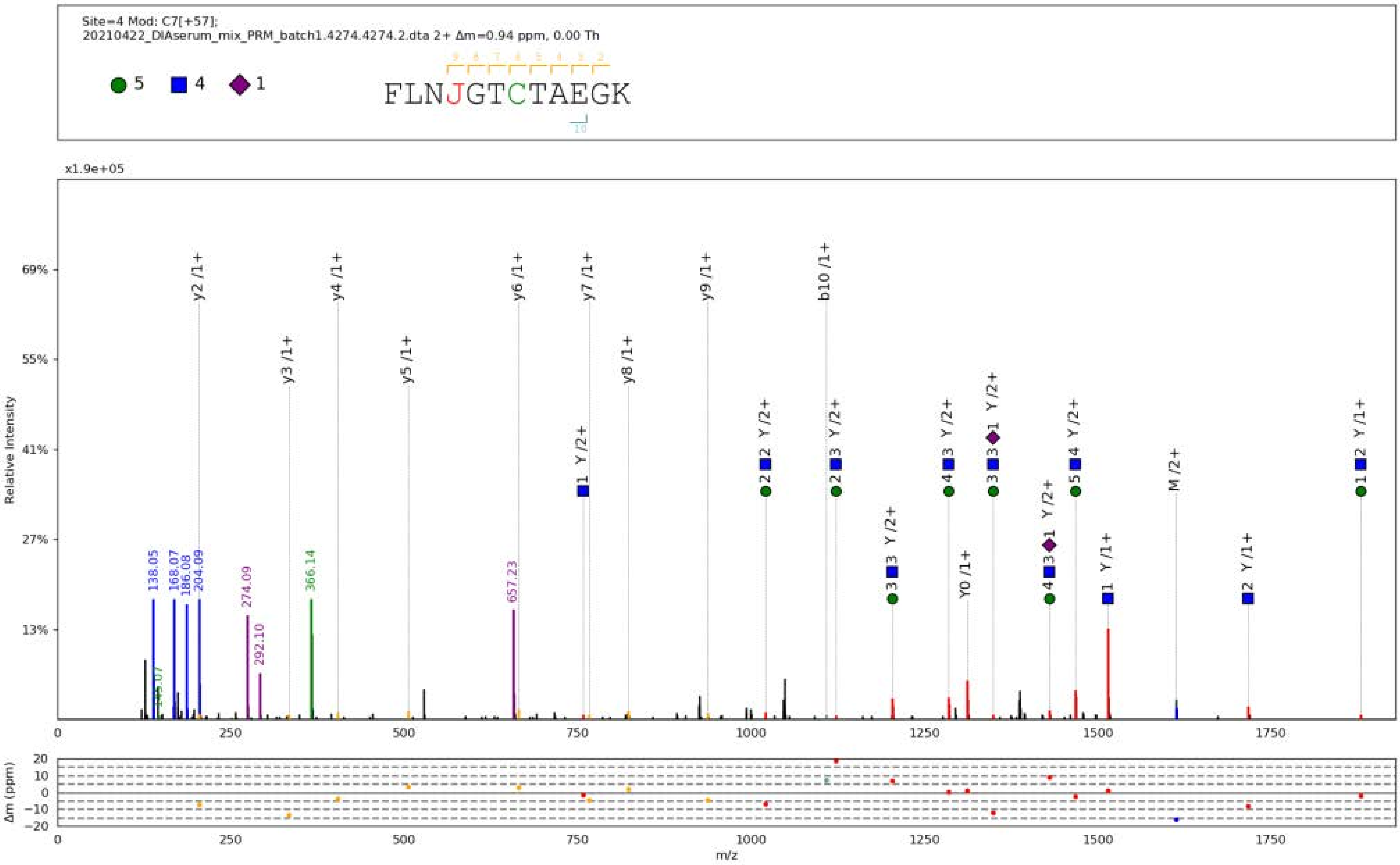

Site=4 Mod: C7[+57];
20210422_DIAserum_mix_PRM_batch1.4274.4274.2.dta 2+ Δm=0.94 ppm, 0.00 Th
FLNJGTCTAEGK
x1.9e+05
Relative Intensity
m/z
Δm (ppm)

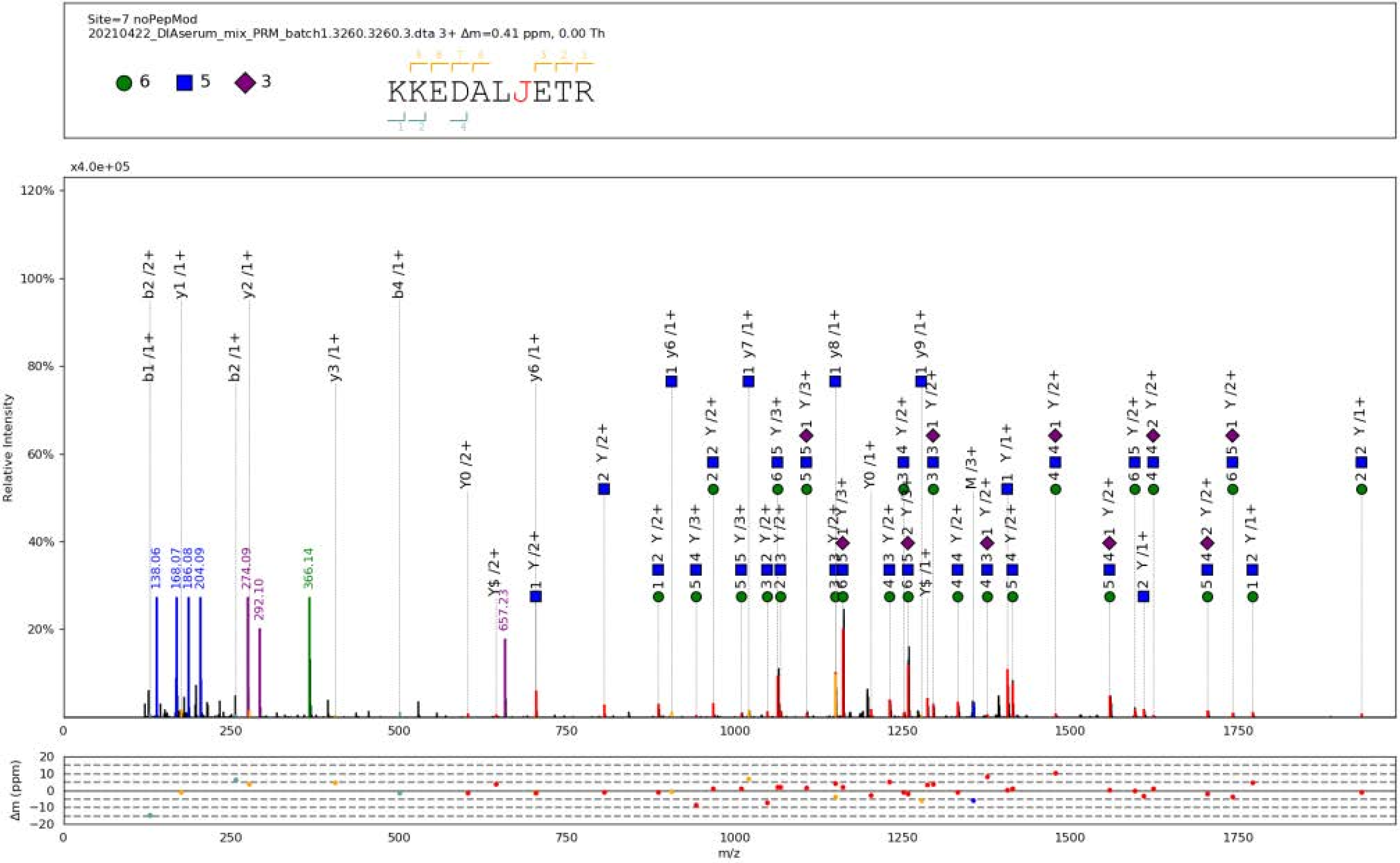
Site=7 noPepMod
20210422_DIAserum_mix_PRM_batch1.3260.3260.3 .dta 3+ Δm=0.41 ppm, 0.00 Th
6
5
3
KKEDALJETR
x4.0e+05
Relative Intensity
b1 /1+
b2 /2+
y1 /1+
b2 /1+
y2 /1+
y3 /1+
b4 /1+
y6 /1+
Y0 /2+
Y$ /2+
y6 /1+
y7 /1+
y8 /1+
y9 /1+
Y0 /1+
M /3+
Y$ /1+
138.06
168.07
186.08
204.09
274.09
292.10
366.14
657.23
m/z
Δm (ppm)

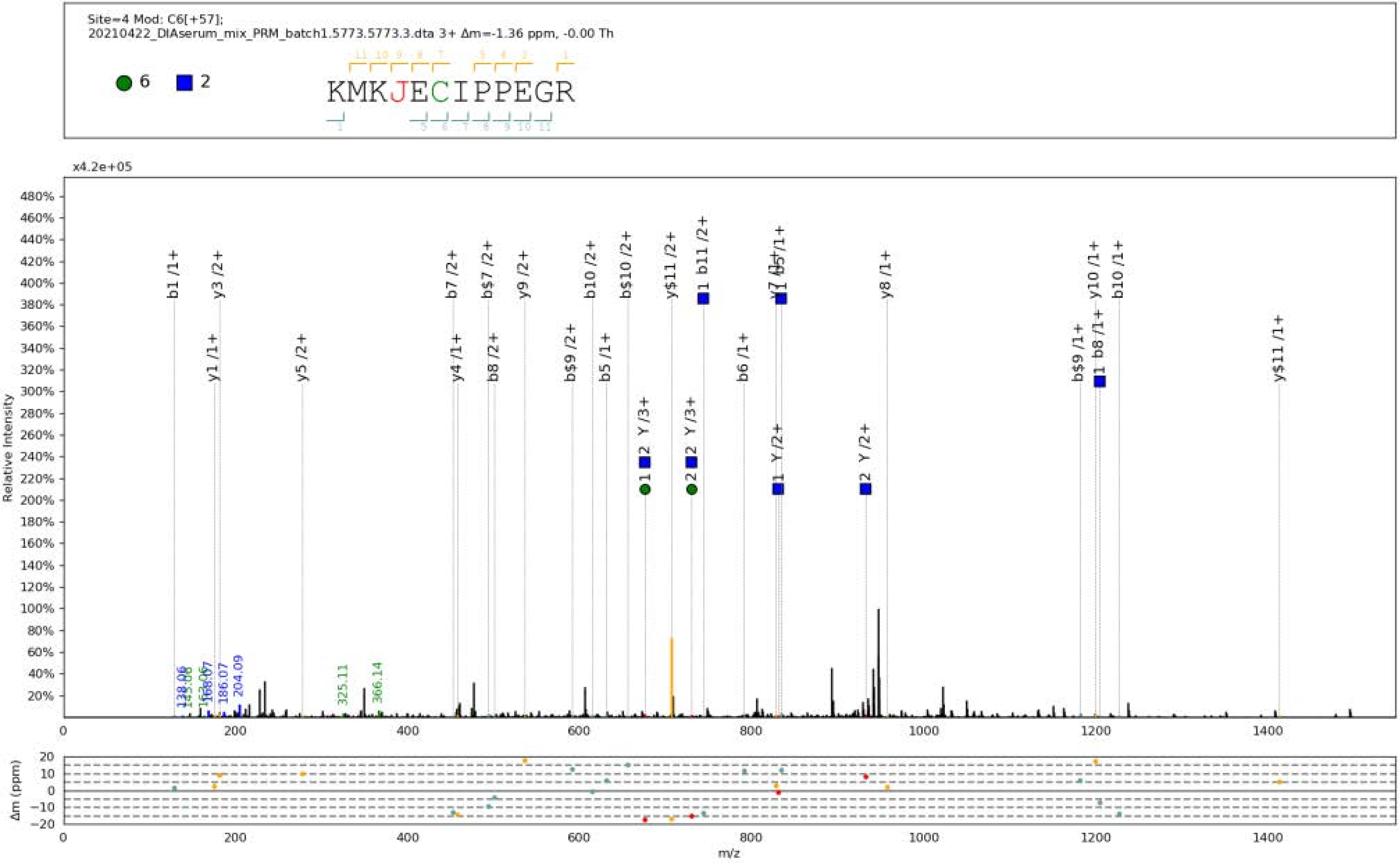
Site=4 Mod: C6[+57];
20210422_DIAserum_mix_PRM_batch1.5773.5773.3.dta 3+ Δm=-1.36 ppm, -0.00 Th
6
2
KMKJECIPPEGR
x4.2e+05
Relative Intensity
b1 /1+
y3 /2+
y1 /1+
y5 /2+
b7 /2+
b$7 /2+
y9 /2+
y4 /1+
b8 /2+
b$9 /2+
b10 /2+
b5 /1+
b$10 /2+
y$11 /2+
b11 /2+
b6 /1+
y7 /1+
b5 /1+
y8 /1+
Y /3+
Y /2+
b$9 /1+
b8 /1+
y10 /1+
b10 /1+
y$11 /1+
138.06
145.08
163.05
168.07
186.07
204.09
325.11
366.14
m/z
Δm (ppm)

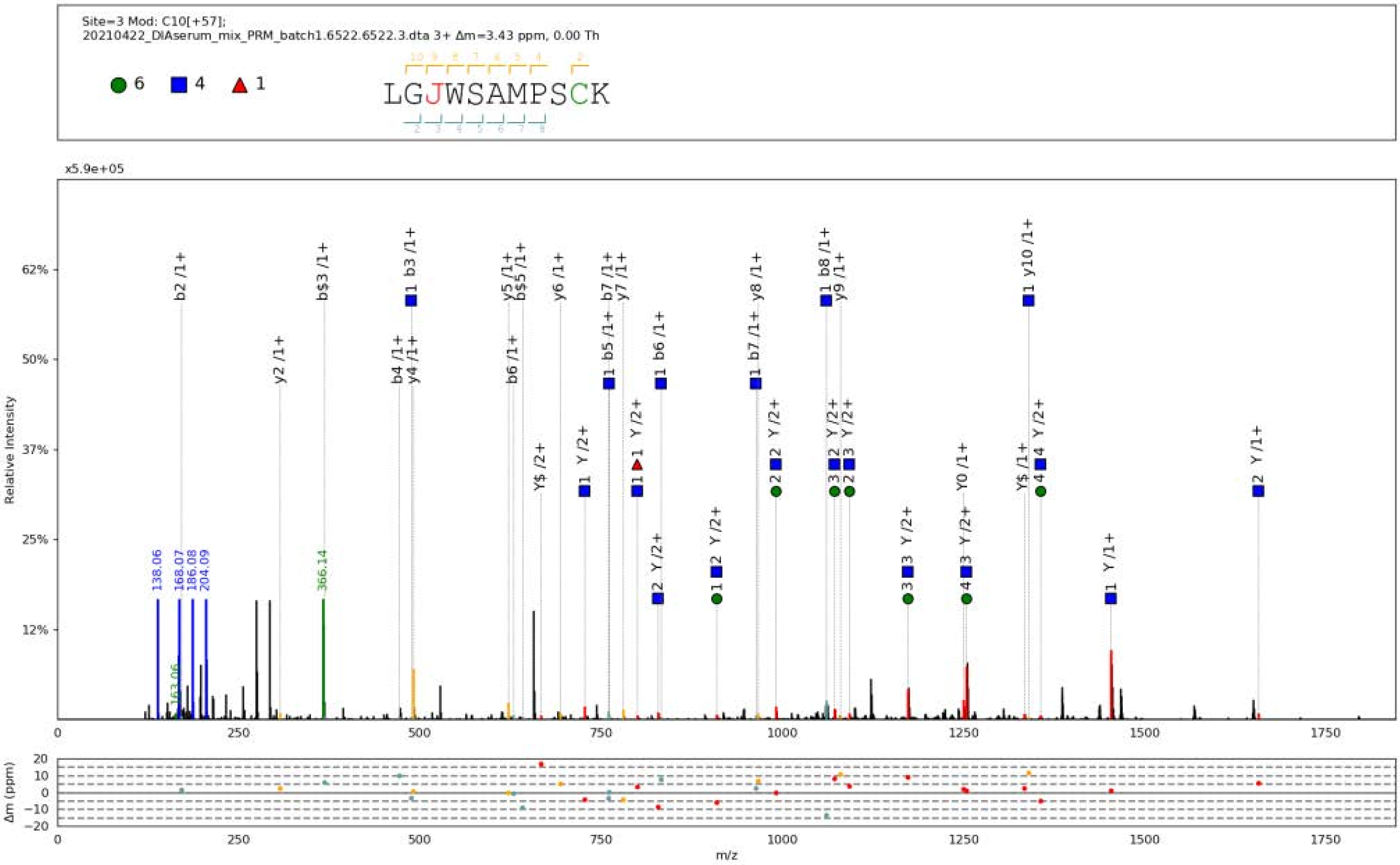
Site=3 Mod: C10[+57];
20210422_DIAserum_mix_PRM_batch1.6522.6522.3.dta 3+ Δm=3.43 ppm, 0.00 Th
LGJWSAMPSCK
x5.9e+05
Relative Intensity
m/z
Δm (ppm)

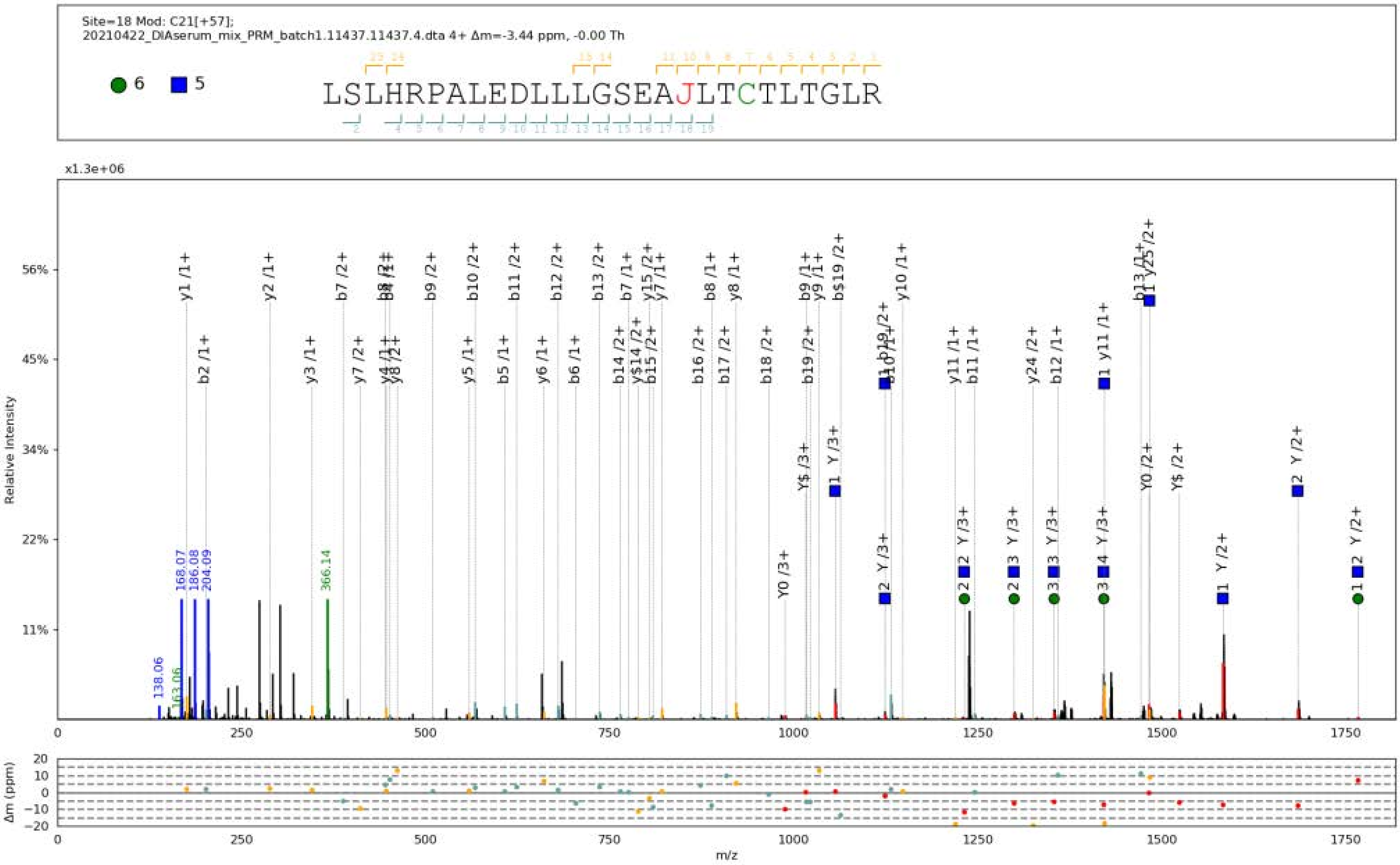
Site=18 Mod: C21[+57];
20210422_DIAserum_mix_PRM_batch1.11437.11437.4.dta 4+ Δm=-3.44 ppm, -0.00 Th
6
5
LSLHRPALEDLLLGSEAJLTCTLTGLR
x1.3e+06
Relative Intensity
m/z
Δm (ppm)
138.06
163.06
168.07
186.08
204.09
366.14

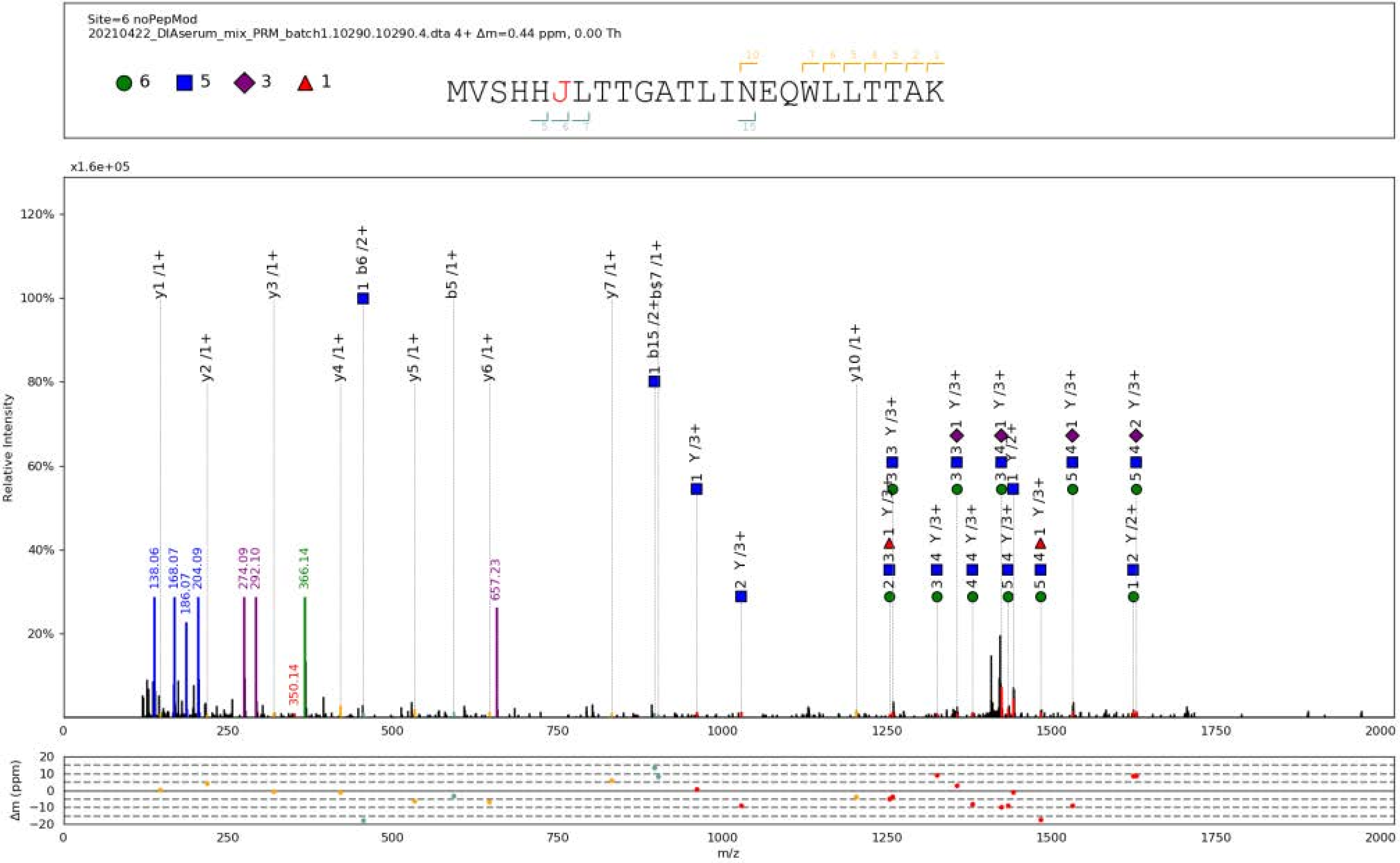

Site=6 noPepMod
20210422_DIAserum_mix_PRM_batch1.10290.10290.4.dta 4+ Δm=0.44 ppm, 0.00 Th
6
5
3
1
MVSHHJLTTGATLINEQWLLTTAK
x1.6e+05
Relative Intensity
Δm (ppm)
m/z
y1 /1+
y2 /1+
y3 /1+
y4 /1+
1 b6 /2+
y5 /1+
b5 /1+
y6 /1+
y7 /1+
1 b15 /2+b$7 /1+
y10 /1+
138.06
168.07
186.07
204.09
274.09
292.10
366.14
350.14
657.23

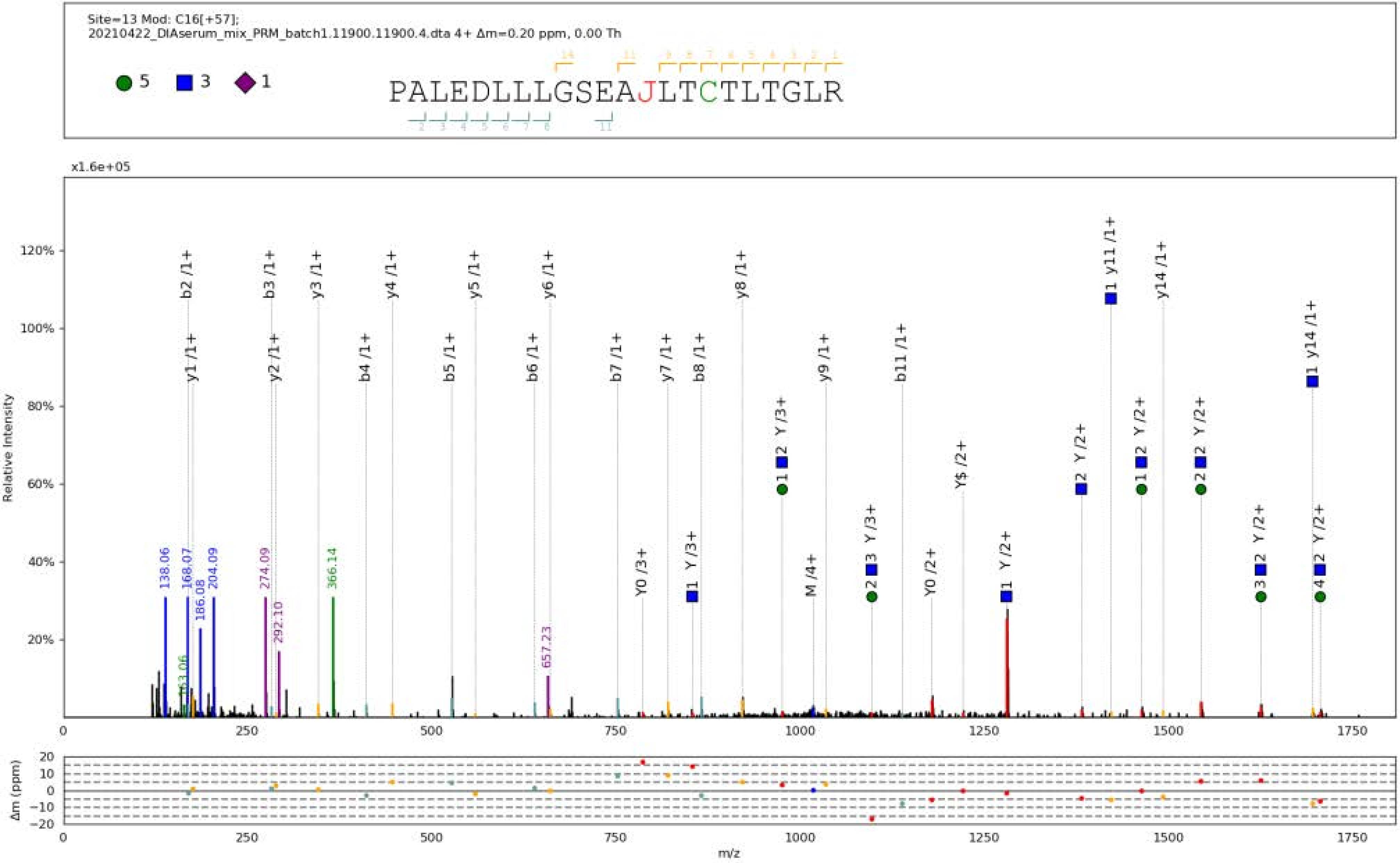

Site=13 Mod: C16[+57];
20210422_DIAserum_mix_PRM_batch1.11900.11900.4.dta 4+ Δm=0.20 ppm, 0.00 Th
5
3
1
PALEDLLLGSEAJLTCTLTGLR
x1.6e+05
Relative Intensity
b2 /1+
y1 /1+
b3 /1+
y2 /1+
y3 /1+
b4 /1+
y4 /1+
b5 /1+
y5 /1+
b6 /1+
y6 /1+
b7 /1+
Y0 /3+
y7 /1+
1 Y /3+
b8 /1+
y8 /1+
1 2 Y /3+
M /4+
y9 /1+
2 3 Y /3+
b11 /1+
Y0 /2+
Y$ /2+
1 Y /2+
2 Y /2+
1 y11 /1+
1 2 Y /2+
y14 /1+
2 2 Y /2+
3 2 Y /2+
1 y14 /1+
4 2 Y /2+
138.06
168.07
186.08
204.09
274.09
292.10
366.14
657.23
m/z
Δm (ppm)

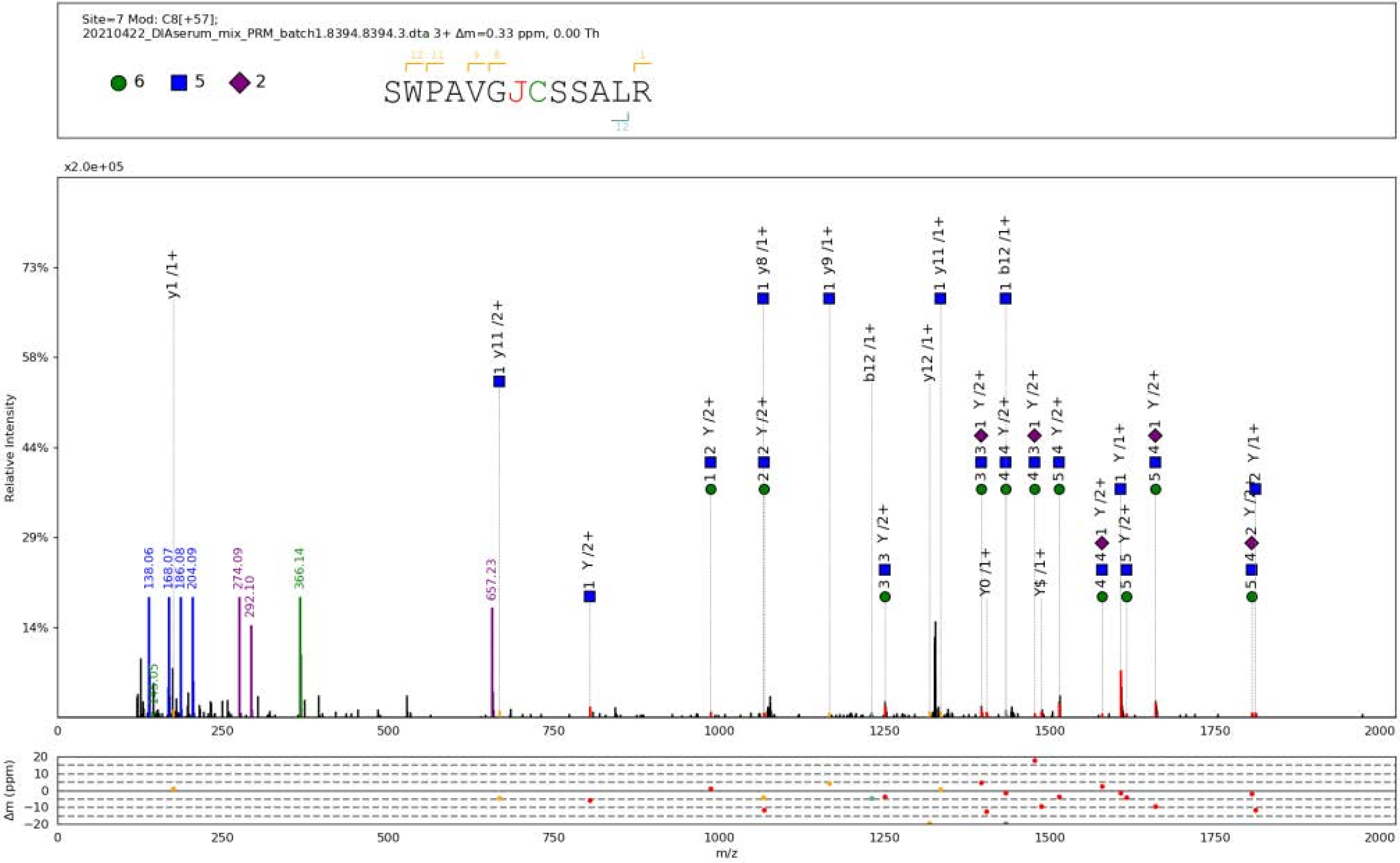

Site=7 Mod: C8[+57];
20210422_DIAserum_mix_PRM_batch1.8394.8394.3.dta 3+ Δm=0.33 ppm, 0.00 Th
6
5
2
SWPAVGJCSSALR
x2.0e+05
Relative Intensity
y1 /1+
1 y11 /2+
1 Y /2+
1 2 Y /2+
2 2 Y /2+
1 y8 /1+
1 y9 /1+
b12 /1+
3 3 Y /2+
y12 /1+
1 y11 /1+
3 3 1 Y /2+
Y0 /1+
1 b12 /1+
4 4 Y /2+
4 3 1 Y /2+
Y$ /1+
5 4 Y /2+
4 4 1 Y /2+
1 Y /1+
5 5 Y /2+
5 4 1 Y /2+
5 4 2 Y /2+
2 Y /1+
138.06
168.07
186.08
204.09
274.09
292.10
366.14
657.23
m/z
Δm (ppm)

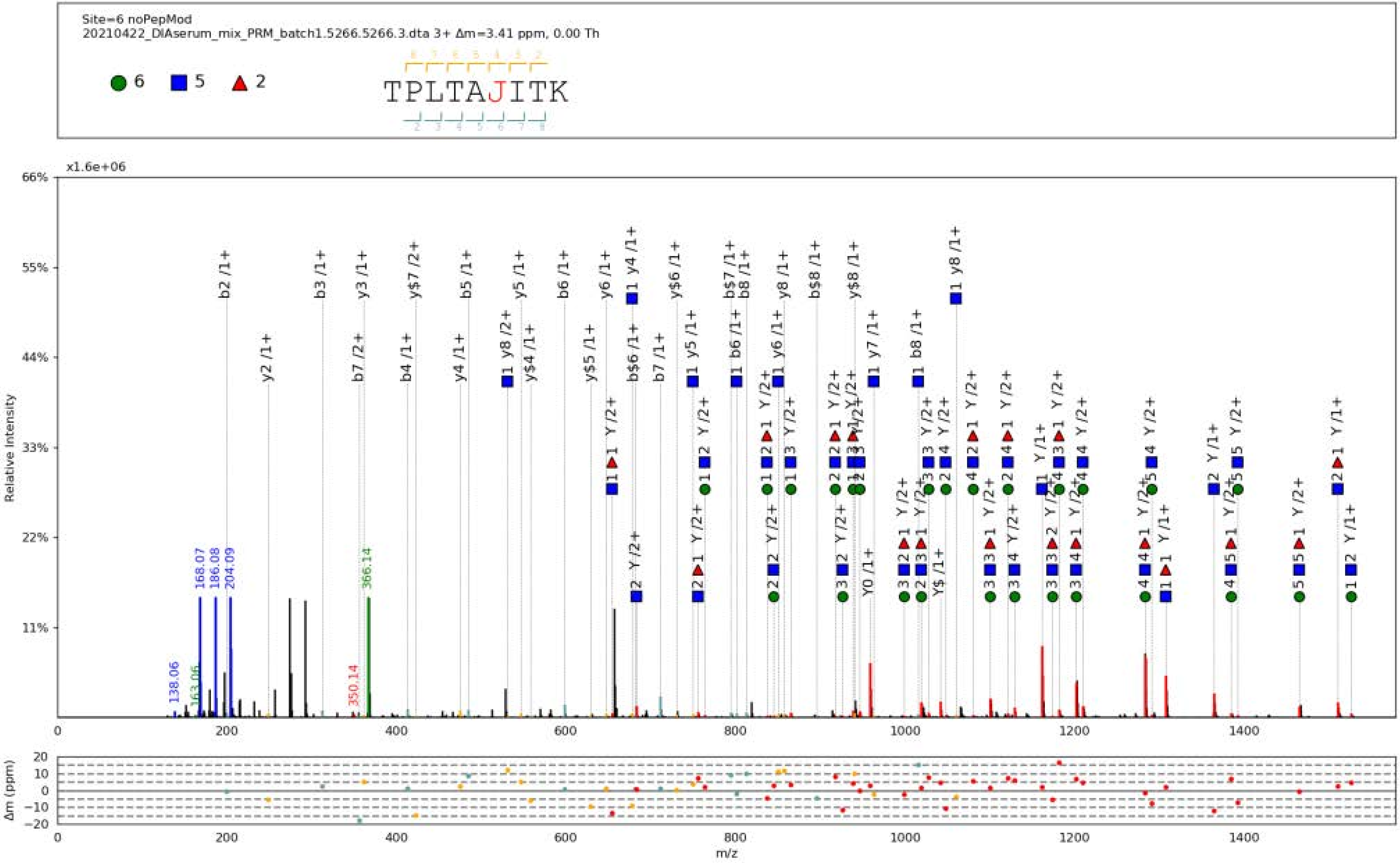
Site=6 noPepMod
20210422_DIAserum_mix_PRM_batch1.5266.5266.3.dta 3+ Δm=3.41 ppm, 0.00 Th
6
5
2
TPLTAJITK
x1.6e+06
Relative Intensity
168.07
186.08
204.09
366.14
138.06
163.06
350.14
m/z
Δm (ppm)

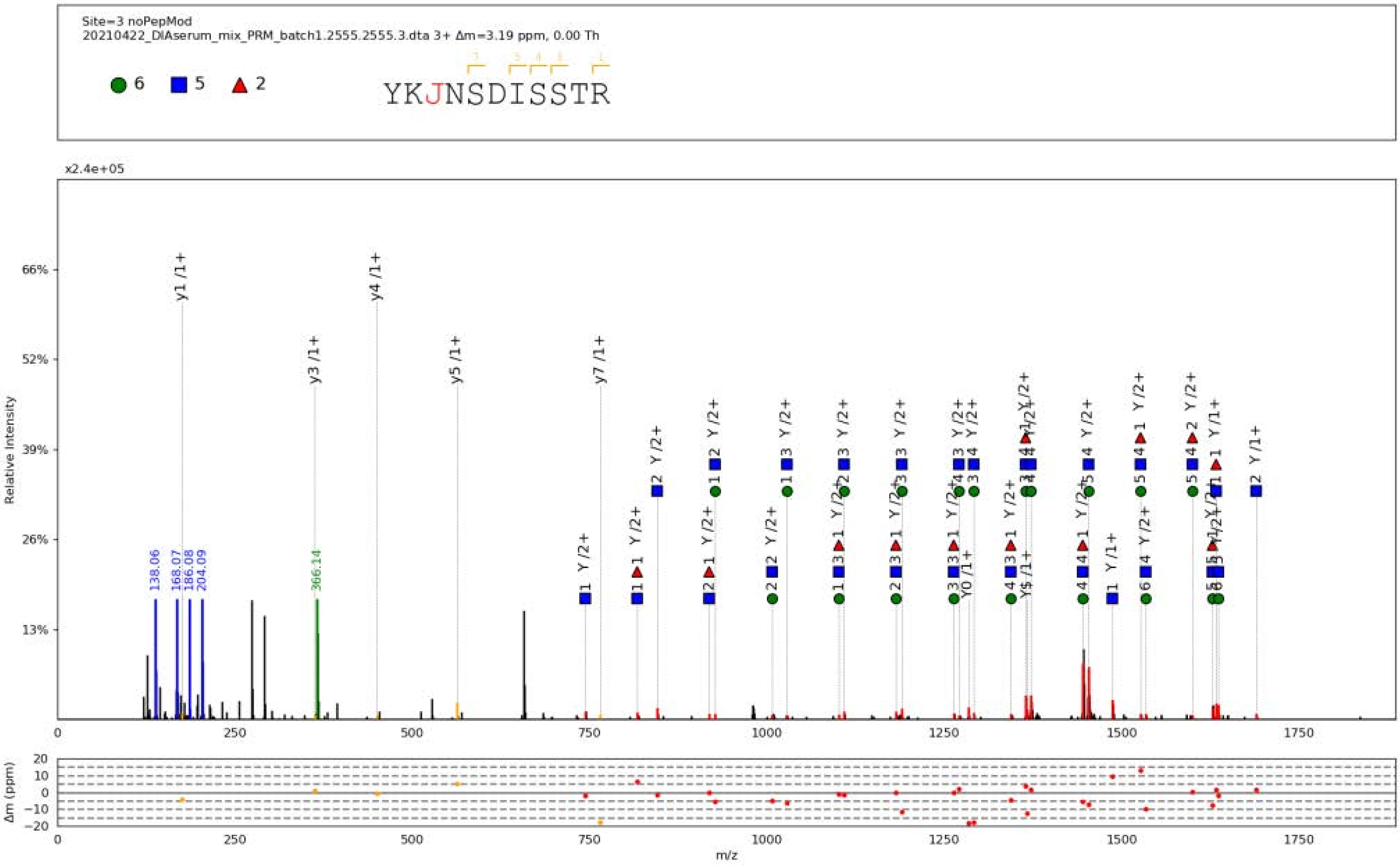
Site=3 noPepMod
20210422_DIAserum_mix_PRM_batch1.2555.2555.3.dta 3+ Δm=3.19 ppm, 0.00 Th
6
5
2
YKJNSDISSTR
x2.4e+05
Relative Intensity
66%
52%
39%
26%
13%
y1 /1+
y3 /1+
y4 /1+
y5 /1+
y7 /1+
138.06
168.07
186.08
204.09
366.14
0
250
500
750
1000
1250
1500
1750
Δm (ppm)
20
10
0
-10
-20
m/z

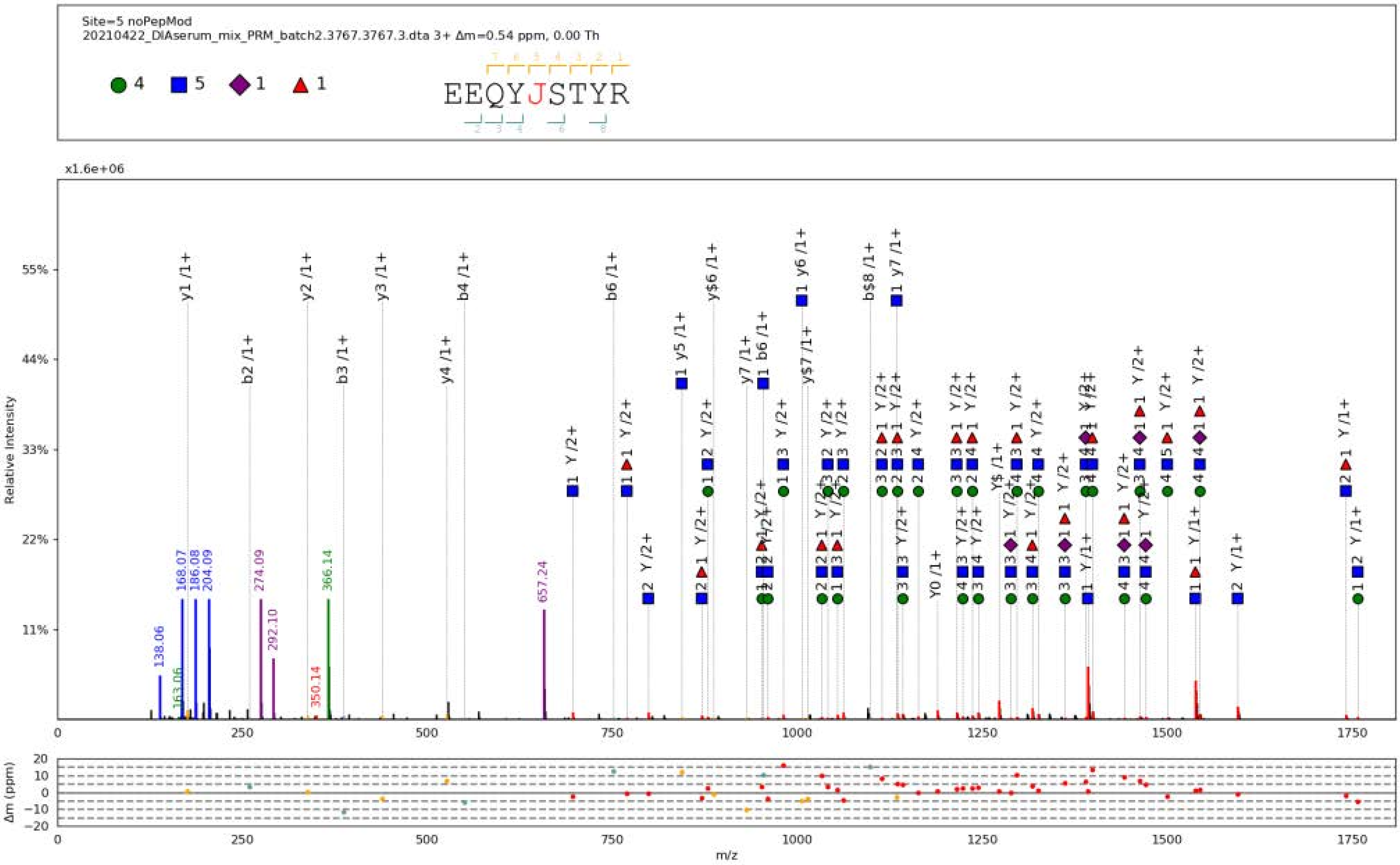
Site=5 noPepMod
20210422_DIAserum_mix_PRM_batch2.3767.3767.3.dta 3+ Δm=0.54 ppm, 0.00 Th
4
5
1
1
EEQYJSTYR
x1.6e+06
Relative Intensity
55%
44%
33%
22%
11%
y1 /1+
b2 /1+
y2 /1+
b3 /1+
y3 /1+
y4 /1+
b4 /1+
b6 /1+
1 y5 /1+
y$6 /1+
y7 /1+
1 b6 /1+
1 y6 /1+
y$7 /1+
b$8 /1+
1 y7 /1+
Y0 /1+
Y$ /1+
138.06
163.06
168.07
186.08
204.09
274.09
292.10
350.14
366.14
657.24
m/z
Δm (ppm)

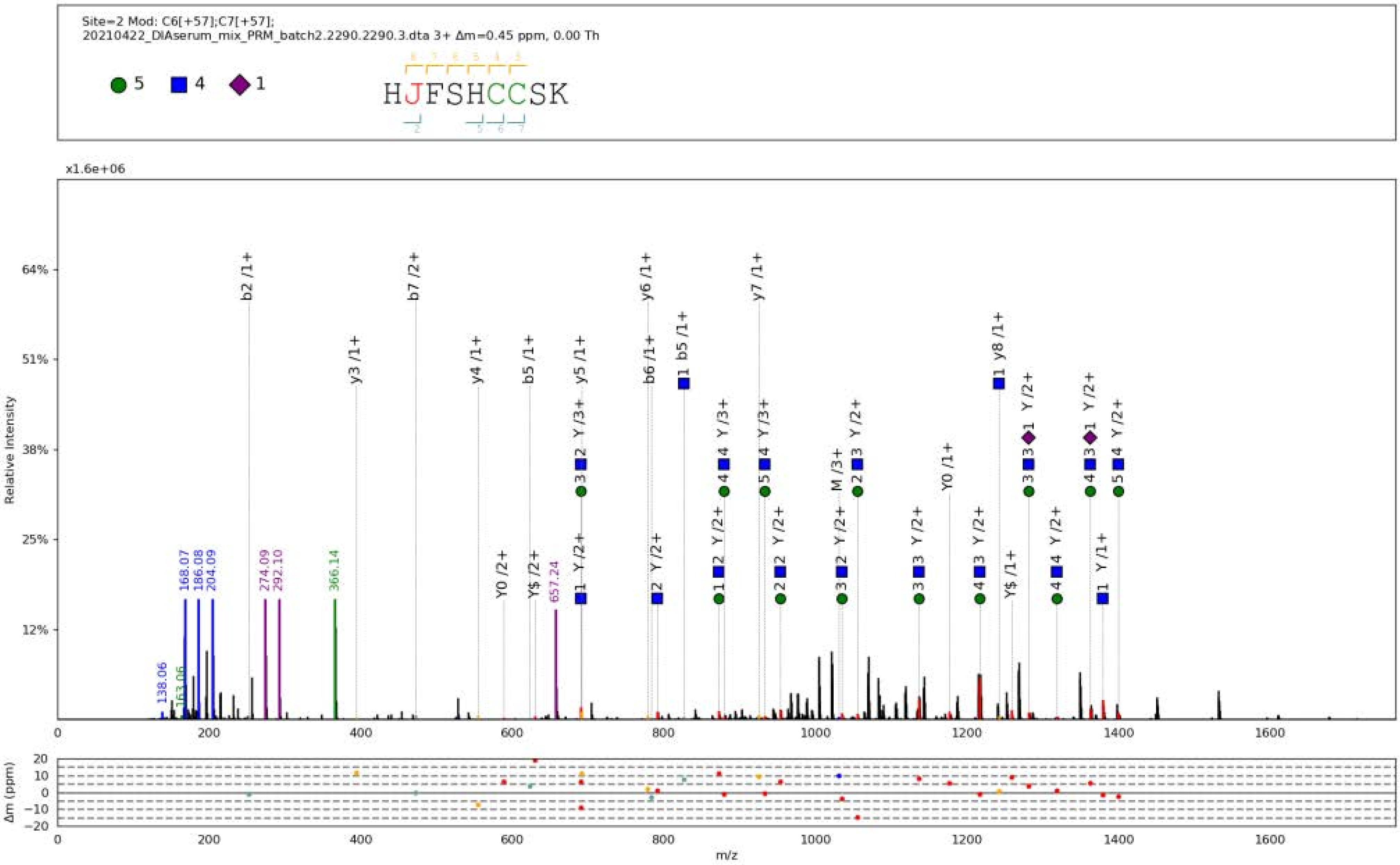

Site=2 Mod: C6[+57];C7[+57];
20210422_DIAserum_mix_PRM_batch2.2290.2290.3.dta 3+ Δm=0.45 ppm, 0.00 Th
5
4
1
HJFSHCCSK
x1.6e+06
Relative Intensity
m/z
Δm (ppm)
b2 /1+
b7 /2+
y3 /1+
y4 /1+
b5 /1+
y5 /1+
y6 /1+
b6 /1+
b5 /1+
y7 /1+
Y0 /1+
y8 /1+
M /3+
Y0 /2+
Y$ /2+
Y$ /1+
138.06
163.06
168.07
186.08
204.09
274.09
292.10
366.14
657.24

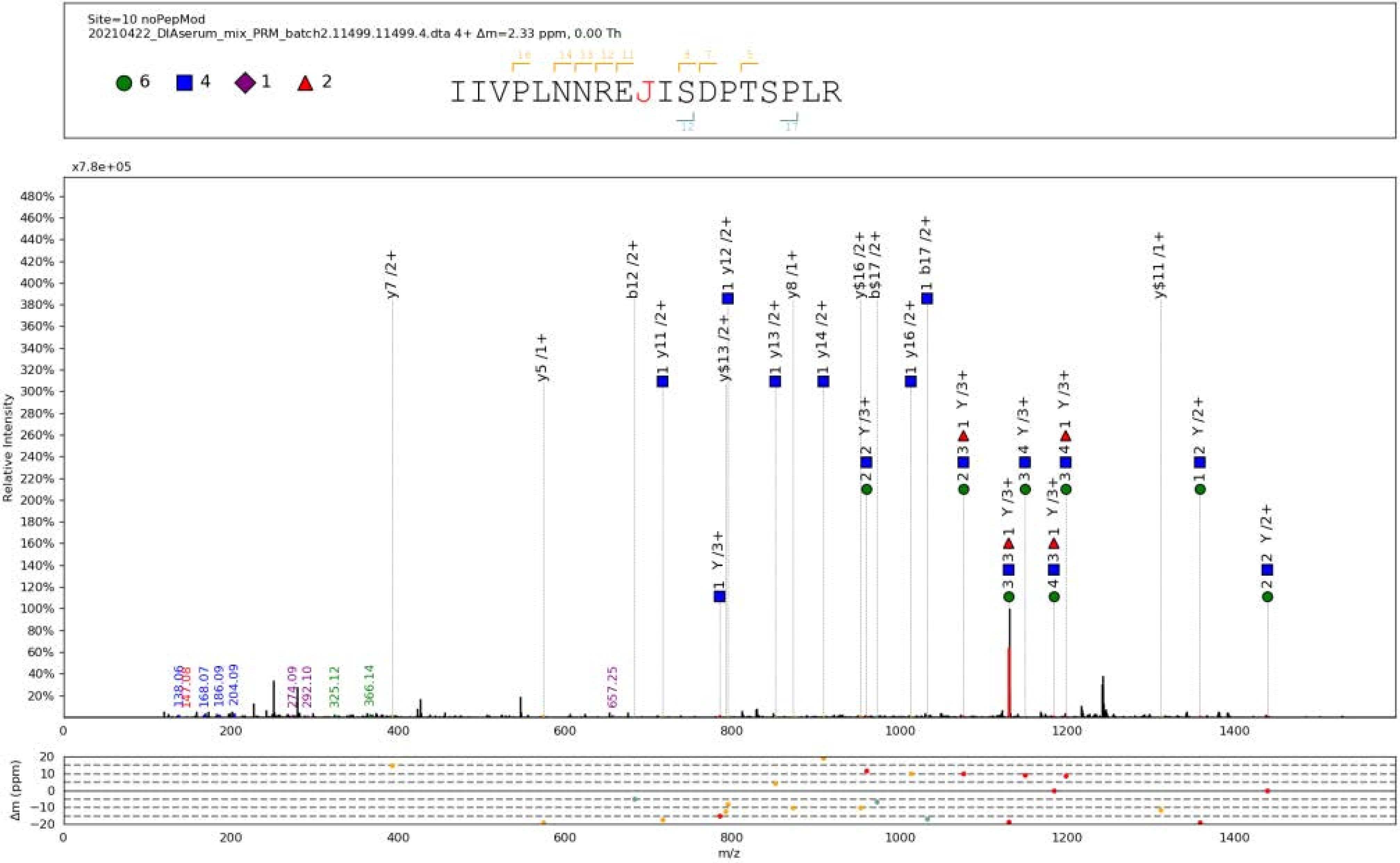
Site=10 noPepMod
20210422_DIAserum_mix_PRM_batch2.11499.11499.4.dta 4+ Δm=2.33 ppm, 0.00 Th
6
4
1
2
IIVPLNNREJISDPTSPLR
x7.8e+05
Relative Intensity
y7 /2+
y5 /1+
b12 /2+
1 y11 /2+
y$13 /2+
1 y12 /2+
1 y13 /2+
y8 /1+
1 y14 /2+
y$16 /2+
b$17 /2+
1 y16 /2+
1 b17 /2+
y$11 /1+
1 Y /3+
2 2 Y /3+
2 3 1 Y /3+
3 3 1 Y /3+
3 4 Y /3+
4 3 1 Y /3+
3 4 1 Y /3+
1 2 Y /2+
2 2 Y /2+
138.06
147.08
168.07
186.09
204.09
274.09
292.10
325.12
366.14
657.25
Δm (ppm)
m/z

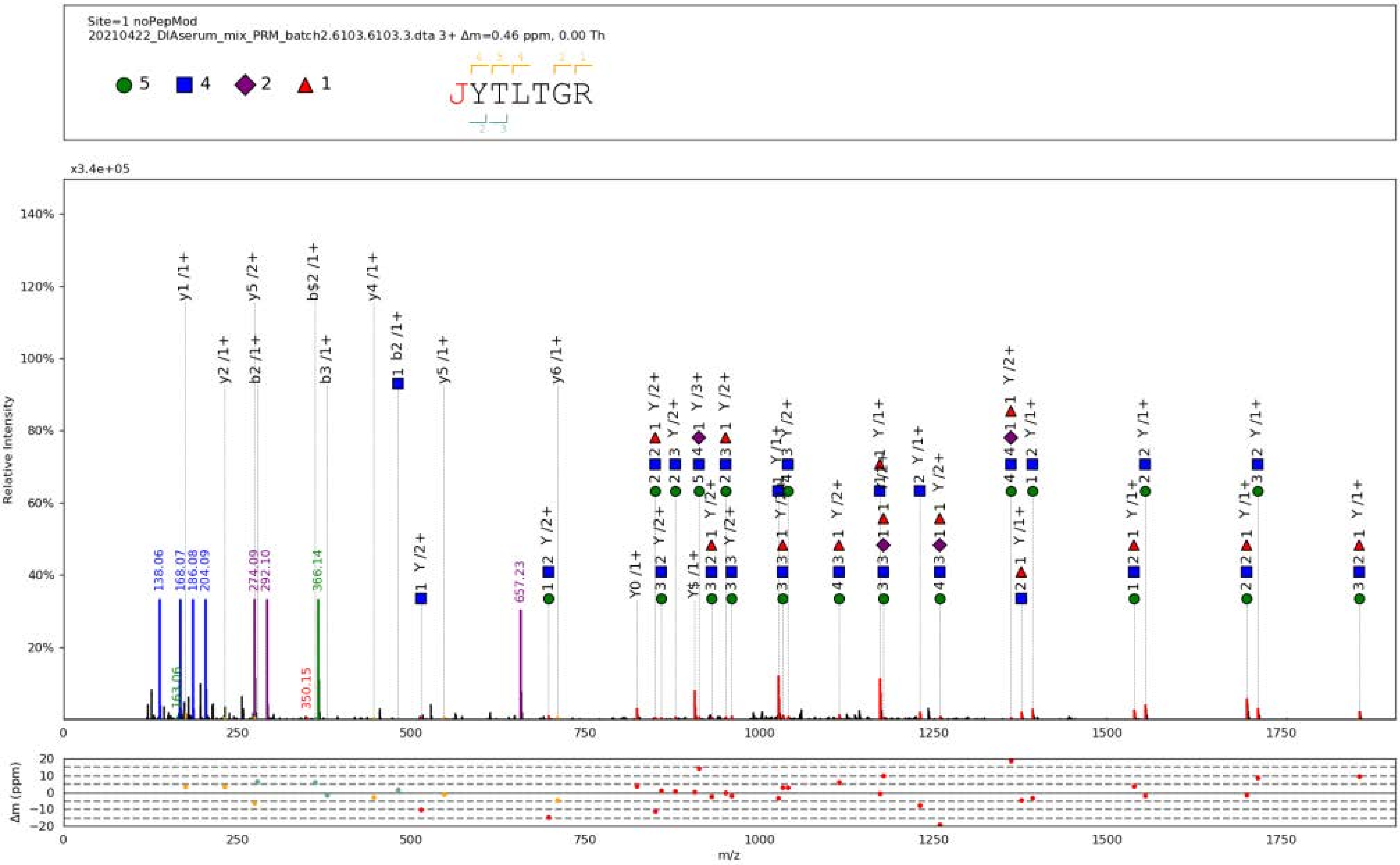

Site=1 noPepMod
20210422_DIAserum_mix_PRM_batch2.6103.6103.3.dta 3+ Δm=0.46 ppm, 0.00 Th
5
4
2
1
JYTLTGR
x3.4e+05
Relative Intensity
m/z
Δm (ppm)
y1 /1+
y2 /1+
y5 /2+
b2 /1+
b$2 /1+
b3 /1+
y4 /1+
b2 /1+
y5 /1+
y6 /1+
Y0 /1+
Y$ /1+
138.06
168.07
186.08
204.09
163.06
274.09
292.10
350.15
366.14
657.23

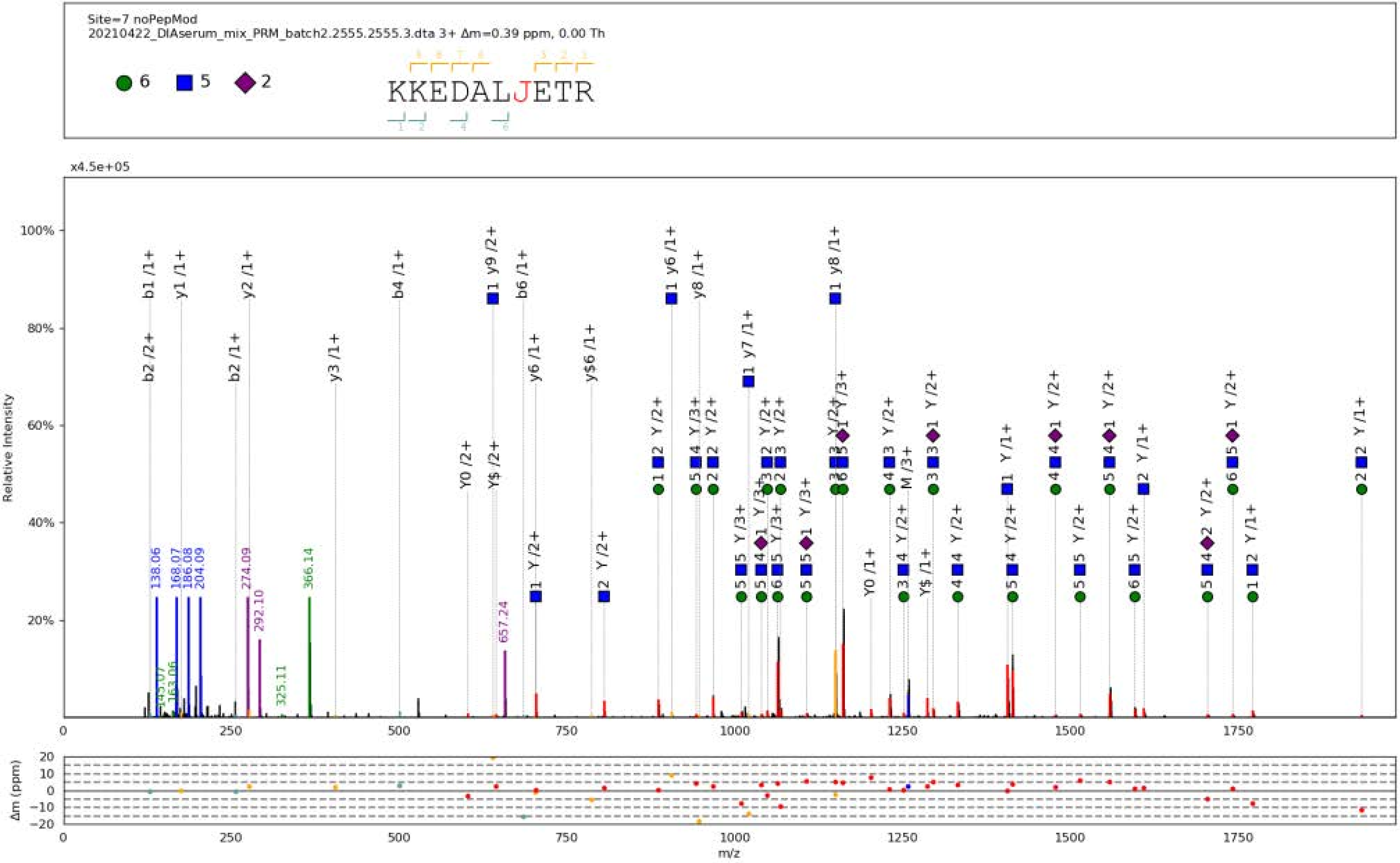

Site=7 noPepMod
20210422_DIAserum_mix_PRM_batch2.2555.2555.3.dta 3+ Δm=0.39 ppm, 0.00 Th
6
5
2
KKEDALJETR
x4.5e+05
Relative Intensity
100%
80%
60%
40%
20%
b1 /1+
b2 /2+
y1 /1+
b2 /1+
y2 /1+
y3 /1+
b4 /1+
1 y9 /2+
Y0 /2+
Y$ /2+
b6 /1+
y6 /1+
y$6 /1+
1 y6 /1+
y8 /1+
1 y7 /1+
1 y8 /1+
138.06
168.07
186.08
204.09
274.09
292.10
366.14
145.07
163.06
325.11
657.24
Y0 /1+
Y$ /1+
M /3+
0
250
500
750
1000
1250
1500
1750
Δm (ppm)
20
10
0
-10
-20
m/z

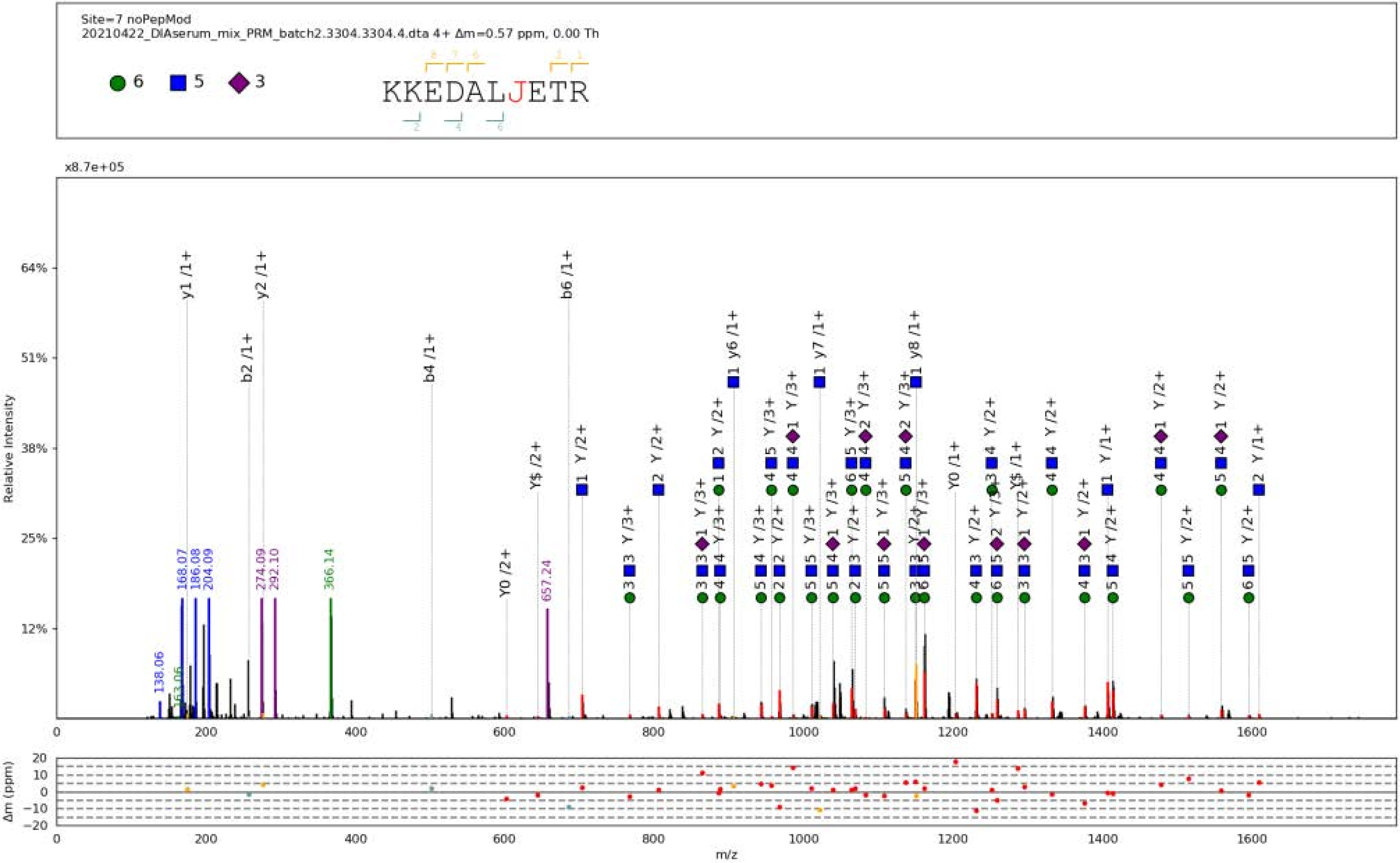
Site=7 noPepMod
20210422_DIAserum_mix_PRM_batch2.3304.3304.4.dta 4+ Δm=0.57 ppm, 0.00 Th
6
5
3
KKEDALJETR
x8.7e+05
Relative Intensity
64%
51%
38%
25%
12%
y1 /1+
y2 /1+
b2 /1+
b4 /1+
b6 /1+
Y0 /2+
Y$ /2+
y6 /1+
y7 /1+
y8 /1+
Y0 /1+
Y$ /1+
138.06
168.07
186.08
204.09
274.09
292.10
366.14
657.24
Δm (ppm)
m/z

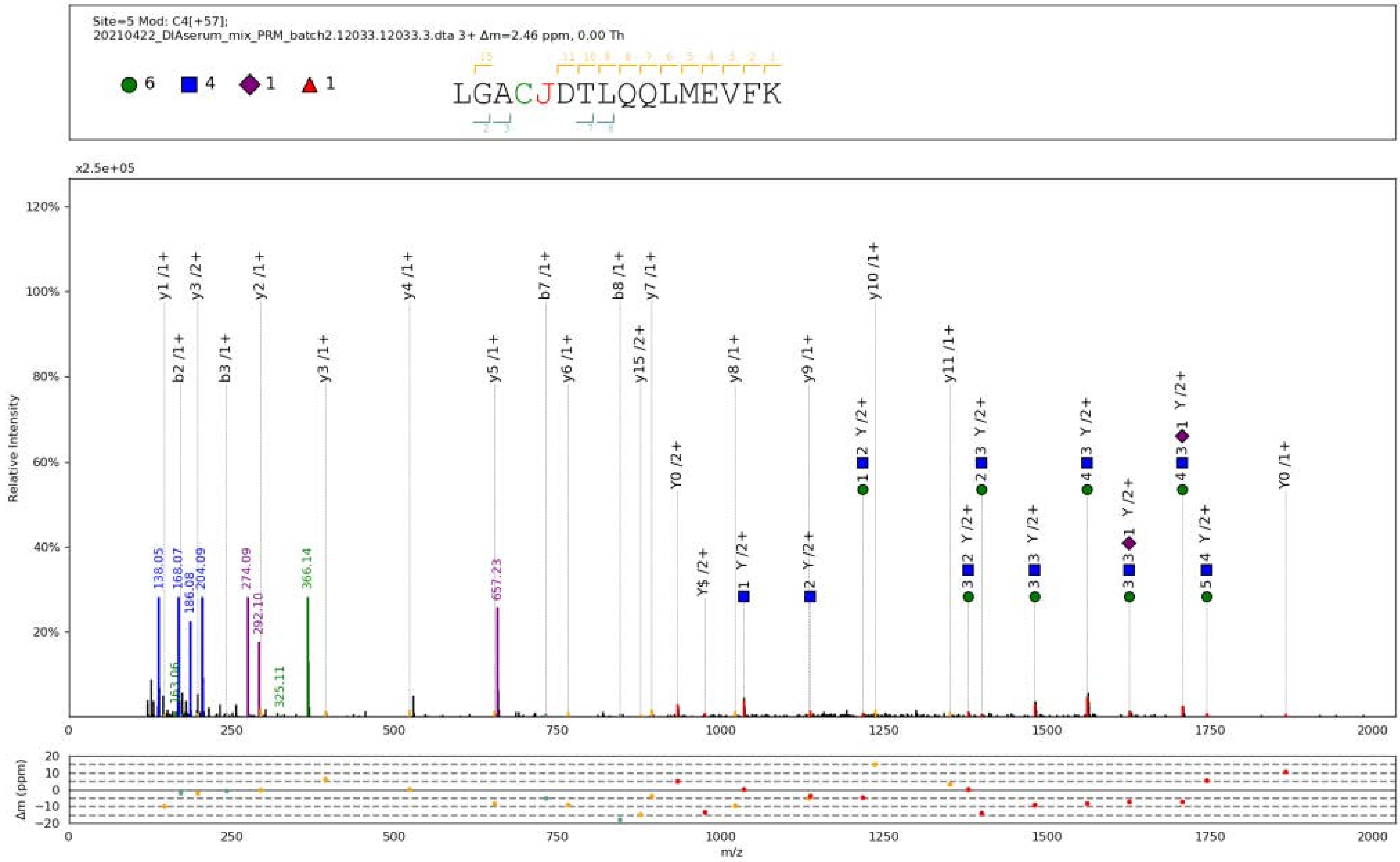

Site=5 Mod: C4[+57];
20210422_DIAserum_mix_PRM_batch2.12033.12033.3.dta 3+ Δm=2.46 ppm, 0.00 Th
6 4 1 1
LGACJDTLQQLMEVFK
x2.5e+05
Relative Intensity
m/z
Δm (ppm)

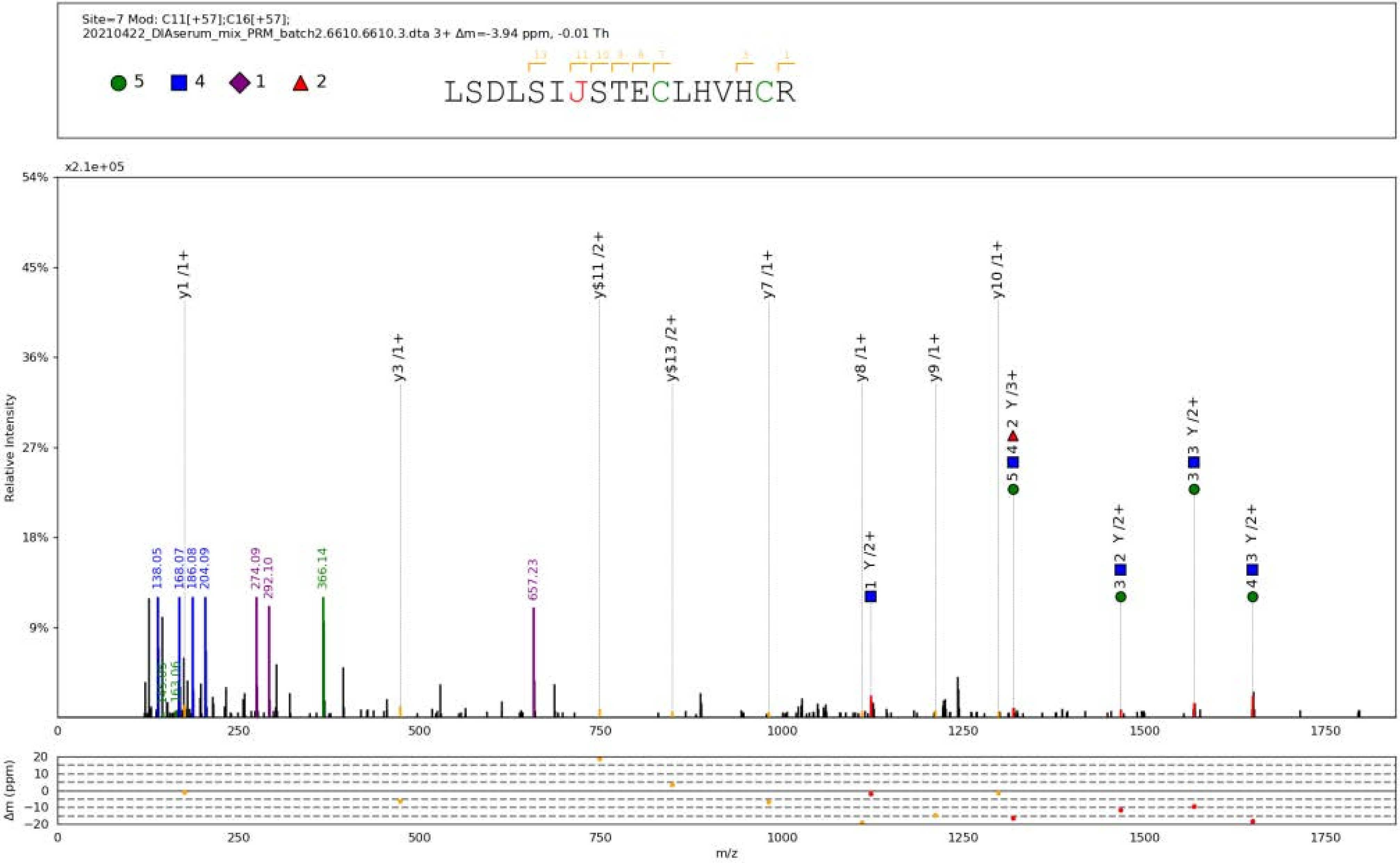
Site=7 Mod: C11[+57];C16[+57];
20210422_DIAserum_mix_PRM_batch2.6610.6610.3.dta 3+ Δm=-3.94 ppm, -0.01 Th
5
4
1
2
LSDLSIJSTECLHVHCR
x2.1e+05
Relative Intensity
y1 /1+
y3 /1+
y$11 /2+
y$13 /2+
y7 /1+
y8 /1+
y9 /1+
y10 /1+
138.05
168.07
186.08
204.09
274.09
292.10
366.14
657.23
163.06
m/z
Δm (ppm)

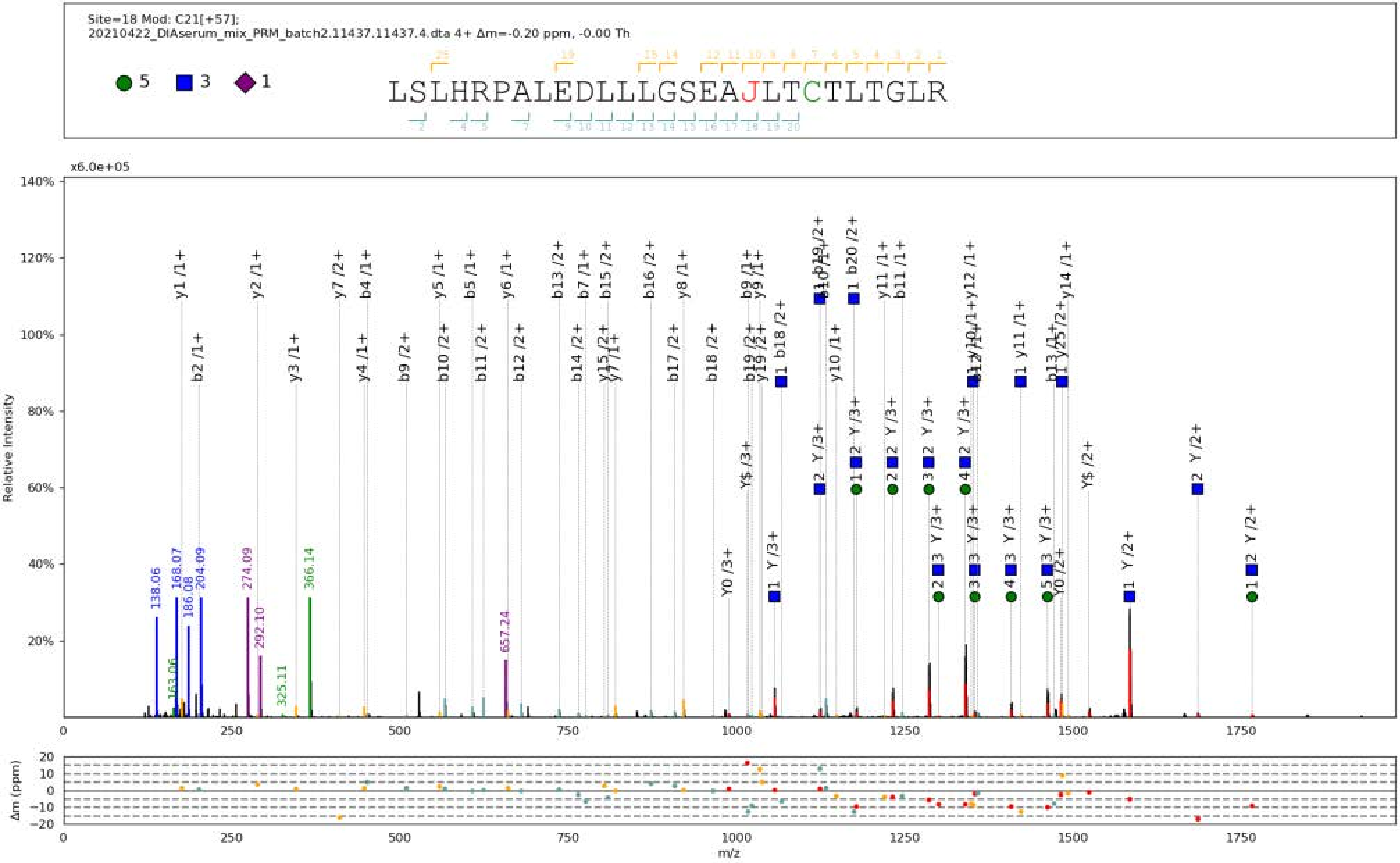
Site=18 Mod: C21[+57];
20210422_DIAserum_mix_PRM_batch2.11437.11437.4.dta 4+ Δm=-0.20 ppm, -0.00 Th
LSLHRPALEDLLLGSEAJLTCTLTGLR
x6.0e+05
Relative Intensity
Δm (ppm)
m/z

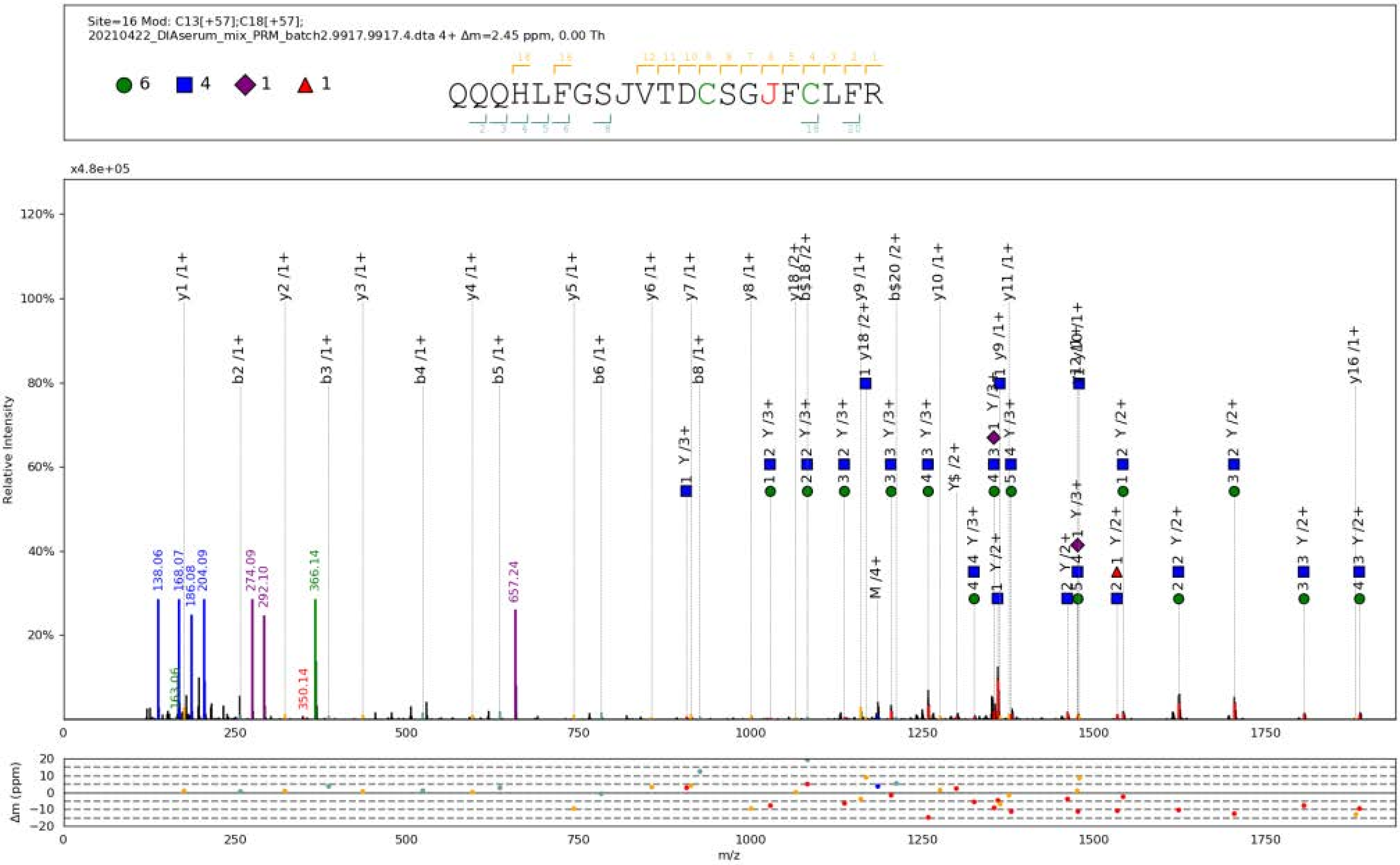
Site=16 Mod: C13[+57];C18[+57];
20210422_DIAserum_mix_PRM_batch2.9917.9917.4.dta 4+ Δm=2.45 ppm, 0.00 Th
6
4
1
1
QQQHLFGSJVTDCSGJFCLFR
x4.8e+05
Relative Intensity
m/z
Δm (ppm)
138.06
168.07
186.08
204.09
274.09
292.10
366.14
657.24
350.14
163.06
y1 /1+
y2 /1+
y3 /1+
y4 /1+
y5 /1+
y6 /1+
y7 /1+
y8 /1+
y10 /1+
y11 /1+
y16 /1+
b2 /1+
b3 /1+
b4 /1+
b5 /1+
b6 /1+
b8 /1+
b$20 /2+
M /4+

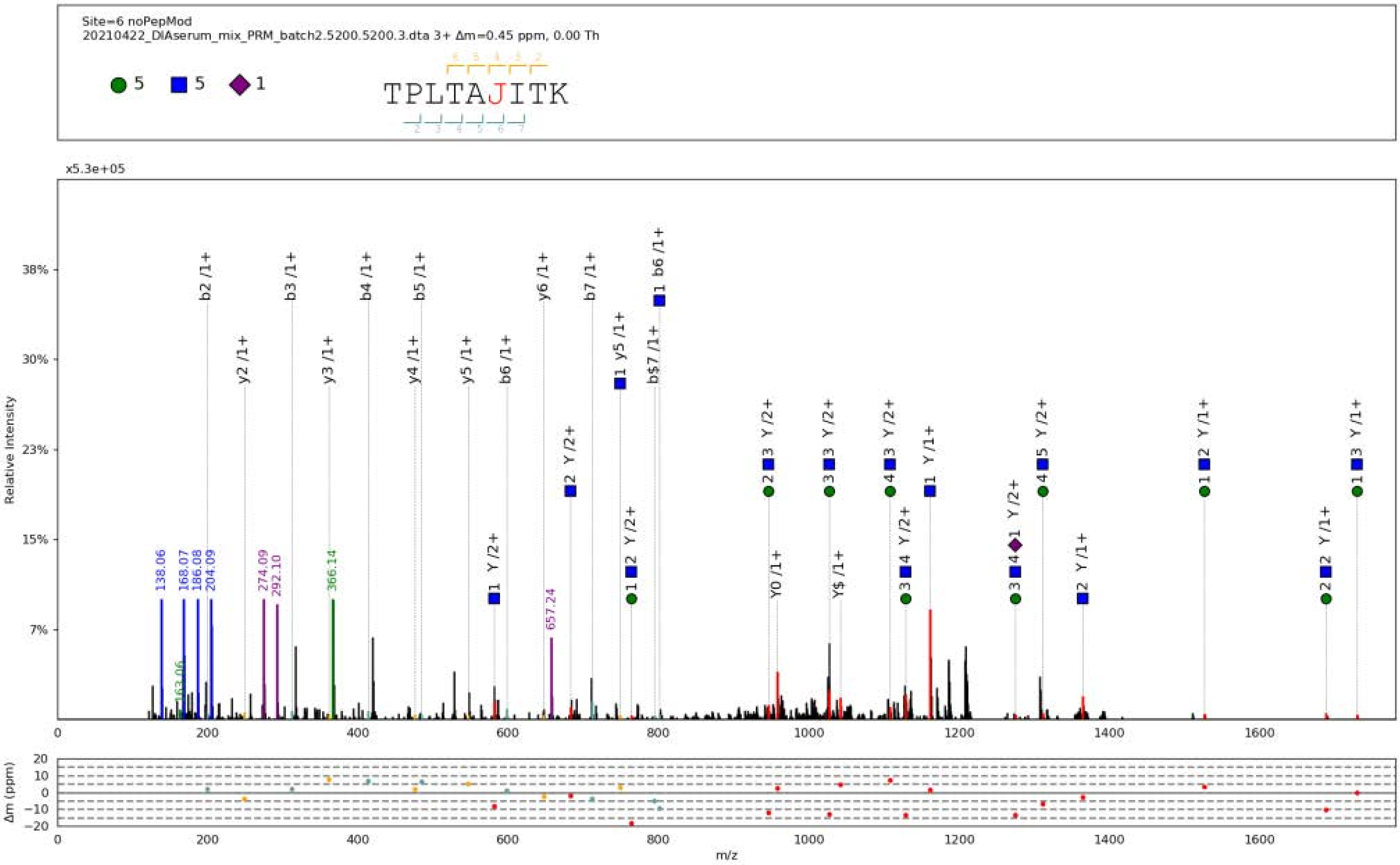

Site=6 noPepMod
20210422_DIAserum_mix_PRM_batch2.5200.5200.3.dta 3+ Δm=0.45 ppm, 0.00 Th
TPLTAJITK
x5.3e+05
Relative Intensity
m/z
Δm (ppm)

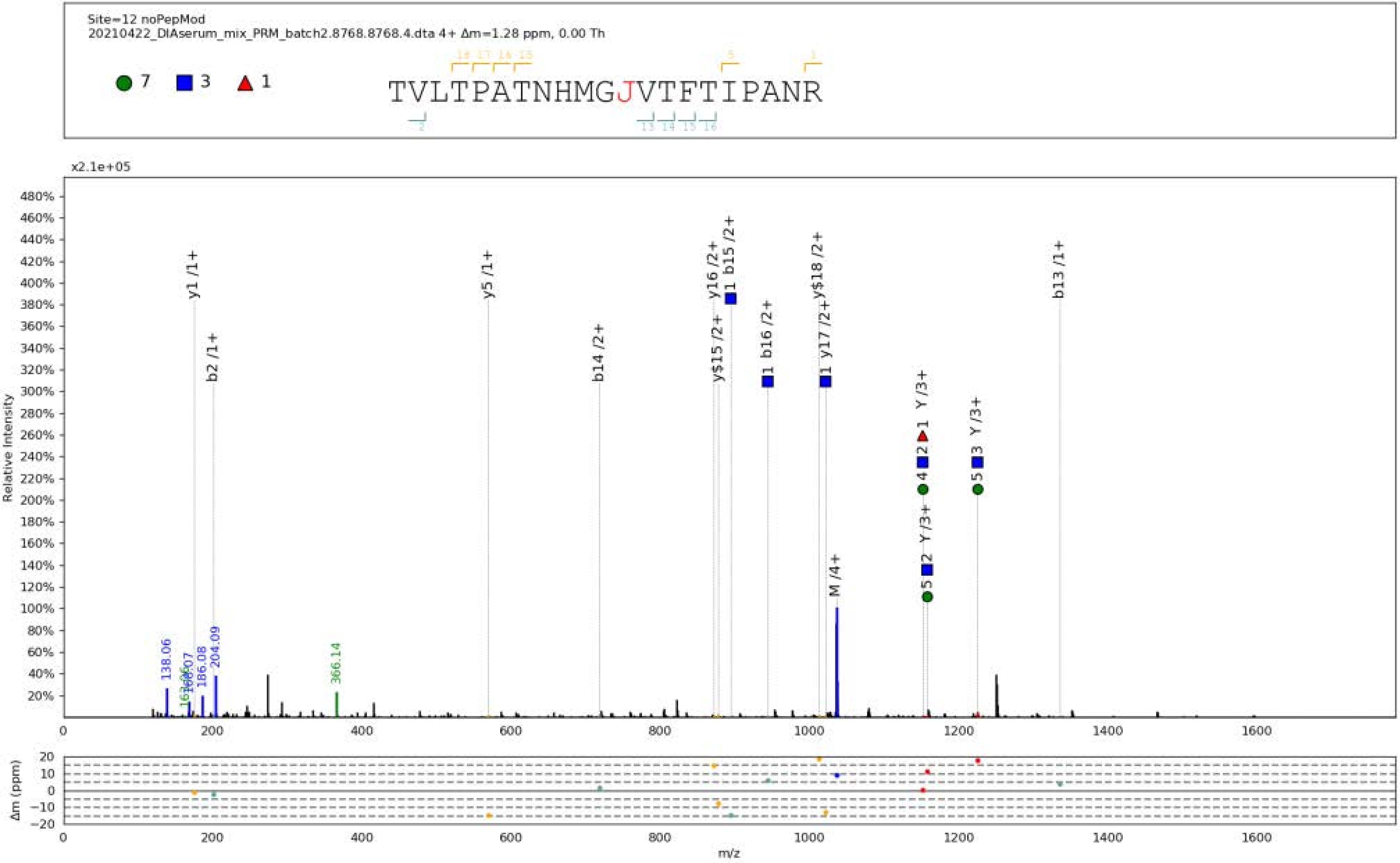
Site=12 noPepMod
20210422_DIAserum_mix_PRM_batch2.8768.8768.4.dta 4+ Δm=1.28 ppm, 0.00 Th
7
3
1
TVLTPATNHMGJVTFTIPANR
x2.1e+05
Relative Intensity
y1 /1+
b2 /1+
y5 /1+
b14 /2+
y$15 /2+
y16 /2+
1 b15 /2+
1 b16 /2+
1 y17 /2+
y$18 /2+
M /4+
1 Y /3+
2 Y /3+
3 Y /3+
138.06
186.08
204.09
366.14
b13 /1+
Δm (ppm)
m/z

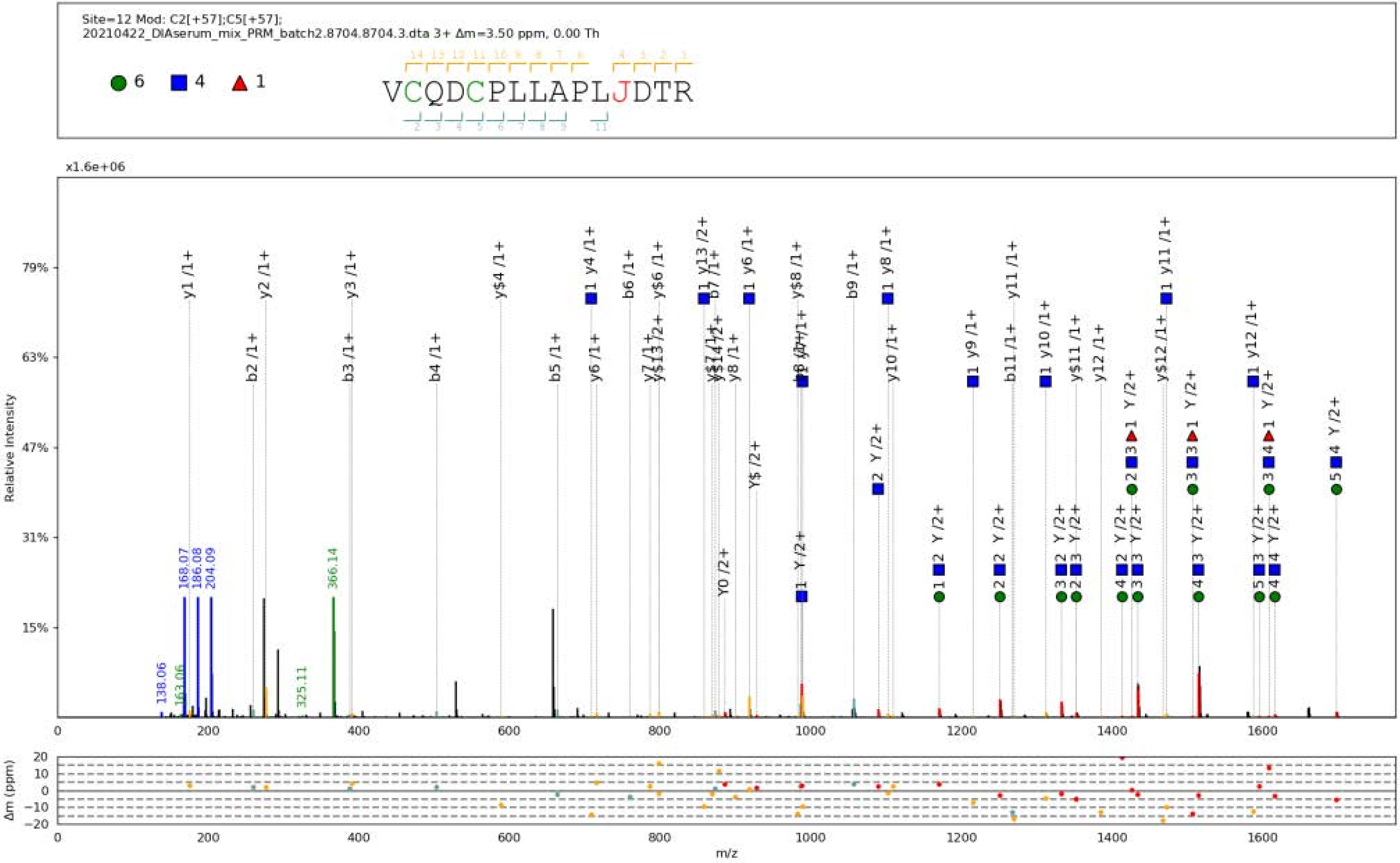

Site=12 Mod: C2[+57];C5[+57];
20210422_DIAserum_mix_PRM_batch2.8704.8704.3.dta 3+ Δm=3.50 ppm, 0.00 Th
6
4
1
VCQDCPLLAPLJDTR
x1.6e+06
Relative Intensity
79%
63%
47%
31%
15%
138.06
163.06
168.07
186.08
204.09
325.11
366.14
y1 /1+
y2 /1+
y3 /1+
y$4 /1+
b2 /1+
b3 /1+
b4 /1+
b5 /1+
b6 /1+
b9 /1+
y11 /1+
y12 /1+
Δm (ppm)
m/z

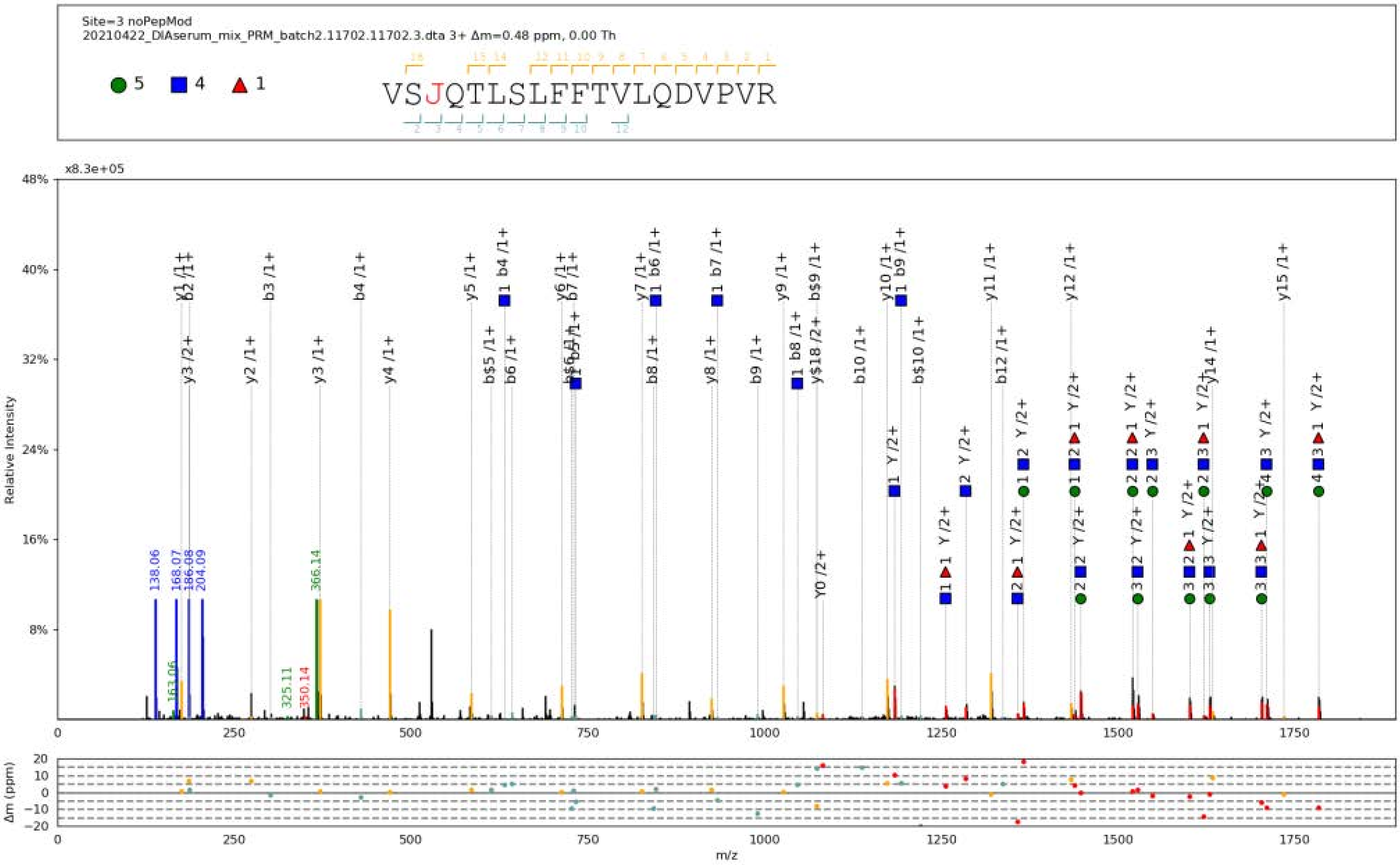

Site=3 noPepMod
20210422_DIAserum_mix_PRM_batch2.11702.11702.3.dta 3+ Δm=0.48 ppm, 0.00 Th
5
4
1
VSJQTLSLFFTVLQDVPVR
x8.3e+05
Relative Intensity
m/z
Δm (ppm)

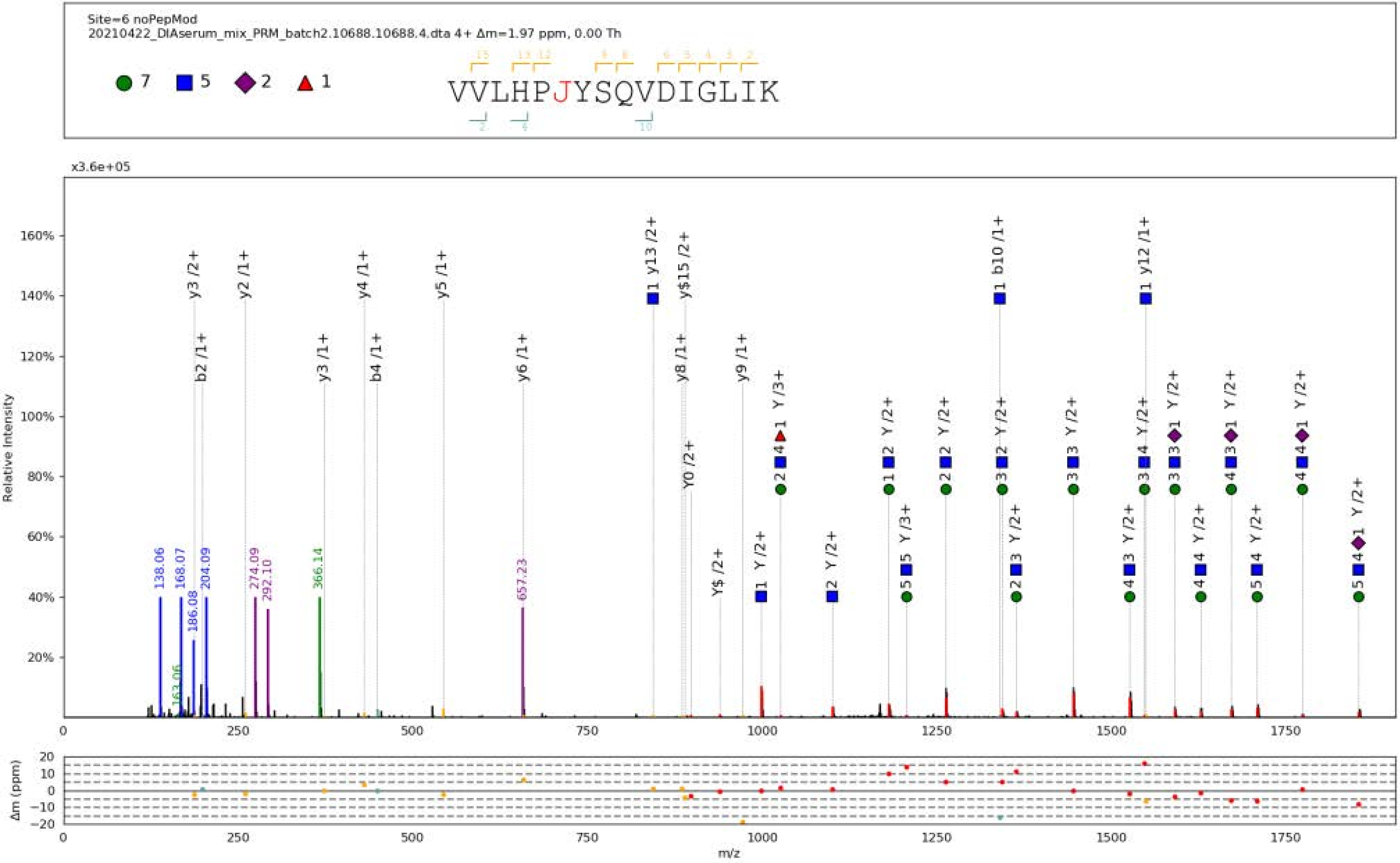

Site=6 noPepMod
20210422_DIAserum_mix_PRM_batch2.10688.10688.4.dta 4+ Δm=1.97 ppm, 0.00 Th
7 5 2 1
VVLHPJYSQVDIGLIK
x3.6e+05
Relative Intensity
Δm (ppm)
m/z

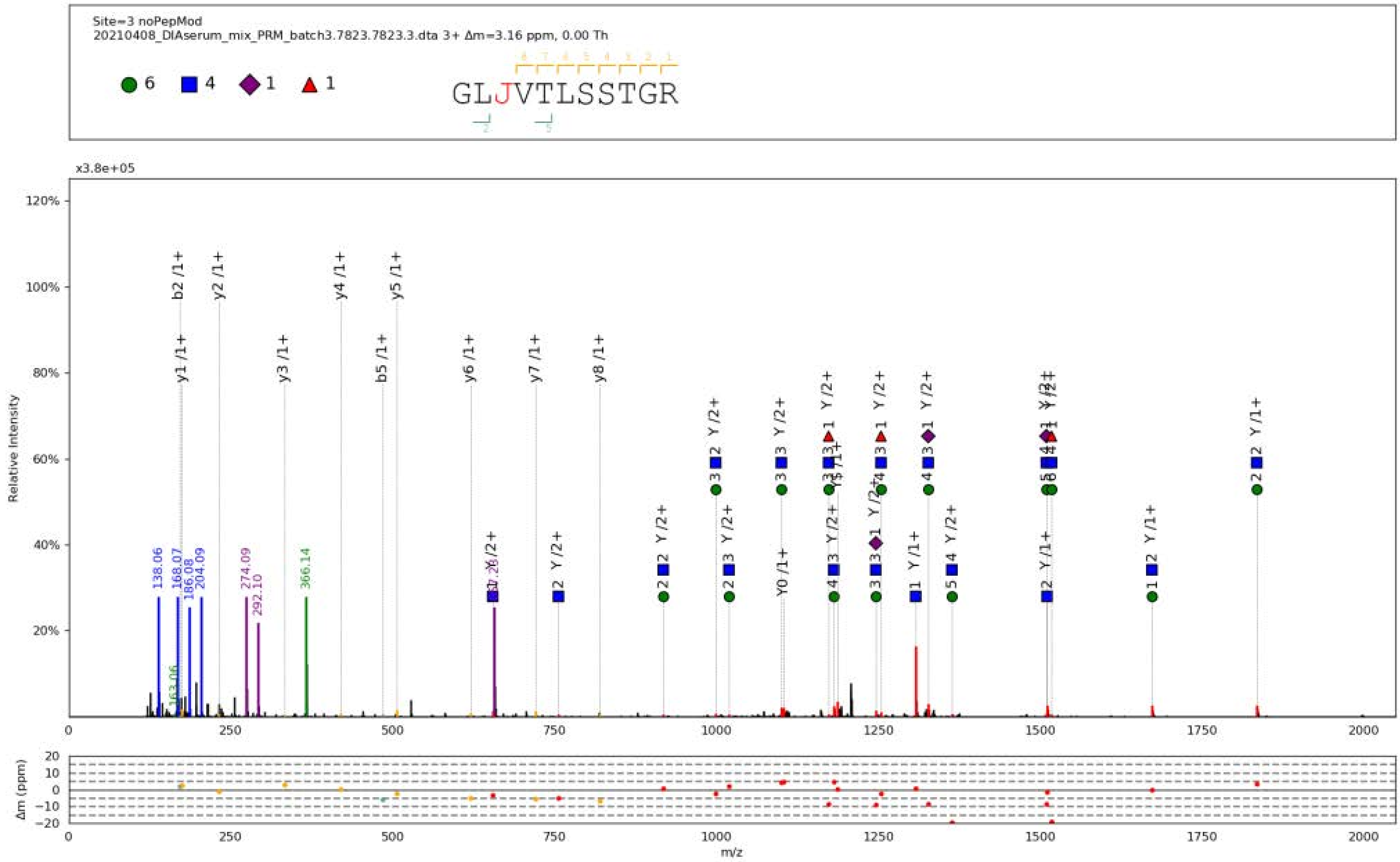

Site=3 noPepMod
20210408_DIAserum_mix_PRM_batch3.7823.7823.3.dta 3+ Δm=3.16 ppm, 0.00 Th
6
4
1
1
GLJVTLSSTGR
x3.8e+05
Relative Intensity
b2 /1+
y1 /1+
y2 /1+
y3 /1+
y4 /1+
b5 /1+
y5 /1+
y6 /1+
y7 /1+
y8 /1+
138.06
168.07
186.08
204.09
163.06
274.09
292.10
366.14
m/z
Δm (ppm)

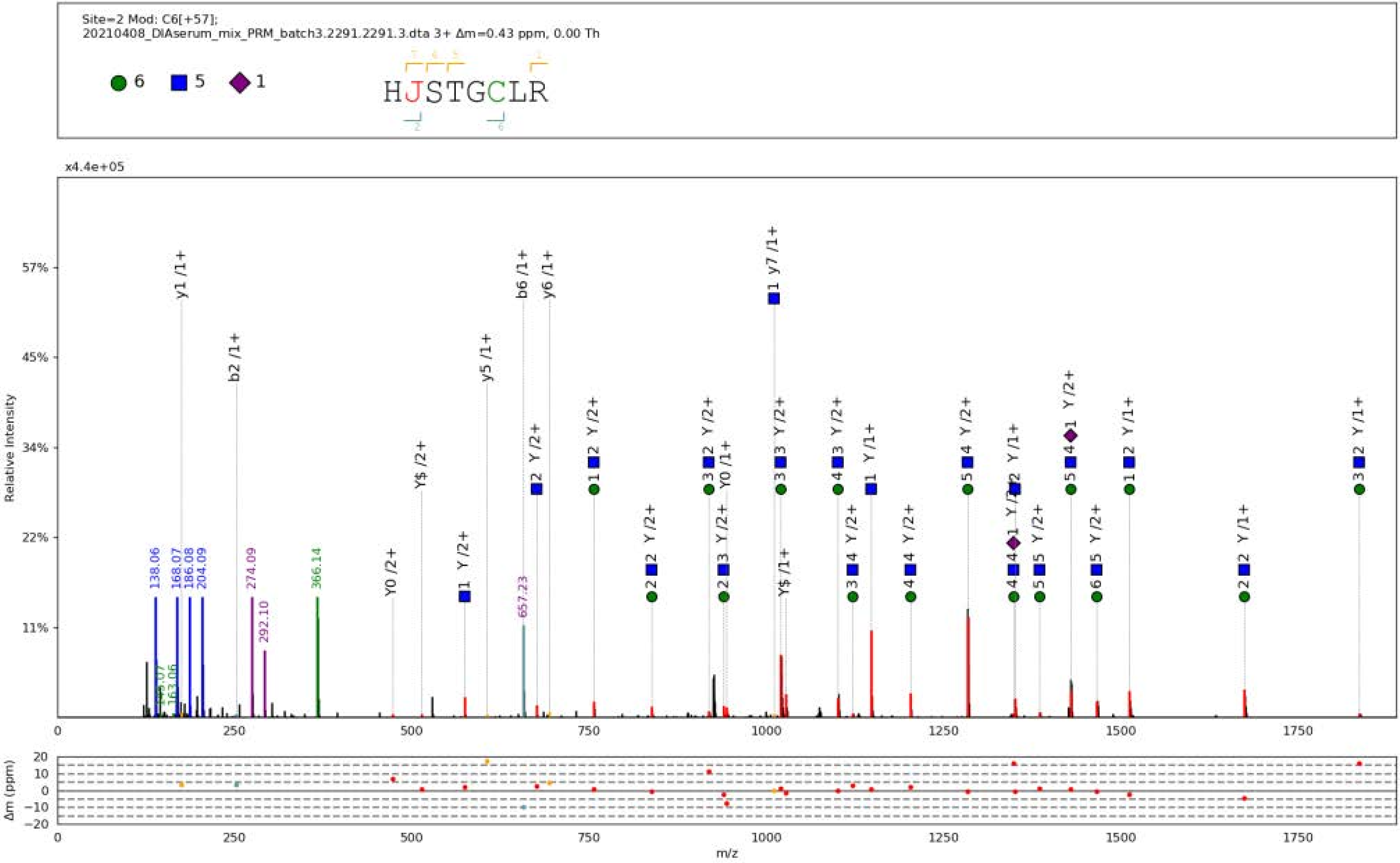
Site=2 Mod: C6[+57];
20210408_DIAserum_mix_PRM_batch3.2291.2291.3.dta 3+ Δm=0.43 ppm, 0.00 Th
6
5
1
HJSTGCLR
x4.4e+05
Relative Intensity
57%
45%
34%
22%
11%
y1 /1+
b2 /1+
y5 /1+
b6 /1+
y6 /1+
1 y7 /1+
Y$ /2+
Y0 /2+
Y0 /1+
Y$ /1+
138.06
168.07
186.08
204.09
274.09
292.10
366.14
657.23
m/z
Δm (ppm)
0
250
500
750
1000
1250
1500
1750

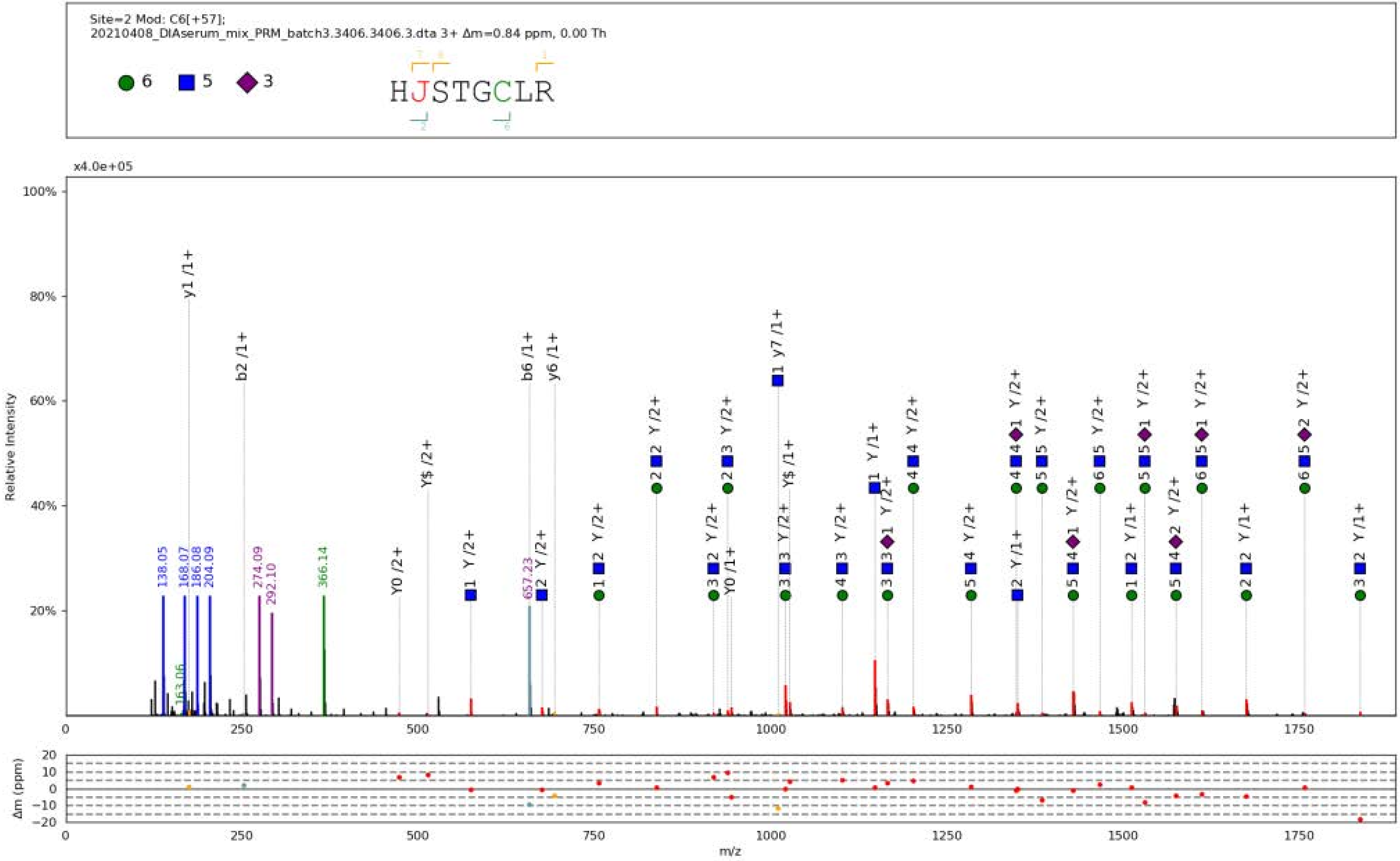
Site=2 Mod: C6[+57];
20210408_DIAserum_mix_PRM_batch3.3406.3406.3.dta 3+ Δm=0.84 ppm, 0.00 Th
6
5
3
HJSTGCLR
x4.0e+05
Relative Intensity
y1 /1+
b2 /1+
b6 /1+
y6 /1+
y7 /1+
Y$ /2+
Y$ /1+
Y0 /2+
Y0 /1+
138.05
168.07
186.08
204.09
274.09
292.10
366.14
163.06
657.23
m/z
Δm (ppm)

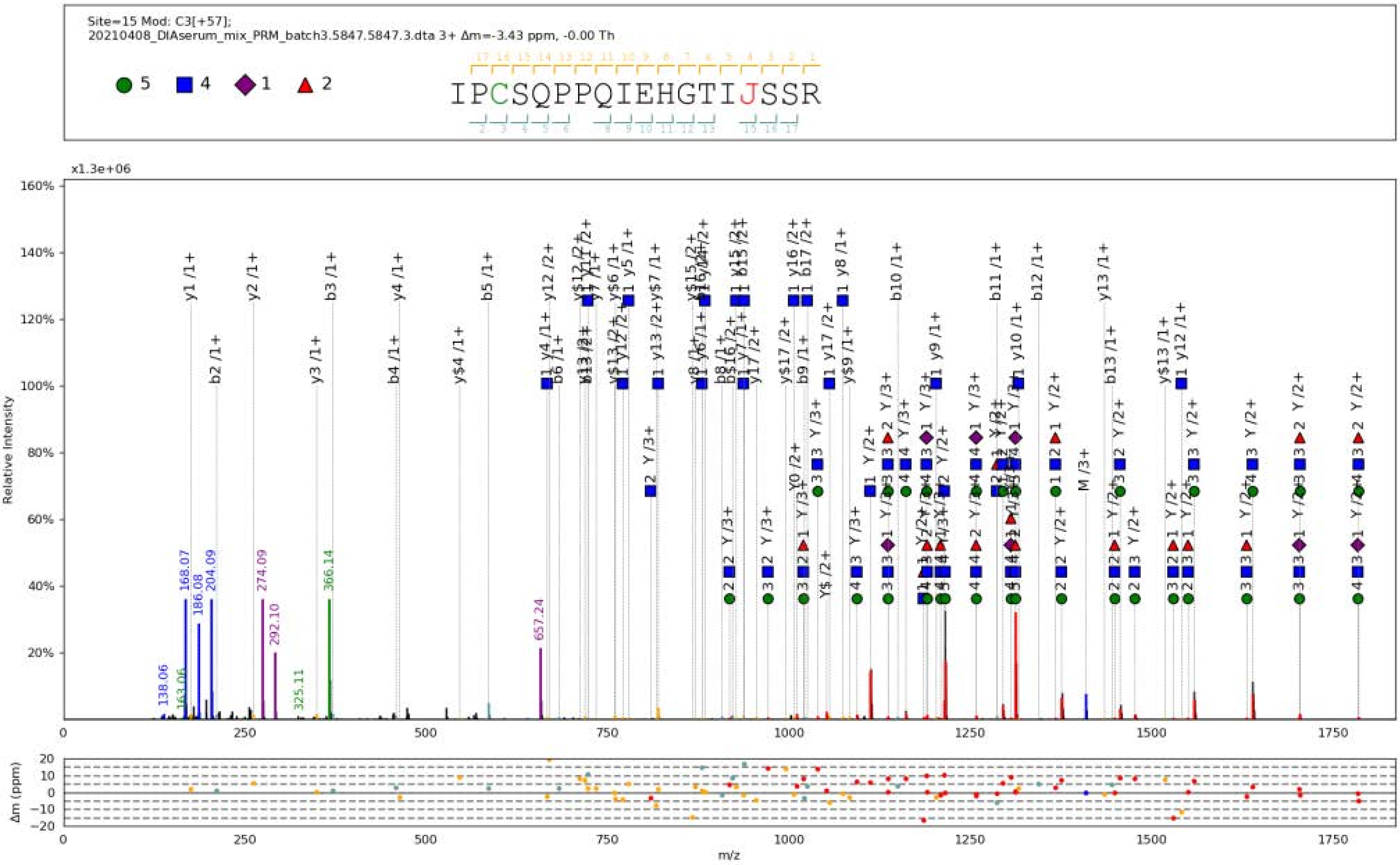
Site=15 Mod: C3[+57];
20210408_DIAserum_mix_PRM_batch3.5847.5847.3.dta 3+ Δm=-3.43 ppm, -0.00 Th
5
4
1
2
IPCSQPPQIEHGTIJSSR
x1.3e+06
Relative Intensity
m/z
Δm (ppm)
y1 /1+
b2 /1+
y2 /1+
y3 /1+
b3 /1+
b4 /1+
y4 /1+
y$4 /1+
b5 /1+
y12 /2+
b6 /1+
b10 /1+
b11 /1+
b12 /1+
y13 /1+
b13 /1+
y$13 /1+
y12 /1+
y10 /1+
y9 /1+
y8 /1+
y$9 /1+
b9 /1+
y17 /2+
y$17 /2+
M /3+
168.07
186.08
204.09
274.09
292.10
366.14
657.24
138.06
163.06
325.11

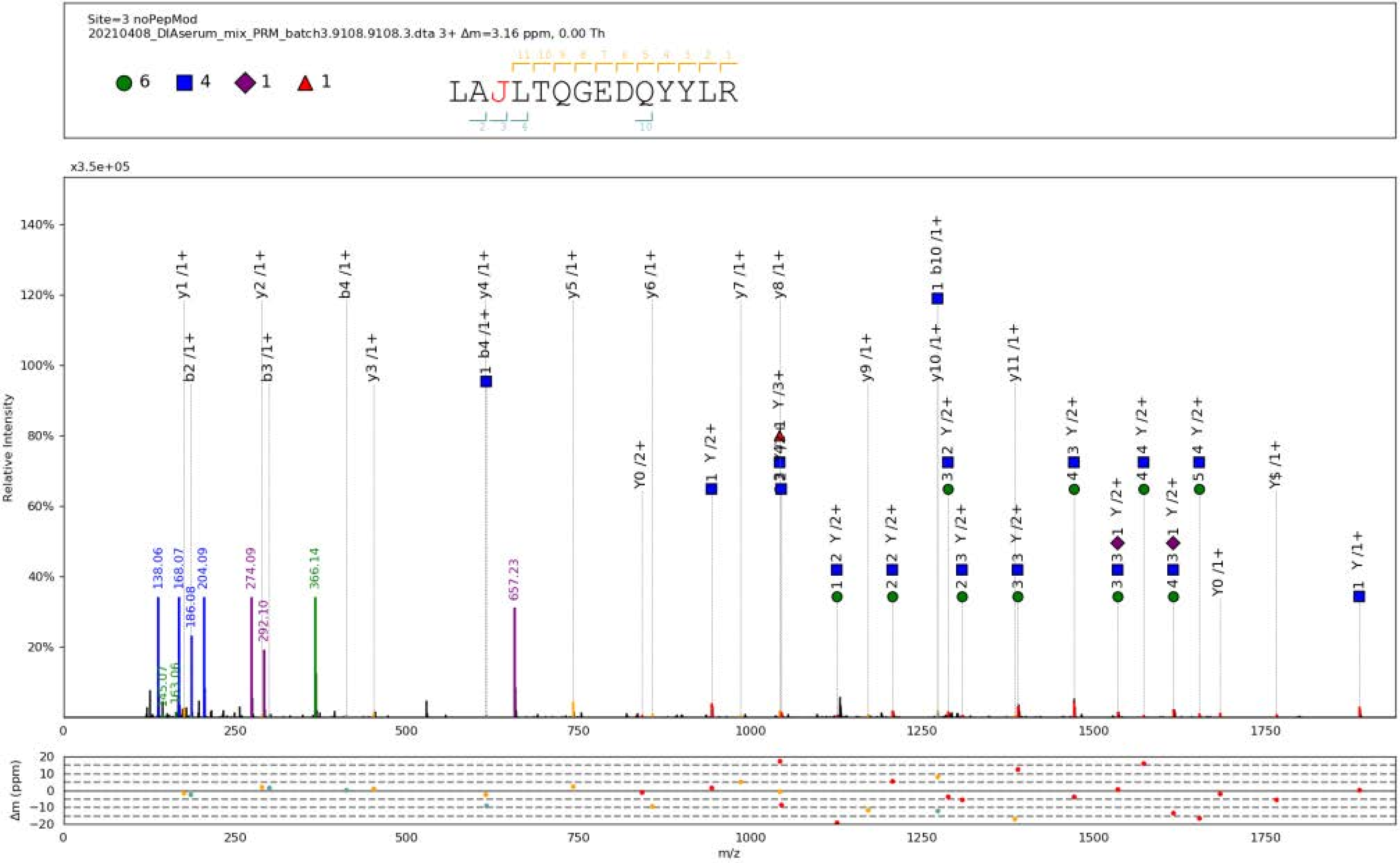

Site=3 noPepMod
20210408_DIAserum_mix_PRM_batch3.9108.9108.3.dta 3+ Δm=3.16 ppm, 0.00 Th
6 4 1 1
LAJLTQGEDQYYLR
x3.5e+05
Relative Intensity
Δm (ppm)
m/z

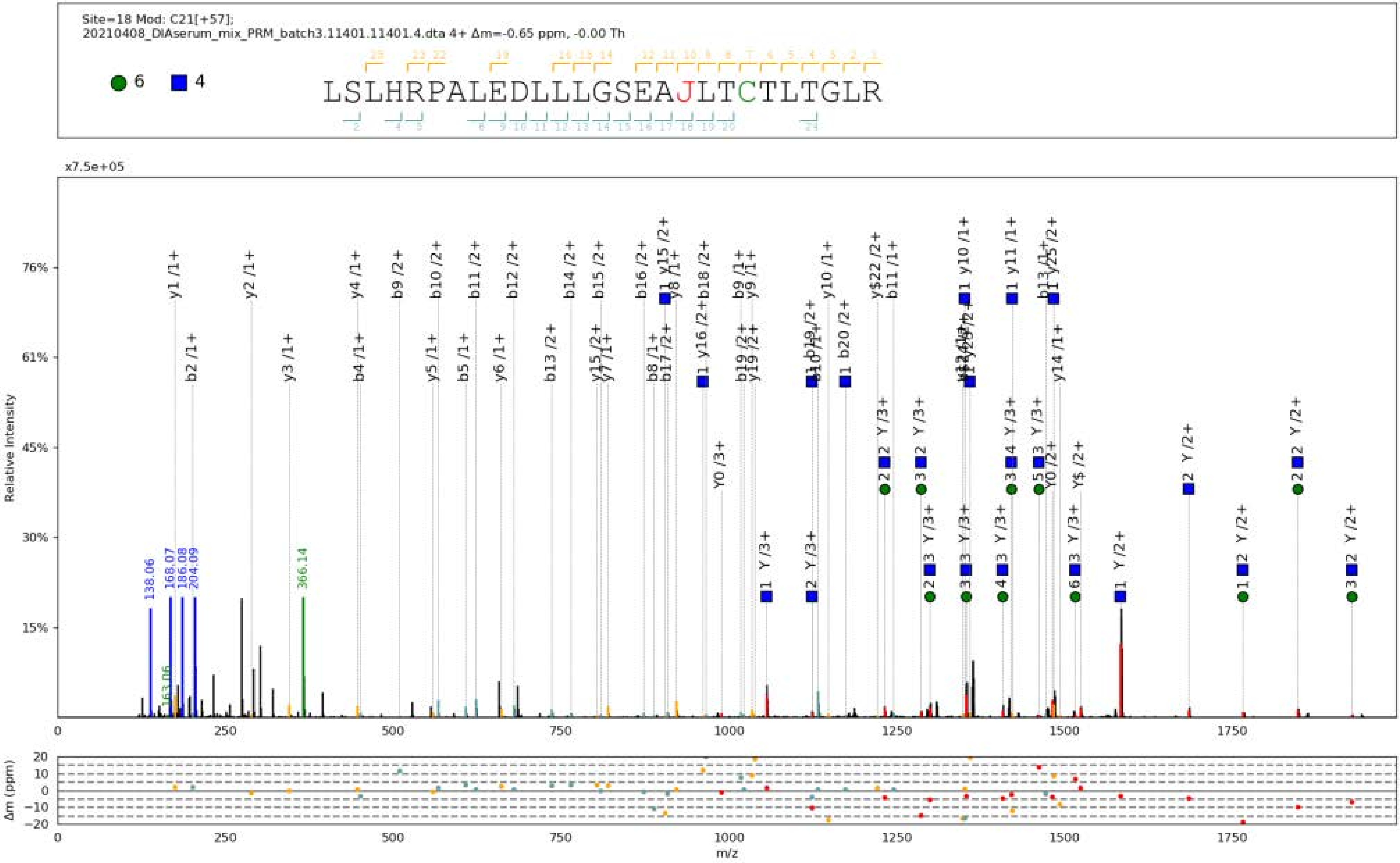
Site=18 Mod: C21[+57];
20210408_DIAserum_mix_PRM_batch3.11401.11401.4.dta 4+ Δm=-0.65 ppm, -0.00 Th
6
4
LSLHRPALEDLLLGSEAJLTCTLTGLR
x7.5e+05
Relative Intensity
76%
61%
45%
30%
15%
y1 /1+
b2 /1+
y2 /1+
y3 /1+
y4 /1+
b4 /1+
b9 /2+
b10 /2+
y5 /1+
b5 /1+
b11 /2+
y6 /1+
b12 /2+
b13 /2+
b14 /2+
b15 /2+
b16 /2+
b8 /1+
b17 /2+
y16 /2+
b18 /2+
b9 /1+
y9 /1+
b19 /2+
y19 /2+
y10 /1+
b20 /2+
b11 /1+
y10 /1+
y11 /1+
y14 /1+
138.06
168.07
186.08
204.09
163.06
366.14
Δm (ppm)
m/z

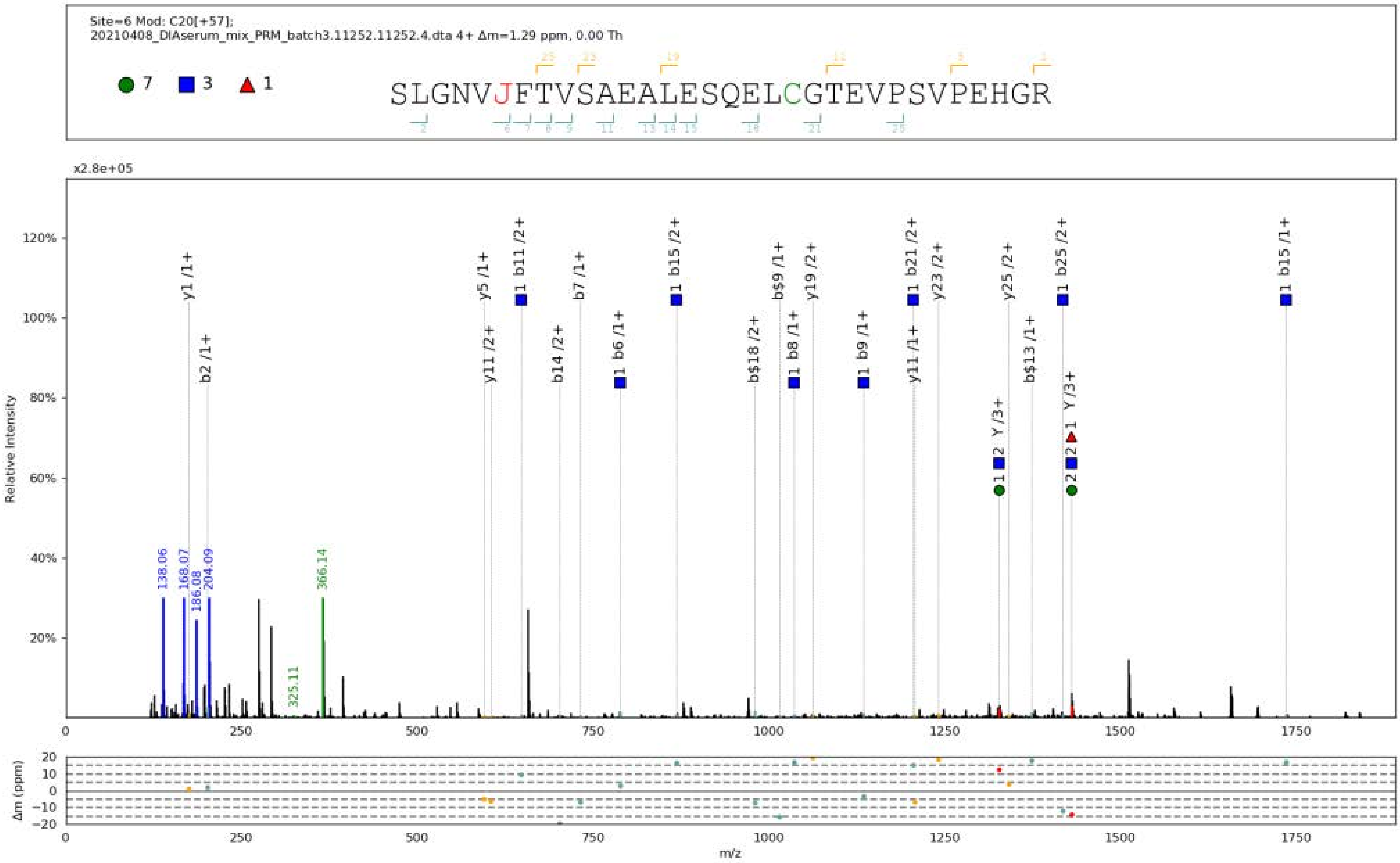

Site=6 Mod: C20[+57];
20210408_DIAserum_mix_PRM_batch3.11252.11252.4.dta 4+ Δm=1.29 ppm, 0.00 Th
7
3
1
SLGNVJFTVSAEALESQELCGTEVPSVPEHGR
x2.8e+05
Relative Intensity
m/z
Δm (ppm)
y1 /1+
b2 /1+
y5 /1+
y11 /2+
1 b11 /2+
b14 /2+
b7 /1+
1 b6 /1+
1 b15 /2+
b$18 /2+
b$9 /1+
1 b8 /1+
y19 /2+
1 b9 /1+
1 b21 /2+
y11 /1+
y23 /2+
y25 /2+
b$13 /1+
1 b25 /2+
1 b15 /1+
Y /3+
138.06
168.07
186.08
204.09
366.14
325.11

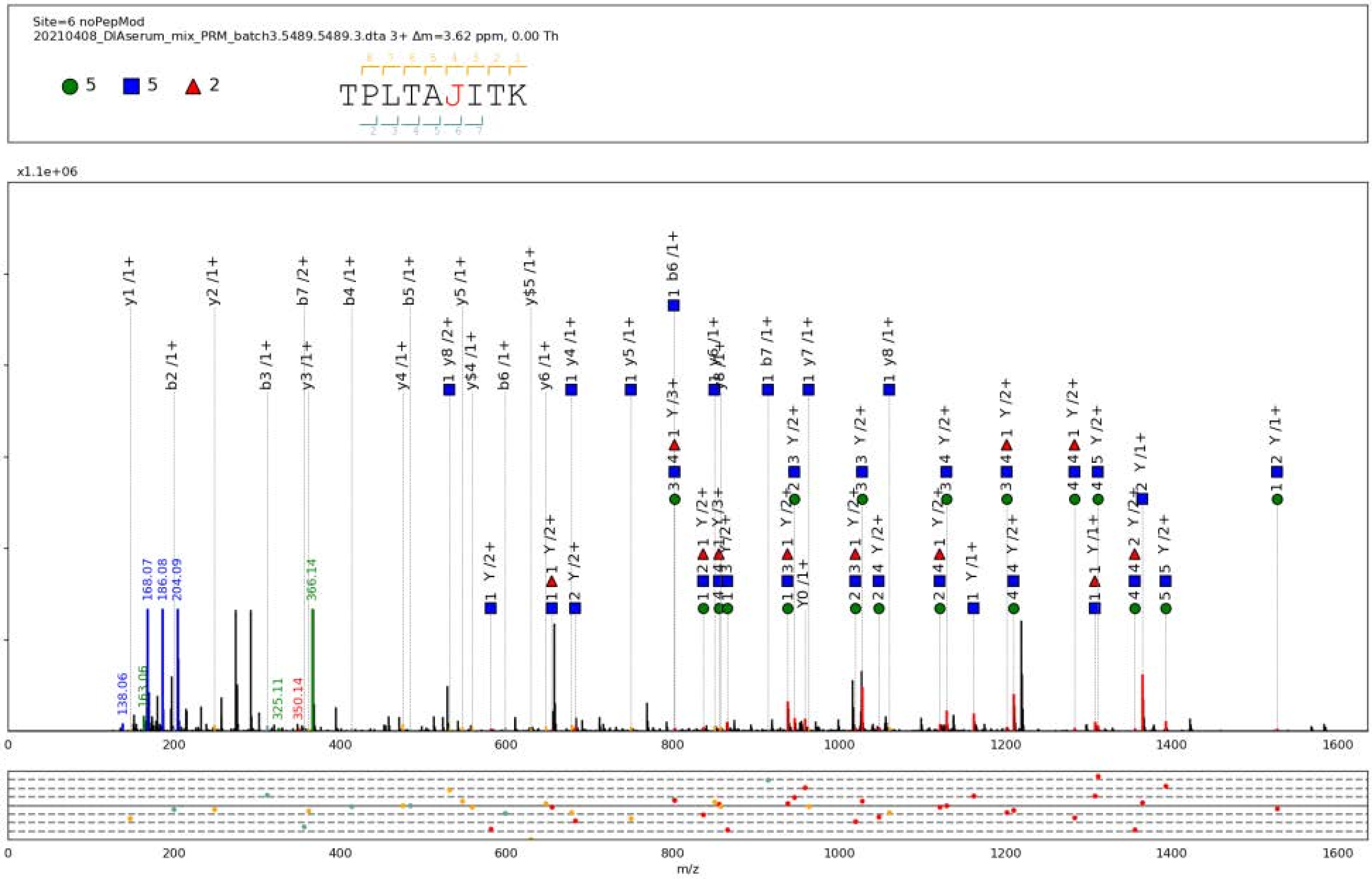

Site=6 noPepMod
20210408_DIAserum_mix_PRM_batch3.5489.5489.3.dta 3+ Δm=3.62 ppm, 0.00 Th
5 5 2
TPLTAJITK
x1.1e+06
Relative Intensity
m/z
Δm (ppm)

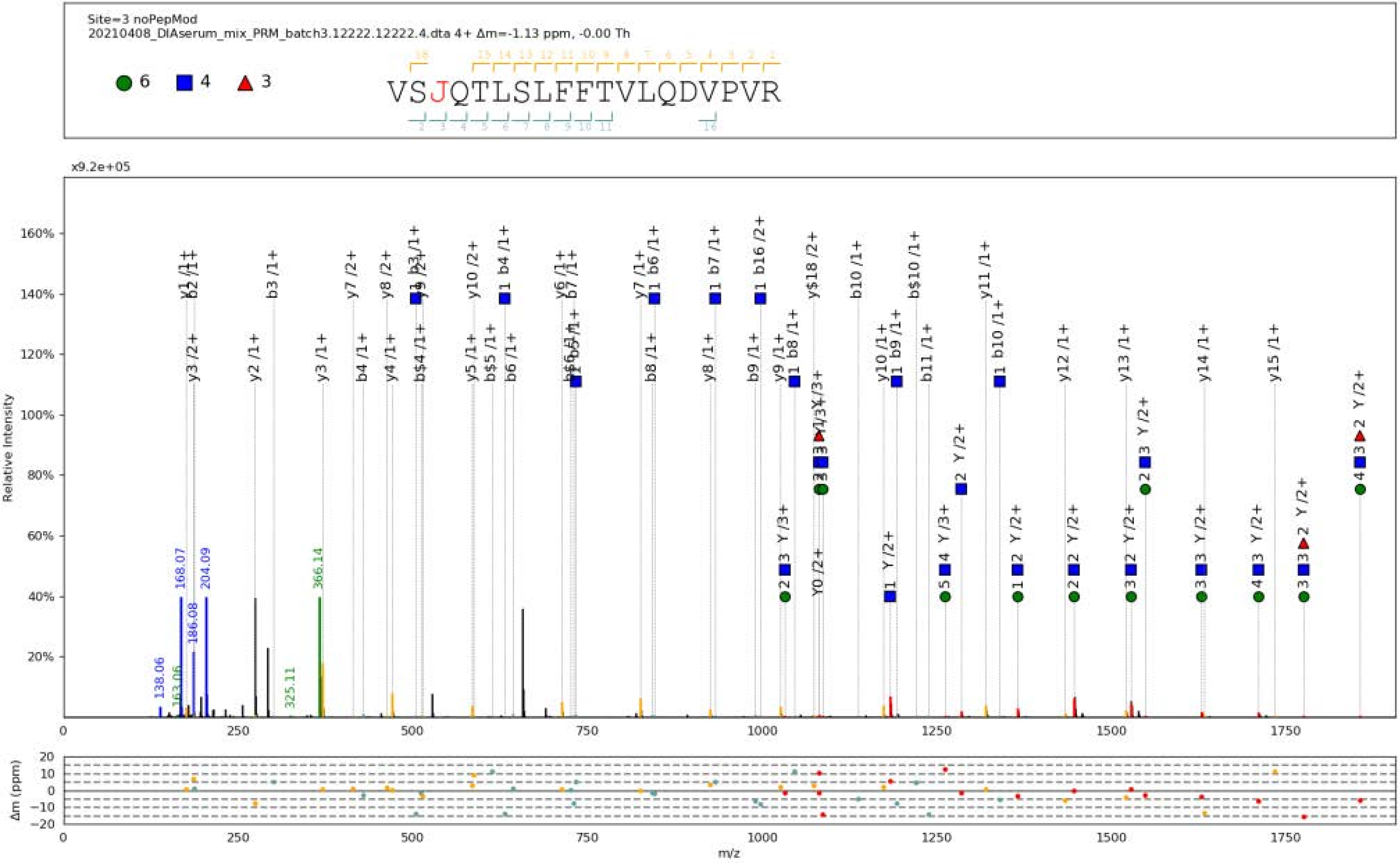

Site=3 noPepMod
20210408_DIAserum_mix_PRM_batch3.12222.12222.4.dta 4+ Δm=-1.13 ppm, -0.00 Th
6
4
3
VSJQTLSLFFTVLQDVPVR
x9.2e+05
Relative Intensity
Δm (ppm)
m/z

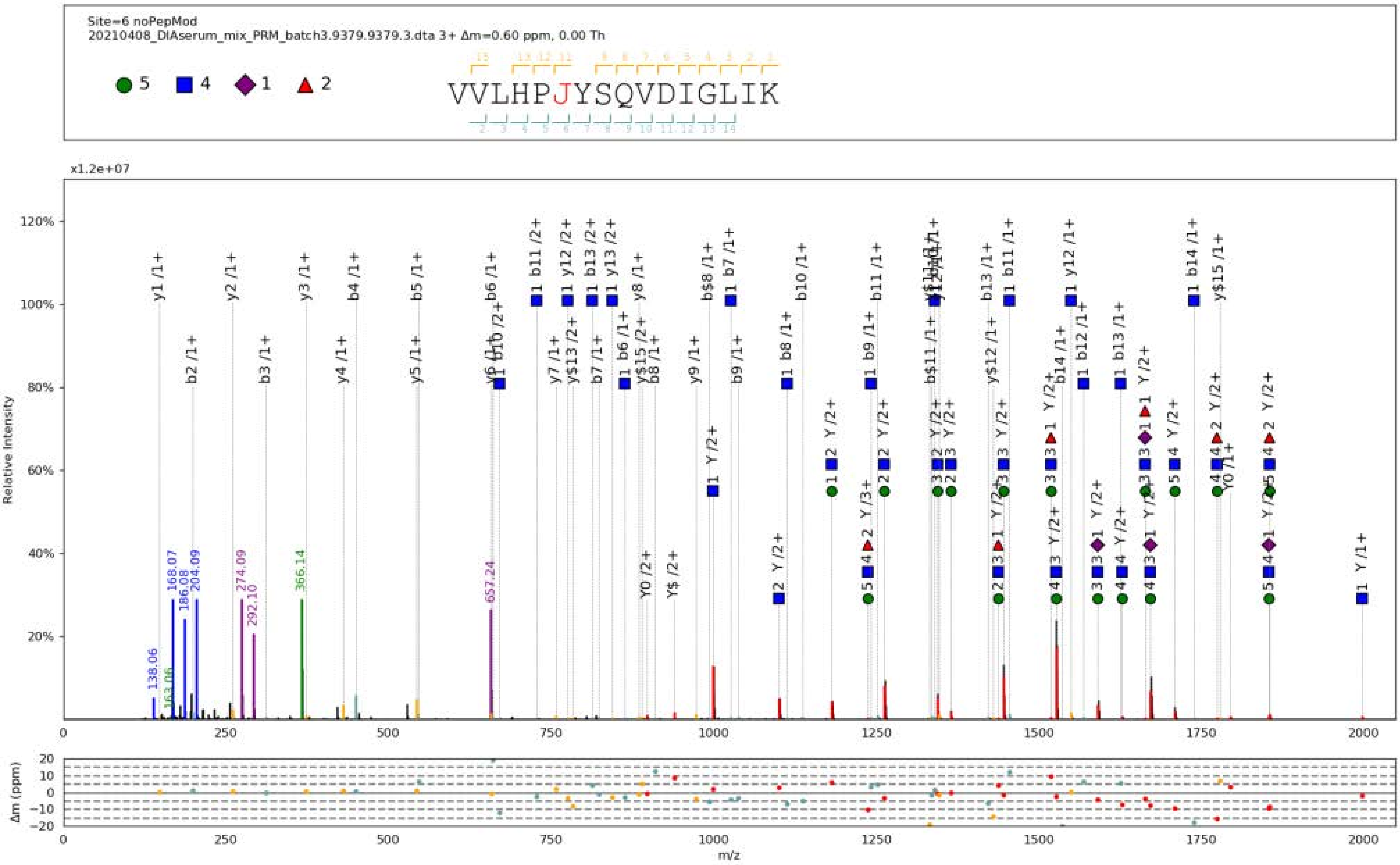

Site=6 noPepMod
20210408_DIAserum_mix_PRM_batch3.9379.9379.3.dta 3+ Δm=0.60 ppm, 0.00 Th
5
4
1
2
VVLHPJYSQVDIGLIK
x1.2e+07
Relative Intensity
m/z
Δm (ppm)

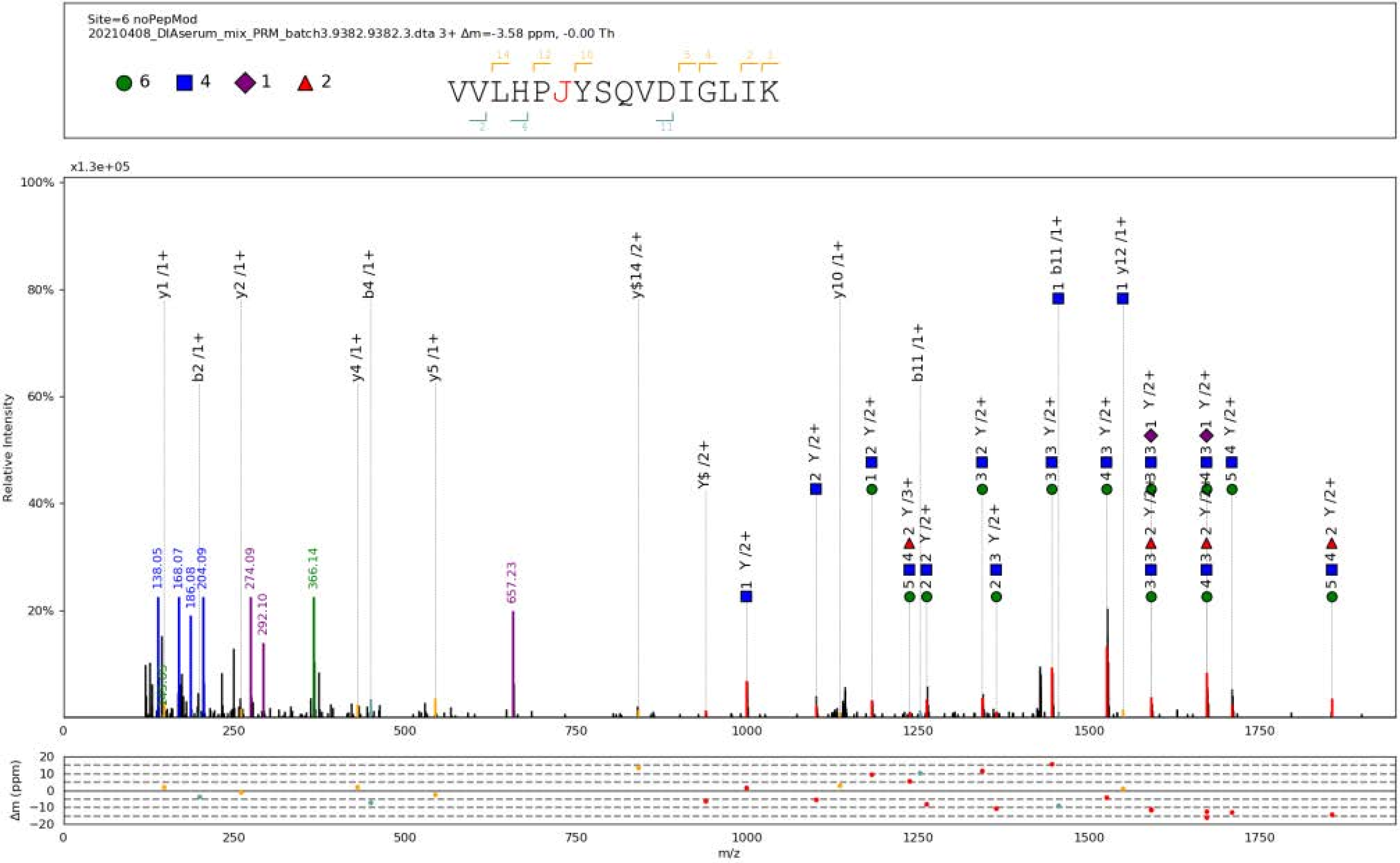
Site=6 noPepMod
20210408_DIAserum_mix_PRM_batch3.9382.9382.3.dta 3+ Δm=-3.58 ppm, -0.00 Th
6 4 1 2
VVLHPJYSQVDIGLIK
x1.3e+05
Relative Intensity
Δm (ppm)
m/z
y1 /1+
b2 /1+
y2 /1+
y4 /1+
b4 /1+
y5 /1+
y$14 /2+
Y$ /2+
y10 /1+
b11 /1+
b11 /1+
y12 /1+
138.05
168.07
186.08
204.09
274.09
292.10
366.14
657.23

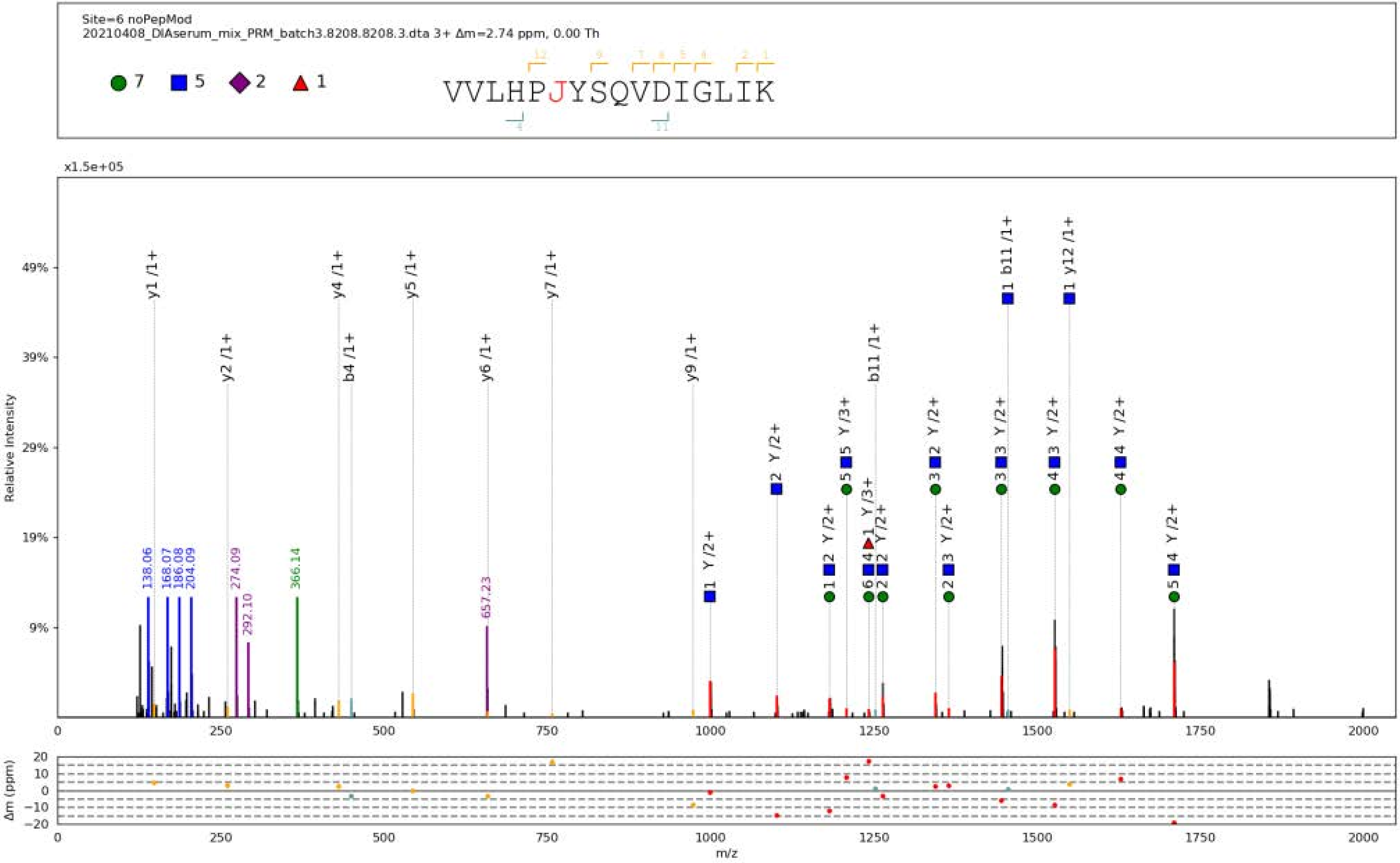

Site=6 noPepMod
20210408_DIAserum_mix_PRM_batch3.8208.8208.3.dta 3+ Δm=2.74 ppm, 0.00 Th
VVLHPJYSQVDIGLIK
x1.5e+05
Relative Intensity
m/z
Δm (ppm)

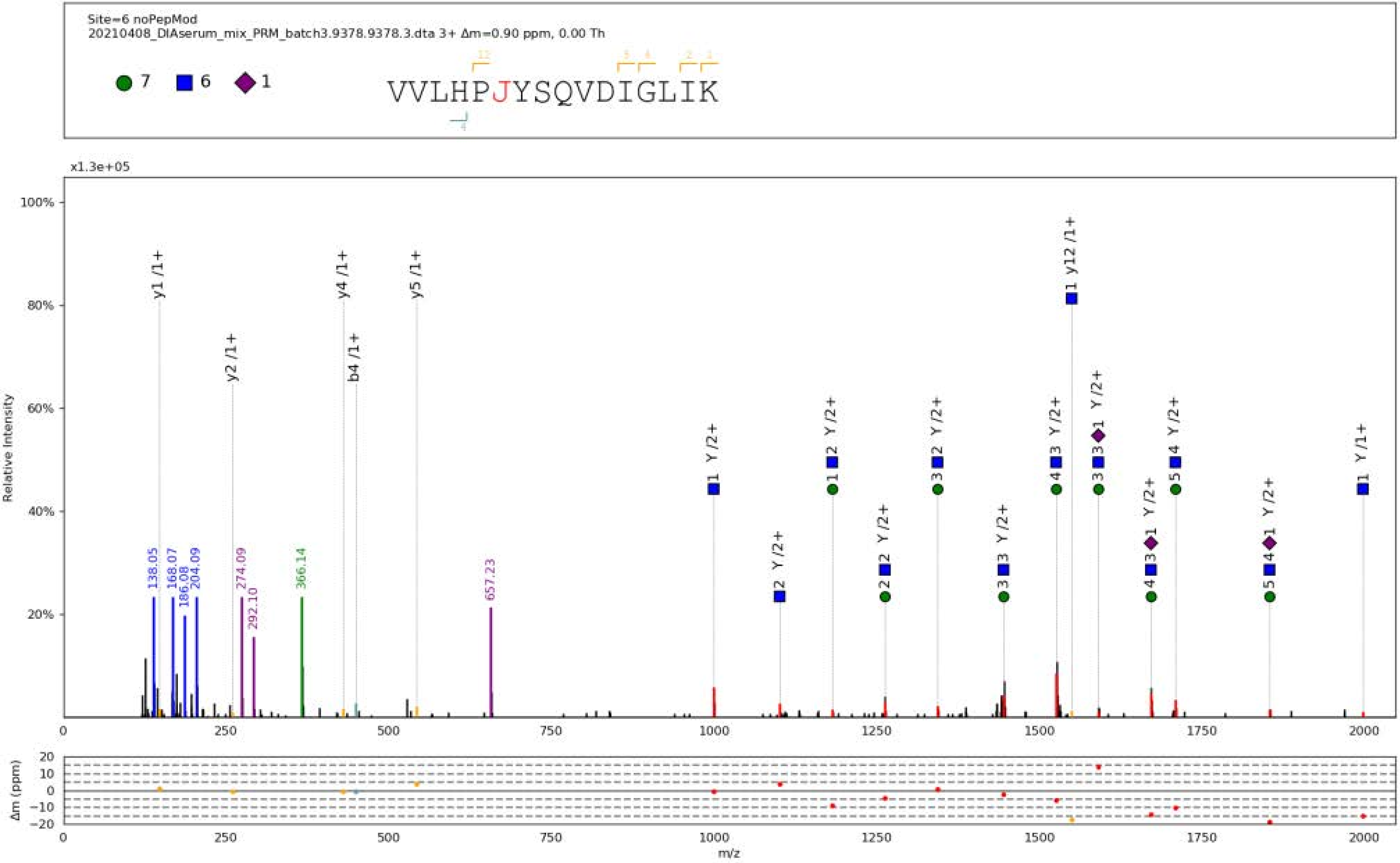

Site=6 noPepMod
20210408_DIAserum_mix_PRM_batch3.9378.9378.3.dta 3+ Δm=0.90 ppm, 0.00 Th
7 6 1
VVLHPJYSQVDIGLIK
x1.3e+05
Relative Intensity
y1 /1+
y2 /1+
y4 /1+
b4 /1+
y5 /1+
y12 /1+
138.05
168.07
186.08
204.09
274.09
292.10
366.14
657.23
Y /2+
Y /1+
m/z
Δm (ppm)

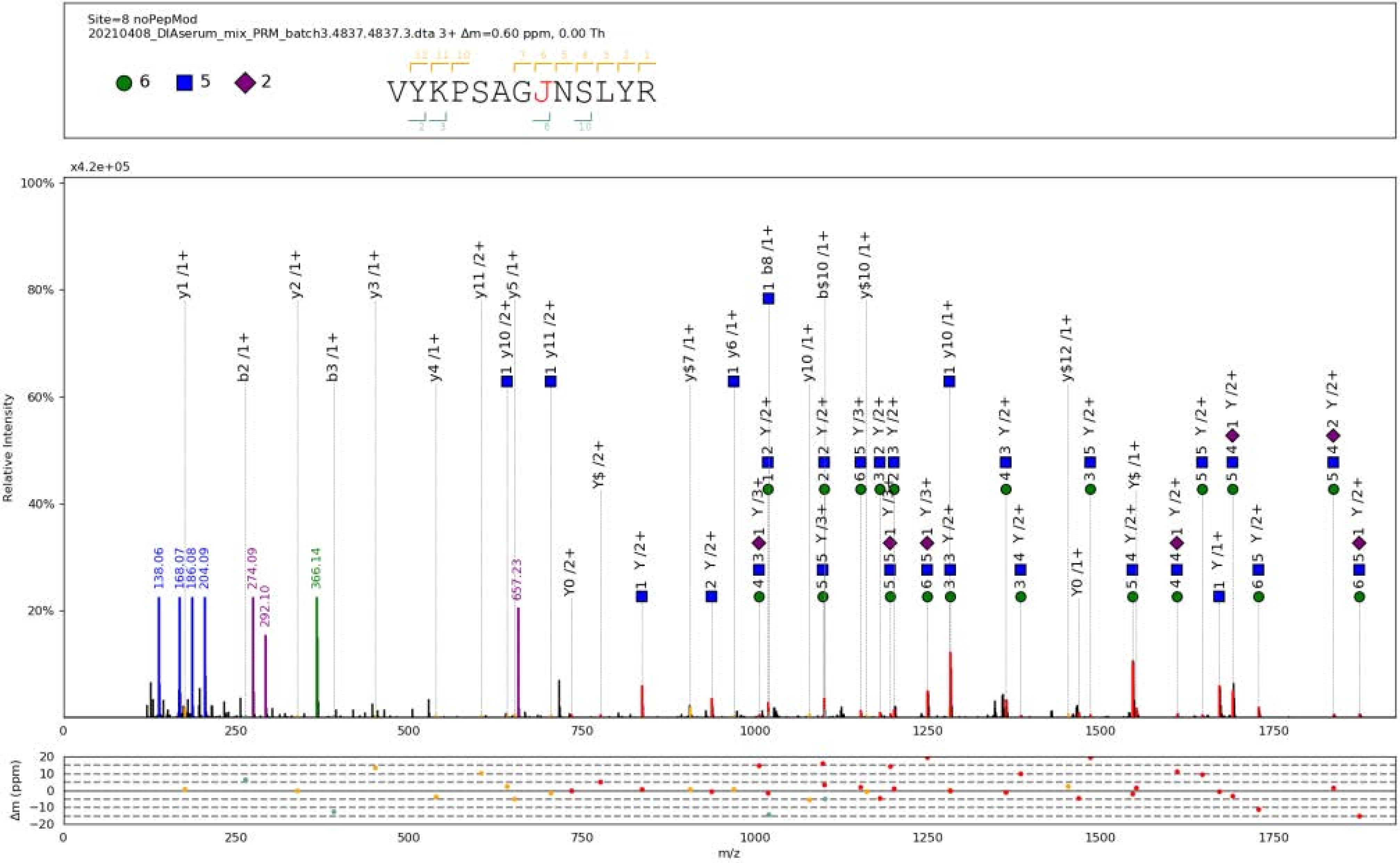
Site=8 noPepMod
20210408_DIAserum_mix_PRM_batch3.4837.4837.3.dta 3+ Δm=0.60 ppm, 0.00 Th
6
5
2
VYKPSAGJNSLYR
x4.2e+05
Relative Intensity
138.06
168.07
186.08
204.09
274.09
292.10
366.14
657.23
y1 /1+
b2 /1+
y2 /1+
b3 /1+
y3 /1+
y4 /1+
y11 /2+
y10 /2+
y5 /1+
y11 /2+
Y0 /2+
Y$ /2+
y$7 /1+
y6 /1+
b8 /1+
y10 /1+
b$10 /1+
y$10 /1+
y10 /1+
y$12 /1+
Y0 /1+
Y$ /1+
Y /1+
m/z
Δm (ppm)

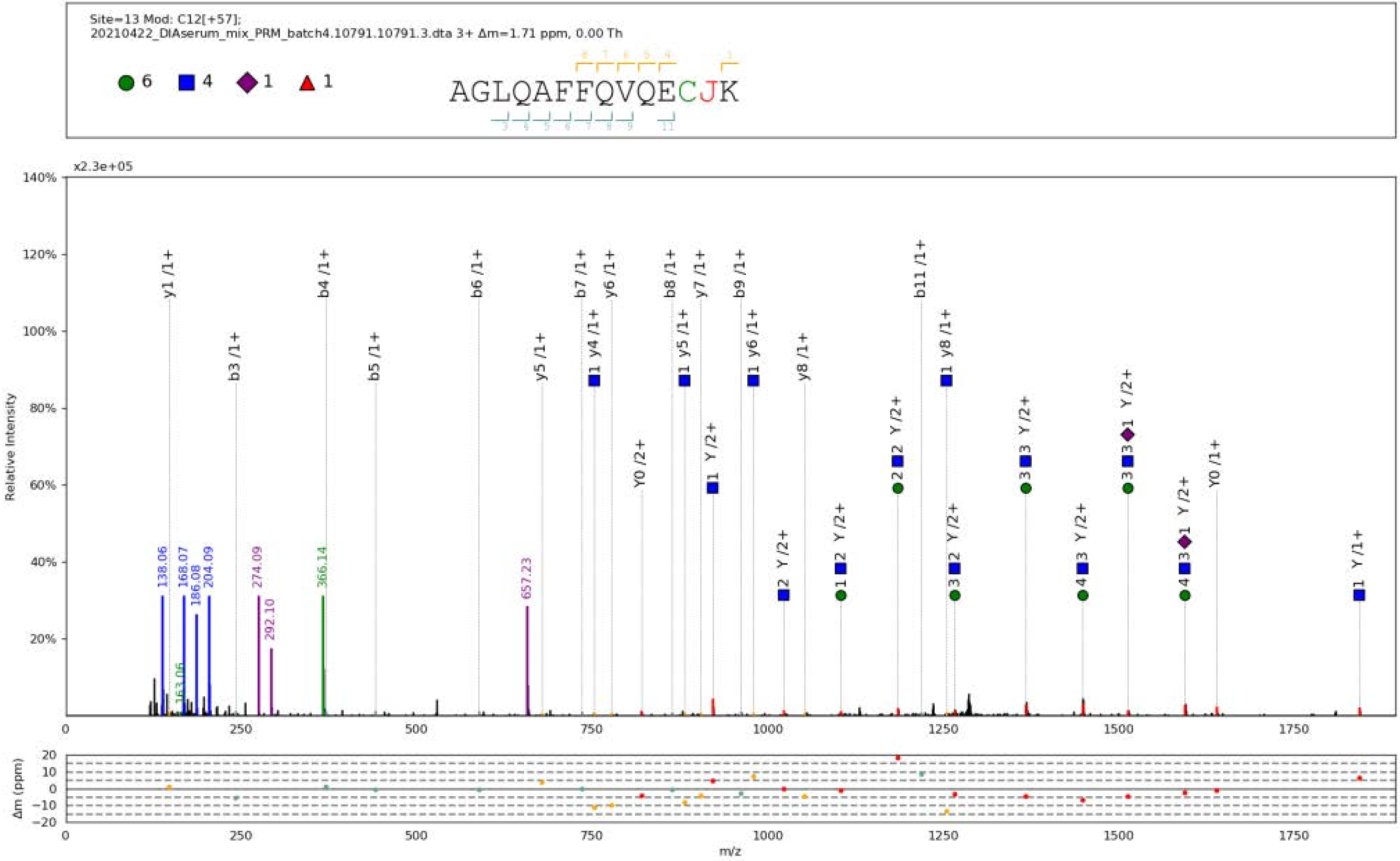

Site=13 Mod: C12[+57];
20210422_DIAserum_mix_PRM_batch4.10791.10791.3.dta 3+ Δm=1.71 ppm, 0.00 Th
6 4 1 1
AGLQAFFQVQECJK
x2.3e+05
Relative Intensity
m/z
Δm (ppm)
y1 /1+
b3 /1+
b4 /1+
b5 /1+
b6 /1+
y5 /1+
b7 /1+
y4 /1+
y6 /1+
Y0 /2+
b8 /1+
y5 /1+
y7 /1+
b9 /1+
y6 /1+
y8 /1+
b11 /1+
y8 /1+
Y0 /1+
138.06
168.07
186.08
204.09
274.09
292.10
366.14
163.06
657.23

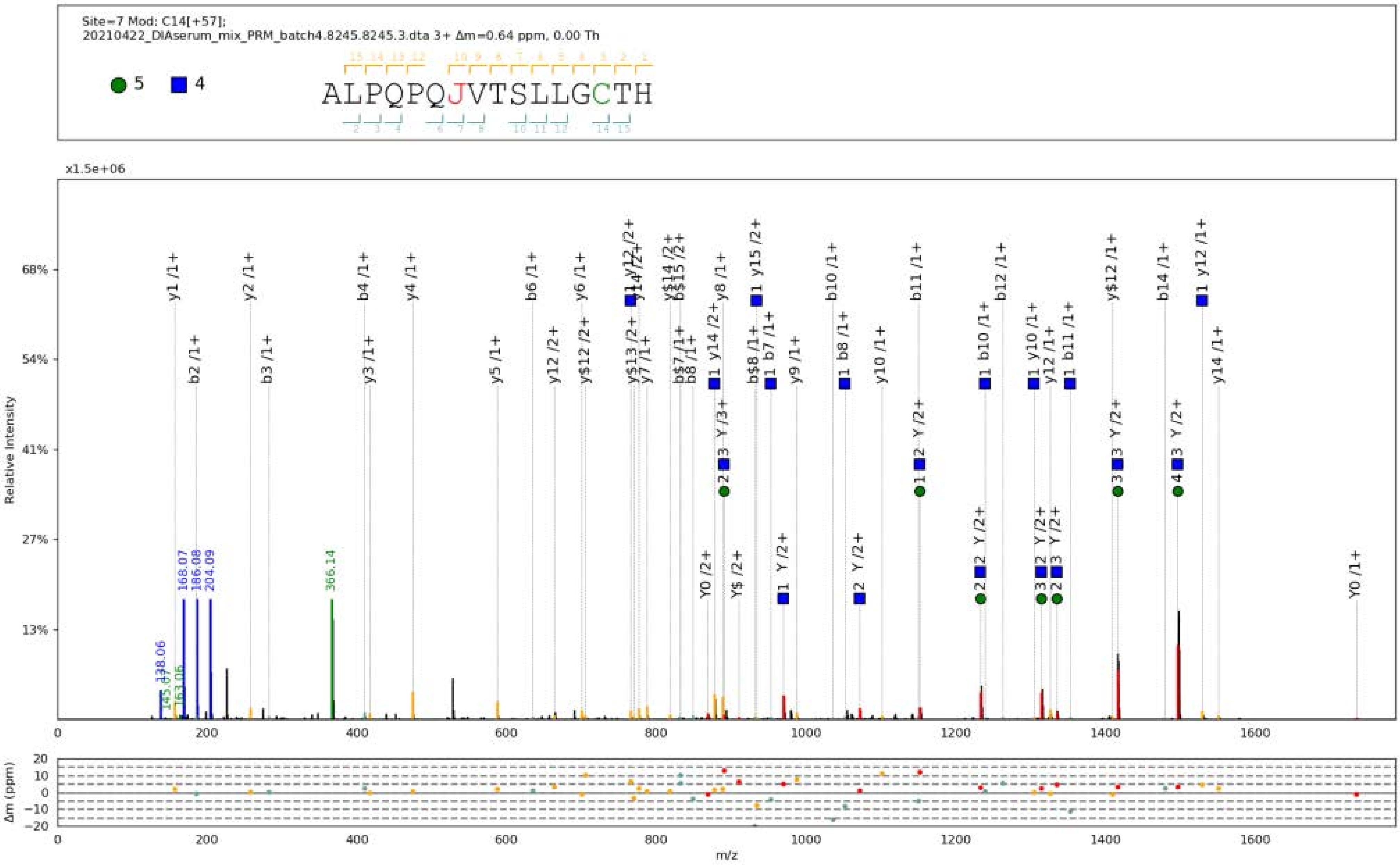
Site=7 Mod: C14[+57];
20210422_DIAserum_mix_PRM_batch4.8245.8245.3.dta 3+ Δm=0.64 ppm, 0.00 Th
ALPQPQJVTSLLGCTH
x1.5e+06
Relative Intensity
m/z
Δm (ppm)

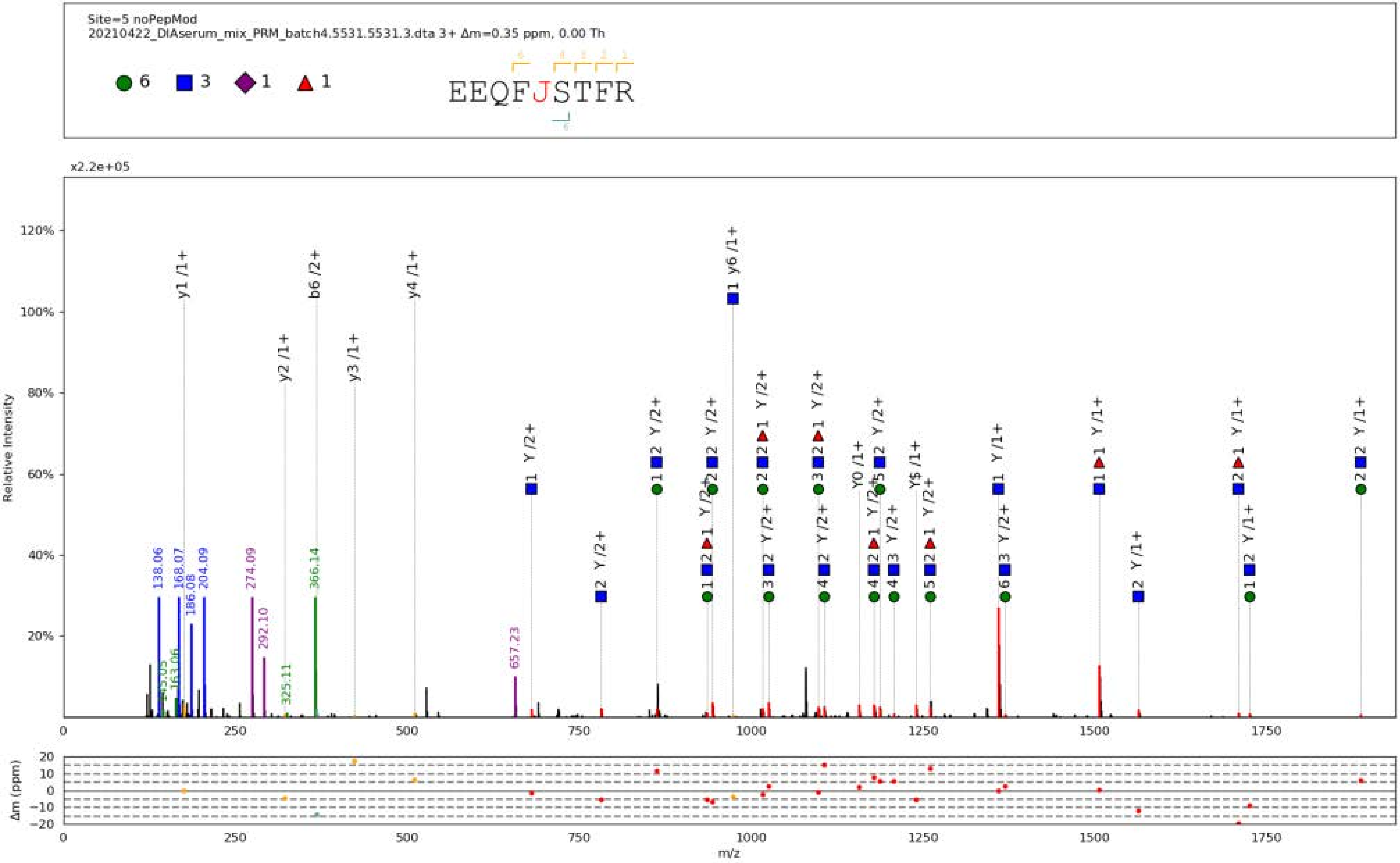
Site=5 noPepMod
20210422_DIAserum_mix_PRM_batch4.5531.5531.3.dta 3+ Δm=0.35 ppm, 0.00 Th
6
3
1
1
EEQFJSTFR
x2.2e+05
Relative Intensity
y1 /1+
b6 /2+
y4 /1+
y2 /1+
y3 /1+
y6 /1+
138.06
168.07
186.08
204.09
274.09
292.10
366.14
325.11
657.23
m/z
Δm (ppm)

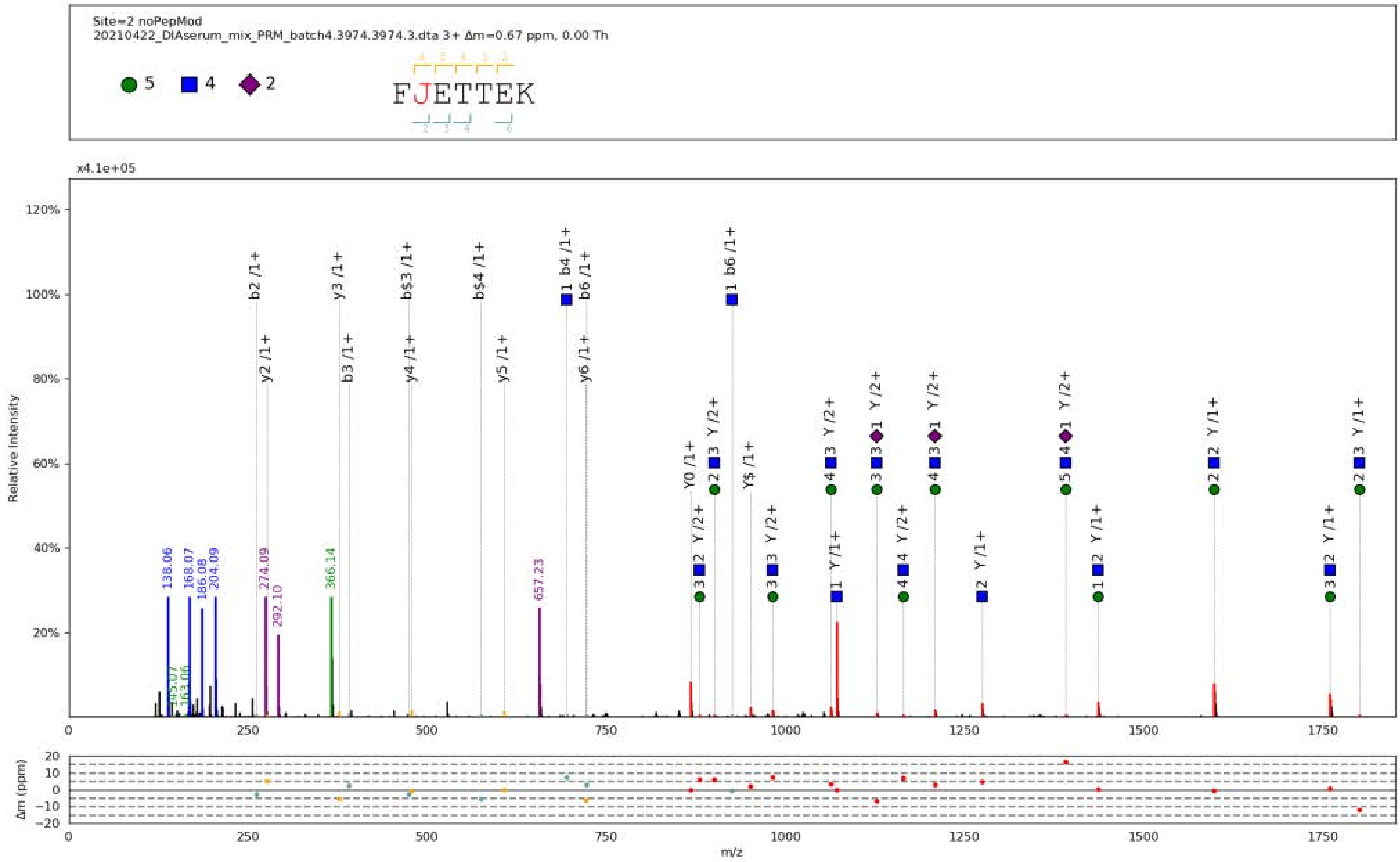

Site=2 noPepMod
20210422_DIAserum_mix_PRM_batch4.3974.3974.3.dta 3+ Δm=0.67 ppm, 0.00 Th
5
4
2
FJETTEK
x4.1e+05
Relative Intensity
b2 /1+
y2 /1+
y3 /1+
b3 /1+
b$3 /1+
y4 /1+
b$4 /1+
y5 /1+
1 b4 /1+
b6 /1+
y6 /1+
1 b6 /1+
Y0 /1+
Y$ /1+
138.06
168.07
186.08
204.09
274.09
292.10
366.14
657.23
m/z
Δm (ppm)

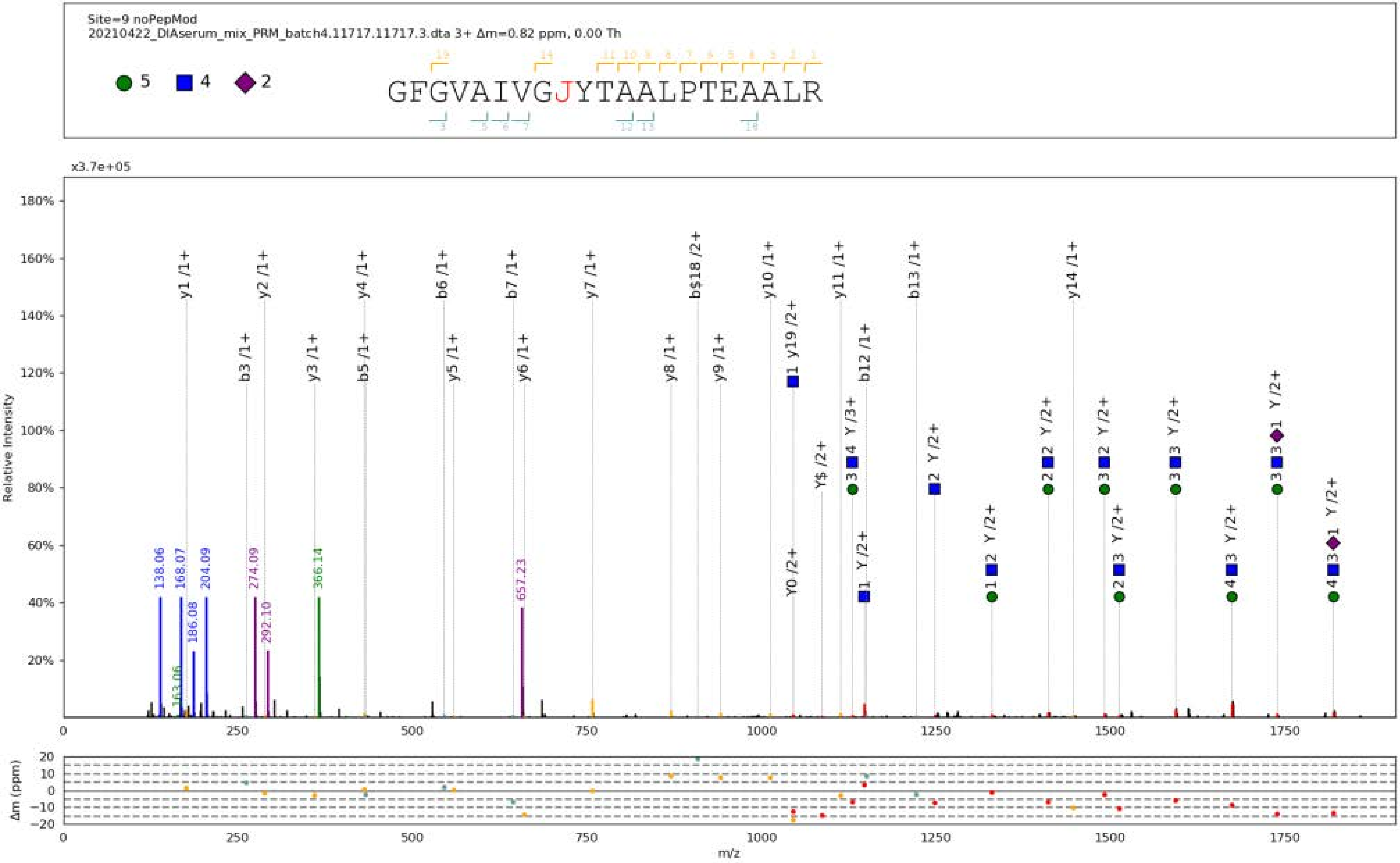
Site=9 noPepMod
20210422_DIAserum_mix_PRM_batch4.11717.11717.3.dta 3+ Δm=0.82 ppm, 0.00 Th
5
4
2
GFGVAIVGJYTAALPTEAALR
x3.7e+05
Relative Intensity
y1 /1+
y2 /1+
y4 /1+
b6 /1+
b7 /1+
y7 /1+
b$18 /2+
y10 /1+
y11 /1+
b13 /1+
y14 /1+
b3 /1+
y3 /1+
b5 /1+
y5 /1+
y6 /1+
y8 /1+
y9 /1+
1 y19 /2+
b12 /1+
Y$ /2+
Y0 /2+
138.06
168.07
204.09
186.08
163.06
274.09
292.10
366.14
657.23
m/z
Δm (ppm)

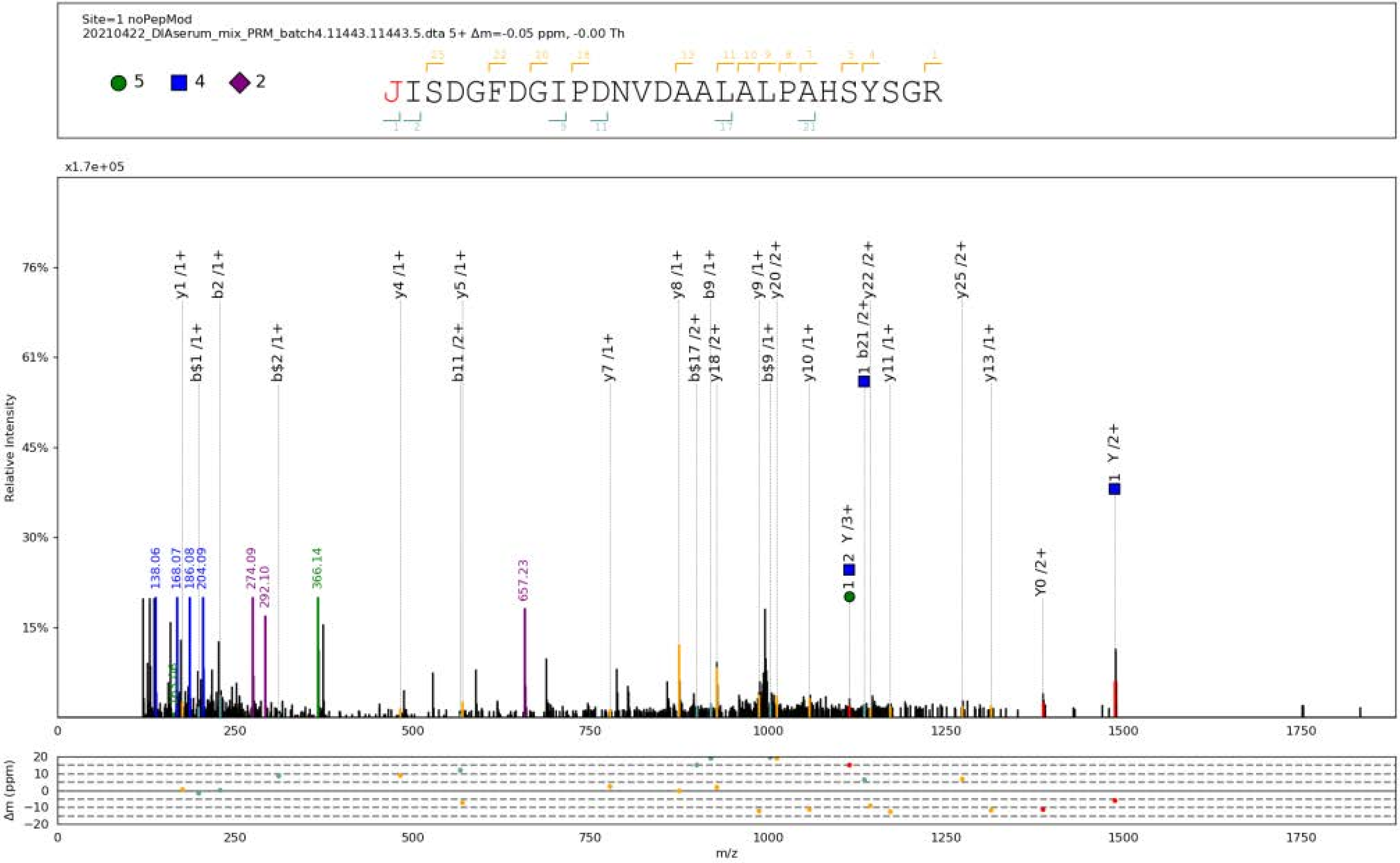

Site=1 noPepMod
20210422_DIAserum_mix_PRM_batch4.11443.11443.5.dta 5+ Δm=-0.05 ppm, -0.00 Th
5
4
2
JISDGFDGIPDNVDAALALPAHSYSGR
x1.7e+05
Relative Intensity
m/z
Δm (ppm)
y1 /1+
b2 /1+
b$1 /1+
b$2 /1+
y4 /1+
y5 /1+
b11 /2+
y7 /1+
y8 /1+
b$17 /2+
b9 /1+
y18 /2+
y9 /1+
y20 /2+
b$9 /1+
y10 /1+
1 Y /3+
b21 /2+
y22 /2+
y11 /1+
y25 /2+
y13 /1+
Y0 /2+
1 Y /2+
138.06
168.07
186.08
204.09
274.09
292.10
366.14
657.23

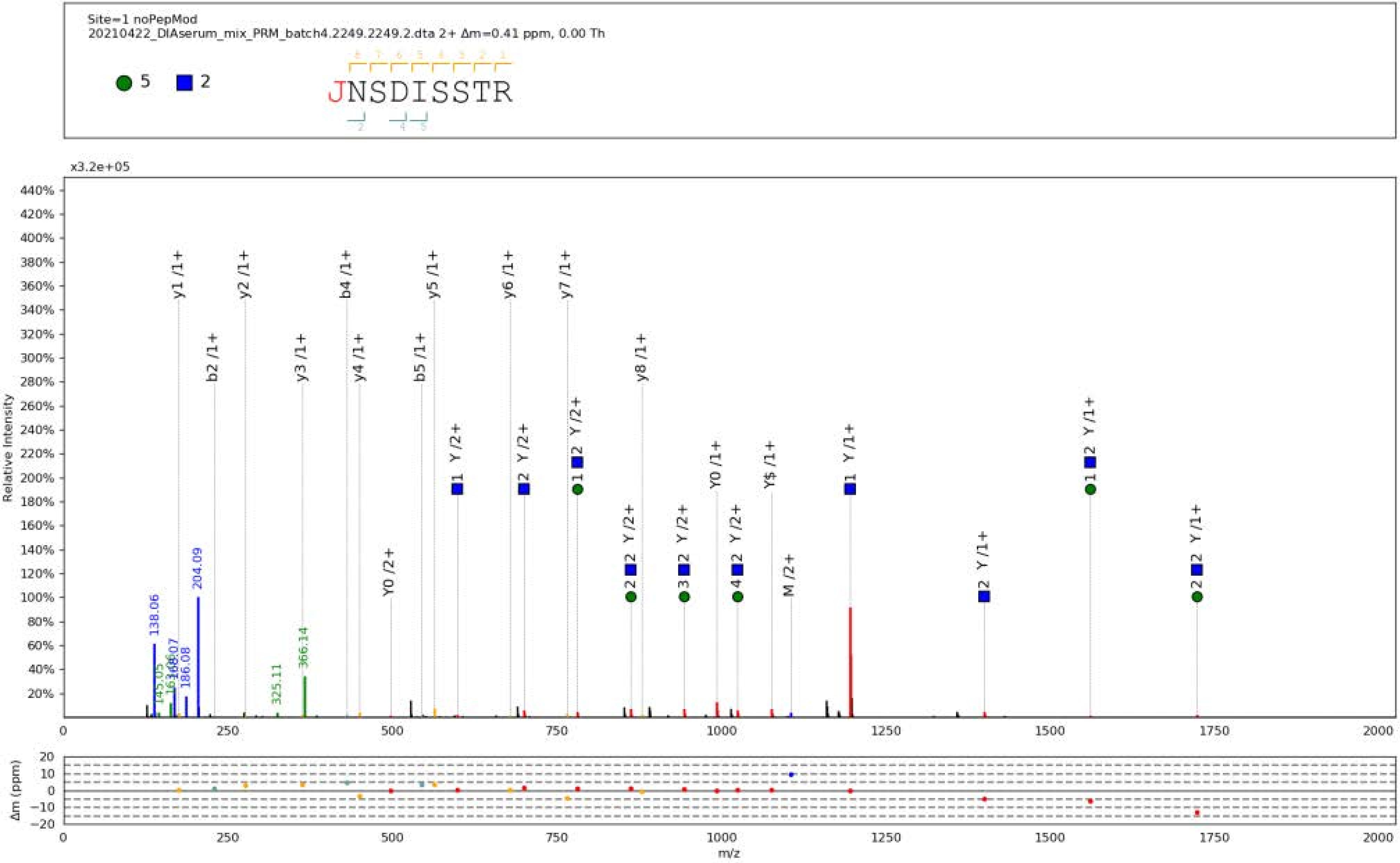

Site=1 noPepMod
20210422_DIAserum_mix_PRM_batch4.2249.2249.2.dta 2+ Δm=0.41 ppm, 0.00 Th
5
2
JNSDISSTR
x3.2e+05
Relative Intensity
m/z
Δm (ppm)

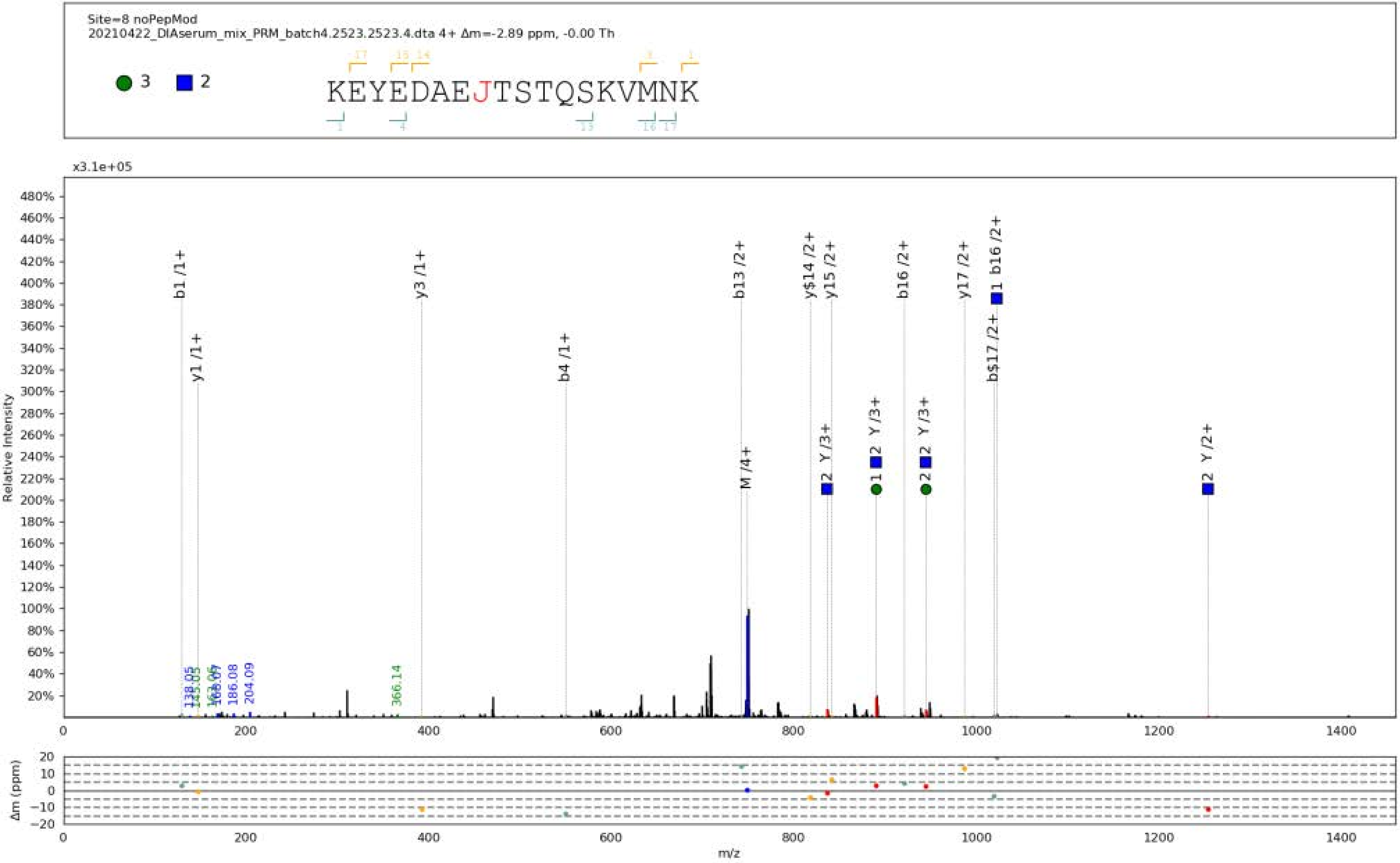

Site=8 noPepMod
20210422_DIAserum_mix_PRM_batch4.2523.2523.4.dta 4+ Δm=-2.89 ppm, -0.00 Th
3
2
KEYEDAEJTSTQSKVMNK
x3.1e+05
Relative Intensity
b1 /1+
y1 /1+
y3 /1+
b4 /1+
b13 /2+
M /4+
y$14 /2+
y15 /2+
2 Y /3+
1 2 Y /3+
b16 /2+
2 2 Y /3+
y17 /2+
b$17 /2+
1 b16 /2+
2 Y /2+
138.05
145.05
163.06
168.07
186.08
204.09
366.14
m/z
Δm (ppm)

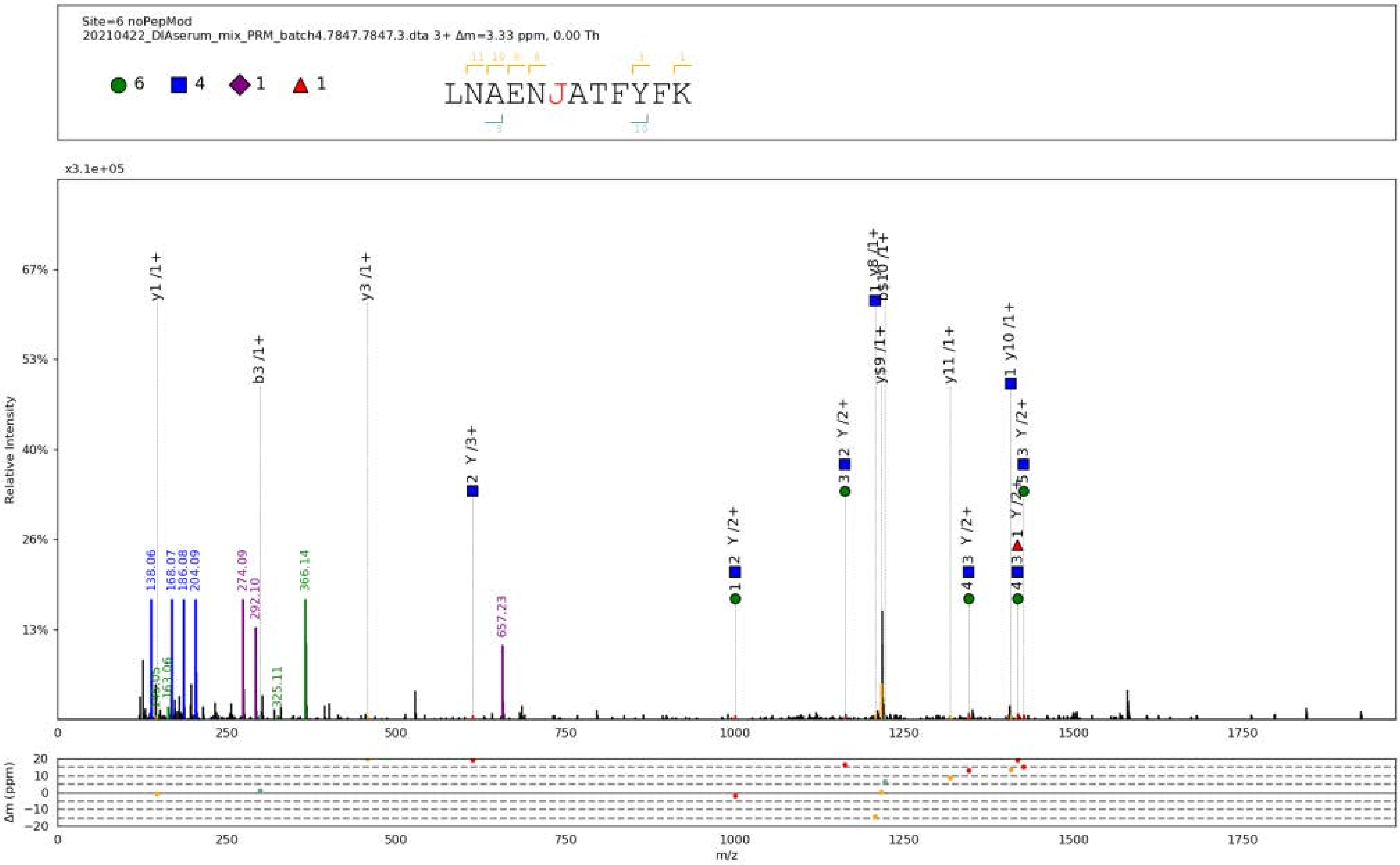

Site=6 noPepMod
20210422_DIAserum_mix_PRM_batch4.7847.7847.3.dta 3+ Δm=3.33 ppm, 0.00 Th
LNAENJATFYFK
x3.1e+05
Relative Intensity
m/z
Δm (ppm)

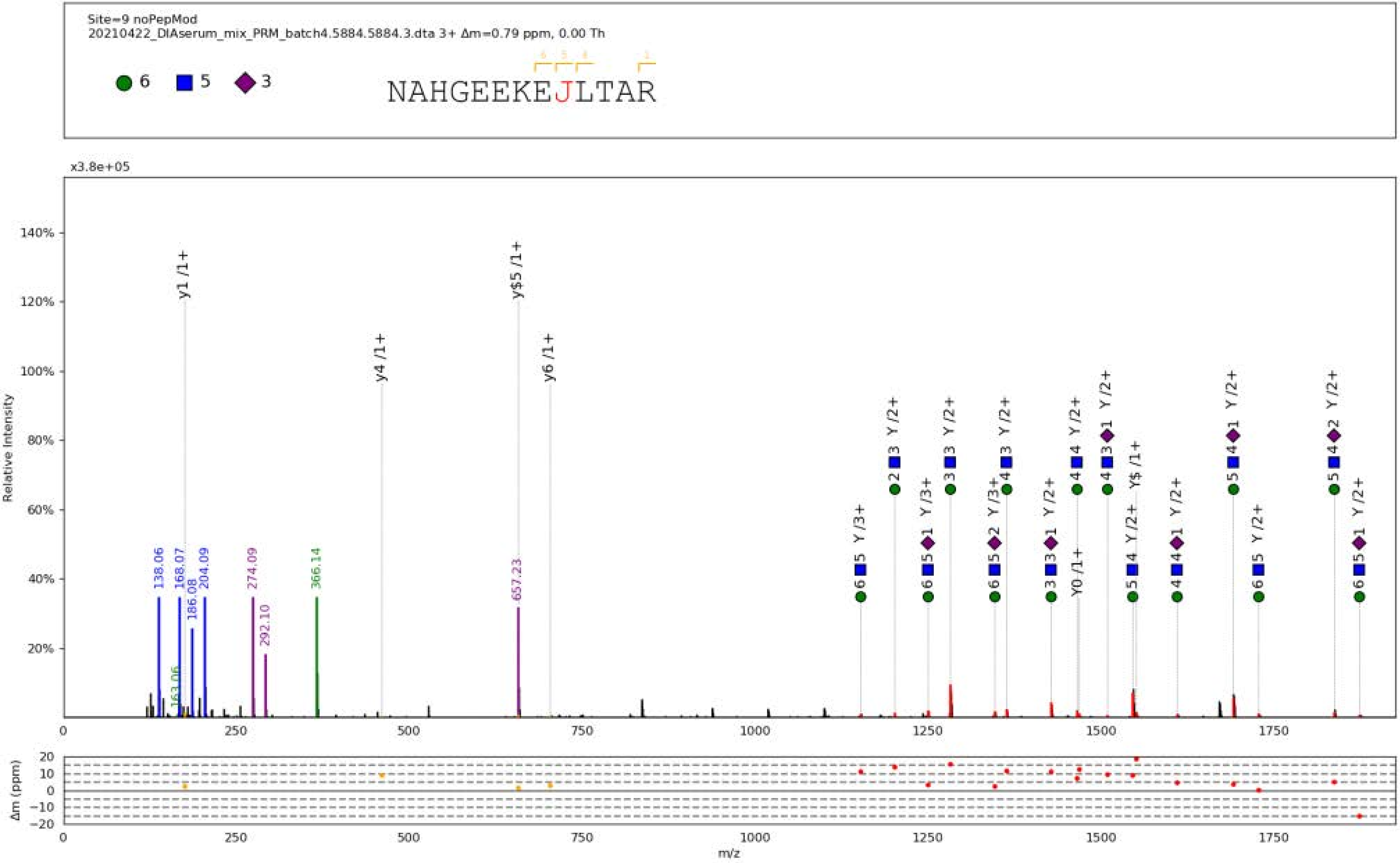

Site=9 noPepMod
20210422_DIAserum_mix_PRM_batch4.5884.5884.3.dta 3+ Δm=0.79 ppm, 0.00 Th
6
5
3
NAHGEEKEJLTAR
x3.8e+05
Relative Intensity
m/z
Δm (ppm)
y1 /1+
y$5 /1+
y4 /1+
y6 /1+
Y0 /1+
Y$ /1+
138.06
168.07
186.08
204.09
163.06
274.09
292.10
366.14
657.23

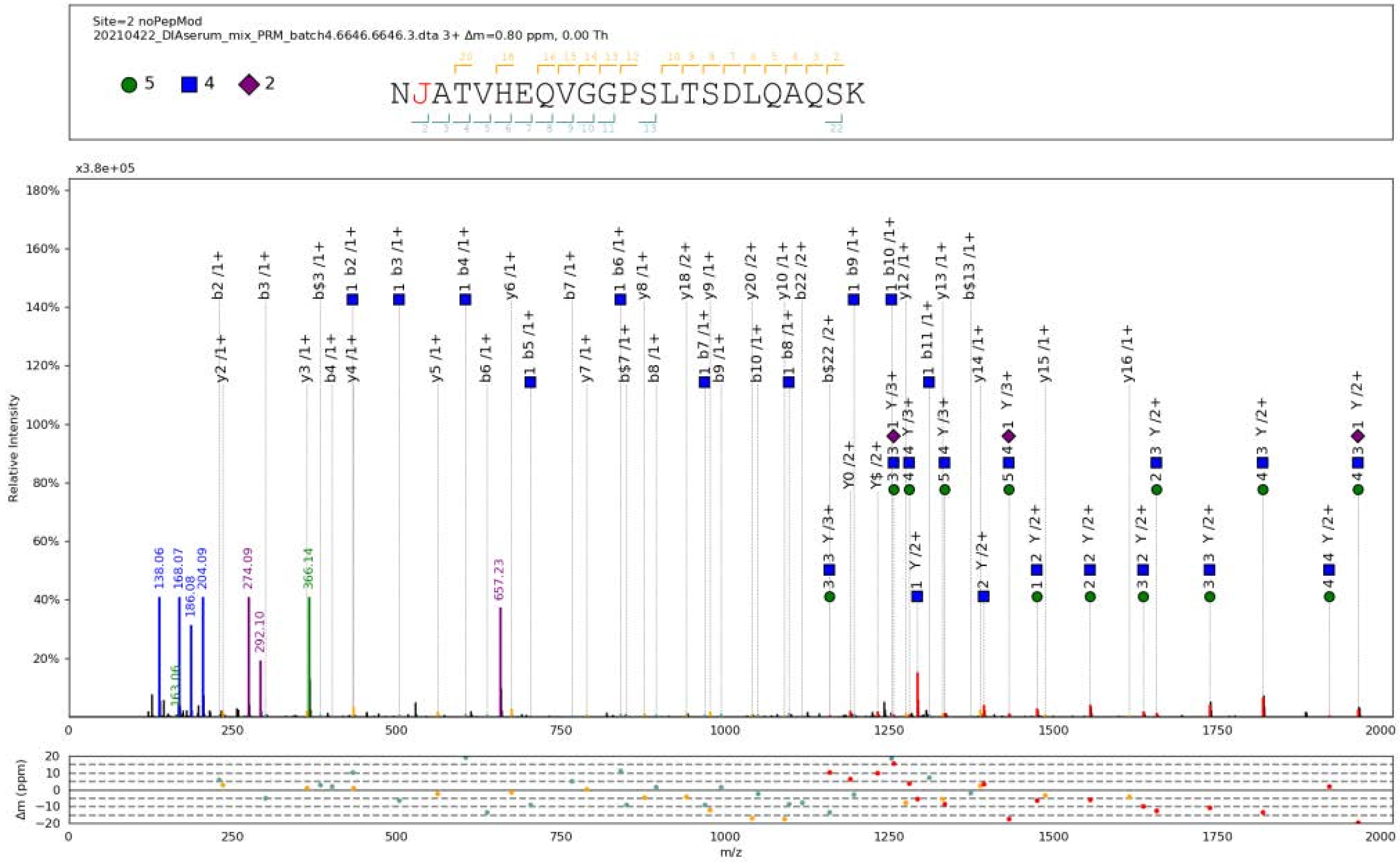

Site=2 noPepMod
20210422_DIAserum_mix_PRM_batch4.6646.6646.3.dta 3+ Δm=0.80 ppm, 0.00 Th
NJATVHEQVGGPSLTSDLQAQSK
x3.8e+05
Relative Intensity
Δm (ppm)
m/z

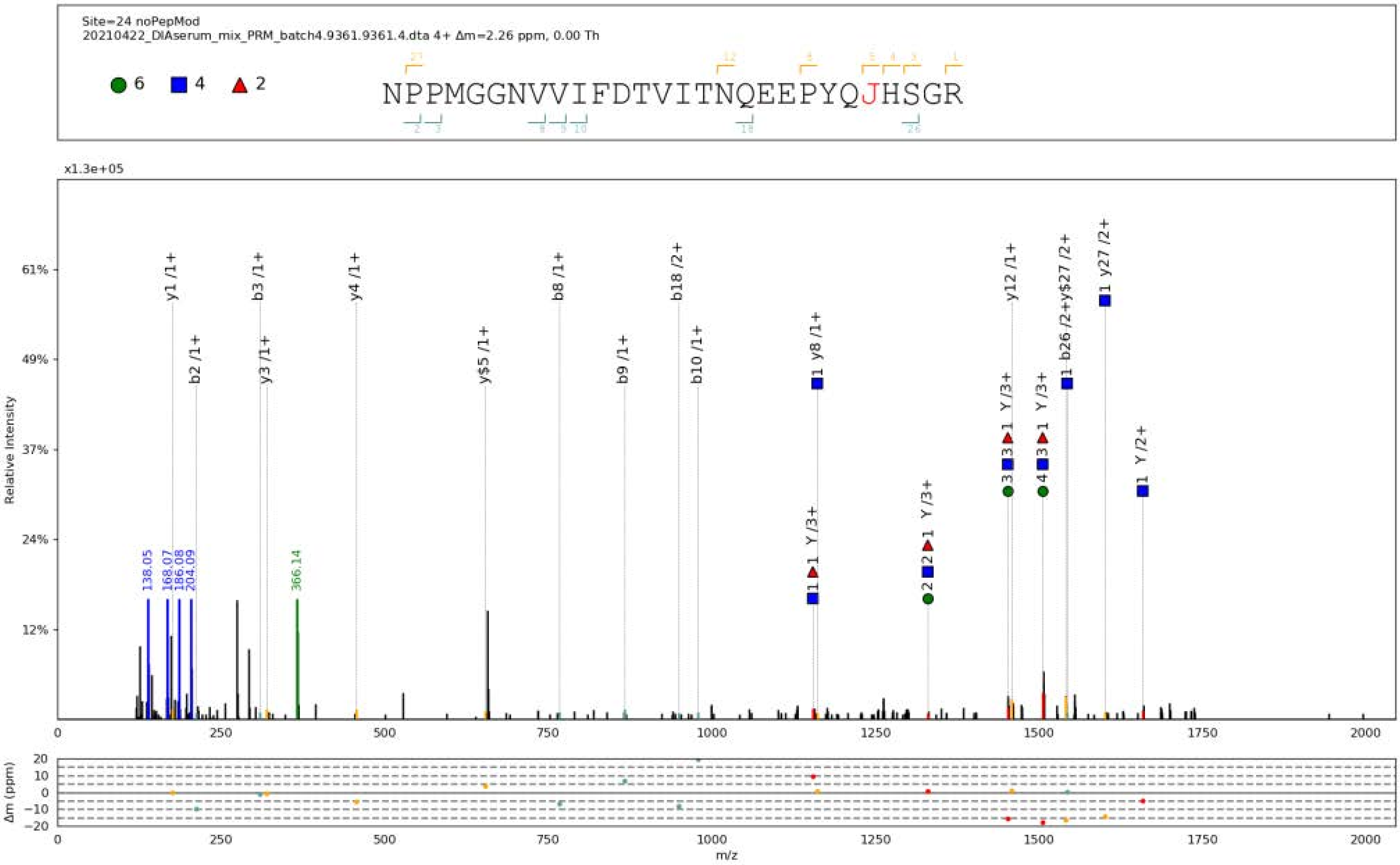
Site=24 noPepMod
20210422_DIAserum_mix_PRM_batch4.9361.9361.4.dta 4+ Δm=2.26 ppm, 0.00 Th
6
4
2
NPPMGGNVVIFDTVITNQEEPYQJHSGR
x1.3e+05
Relative Intensity
61%
49%
37%
24%
12%
y1 /1+
b2 /1+
b3 /1+
y3 /1+
y4 /1+
y$5 /1+
b8 /1+
b9 /1+
b18 /2+
b10 /1+
1 y8 /1+
1 1 Y /3+
2 2 1 Y /3+
3 3 1 Y /3+
y12 /1+
4 3 1 Y /3+
1 b26 /2+y$27 /2+
1 y27 /2+
1 Y /2+
138.05
168.07
186.08
204.09
366.14
Δm (ppm)
m/z

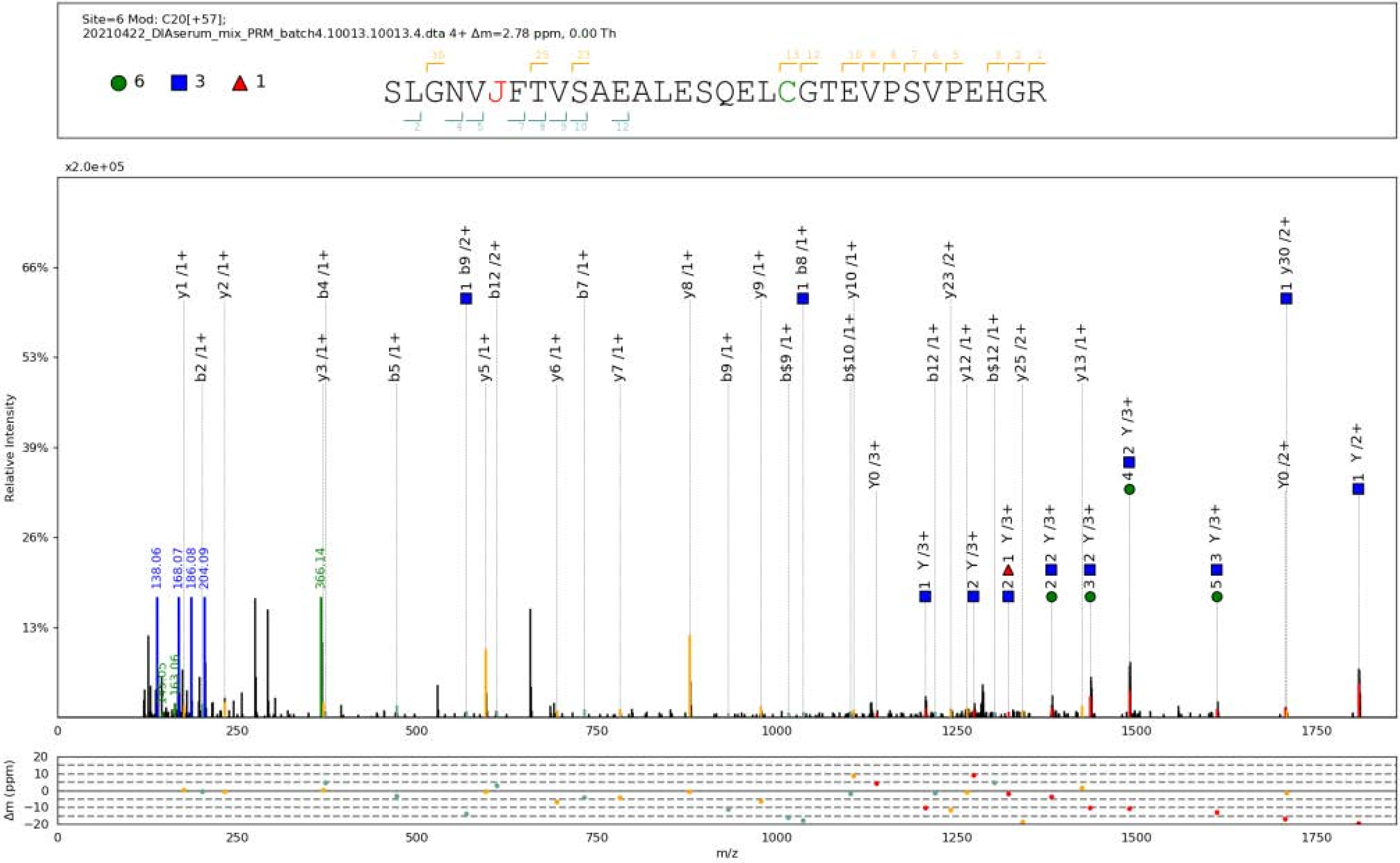

Site=6 Mod: C20[+57];
20210422_DIAserum_mix_PRM_batch4.10013.10013.4.dta 4+ Δm=2.78 ppm, 0.00 Th
6 3 1
SLGNVJFTVSAEALESQELCGTEVPSVPEHGR
x2.0e+05
Relative Intensity
66%
53%
39%
26%
13%
y1 /1+
b2 /1+
y2 /1+
b4 /1+
y3 /1+
b5 /1+
1 b9 /2+
y5 /1+
b12 /2+
y6 /1+
b7 /1+
y7 /1+
y8 /1+
b9 /1+
y9 /1+
b$9 /1+
1 b8 /1+
b$10 /1+
y10 /1+
Y0 /3+
1 Y /3+
b12 /1+
y23 /2+
2 Y /3+
y12 /1+
b$12 /1+
2 1 Y /3+
y25 /2+
2 2 Y /3+
y13 /1+
3 2 Y /3+
4 2 Y /3+
5 3 Y /3+
Y0 /2+
1 y30 /2+
1 Y /2+
138.06
168.07
186.08
204.09
366.14
163.06
0
250
500
750
1000
1250
1500
1750
Δm (ppm)
20
10
0
-10
-20
m/z

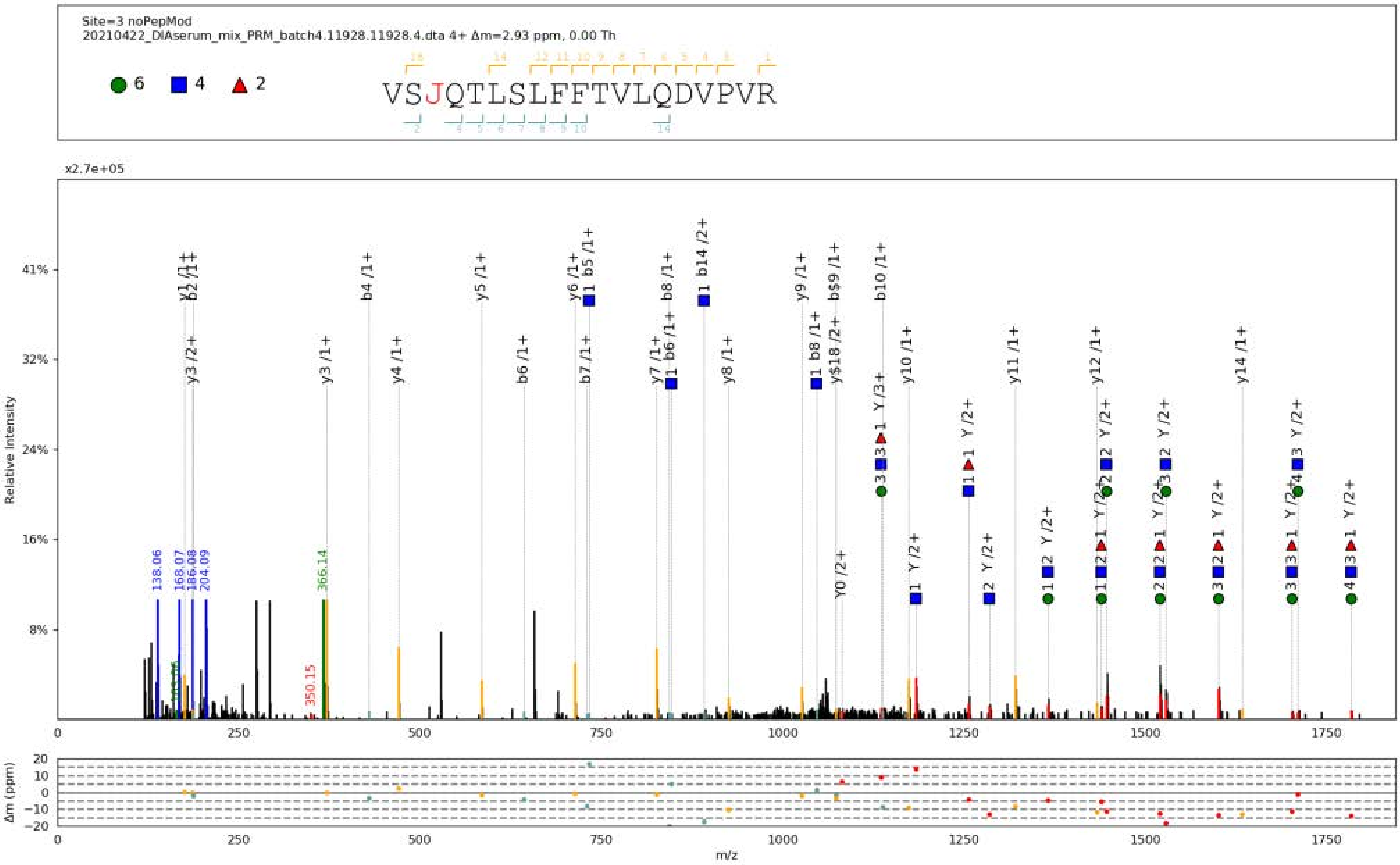

Site=3 noPepMod
20210422_DIAserum_mix_PRM_batch4.11928.11928.4.dta 4+ Δm=2.93 ppm, 0.00 Th
6
4
2
VSJQTLSLFFTVLQDVPVR
x2.7e+05
Relative Intensity
41%
32%
24%
16%
8%
y1 /1+
b2 /1+
y3 /2+
y3 /1+
b4 /1+
y4 /1+
y5 /1+
b6 /1+
y6 /1+
b5 /1+
b7 /1+
y7 /1+
b6 /1+
b8 /1+
b14 /2+
y8 /1+
y9 /1+
b8 /1+
b$9 /1+
y$18 /2+
Y0 /2+
b10 /1+
Y /3+
y10 /1+
Y /2+
y11 /1+
y12 /1+
y14 /1+
138.06
168.07
186.08
204.09
366.14
350.15
m/z
Δm (ppm)

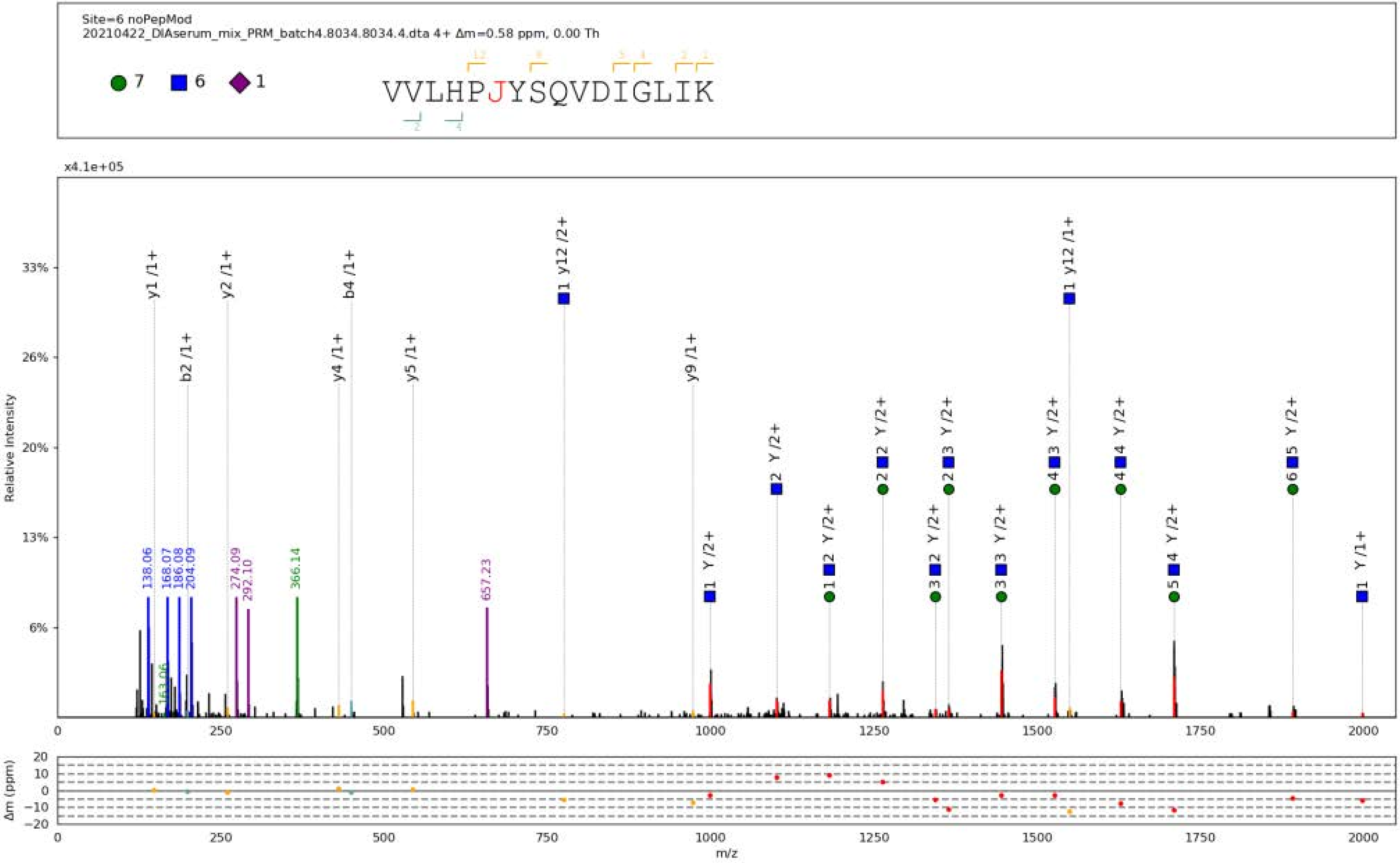

Site=6 noPepMod
20210422_DIAserum_mix_PRM_batch4.8034.8034.4.dta 4+ Δm=0.58 ppm, 0.00 Th
7
6
1
VVLHPJYSQVDIGLIK
x4.1e+05
Relative Intensity
y1 /1+
b2 /1+
y2 /1+
y4 /1+
b4 /1+
y5 /1+
1 y12 /2+
y9 /1+
1 y12 /1+
138.06
168.07
186.08
204.09
274.09
292.10
366.14
657.23
163.06
m/z
Δm (ppm)

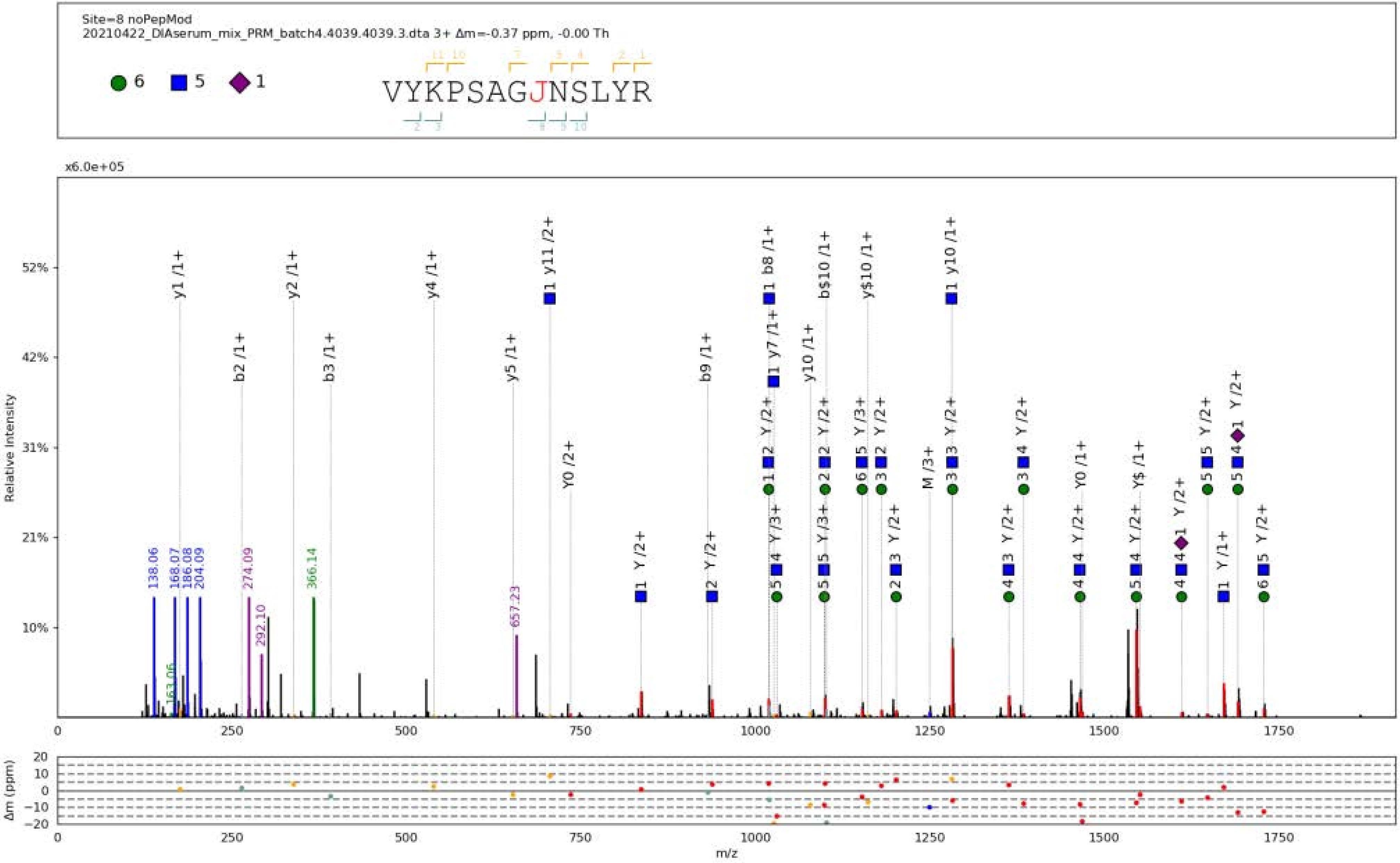

Site=8 noPepMod
20210422_DIAserum_mix_PRM_batch4.4039.4039.3.dta 3+ Δm=-0.37 ppm, -0.00 Th
6 5 1
VYKPSAGJNSLYR
x6.0e+05
Relative Intensity
52%
42%
31%
21%
10%
y1 /1+
b2 /1+
y2 /1+
b3 /1+
y4 /1+
y5 /1+
1 y11 /2+
Y0 /2+
b9 /1+
1 b8 /1+
1 y7 /1+
y10 /1+
b$10 /1+
y$10 /1+
1 y10 /1+
M /3+
Y0 /1+
Y$ /1+
138.06
168.07
186.08
204.09
163.06
274.09
292.10
366.14
657.23
0
250
500
750
1000
1250
1500
1750
Δm (ppm)
20
10
0
-10
-20
m/z

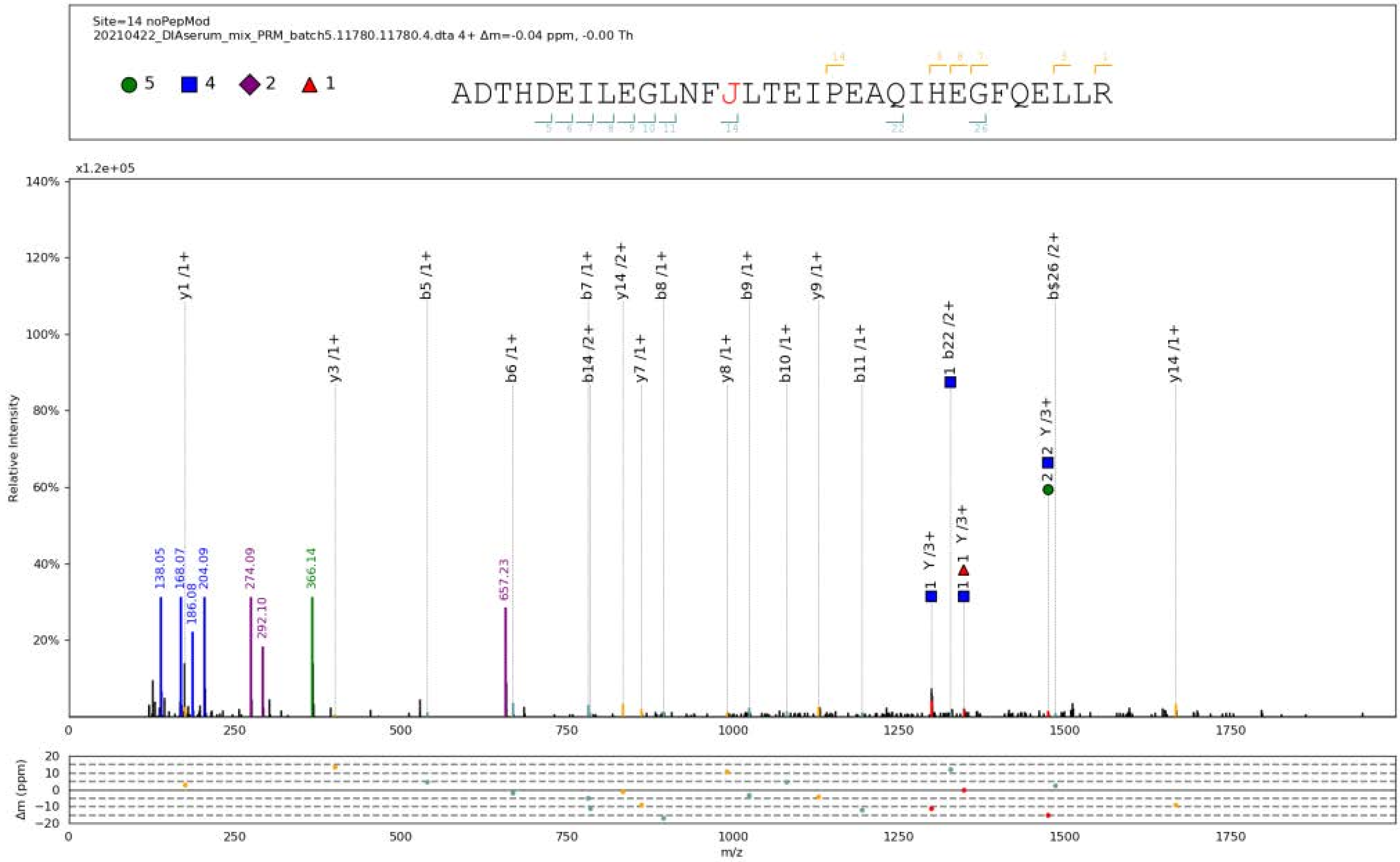

Site=14 noPepMod
20210422_DIAserum_mix_PRM_batch5.11780.11780.4.dta 4+ Δm=-0.04 ppm, -0.00 Th
5
4
2
1
ADTHDEILEGLNFJLTEIPEAQIHEGFQELLR
x1.2e+05
Relative Intensity
y1 /1+
b5 /1+
y3 /1+
b6 /1+
b7 /1+
b14 /2+
y14 /2+
y7 /1+
b8 /1+
y8 /1+
b9 /1+
b10 /1+
y9 /1+
b11 /1+
b22 /2+
b$26 /2+
y14 /1+
138.05
168.07
186.08
204.09
274.09
292.10
366.14
657.23
Y /3+
m/z
Δm (ppm)

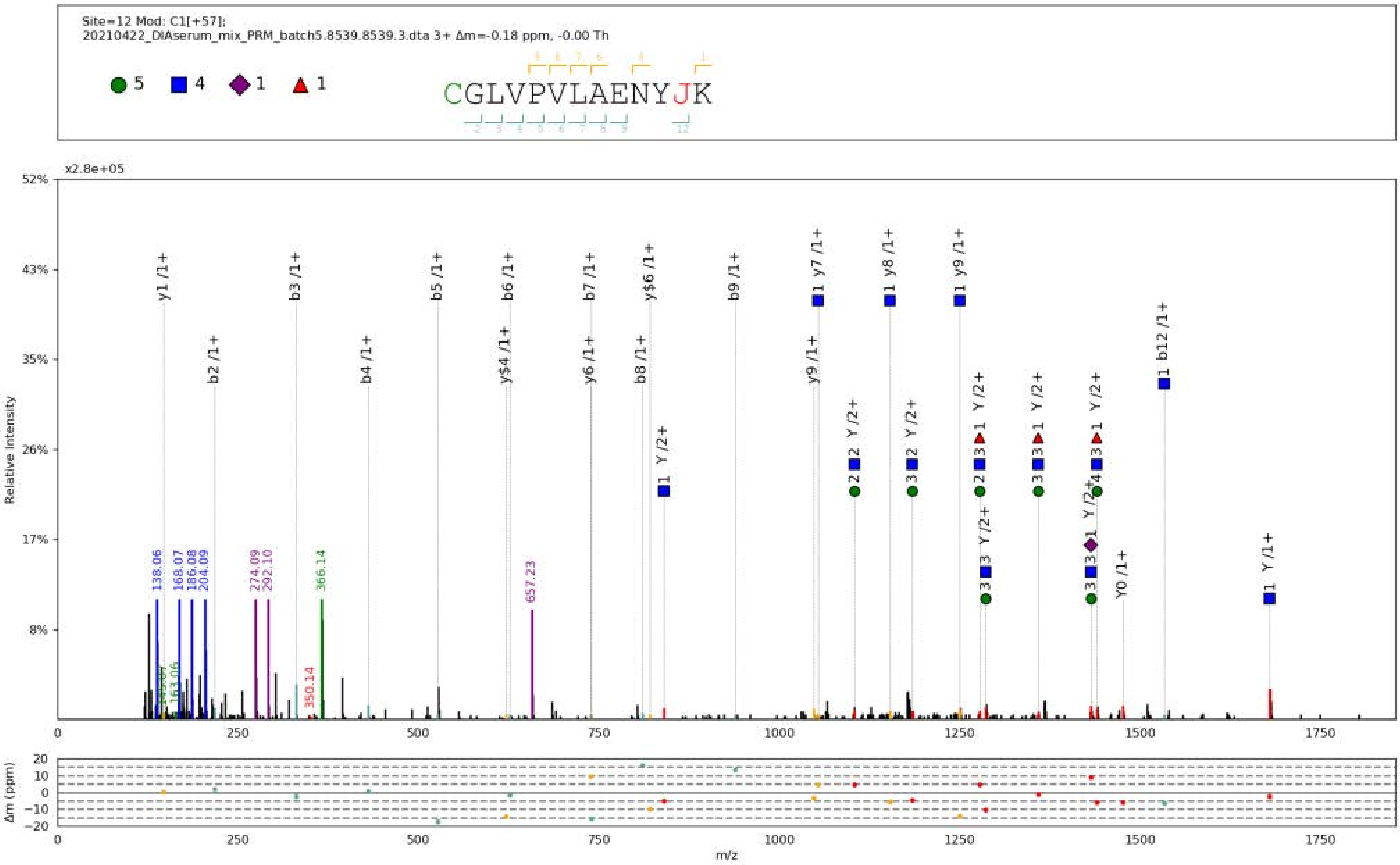
Site=12 Mod: C1[+57];
20210422_DIAserum_mix_PRM_batch5.8539.8539.3.dta 3+ Δm=-0.18 ppm, -0.00 Th
5 4 1 1
CGLVPVLAENYJK
x2.8e+05
Relative Intensity
52%
43%
35%
26%
17%
8%
y1 /1+
b2 /1+
b3 /1+
b4 /1+
b5 /1+
y$4 /1+
b6 /1+
y6 /1+
b7 /1+
b8 /1+
y$6 /1+
1 Y /2+
b9 /1+
y9 /1+
1 y7 /1+
2 2 Y /2+
1 y8 /1+
3 2 Y /2+
1 y9 /1+
2 3 1 Y /2+
3 3 Y /2+
3 3 1 Y /2+
3 4 1 Y /2+
3 3 1 Y /2+
Y0 /1+
1 b12 /1+
1 Y /1+
138.06
168.07
186.08
204.09
274.09
292.10
366.14
657.23
163.06
350.14
Δm (ppm)
m/z
0
250
500
750
1000
1250
1500
1750

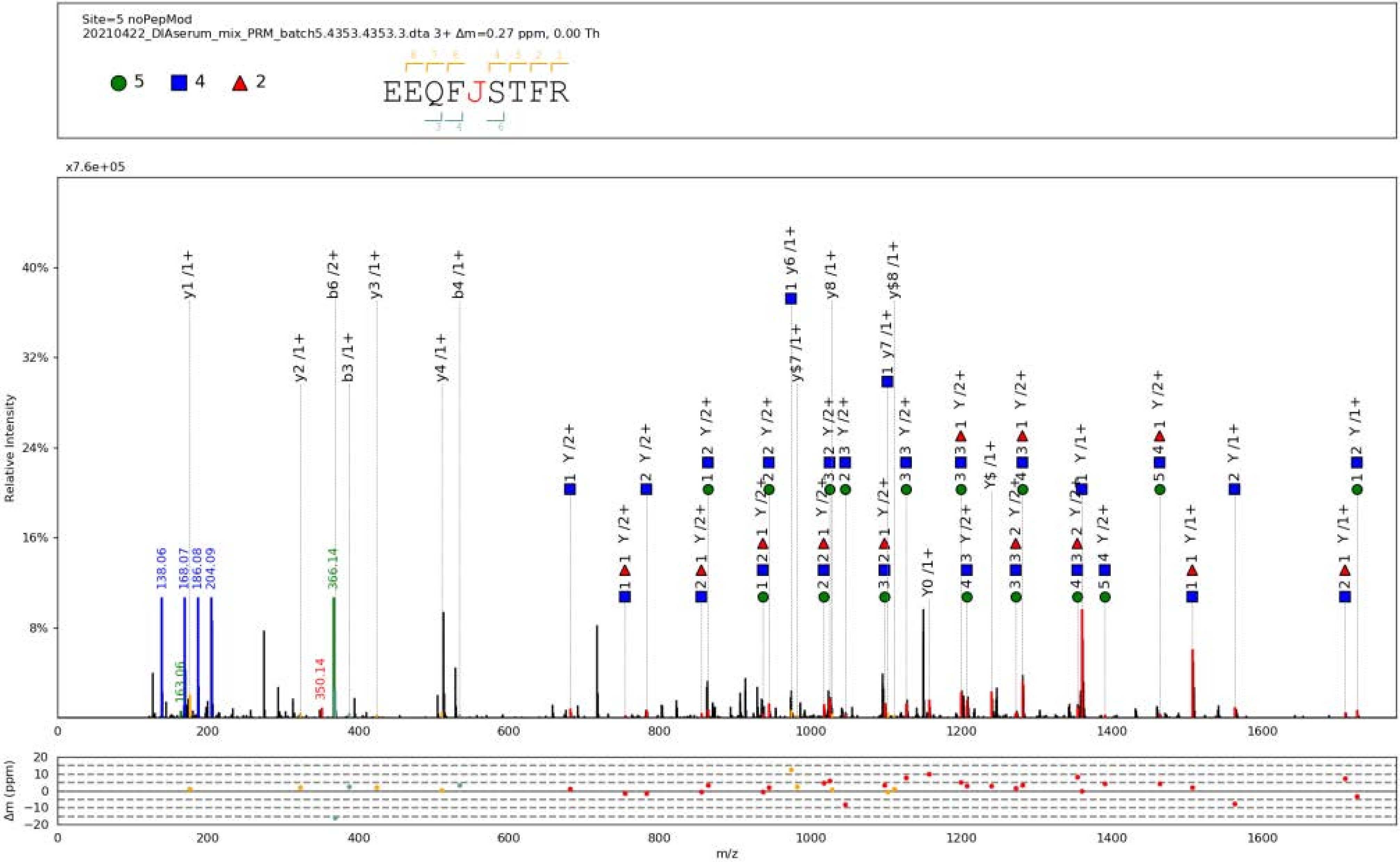
Site=5 noPepMod
20210422_DIAserum_mix_PRM_batch5.4353.4353.3.dta 3+ Δm=0.27 ppm, 0.00 Th
5 4 2
EEQFJSTFR
x7.6e+05
Relative Intensity
40%
32%
24%
16%
8%
y1 /1+
y2 /1+
b6 /2+
b3 /1+
y3 /1+
y4 /1+
b4 /1+
1 y6 /1+
y$7 /1+
y8 /1+
y$8 /1+
1 y7 /1+
Y0 /1+
Y$ /1+
138.06
168.07
186.08
204.09
163.06
366.14
350.14
m/z
Δm (ppm)

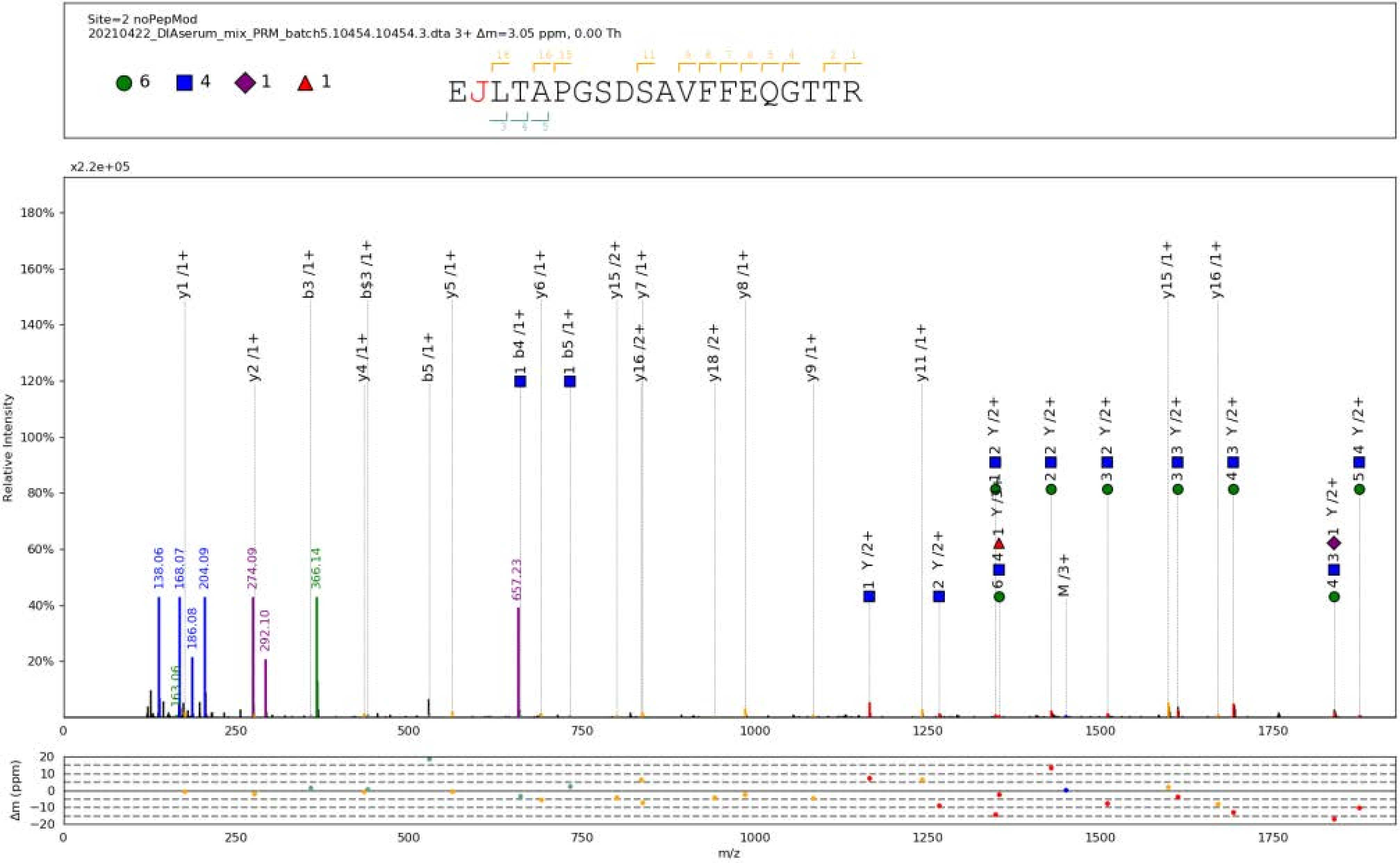
Site=2 noPepMod
20210422_DIAserum_mix_PRM_batch5.10454.10454.3.dta 3+ Δm=3.05 ppm, 0.00 Th
EJLTAPGSDSAVFFEQGTTR
x2.2e+05
Relative Intensity
m/z
Δm (ppm)
y1 /1+
y2 /1+
b3 /1+
b$3 /1+
y4 /1+
b5 /1+
y5 /1+
b4 /1+
y6 /1+
b5 /1+
y15 /2+
y7 /1+
y16 /2+
y18 /2+
y8 /1+
y9 /1+
y11 /1+
y15 /1+
y16 /1+
M /3+
138.06
168.07
186.08
204.09
163.06
274.09
292.10
366.14
657.23

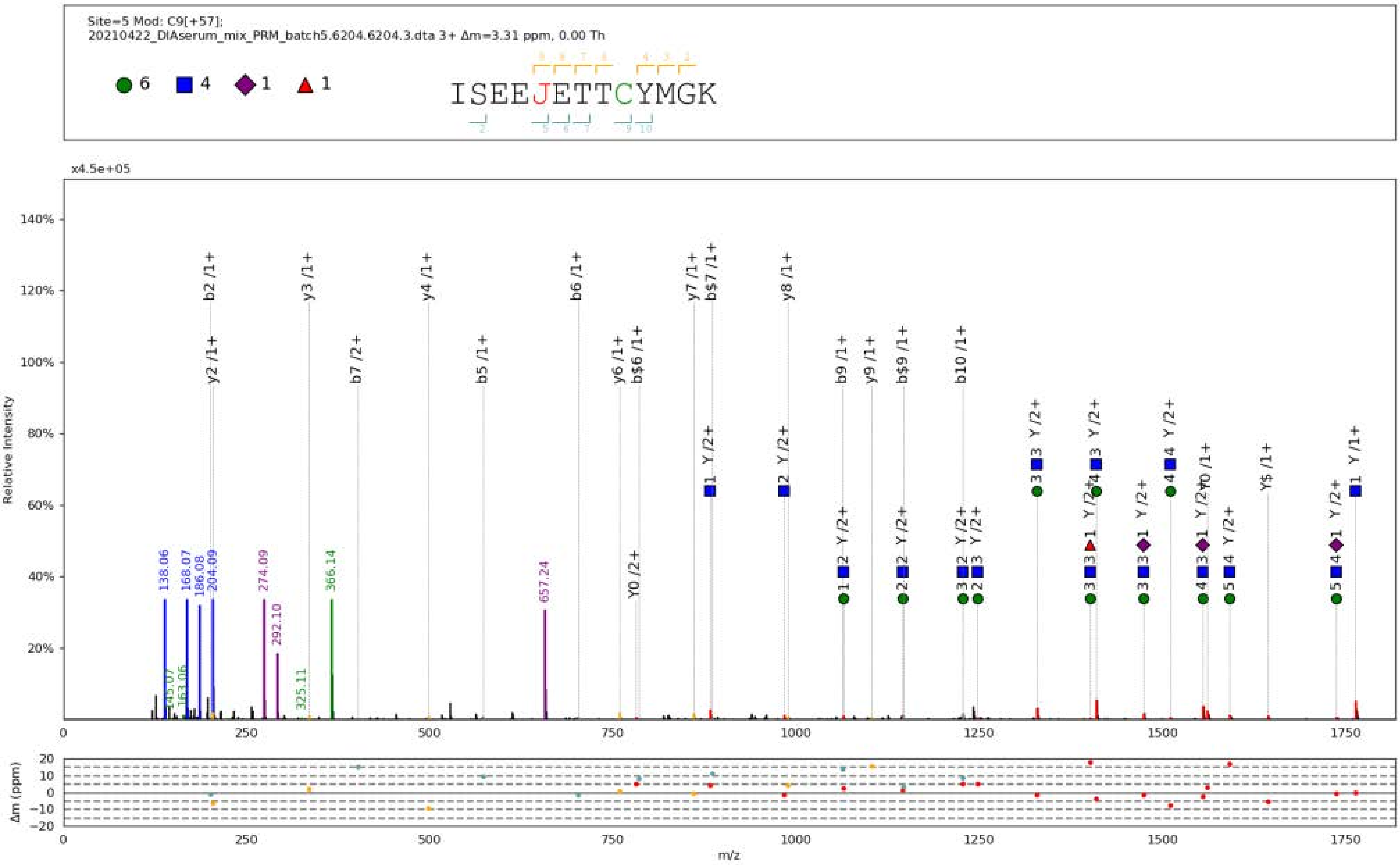
Site=5 Mod: C9[+57];
20210422_DIAserum_mix_PRM_batch5.6204.6204.3.dta 3+ Δm=3.31 ppm, 0.00 Th
6 4 1 1
ISEEJETTCYMGK
x4.5e+05
Relative Intensity
m/z
Δm (ppm)

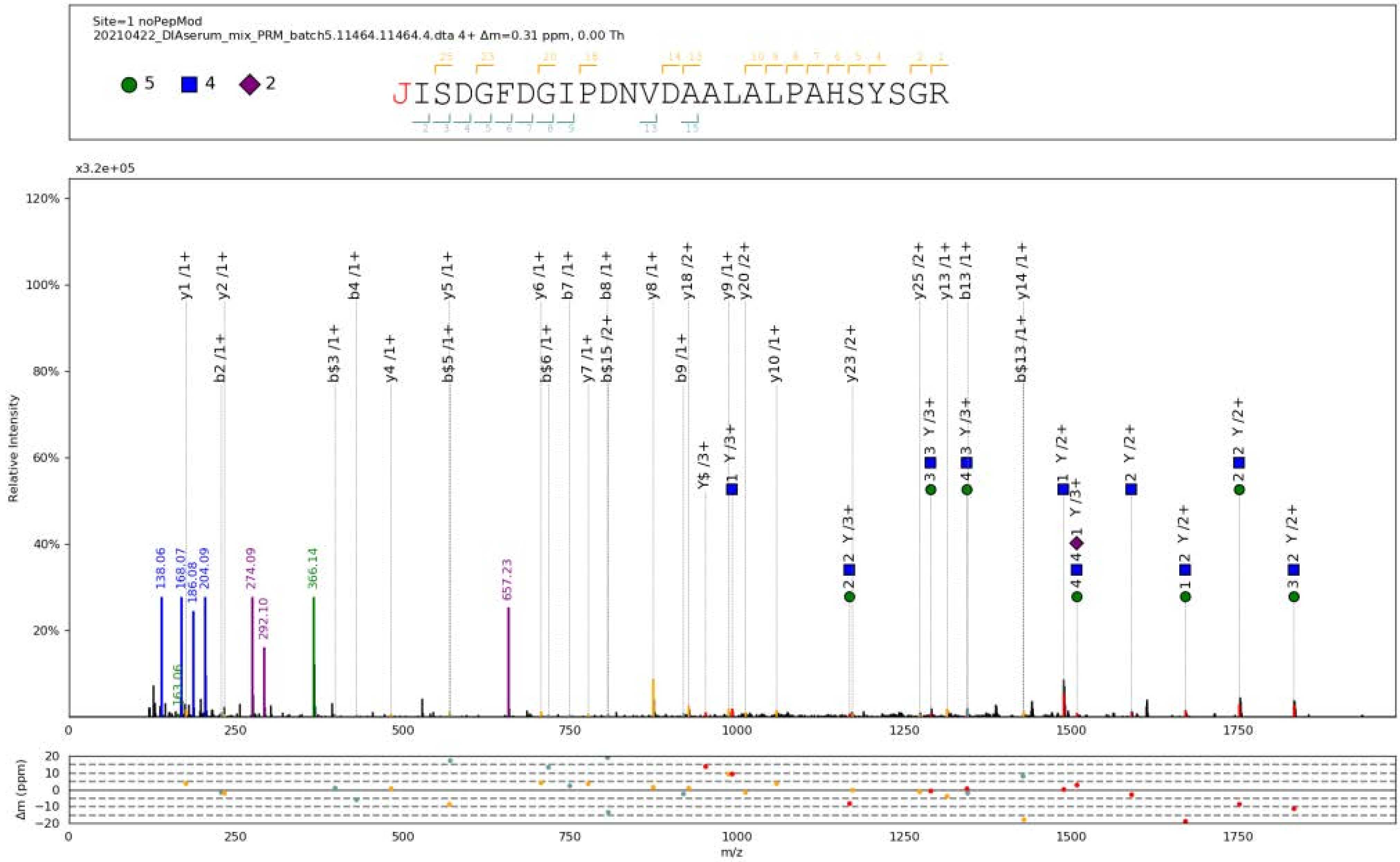
Site=1 noPepMod
20210422_DIAserum_mix_PRM_batch5.11464.11464.4.dta 4+ Δm=0.31 ppm, 0.00 Th
JISDGFDGIPDNVDAALALPAHSYSGR
x3.2e+05
Relative Intensity
m/z
Δm (ppm)

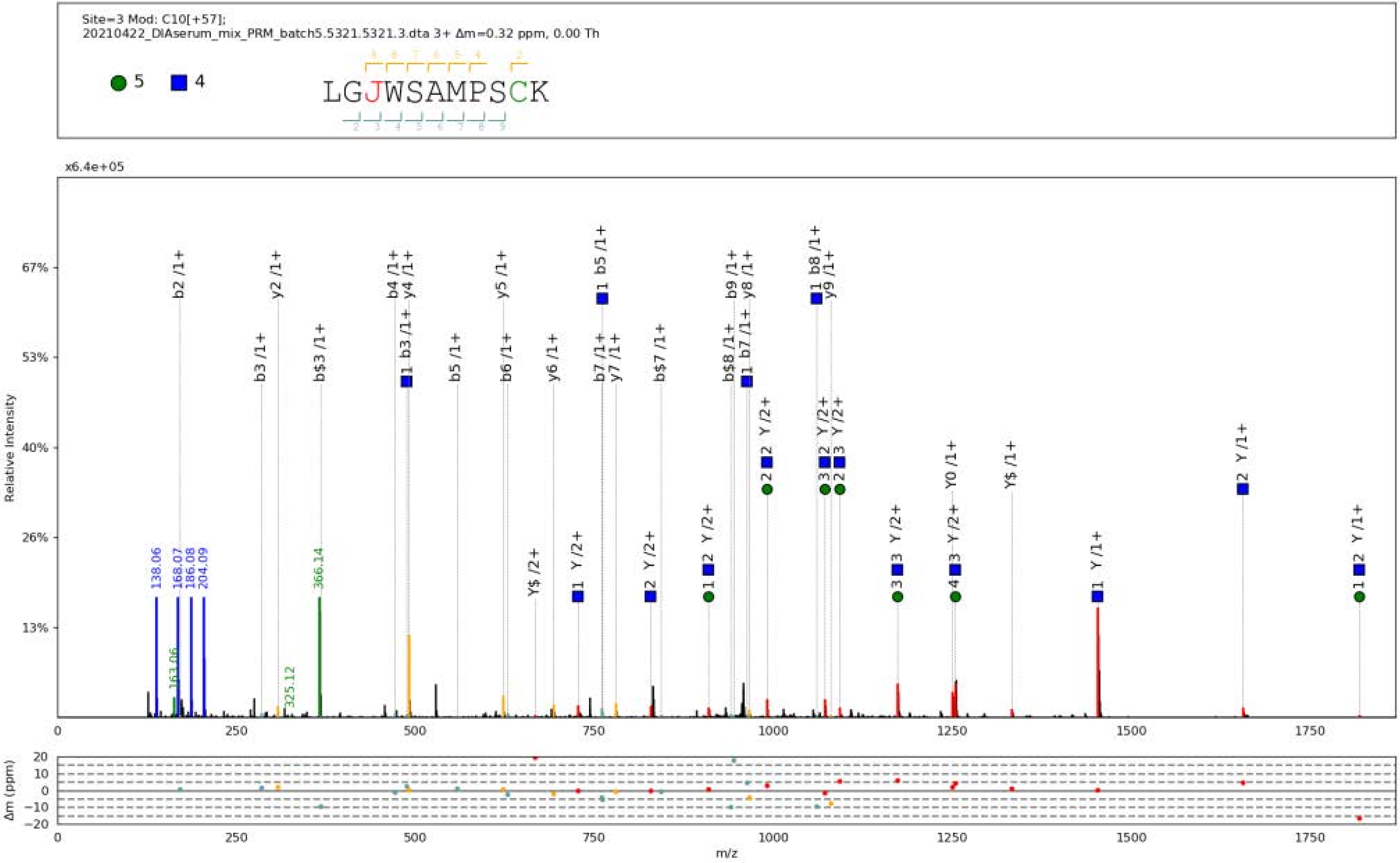
Site=3 Mod: C10[+57];
20210422_DIAserum_mix_PRM_batch5.5321.5321.3.dta 3+ Δm=0.32 ppm, 0.00 Th
LGJWSAMPSCK
x6.4e+05
Relative Intensity
m/z
Δm (ppm)

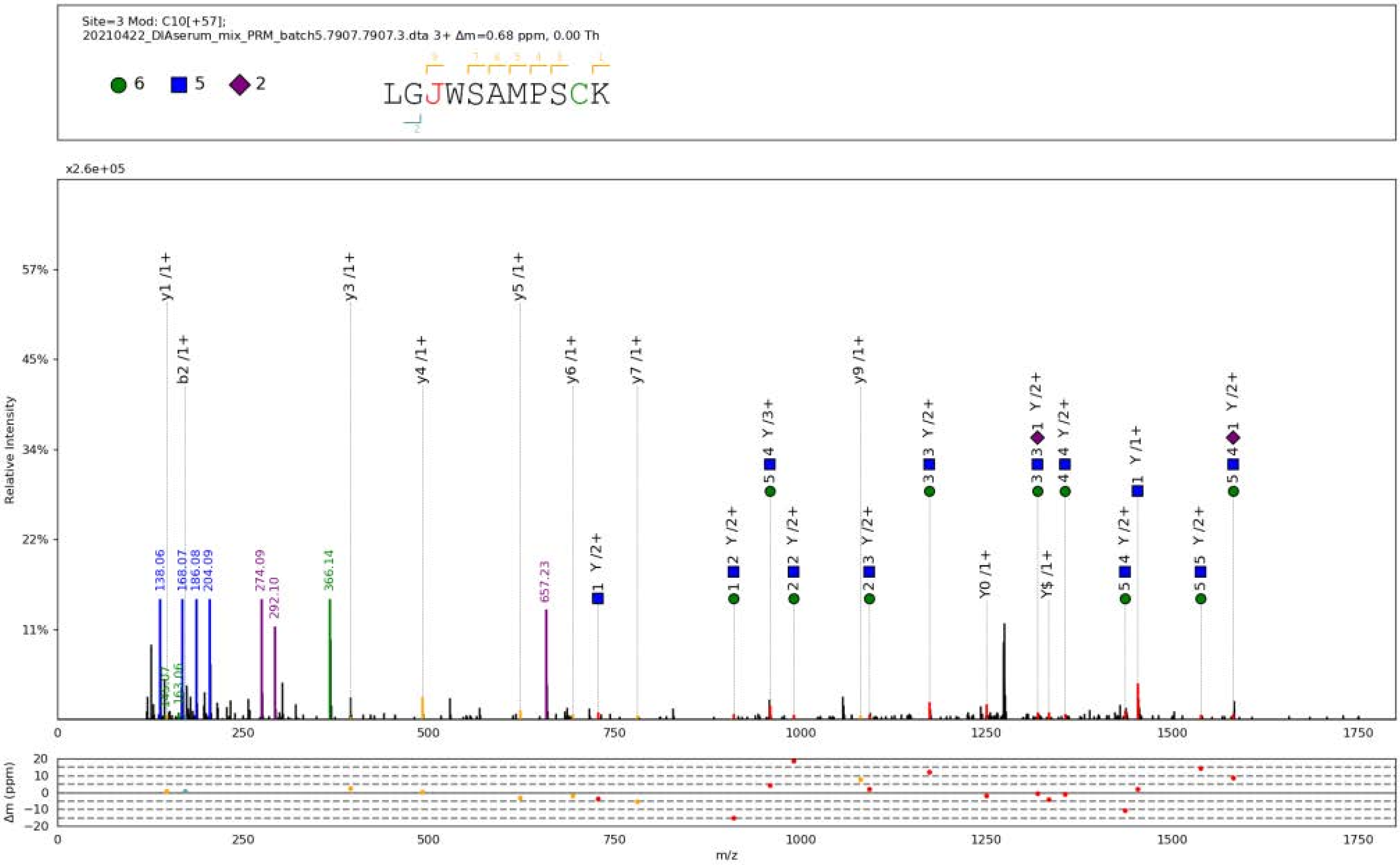
Site=3 Mod: C10[+57];
20210422_DIAserum_mix_PRM_batch5.7907.7907.3.dta 3+ Δm=0.68 ppm, 0.00 Th
6
5
2
LGJWSAMPSCK
x2.6e+05
Relative Intensity
57%
45%
34%
22%
11%
y1 /1+
b2 /1+
y3 /1+
y4 /1+
y5 /1+
y6 /1+
y7 /1+
y9 /1+
Y /2+
Y /3+
Y /1+
Y0 /1+
Y$ /1+
138.06
168.07
186.08
204.09
274.09
292.10
366.14
657.23
163.06
m/z
Δm (ppm)
0
250
500
750
1000
1250
1500
1750
20
10
-10
-20

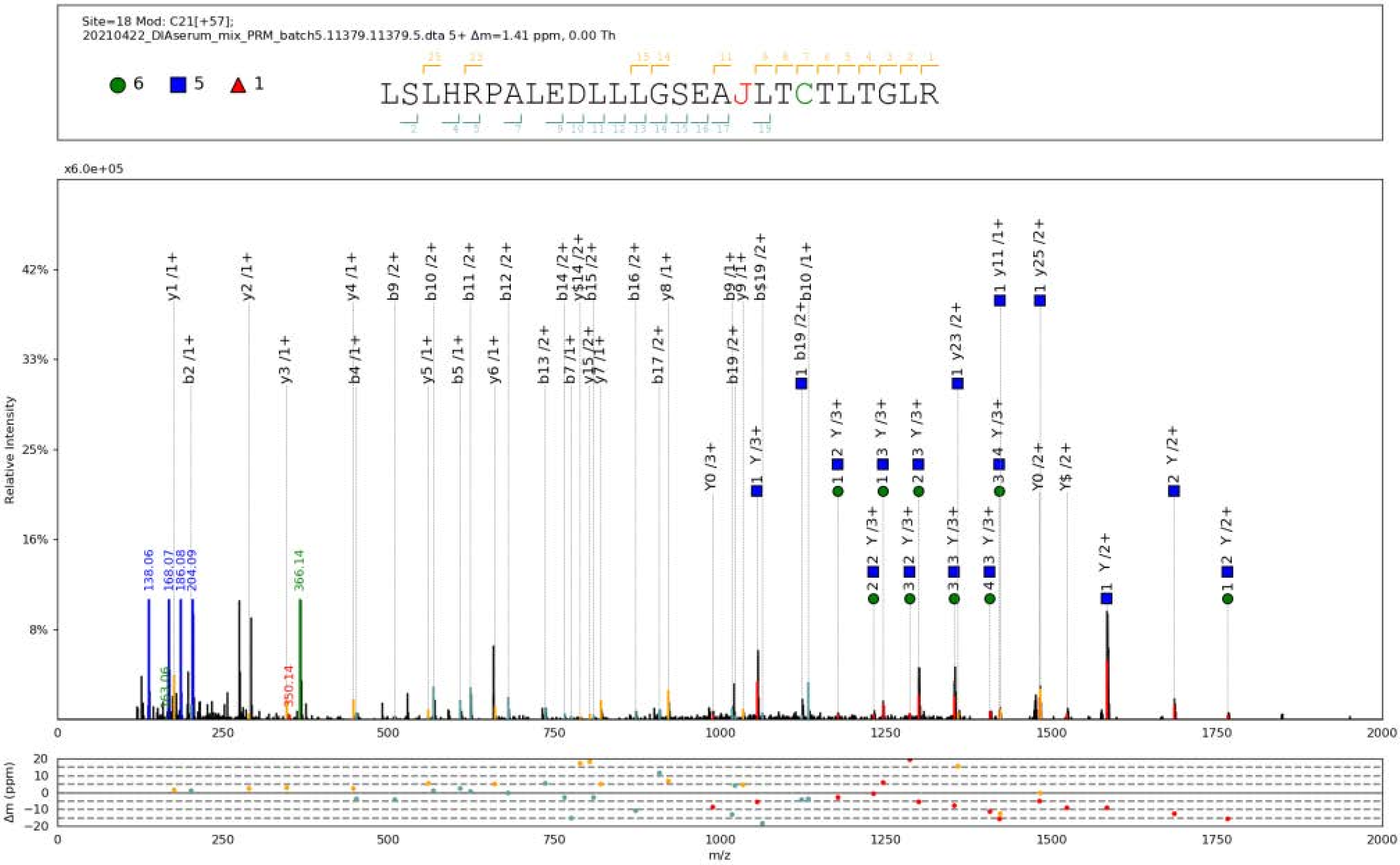

Site=18 Mod: C21[+57];
20210422_DIAserum_mix_PRM_batch5.11379.11379.5.dta 5+ Δm=1.41 ppm, 0.00 Th
6
5
1
LSLHRPALEDLLLGSEAJLTCTLTGLR
x6.0e+05
Relative Intensity
m/z
Δm (ppm)

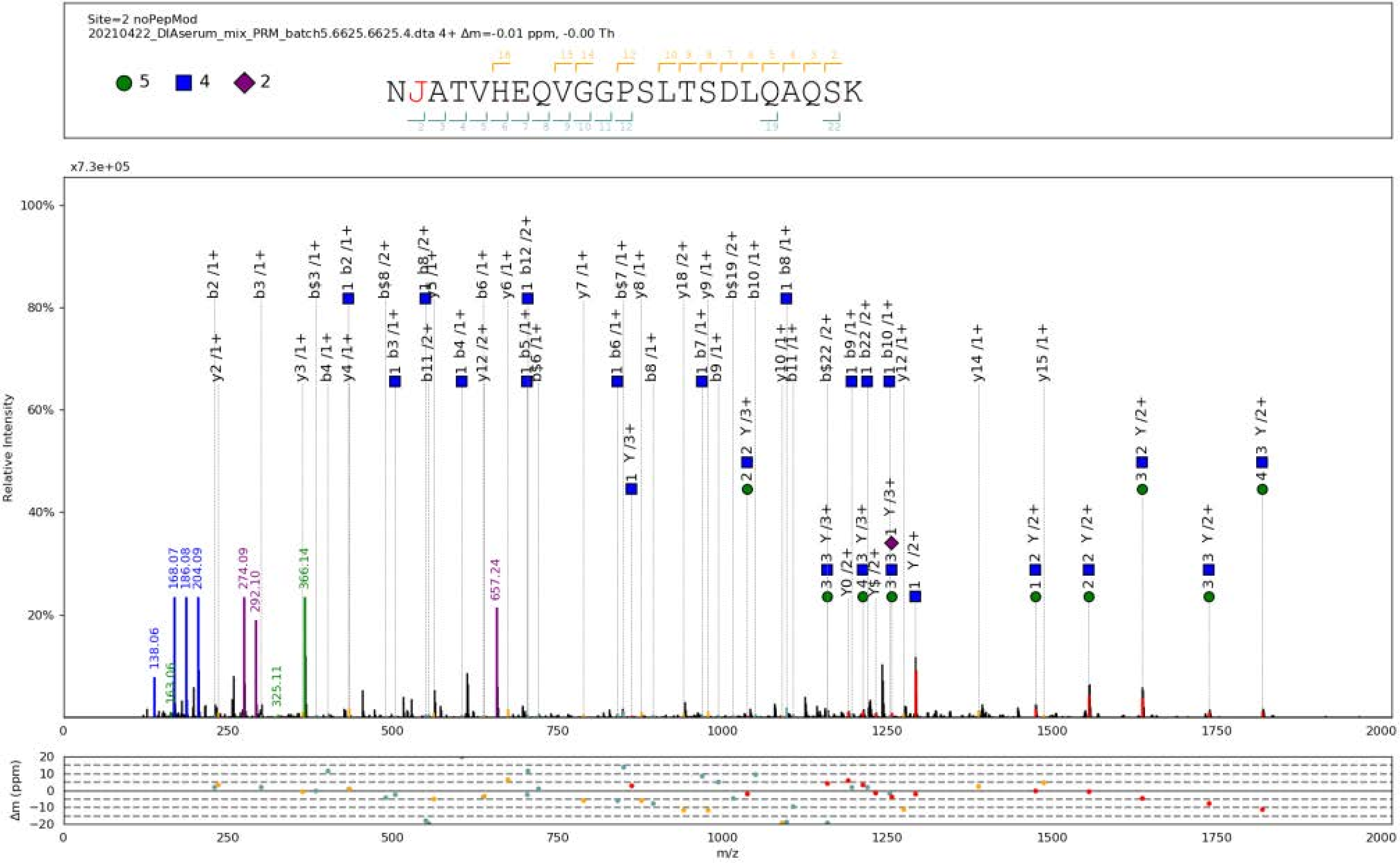

Site=2 noPepMod
20210422_DIAserum_mix_PRM_batch5.6625.6625.4.dta 4+ Δm=-0.01 ppm, -0.00 Th
5
4
2
NJATVHEQVGGPSLTSDLQAQSK
x7.3e+05
Relative Intensity
m/z
Δm (ppm)
138.06
168.07
186.08
204.09
274.09
292.10
366.14
325.11
163.06
657.24

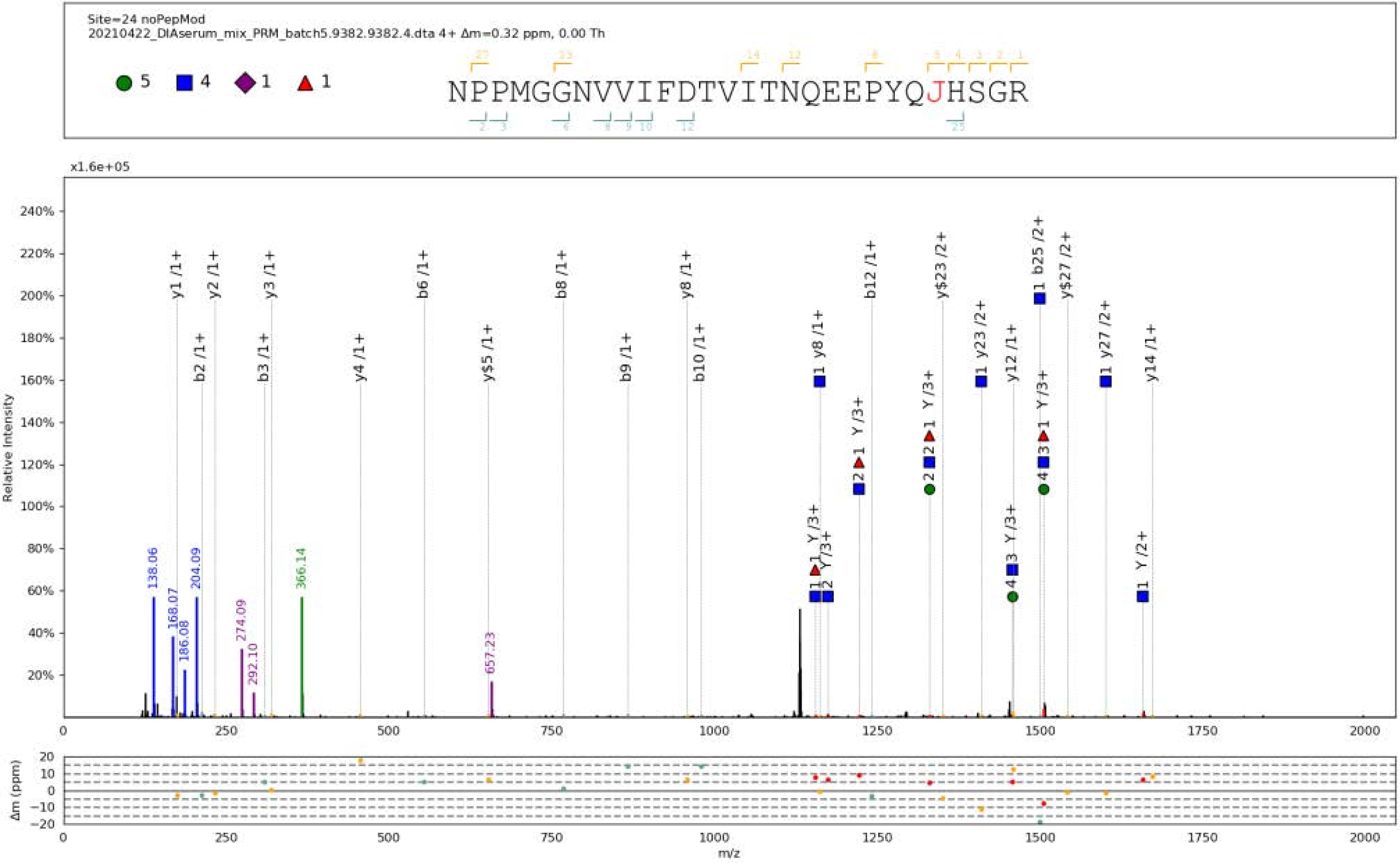

Site=24 noPepMod
20210422_DIAserum_mix_PRM_batch5.9382.9382.4.dta 4+ Δm=0.32 ppm, 0.00 Th
5
4
1
1
NPPMGGNVVIFDTVITNQEEPYQJHSGR
x1.6e+05
Relative Intensity
m/z
Δm (ppm)
y1 /1+
y2 /1+
y3 /1+
b2 /1+
b3 /1+
y4 /1+
b6 /1+
y$5 /1+
b8 /1+
b9 /1+
y8 /1+
b10 /1+
b12 /1+
y$23 /2+
b25 /2+
y$27 /2+
y23 /2+
y12 /1+
y27 /2+
y14 /1+
138.06
168.07
186.08
204.09
274.09
292.10
366.14
657.23

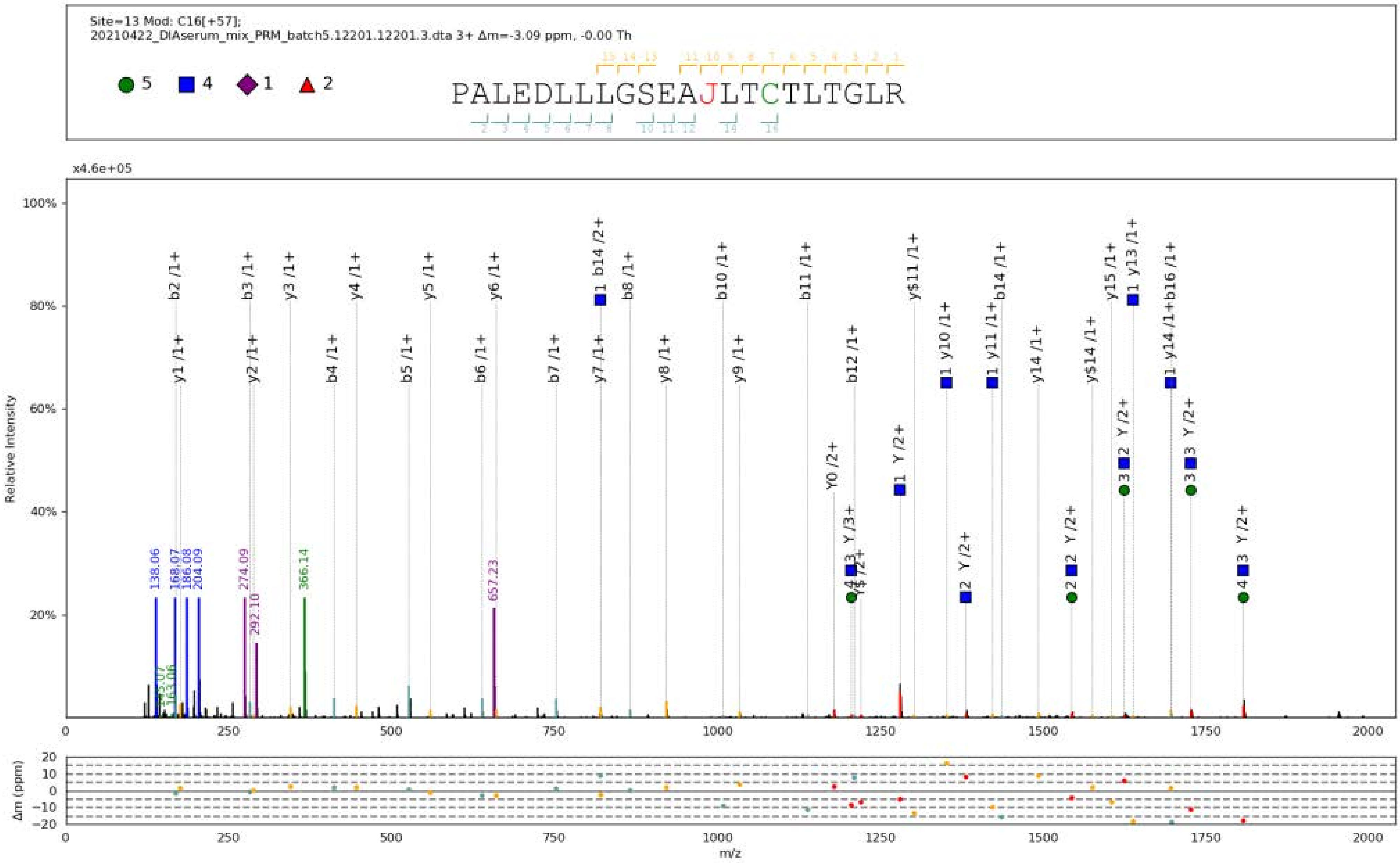

Site=13 Mod: C16[+57];
20210422_DIAserum_mix_PRM_batch5.12201.12201.3.dta 3+ Δm=-3.09 ppm, -0.00 Th
5
4
1
2
PALEDLLLGSEAJLTCTLTGLR
x4.6e+05
Relative Intensity
100%
80%
60%
40%
20%
b2 /1+
y1 /1+
b3 /1+
y2 /1+
y3 /1+
b4 /1+
y4 /1+
b5 /1+
y5 /1+
b6 /1+
y6 /1+
b7 /1+
y7 /1+
1 b14 /2+
b8 /1+
y8 /1+
b10 /1+
y9 /1+
b11 /1+
Y0 /2+
b12 /1+
y$11 /1+
1 Y /2+
1 y10 /1+
1 y11 /1+
b14 /1+
y14 /1+
y$14 /1+
y15 /1+
1 y13 /1+
1 y14 /1+b16 /1+
3 2 Y /2+
3 3 Y /2+
2 Y /2+
2 2 Y /2+
4 3 Y /2+
138.06
168.07
186.08
204.09
274.09
292.10
366.14
657.23
0
250
500
750
1000
1250
1500
1750
2000
Δm (ppm)
20
10
0
-10
-20
m/z

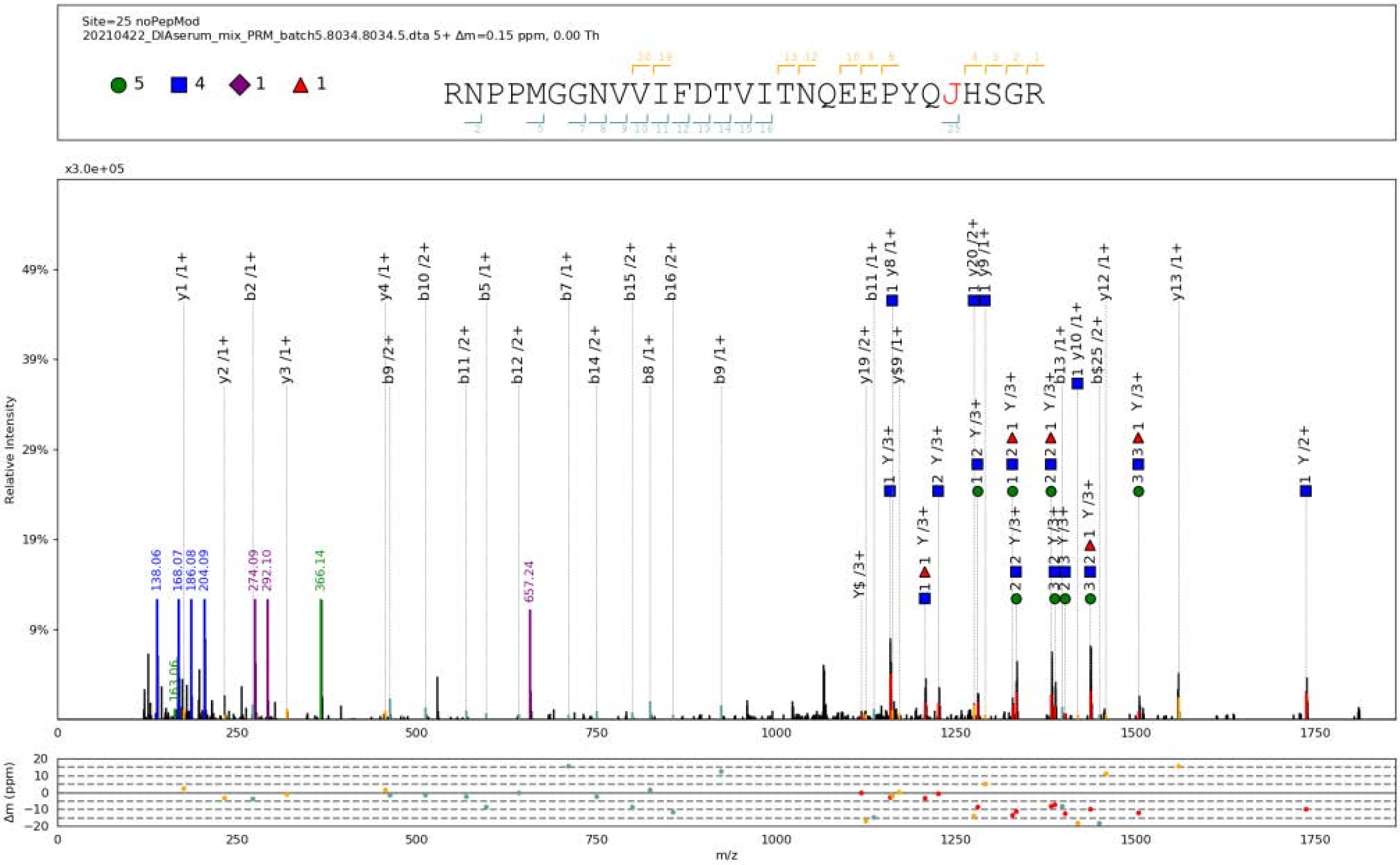
Site=25 noPepMod
20210422_DIAserum_mix_PRM_batch5.8034.8034.5.dta 5+ Δm=0.15 ppm, 0.00 Th
RNPPMGGNVVIFDTVITNQEEPYQJHSGR
x3.0e+05
Relative Intensity
m/z
Δm (ppm)

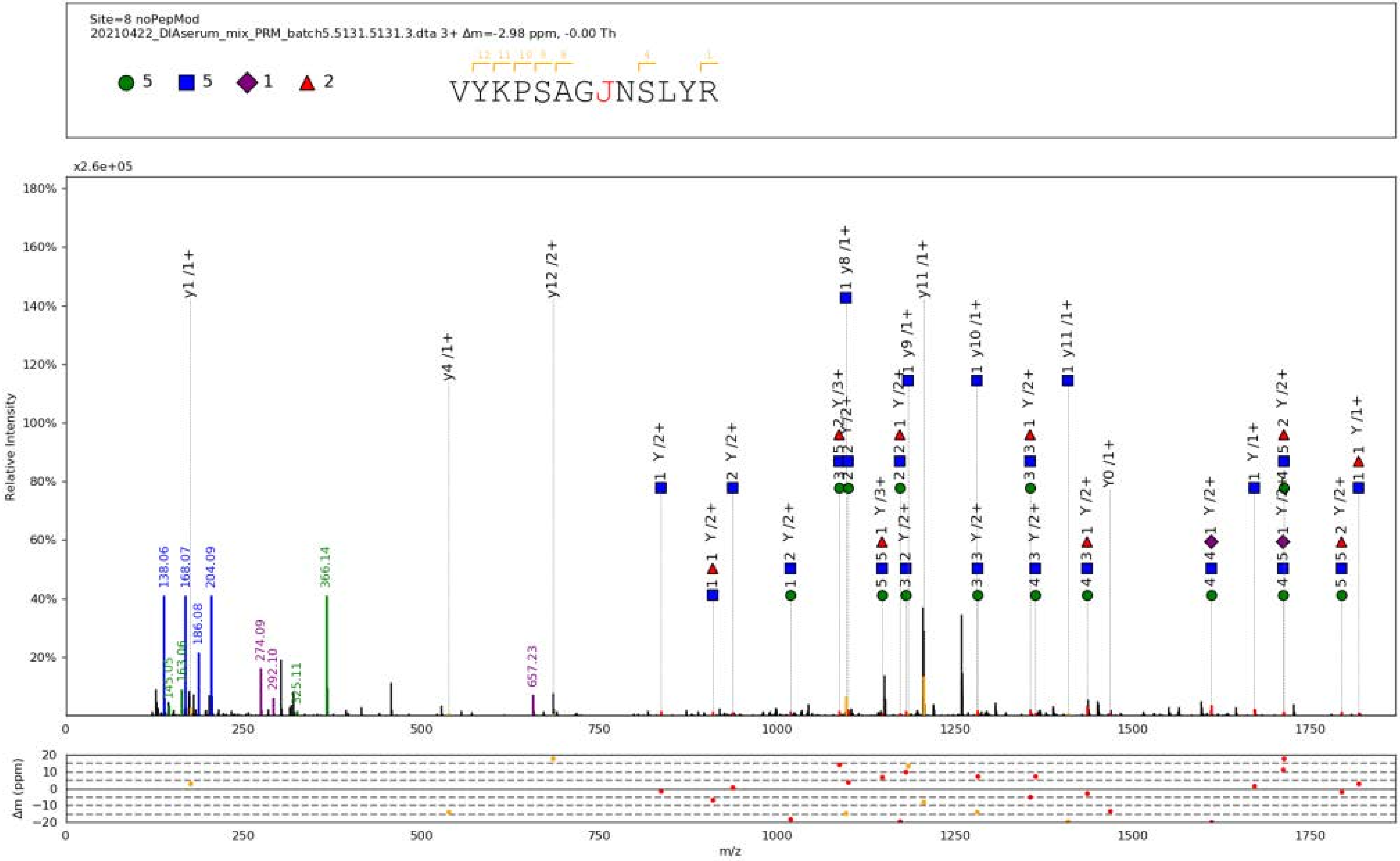

Site=8 noPepMod
20210422_DIAserum_mix_PRM_batch5.5131.5131.3.dta 3+ Δm=-2.98 ppm, -0.00 Th
VYKPSAGJNSLYR
x2.6e+05
Relative Intensity
y1 /1+
y12 /2+
y4 /1+
y8 /1+
y9 /1+
y11 /1+
y10 /1+
Y0 /1+
138.06
168.07
204.09
186.08
366.14
274.09
292.10
657.23
m/z
Δm (ppm)

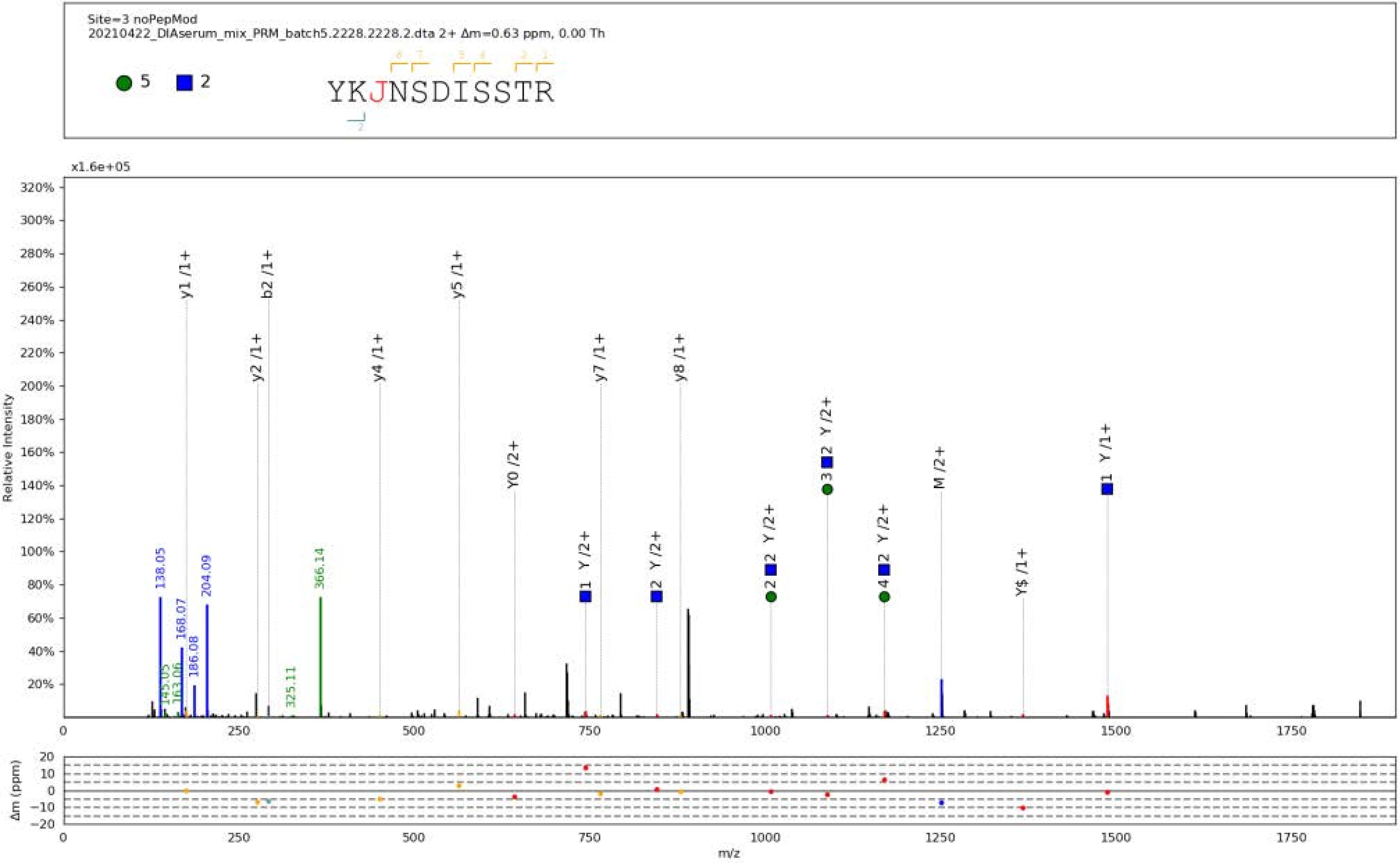

Site=3 noPepMod
20210422_DIAserum_mix_PRM_batch5.2228.2228.2.dta 2+ Δm=0.63 ppm, 0.00 Th
5
2
YKJNSDISSTR
x1.6e+05
Relative Intensity
y1 /1+
b2 /1+
y5 /1+
y2 /1+
y4 /1+
y7 /1+
y8 /1+
Y0 /2+
1 Y /2+
2 Y /2+
2 2 Y /2+
3 2 Y /2+
4 2 Y /2+
M /2+
Y$ /1+
1 Y /1+
138.05
168.07
186.08
204.09
145.05
163.06
366.14
325.11
Δm (ppm)
m/z

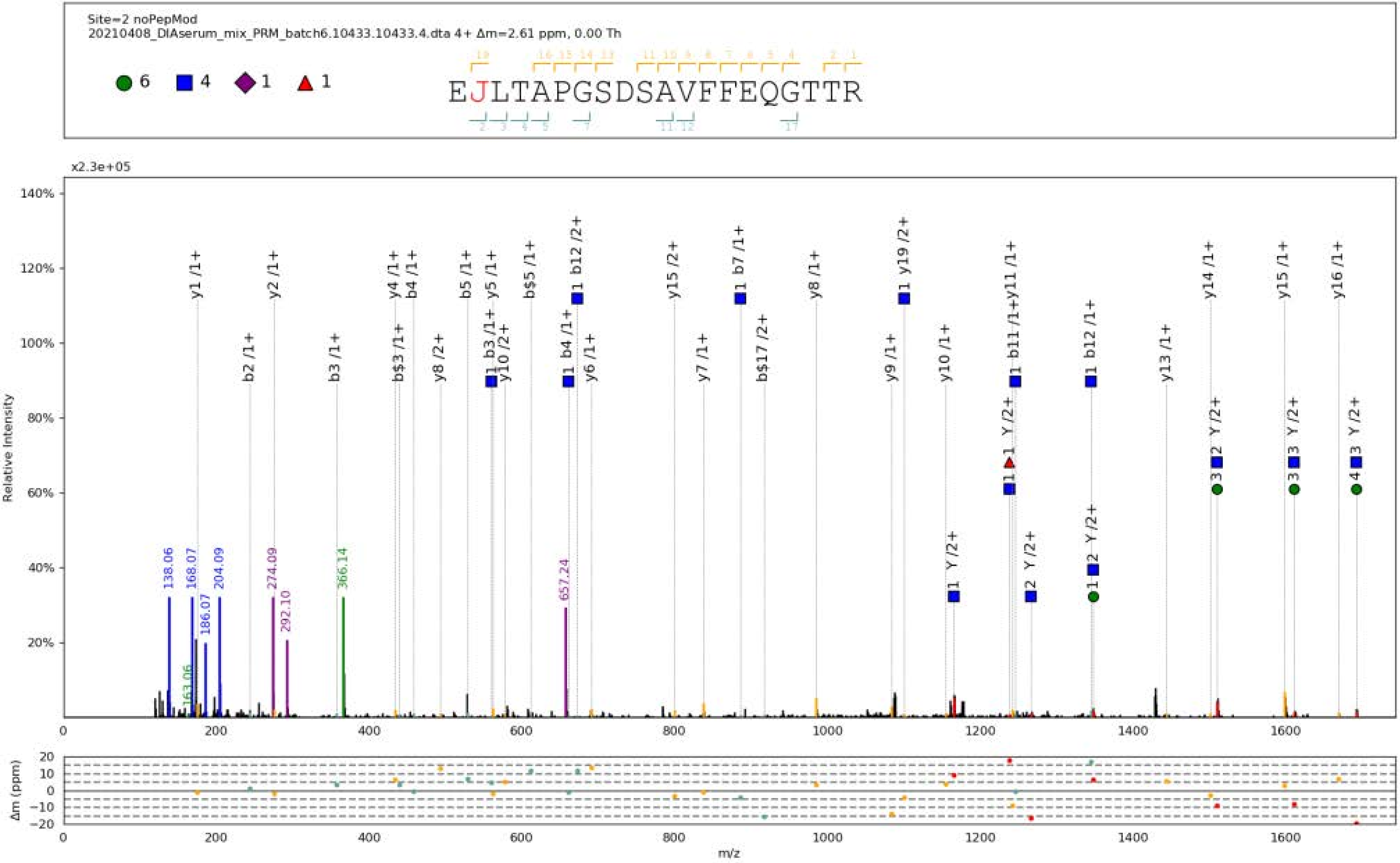
Site=2 noPepMod
20210408_DIAserum_mix_PRM_batch6.10433.10433.4.dta 4+ Δm=2.61 ppm, 0.00 Th
6 4 1 1
EJLTAPGSDSAVFFEQGTTR
x2.3e+05
Relative Intensity
y1 /1+
b2 /1+
y2 /1+
b3 /1+
y4 /1+
b$3 /1+
b4 /1+
y8 /2+
b5 /1+
y5 /1+
b3 /1+
y10 /2+
b$5 /1+
b4 /1+
b12 /2+
y6 /1+
y15 /2+
y7 /1+
b7 /1+
b$17 /2+
y8 /1+
y9 /1+
y19 /2+
y10 /1+
b11 /1+
y11 /1+
b12 /1+
y13 /1+
y14 /1+
y15 /1+
y16 /1+
Y /2+
138.06
168.07
186.07
204.09
163.06
274.09
292.10
366.14
657.24
m/z
Δm (ppm)

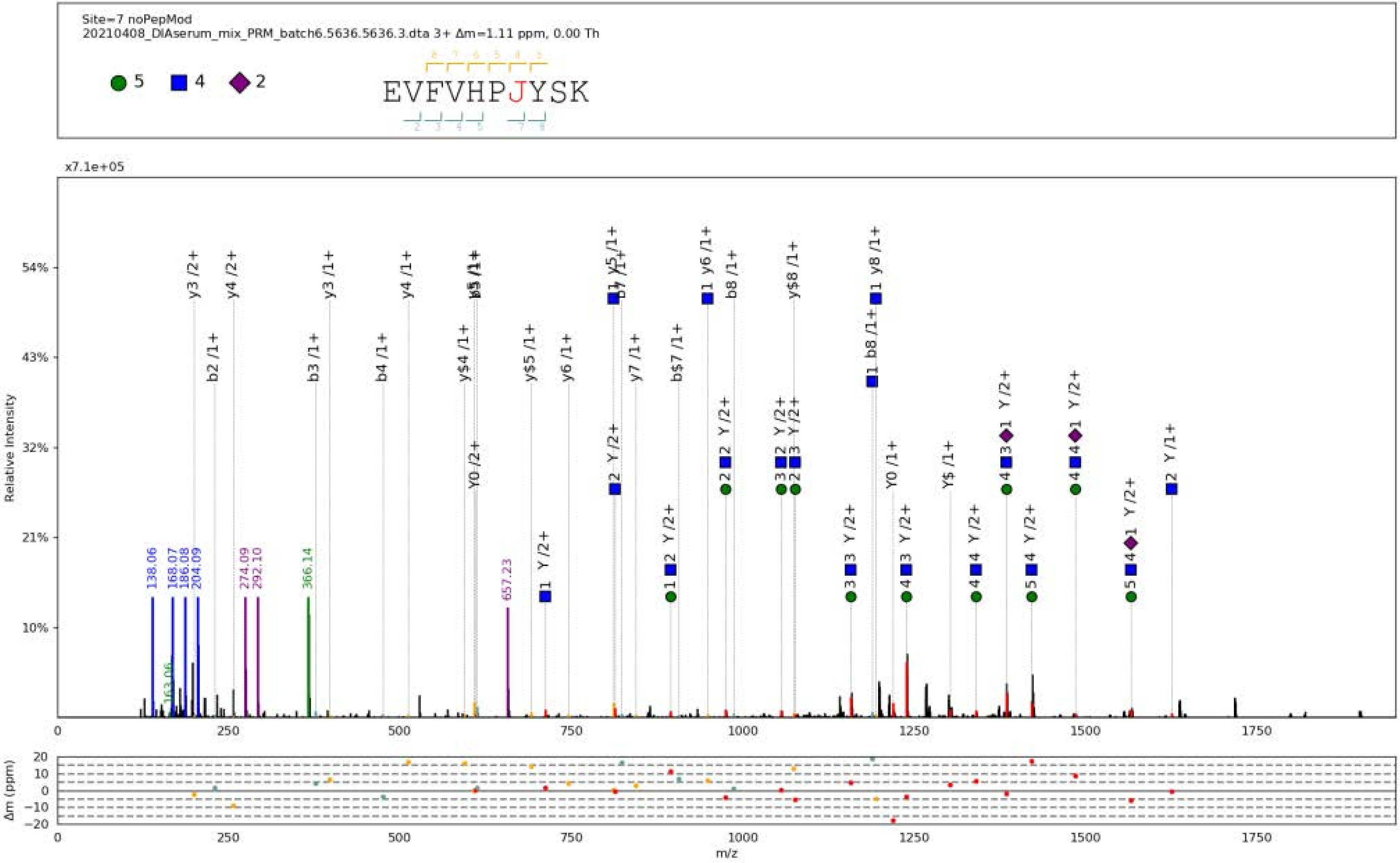

Site=7 noPepMod
20210408_DIAserum_mix_PRM_batch6.5636.5636.3.dta 3+ Δm=1.11 ppm, 0.00 Th
5
4
2
EVFVHPJYSK
x7.1e+05
Relative Intensity
54%
43%
32%
21%
10%
138.06
168.07
186.08
204.09
274.09
292.10
366.14
657.23
163.06
y3 /2+
b2 /1+
y4 /2+
b3 /1+
y3 /1+
b4 /1+
y4 /1+
y$4 /1+
Y0 /2+
y$5 /1+
y6 /1+
y7 /1+
b$7 /1+
y6 /1+
b8 /1+
y$8 /1+
b8 /1+
y8 /1+
Y0 /1+
Y$ /1+
Y /2+
Y /1+
m/z
Δm (ppm)
0
250
500
750
1000
1250
1500
1750
20
10
0
−10
−20

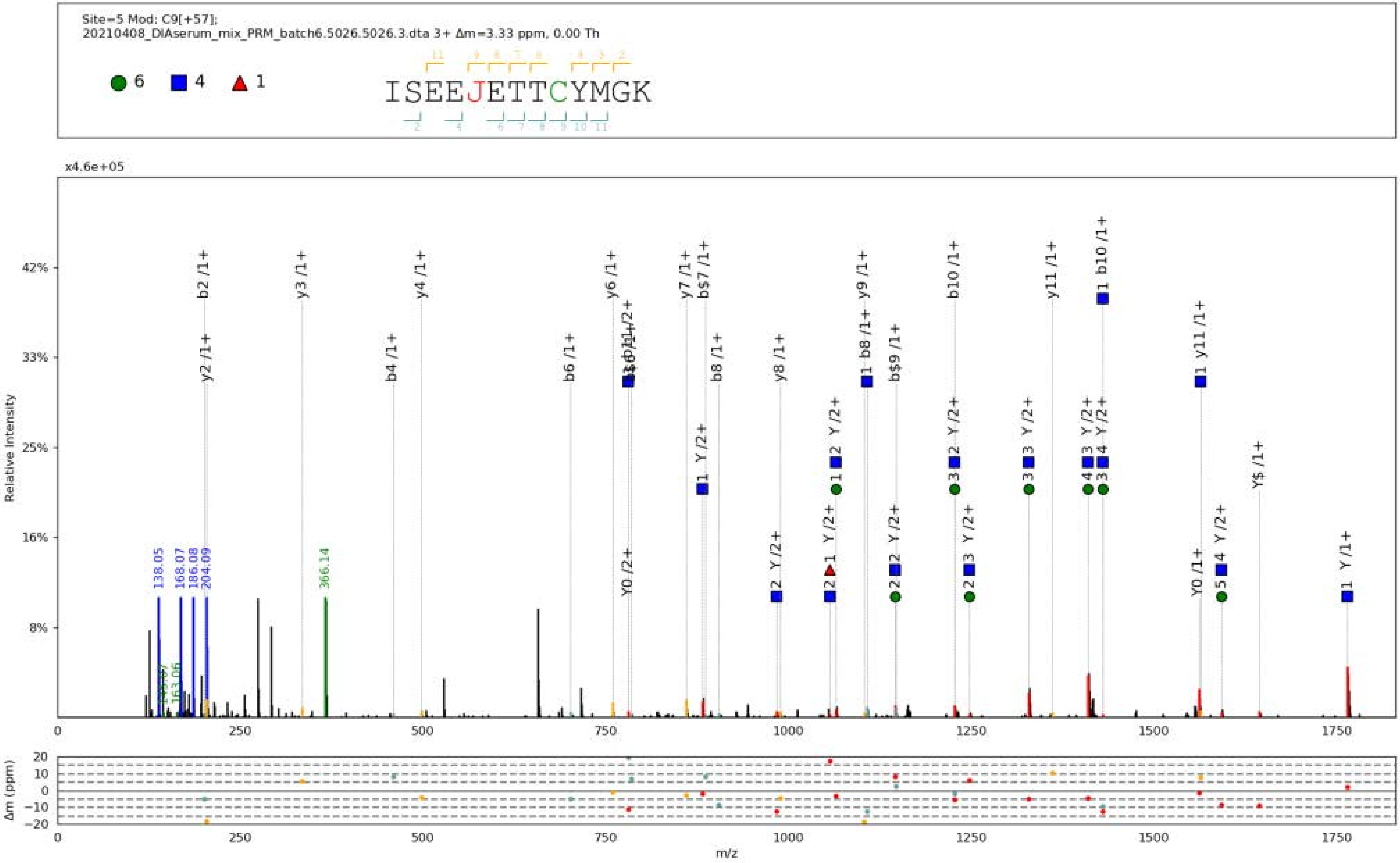
Site=5 Mod: C9[+57];
20210408_DIAserum_mix_PRM_batch6.5026.5026.3.dta 3+ Δm=3.33 ppm, 0.00 Th
ISEEJETTCYMGK
x4.6e+05
Relative Intensity
m/z
Δm (ppm)

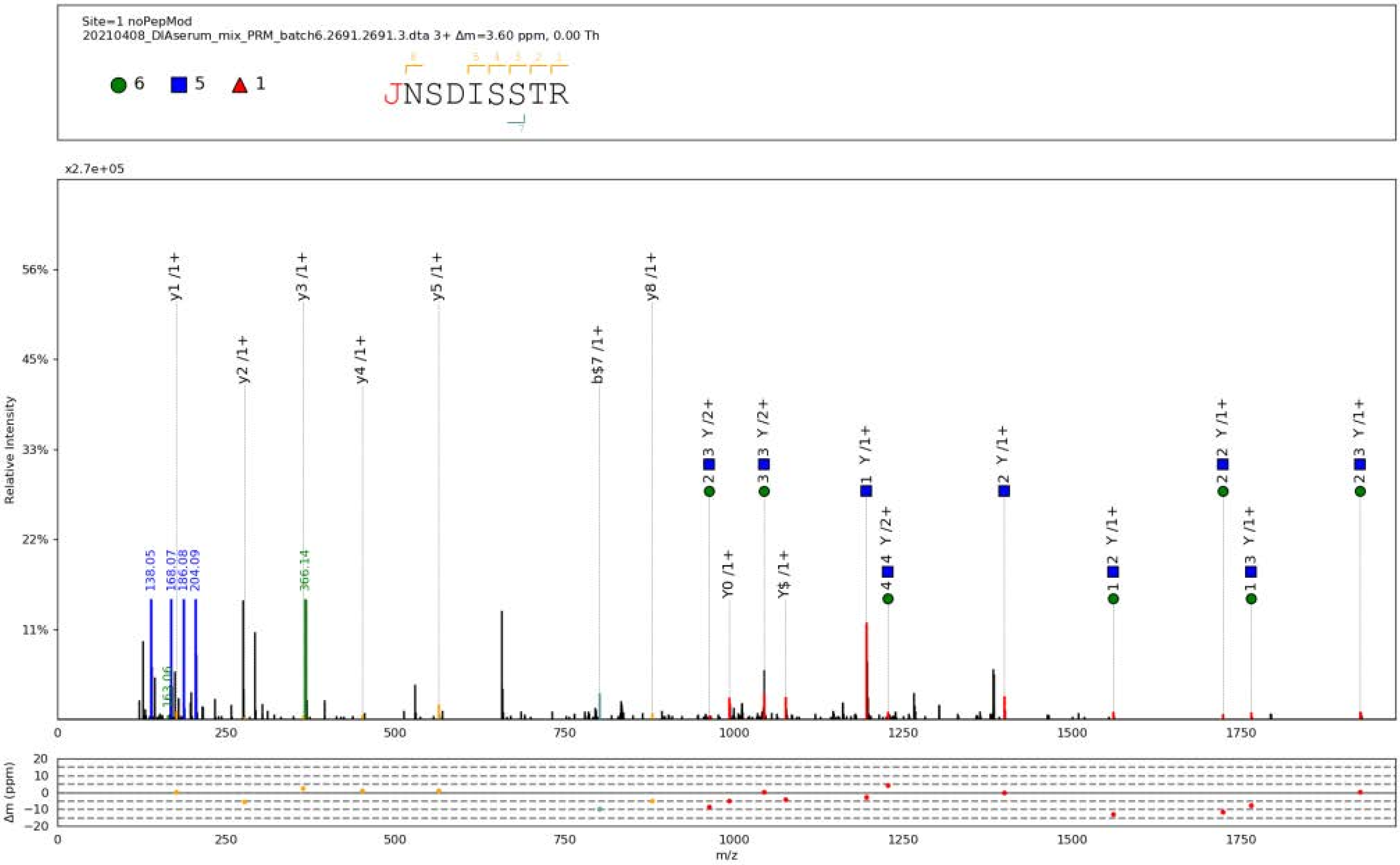
Site=1 noPepMod
20210408_DIAserum_mix_PRM_batch6.2691.2691.3.dta 3+ Δm=3.60 ppm, 0.00 Th
6
5
1
JNSDISSTR
x2.7e+05
Relative Intensity
56%
45%
33%
22%
11%
y1 /1+
y2 /1+
y3 /1+
y4 /1+
y5 /1+
b$7 /1+
y8 /1+
2 3 Y /2+
3 3 Y /2+
Y0 /1+
Y$ /1+
1 Y /1+
4 4 Y /2+
2 Y /1+
1 2 Y /1+
2 2 Y /1+
1 3 Y /1+
2 3 Y /1+
138.05
168.07
186.08
204.09
366.14
163.06
0
250
500
750
1000
1250
1500
1750
Δm (ppm)
20
10
0
-10
-20
m/z

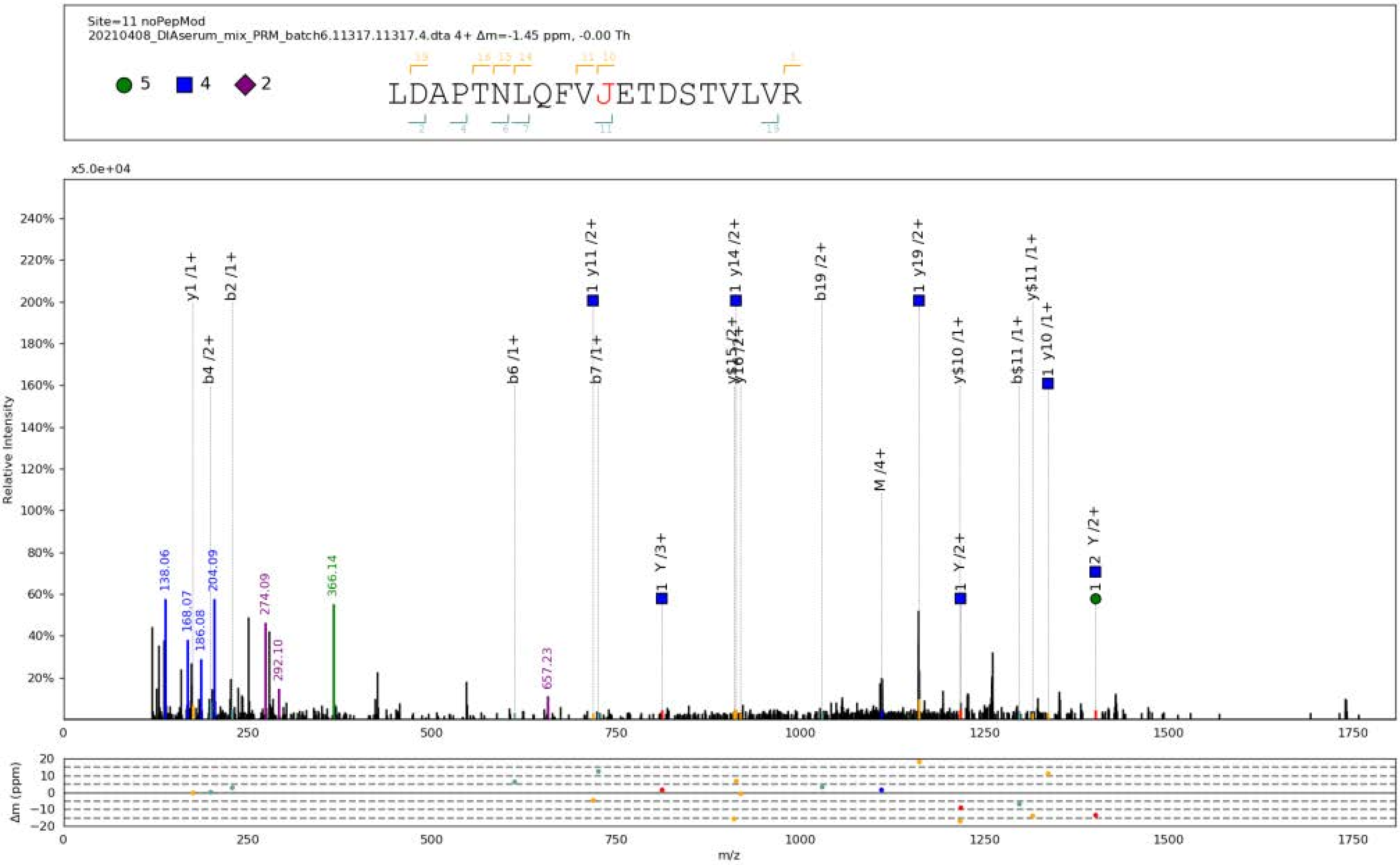

Site=11 noPepMod
20210408_DIAserum_mix_PRM_batch6.11317.11317.4.dta 4+ Δm=-1.45 ppm, -0.00 Th
5
4
2
LDAPTNLQFVJETDSTVLVR
x5.0e+04
Relative Intensity
m/z
Δm (ppm)
y1 /1+
b2 /1+
b4 /2+
b6 /1+
b7 /1+
1 y11 /2+
1 y14 /2+
b19 /2+
1 y19 /2+
y$11 /1+
1 y10 /1+
y$10 /1+
b$11 /1+
M /4+
1 Y /3+
1 Y /2+
1 2 Y /2+
138.06
168.07
186.08
204.09
274.09
292.10
366.14
657.23

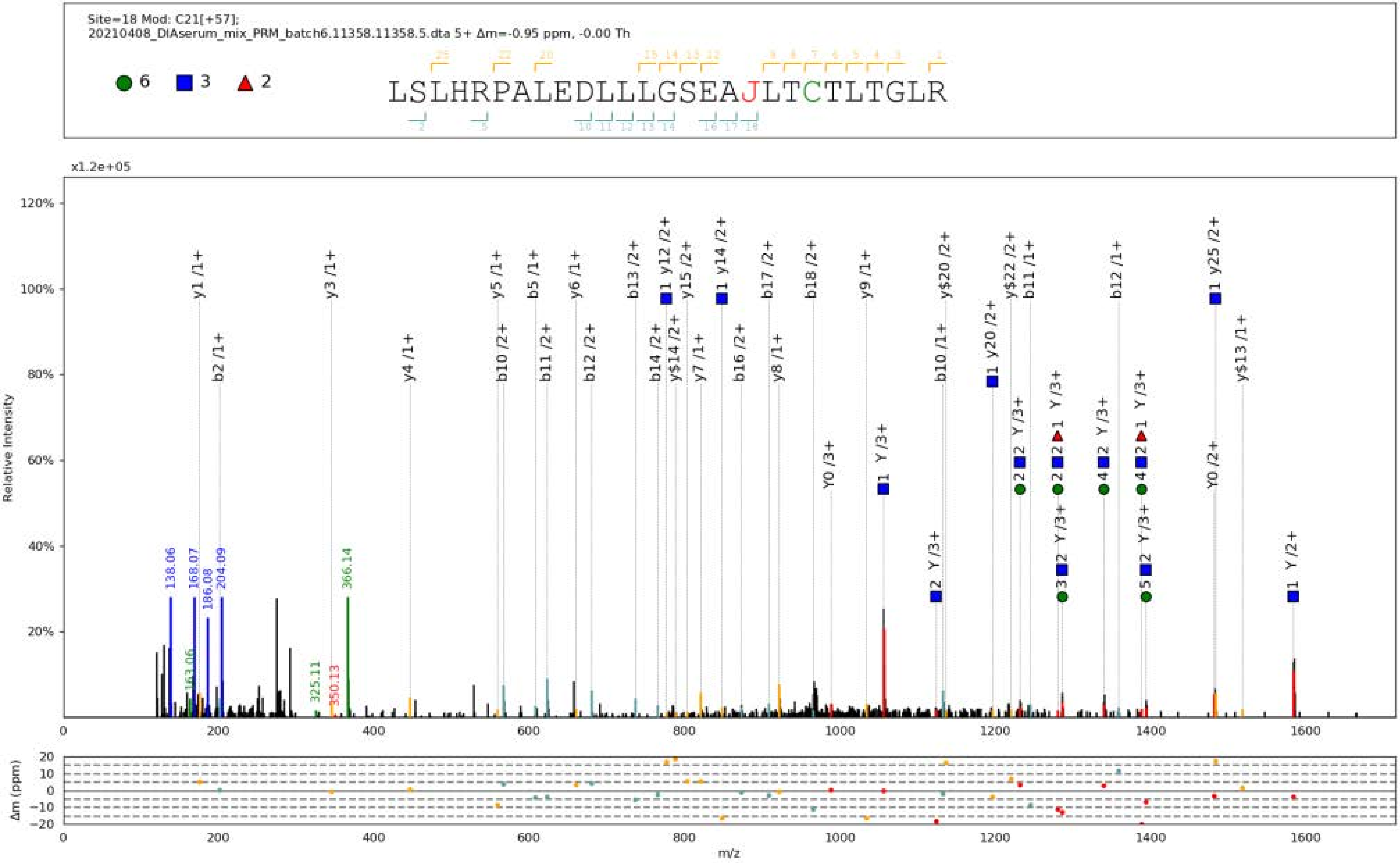
Site=18 Mod: C21[+57];
20210408_DIAserum_mix_PRM_batch6.11358.11358.5.dta 5+ Δm=-0.95 ppm, -0.00 Th
LSLHRPALEDLLLGSEAJLTCTLTGLR
x1.2e+05
Relative Intensity
m/z
Δm (ppm)

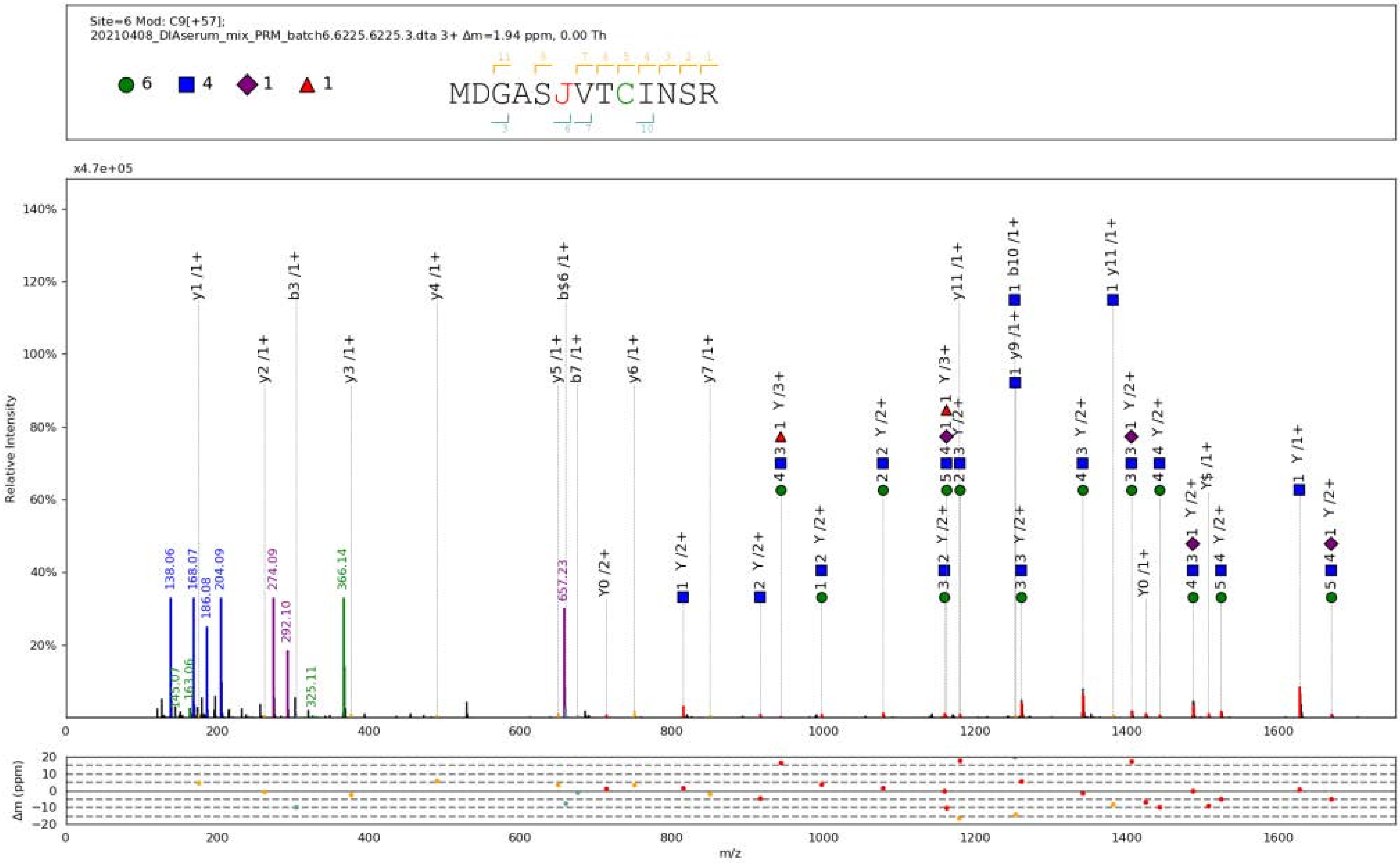

Site=6 Mod: C9[+57];
20210408_DIAserum_mix_PRM_batch6.6225.6225.3.dta 3+ Δm=1.94 ppm, 0.00 Th
6 4 1 1
MDGASJVTCINSR
x4.7e+05
Relative Intensity
m/z
Δm (ppm)

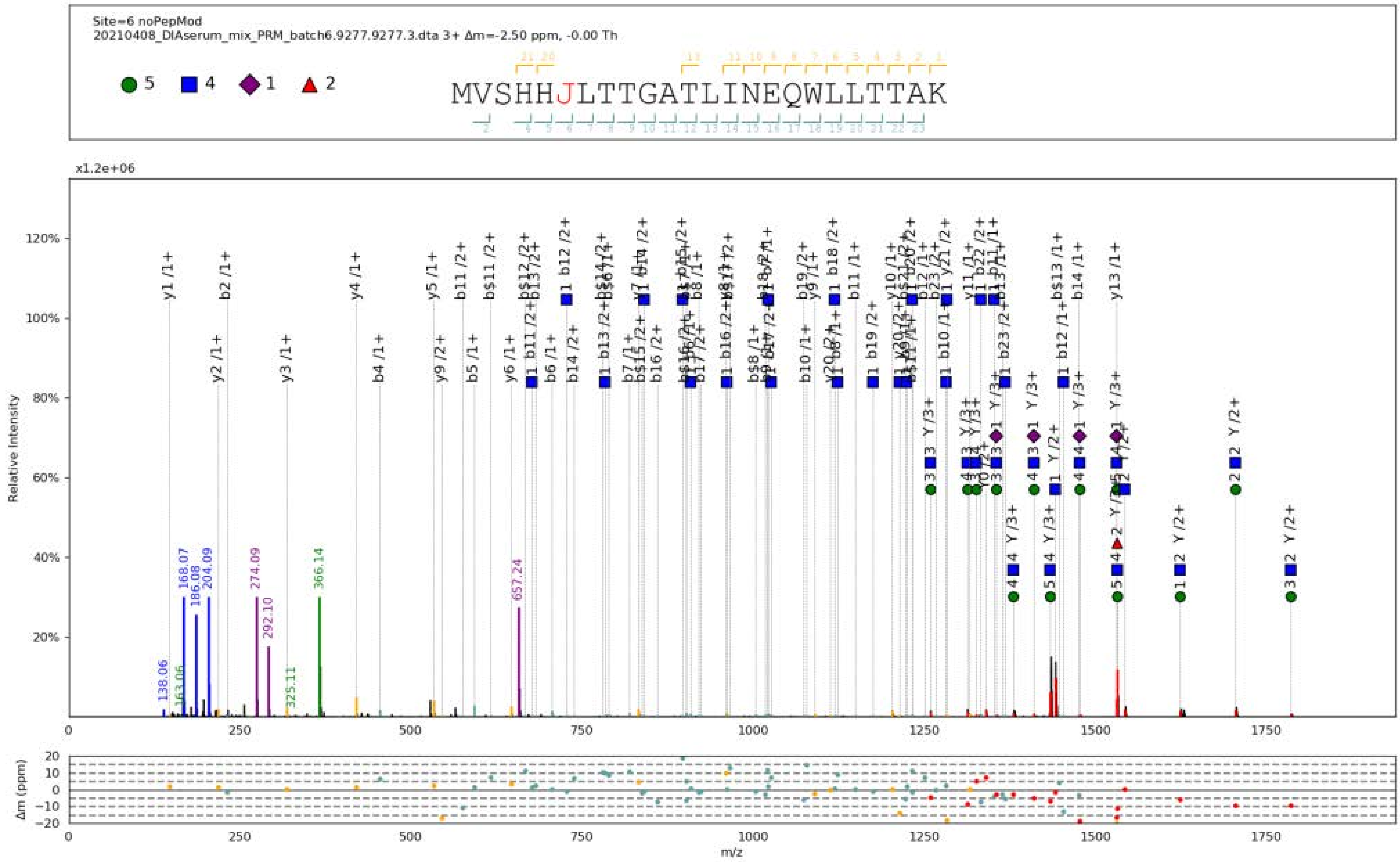
Site=6 noPepMod
20210408_DIAserum_mix_PRM_batch6.9277.9277.3.dta 3+ Δm=-2.50 ppm, -0.00 Th
5
4
1
2
MVSHHJLTTGATLINEQWLLTTAK
x1.2e+06
Relative Intensity
138.06
163.06
168.07
186.08
204.09
274.09
292.10
325.11
366.14
657.24
Δm (ppm)
m/z

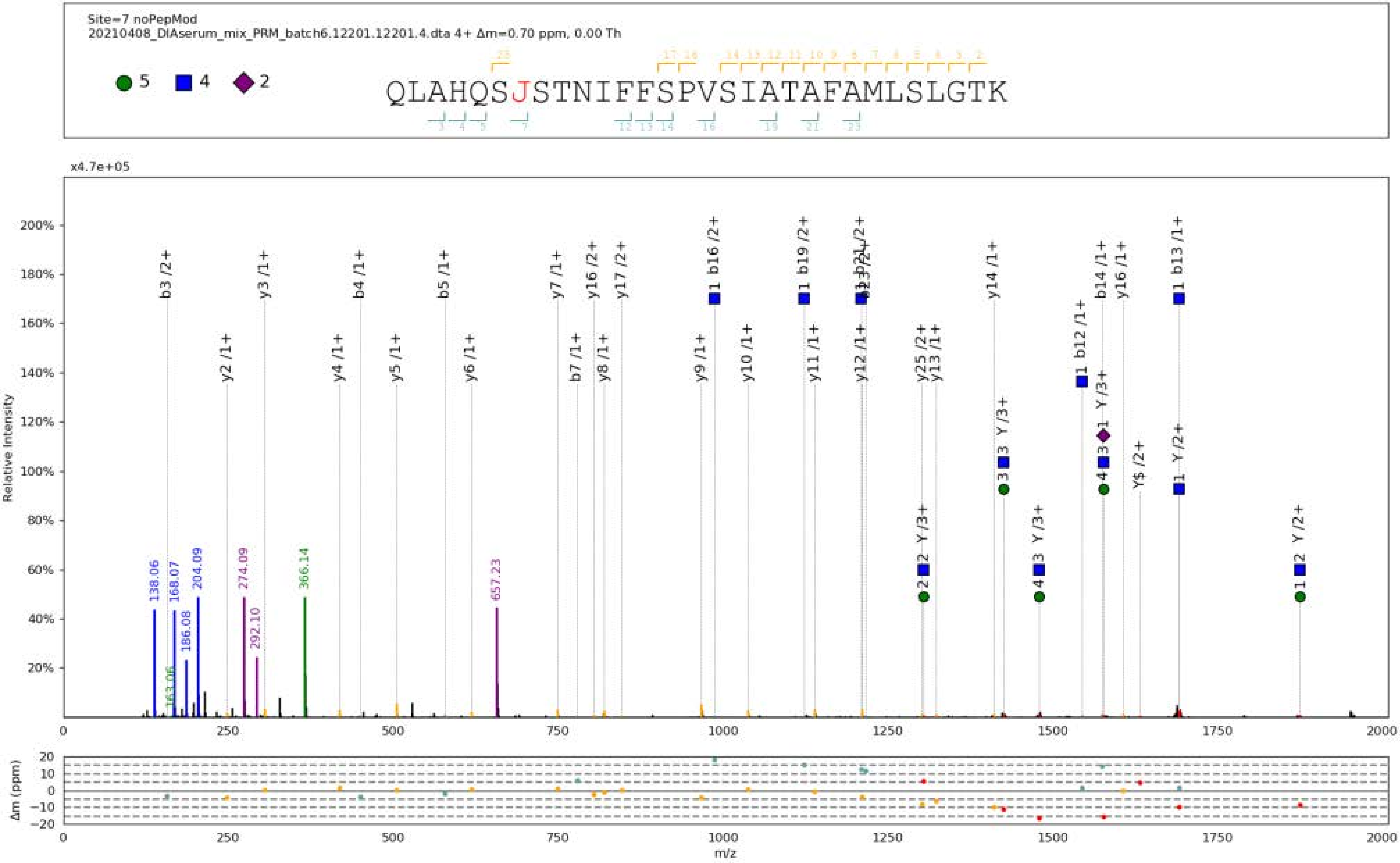

Site=7 noPepMod
20210408_DIAserum_mix_PRM_batch6.12201.12201.4.dta 4+ Δm=0.70 ppm, 0.00 Th
QLAHQSJSTNIFFSPVSIATAFAMLSLGTK
Relative Intensity
Δm (ppm)
m/z

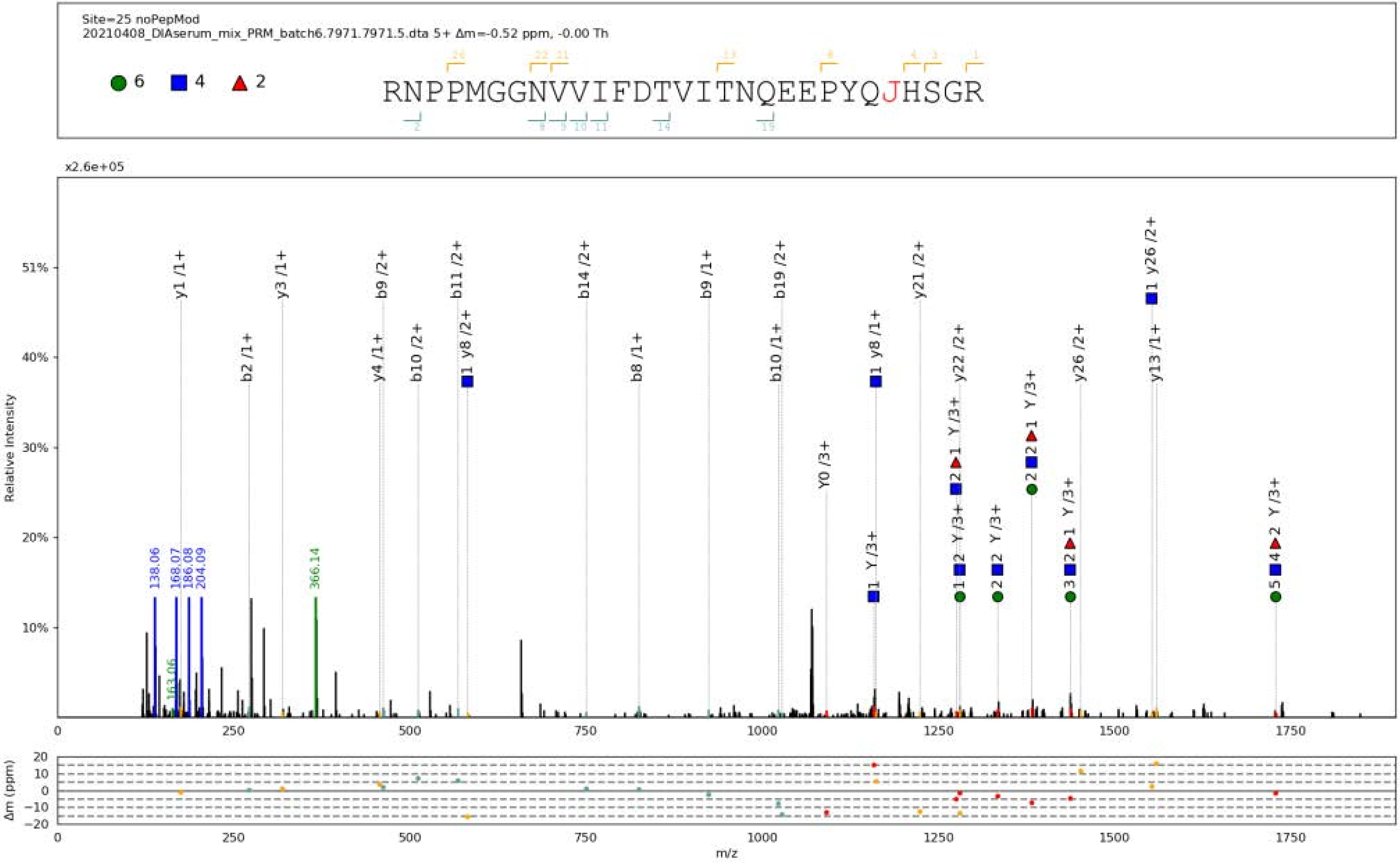
Site=25 noPepMod
20210408_DIAserum_mix_PRM_batch6.7971.7971.5.dta 5+ Δm=-0.52 ppm, -0.00 Th
6
4
2
RNPPMGGNVVIFDTVITNQEEPYQJHSGR
x2.6e+05
Relative Intensity
m/z
Δm (ppm)

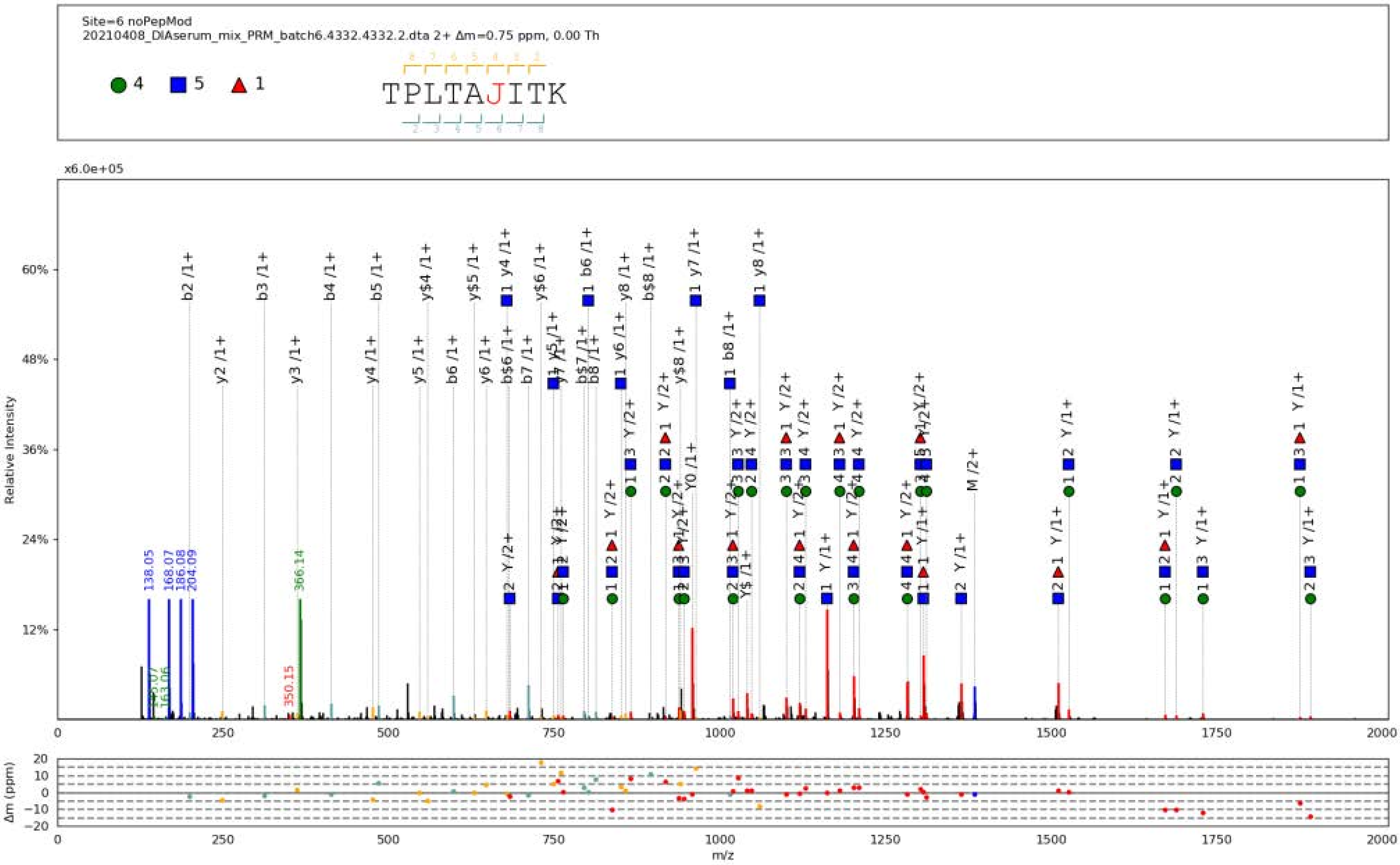

Site=6 noPepMod
20210408_DIAserum_mix_PRM_batch6.4332.4332.2.dta 2+ Δm=0.75 ppm, 0.00 Th
4
5
1
TPLTAJITK
x6.0e+05
Relative Intensity
60%
48%
36%
24%
12%
b2 /1+
y2 /1+
b3 /1+
y3 /1+
b4 /1+
y4 /1+
b5 /1+
y5 /1+
y$4 /1+
b6 /1+
y$5 /1+
y6 /1+
b$6 /1+
1 y4 /1+
b7 /1+
y$6 /1+
1 b6 /1+
b$7 /1+
b8 /1+
1 y6 /1+
y8 /1+
b$8 /1+
y$8 /1+
1 y7 /1+
1 b8 /1+
1 y8 /1+
Y0 /1+
Y$ /1+
M /2+
138.05
168.07
186.08
204.09
366.14
350.15
163.06
0
250
500
750
1000
1250
1500
1750
2000
Δm (ppm)
20
10
0
−10
−20
m/z

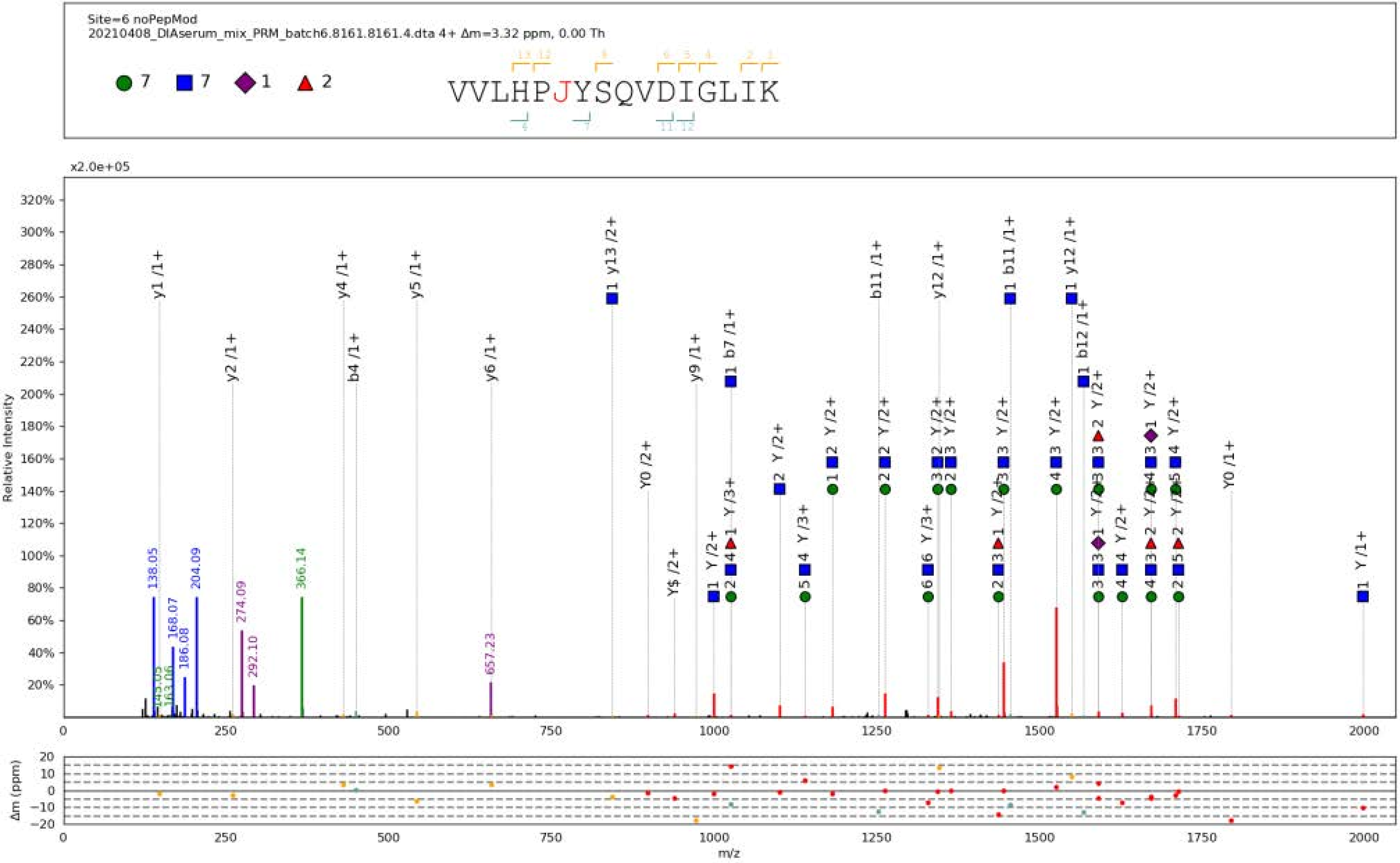

Site=6 noPepMod
20210408_DIAserum_mix_PRM_batch6.8161.8161.4.dta 4+ Δm=3.32 ppm, 0.00 Th
VVLHPJYSQVDIGLIK
x2.0e+05
Relative Intensity
m/z
Δm (ppm)

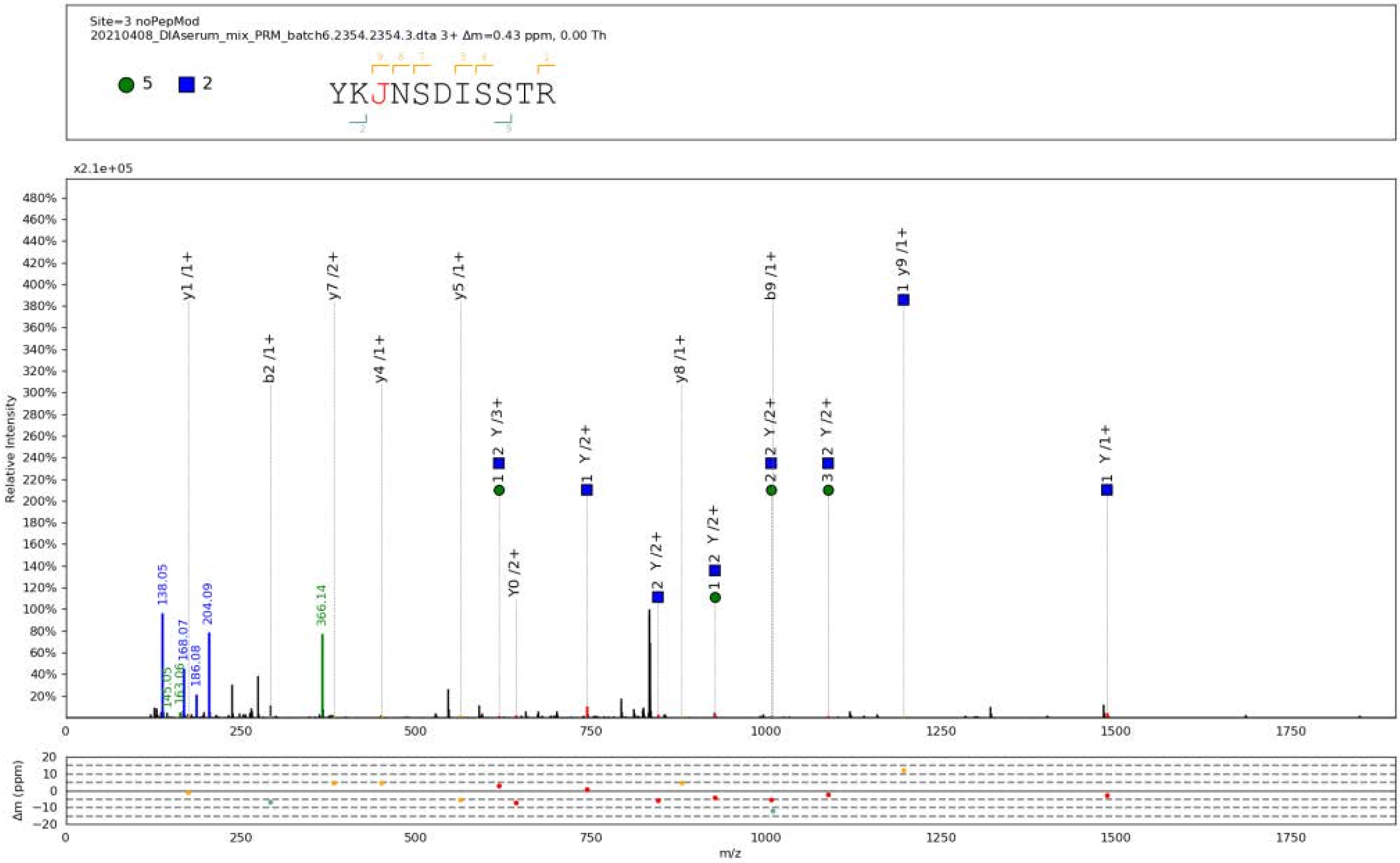

Site=3 noPepMod
20210408_DIAserum_mix_PRM_batch6.2354.2354.3.dta 3+ Δm=0.43 ppm, 0.00 Th
5
2
YKJNSDISSTR
x2.1e+05
Relative Intensity
y1 /1+
b2 /1+
y7 /2+
y4 /1+
y5 /1+
1 2 Y /3+
Y0 /2+
1 Y /2+
2 Y /2+
1 2 Y /2+
y8 /1+
b9 /1+
2 2 Y /2+
3 2 Y /2+
1 y9 /1+
1 Y /1+
138.05
145.05
163.06
168.07
186.08
204.09
366.14
m/z
Δm (ppm)

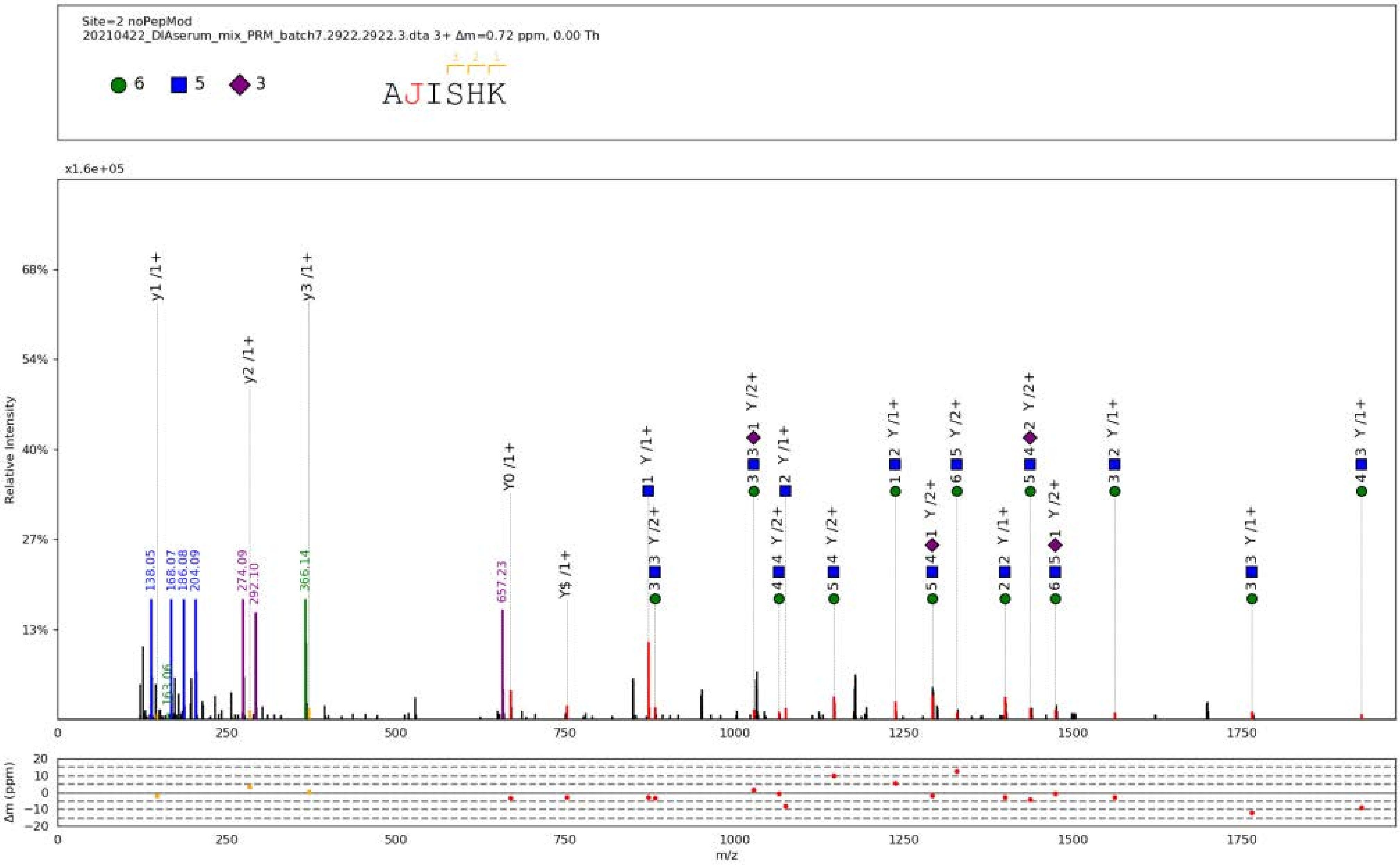

Site=2 noPepMod
20210422_DIAserum_mix_PRM_batch7.2922.2922.3.dta 3+ Δm=0.72 ppm, 0.00 Th
6
5
3
AJISHK
x1.6e+05
Relative Intensity
y1 /1+
y2 /1+
y3 /1+
Y0 /1+
Y$ /1+
138.05
163.06
168.07
186.08
204.09
274.09
292.10
366.14
657.23
m/z
Δm (ppm)

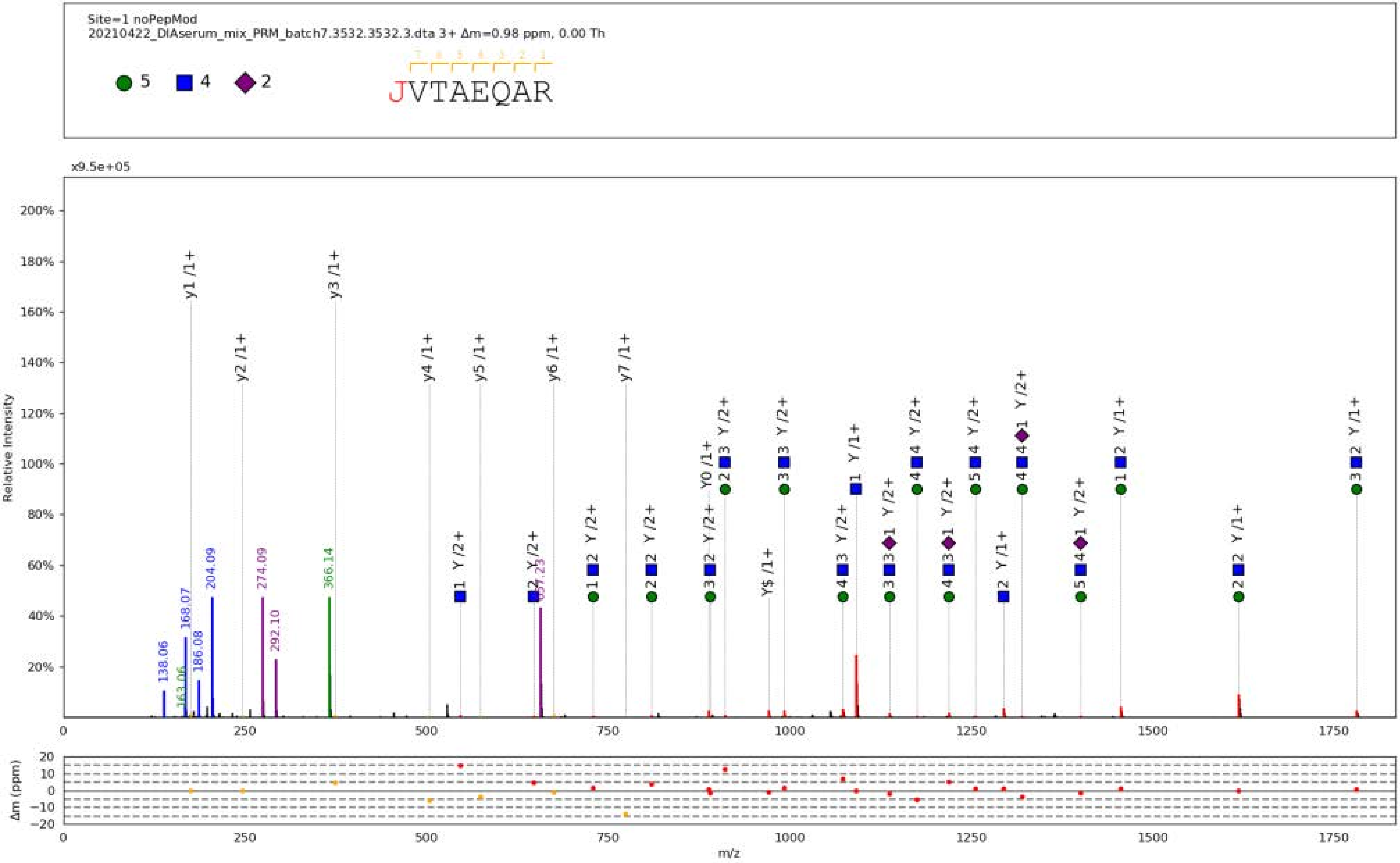
Site=1 noPepMod
20210422_DIAserum_mix_PRM_batch7.3532.3532.3.dta 3+ Δm=0.98 ppm, 0.00 Th
5
4
2
JVTAEQAR
x9.5e+05
Relative Intensity
y1 /1+
y2 /1+
y3 /1+
y4 /1+
y5 /1+
y6 /1+
y7 /1+
Y0 /1+
Y$ /1+
138.06
163.06
168.07
186.08
204.09
274.09
292.10
366.14
m/z
Δm (ppm)

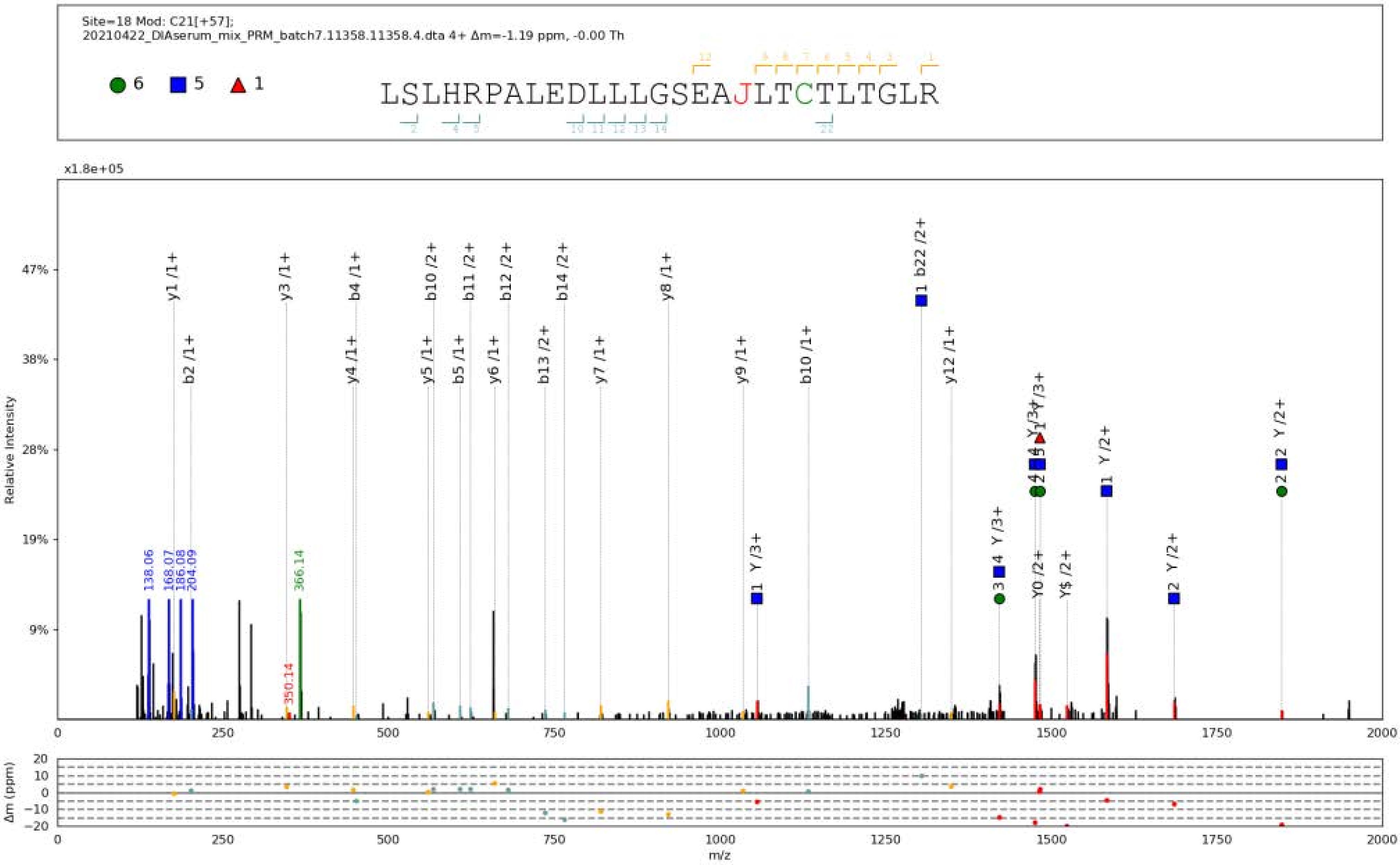
Site=18 Mod: C21[+57];
20210422_DIAserum_mix_PRM_batch7.11358.11358.4.dta 4+ Δm=-1.19 ppm, -0.00 Th
6
5
1
LSLHRPALEDLLLGSEAJLTCTLTGLR
x1.8e+05
Relative Intensity
47%
38%
28%
19%
9%
y1 /1+
b2 /1+
y3 /1+
b4 /1+
y4 /1+
b10 /2+
y5 /1+
b11 /2+
b5 /1+
b12 /2+
y6 /1+
b13 /2+
b14 /2+
y7 /1+
y8 /1+
y9 /1+
b10 /1+
b22 /2+
y12 /1+
Y /3+
Y0 /2+
Y$ /2+
Y /2+
138.06
168.07
186.08
204.09
366.14
350.14
0
250
500
750
1000
1250
1500
1750
2000
m/z
Δm (ppm)
20
10
0
-10
-20

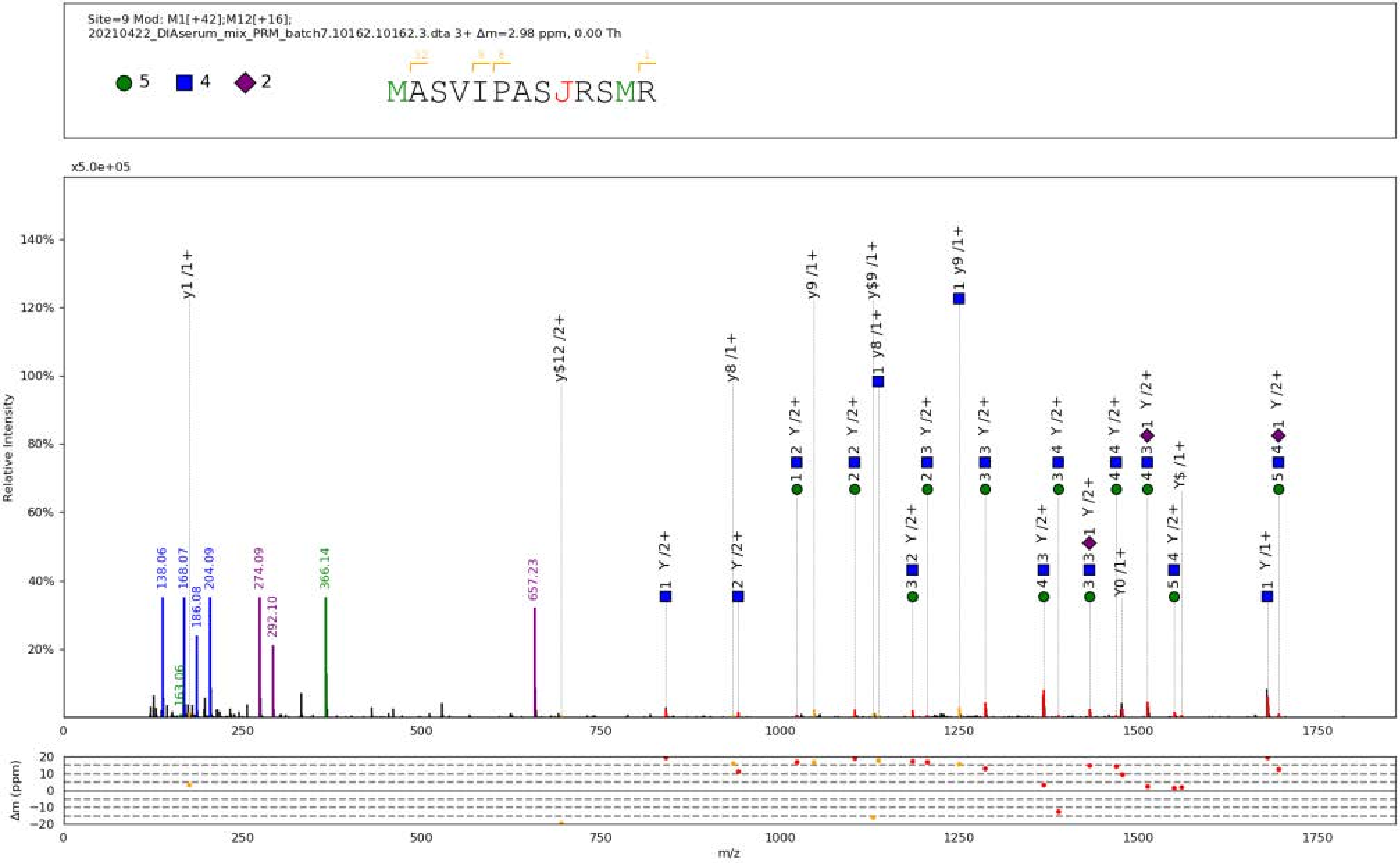

Site=9 Mod: M1[+42];M12[+16];
20210422_DIAserum_mix_PRM_batch7.10162.10162.3.dta 3+ Δm=2.98 ppm, 0.00 Th
5
4
2
MASVIPASJRSMR
x5.0e+05
Relative Intensity
y1 /1+
y$12 /2+
y8 /1+
y9 /1+
y$9 /1+
1 y8 /1+
1 y9 /1+
Y0 /1+
Y$ /1+
1 Y /1+
138.06
168.07
186.08
204.09
163.06
274.09
292.10
366.14
657.23
Δm (ppm)
m/z

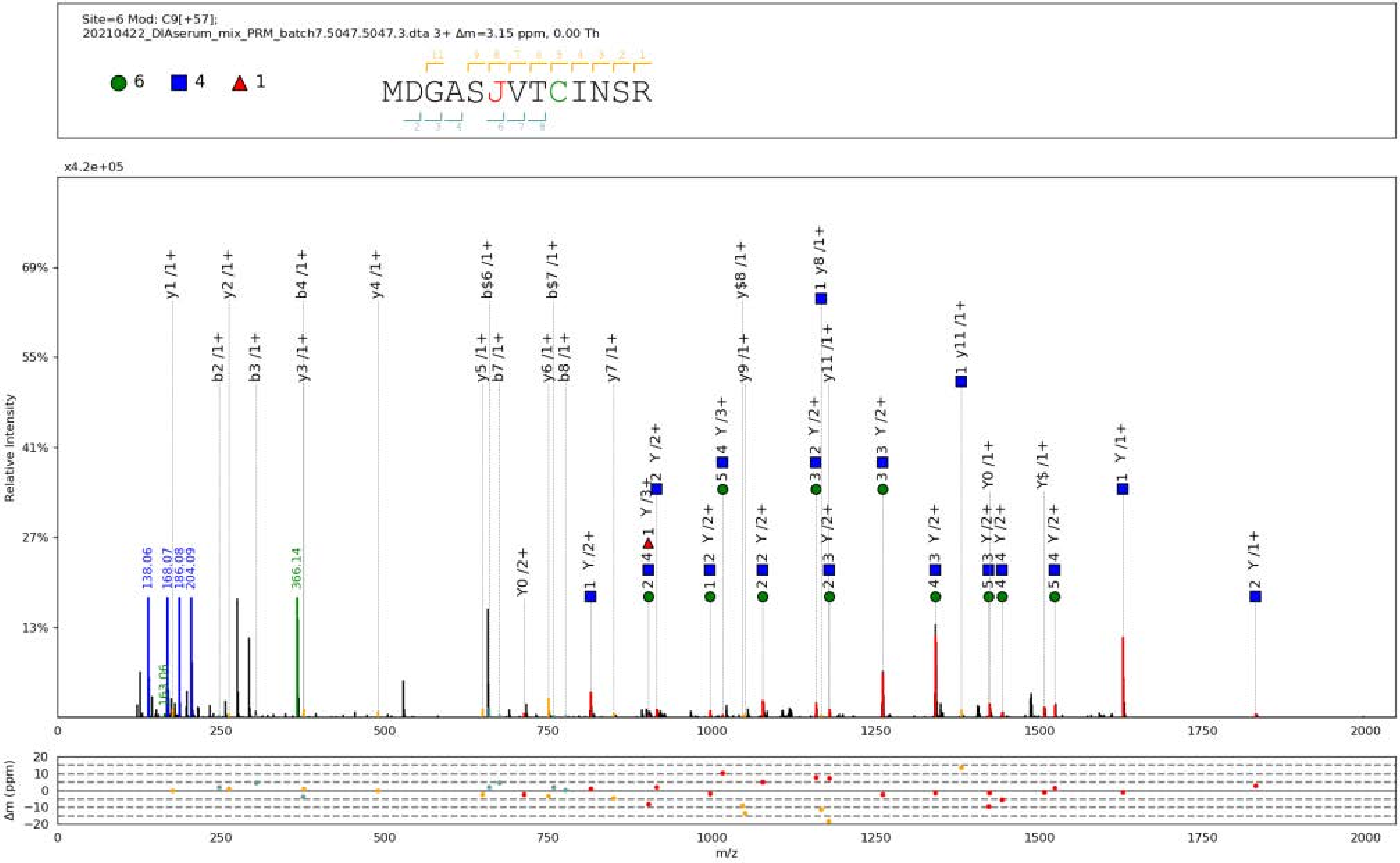
Site=6 Mod: C9[+57];
20210422_DIAserum_mix_PRM_batch7.5047.5047.3.dta 3+ Δm=3.15 ppm, 0.00 Th
6
4
1
MDGASJVTCINSR
x4.2e+05
Relative Intensity
69%
55%
41%
27%
13%
y1 /1+
y2 /1+
b2 /1+
b3 /1+
b4 /1+
y3 /1+
y4 /1+
b$6 /1+
y5 /1+
b7 /1+
b$7 /1+
y6 /1+
b8 /1+
y7 /1+
Y0 /2+
y$8 /1+
y9 /1+
1 y8 /1+
y11 /1+
1 y11 /1+
Y0 /1+
Y$ /1+
138.06
168.07
186.08
204.09
366.14
163.06
m/z
Δm (ppm)
0
250
500
750
1000
1250
1500
1750
2000

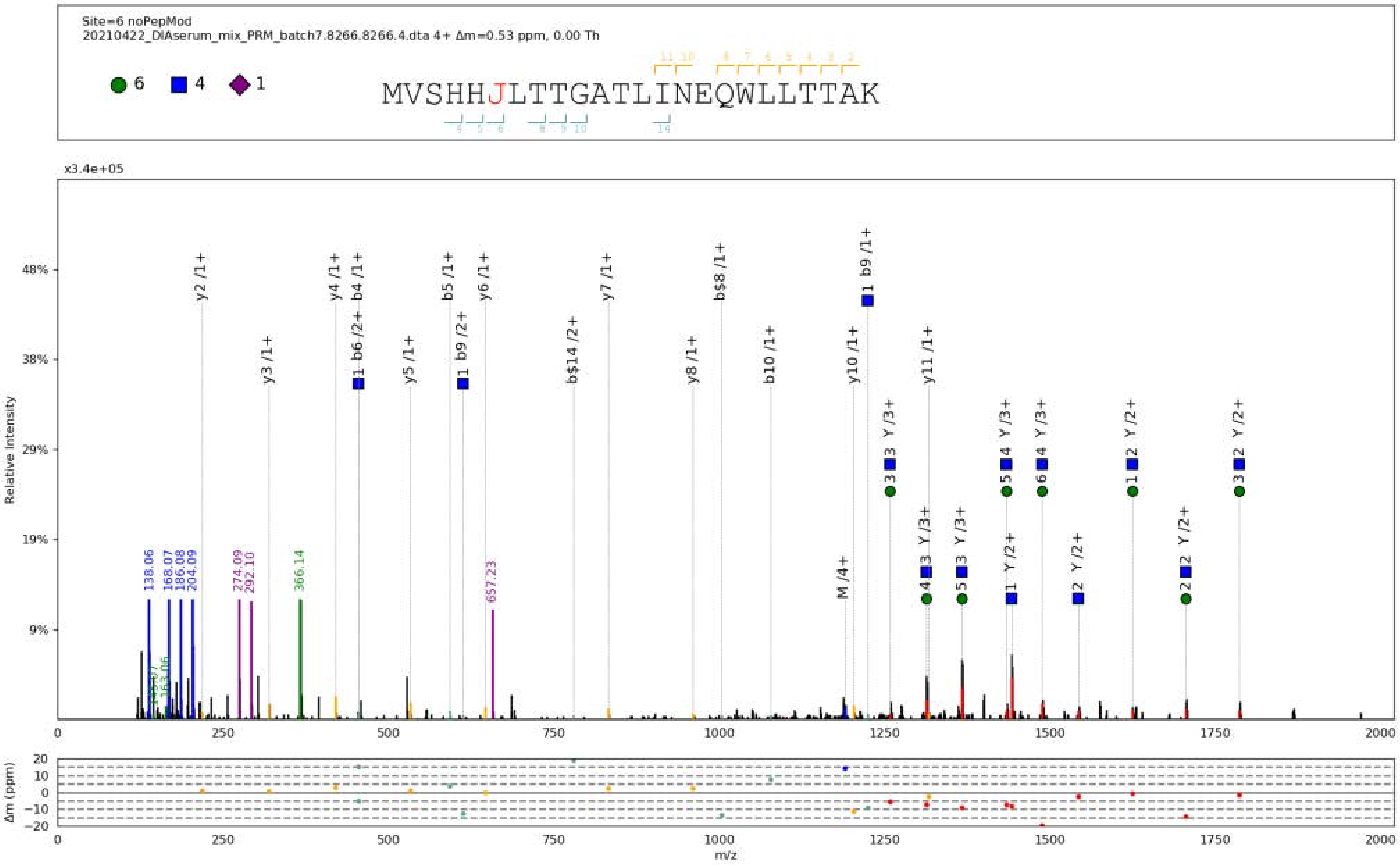
Site=6 noPepMod
20210422_DIAserum_mix_PRM_batch7.8266.8266.4.dta 4+ Δm=0.53 ppm, 0.00 Th
6 4 1
MVSHHJLTTGATLINEQWLLTTAK
x3.4e+05
Relative Intensity
m/z
Δm (ppm)

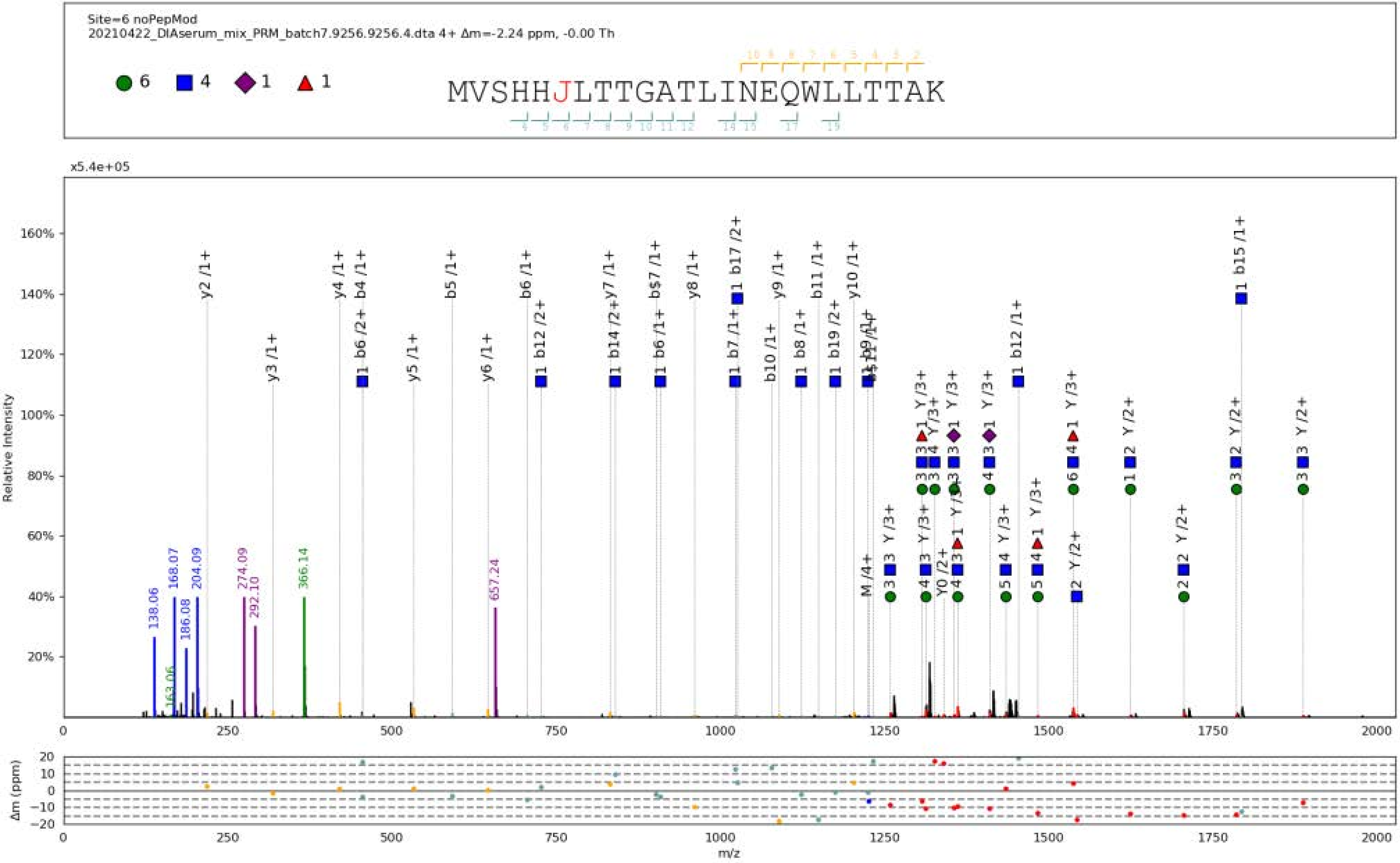
Site=6 noPepMod
20210422_DIAserum_mix_PRM_batch7.9256.9256.4.dta 4+ Δm=-2.24 ppm, -0.00 Th
6 4 1 1
MVSHHJLTTGATLINEQWLLTTAK
x5.4e+05
Relative Intensity
m/z
Δm (ppm)

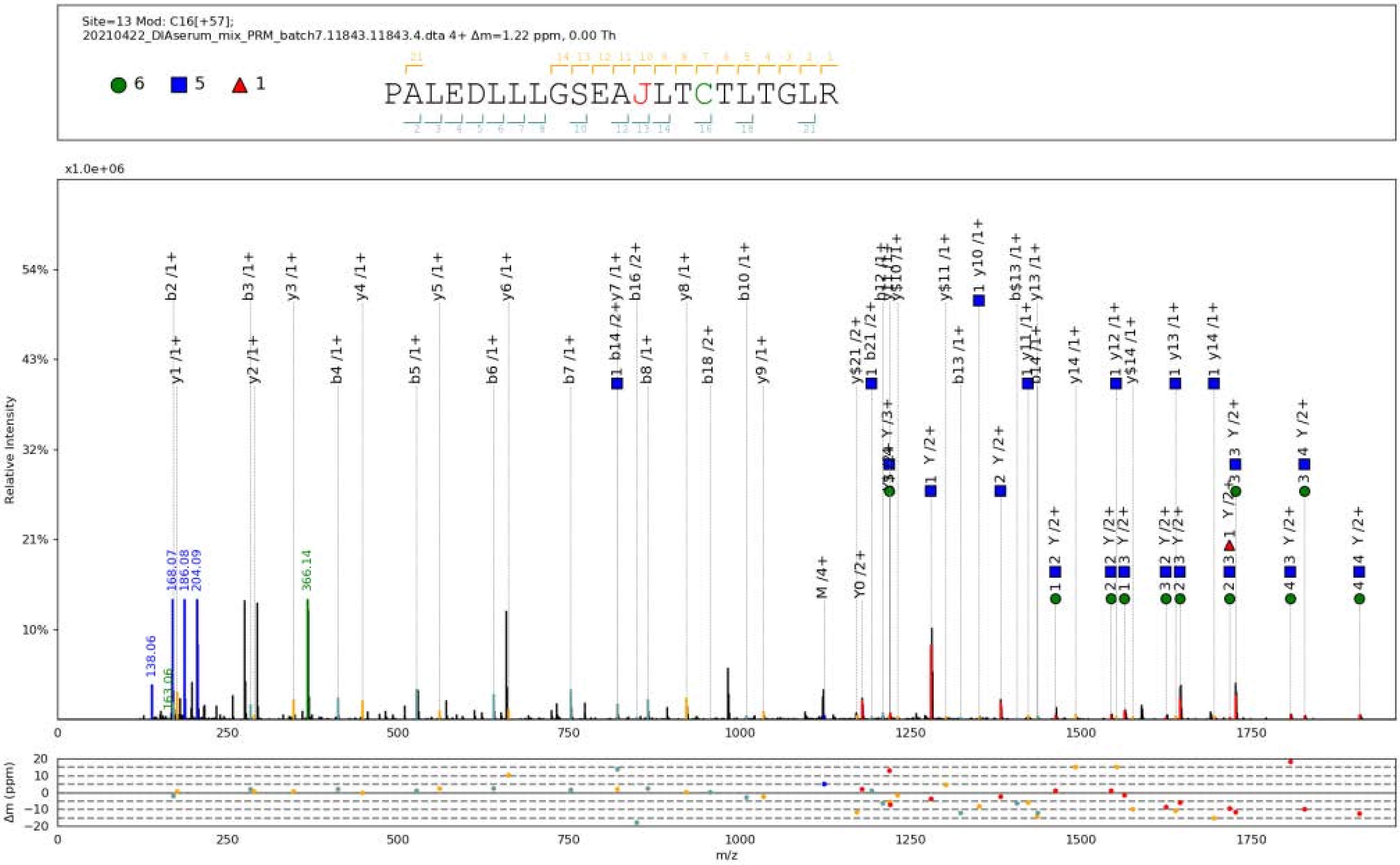
Site=13 Mod: C16[+57];
20210422_DIAserum_mix_PRM_batch7.11843.11843.4.dta 4+ Δm=1.22 ppm, 0.00 Th
PALEDLLLGSEAJLTCTLTGLR
x1.0e+06
Relative Intensity
m/z
Δm (ppm)

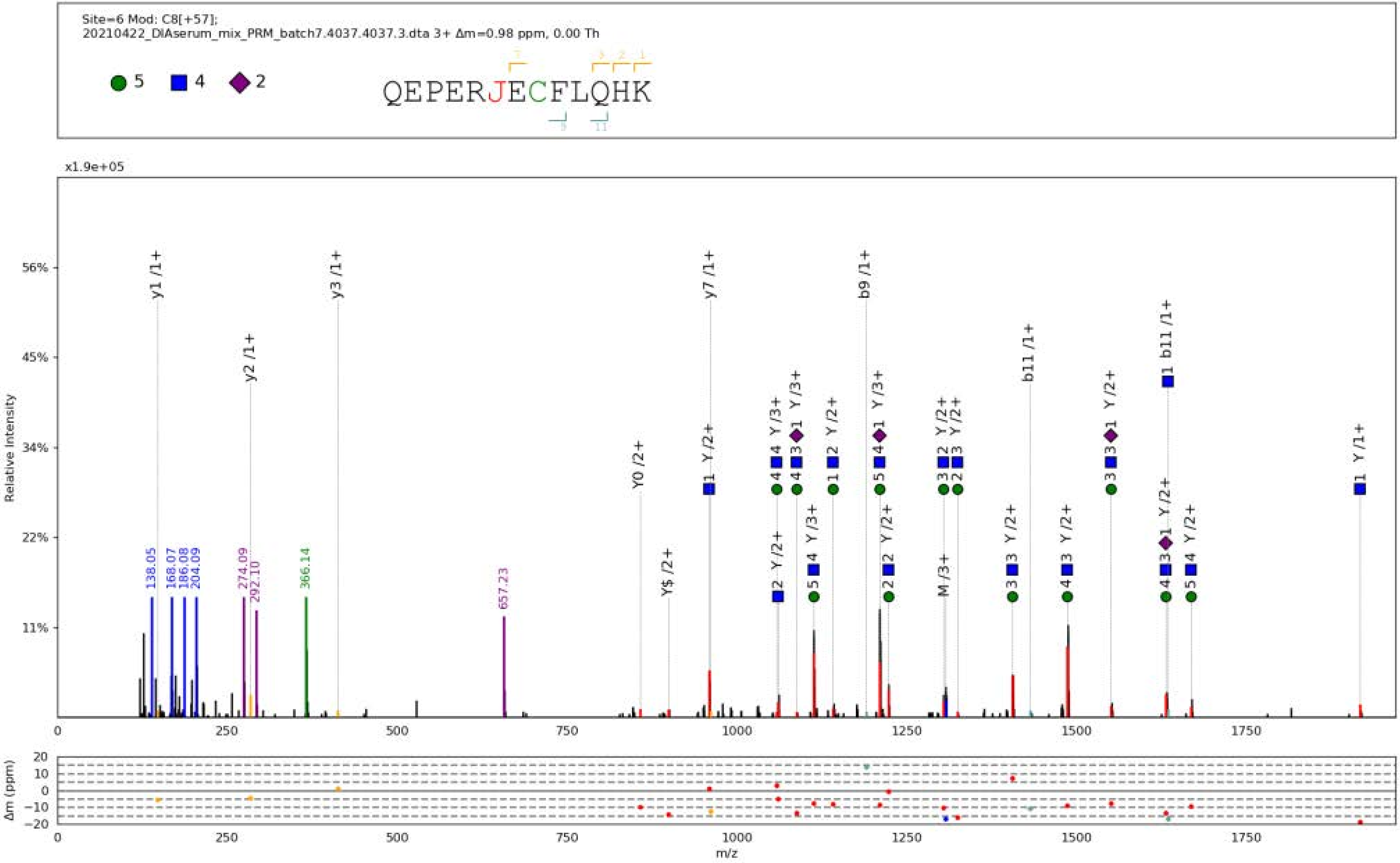
Site=6 Mod: C8[+57];
20210422_DIAserum_mix_PRM_batch7.4037.4037.3.dta 3+ Δm=0.98 ppm, 0.00 Th
5
4
2
QEPERJECFLQHK
x1.9e+05
Relative Intensity
y1 /1+
y2 /1+
y3 /1+
y7 /1+
b9 /1+
b11 /1+
Y0 /2+
Y$ /2+
M /3+
138.05
168.07
186.08
204.09
274.09
292.10
366.14
657.23
m/z
Δm (ppm)

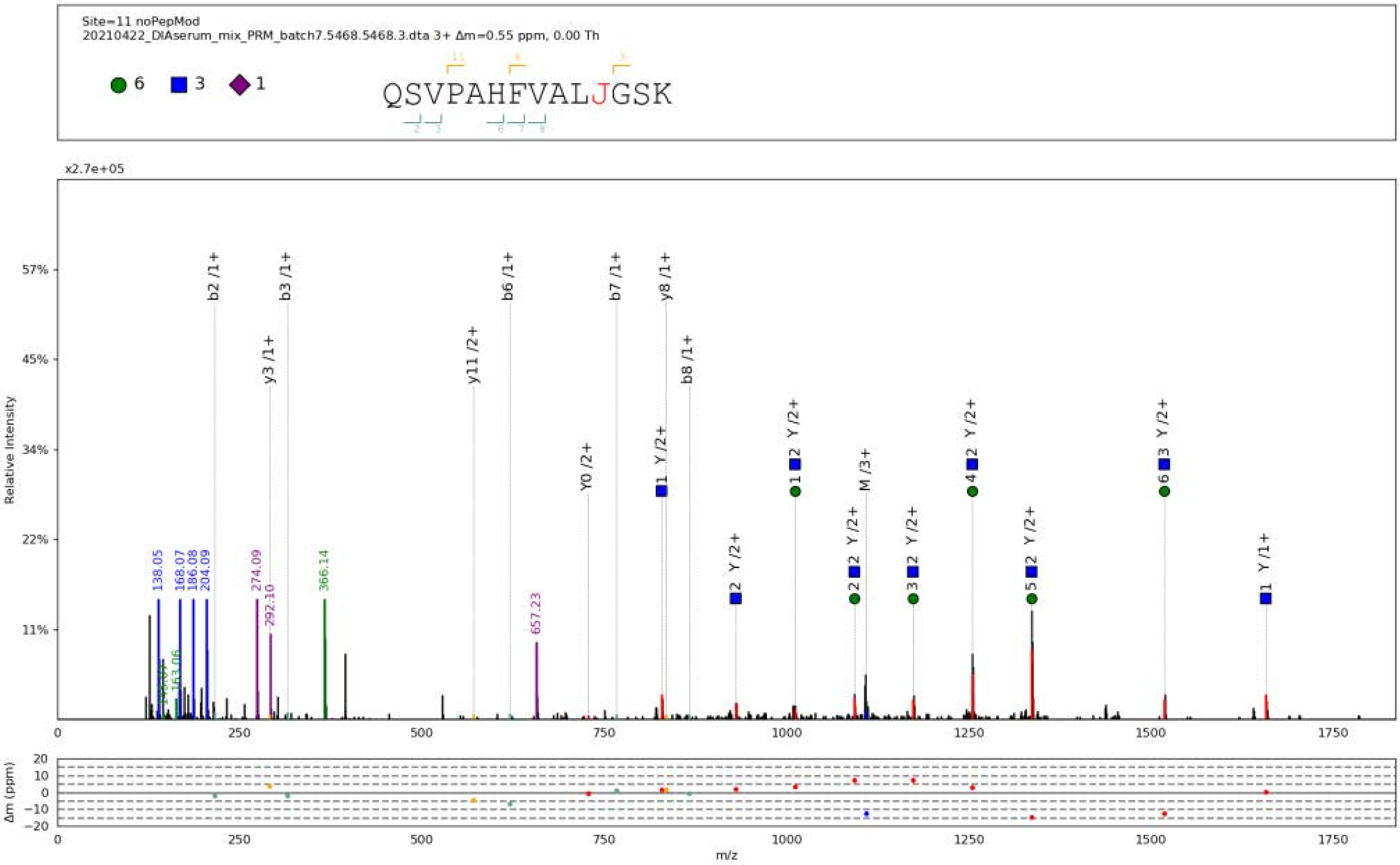
Site=11 noPepMod
20210422_DIAserum_mix_PRM_batch7.5468.5468.3.dta 3+ Δm=0.55 ppm, 0.00 Th
6
3
1
QSVPAHFVALJGSK
x2.7e+05
Relative Intensity
57%
45%
34%
22%
11%
b2 /1+
b3 /1+
y3 /1+
y11 /2+
b6 /1+
b7 /1+
y8 /1+
b8 /1+
Y0 /2+
M /3+
Y /2+
Y /1+
138.05
168.07
186.08
204.09
274.09
292.10
366.14
163.06
657.23
m/z
Δm (ppm)

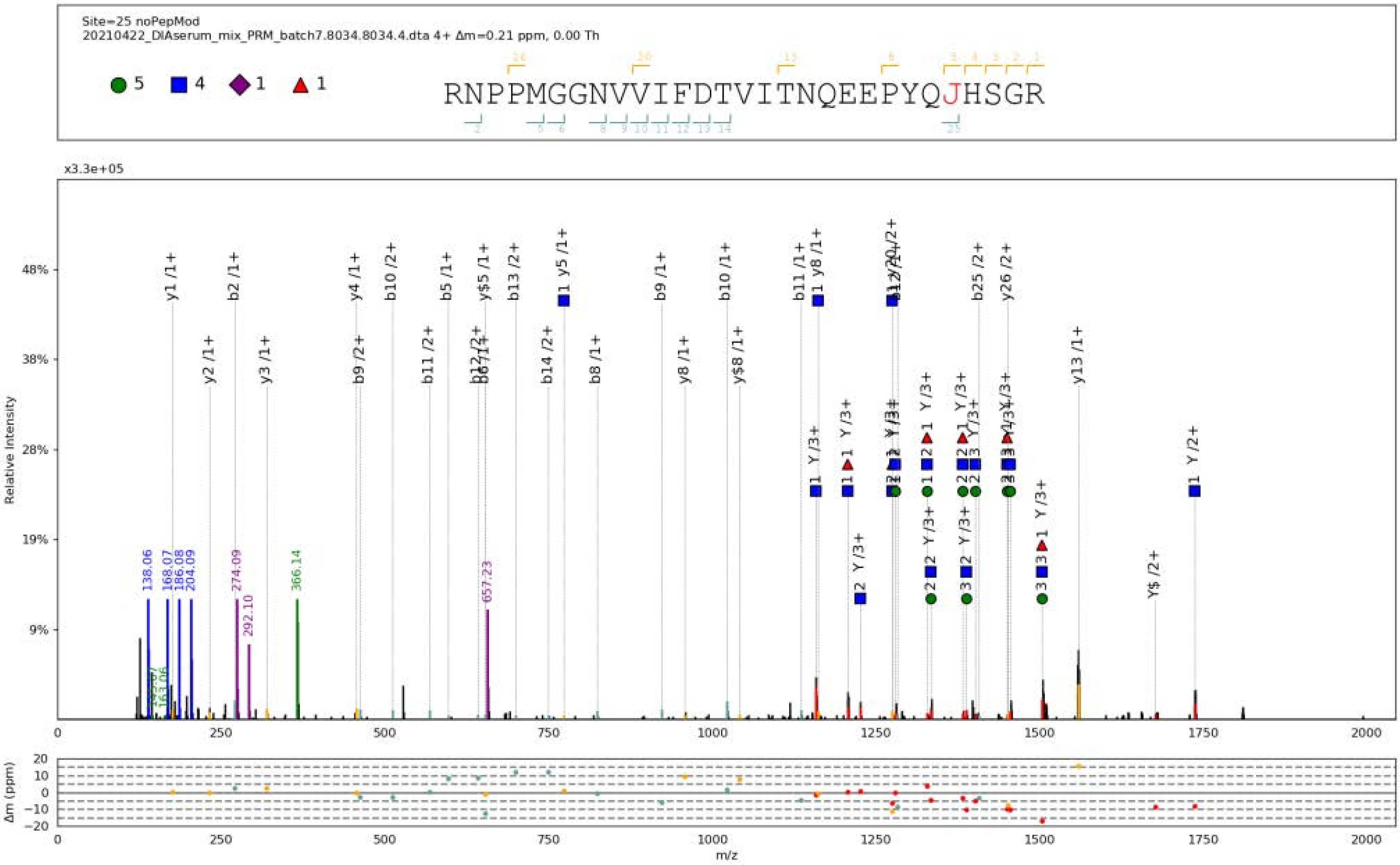
Site=25 noPepMod
20210422_DIAserum_mix_PRM_batch7.8034.8034.4.dta 4+ Δm=0.21 ppm, 0.00 Th
RNPPMGGNVVIFDTVITNQEEPYQJHSGR
x3.3e+05
Relative Intensity
Δm (ppm)
m/z

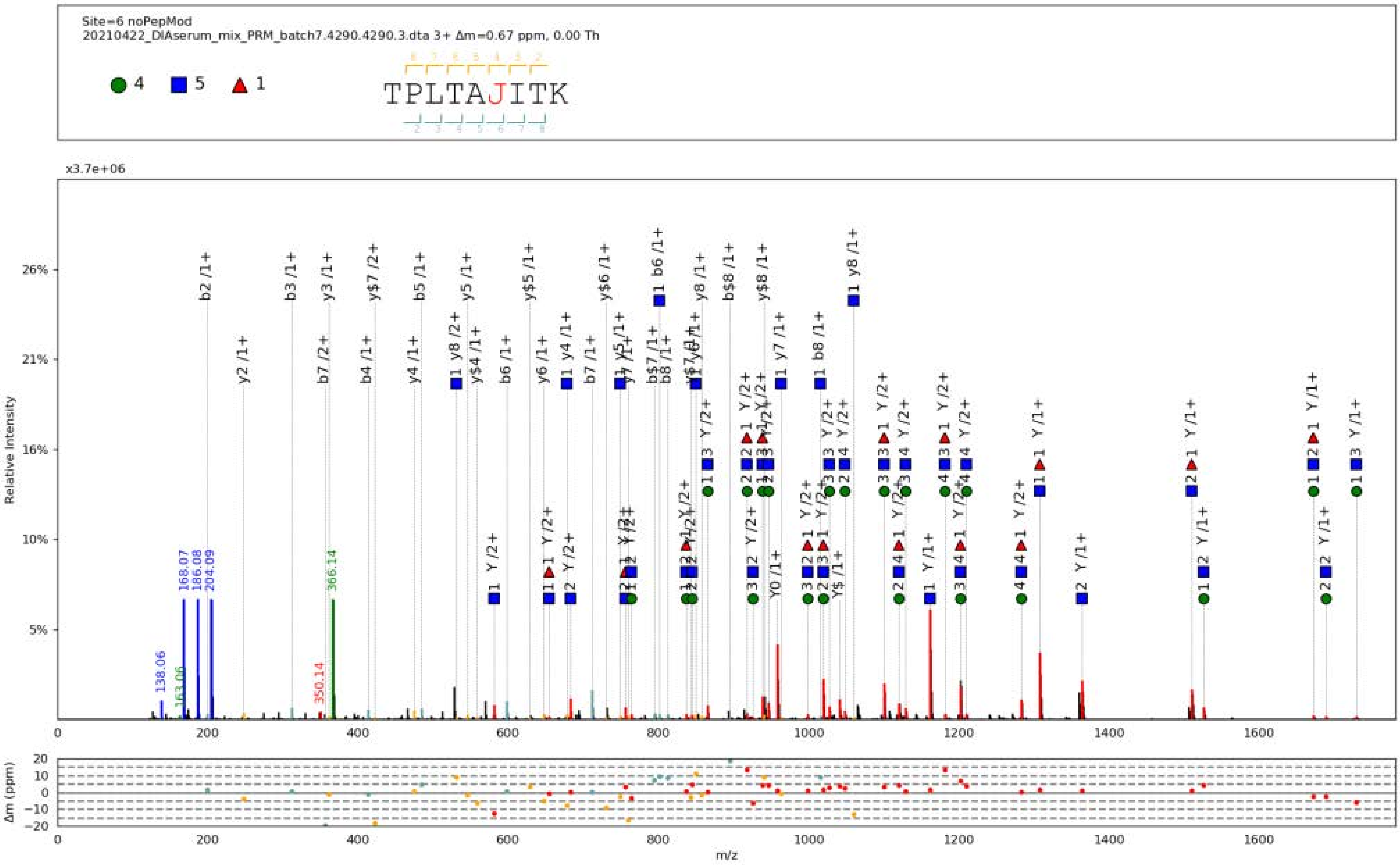

Site=6 noPepMod
20210422_DIAserum_mix_PRM_batch7.4290.4290.3.dta 3+ Δm=0.67 ppm, 0.00 Th
TPLTAJITK
x3.7e+06
Relative Intensity
m/z
Δm (ppm)

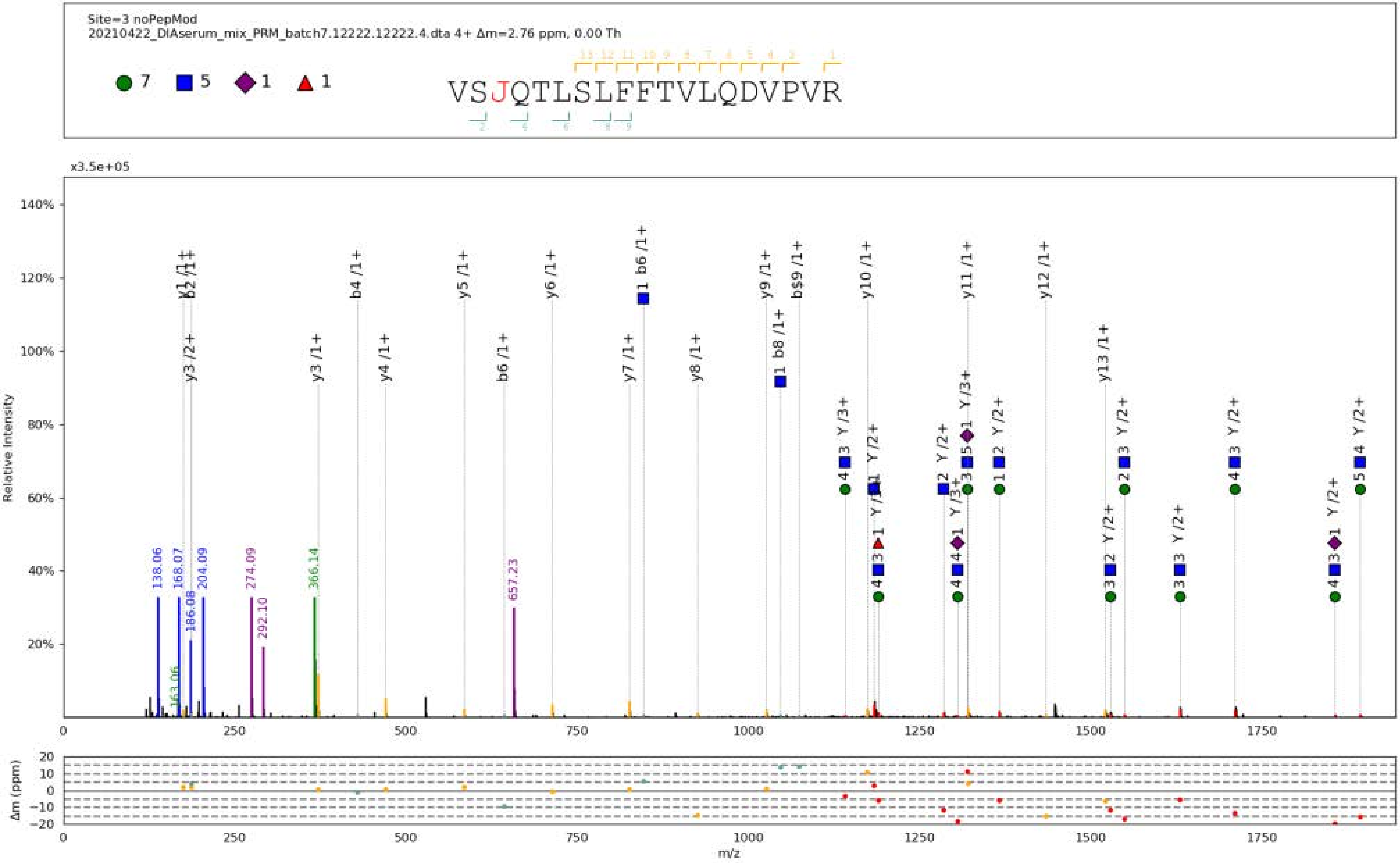
Site=3 noPepMod
20210422_DIAserum_mix_PRM_batch7.12222.12222.4.dta 4+ Δm=2.76 ppm, 0.00 Th
VSJQTLSLFFTVLQDVPVR
x3.5e+05
Relative Intensity
m/z
Δm (ppm)

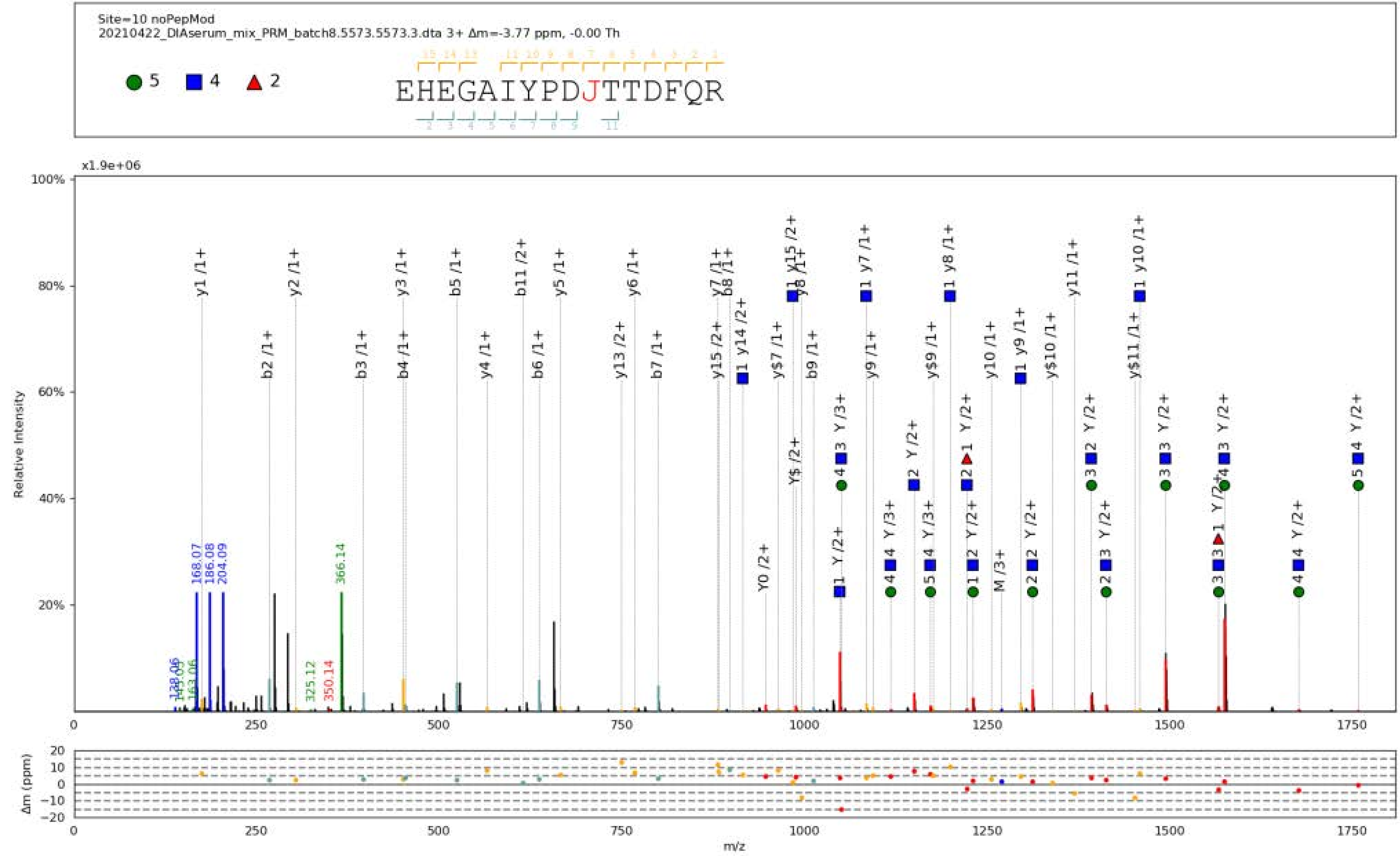

Site=10 noPepMod
20210422_DIAserum_mix_PRM_batch8.5573.5573.3.dta 3+ Δm=-3.77 ppm, -0.00 Th
5
4
2
EHEGAIYPDJTTDFQR
x1.9e+06
Relative Intensity
Δm (ppm)
m/z

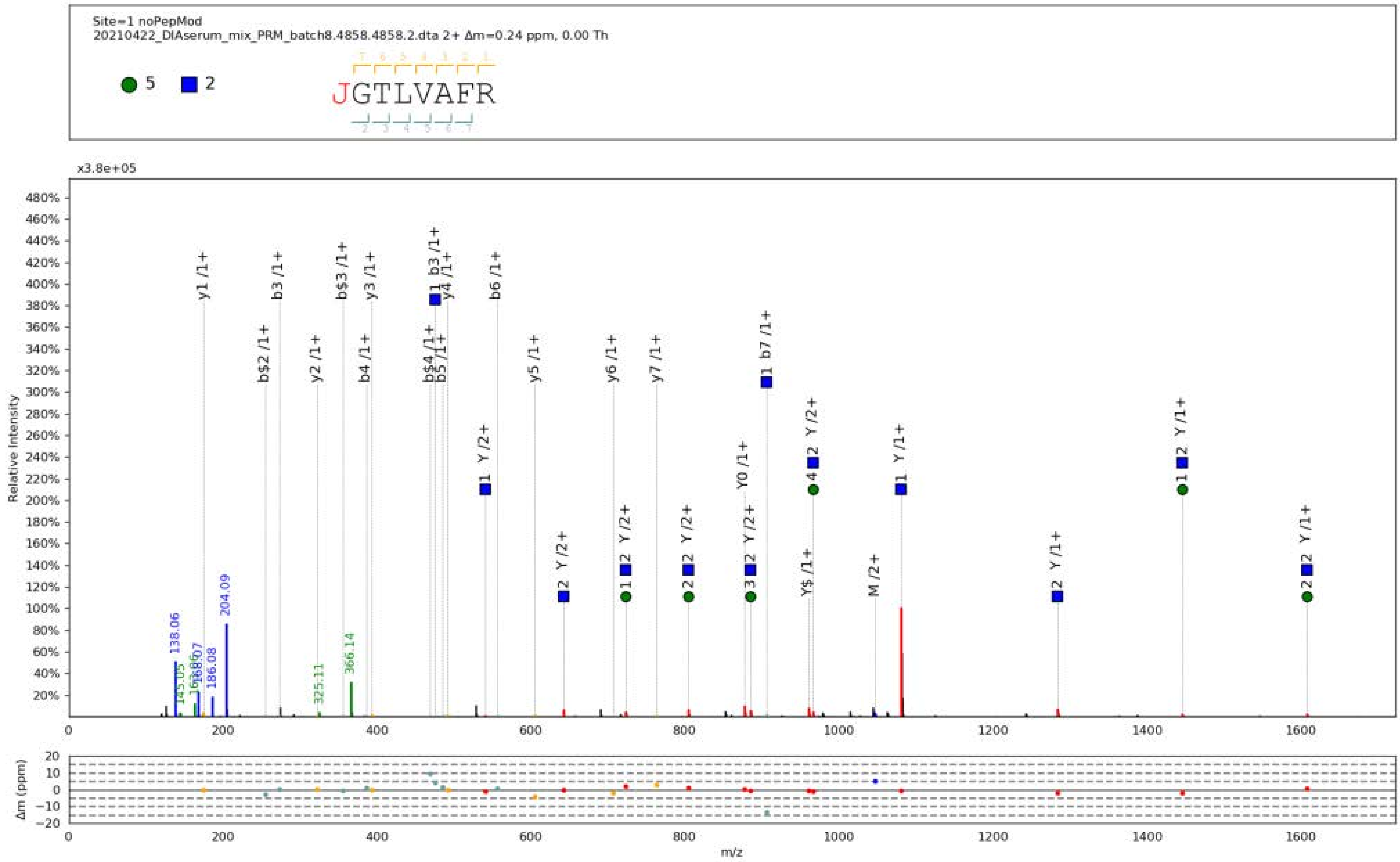

Site=1 noPepMod
20210422_DIAserum_mix_PRM_batch8.4858.4858.2.dta 2+ Δm=0.24 ppm, 0.00 Th
5
2
JGTLVAFR
x3.8e+05
Relative Intensity
m/z
Δm (ppm)

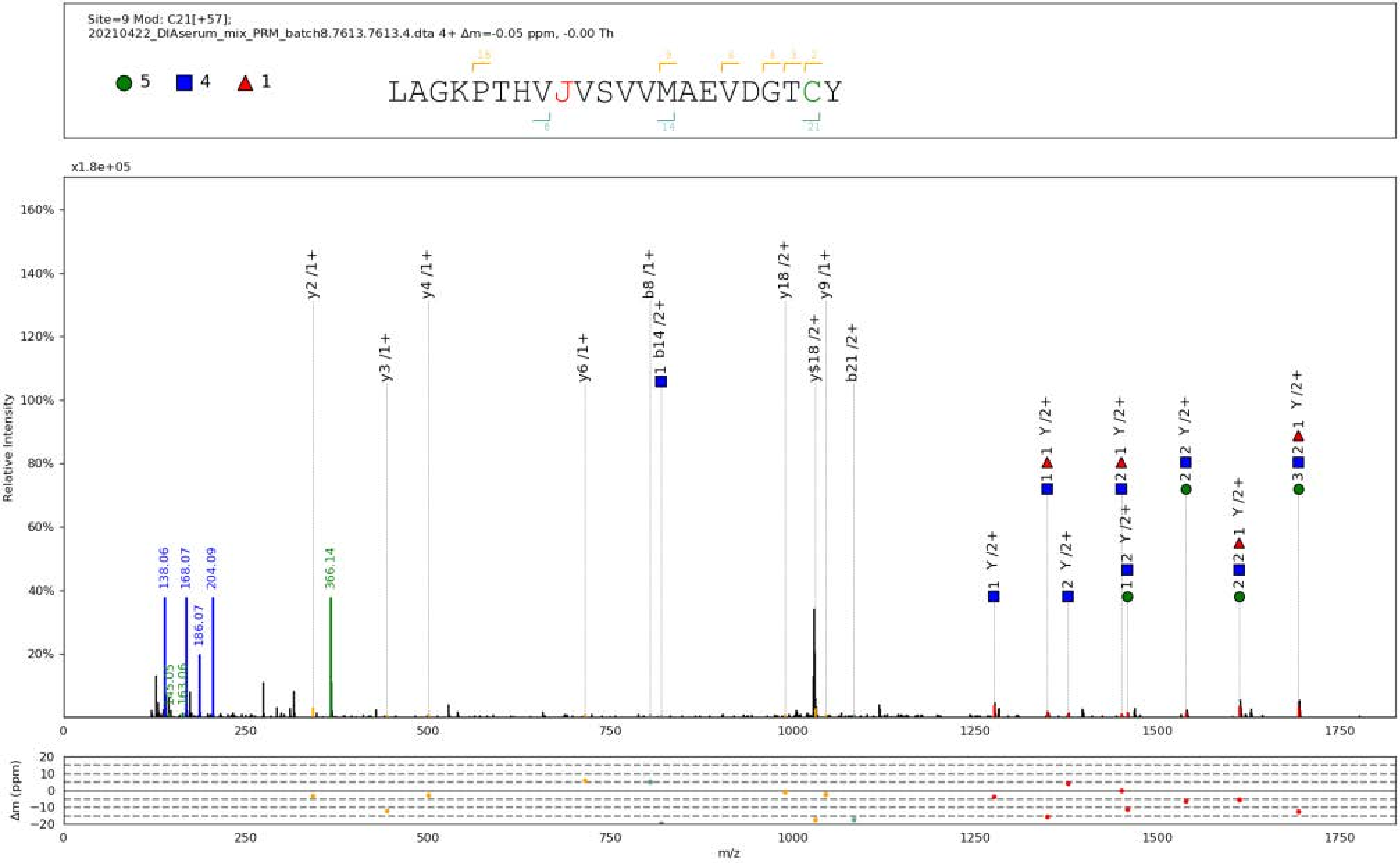

Site=9 Mod: C21[+57];
20210422_DIAserum_mix_PRM_batch8.7613.7613.4.dta 4+ Δm=-0.05 ppm, -0.00 Th
5 4 1
LAGKPTHVJVSVVMAEVDGTCY
x1.8e+05
Relative Intensity
y2 /1+
y3 /1+
y4 /1+
y6 /1+
b8 /1+
b14 /2+
y18 /2+
y$18 /2+
y9 /1+
b21 /2+
138.06
168.07
186.07
204.09
366.14
145.05
163.06
m/z
Δm (ppm)

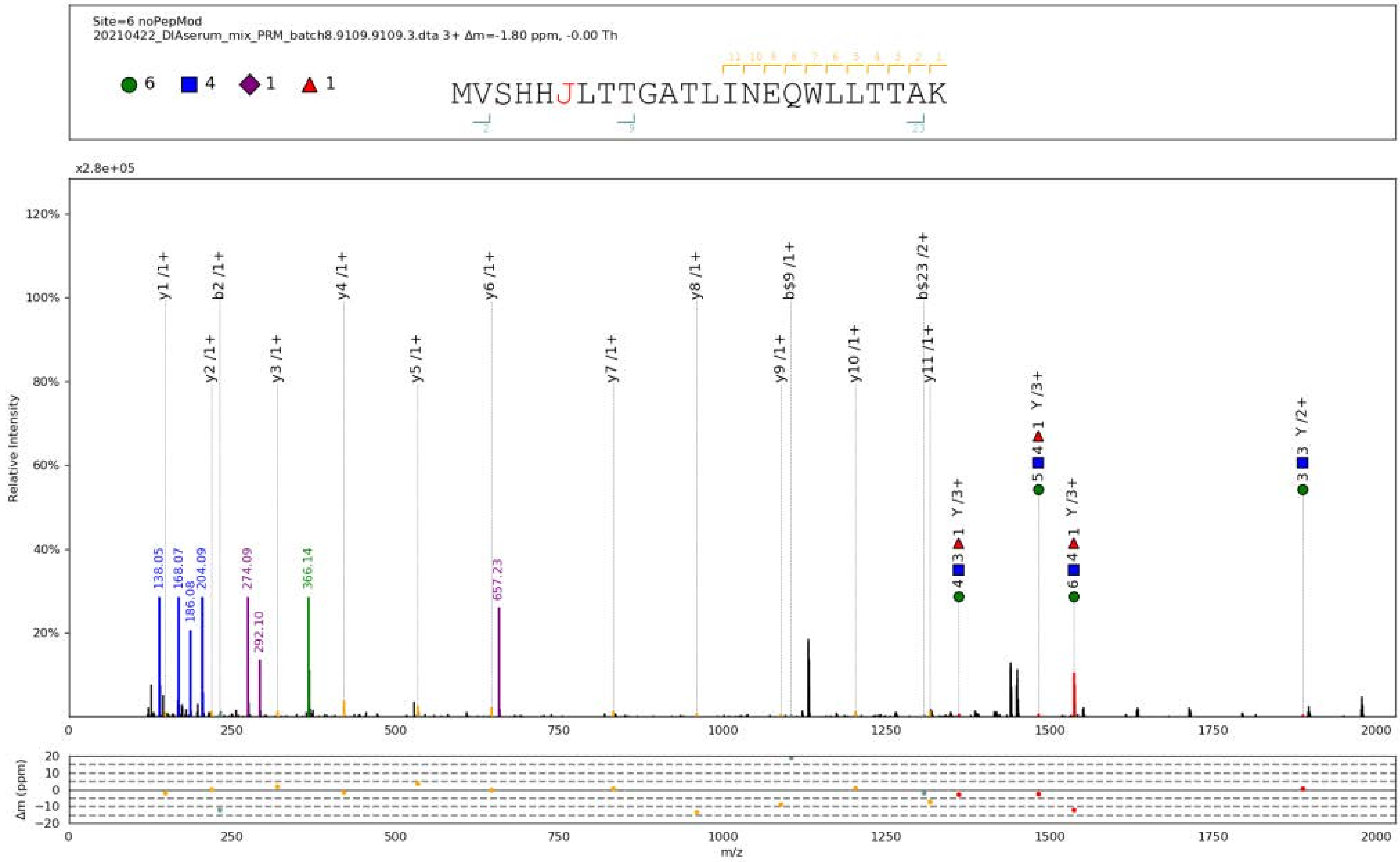

Site=6 noPepMod
20210422_DIAserum_mix_PRM_batch8.9109.9109.3.dta 3+ Δm=-1.80 ppm, -0.00 Th
6 4 1 1
MVSHHJLTTGATLINEQWLLTTAK
x2.8e+05
Relative Intensity
y1 /1+
b2 /1+
y2 /1+
y3 /1+
y4 /1+
y5 /1+
y6 /1+
y7 /1+
y8 /1+
y9 /1+
b$9 /1+
y10 /1+
y11 /1+
b$23 /2+
Y /3+
Y /2+
138.05
168.07
186.08
204.09
274.09
292.10
366.14
657.23
Δm (ppm)
m/z

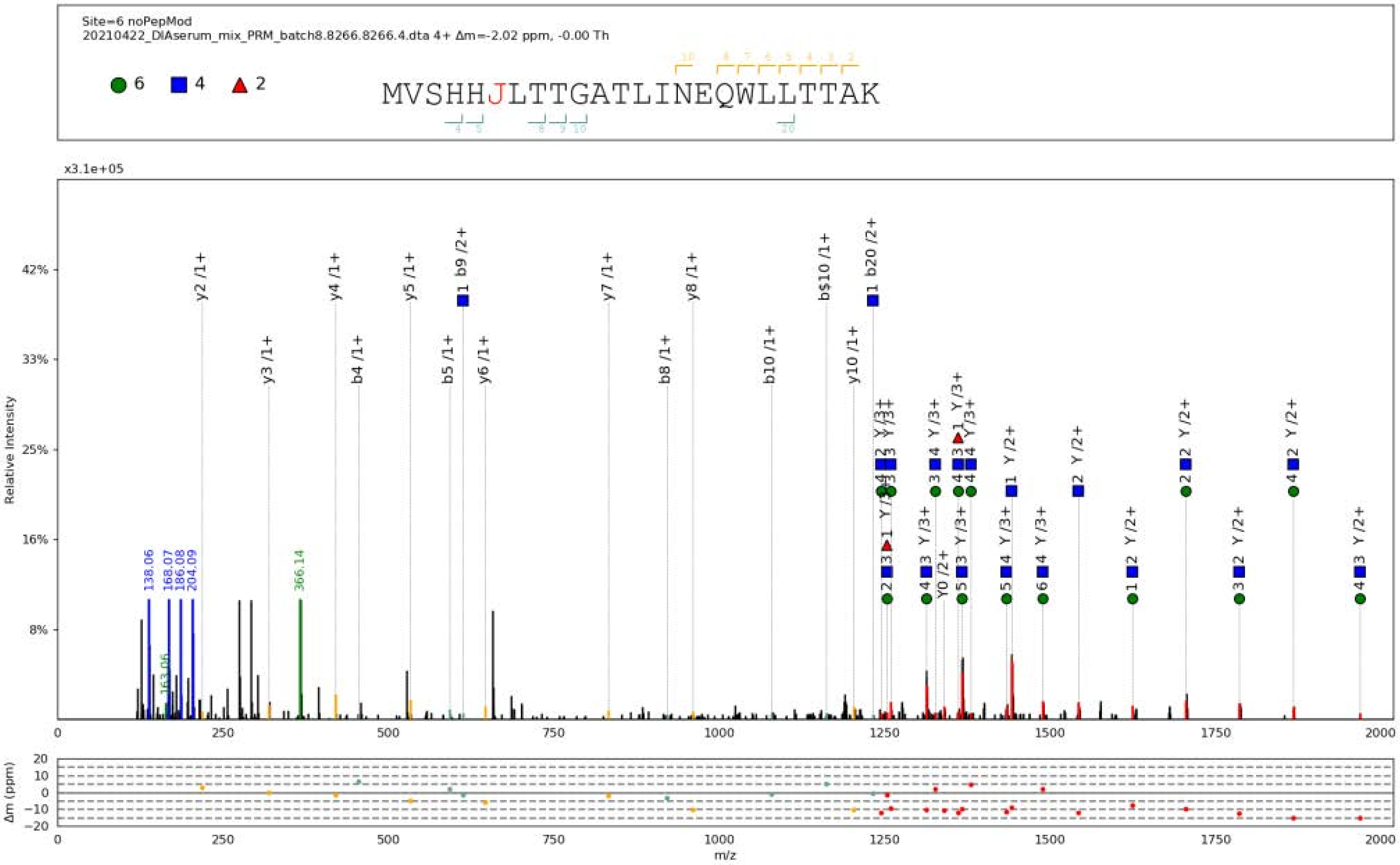

Site=6 noPepMod
20210422_DIAserum_mix_PRM_batch8.8266.8266.4.dta 4+ Δm=-2.02 ppm, -0.00 Th
6
4
2
MVSHHJLTTGATLINEQWLLTTAK
x3.1e+05
Relative Intensity
y2 /1+
y3 /1+
y4 /1+
b4 /1+
y5 /1+
b5 /1+
b9 /2+
y6 /1+
y7 /1+
b8 /1+
y8 /1+
b10 /1+
b$10 /1+
y10 /1+
b20 /2+
Y0 /2+
138.06
168.07
186.08
204.09
163.06
366.14
m/z
Δm (ppm)

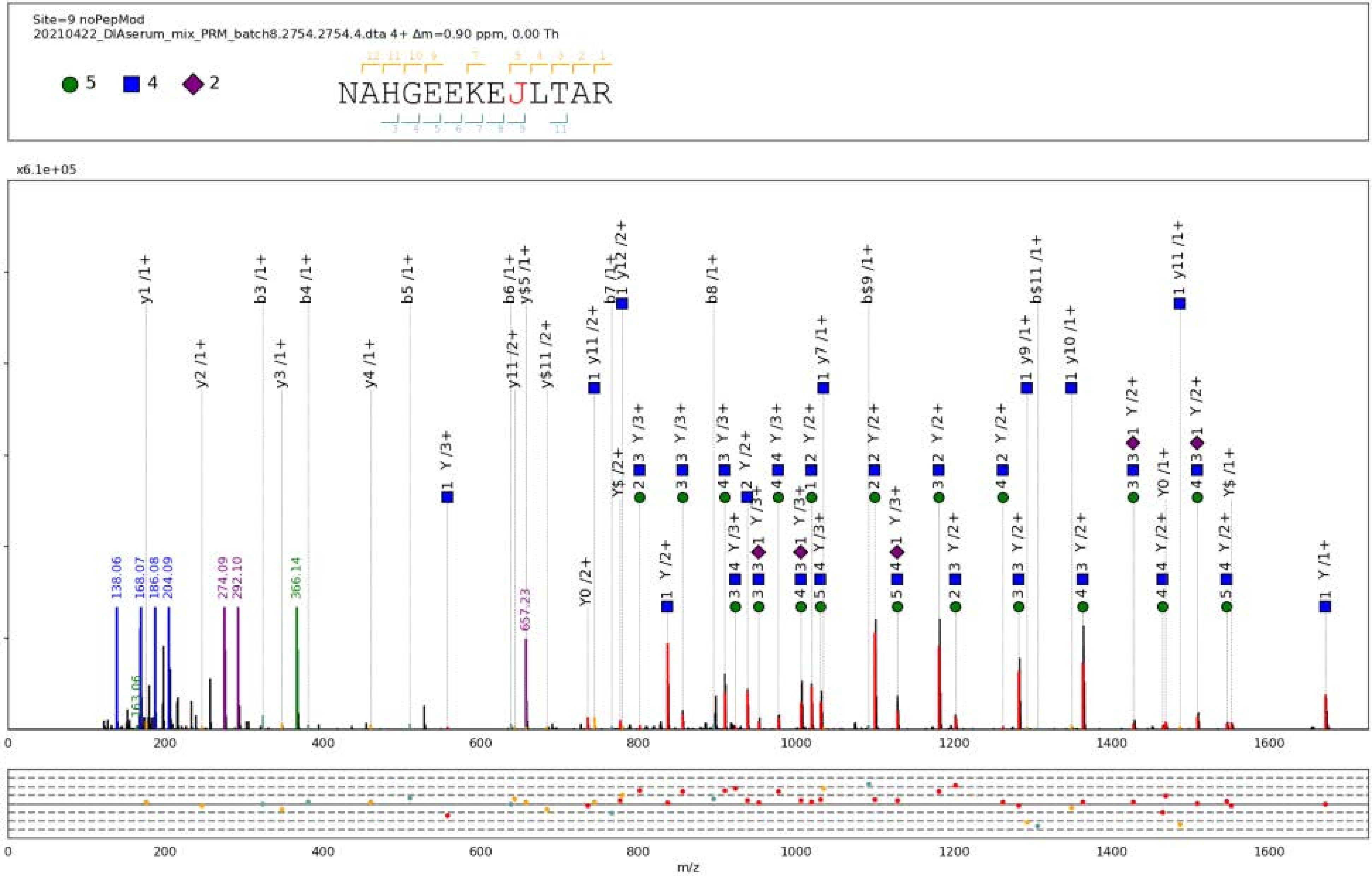
Site=9 noPepMod
20210422_DIAserum_mix_PRM_batch8.2754.2754.4.dta 4+ Δm=0.90 ppm, 0.00 Th
5
4
2
NAHGEEKEJLTAR
x6.1e+05
Relative Intensity
73%
58%
44%
29%
14%
y1 /1+
y2 /1+
b3 /1+
y3 /1+
b4 /1+
y4 /1+
b5 /1+
b6 /1+
y$5 /1+
y11 /2+
y$11 /2+
y11 /2+
b7 /1+
y12 /2+
b8 /1+
y7 /1+
b$9 /1+
b$11 /1+
y9 /1+
y10 /1+
y11 /1+
Y0 /2+
Y$ /2+
Y0 /1+
Y$ /1+
138.06
168.07
186.08
204.09
163.06
274.09
292.10
366.14
657.23
m/z
Δm (ppm)

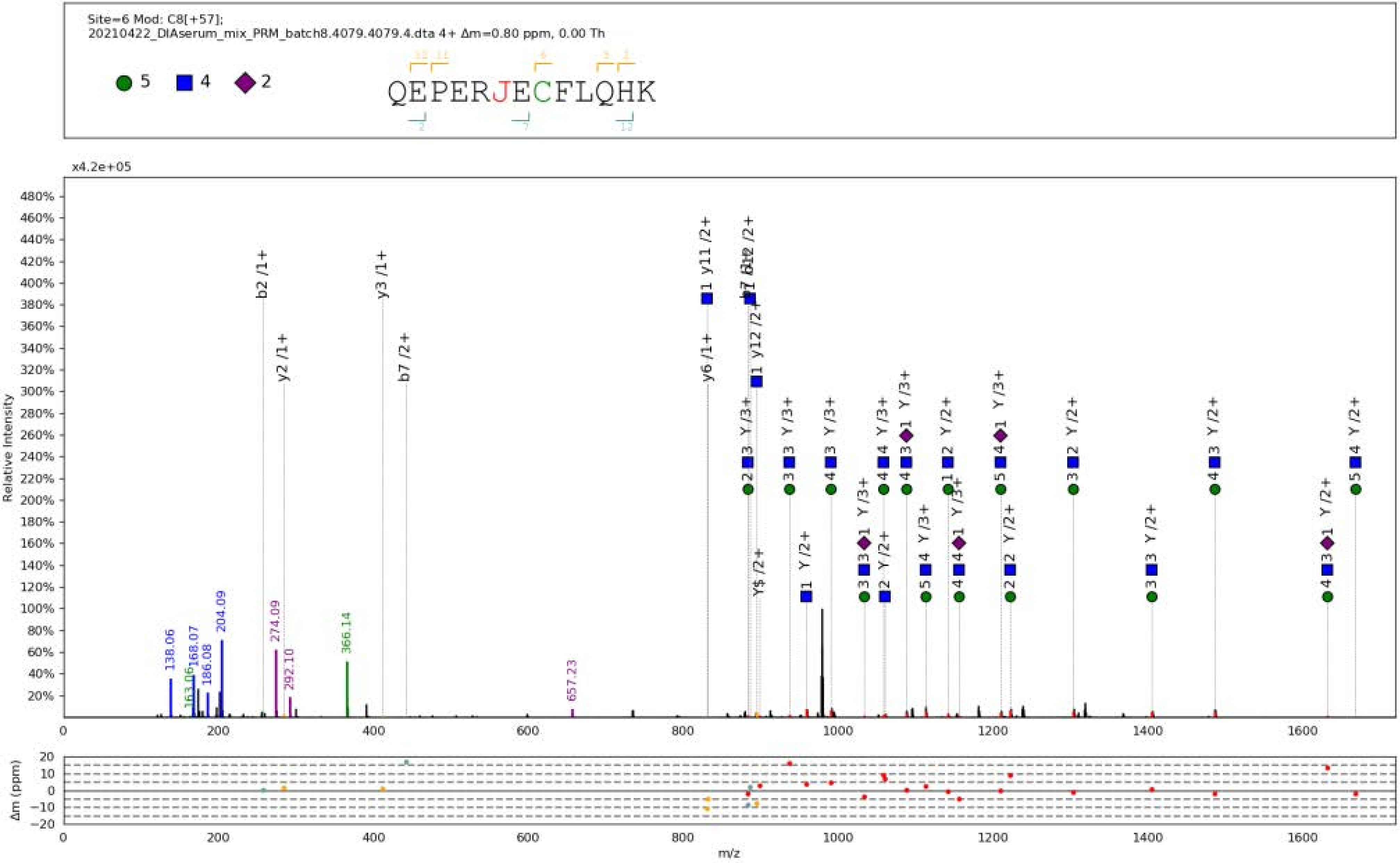

Site=6 Mod: C8[+57];
20210422_DIAserum_mix_PRM_batch8.4079.4079.4.dta 4+ Δm=0.80 ppm, 0.00 Th
5
4
2
QEPERJECFLQHK
x4.2e+05
Relative Intensity
b2 /1+
y2 /1+
y3 /1+
b7 /2+
y6 /1+
y11 /2+
y12 /2+
138.06
163.06
168.07
186.08
204.09
274.09
292.10
366.14
657.23
Δm (ppm)
m/z

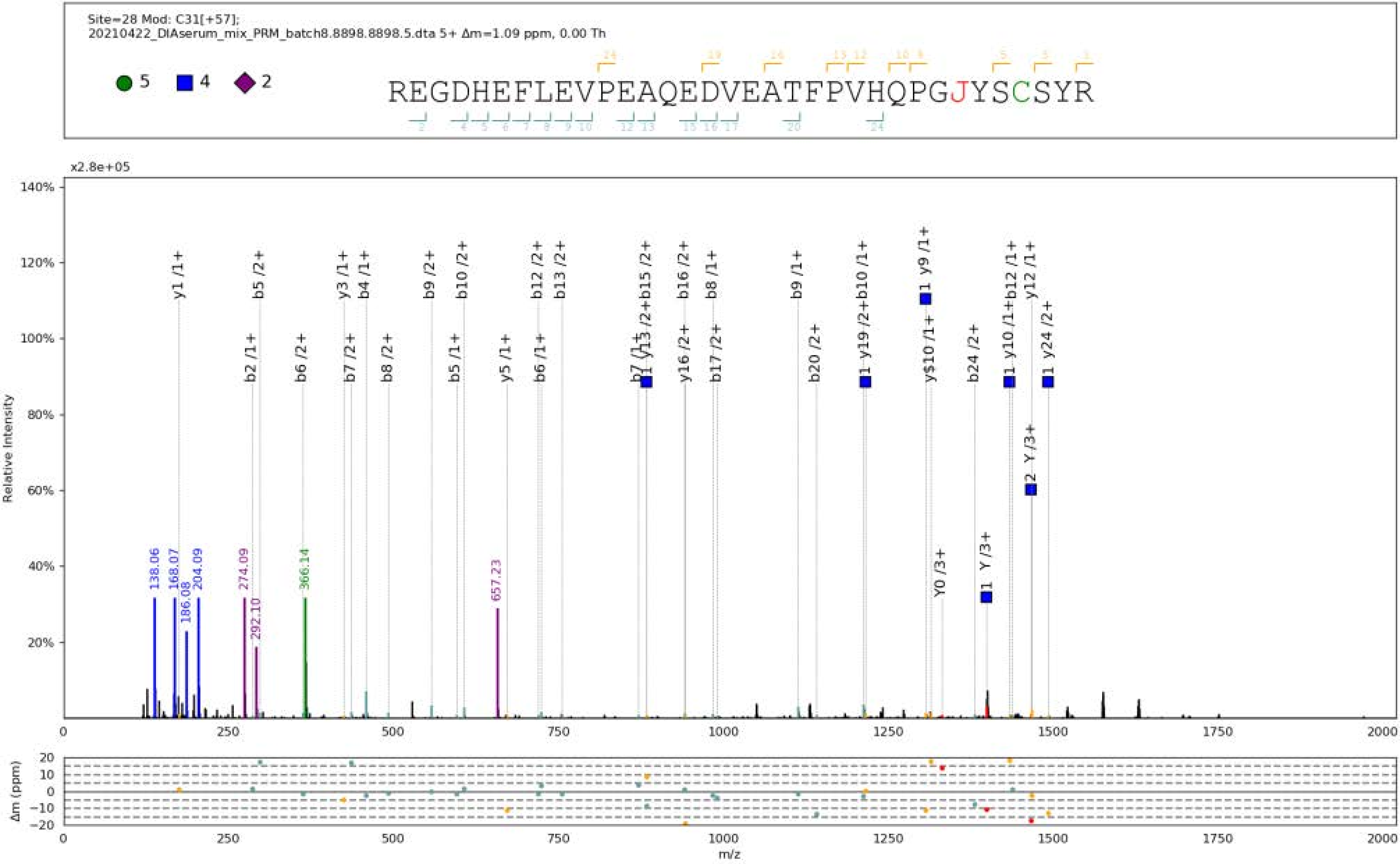

Site=28 Mod: C31[+57];
20210422_DIAserum_mix_PRM_batch8.8898.8898.5.dta 5+ Δm=1.09 ppm, 0.00 Th
5
4
2
REGDHEFLEVPEAQEDVEATFPVHQPGJYSCSYR
x2.8e+05
Relative Intensity
y1 /1+
b5 /2+
y3 /1+
b4 /1+
b9 /2+
b10 /2+
b12 /2+
b13 /2+
b16 /2+
b8 /1+
b9 /1+
y9 /1+
y12 /1+
b2 /1+
b6 /2+
b7 /2+
b8 /2+
b5 /1+
y5 /1+
b6 /1+
y16 /2+
b17 /2+
b20 /2+
y19 /2+b10 /1+
y$10 /1+
b24 /2+
y10 /1+b12 /1+
y24 /2+
Y /3+
Y0 /3+
138.06
168.07
186.08
204.09
274.09
292.10
366.14
657.23
Δm (ppm)
m/z

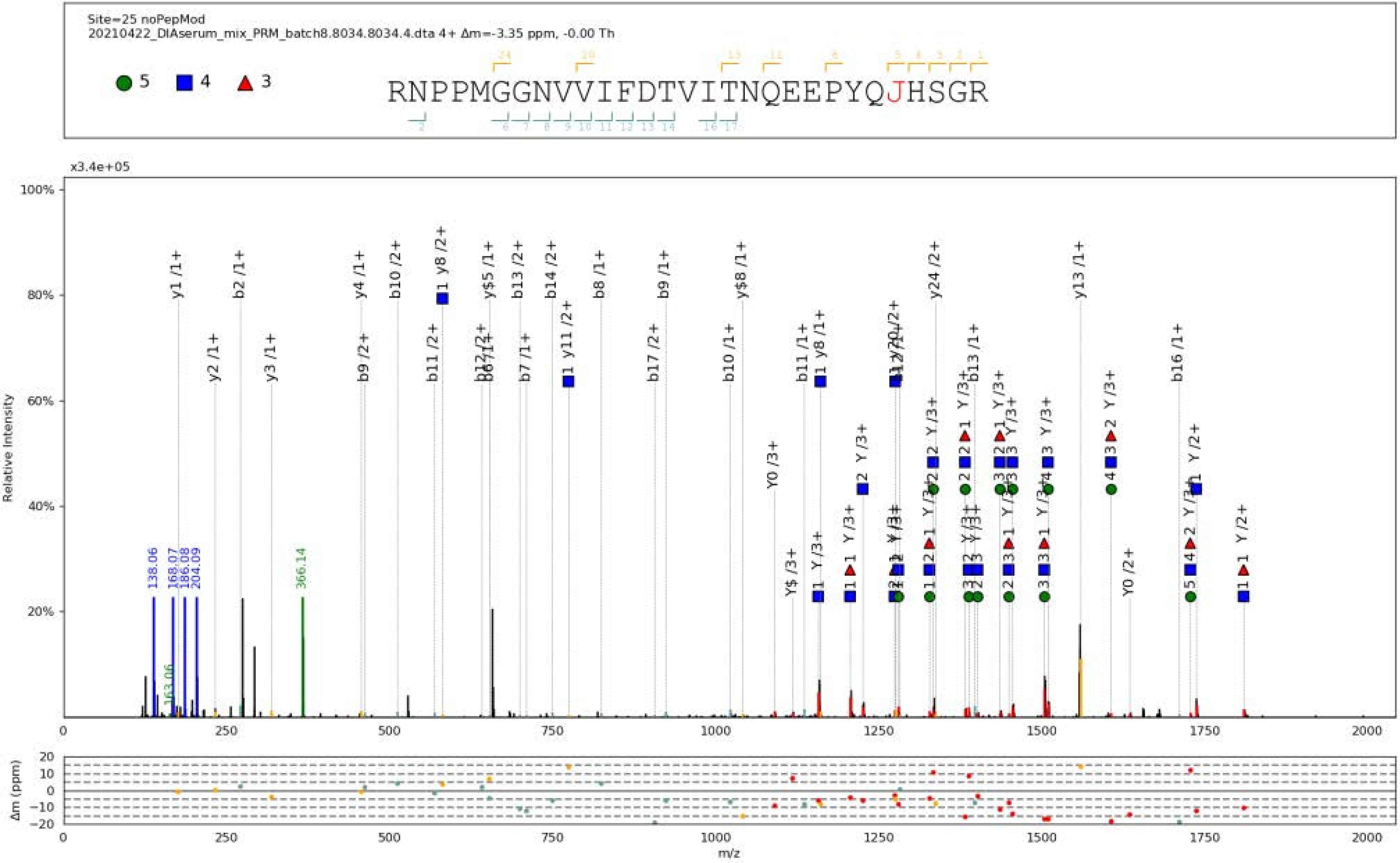
Site=25 noPepMod
20210422_DIAserum_mix_PRM_batch8.8034.8034.4.dta 4+ Δm=-3.35 ppm, -0.00 Th
5
4
3
RNPPMGGNVVIFDTVITNQEEPYQJHSGR
x3.4e+05
Relative Intensity
y1 /1+
y2 /1+
b2 /1+
y3 /1+
y4 /1+
b9 /2+
b10 /2+
b11 /2+
b13 /2+
b14 /2+
b7 /1+
b8 /1+
b17 /2+
b9 /1+
y$8 /1+
b10 /1+
b11 /1+
y24 /2+
b13 /1+
y13 /1+
b16 /1+
Y0 /3+
Y0 /2+
138.06
168.07
186.08
204.09
366.14
163.06
m/z
Δm (ppm)

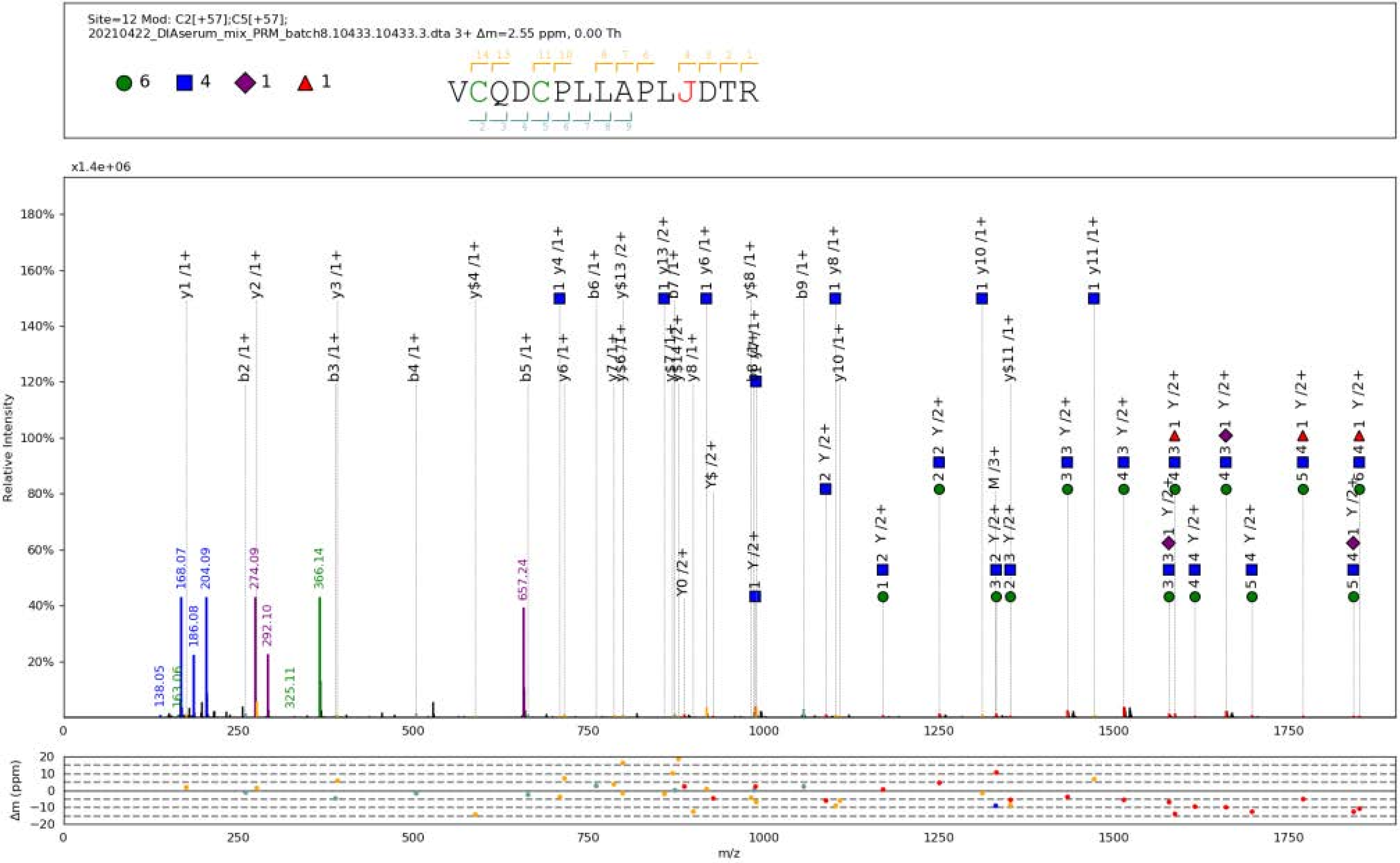

Site=12 Mod: C2[+57];C5[+57];
20210422_DIAserum_mix_PRM_batch8.10433.10433.3.dta 3+ Δm=2.55 ppm, 0.00 Th
6
4
1
1
VCQDCPLLAPLJDTR
x1.4e+06
Relative Intensity
y1 /1+
y2 /1+
y3 /1+
y$4 /1+
b2 /1+
b3 /1+
b4 /1+
b5 /1+
y6 /1+
b6 /1+
y$13 /2+
b7 /1+
y8 /1+
b9 /1+
y10 /1+
y$11 /1+
Y0 /2+
Y$ /2+
M /3+
168.07
186.08
204.09
138.05
163.06
274.09
292.10
325.11
366.14
657.24
m/z
Δm (ppm)

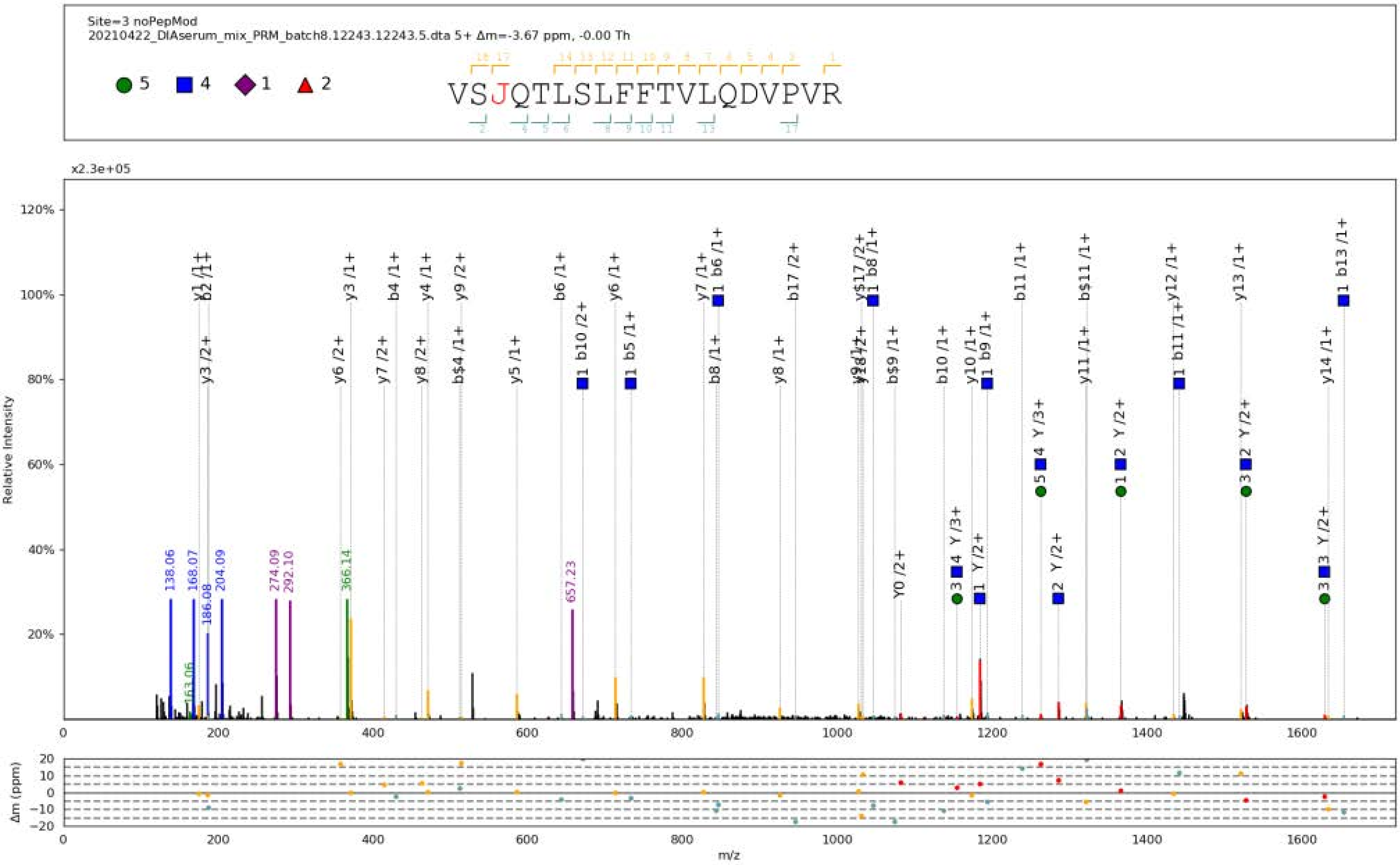
Site=3 noPepMod
20210422_DIAserum_mix_PRM_batch8.12243.12243.5.dta 5+ Δm=-3.67 ppm, -0.00 Th
5
4
1
2
VSJQTLSLFFTVLQDVPVR
x2.3e+05
Relative Intensity
120%
100%
80%
60%
40%
20%
y1 /1+
b2 /1+
y3 /2+
y6 /2+
y3 /1+
y7 /2+
b4 /1+
y8 /2+
y4 /1+
b$4 /1+
y9 /2+
y5 /1+
b6 /1+
b10 /2+
y6 /1+
b5 /1+
y7 /1+
b6 /1+
b8 /1+
y8 /1+
b17 /2+
y$17 /2+
b8 /1+
b$9 /1+
Y0 /2+
b10 /1+
y10 /1+
b9 /1+
Y /3+
Y /2+
b11 /1+
Y /3+
Y /2+
y11 /1+
b$11 /1+
Y /2+
y12 /1+
b11 /1+
y13 /1+
Y /2+
y14 /1+
Y /2+
b13 /1+
138.06
168.07
186.08
204.09
163.06
274.09
292.10
366.14
657.23
0
200
400
600
800
1000
1200
1400
1600
Δm (ppm)
20
10
0
-10
-20
m/z

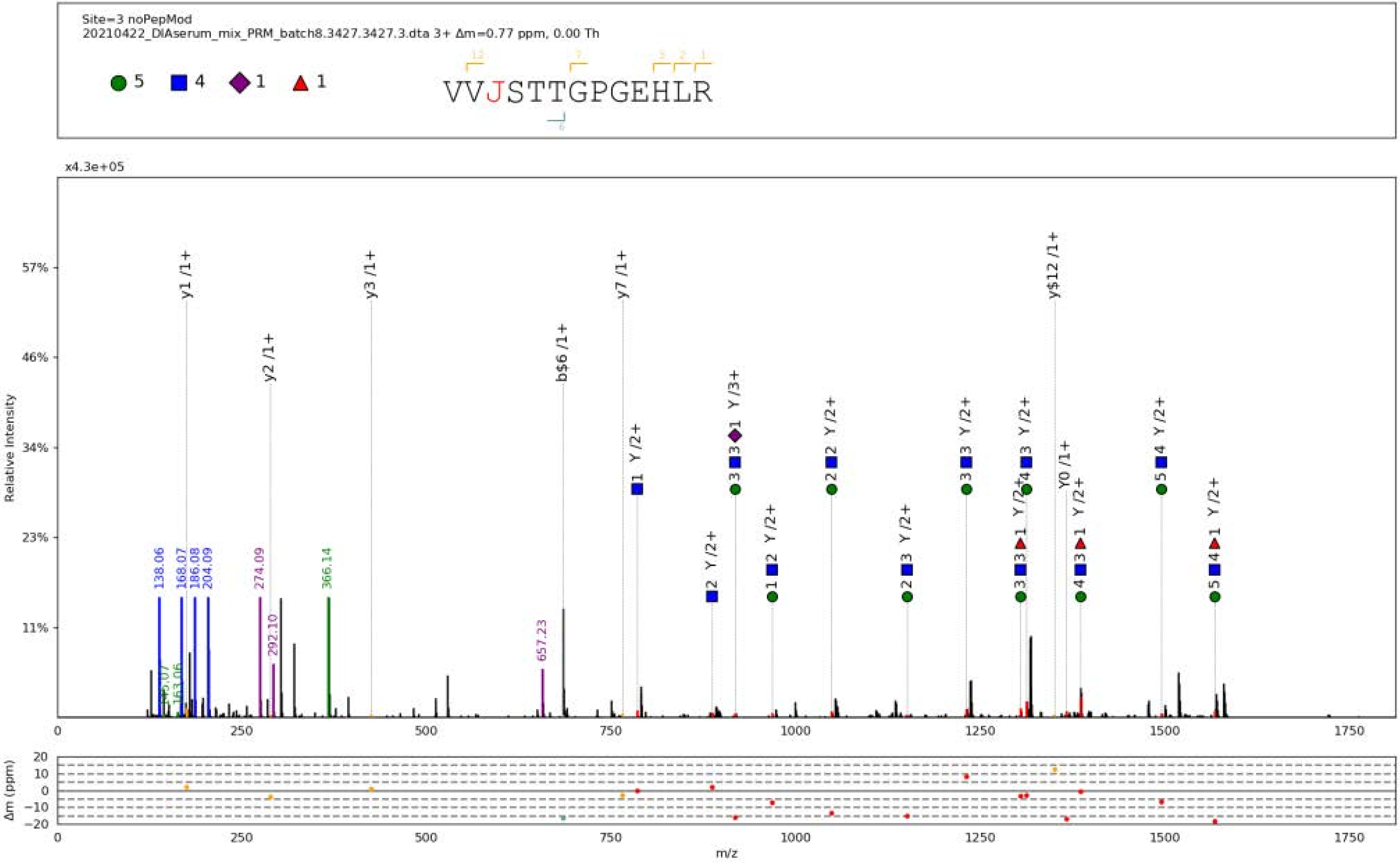

Site=3 noPepMod
20210422_DIAserum_mix_PRM_batch8.3427.3427.3.dta 3+ Δm=0.77 ppm, 0.00 Th
VVJSTTGPGEHLR
x4.3e+05
Relative Intensity
m/z
Δm (ppm)

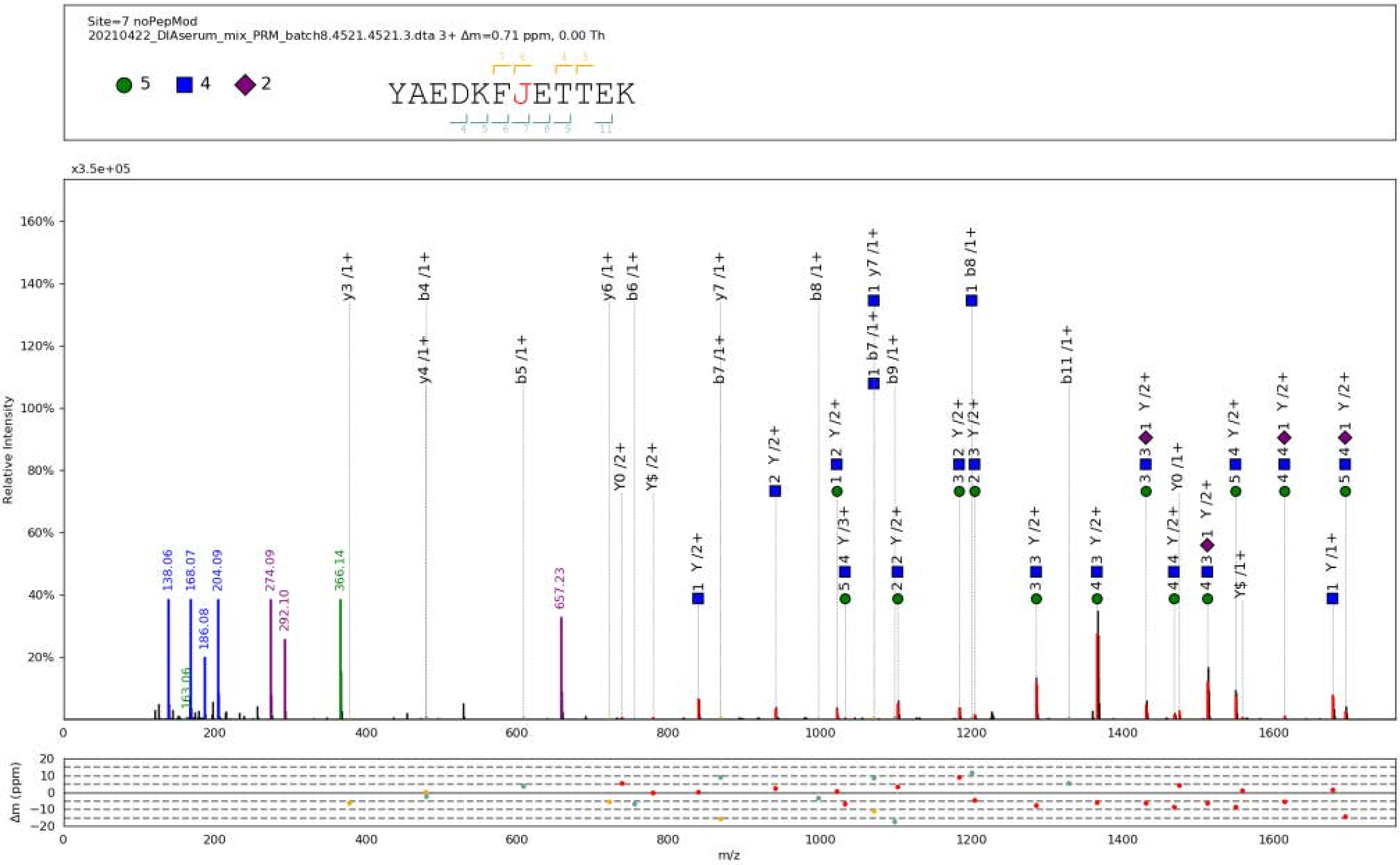

Site=7 noPepMod
20210422_DIAserum_mix_PRM_batch8.4521.4521.3.dta 3+ Δm=0.71 ppm, 0.00 Th
5
4
2
YAEDKFJETTEK
x3.5e+05
Relative Intensity
160%
140%
120%
100%
80%
60%
40%
20%
y3 /1+
b4 /1+
y4 /1+
b5 /1+
y6 /1+
b6 /1+
Y0 /2+
Y$ /2+
y7 /1+
b7 /1+
b8 /1+
b7 /1+
y7 /1+
b9 /1+
b8 /1+
b11 /1+
Y0 /1+
Y$ /1+
Y /2+
Y /3+
Y /1+
138.06
168.07
186.08
204.09
163.06
274.09
292.10
366.14
657.23
m/z
Δm (ppm)

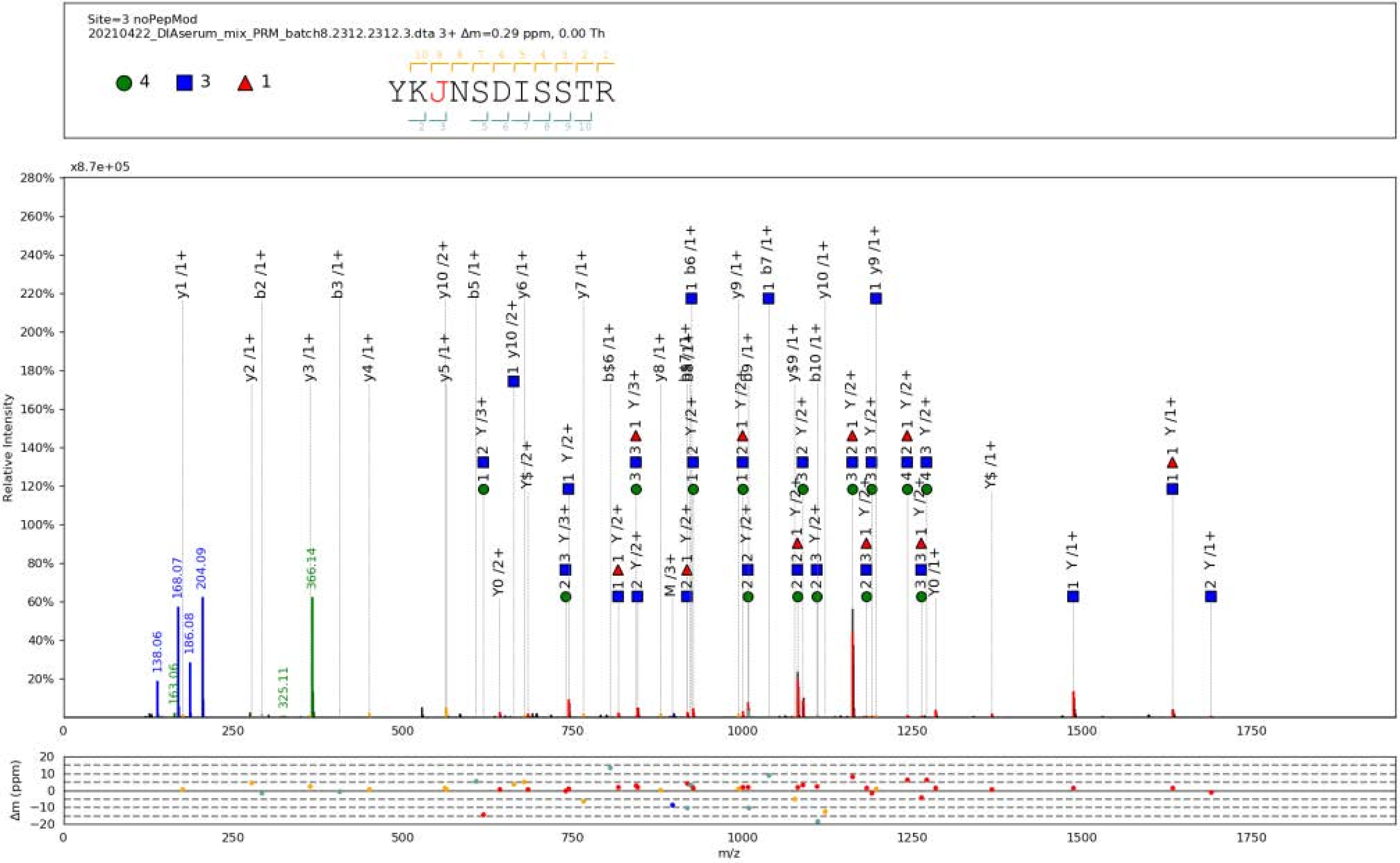

Site=3 noPepMod
20210422_DIAserum_mix_PRM_batch8.2312.2312.3.dta 3+ Δm=0.29 ppm, 0.00 Th
4 3 1
YKJNSDISSTR
x8.7e+05
Relative Intensity
Δm (ppm)
m/z

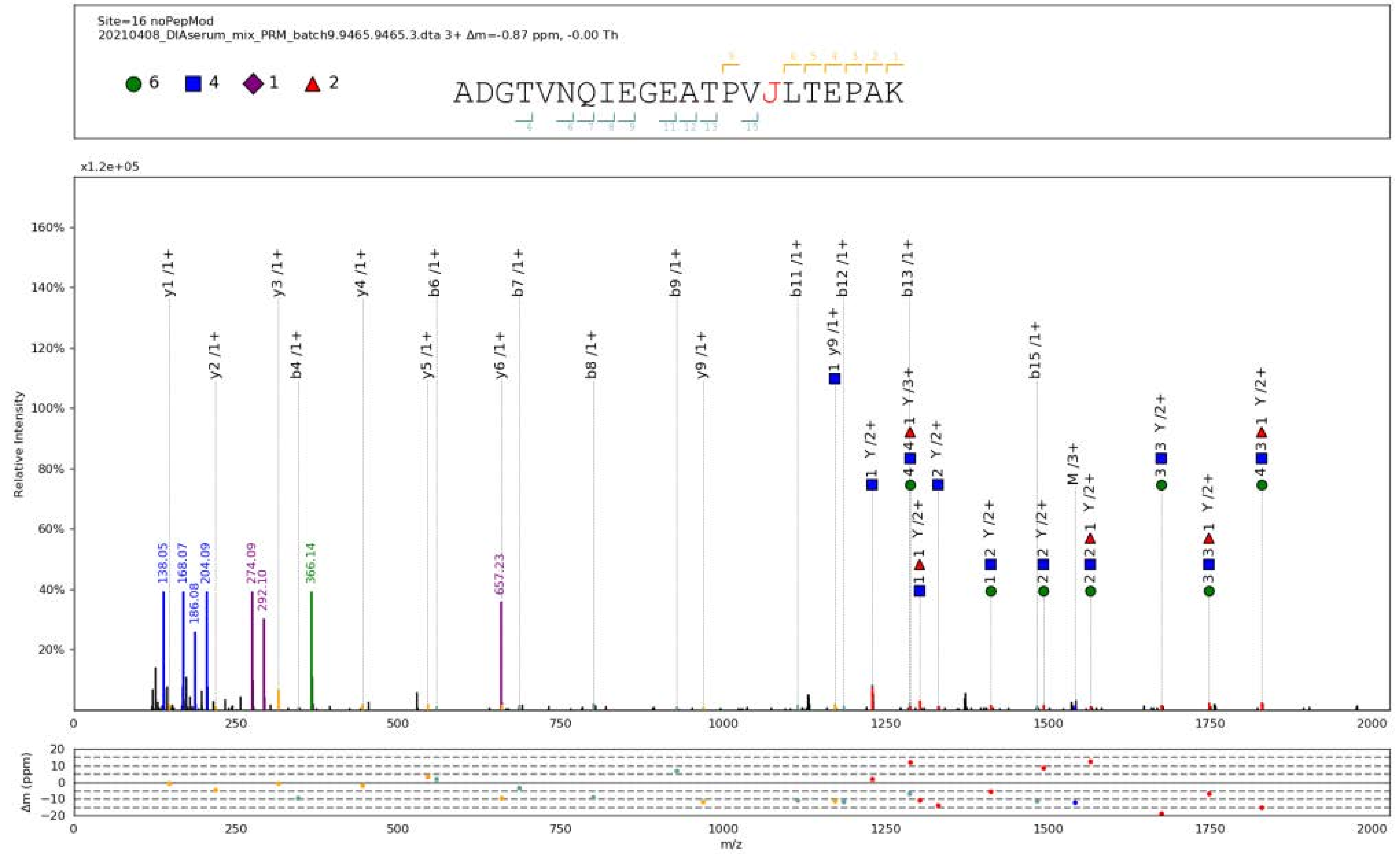

Site=16 noPepMod
20210408_DIAserum_mix_PRM_batch9.9465.9465.3.dta 3+ Δm=-0.87 ppm, -0.00 Th
ADGTVNQIEGEATPVJLTEPAK
6
4
1
2
x1.2e+05
Relative Intensity
m/z
Δm (ppm)

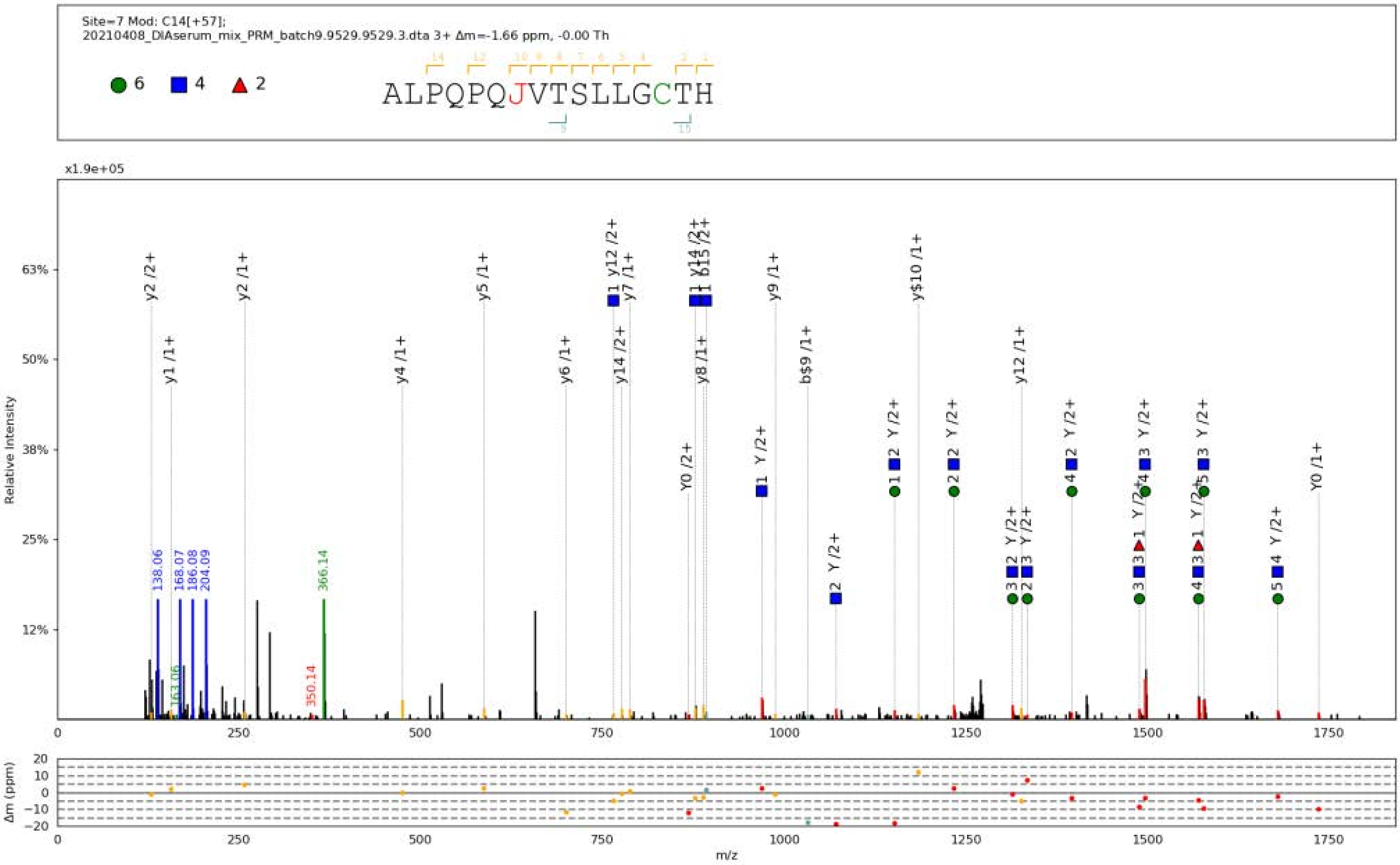

Site=7 Mod: C14[+57];
20210408_DIAserum_mix_PRM_batch9.9529.9529.3.dta 3+ Δm=-1.66 ppm, -0.00 Th
ALPQPQJVTSLLGCTH
x1.9e+05
Relative Intensity
m/z
Δm (ppm)

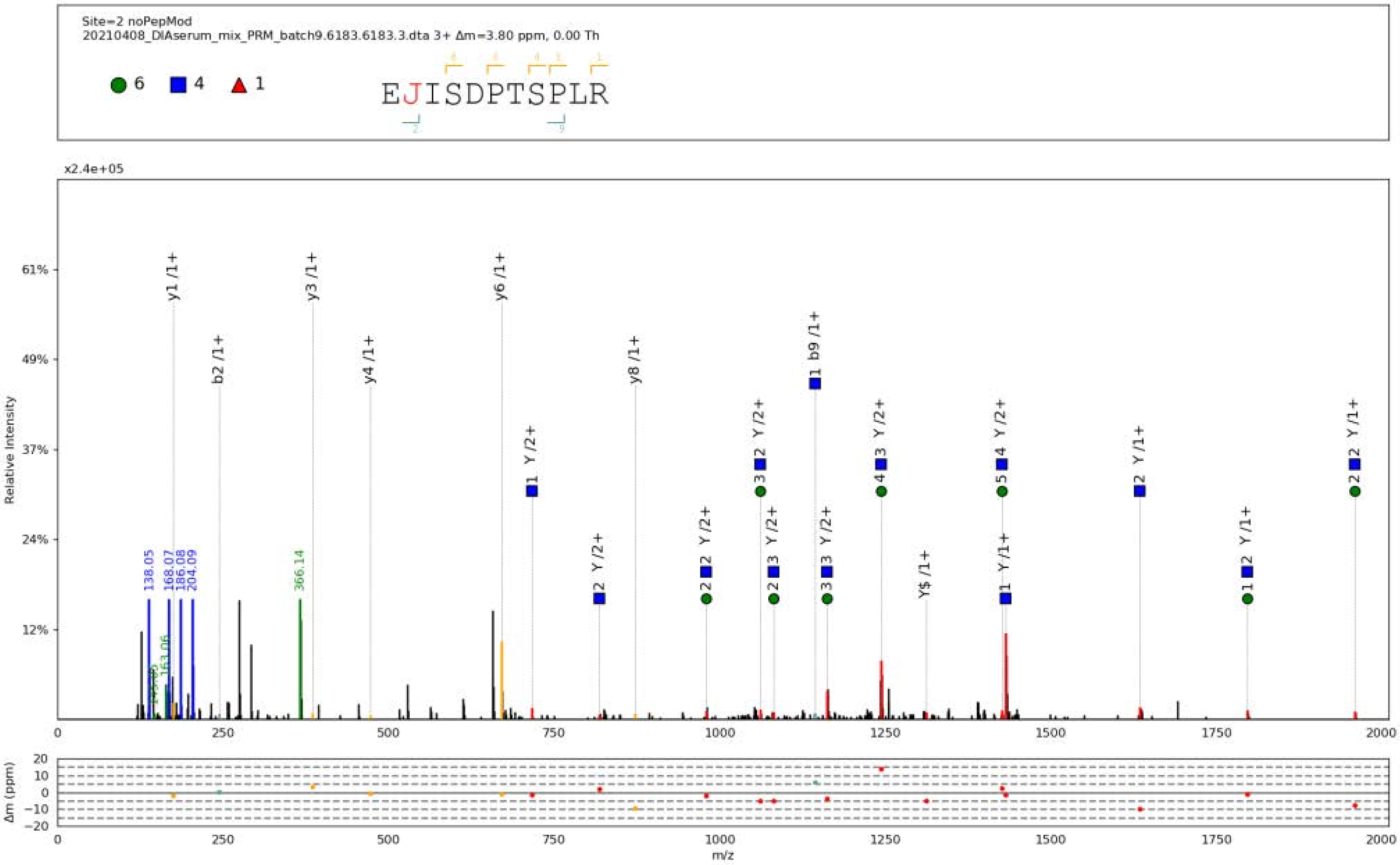

Site=2 noPepMod
20210408_DIAserum_mix_PRM_batch9.6183.6183.3.dta 3+ Δm=3.80 ppm, 0.00 Th
6 4 1
EJISDPTSPLR
x2.4e+05
Relative Intensity
m/z
Δm (ppm)

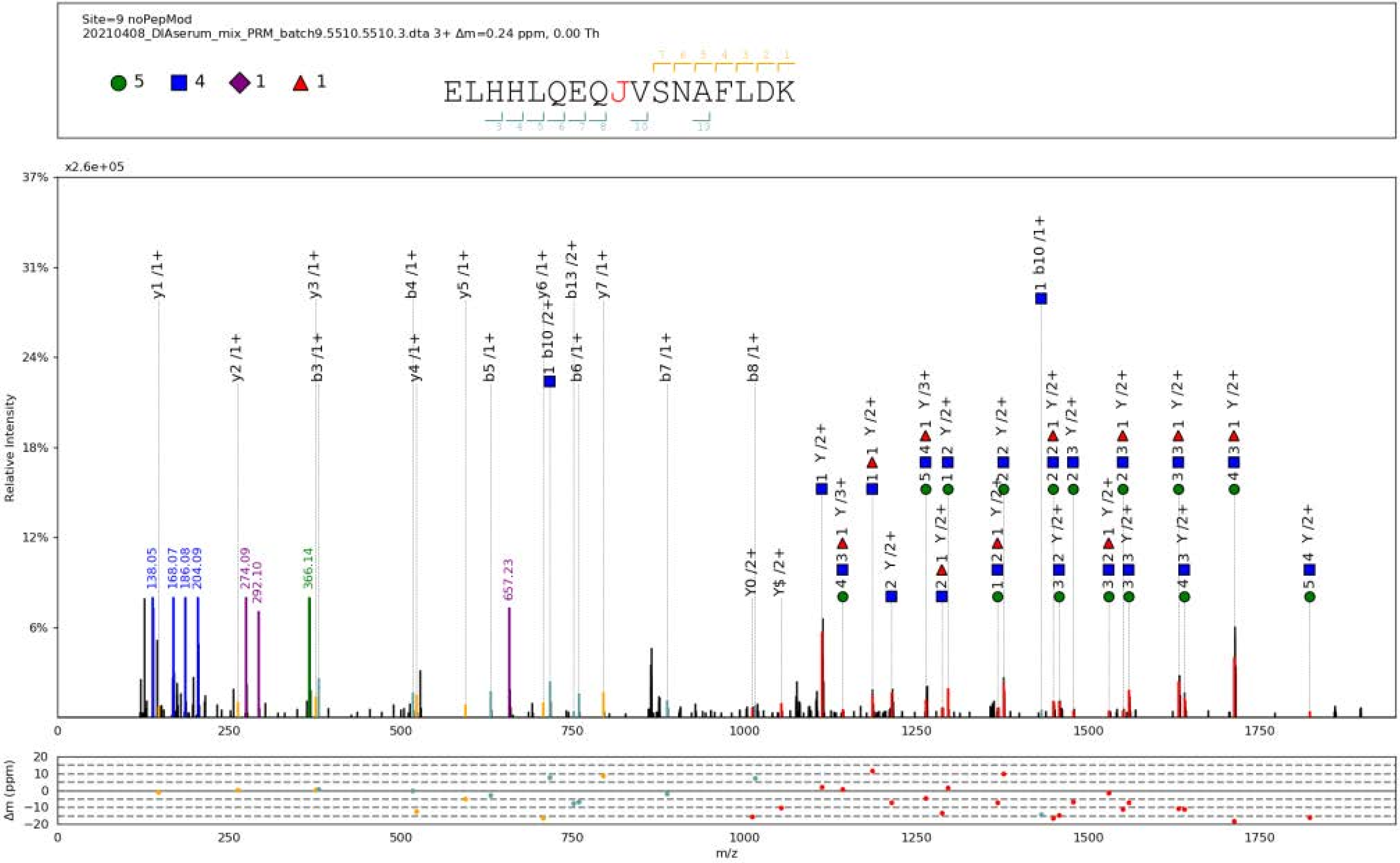
Site=9 noPepMod
20210408_DIAserum_mix_PRM_batch9.5510.5510.3.dta 3+ Δm=0.24 ppm, 0.00 Th
5 4 1 1
ELHHLQEQJVSNAFLDK
x2.6e+05
Relative Intensity
y1 /1+
y2 /1+
y3 /1+
b3 /1+
b4 /1+
y4 /1+
y5 /1+
b5 /1+
b10 /2+
y6 /1+
b13 /2+
b6 /1+
y7 /1+
b7 /1+
b8 /1+
b10 /1+
Y0 /2+
Y$ /2+
Y /2+
Y /3+
138.05
168.07
186.08
204.09
274.09
292.10
366.14
657.23
Δm (ppm)
m/z

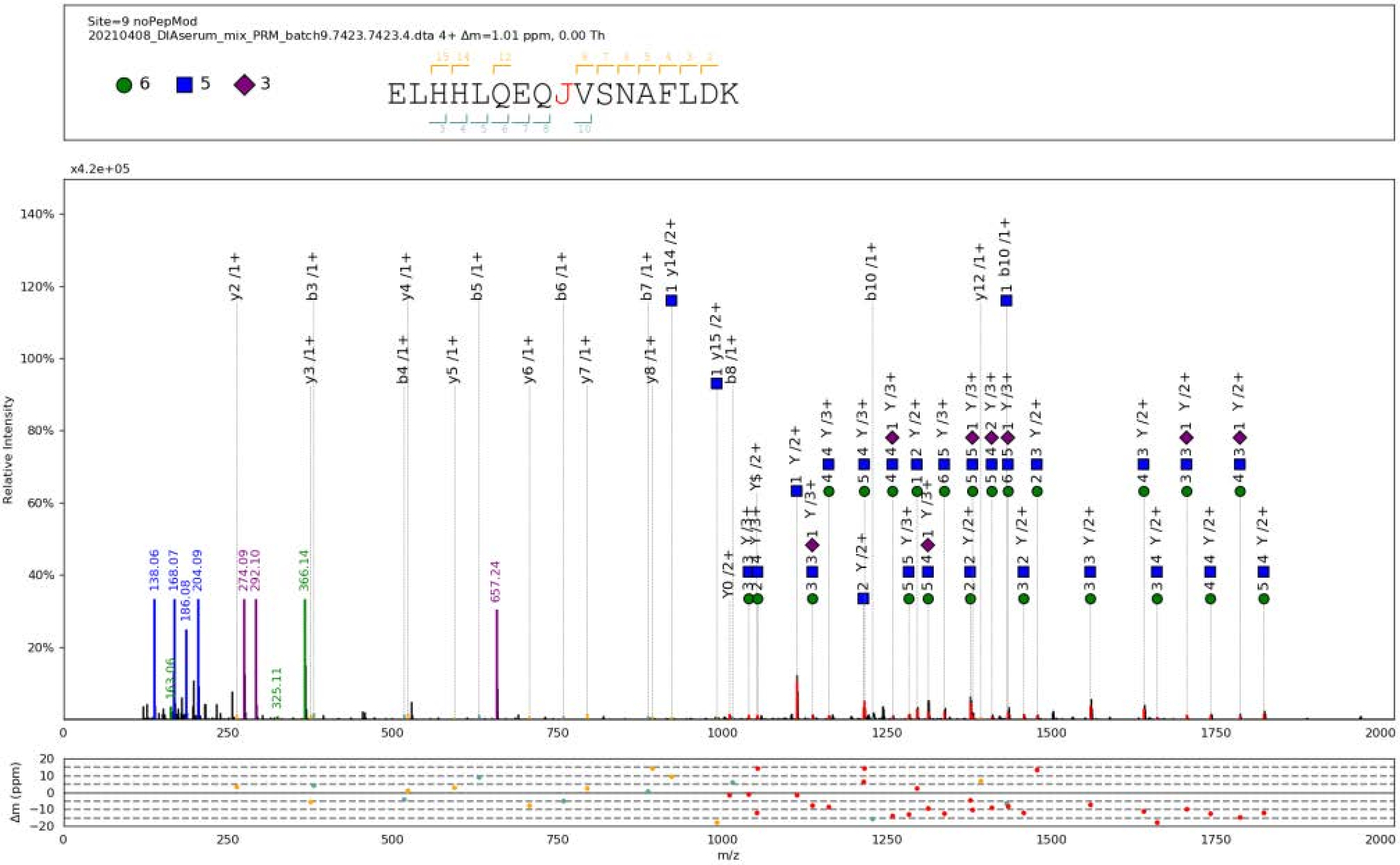
Site=9 noPepMod
20210408_DIAserum_mix_PRM_batch9.7423.7423.4.dta 4+ Δm=1.01 ppm, 0.00 Th
6
5
3
ELHHLQEQJVSNAFLDK
x4.2e+05
Relative Intensity
y2 /1+
b3 /1+
y3 /1+
y4 /1+
b4 /1+
y5 /1+
b5 /1+
y6 /1+
b6 /1+
y7 /1+
b7 /1+
y8 /1+
y14 /2+
y15 /2+
b8 /1+
Y0 /2+
Y$ /2+
b10 /1+
y12 /1+
b10 /1+
138.06
168.07
186.08
204.09
163.06
274.09
292.10
366.14
325.11
657.24
m/z
Δm (ppm)

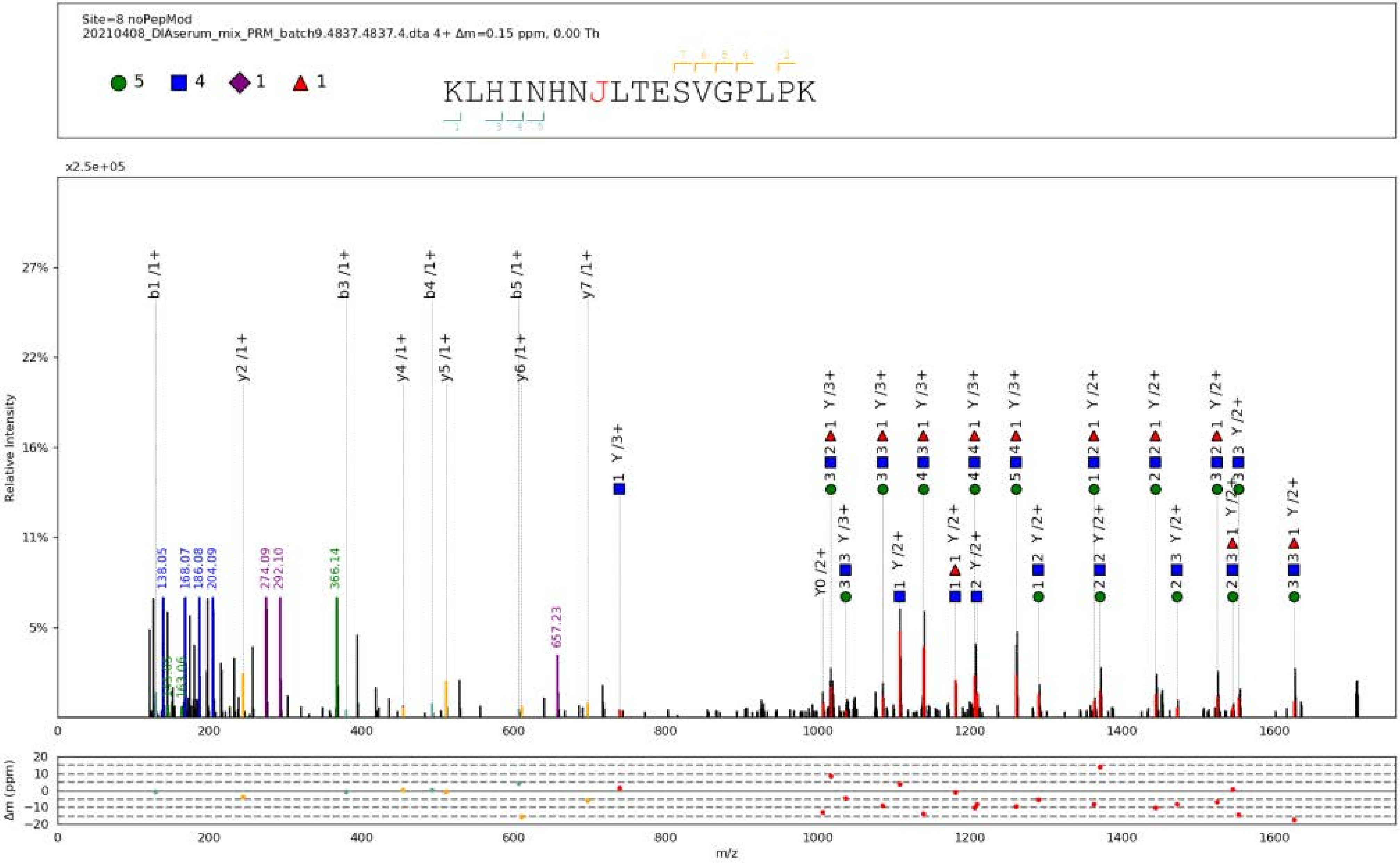

Site=8 noPepMod
20210408_DIAserum_mix_PRM_batch9.4837.4837.4.dta 4+ Δm=0.15 ppm, 0.00 Th
5 4 1 1
KLHINHNJLTESVGPLPK
x2.5e+05
Relative Intensity
b1 /1+
b3 /1+
b4 /1+
b5 /1+
y7 /1+
y2 /1+
y4 /1+
y5 /1+
y6 /1+
Y0 /2+
138.05
168.07
186.08
204.09
274.09
292.10
366.14
657.23
m/z
Δm (ppm)

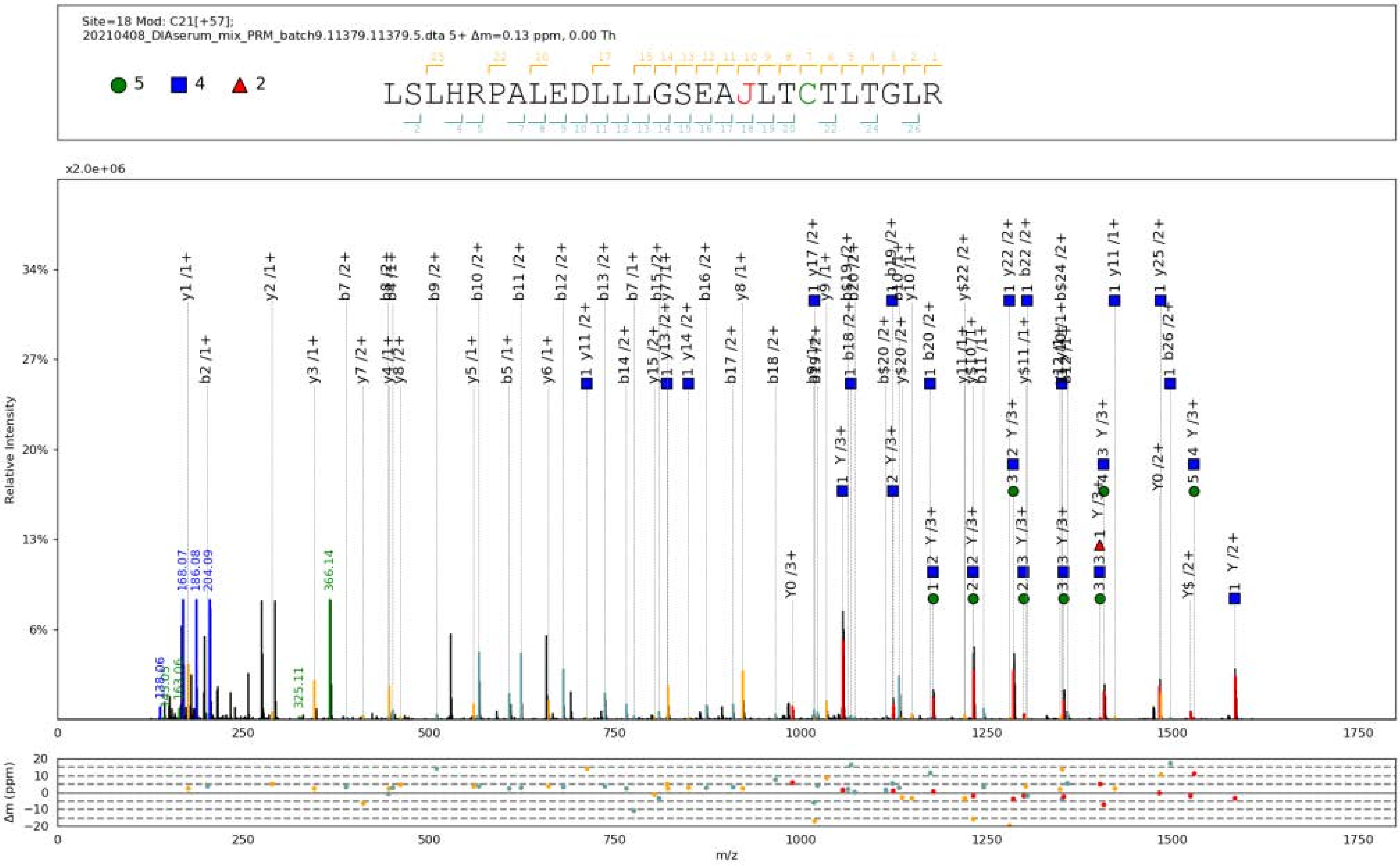
Site=18 Mod: C21[+57];
20210408_DIAserum_mix_PRM_batch9.11379.11379.5.dta 5+ Δm=0.13 ppm, 0.00 Th
5
4
2
LSLHRPALEDLLLGSEAJLTCTLTGLR
x2.0e+06
Relative Intensity
34%
27%
20%
13%
6%
0
250
500
750
1000
1250
1500
1750
Δm (ppm)
m/z
168.07
186.08
204.09
366.14
325.11
y1 /1+
b2 /1+
y2 /1+
y3 /1+
b7 /2+
y7 /2+
b9 /2+
b10 /2+
y5 /1+
b11 /2+
b5 /1+
b12 /2+
y6 /1+
1 y11 /2+
b13 /2+
b14 /2+
b7 /1+
b16 /2+
1 y14 /2+
b17 /2+
y8 /1+
b18 /2+
Y0 /3+
1 y17 /2+
y9 /1+
1 b18 /2+
1 b20 /2+
y10 /1+
y$22 /2+
1 y22 /2+
1 b22 /2+
y$11 /1+
1 y11 /1+
1 y25 /2+
1 b26 /2+
Y0 /2+
Y$ /2+
1 Y /2+

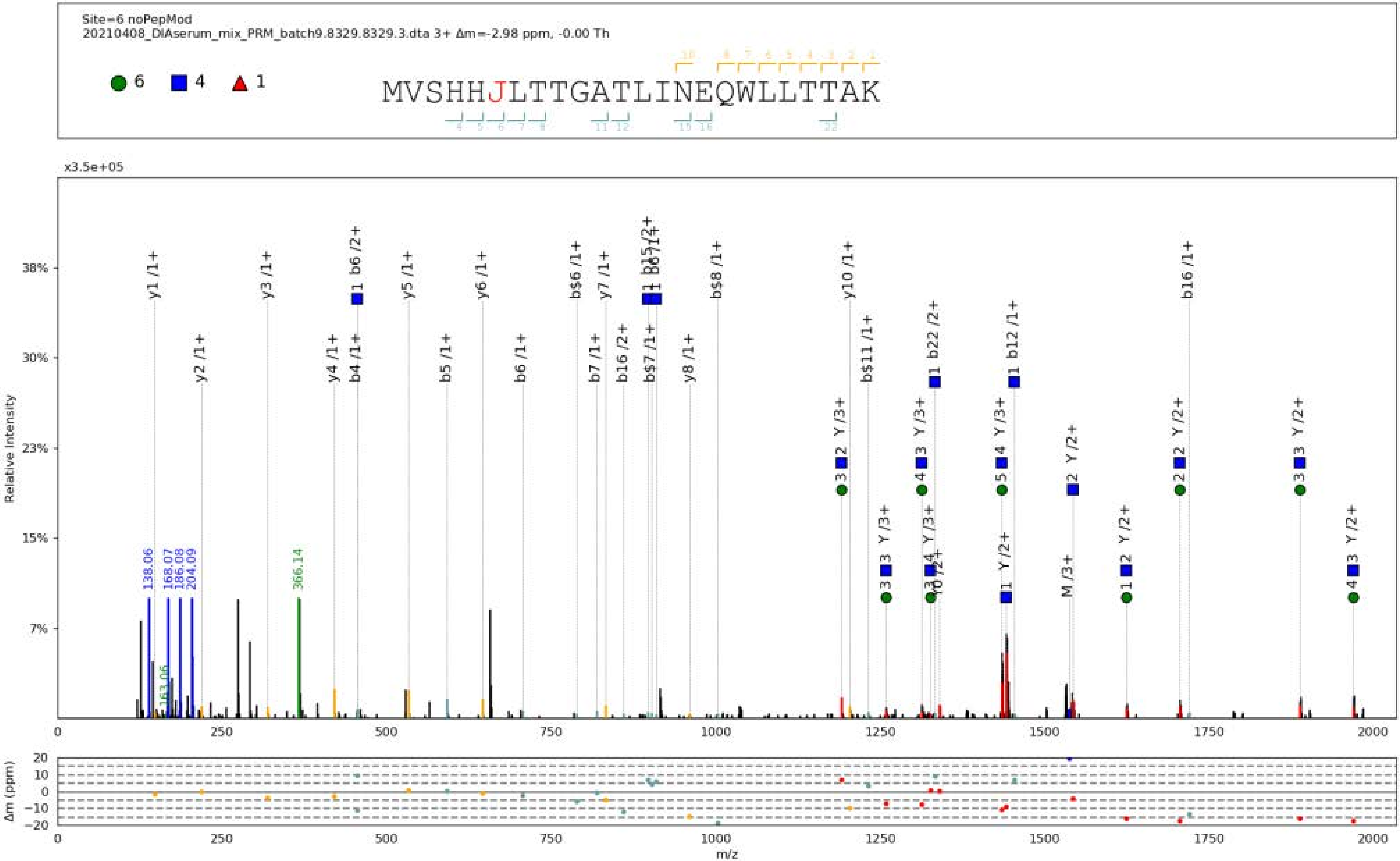

Site=6 noPepMod
20210408_DIAserum_mix_PRM_batch9.8329.8329.3.dta 3+ Δm=-2.98 ppm, -0.00 Th
6
4
1
MVSHHJLTTGATLINEQWLLTTAK
x3.5e+05
Relative Intensity
38%
30%
23%
15%
7%
y1 /1+
y2 /1+
y3 /1+
y4 /1+
b4 /1+
b6 /2+
y5 /1+
b5 /1+
y6 /1+
b6 /1+
b$6 /1+
b7 /1+
y7 /1+
b16 /2+
b$7 /1+
y8 /1+
b$8 /1+
y10 /1+
b$11 /1+
b22 /2+
b12 /1+
b16 /1+
138.06
168.07
186.08
204.09
163.06
366.14
Δm (ppm)
m/z

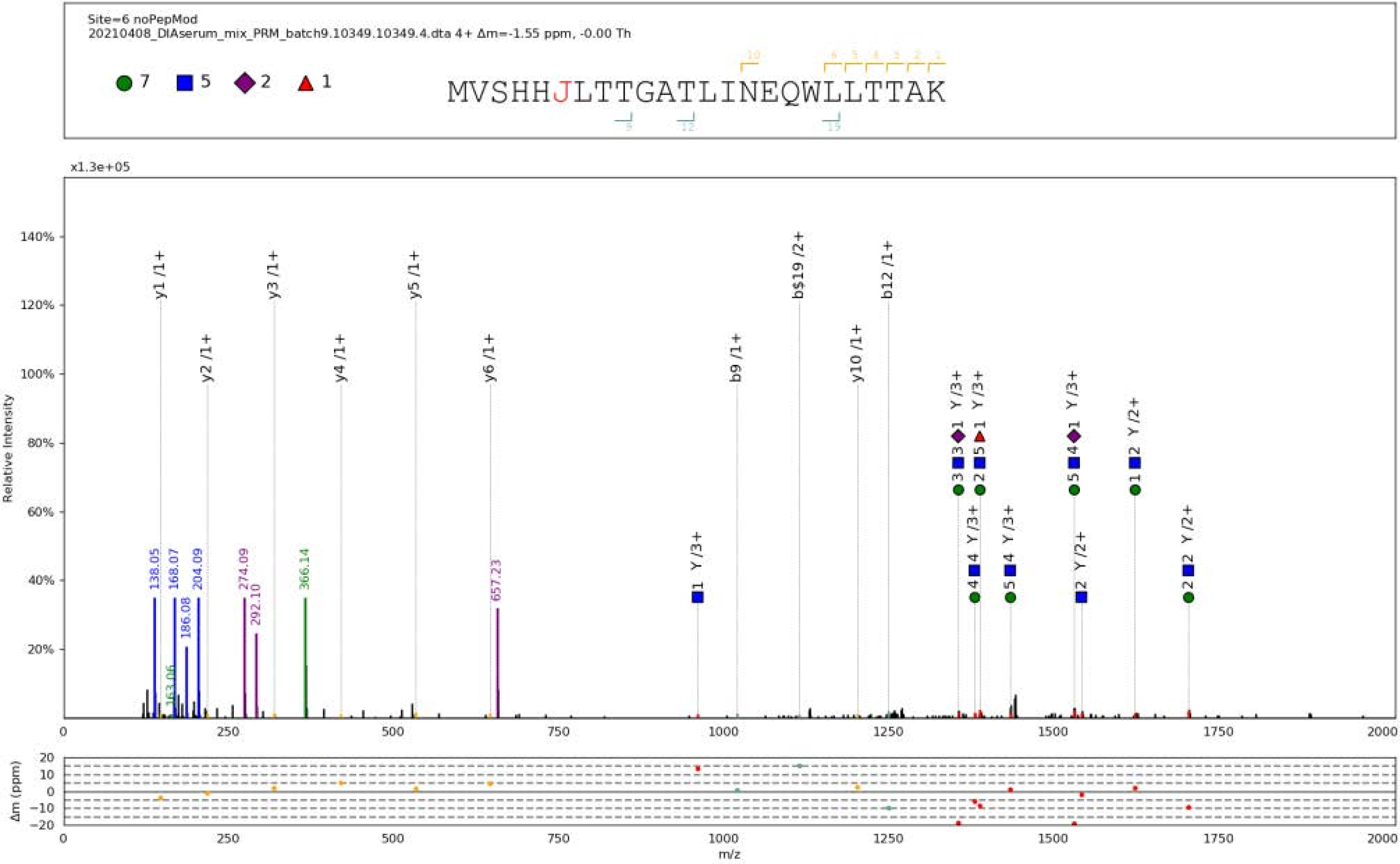

Site=6 noPepMod
20210408_DIAserum_mix_PRM_batch9.10349.10349.4.dta 4+ Δm=-1.55 ppm, -0.00 Th
7 5 2 1
MVSHHJLTTGATLINEQWLLTTAK
x1.3e+05
Relative Intensity
y1 /1+
y2 /1+
y3 /1+
y4 /1+
y5 /1+
y6 /1+
b9 /1+
b$19 /2+
y10 /1+
b12 /1+
138.05
168.07
186.08
204.09
163.06
274.09
292.10
366.14
657.23
m/z
Δm (ppm)

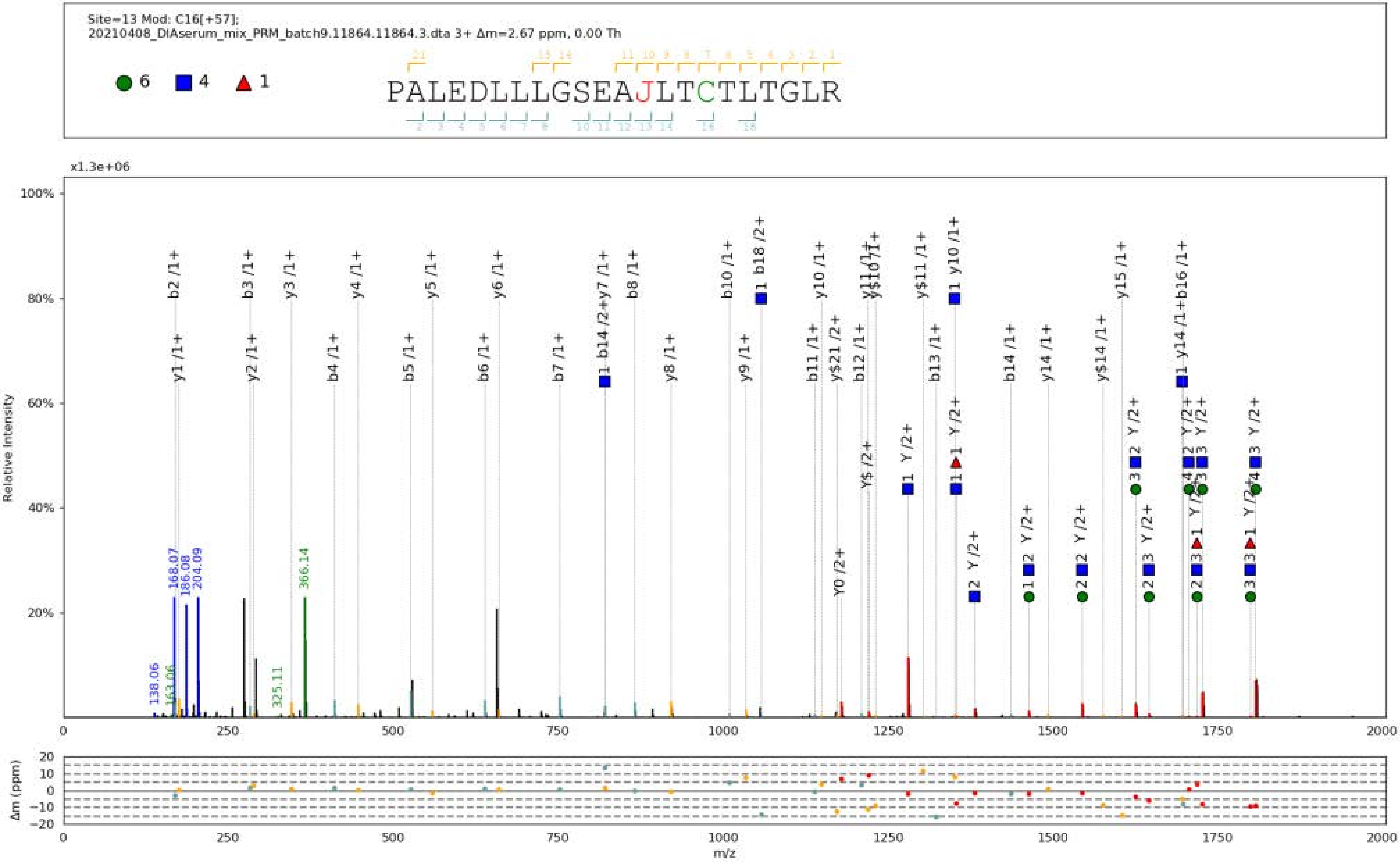

Site=13 Mod: C16[+57];
20210408_DIAserum_mix_PRM_batch9.11864.11864.3.dta 3+ Δm=2.67 ppm, 0.00 Th
PALEDLLLGSEAJLTCTLTGLR
x1.3e+06
Relative Intensity
Δm (ppm)
m/z

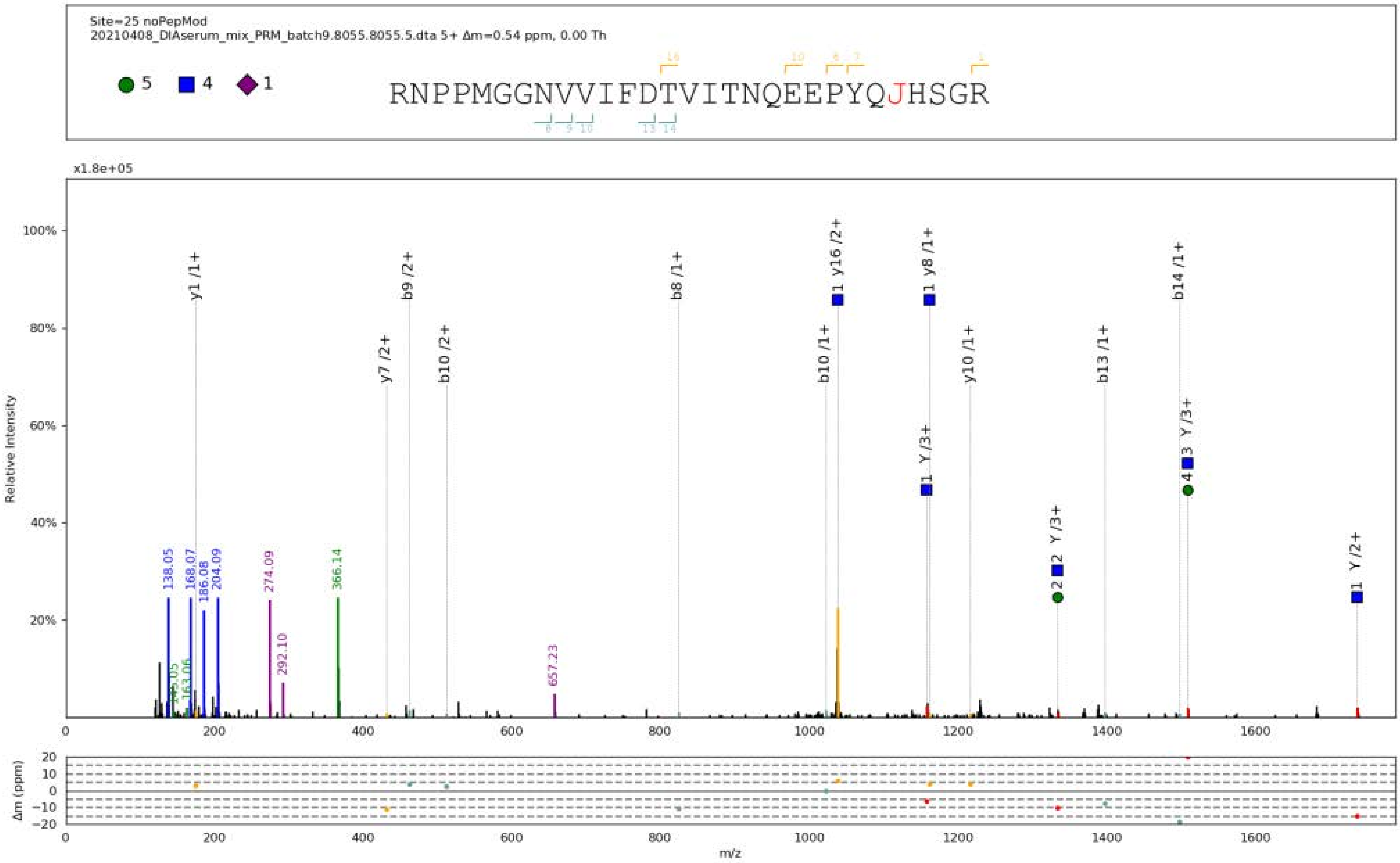

Site=25 noPepMod
20210408_DIAserum_mix_PRM_batch9.8055.8055.5.dta 5+ Δm=0.54 ppm, 0.00 Th
5
4
1
RNPPMGGNVVIFDTVITNQEEPYQJHSGR
x1.8e+05
Relative Intensity
y1 /1+
b9 /2+
y7 /2+
b10 /2+
b8 /1+
b10 /1+
1 y16 /2+
1 y8 /1+
1 Y /3+
y10 /1+
b13 /1+
b14 /1+
4 3 Y /3+
2 2 Y /3+
1 Y /2+
138.05
168.07
186.08
204.09
274.09
292.10
366.14
657.23
163.06
m/z
Δm (ppm)

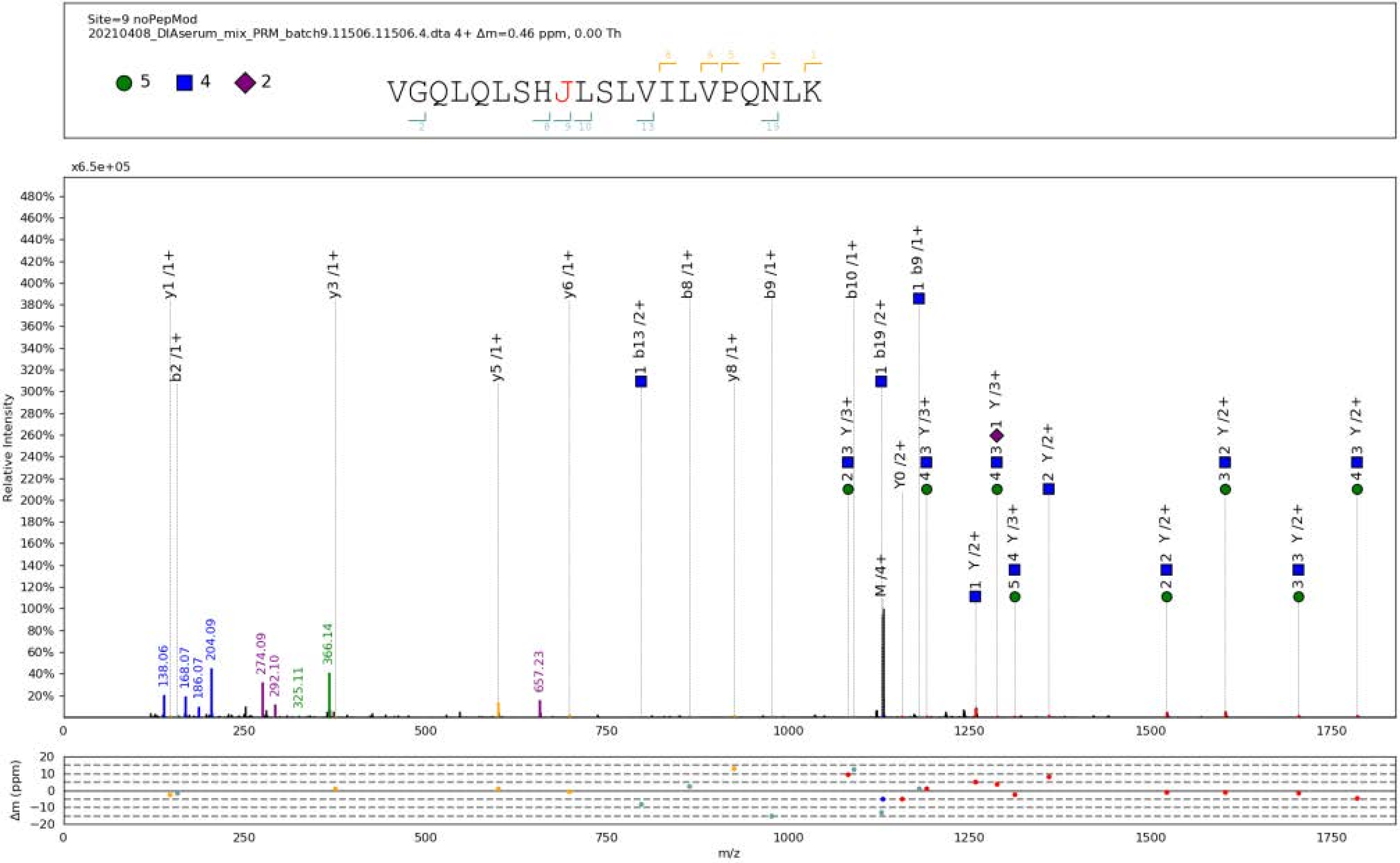

Site=9 noPepMod
20210408_DIAserum_mix_PRM_batch9.11506.11506.4.dta 4+ Δm=0.46 ppm, 0.00 Th
VGQLQLSHJLSLVILVPQNLK
x6.5e+05
Relative Intensity
m/z
Δm (ppm)
y1 /1+
b2 /1+
y3 /1+
y5 /1+
y6 /1+
b13 /2+
b8 /1+
y8 /1+
b9 /1+
b10 /1+
b19 /2+
b9 /1+
Y0 /2+
M /4+
138.06
168.07
186.07
204.09
274.09
292.10
325.11
366.14
657.23

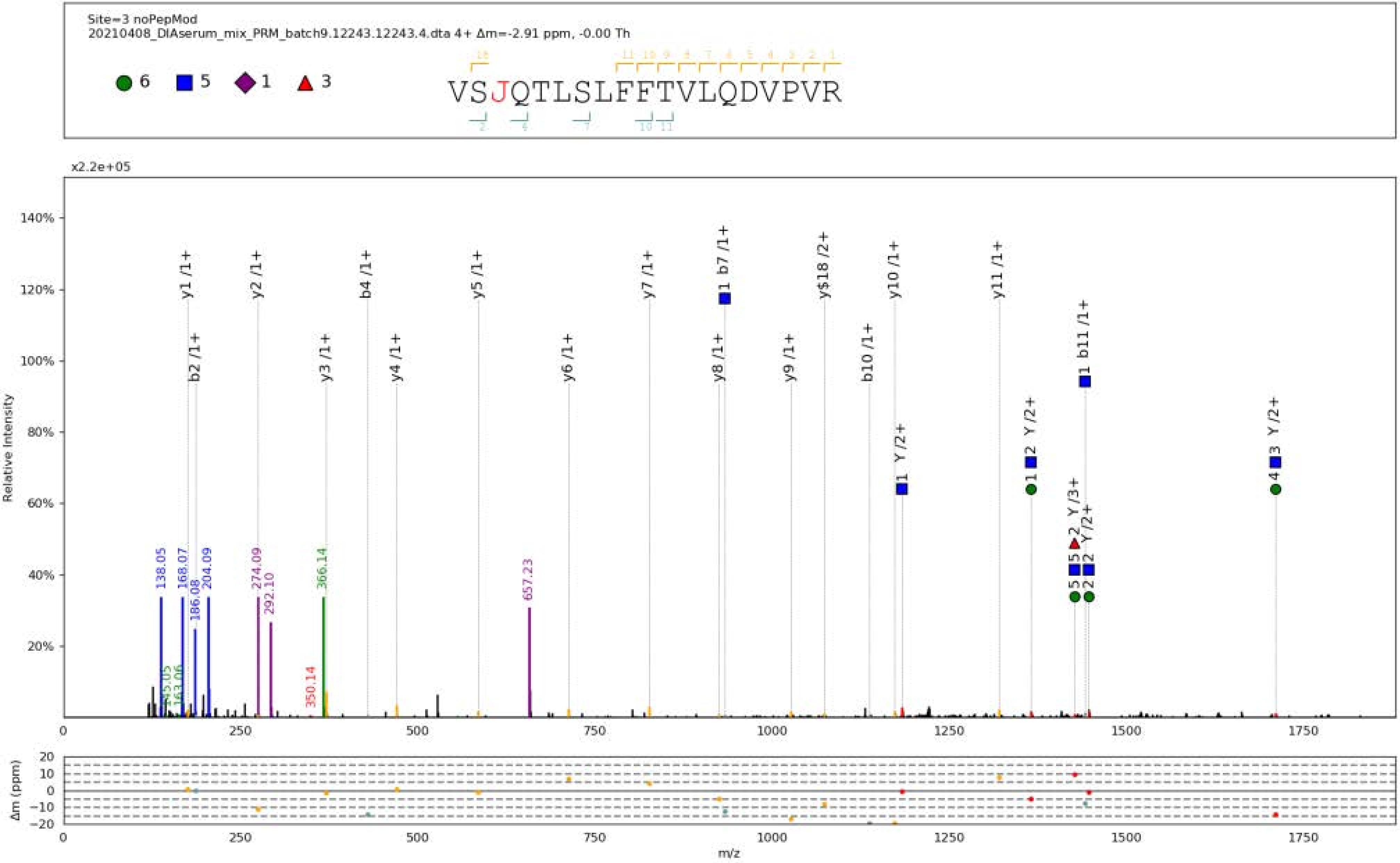
Site=3 noPepMod
20210408_DIAserum_mix_PRM_batch9.12243.12243.4.dta 4+ Δm=-2.91 ppm, -0.00 Th
VSJQTLSLFFTVLQDVPVR
x2.2e+05
Relative Intensity
Δm (ppm)
m/z

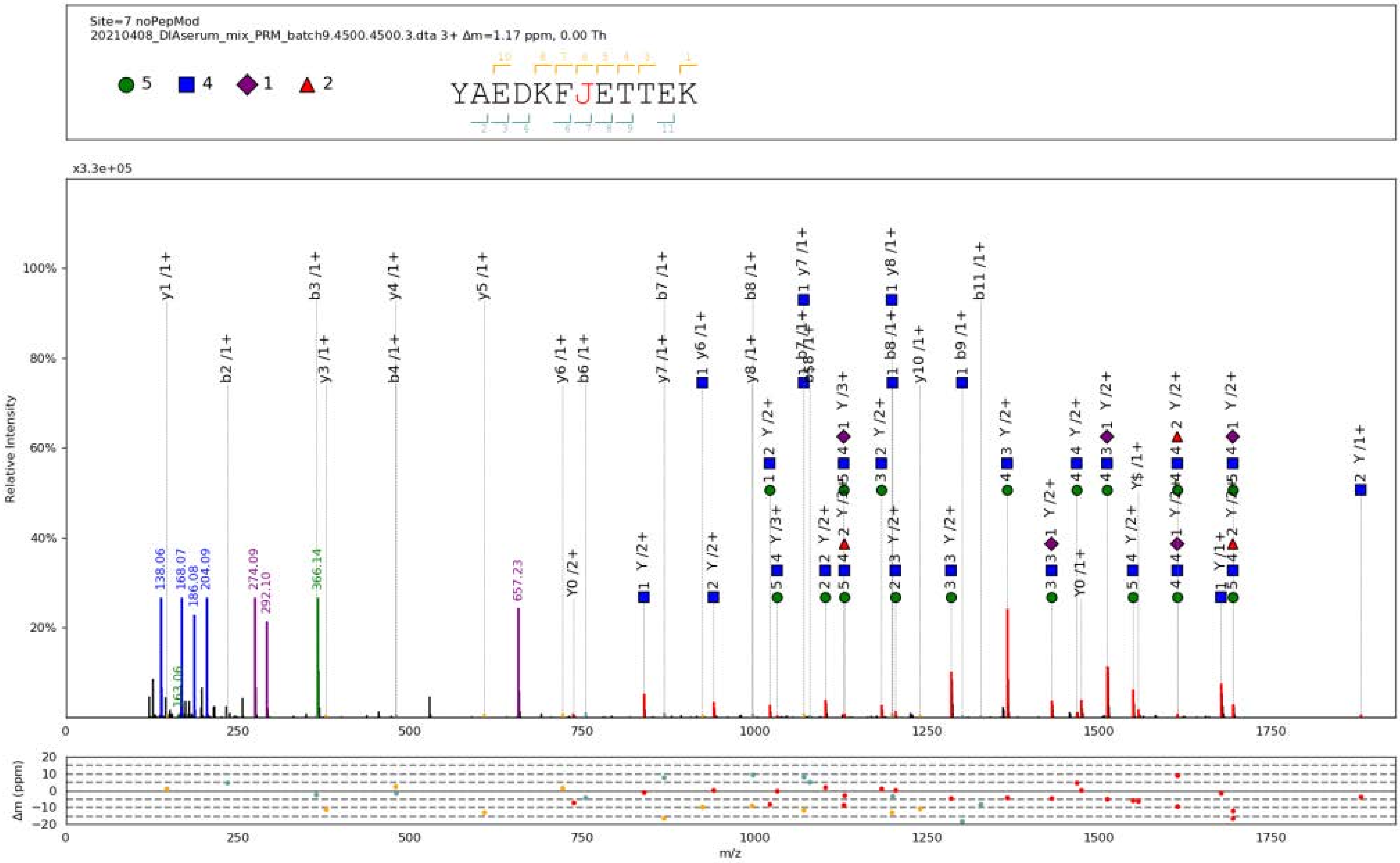
Site=7 noPepMod
20210408_DIAserum_mix_PRM_batch9.4500.4500.3.dta 3+ Δm=1.17 ppm, 0.00 Th
5
4
1
2
YAEDKFJETTEK
x3.3e+05
Relative Intensity
y1 /1+
b2 /1+
b3 /1+
y3 /1+
y4 /1+
b4 /1+
y5 /1+
y6 /1+
b6 /1+
Y0 /2+
b7 /1+
y7 /1+
1 y6 /1+
b8 /1+
y8 /1+
1 y7 /1+
1 y8 /1+
1 b8 /1+
y10 /1+
1 b9 /1+
b11 /1+
Y0 /1+
2 Y /1+
138.06
168.07
186.08
204.09
163.06
274.09
292.10
366.14
657.23
m/z
Δm (ppm)

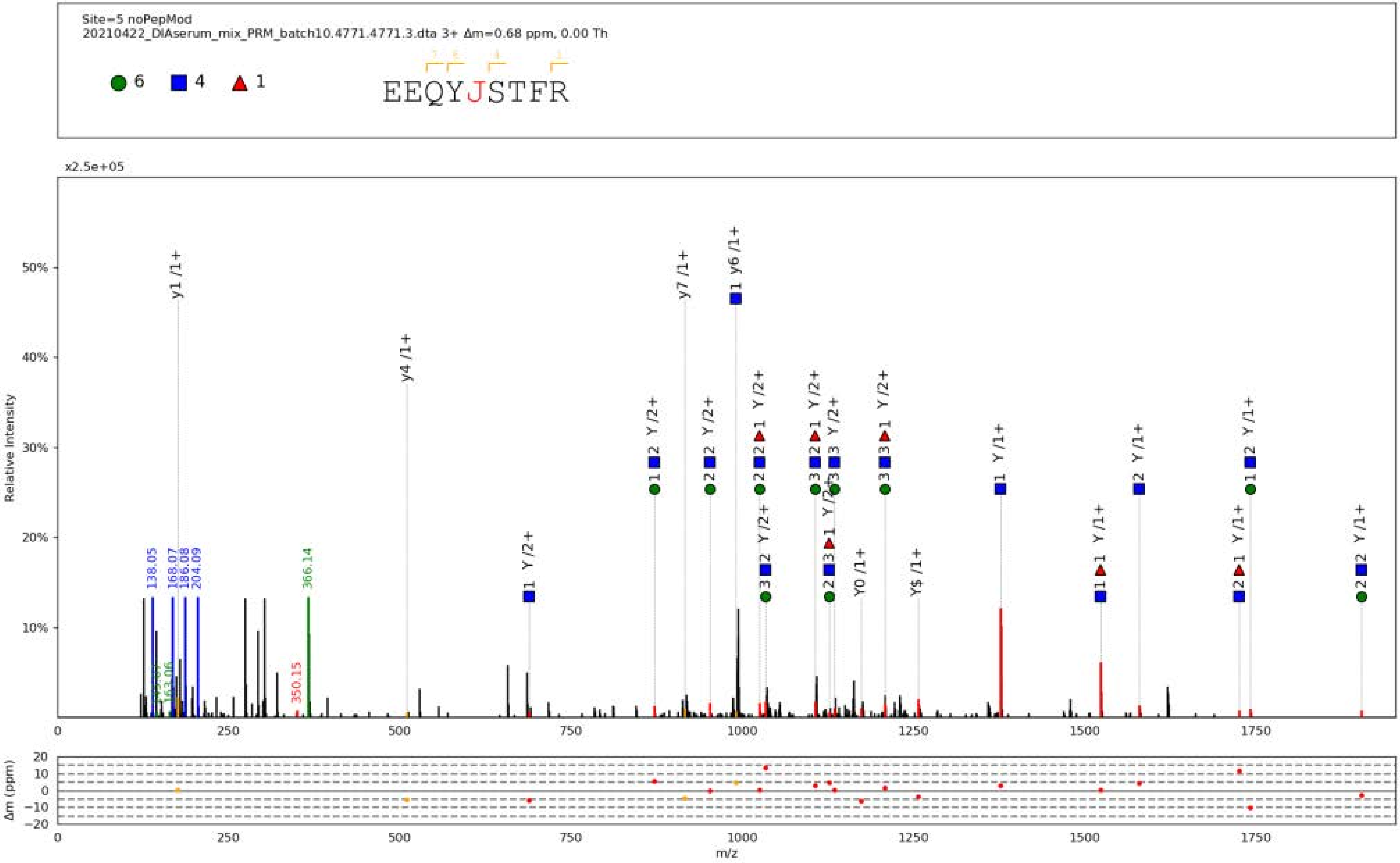

Site=5 noPepMod
20210422_DIAserum_mix_PRM_batch10.4771.4771.3.dta 3+ Δm=0.68 ppm, 0.00 Th
6
4
1
EEQYJSTFR
x2.5e+05
Relative Intensity
y1 /1+
y4 /1+
y7 /1+
1 y6 /1+
Y0 /1+
Y$ /1+
138.05
168.07
186.08
204.09
366.14
163.06
350.15
m/z
Δm (ppm)

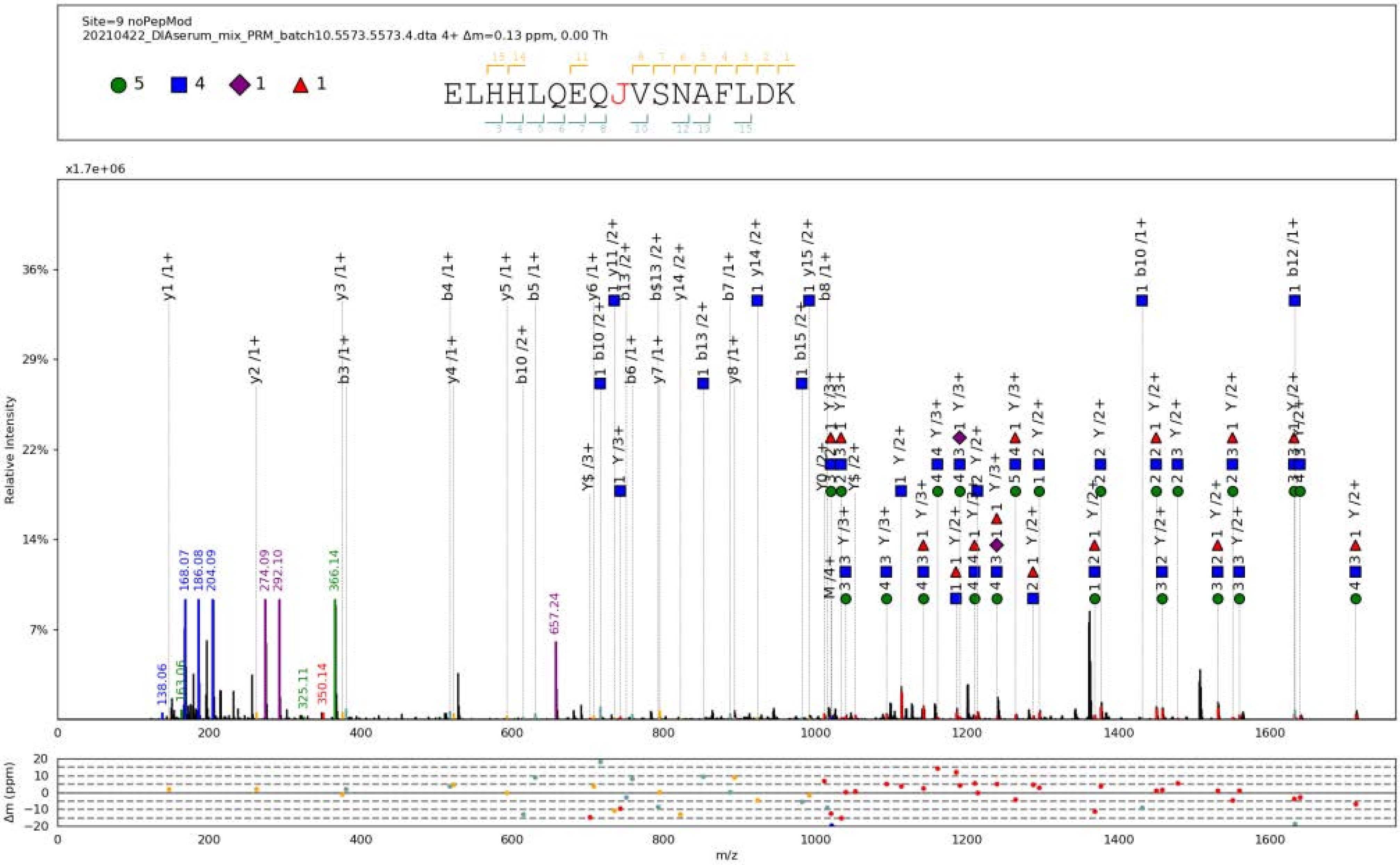

Site=9 noPepMod
20210422_DIAserum_mix_PRM_batch10.5573.5573.4.dta 4+ Δm=0.13 ppm, 0.00 Th
ELHHLQEQJVSNAFLDK
x1.7e+06
Relative Intensity
m/z
Δm (ppm)

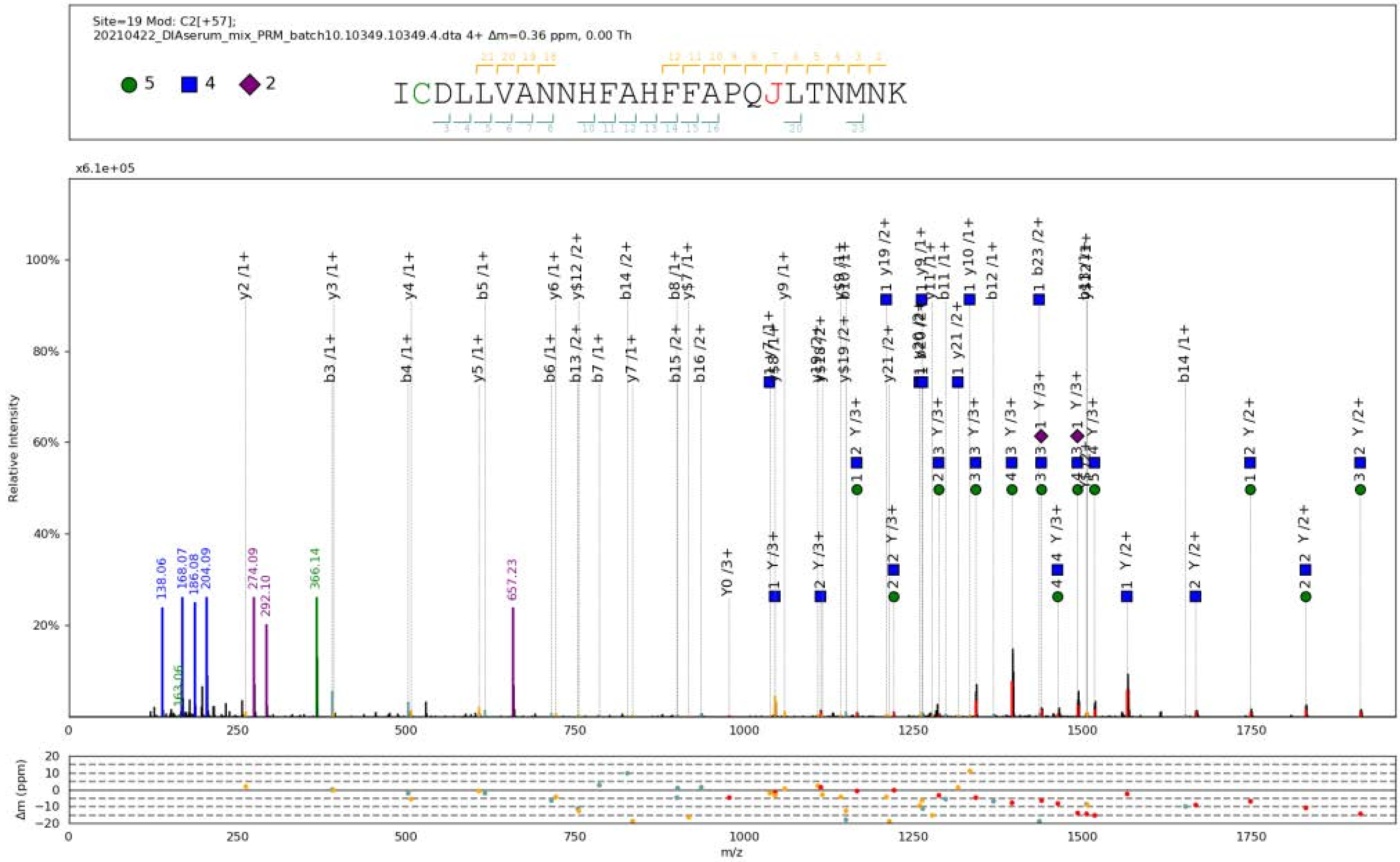

Site=19 Mod: C2[+57];
20210422_DIAserum_mix_PRM_batch10.10349.10349.4.dta 4+ Δm=0.36 ppm, 0.00 Th
5
4
2
ICDLLVANNHFAHFFAPQJLTNMNK
x6.1e+05
Relative Intensity
m/z
Δm (ppm)
138.06
168.07
186.08
204.09
163.06
274.09
292.10
366.14
657.23
y2 /1+
y3 /1+
b3 /1+
y4 /1+
b4 /1+
b5 /1+
y5 /1+
y6 /1+
b6 /1+
y$12 /2+
b13 /2+
b7 /1+
b14 /2+
y7 /1+
b8 /1+
y$7 /1+
b15 /2+
b16 /2+
y9 /1+
Y0 /3+
y21 /2+
b11 /1+
b12 /1+
b14 /1+

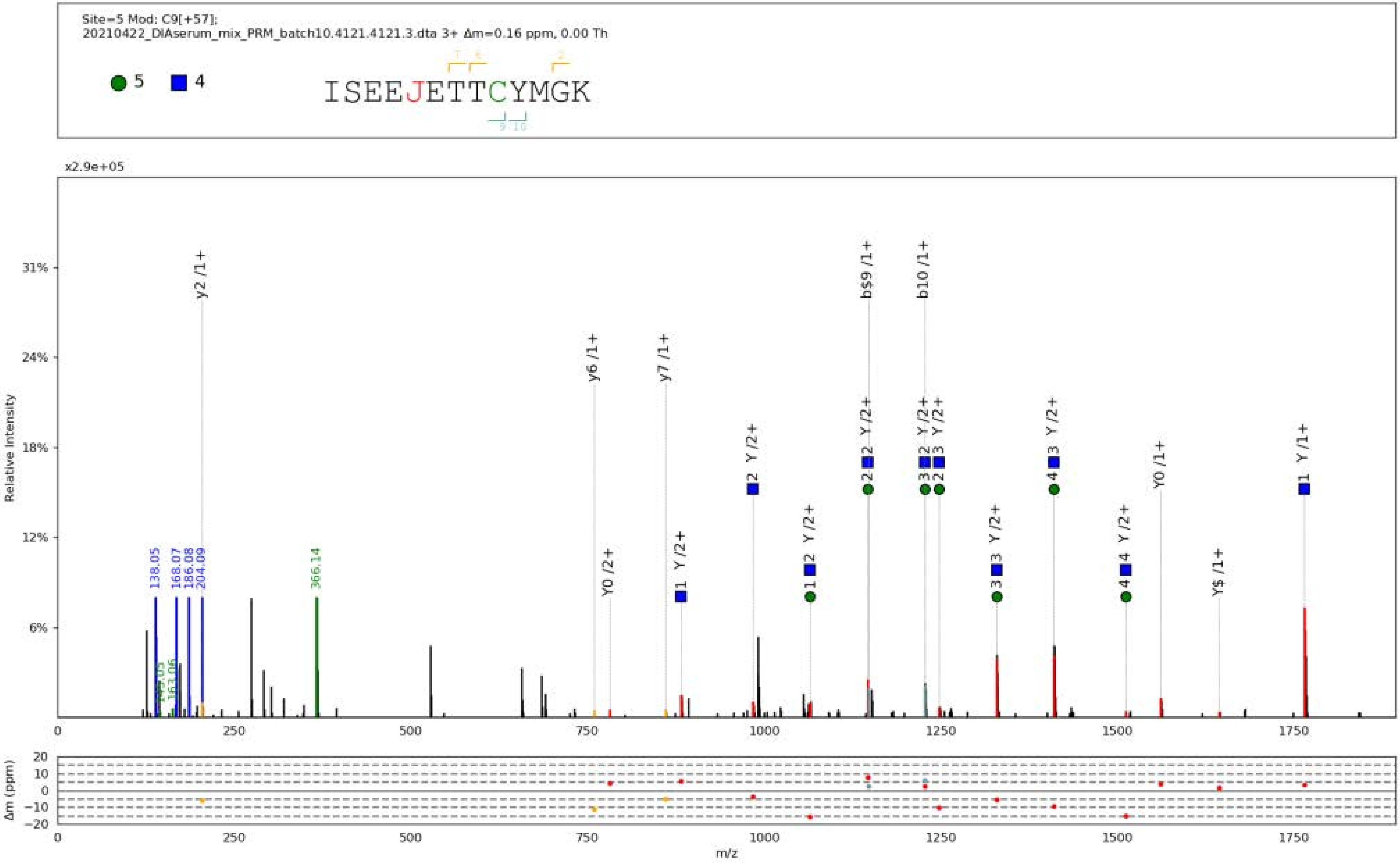

Site=5 Mod: C9[+57];
20210422_DIAserum_mix_PRM_batch10.4121.4121.3.dta 3+ Δm=0.16 ppm, 0.00 Th
5
4
ISEEJETTCYMGK
x2.9e+05
Relative Intensity
y2 /1+
y6 /1+
y7 /1+
b$9 /1+
b10 /1+
Y0 /2+
Y0 /1+
Y$ /1+
Y /2+
Y /1+
138.05
168.07
186.08
204.09
366.14
163.06
Δm (ppm)
m/z

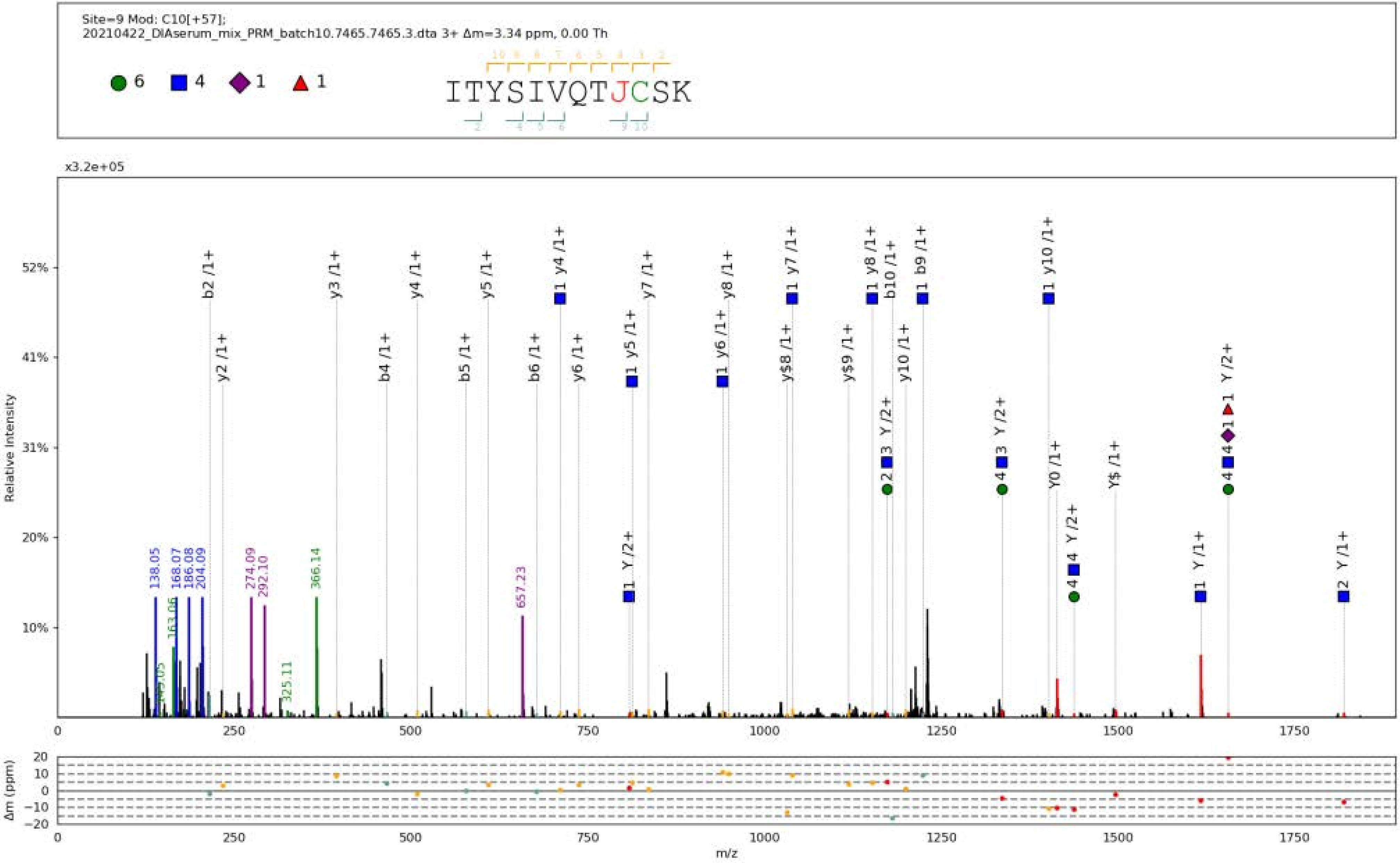
Site=9 Mod: C10[+57];
20210422_DIAserum_mix_PRM_batch10.7465.7465.3.dta 3+ Δm=3.34 ppm, 0.00 Th
6 4 1 1
ITYSIVQTJCSK
x3.2e+05
Relative Intensity
m/z
Δm (ppm)

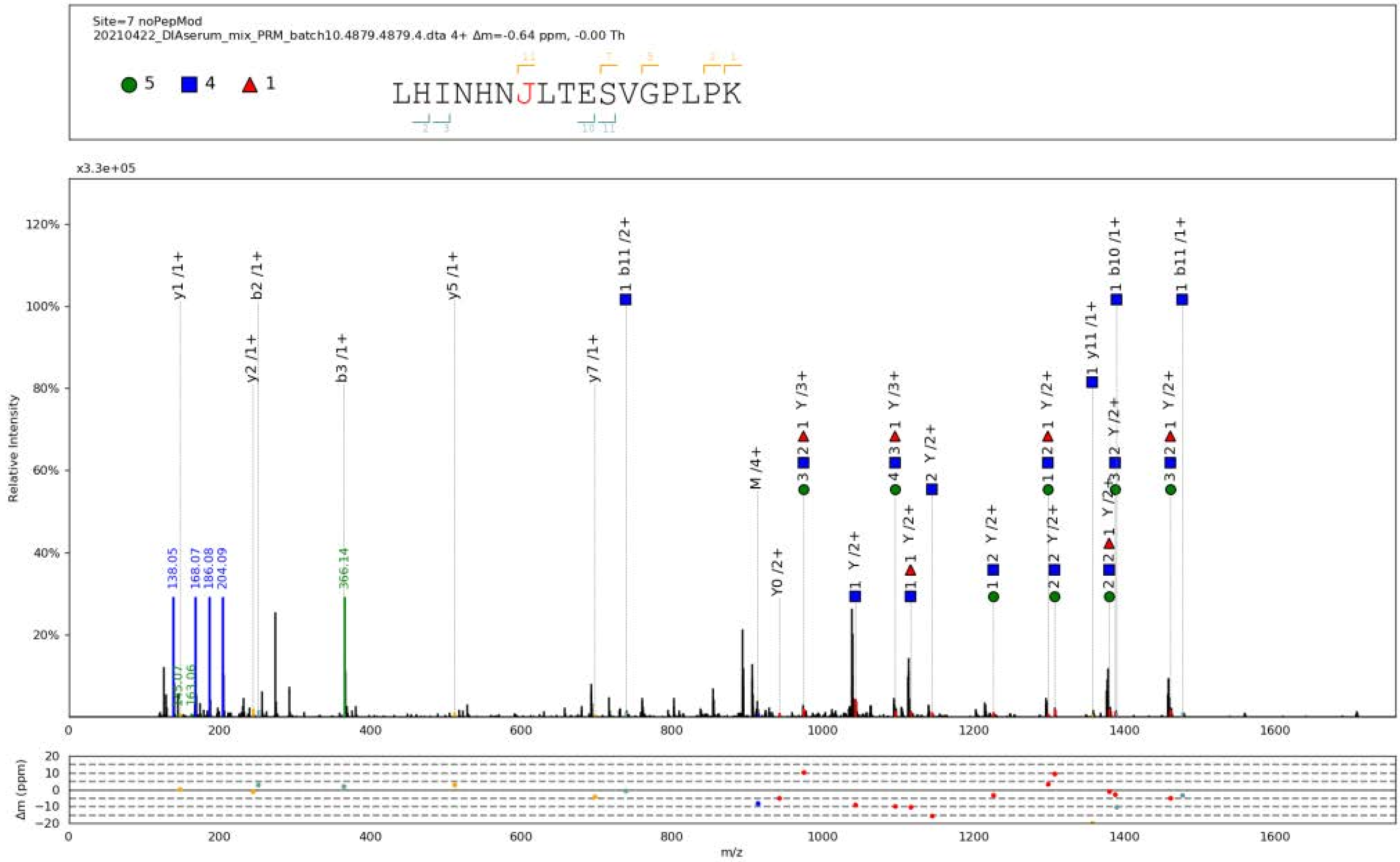

Site=7 noPepMod
20210422_DIAserum_mix_PRM_batch10.4879.4879.4.dta 4+ Δm=-0.64 ppm, -0.00 Th
LHINHNJLTESVGPLPK
x3.3e+05
Relative Intensity
m/z
Δm (ppm)

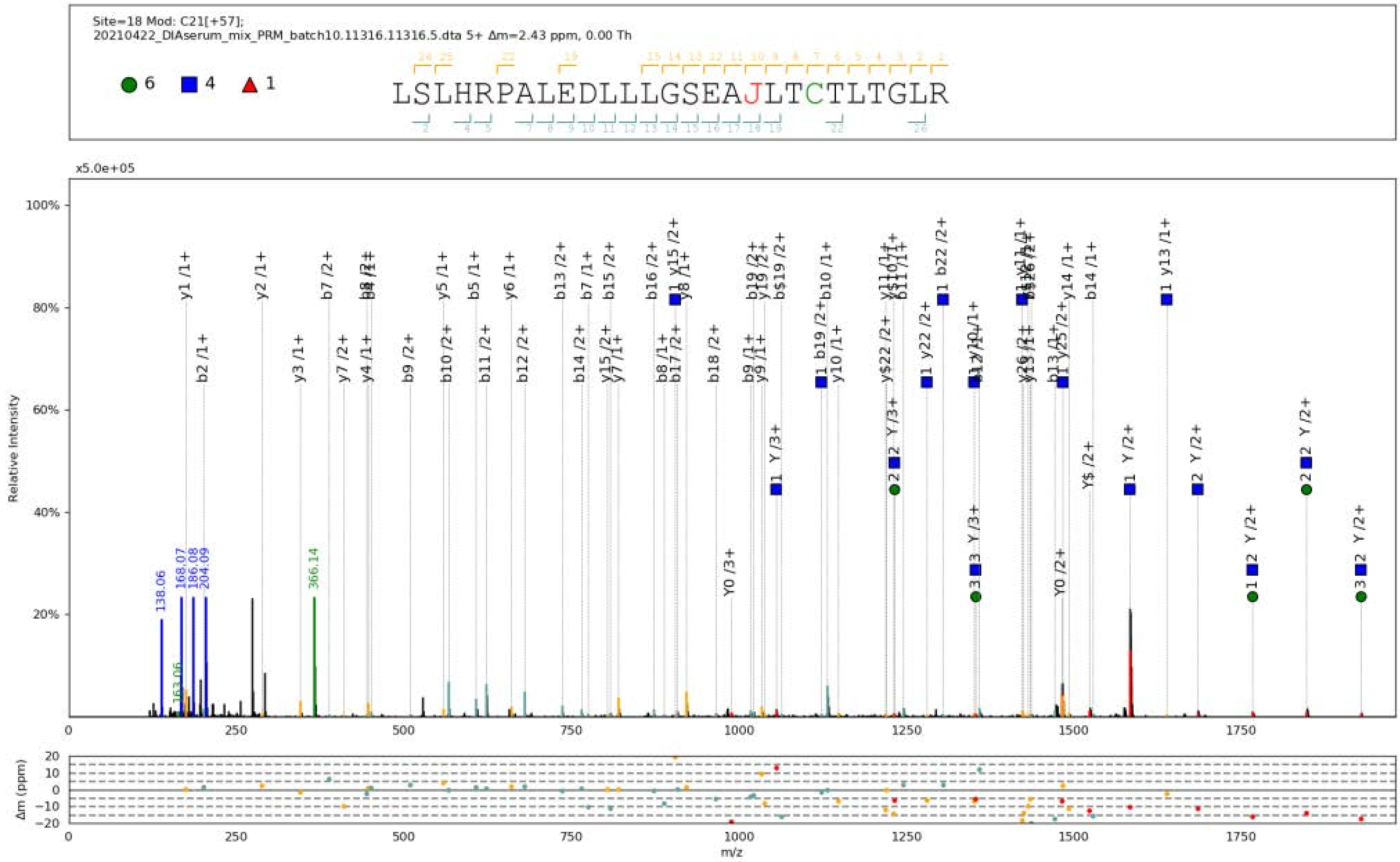

Site=18 Mod: C21[+57];
20210422_DIAserum_mix_PRM_batch10.11316.11316.5.dta 5+ Δm=2.43 ppm, 0.00 Th
6
4
1
LSLHRPALEDLLLGSEAJLTCTLTGLR
x5.0e+05
Relative Intensity
Δm (ppm)
m/z

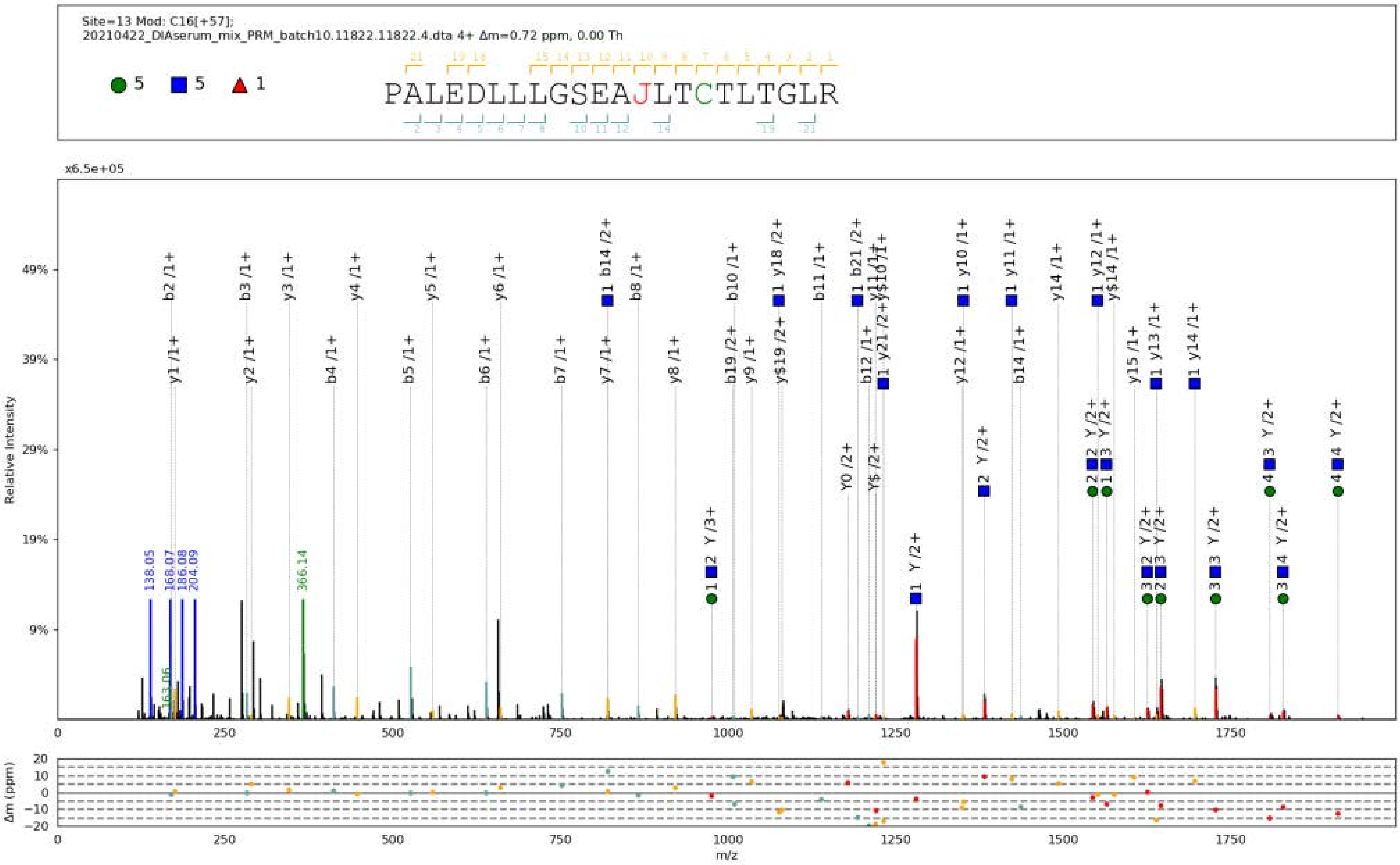

Site=13 Mod: C16[+57];
20210422_DIAserum_mix_PRM_batch10.11822.11822.4.dta 4+ Δm=0.72 ppm, 0.00 Th
PALEDLLLGSEAJLTCTLTGLR
x6.5e+05
Relative Intensity
m/z
Δm (ppm)

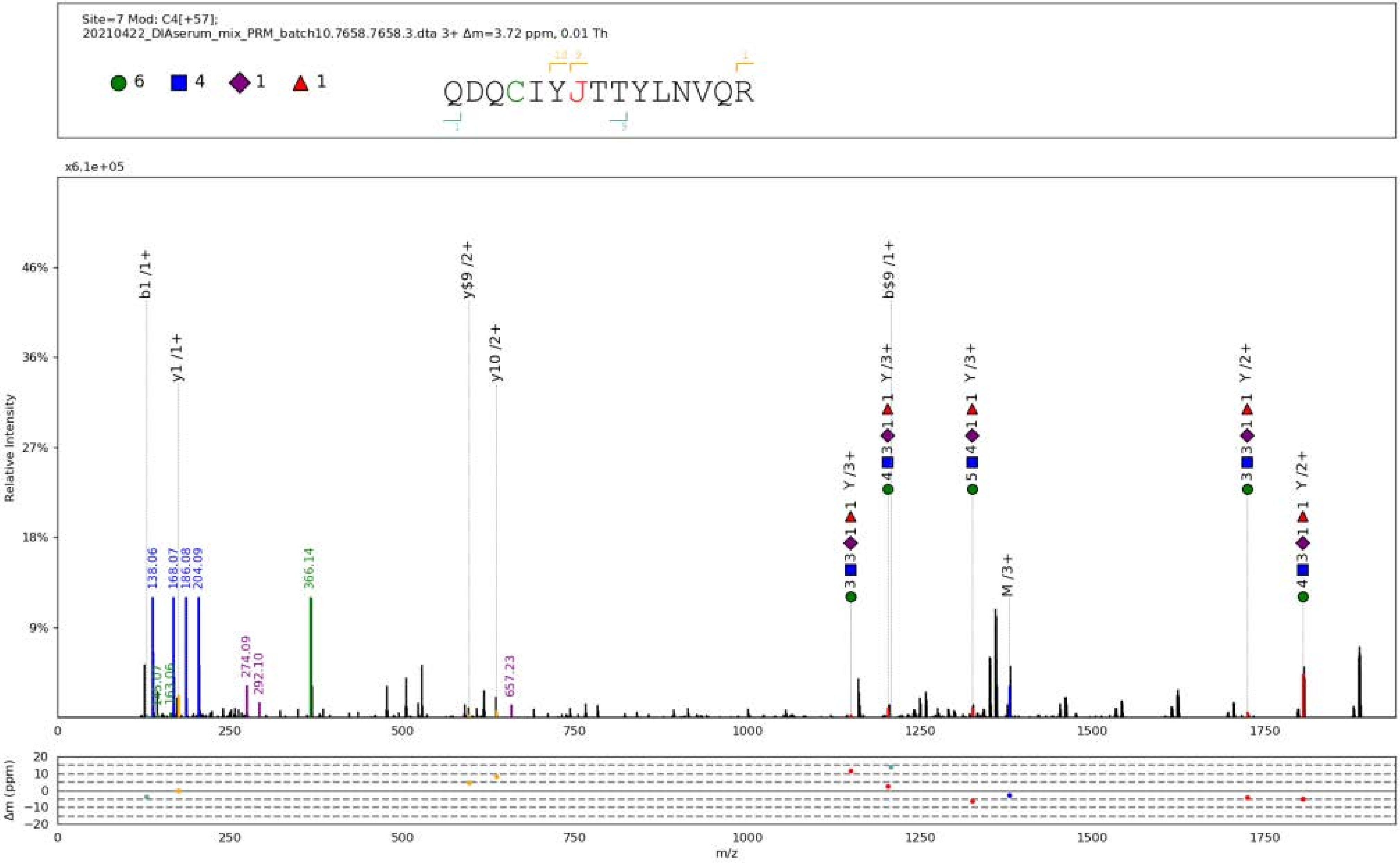

Site=7 Mod: C4[+57];
20210422_DIAserum_mix_PRM_batch10.7658.7658.3.dta 3+ Δm=3.72 ppm, 0.01 Th
6 4 1 1
QDQCIYJTTYLNVQR
x6.1e+05
Relative Intensity
b1 /1+
y1 /1+
y$9 /2+
y10 /2+
b$9 /1+
M /3+
138.06
168.07
186.08
204.09
366.14
274.09
292.10
657.23
m/z
Δm (ppm)

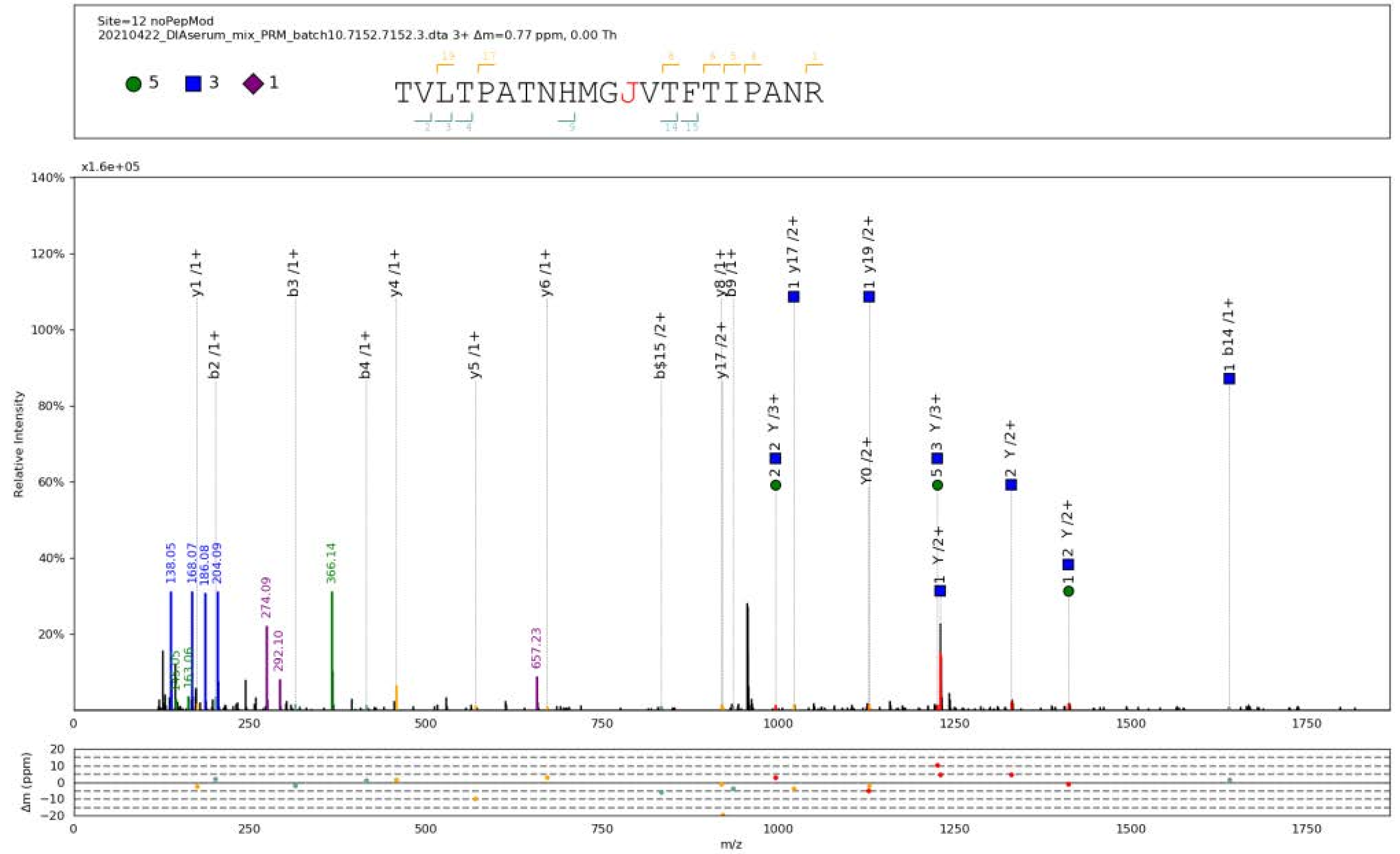

Site=12 noPepMod
20210422_DIAserum_mix_PRM_batch10.7152.7152.3.dta 3+ Δm=0.77 ppm, 0.00 Th
5
3
1
TVLTPATNHMGJVTFTIPANR
x1.6e+05
Relative Intensity
y1 /1+
b2 /1+
b3 /1+
b4 /1+
y4 /1+
y5 /1+
y6 /1+
b$15 /2+
y17 /2+
y8 /1+
b9 /1+
2 2 Y /3+
1 y17 /2+
1 y19 /2+
Y0 /2+
5 3 Y /3+
1 Y /2+
2 Y /2+
1 2 Y /2+
1 b14 /1+
138.05
168.07
186.08
204.09
163.06
274.09
292.10
366.14
657.23
m/z
Δm (ppm)

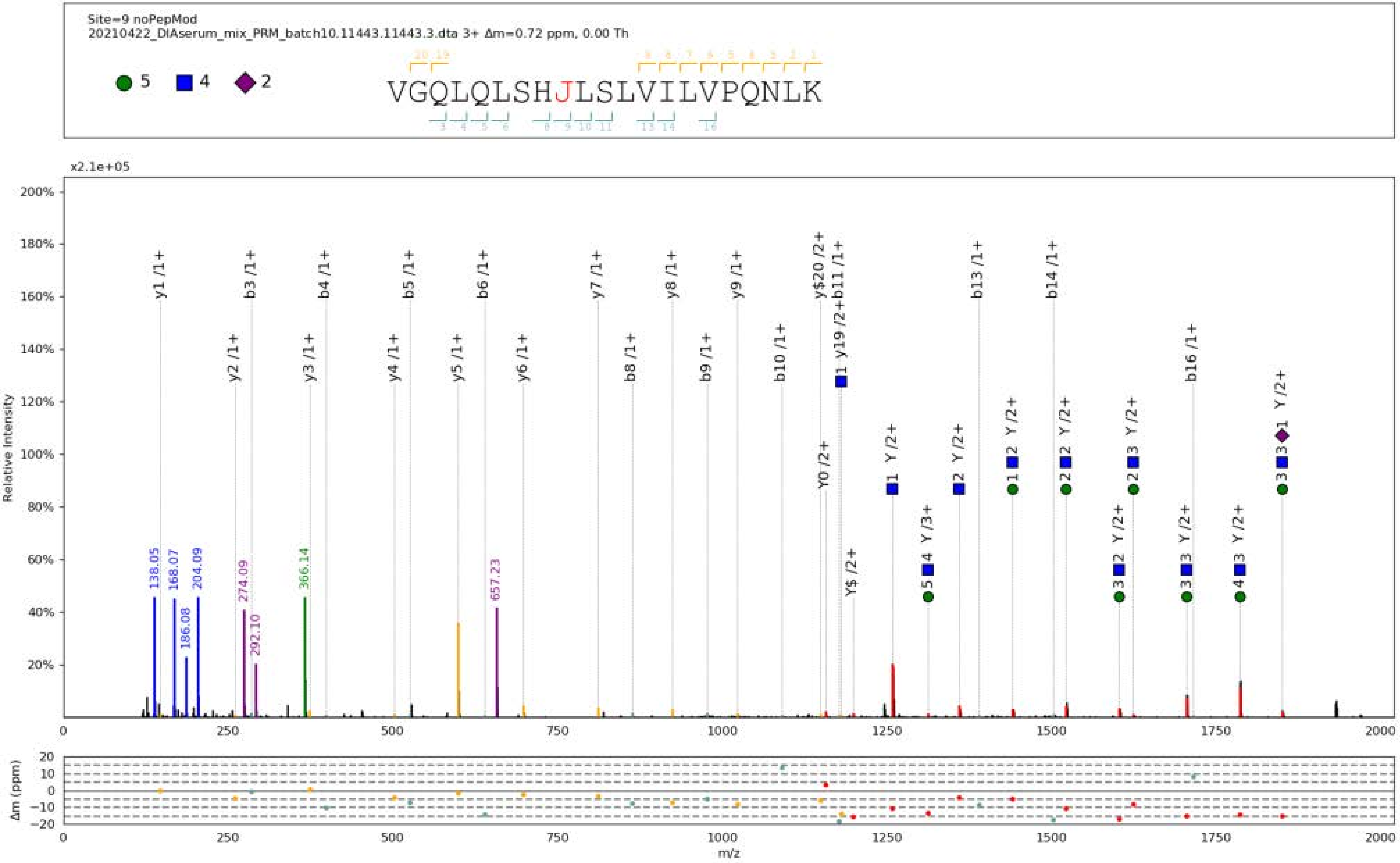
Site=9 noPepMod
20210422_DIAserum_mix_PRM_batch10.11443.11443.3.dta 3+ Δm=0.72 ppm, 0.00 Th
VGQLQLSHJLSLVILVPQNLK
x2.1e+05
Relative Intensity
m/z
Δm (ppm)

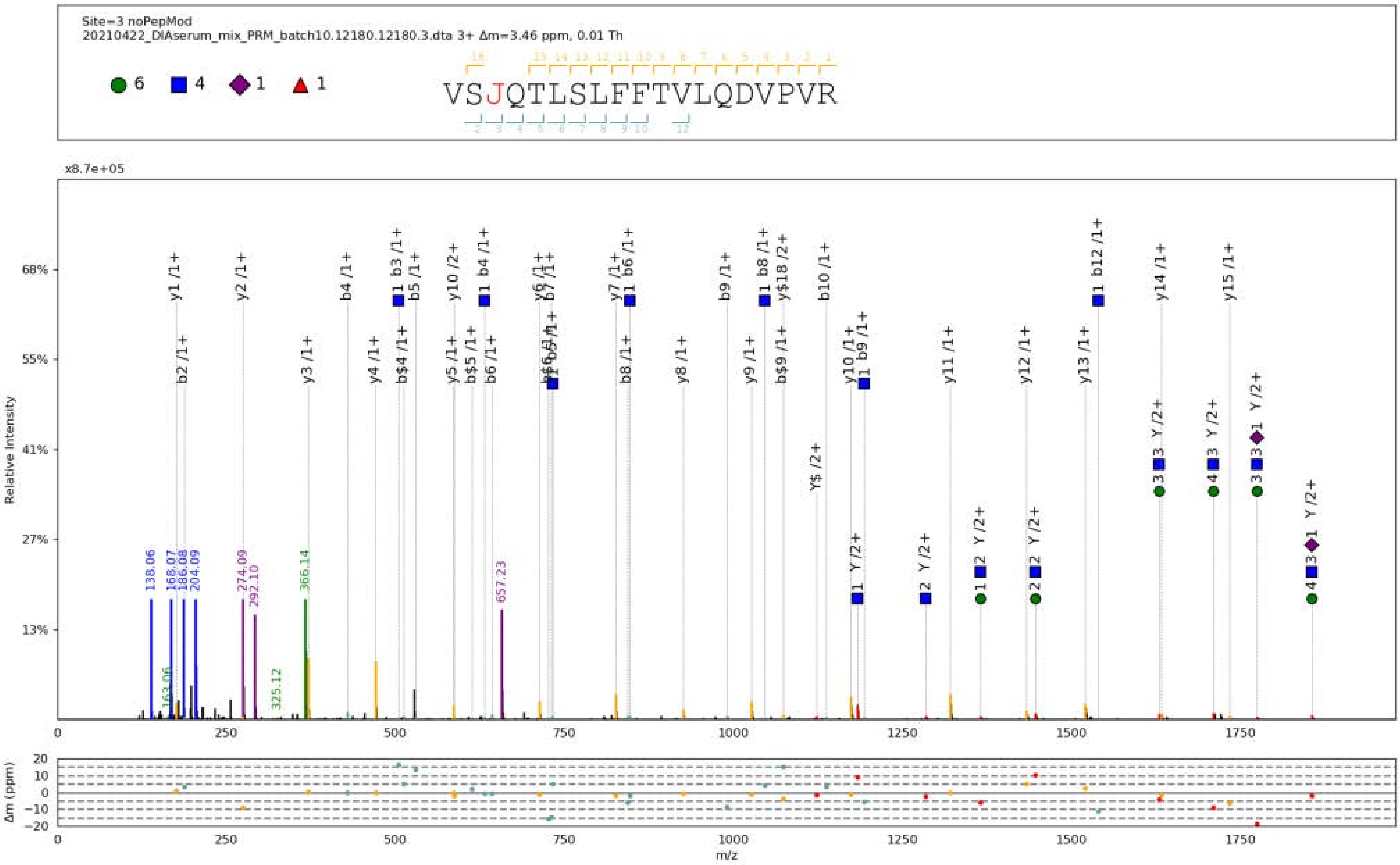

Site=3 noPepMod
20210422_DIAserum_mix_PRM_batch10.12180.12180.3.dta 3+ Δm=3.46 ppm, 0.01 Th
6
4
1
1
VSJQTLSLFFTVLQDVPVR
x8.7e+05
Relative Intensity
m/z
Δm (ppm)

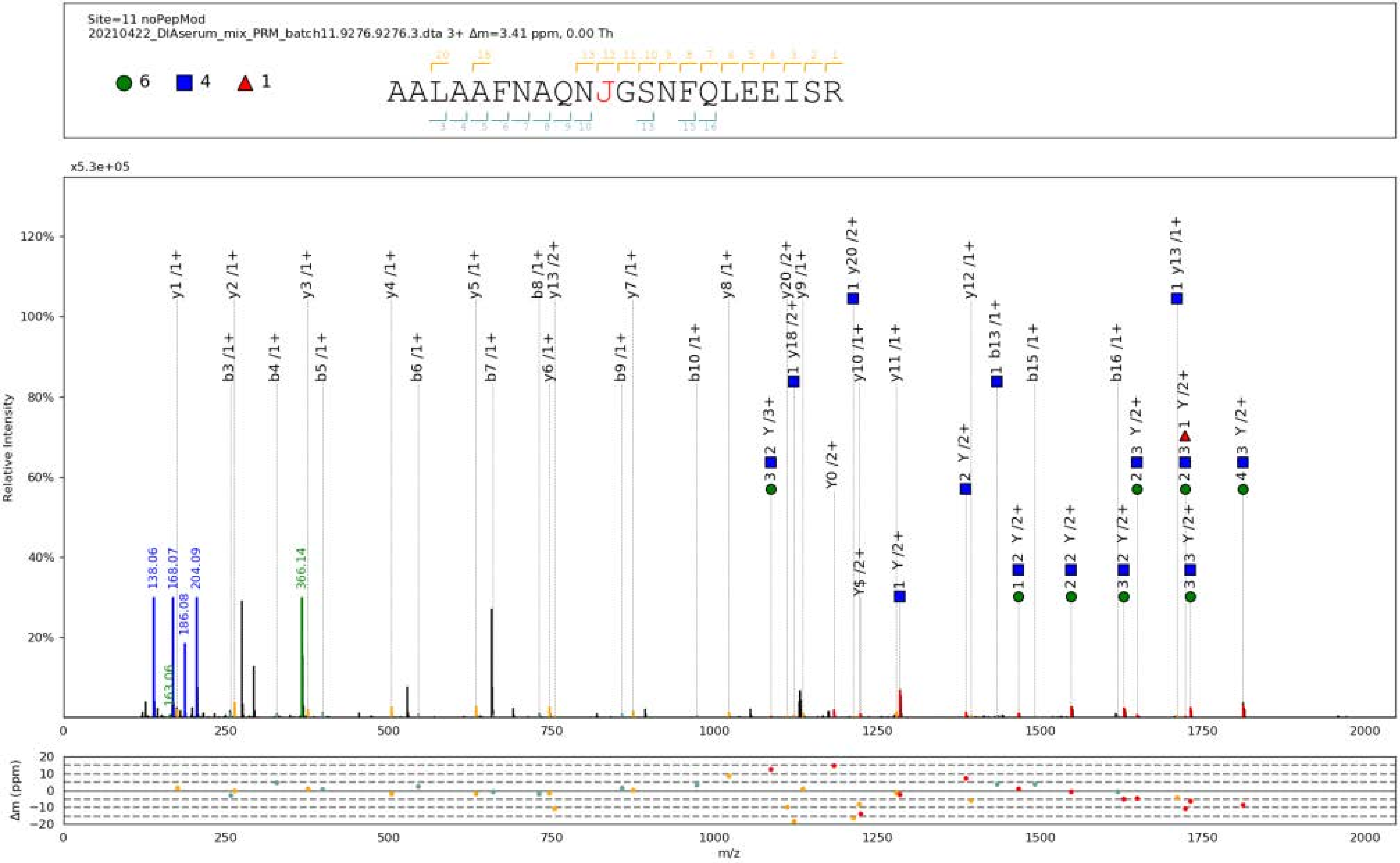

Site=11 noPepMod
20210422_DIAserum_mix_PRM_batch11.9276.9276.3.dta 3+ Δm=3.41 ppm, 0.00 Th
6
4
1
AALAAFNAQNJGSNFQLEEISR
x5.3e+05
Relative Intensity
y1 /1+
y2 /1+
b3 /1+
b4 /1+
y3 /1+
b5 /1+
y4 /1+
b6 /1+
y5 /1+
b7 /1+
b8 /1+
y13 /2+
y6 /1+
b9 /1+
y7 /1+
b10 /1+
y8 /1+
y20 /2+
y18 /2+
y9 /1+
Y0 /2+
y20 /2+
y10 /1+
Y$ /2+
y11 /1+
y12 /1+
b13 /1+
b15 /1+
b16 /1+
y13 /1+
138.06
168.07
186.08
204.09
163.06
366.14
m/z
Δm (ppm)

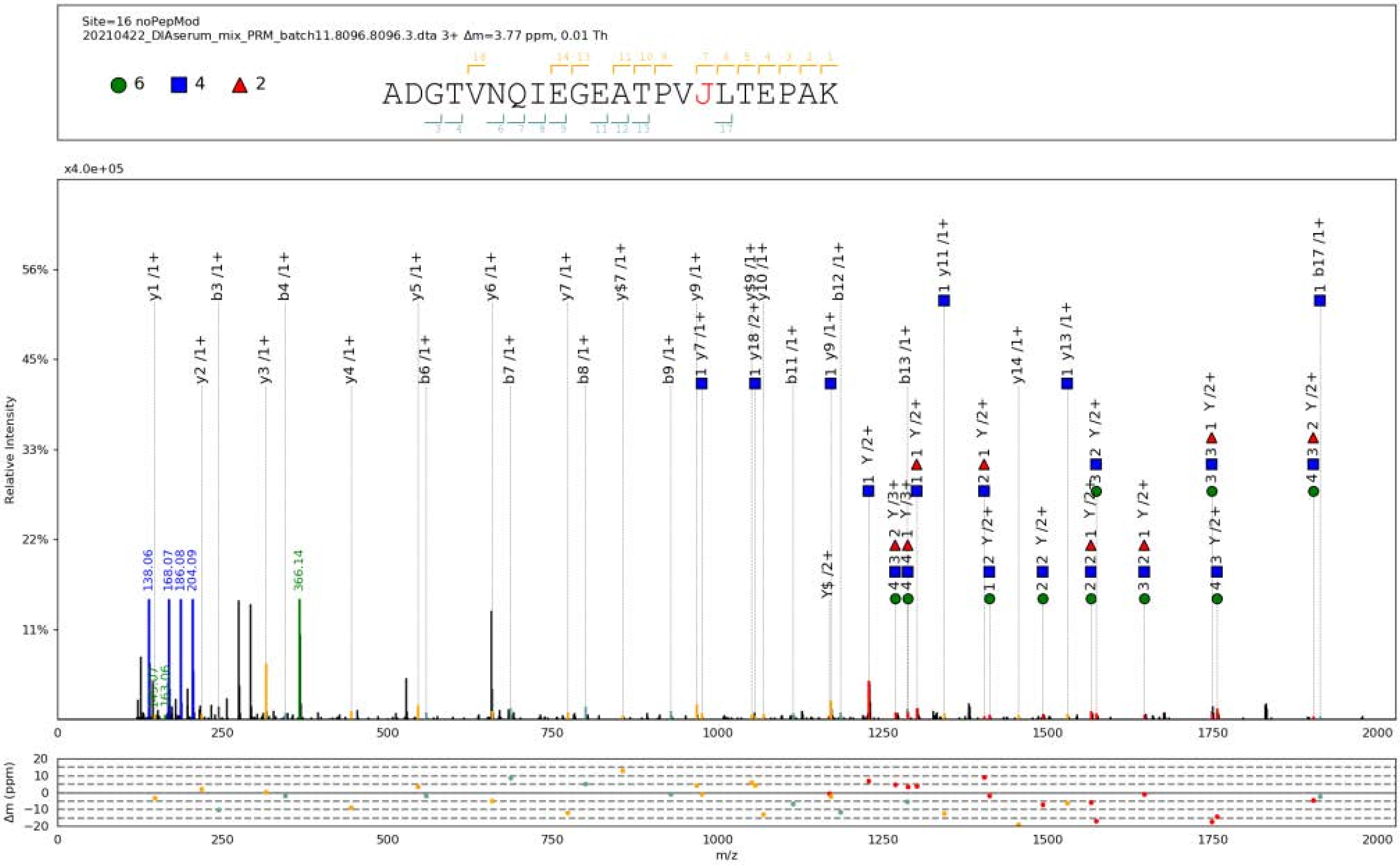
Site=16 noPepMod
20210422_DIAserum_mix_PRM_batch11.8096.8096.3.dta 3+ Δm=3.77 ppm, 0.01 Th
ADGTVNQIEGEATPVJLTEPAK
x4.0e+05
Relative Intensity
Δm (ppm)
m/z

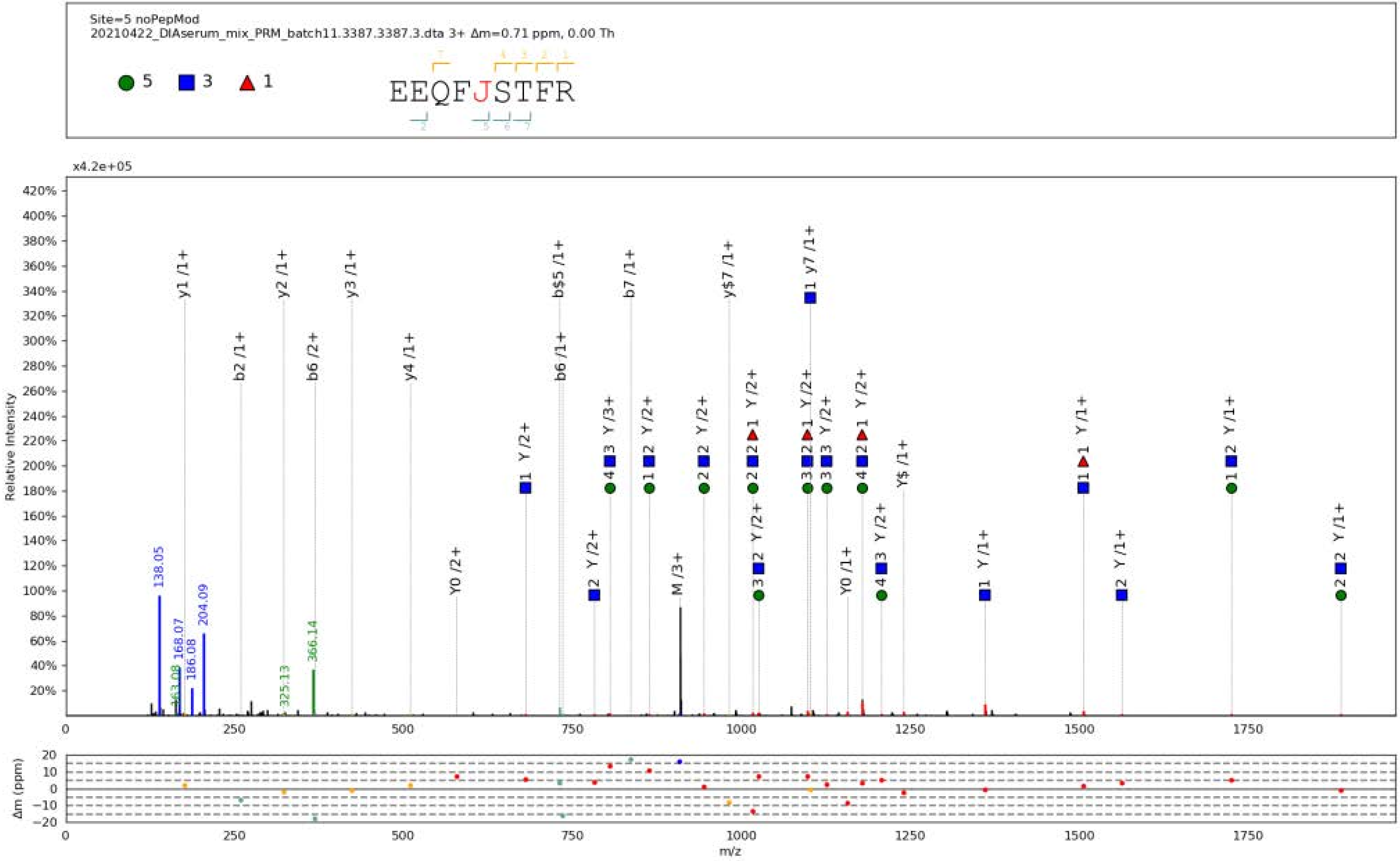
Site=5 noPepMod
20210422_DIAserum_mix_PRM_batch11.3387.3387.3.dta 3+ Δm=0.71 ppm, 0.00 Th
5
3
1
EEQFJSTFR
x4.2e+05
Relative Intensity
m/z
Δm (ppm)

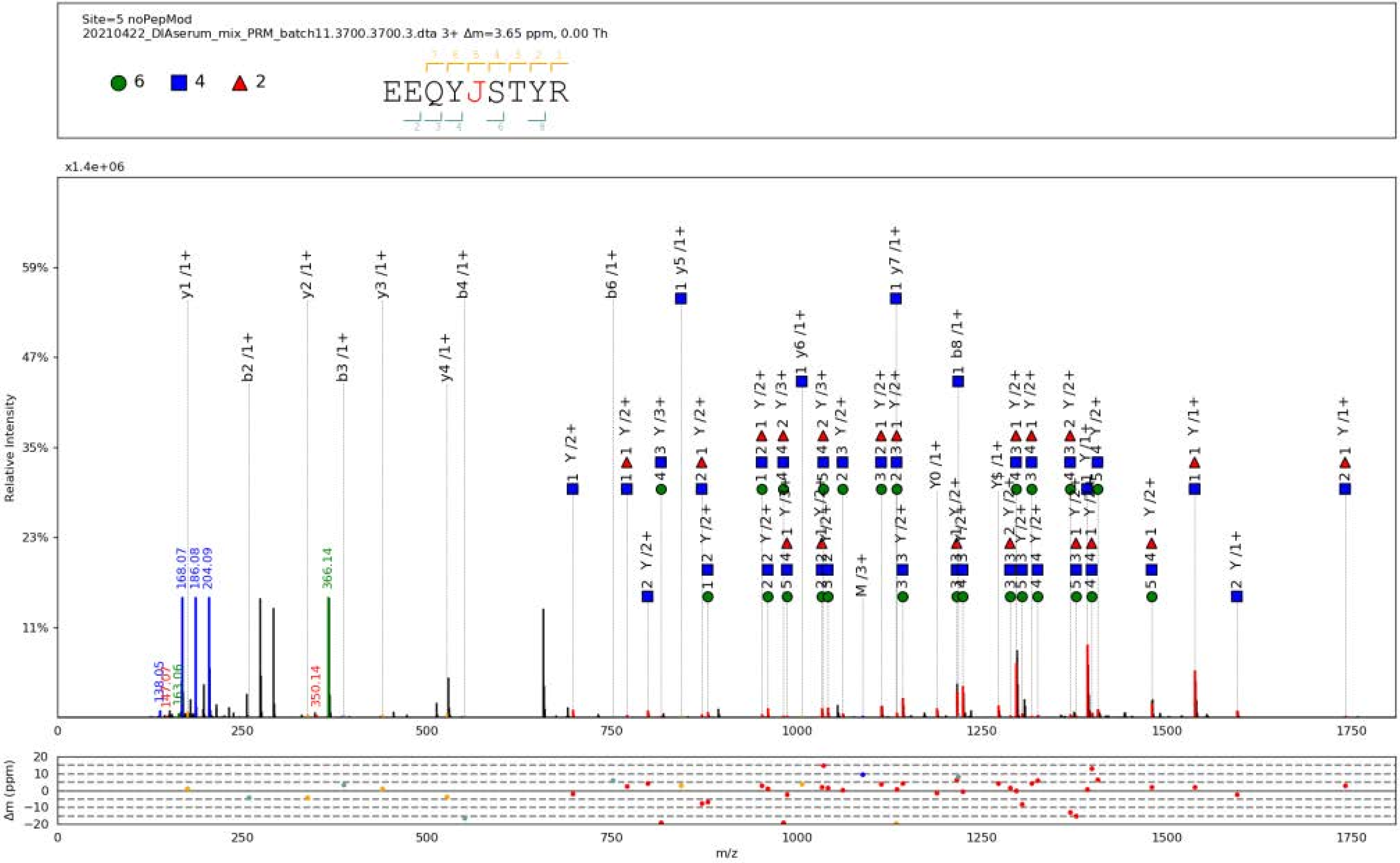
Site=5 noPepMod
20210422_DIAserum_mix_PRM_batch11.3700.3700.3.dta 3+ Δm=3.65 ppm, 0.00 Th
6
4
2
EEQYJSTYR
x1.4e+06
Relative Intensity
59%
47%
35%
23%
11%
y1 /1+
b2 /1+
y2 /1+
b3 /1+
y3 /1+
y4 /1+
b4 /1+
b6 /1+
1 y5 /1+
1 y6 /1+
1 y7 /1+
1 b8 /1+
Y0 /1+
M /3+
168.07
186.08
204.09
366.14
350.14
138.05
147.07
163.06
0
250
500
750
1000
1250
1500
1750
Δm (ppm)
m/z

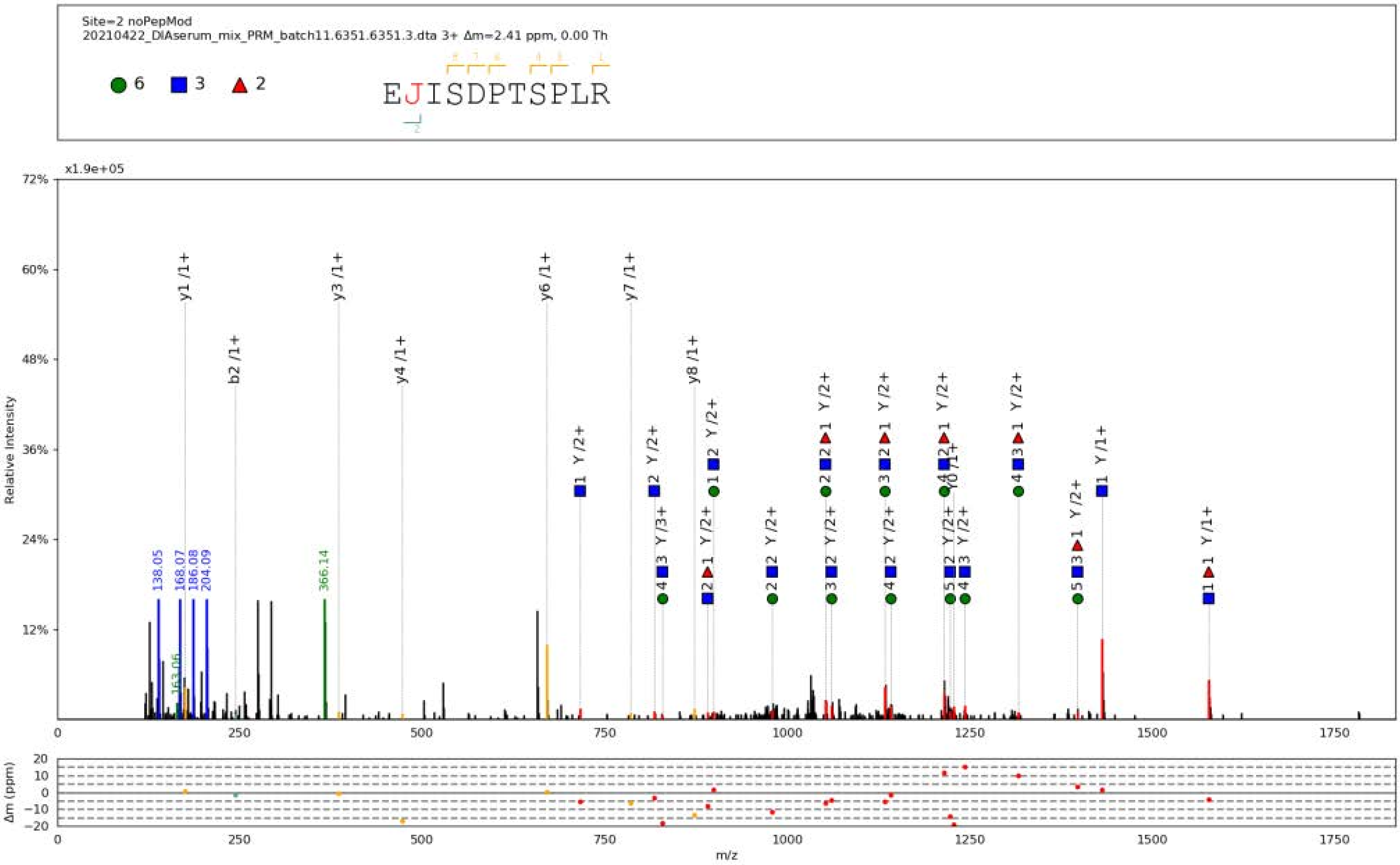
Site=2 noPepMod
20210422_DIAserum_mix_PRM_batch11.6351.6351.3.dta 3+ Δm=2.41 ppm, 0.00 Th
6
3
2
EJISDPTSPLR
x1.9e+05
Relative Intensity
m/z
Δm (ppm)
y1 /1+
b2 /1+
y3 /1+
y4 /1+
y6 /1+
y7 /1+
y8 /1+
138.05
168.07
186.08
204.09
366.14
163.06

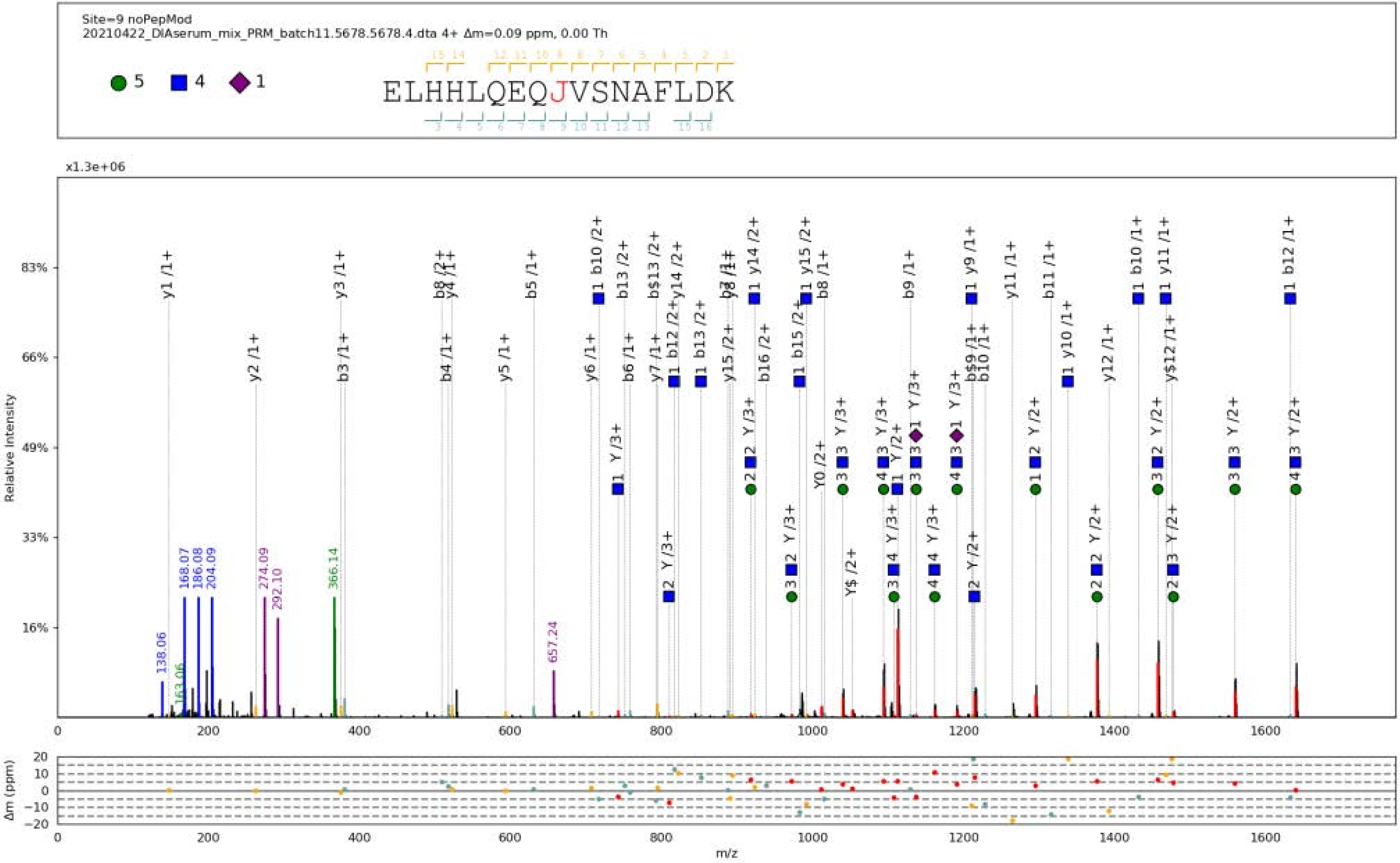
Site=9 noPepMod
20210422_DIAserum_mix_PRM_batch11.5678.5678.4.dta 4+ Δm=0.09 ppm, 0.00 Th
5
4
1
ELHHLQEQJVSNAFLDK
x1.3e+06
Relative Intensity
83%
66%
49%
33%
16%
m/z
Δm (ppm)

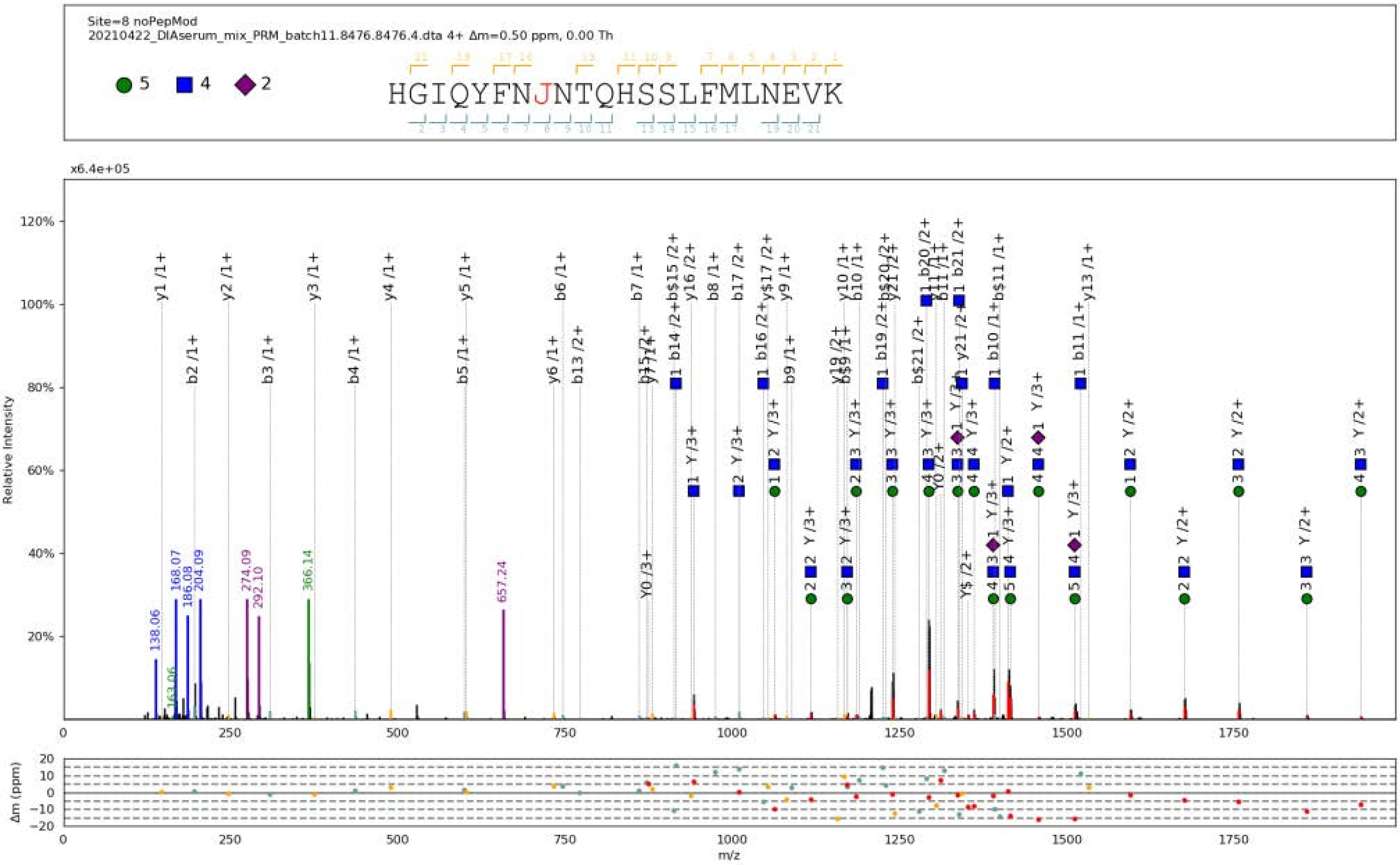

Site=8 noPepMod
20210422_DIAserum_mix_PRM_batch11.8476.8476.4.dta 4+ Δm=0.50 ppm, 0.00 Th
5
4
2
HGIQYFNJNTQHSSLFMLNEVK
x6.4e+05
Relative Intensity
y1 /1+
b2 /1+
y2 /1+
b3 /1+
y3 /1+
b4 /1+
y4 /1+
b5 /1+
y5 /1+
y6 /1+
b6 /1+
b13 /2+
b7 /1+
Y0 /3+
b14 /2+
b$15 /2+
y16 /2+
b8 /1+
b17 /2+
b16 /2+
y$17 /2+
y9 /1+
b9 /1+
y10 /1+
b10 /1+
b19 /2+
b$20 /2+
b$21 /2+
b20 /2+
b21 /2+
y21 /2+
b10 /1+
b$11 /1+
b11 /1+
y13 /1+
Y0 /2+
Y$ /2+
Y /3+
Y /2+
138.06
163.06
168.07
186.08
204.09
274.09
292.10
366.14
657.24
Δm (ppm)
m/z

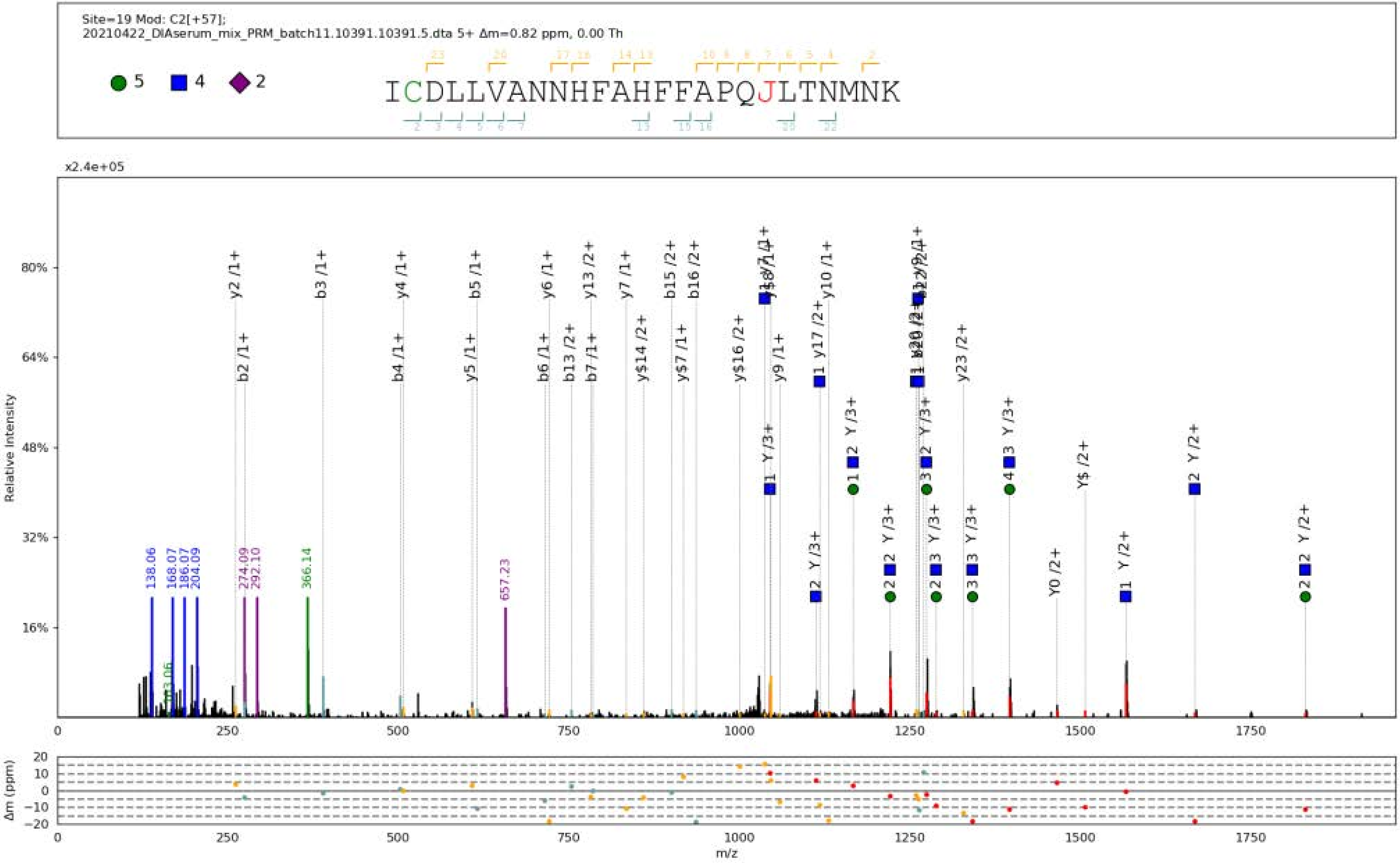

Site=19 Mod: C2[+57];
20210422_DIAserum_mix_PRM_batch11.10391.10391.5.dta 5+ Δm=0.82 ppm, 0.00 Th
5
4
2
ICDLLVANNHFAHFFAPQJLTNMNK
x2.4e+05
Relative Intensity
80%
64%
48%
32%
16%
y2 /1+
b2 /1+
b3 /1+
y4 /1+
b4 /1+
b5 /1+
y5 /1+
y6 /1+
b6 /1+
y13 /2+
b13 /2+
b7 /1+
y7 /1+
y$14 /2+
b15 /2+
y$7 /1+
b16 /2+
y$16 /2+
y9 /1+
y17 /2+
y10 /1+
y23 /2+
Y0 /2+
Y$ /2+
138.06
168.07
186.07
204.09
274.09
292.10
366.14
657.23
m/z
Δm (ppm)

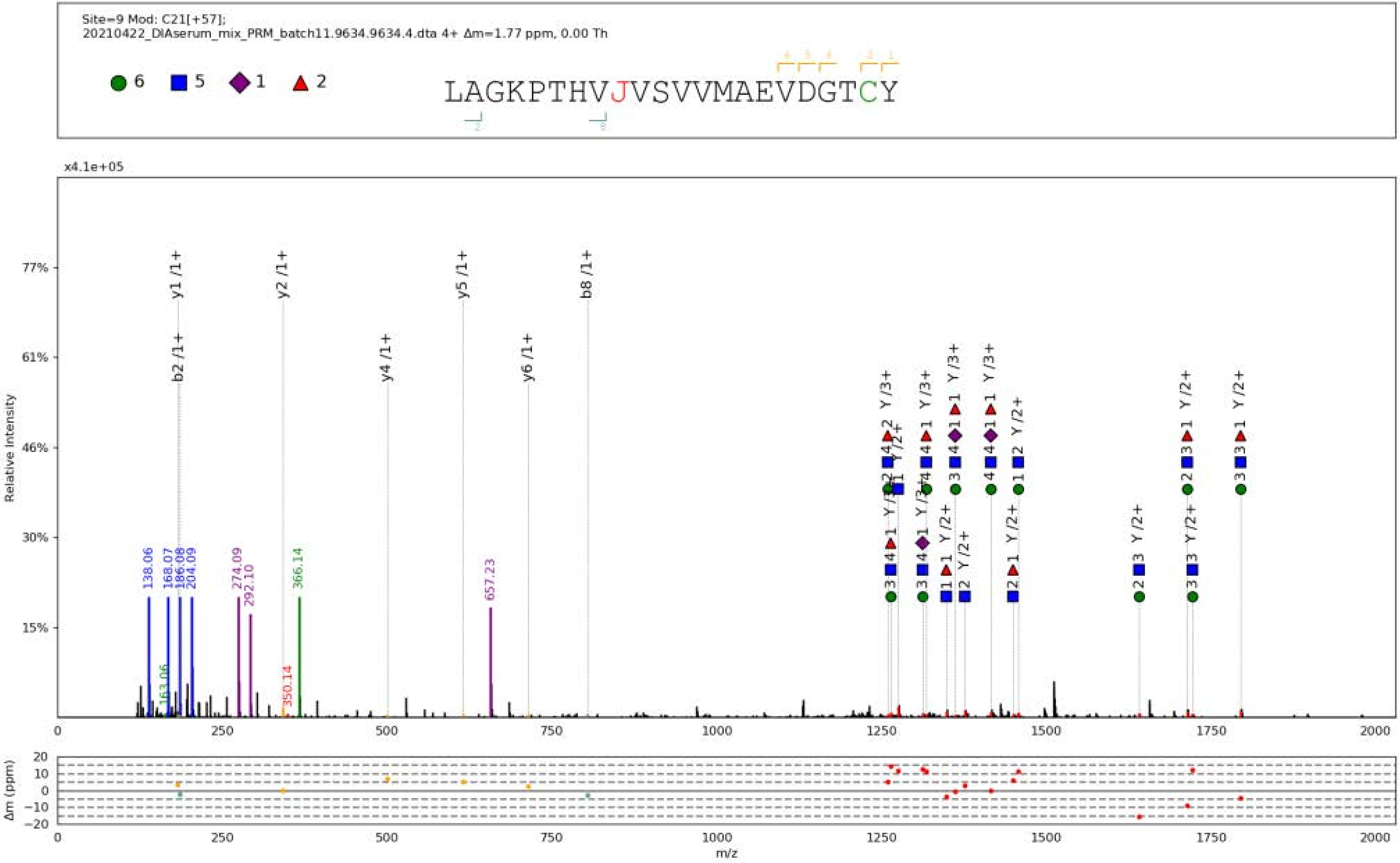

Site=9 Mod: C21[+57];
20210422_DIAserum_mix_PRM_batch11.9634.9634.4.dta 4+ Δm=1.77 ppm, 0.00 Th
6 5 1 2
LAGKPTHVJVSVVMAEVDGTCY
x4.1e+05
Relative Intensity
y1 /1+
b2 /1+
y2 /1+
y4 /1+
y5 /1+
y6 /1+
b8 /1+
138.06
168.07
186.08
204.09
163.06
274.09
292.10
350.14
366.14
657.23
m/z
Δm (ppm)

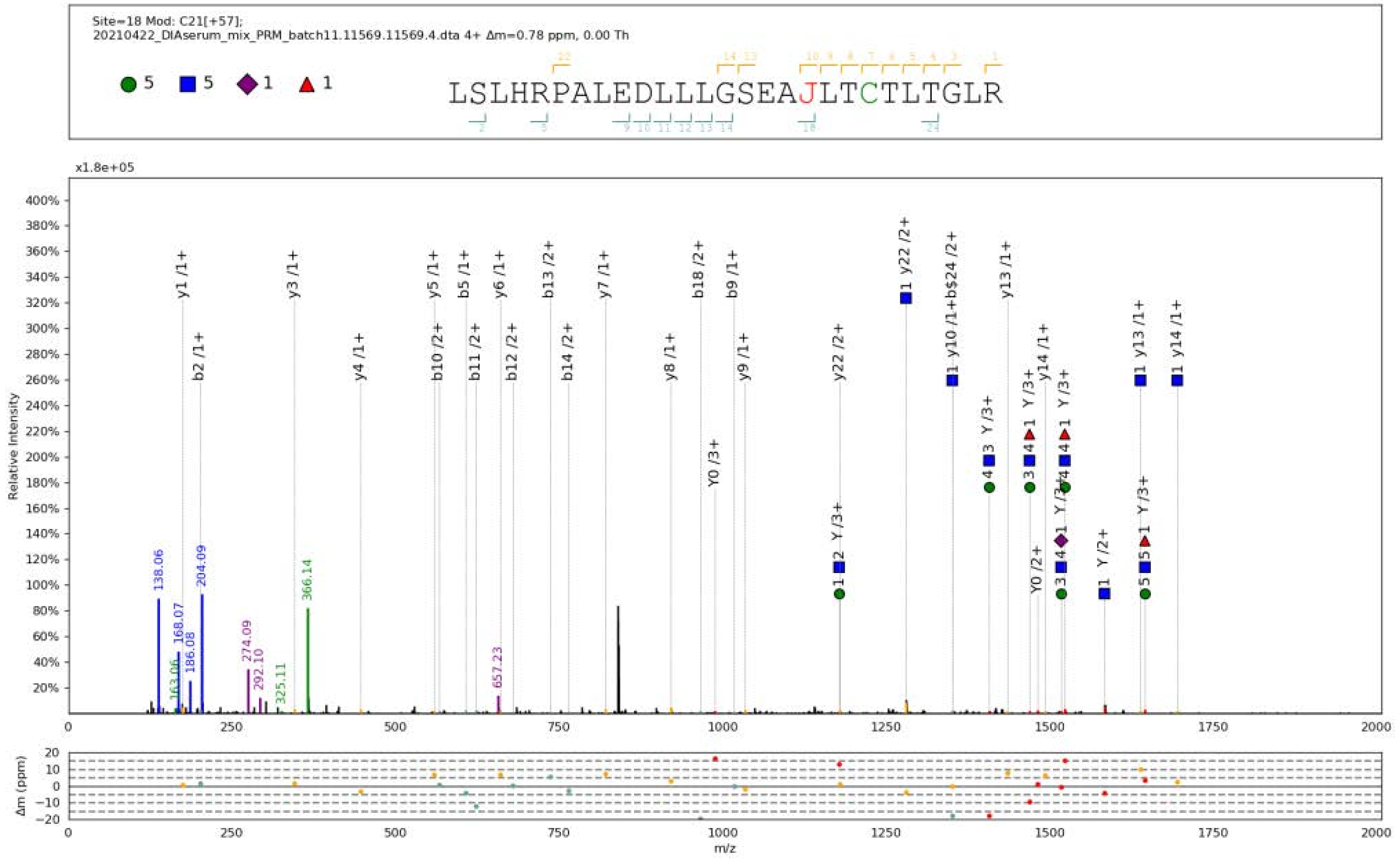
Site=18 Mod: C21[+57];
20210422_DIAserum_mix_PRM_batch11.11569.11569.4.dta 4+ Δm=0.78 ppm, 0.00 Th
LSLHRPALEDLLLGSEAJLTCTLTGLR
x1.8e+05
Relative Intensity
m/z
Δm (ppm)

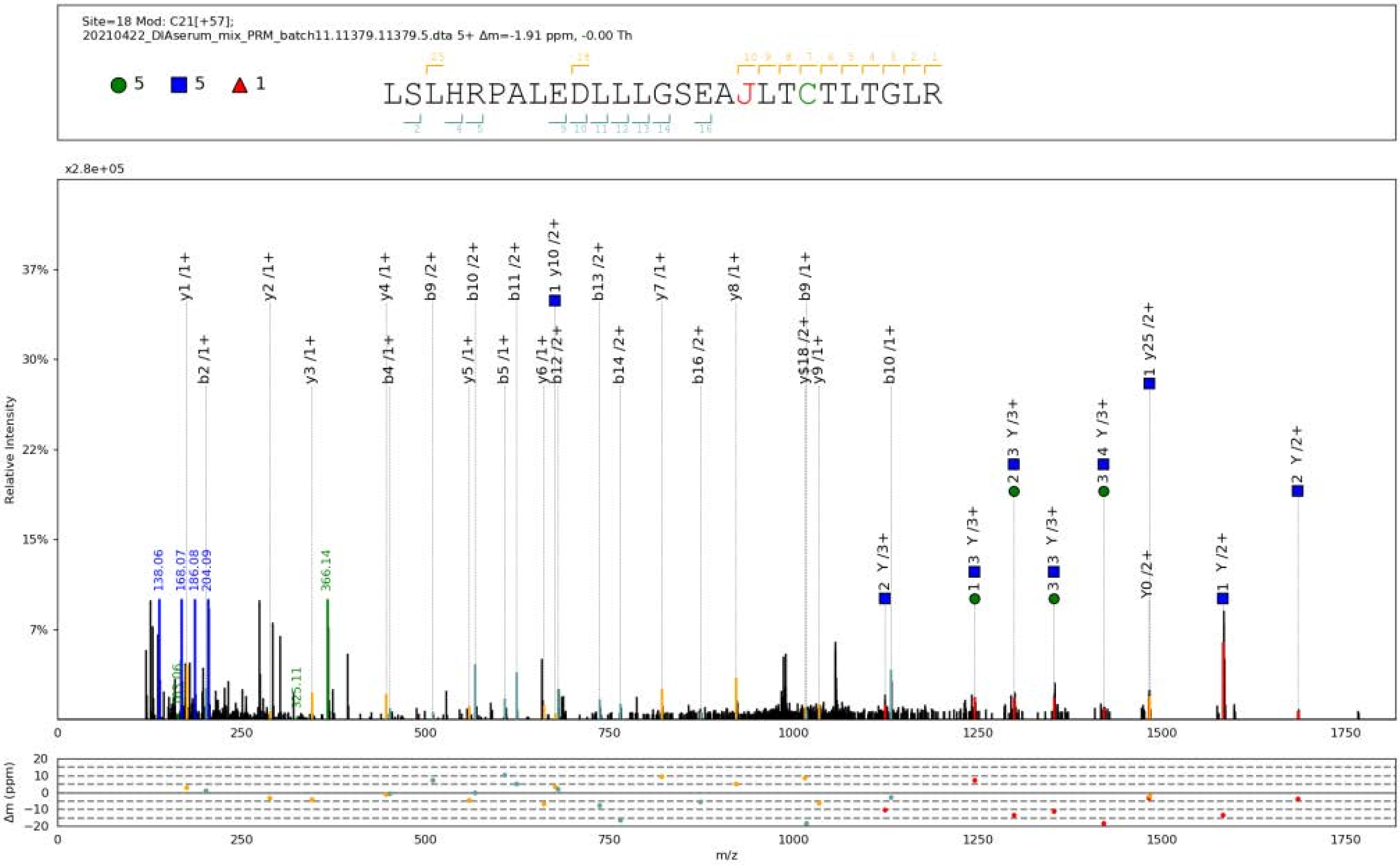

Site=18 Mod: C21[+57];
20210422_DIAserum_mix_PRM_batch11.11379.11379.5.dta 5+ Δm=-1.91 ppm, -0.00 Th
5
5
1
LSLHRPALEDLLLGSEAJLTCTLTGLR
x2.8e+05
Relative Intensity
37%
30%
22%
15%
7%
y1 /1+
b2 /1+
y2 /1+
y3 /1+
y4 /1+
b4 /1+
b9 /2+
y5 /1+
b10 /2+
b5 /1+
b11 /2+
y6 /1+
b12 /2+
1 y10 /2+
b13 /2+
b14 /2+
y7 /1+
b16 /2+
y8 /1+
y$18 /2+
b9 /1+
y9 /1+
b10 /1+
2 Y /3+
1 3 Y /3+
2 3 Y /3+
3 3 Y /3+
3 4 Y /3+
Y0 /2+
1 y25 /2+
1 Y /2+
2 Y /2+
138.06
168.07
186.08
204.09
366.14
m/z
Δm (ppm)

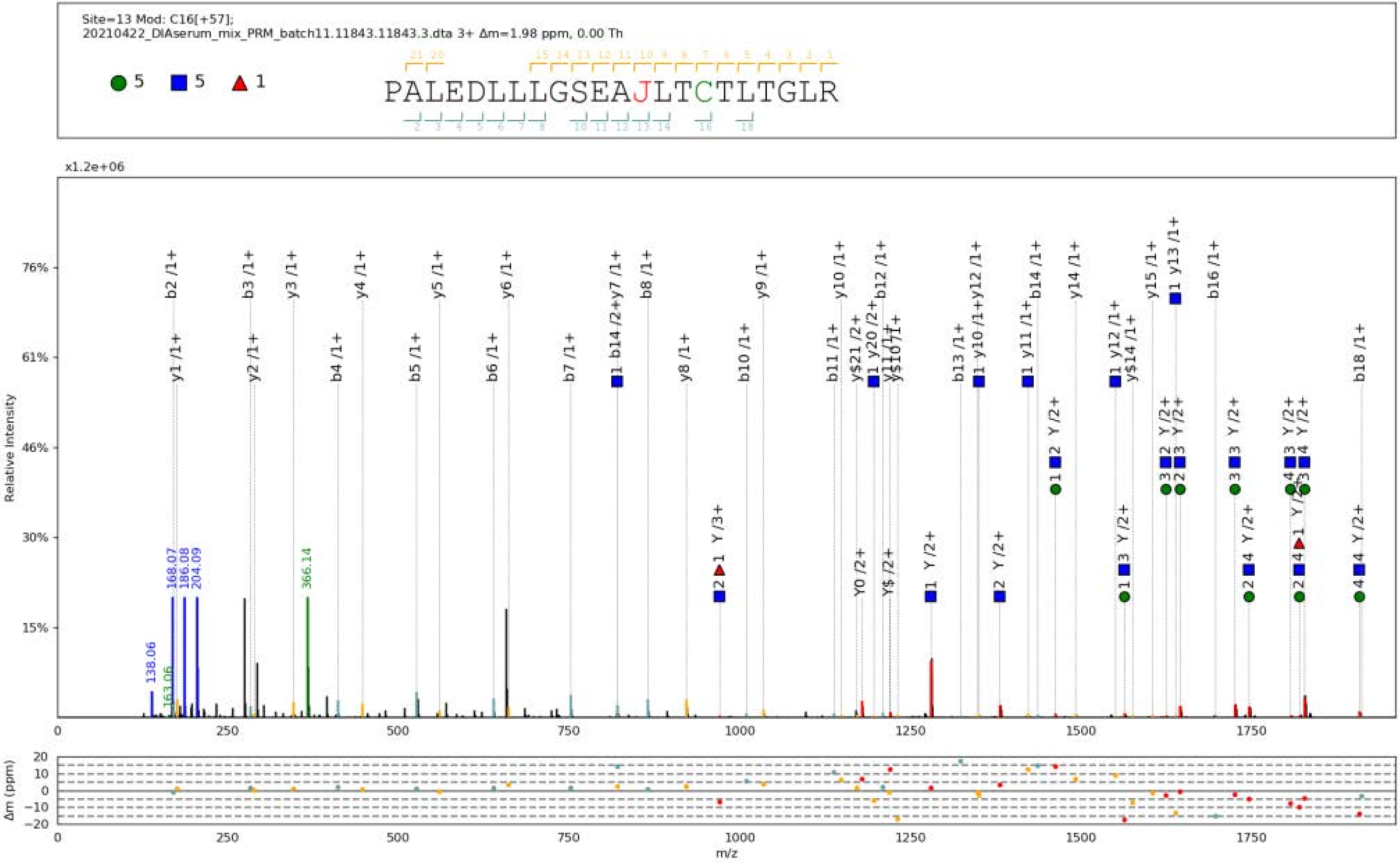

Site=13 Mod: C16[+57];
20210422_DIAserum_mix_PRM_batch11.11843.11843.3.dta 3+ Δm=1.98 ppm, 0.00 Th
5
5
1
PALEDLLLGSEAJLTCTLTGLR
x1.2e+06
Relative Intensity
76%
61%
46%
30%
15%
b2 /1+
y1 /1+
b3 /1+
y2 /1+
y3 /1+
b4 /1+
y4 /1+
b5 /1+
y5 /1+
b6 /1+
y6 /1+
b7 /1+
b14 /2+y7 /1+
b8 /1+
y8 /1+
b10 /1+
y9 /1+
b11 /1+
y10 /1+
y$21 /2+
y20 /2+
b12 /1+
b13 /1+
y10 /1+y12 /1+
y11 /1+
b14 /1+
y14 /1+
y12 /1+
y$14 /1+
y15 /1+
y13 /1+
b16 /1+
b18 /1+
Y0 /2+
Y$ /2+
138.06
163.06
168.07
186.08
204.09
366.14
0
250
500
750
1000
1250
1500
1750
Δm (ppm)
20
10
0
−10
−20
m/z

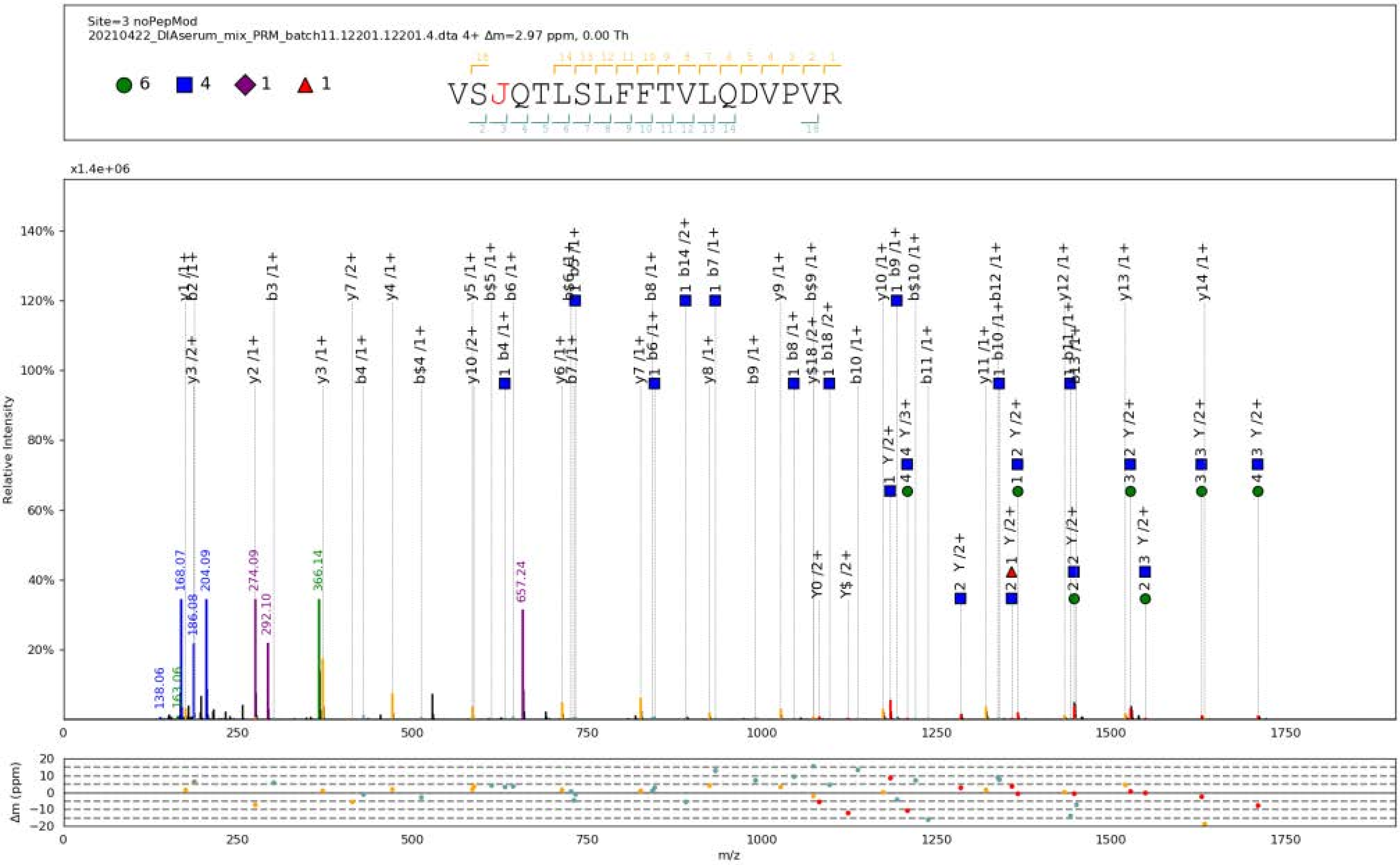
Site=3 noPepMod
20210422_DIAserum_mix_PRM_batch11.12201.12201.4.dta 4+ Δm=2.97 ppm, 0.00 Th
6
4
1
1
VSJQTLSLFFTVLQDVPVR
x1.4e+06
Relative Intensity
140%
120%
100%
80%
60%
40%
20%
138.06
163.06
168.07
186.08
204.09
274.09
292.10
366.14
657.24
y1 /1+
b2 /1+
y3 /2+
y2 /1+
b3 /1+
y3 /1+
y7 /2+
b4 /1+
y4 /1+
b$4 /1+
y5 /1+
y10 /2+
b$5 /1+
b6 /1+
y6 /1+
b7 /1+
y7 /1+
b8 /1+
b14 /2+
b7 /1+
y8 /1+
b9 /1+
y9 /1+
b8 /1+
b$9 /1+
y$18 /2+
b18 /2+
b10 /1+
Y0 /2+
Y$ /2+
y10 /1+
b9 /1+
b$10 /1+
b11 /1+
y11 /1+
b10 /1+
b12 /1+
y12 /1+
b11 /1+
b13 /1+
y13 /1+
y14 /1+
Y /2+
Y /3+
0
250
500
750
1000
1250
1500
1750
Δm (ppm)
20
10
0
-10
-20
m/z

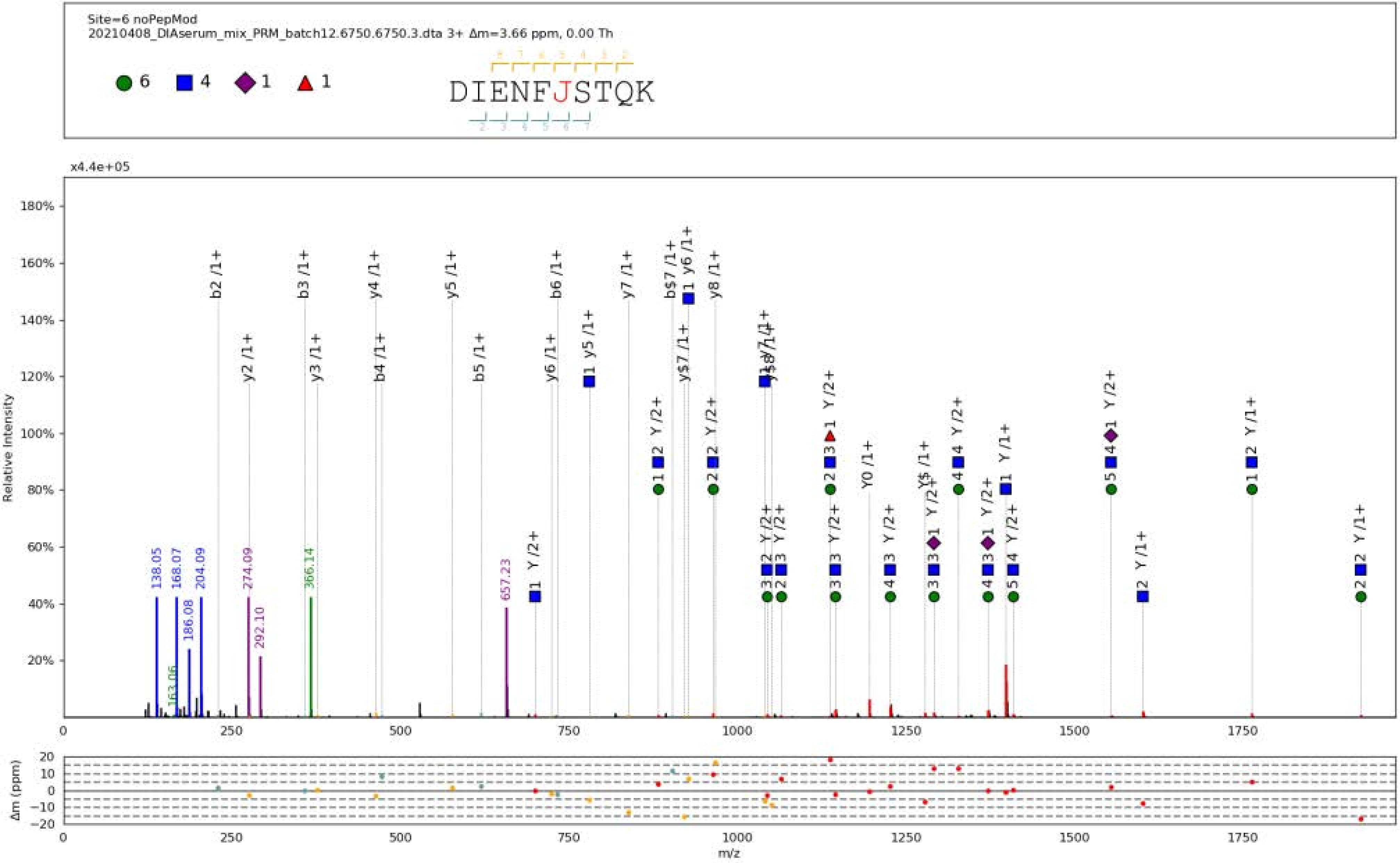

Site=6 noPepMod
20210408_DIAserum_mix_PRM_batch12.6750.6750.3.dta 3+ Δm=3.66 ppm, 0.00 Th
6
4
1
1
DIENFJSTQK
x4.4e+05
Relative Intensity
180%
160%
140%
120%
100%
80%
60%
40%
20%
b2 /1+
b3 /1+
y4 /1+
y5 /1+
b6 /1+
y7 /1+
b$7 /1+
y6 /1+
y8 /1+
y2 /1+
y3 /1+
b4 /1+
b5 /1+
y6 /1+
y5 /1+
y$7 /1+
Y0 /1+
138.05
168.07
186.08
204.09
163.06
274.09
292.10
366.14
657.23
m/z
Δm (ppm)
0
250
500
750
1000
1250
1500
1750

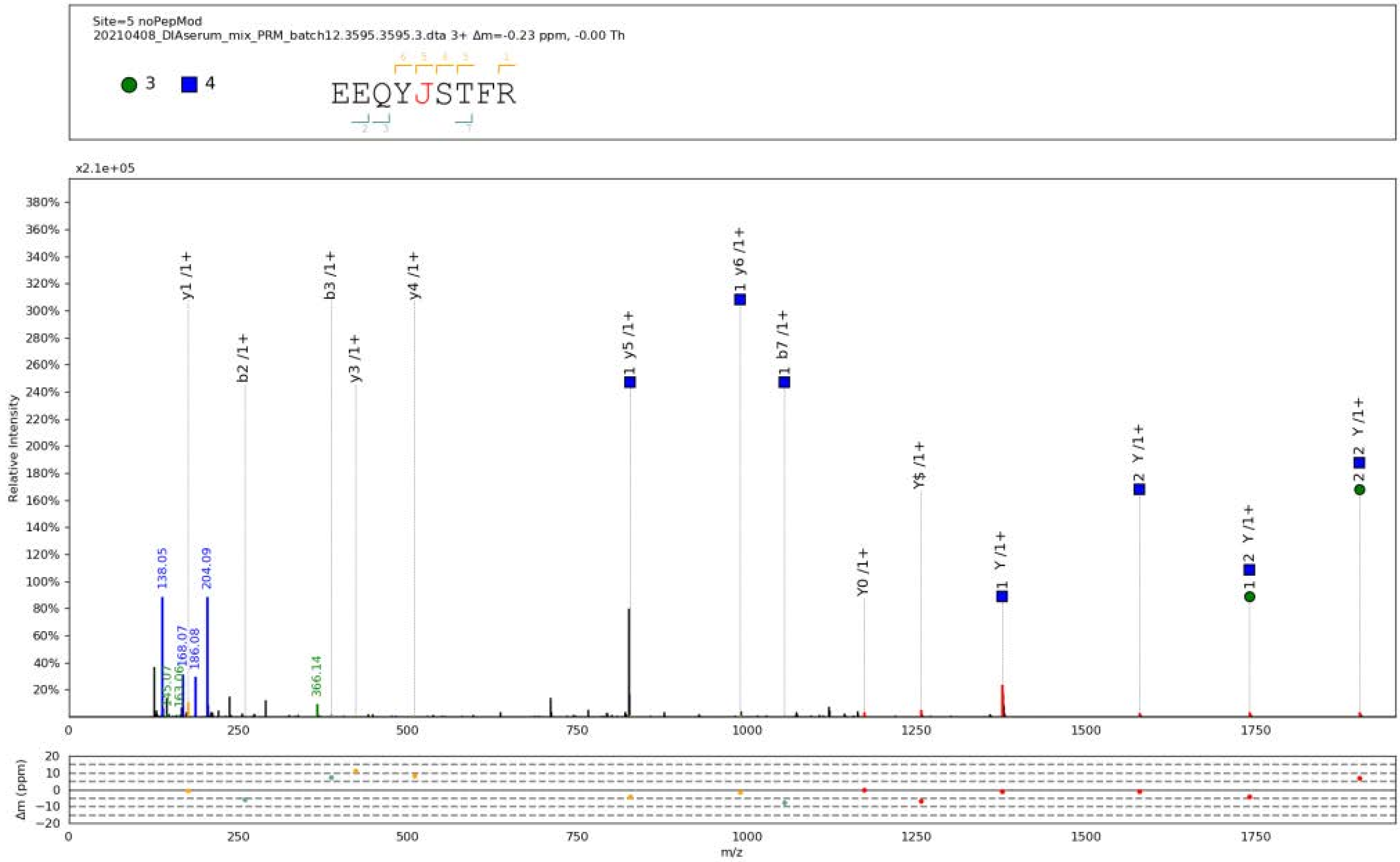

Site=5 noPepMod
20210408_DIAserum_mix_PRM_batch12.3595.3595.3.dta 3+ Δm=-0.23 ppm, -0.00 Th
3
4
EEQYJSTFR
x2.1e+05
Relative Intensity
y1 /1+
b2 /1+
b3 /1+
y3 /1+
y4 /1+
1 y5 /1+
1 y6 /1+
1 b7 /1+
Y0 /1+
Y$ /1+
1 Y /1+
2 Y /1+
1 2 Y /1+
2 2 Y /1+
138.05
204.09
168.07
186.08
366.14
m/z
Δm (ppm)

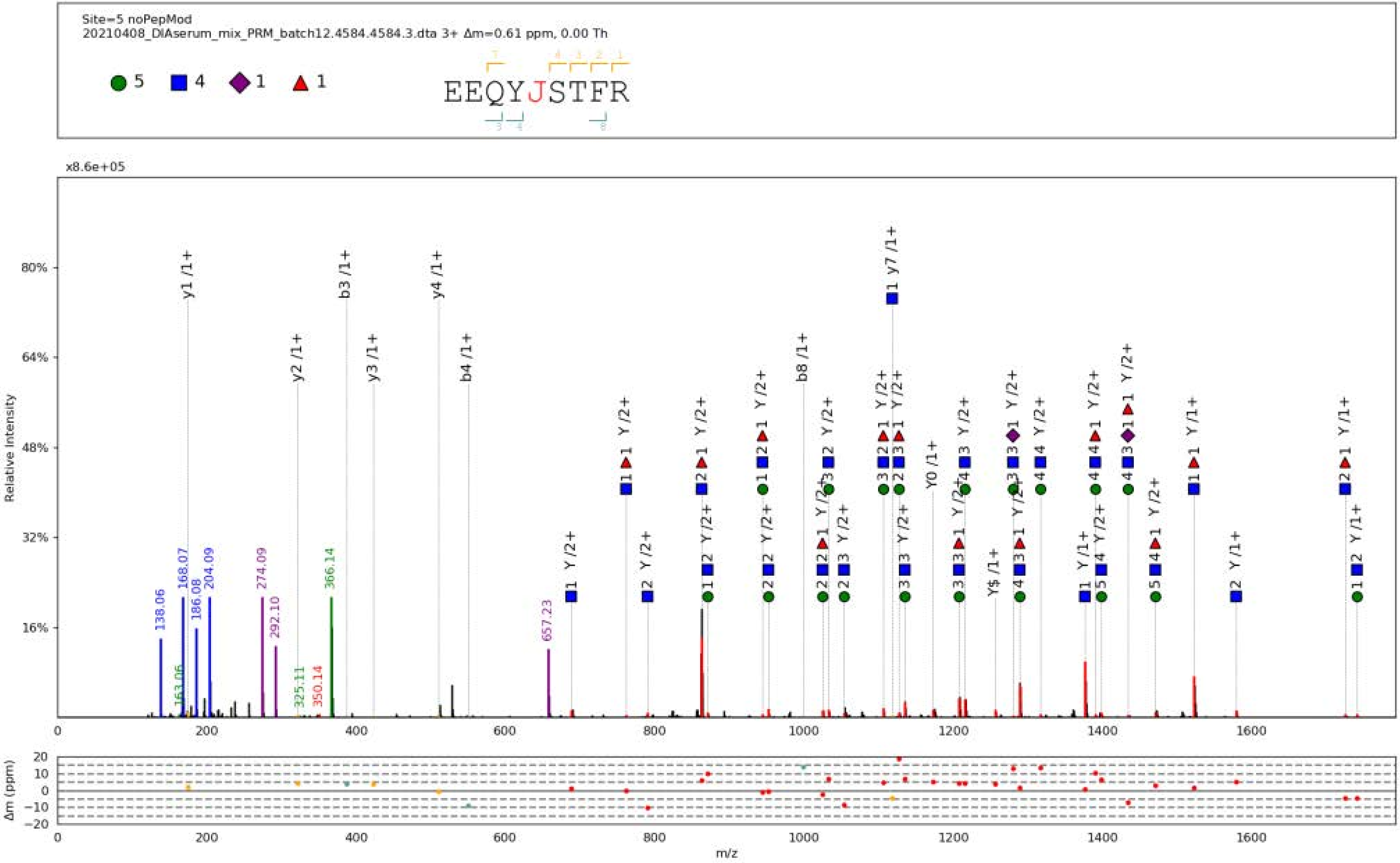
Site=5 noPepMod
20210408_DIAserum_mix_PRM_batch12.4584.4584.3.dta 3+ Δm=0.61 ppm, 0.00 Th
5 4 1 1
EEQYJSTFR
x8.6e+05
Relative Intensity
m/z
Δm (ppm)
y1 /1+
b3 /1+
y4 /1+
y2 /1+
y3 /1+
b4 /1+
b8 /1+
1 y7 /1+
Y0 /1+
Y$ /1+
138.06
168.07
186.08
204.09
274.09
292.10
366.14
163.06
325.11
350.14
657.23

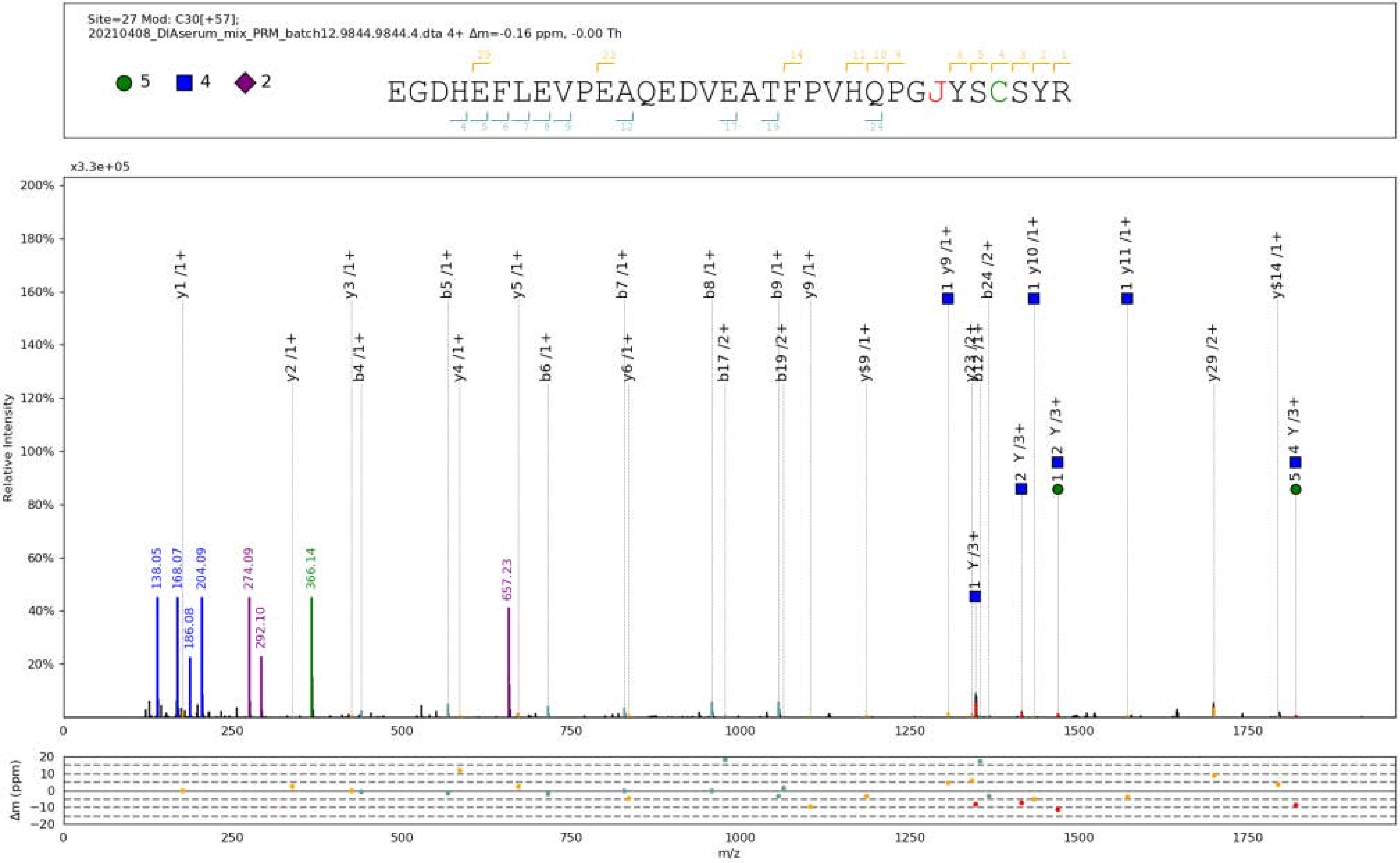
Site=27 Mod: C30[+57];
20210408_DIAserum_mix_PRM_batch12.9844.9844.4.dta 4+ Δm=-0.16 ppm, -0.00 Th
EGDHEFLEVPEAQEDVEATFPVHQPGJYSCSYR
x3.3e+05
Relative Intensity
m/z
Δm (ppm)

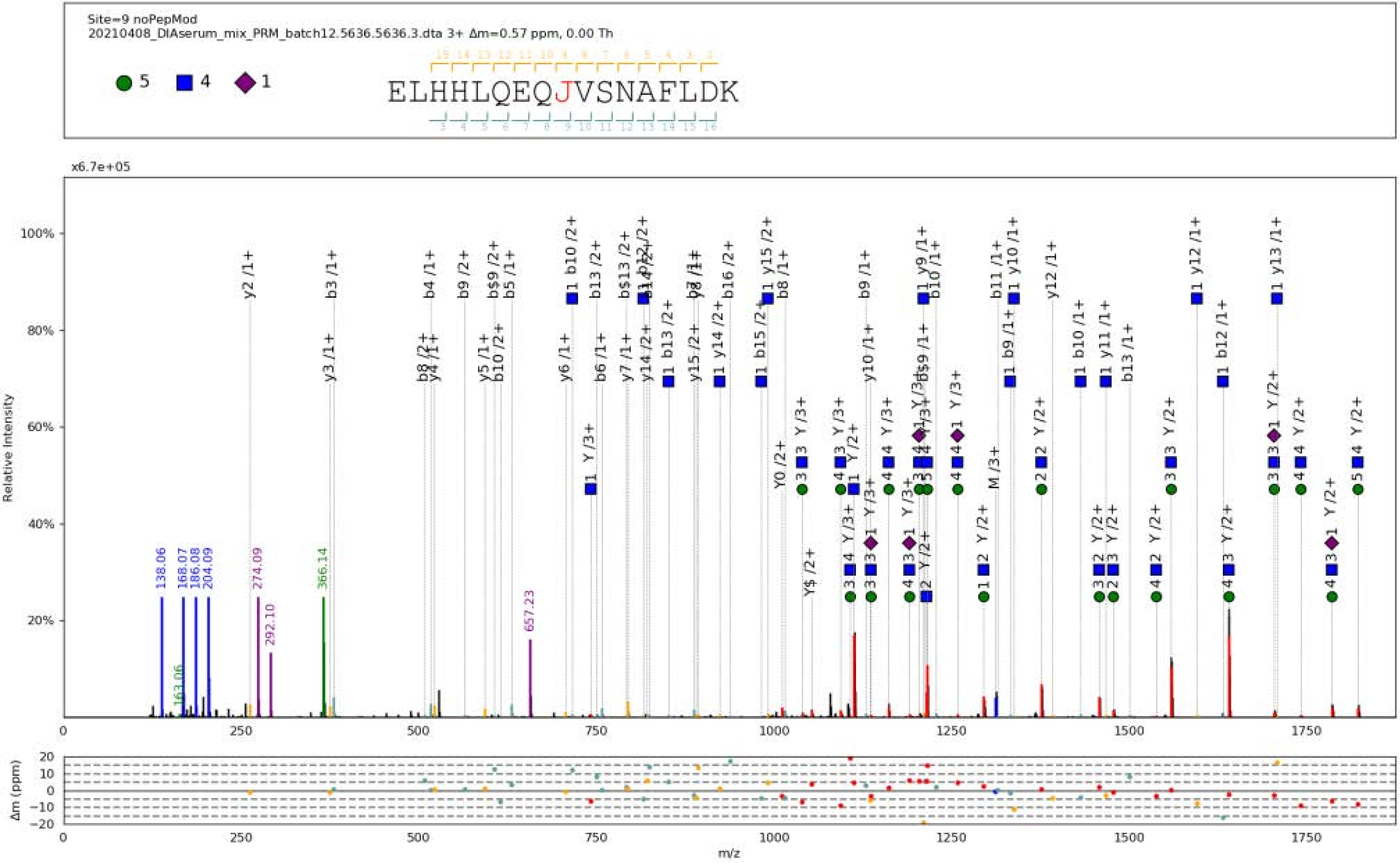
Site=9 noPepMod
20210408_DIAserum_mix_PRM_batch12.5636.5636.3.dta 3+ Δm=0.57 ppm, 0.00 Th
5
4
1
ELHHLQEQJVSNAFLDK
x6.7e+05
Relative Intensity
m/z
Δm (ppm)

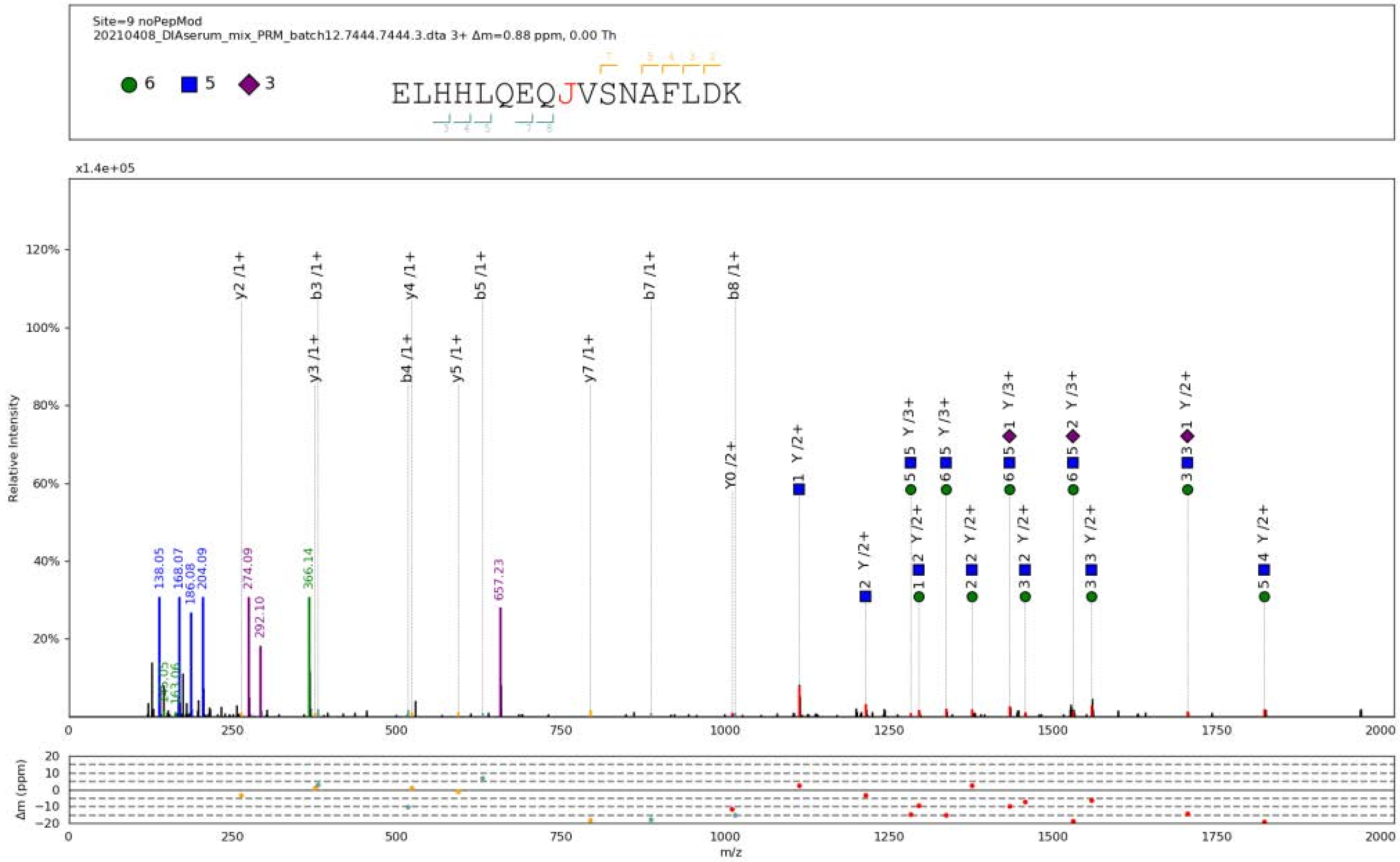

Site=9 noPepMod
20210408_DIAserum_mix_PRM_batch12.7444.7444.3.dta 3+ Δm=0.88 ppm, 0.00 Th
6
5
3
ELHHLQEQJVSNAFLDK
x1.4e+05
Relative Intensity
m/z
Δm (ppm)

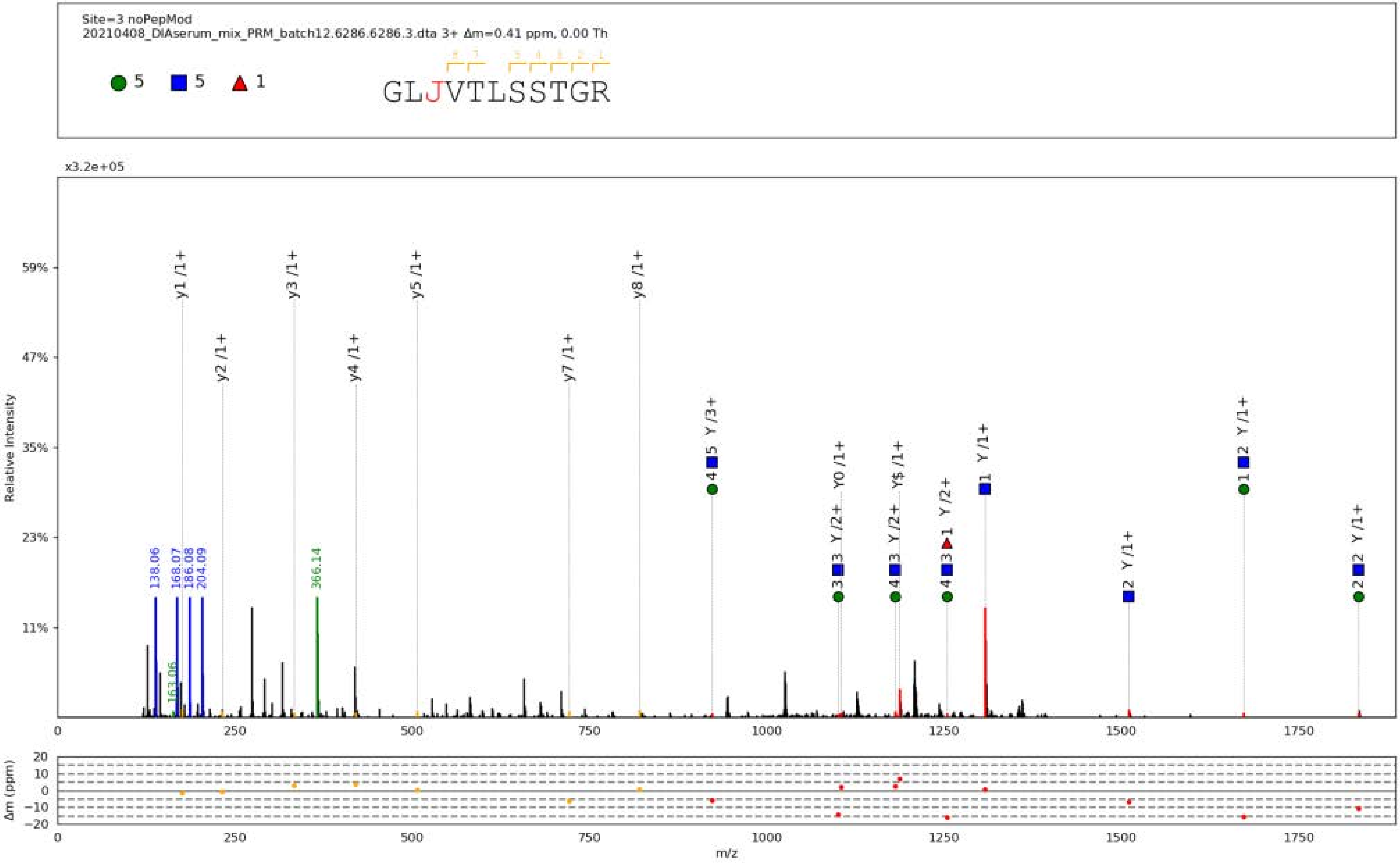
Site=3 noPepMod
20210408_DIAserum_mix_PRM_batch12.6286.6286.3.dta 3+ Δm=0.41 ppm, 0.00 Th
5 5 1
GLJVTLSSTGR
x3.2e+05
Relative Intensity
59%
47%
35%
23%
11%
y1 /1+
y2 /1+
y3 /1+
y4 /1+
y5 /1+
y7 /1+
y8 /1+
4 5 Y /3+
3 3 Y /2+ Y0 /1+
4 3 Y /2+ Y$ /1+
4 3 1 Y /2+
1 Y /1+
2 Y /1+
1 2 Y /1+
2 2 Y /1+
138.06
168.07
186.08
204.09
163.06
366.14
0
250
500
750
1000
1250
1500
1750
Δm (ppm)
20
10
0
−10
−20
m/z

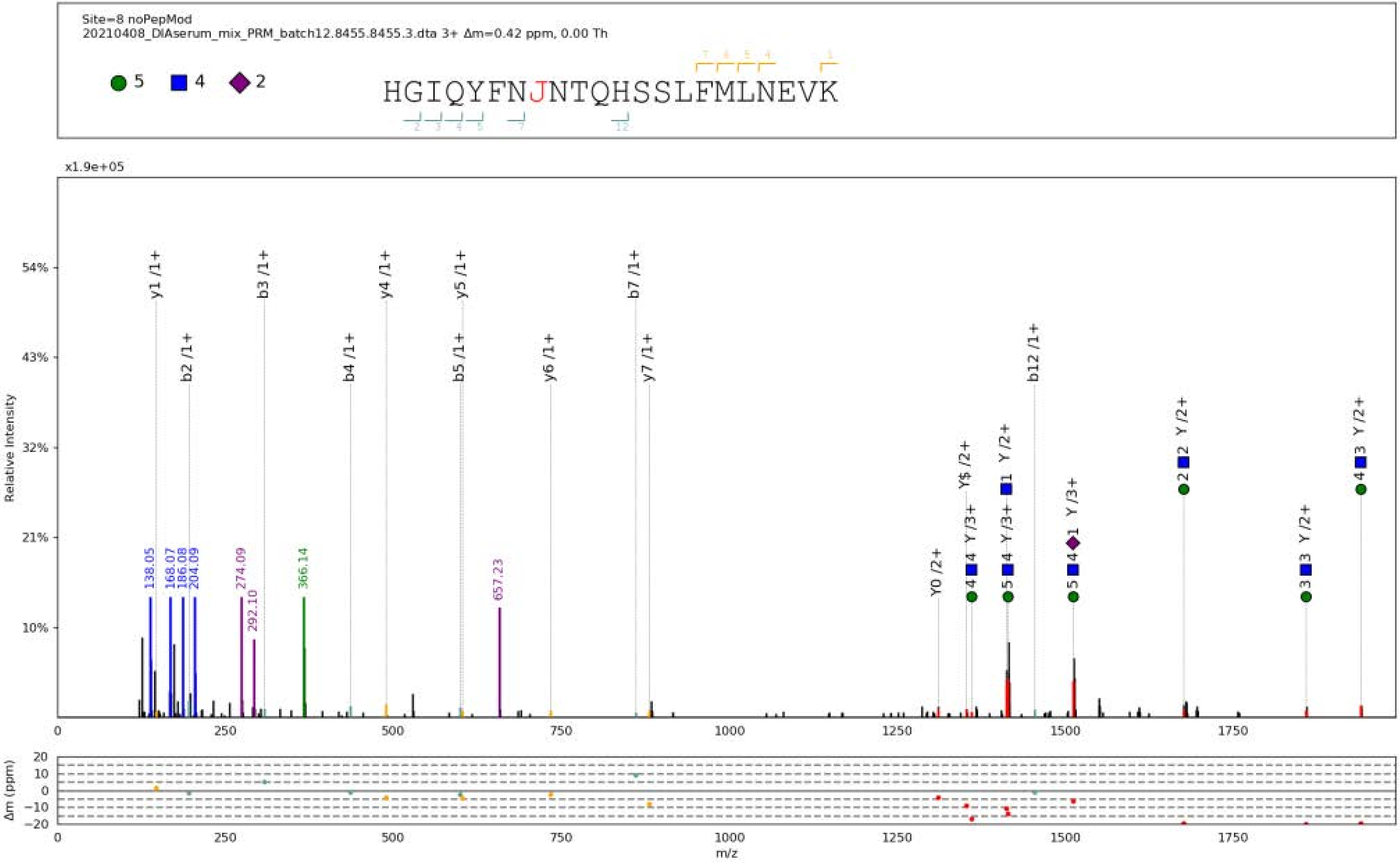

Site=8 noPepMod
20210408_DIAserum_mix_PRM_batch12.8455.8455.3.dta 3+ Δm=0.42 ppm, 0.00 Th
5
4
2
HGIQYFNJNTQHSSLFMLNEVK
x1.9e+05
Relative Intensity
y1 /1+
b2 /1+
b3 /1+
b4 /1+
y4 /1+
y5 /1+
b5 /1+
y6 /1+
b7 /1+
y7 /1+
b12 /1+
Y0 /2+
Y$ /2+
138.05
168.07
186.08
204.09
274.09
292.10
366.14
657.23
m/z
Δm (ppm)

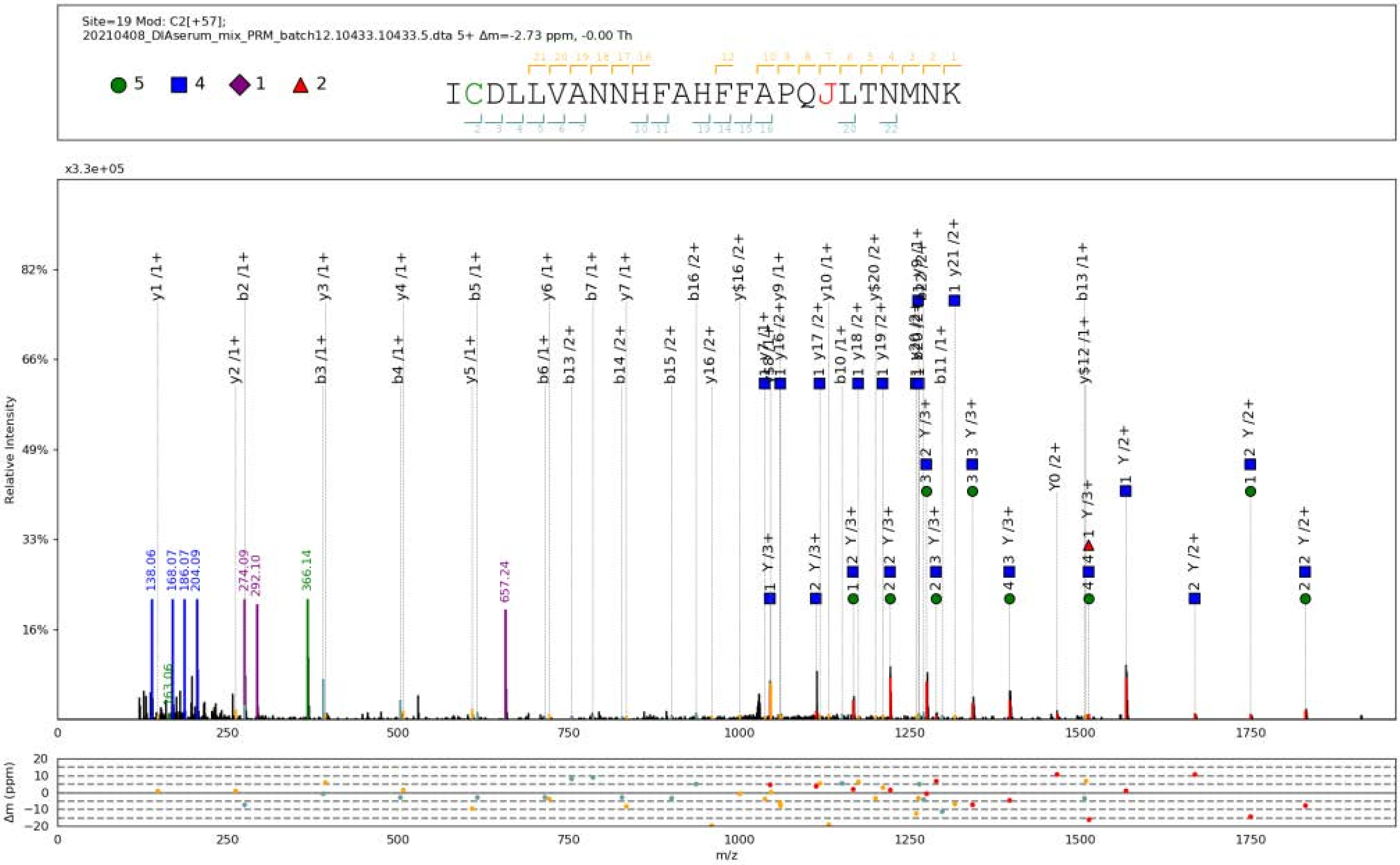
Site=19 Mod: C2[+57];
20210408_DIAserum_mix_PRM_batch12.10433.10433.5.dta 5+ Δm=-2.73 ppm, -0.00 Th
ICDLLVANNHFAHFFAPQJLTNMNK
x3.3e+05
Relative Intensity
Δm (ppm)
m/z

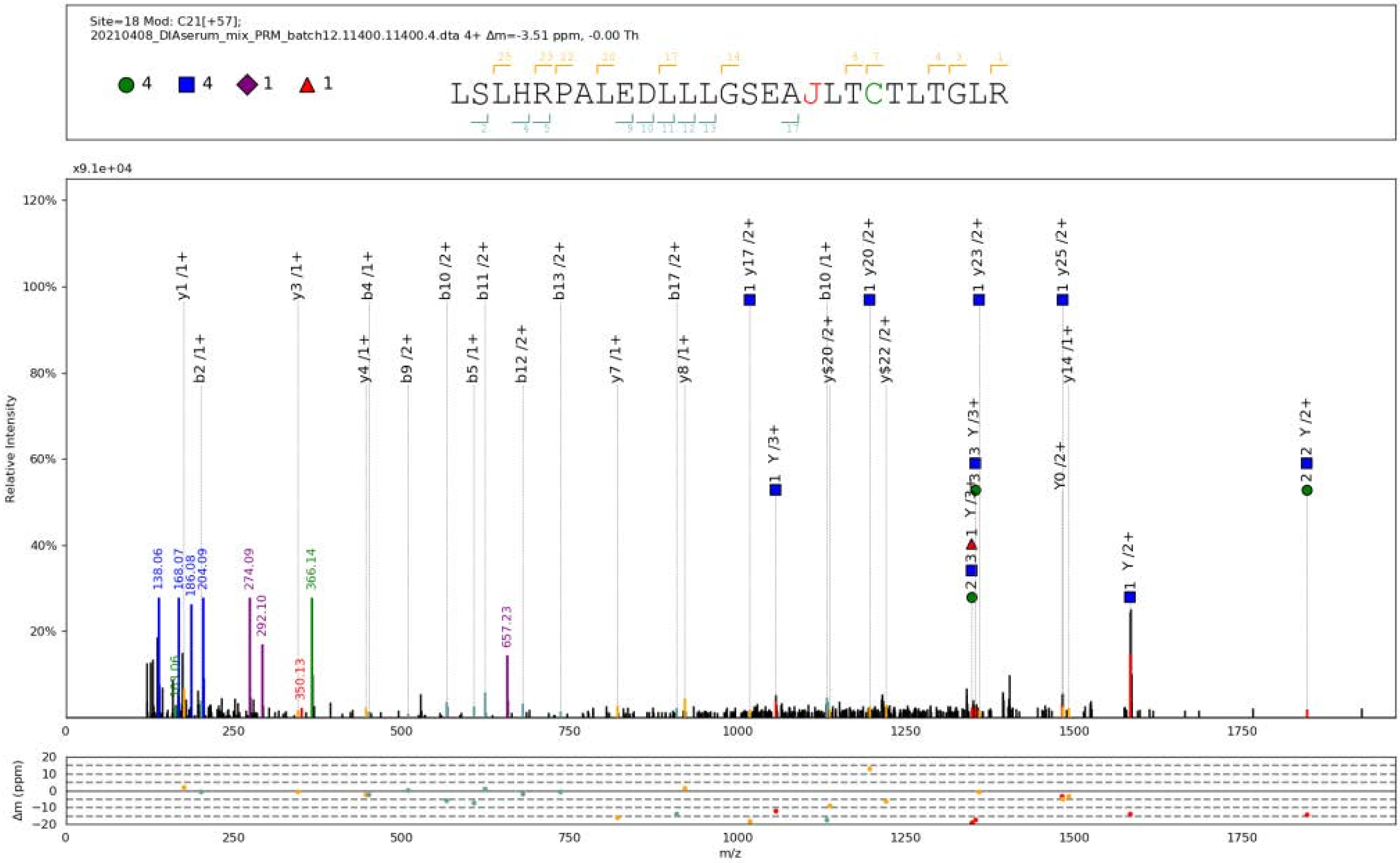
Site=18 Mod: C21[+57];
20210408_DIAserum_mix_PRM_batch12.11400.11400.4.dta 4+ Δm=-3.51 ppm, -0.00 Th
LSLHRPALEDLLLGSEAJLTCTLTGLR
x9.1e+04
Relative Intensity
Δm (ppm)
m/z

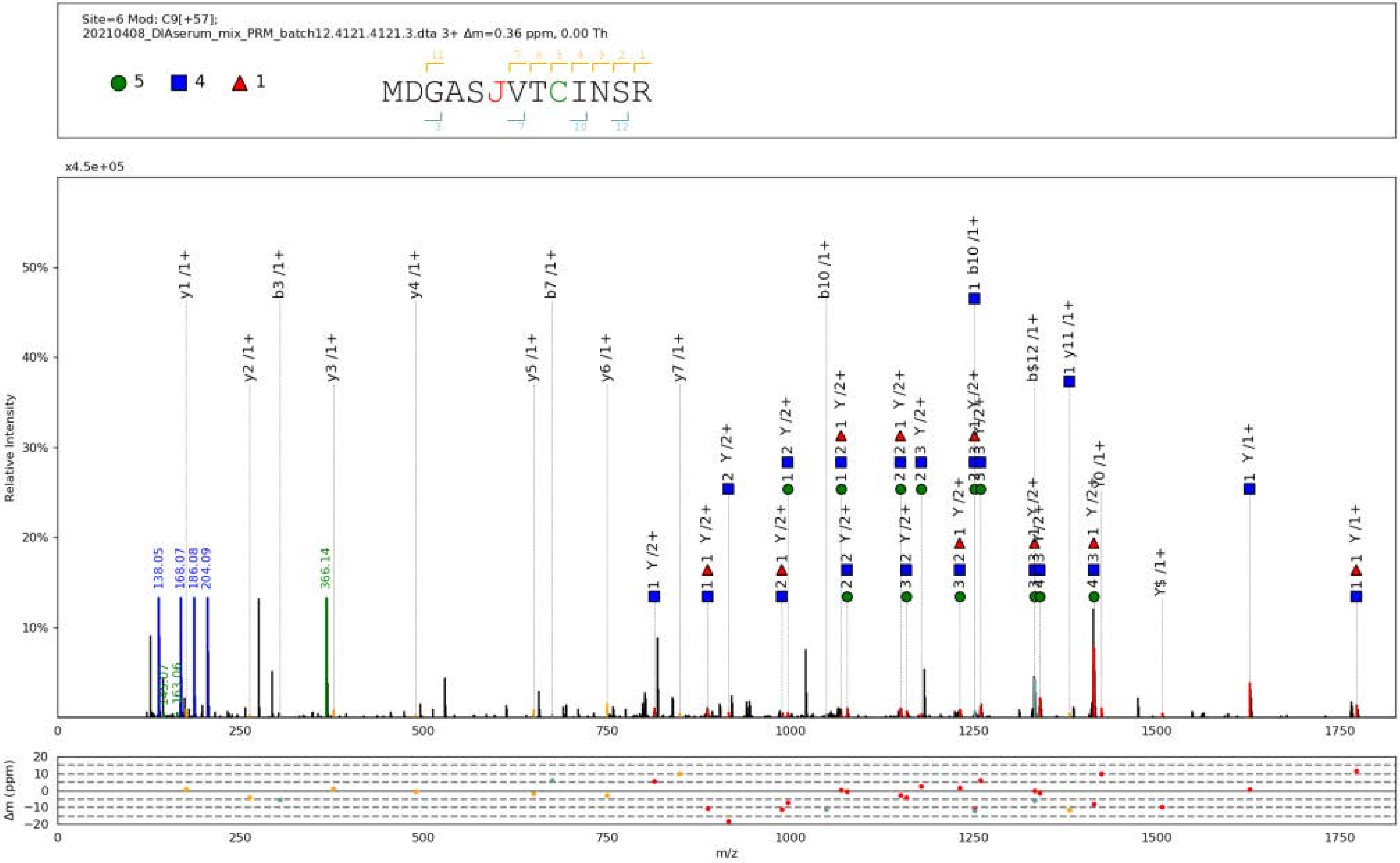

Site=6 Mod: C9[+57];
20210408_DIAserum_mix_PRM_batch12.4121.4121.3.dta 3+ Δm=0.36 ppm, 0.00 Th
5
4
1
MDGASJVTCINSR
x4.5e+05
Relative Intensity
y1 /1+
y2 /1+
b3 /1+
y3 /1+
y4 /1+
y5 /1+
b7 /1+
y6 /1+
y7 /1+
b10 /1+
1 b10 /1+
b$12 /1+
1 y11 /1+
Y$ /1+
138.05
168.07
186.08
204.09
366.14
163.06
Δm (ppm)
m/z

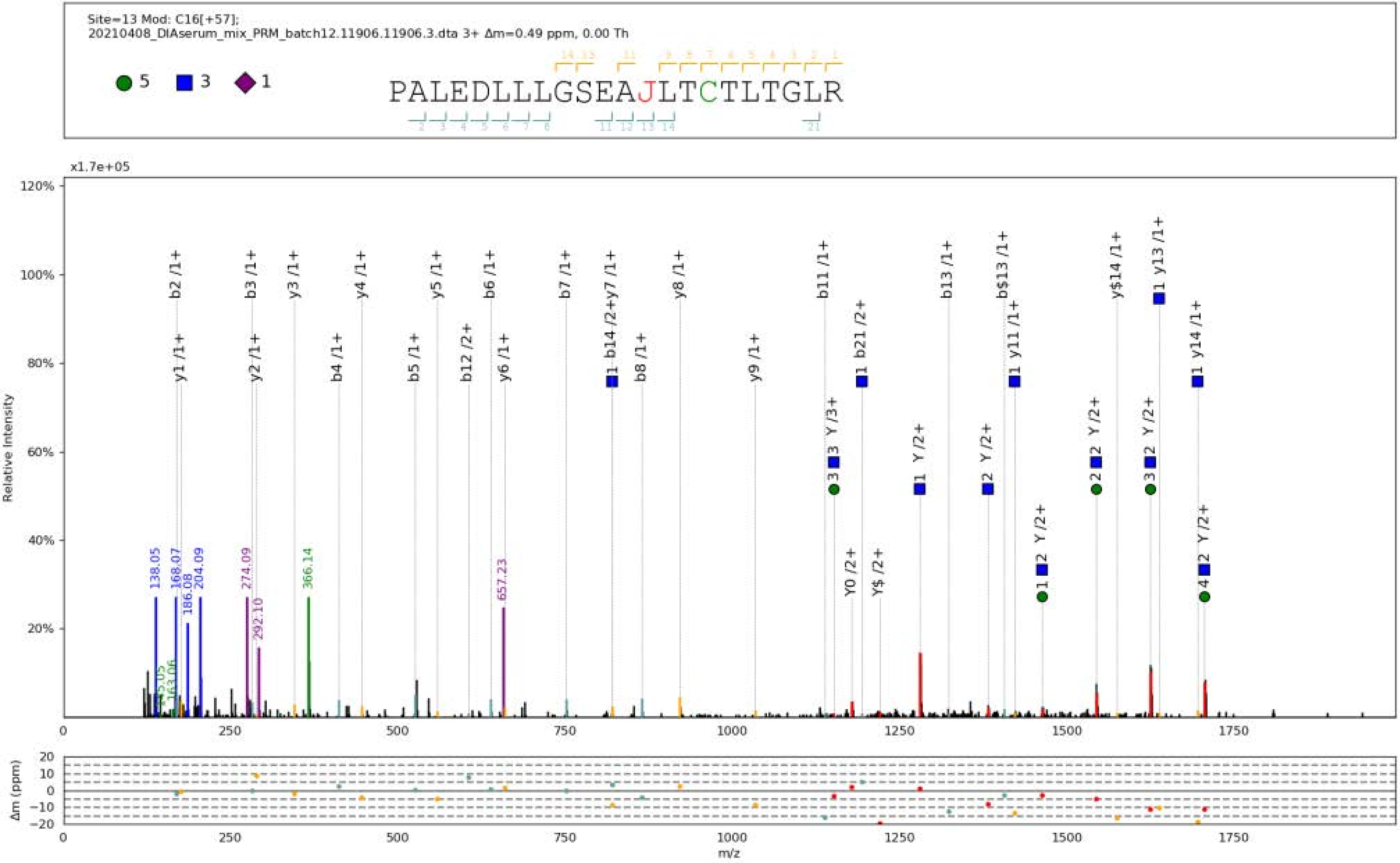
Site=13 Mod: C16[+57];
20210408_DIAserum_mix_PRM_batch12.11906.11906.3.dta 3+ Δm=0.49 ppm, 0.00 Th
5
3
1
PALEDLLLGSEAJLTCTLTGLR
x1.7e+05
Relative Intensity
b2 /1+
y1 /1+
b3 /1+
y2 /1+
y3 /1+
b4 /1+
y4 /1+
b5 /1+
y5 /1+
b12 /2+
b6 /1+
y6 /1+
b7 /1+
1 b14 /2+y7 /1+
b8 /1+
y8 /1+
y9 /1+
b11 /1+
3 3 Y /3+
Y0 /2+
1 b21 /2+
Y$ /2+
1 Y /2+
b13 /1+
2 Y /2+
b$13 /1+
1 y11 /1+
1 2 Y /2+
2 2 Y /2+
y$14 /1+
3 2 Y /2+
1 y13 /1+
1 y14 /1+
4 2 Y /2+
138.05
168.07
186.08
204.09
274.09
292.10
366.14
657.23
Δm (ppm)
m/z

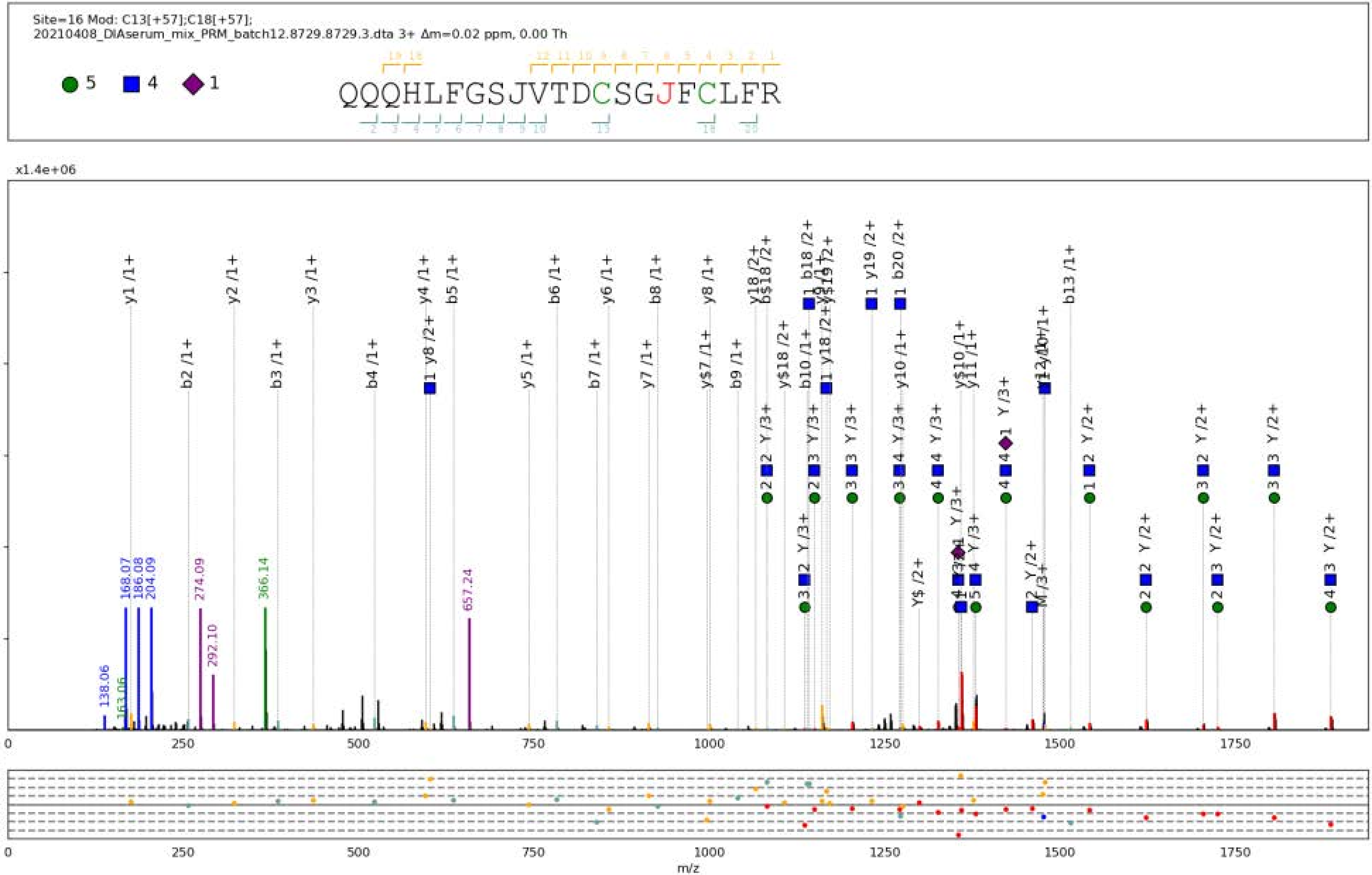

Site=16 Mod: C13[+57];C18[+57];
20210408_DIAserum_mix_PRM_batch12.8729.8729.3.dta 3+ Δm=0.02 ppm, 0.00 Th
5
4
1
QQQHLFGSJVTDCSGJFCLFR
x1.4e+06
Relative Intensity
138.06
163.06
168.07
186.08
204.09
274.09
292.10
366.14
657.24
Δm (ppm)
m/z

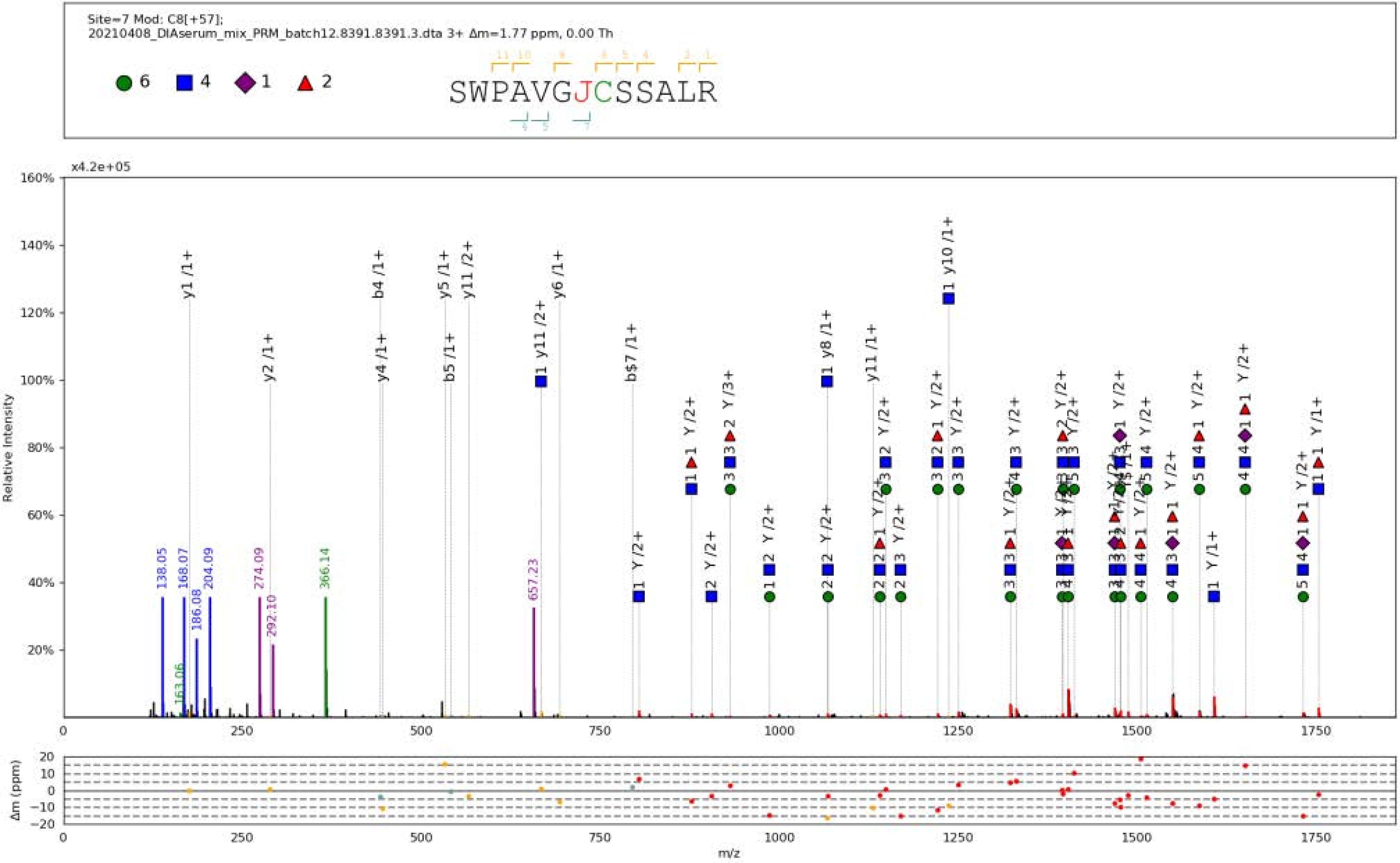

Site=7 Mod: C8[+57];
20210408_DIAserum_mix_PRM_batch12.8391.8391.3.dta 3+ Δm=1.77 ppm, 0.00 Th
SWPAVGJCSSALR
Relative Intensity
m/z
Δm (ppm)

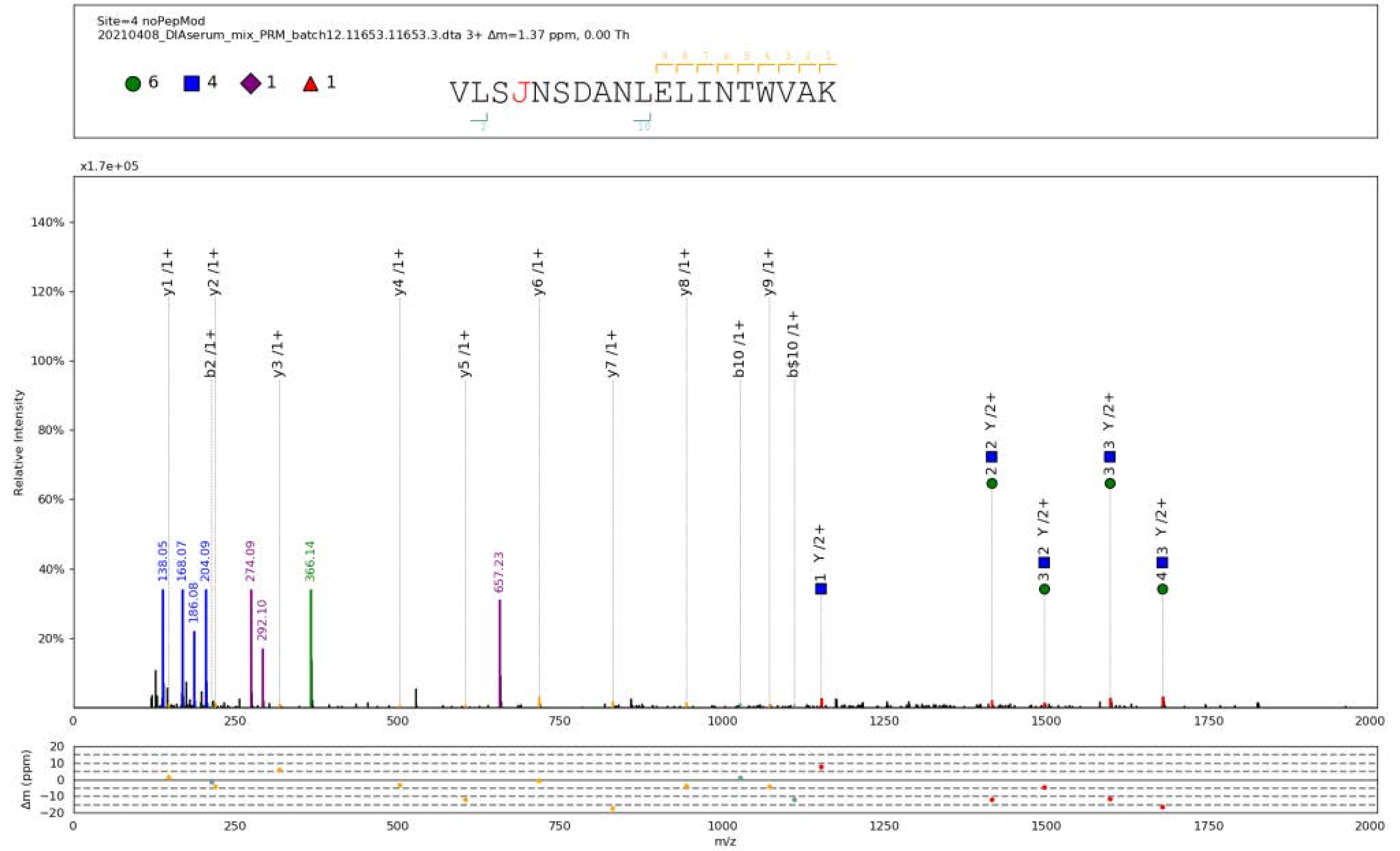

Site=4 noPepMod
20210408_DIAserum_mix_PRM_batch12.11653.11653.3.dta 3+ Δm=1.37 ppm, 0.00 Th
6
4
1
1
VLSJNSDANLELINTWVAK
x1.7e+05
Relative Intensity
y1 /1+
y2 /1+
b2 /1+
y3 /1+
y4 /1+
y5 /1+
y6 /1+
y7 /1+
y8 /1+
b10 /1+
y9 /1+
b$10 /1+
1 Y /2+
2 2 Y /2+
3 2 Y /2+
3 3 Y /2+
4 3 Y /2+
138.05
168.07
186.08
204.09
274.09
292.10
366.14
657.23
m/z
Δm (ppm)

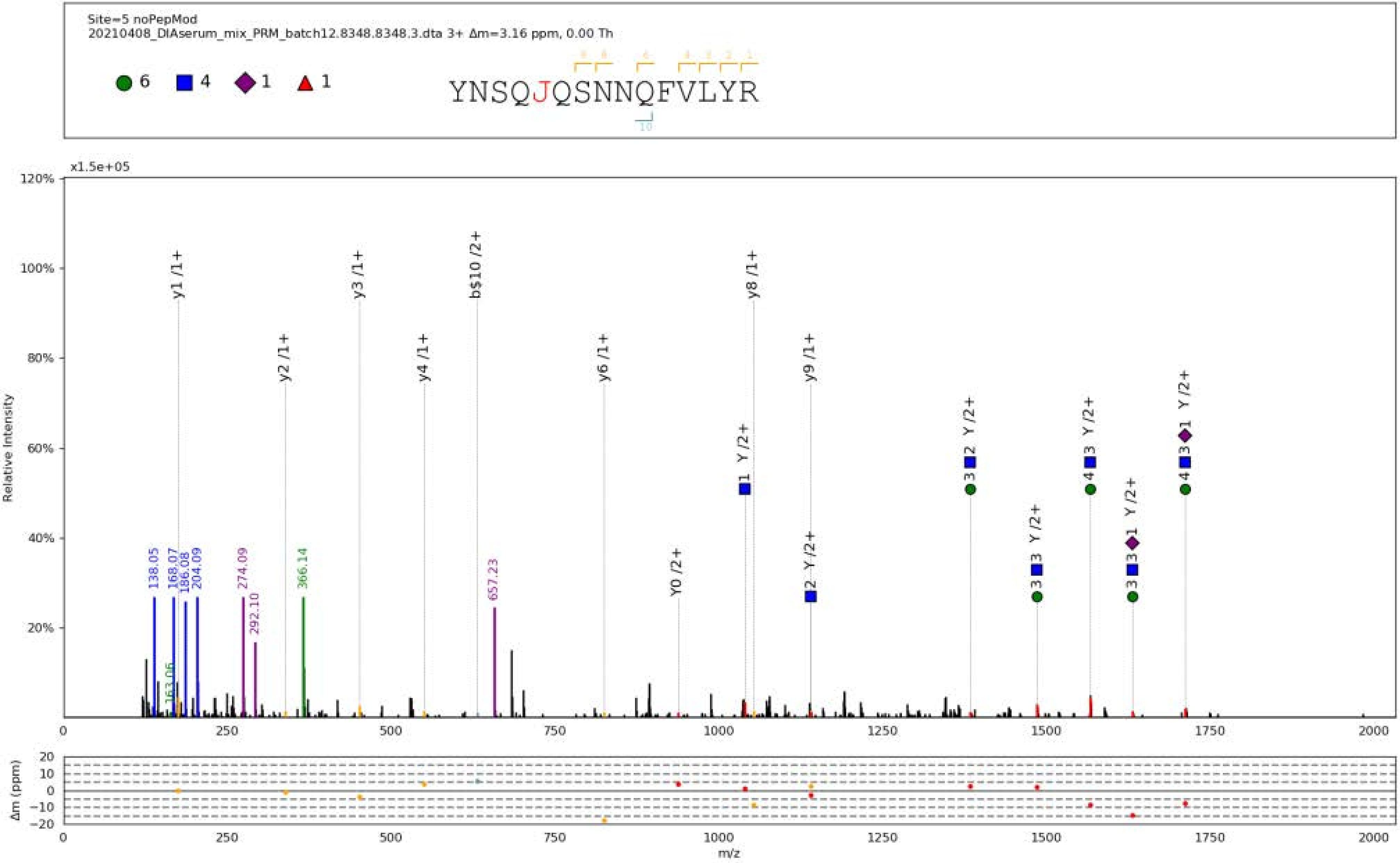

Site=5 noPepMod
20210408_DIAserum_mix_PRM_batch12.8348.8348.3.dta 3+ Δm=3.16 ppm, 0.00 Th
6
4
1
1
YNSQJQSNNQFVLYR
x1.5e+05
Relative Intensity
y1 /1+
y2 /1+
y3 /1+
y4 /1+
b$10 /2+
y6 /1+
Y0 /2+
y8 /1+
y9 /1+
138.05
168.07
186.08
204.09
163.06
274.09
292.10
366.14
657.23
m/z
Δm (ppm)

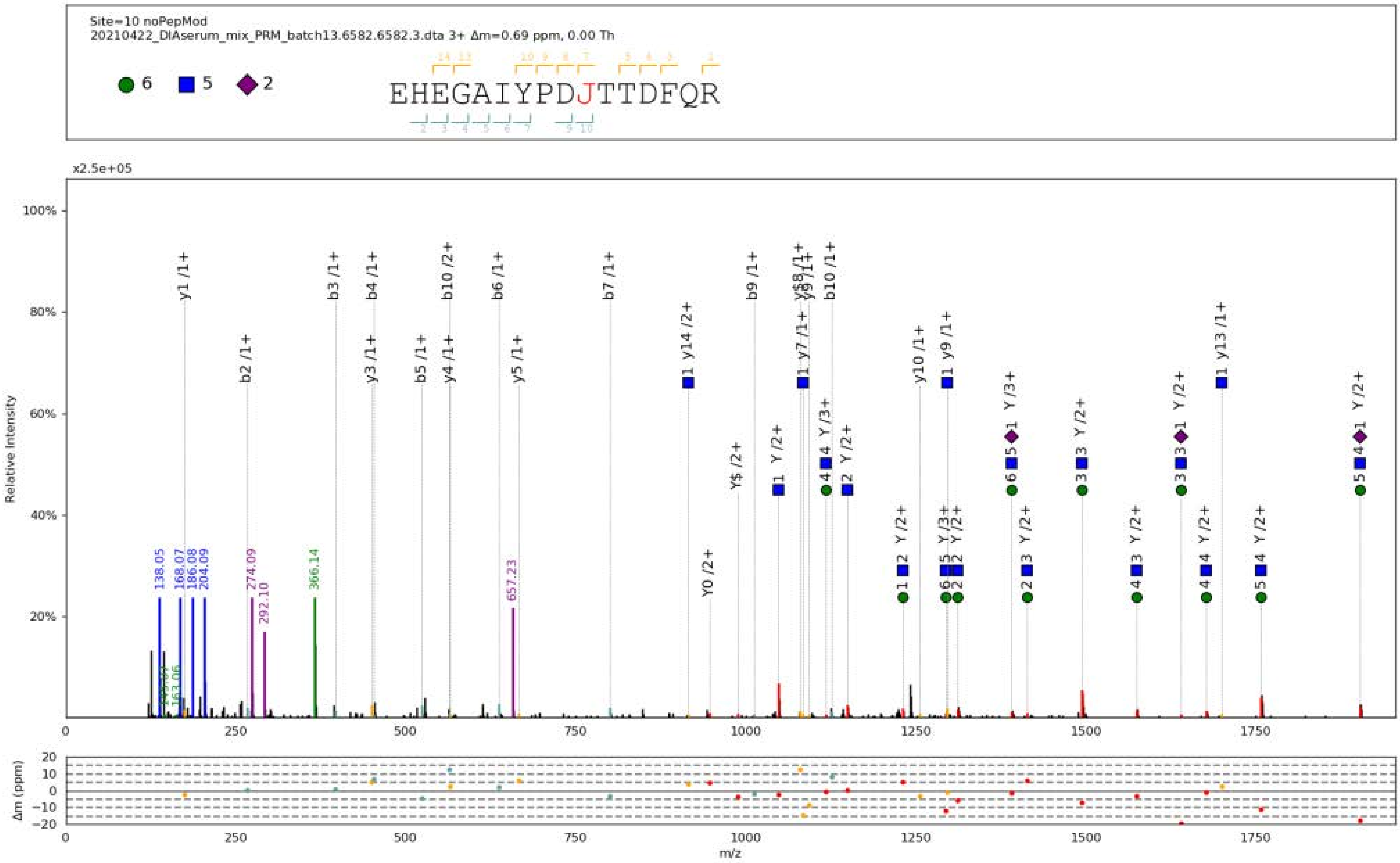

Site=10 noPepMod
20210422_DIAserum_mix_PRM_batch13.6582.6582.3.dta 3+ Δm=0.69 ppm, 0.00 Th
6
5
2
EHEGAIYPDJTTDFQR
x2.5e+05
Relative Intensity
Δm (ppm)
m/z

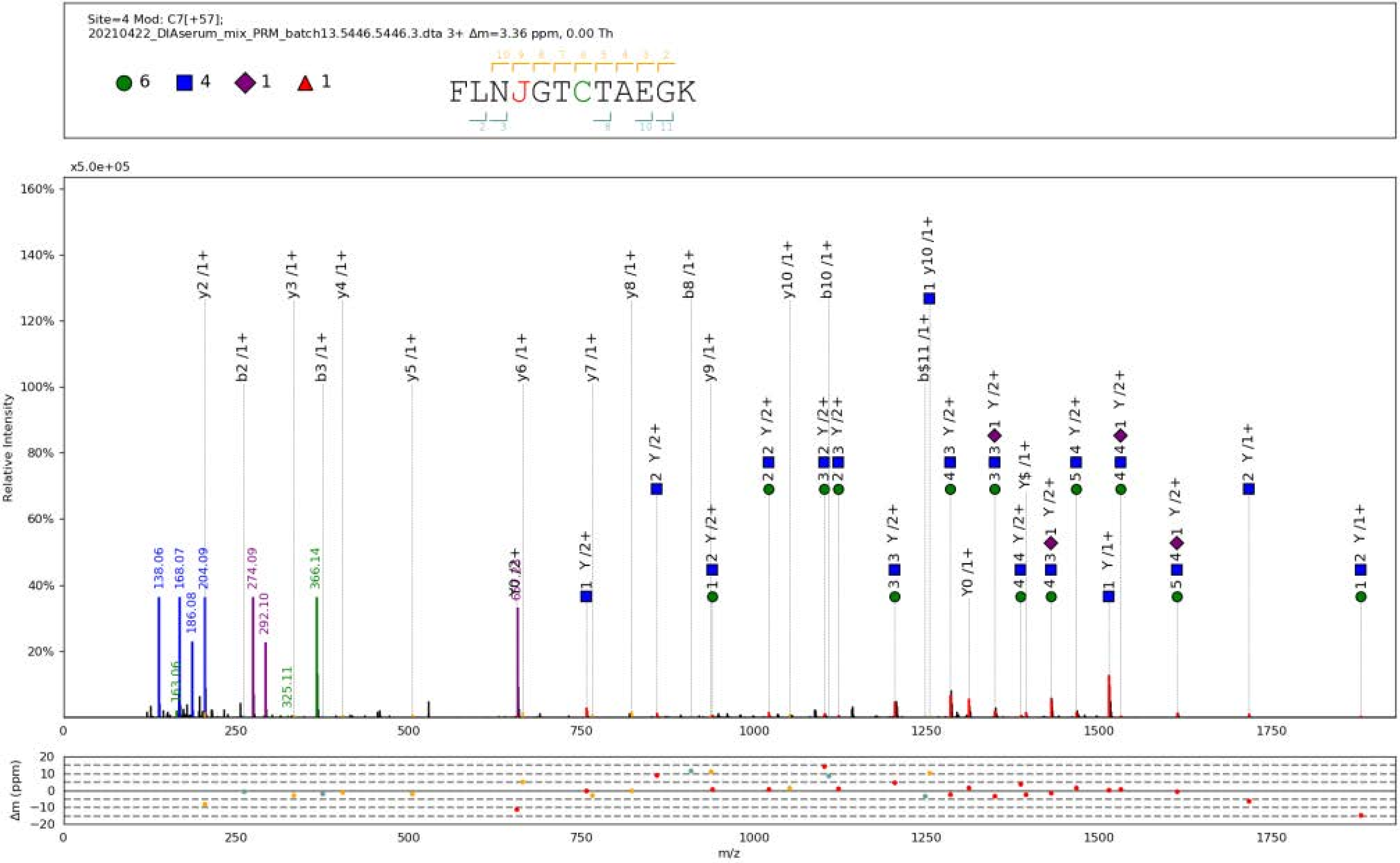
Site=4 Mod: C7[+57];
20210422_DIAserum_mix_PRM_batch13.5446.5446.3.dta 3+ Δm=3.36 ppm, 0.00 Th
FLNJGTCTAEGK
Relative Intensity
x5.0e+05
m/z
Δm (ppm)

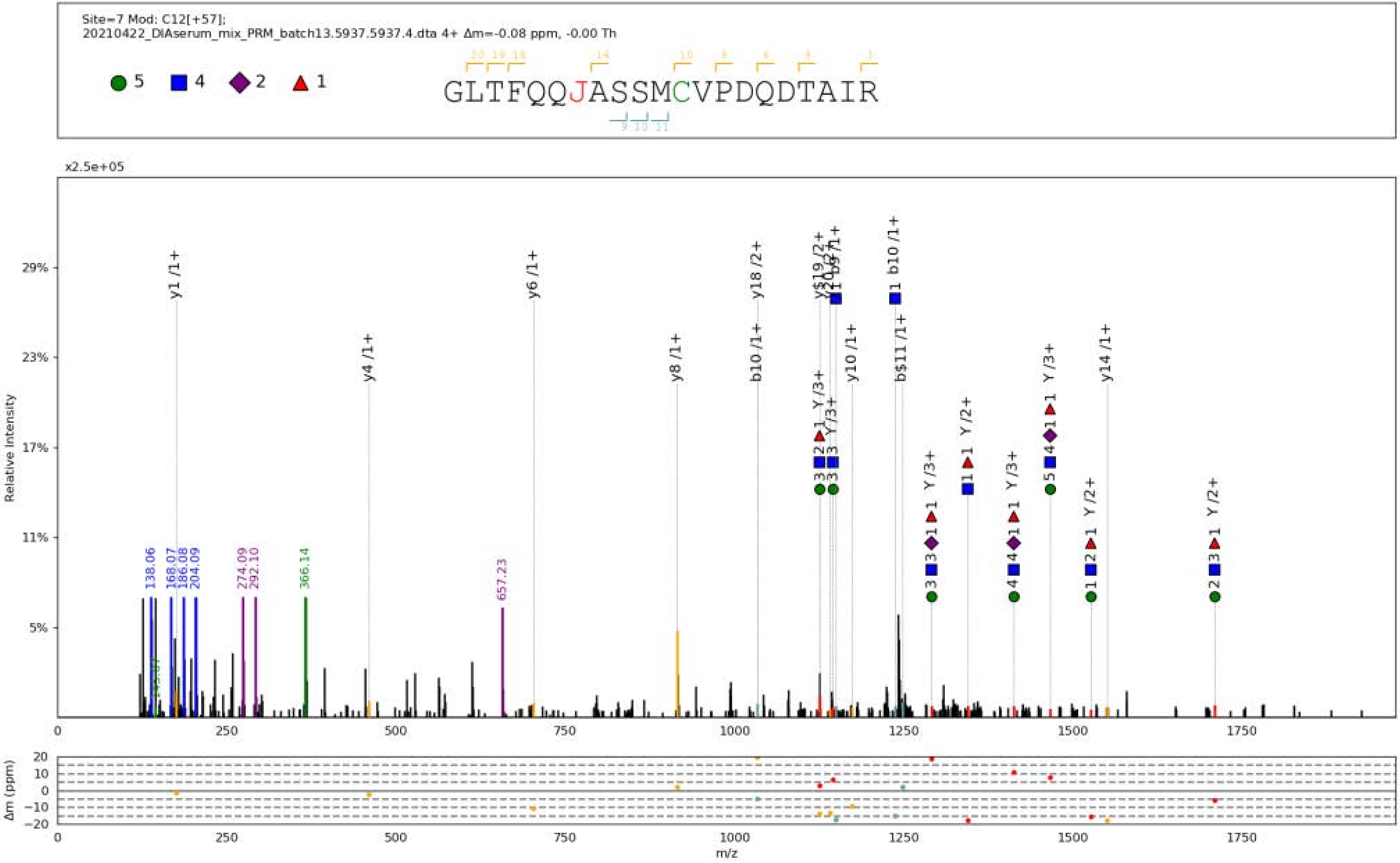

Site=7 Mod: C12[+57];
20210422_DIAserum_mix_PRM_batch13.5937.5937.4.dta 4+ Δm=-0.08 ppm, -0.00 Th
5 4 2 1
GLTFQQJASSMCVPDQDTAIR
x2.5e+05
Relative Intensity
m/z
Δm (ppm)
y1 /1+
y4 /1+
y6 /1+
y8 /1+
b10 /1+
y18 /2+
y10 /1+
b$11 /1+
b10 /1+
y14 /1+
138.06
168.07
186.08
204.09
274.09
292.10
366.14
657.23

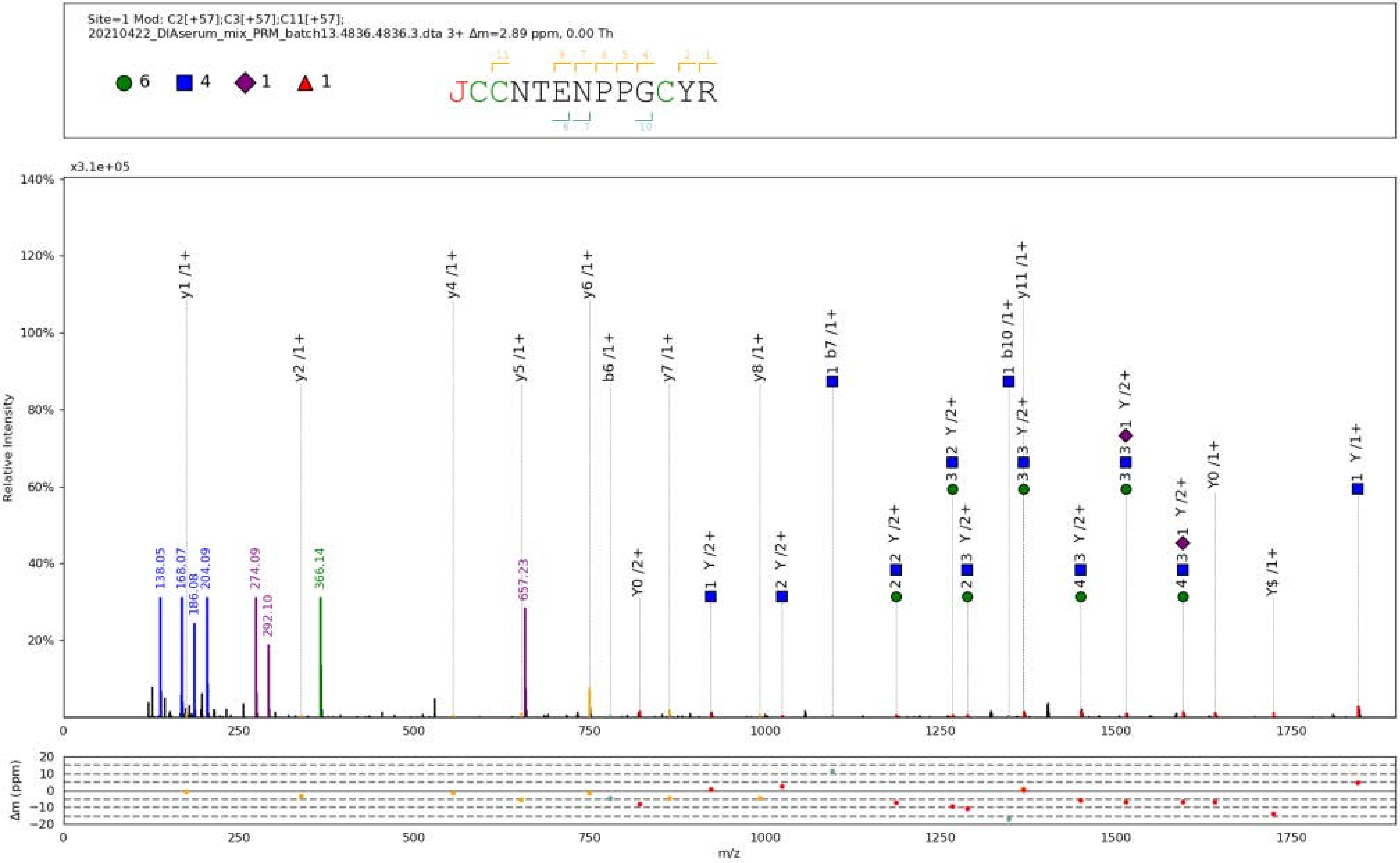
Site=1 Mod: C2[+57];C3[+57];C11[+57];
20210422_DIAserum_mix_PRM_batch13.4836.4836.3.dta 3+ Δm=2.89 ppm, 0.00 Th
6
4
1
1
JCCNTENPPGCYR
x3.1e+05
Relative Intensity
y1 /1+
y2 /1+
y4 /1+
y5 /1+
y6 /1+
b6 /1+
y7 /1+
y8 /1+
b7 /1+
b10 /1+
y11 /1+
Y0 /2+
Y0 /1+
Y$ /1+
Y /2+
Y /1+
138.05
168.07
186.08
204.09
274.09
292.10
366.14
657.23
m/z
Δm (ppm)

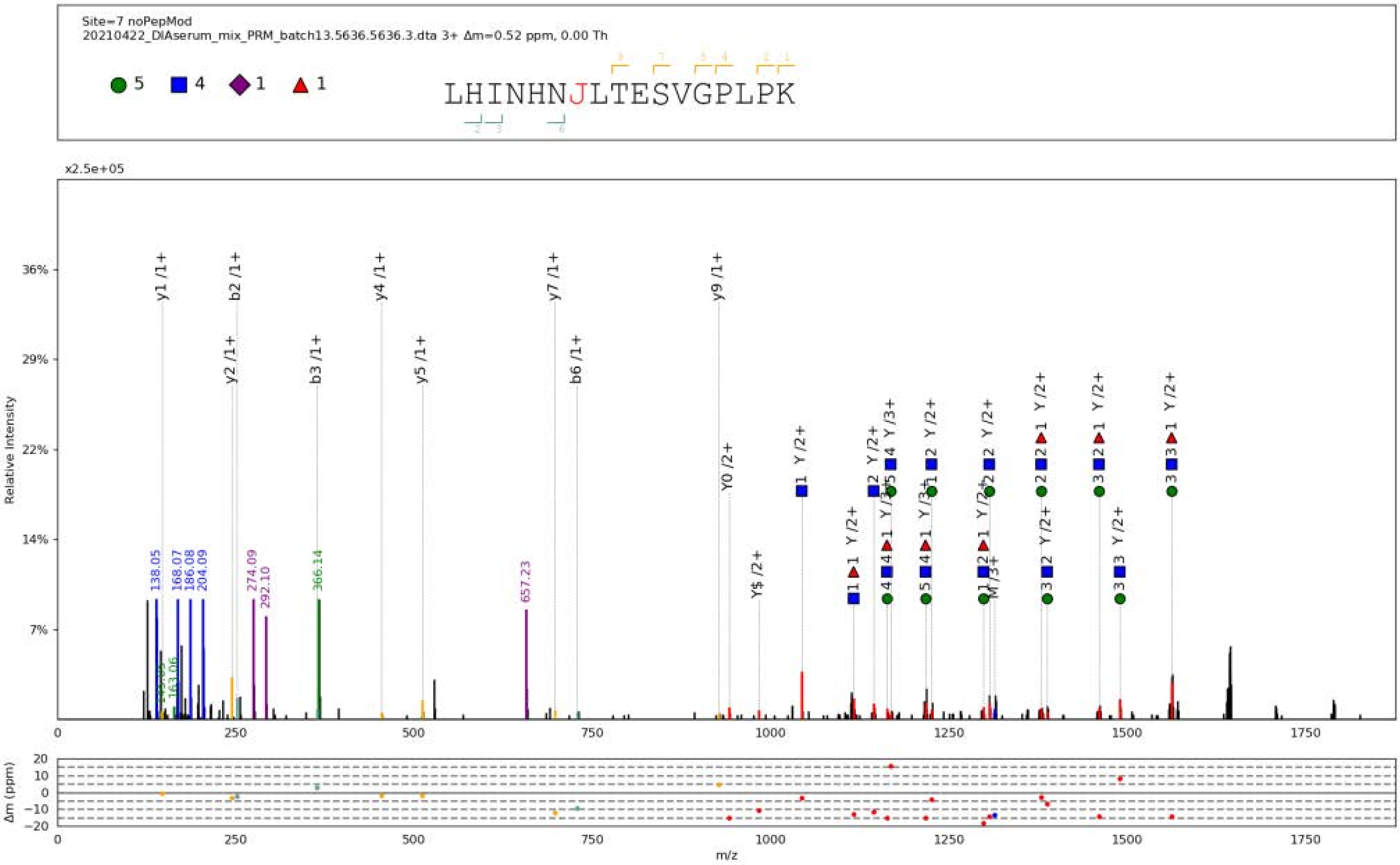

Site=7 noPepMod
20210422_DIAserum_mix_PRM_batch13.5636.5636.3.dta 3+ Δm=0.52 ppm, 0.00 Th
LHINHNJLTESVGPLPK
x2.5e+05
Relative Intensity
m/z
Δm (ppm)
y1 /1+
b2 /1+
y2 /1+
b3 /1+
y4 /1+
y5 /1+
y7 /1+
b6 /1+
y9 /1+
Y0 /2+
Y$ /2+
138.05
168.07
186.08
204.09
274.09
292.10
366.14
657.23
163.06

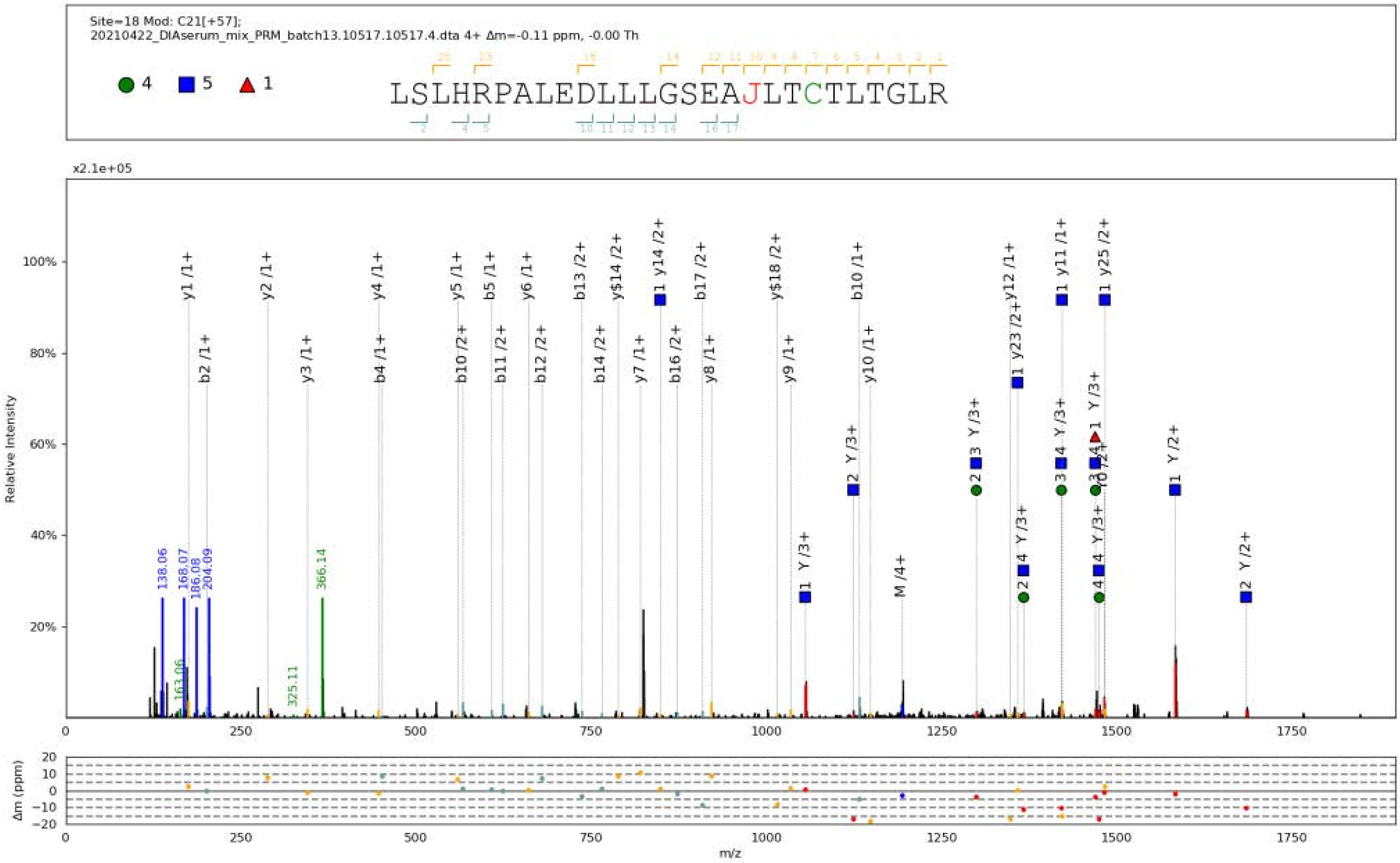
Site=18 Mod: C21[+57];
20210422_DIAserum_mix_PRM_batch13.10517.10517.4.dta 4+ Δm=-0.11 ppm, -0.00 Th
LSLHRPALEDLLLGSEAJLTCTLTGLR
x2.1e+05
Relative Intensity
m/z
Δm (ppm)

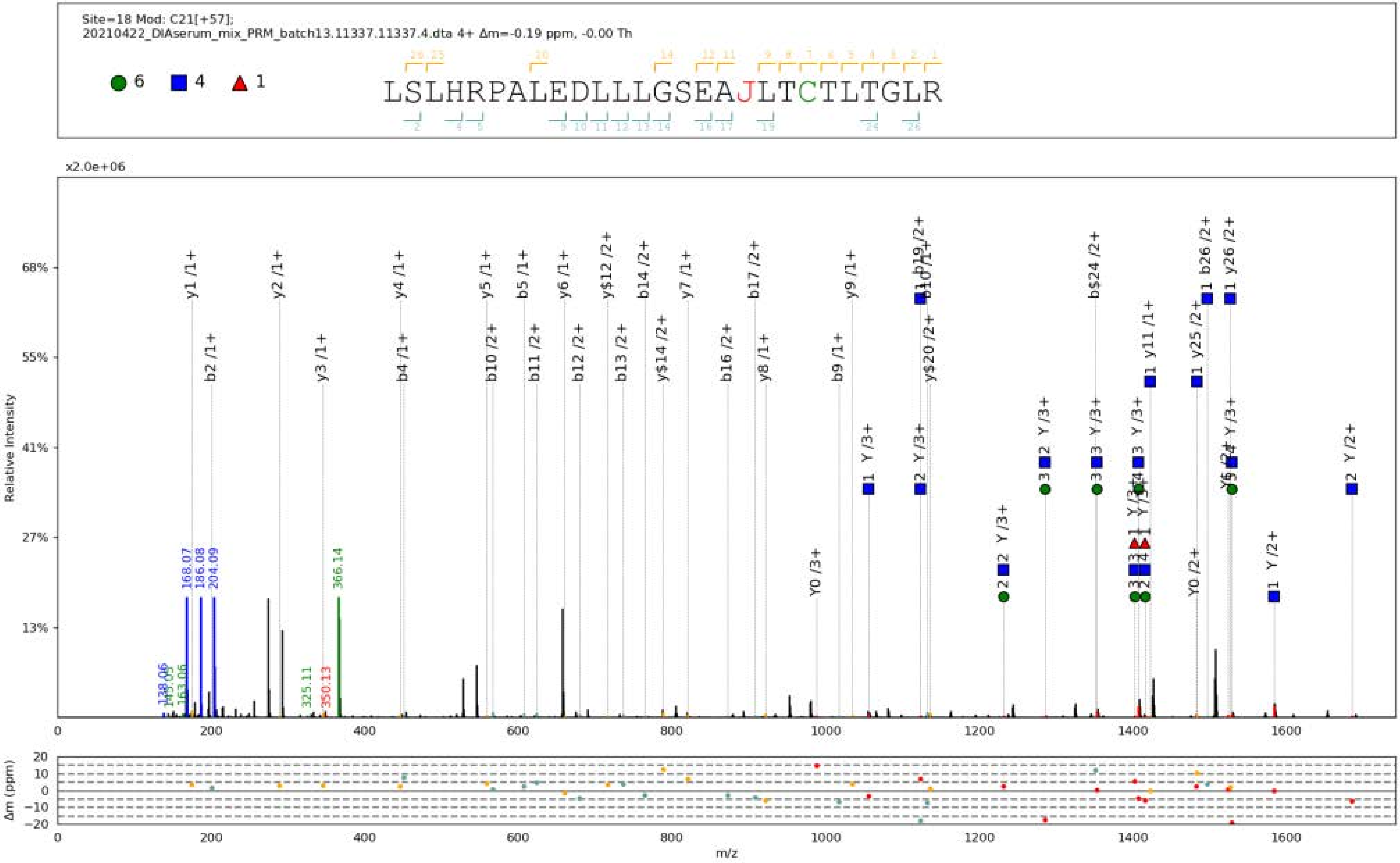
Site=18 Mod: C21[+57];
20210422_DIAserum_mix_PRM_batch13.11337.11337.4.dta 4+ Δm=-0.19 ppm, -0.00 Th
LSLHRPALEDLLLGSEAJLTCTLTGLR
x2.0e+06
Relative Intensity
m/z
Δm (ppm)

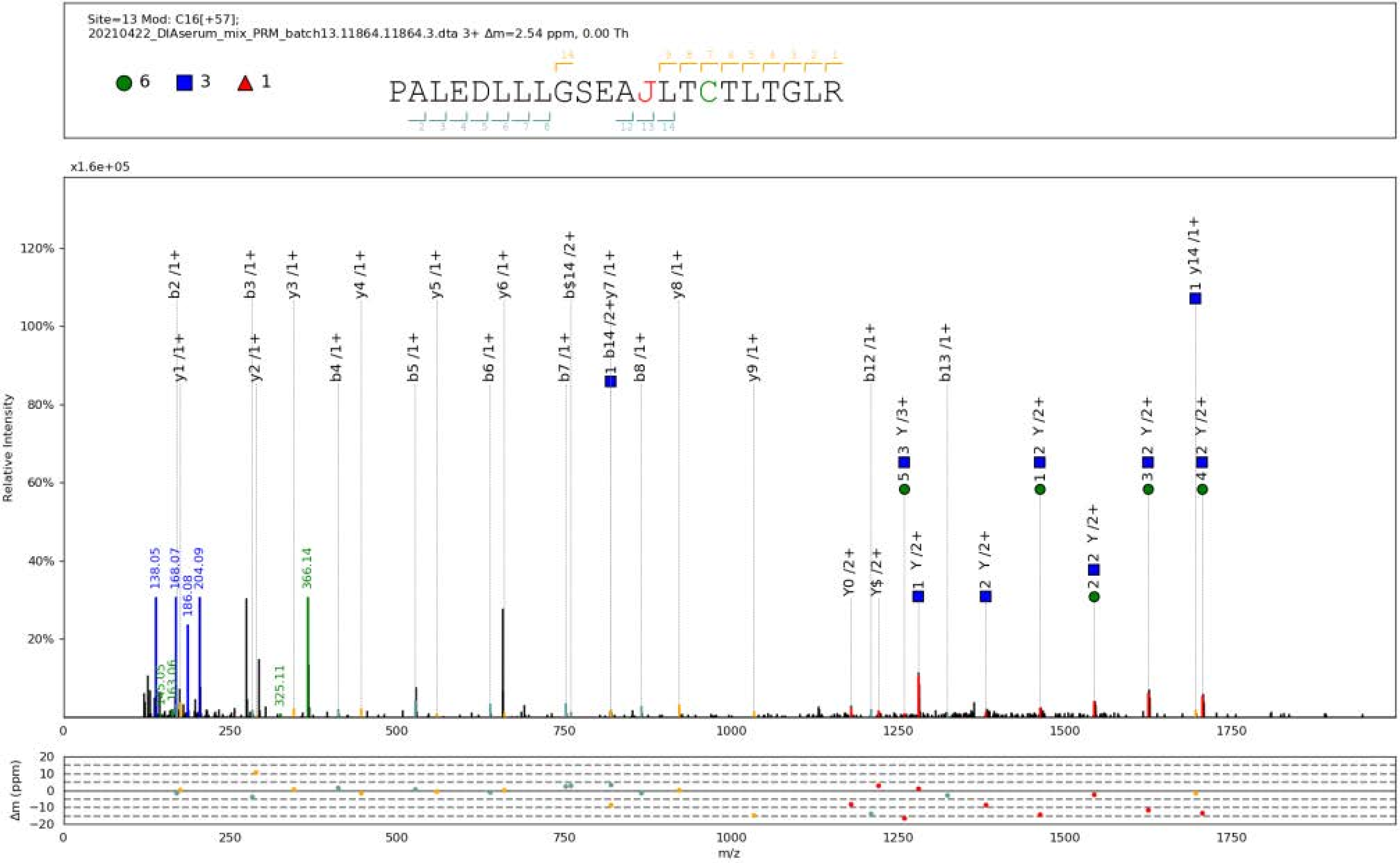

Site=13 Mod: C16[+57];
20210422_DIAserum_mix_PRM_batch13.11864.11864.3.dta 3+ Δm=2.54 ppm, 0.00 Th
6
3
1
PALEDLLLGSEAJLTCTLTGLR
x1.6e+05
Relative Intensity
b2 /1+
y1 /1+
b3 /1+
y2 /1+
y3 /1+
b4 /1+
y4 /1+
b5 /1+
y5 /1+
b6 /1+
y6 /1+
b7 /1+
b$14 /2+
b14 /2+y7 /1+
b8 /1+
y8 /1+
y9 /1+
Y0 /2+
b12 /1+
Y$ /2+
Y /3+
Y /2+
b13 /1+
y14 /1+
138.05
168.07
186.08
204.09
366.14
325.11
Δm (ppm)
m/z

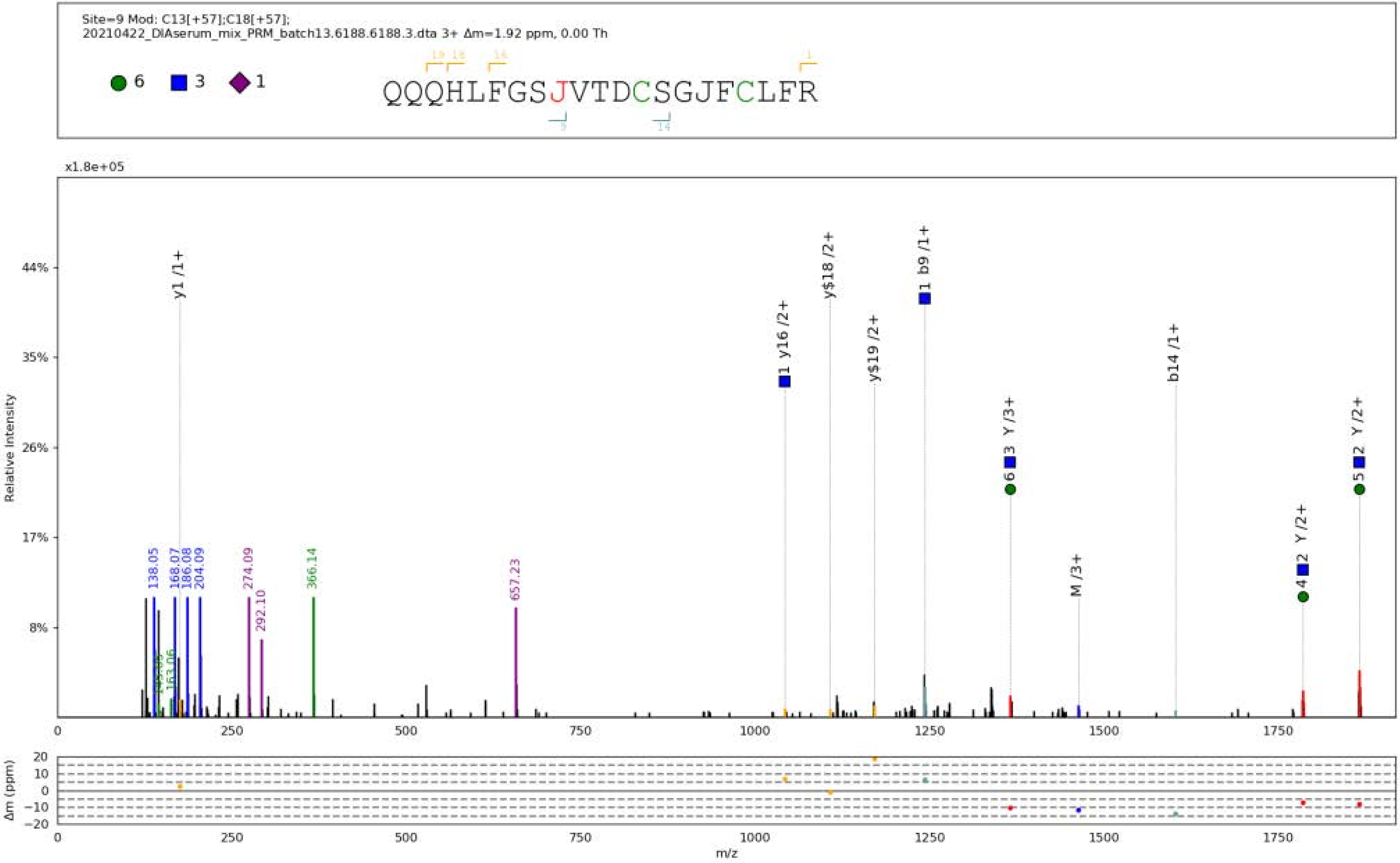
Site=9 Mod: C13[+57];C18[+57];
20210422_DIAserum_mix_PRM_batch13.6188.6188.3.dta 3+ Δm=1.92 ppm, 0.00 Th
6
3
1
QQQHLFGSJVTDCSGJFCLFR
x1.8e+05
Relative Intensity
y1 /1+
1 y16 /2+
y$18 /2+
y$19 /2+
1 b9 /1+
b14 /1+
6 3 Y /3+
M /3+
4 2 Y /2+
5 2 Y /2+
138.05
168.07
186.08
204.09
274.09
292.10
366.14
657.23
m/z
Δm (ppm)

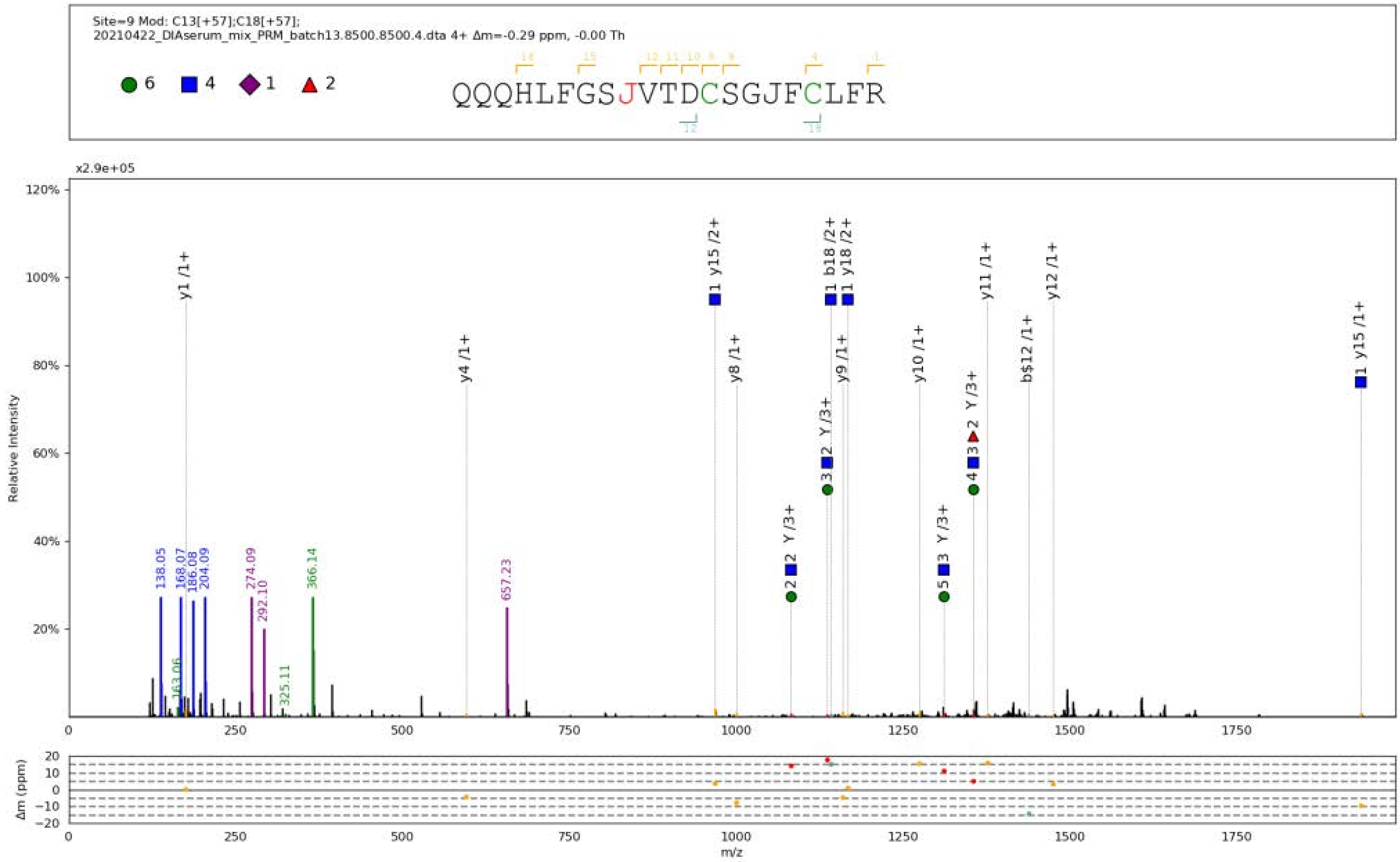

Site=9 Mod: C13[+57];C18[+57];
20210422_DIAserum_mix_PRM_batch13.8500.8500.4.dta 4+ Δm=-0.29 ppm, -0.00 Th
6 4 1 2
QQQHLFGSJVTDCSGJFCLFR
x2.9e+05
Relative Intensity
y1 /1+
y4 /1+
1 y15 /2+
y8 /1+
2 2 Y /3+
3 2 Y /3+
1 b18 /2+
1 y18 /2+
y9 /1+
y10 /1+
5 3 Y /3+
4 3 2 Y /3+
y11 /1+
b$12 /1+
y12 /1+
1 y15 /1+
138.05
168.07
186.08
204.09
163.06
274.09
292.10
325.11
366.14
657.23
m/z
Δm (ppm)

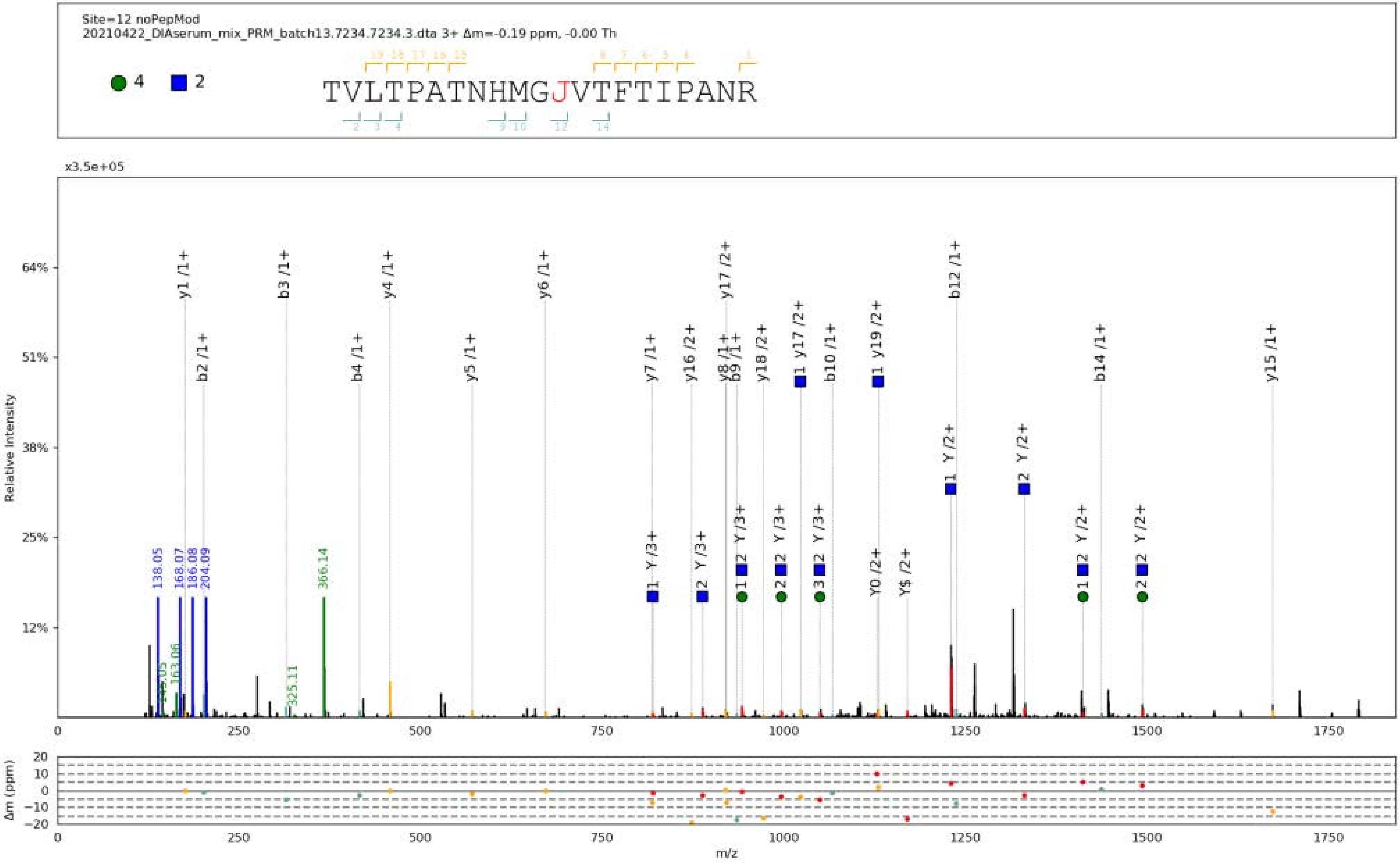

Site=12 noPepMod
20210422_DIAserum_mix_PRM_batch13.7234.7234.3.dta 3+ Δm=-0.19 ppm, -0.00 Th
4
2
TVLTPATNHMGJVTFTIPANR
x3.5e+05
Relative Intensity
64%
51%
38%
25%
12%
y1 /1+
b2 /1+
b3 /1+
b4 /1+
y4 /1+
y5 /1+
y6 /1+
y7 /1+
y16 /2+
y8 /1+
b9 /1+
y17 /2+
y18 /2+
1 y17 /2+
b10 /1+
1 y19 /2+
b12 /1+
b14 /1+
y15 /1+
1 Y /2+
2 Y /2+
1 Y /3+
2 Y /3+
Y0 /2+
Y$ /2+
138.05
168.07
186.08
204.09
366.14
163.06
325.11
m/z
Δm (ppm)

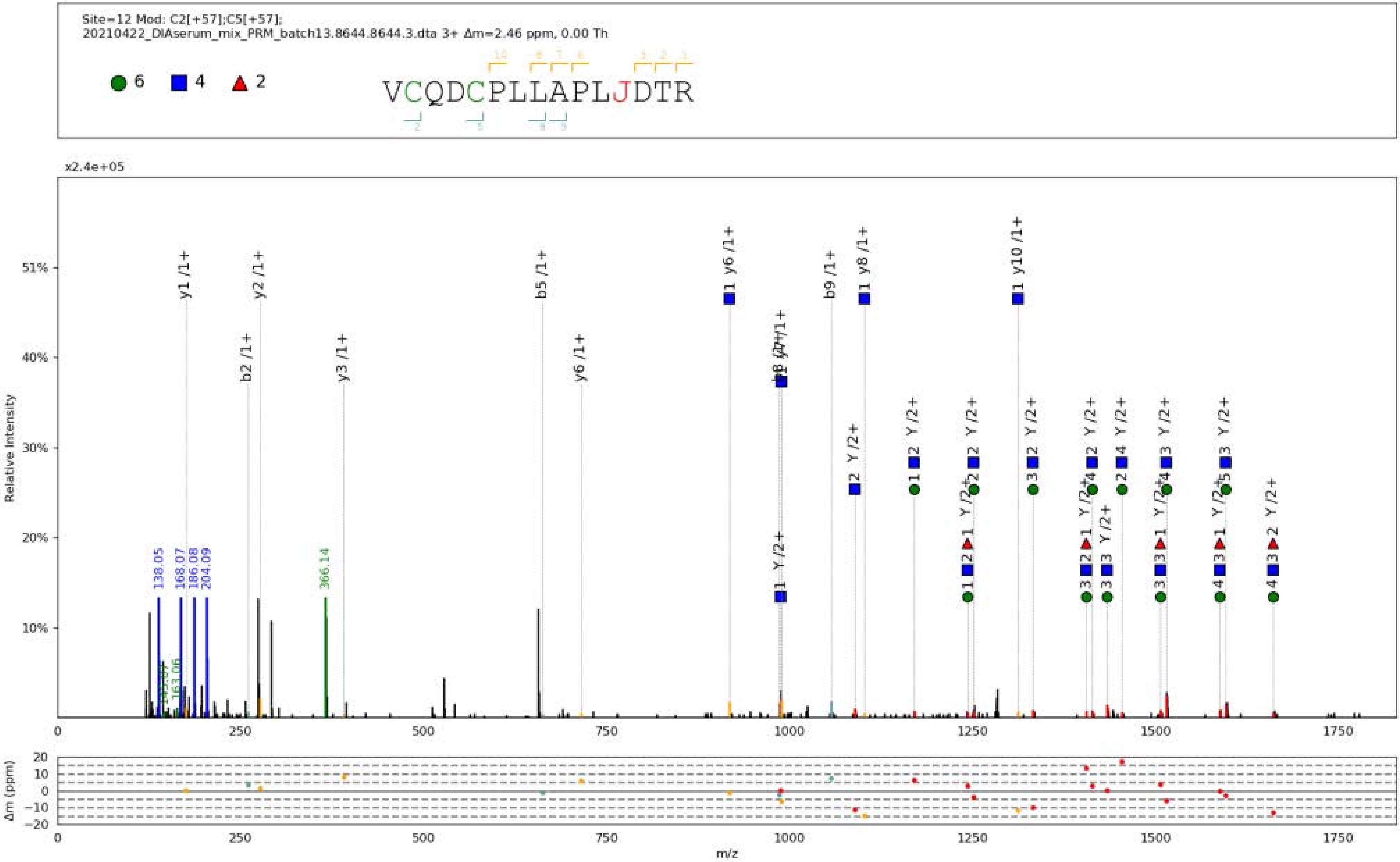

Site=12 Mod: C2[+57];C5[+57];
20210422_DIAserum_mix_PRM_batch13.8644.8644.3.dta 3+ Δm=2.46 ppm, 0.00 Th
6
4
2
VCQDCPLLAPLJDTR
x2.4e+05
Relative Intensity
51%
40%
30%
20%
10%
y1 /1+
y2 /1+
b2 /1+
y3 /1+
b5 /1+
y6 /1+
1 y6 /1+
b9 /1+
1 y8 /1+
1 y10 /1+
Y /2+
138.05
168.07
186.08
204.09
366.14
163.06
0
250
500
750
1000
1250
1500
1750
Δm (ppm)
20
10
0
−10
−20
m/z

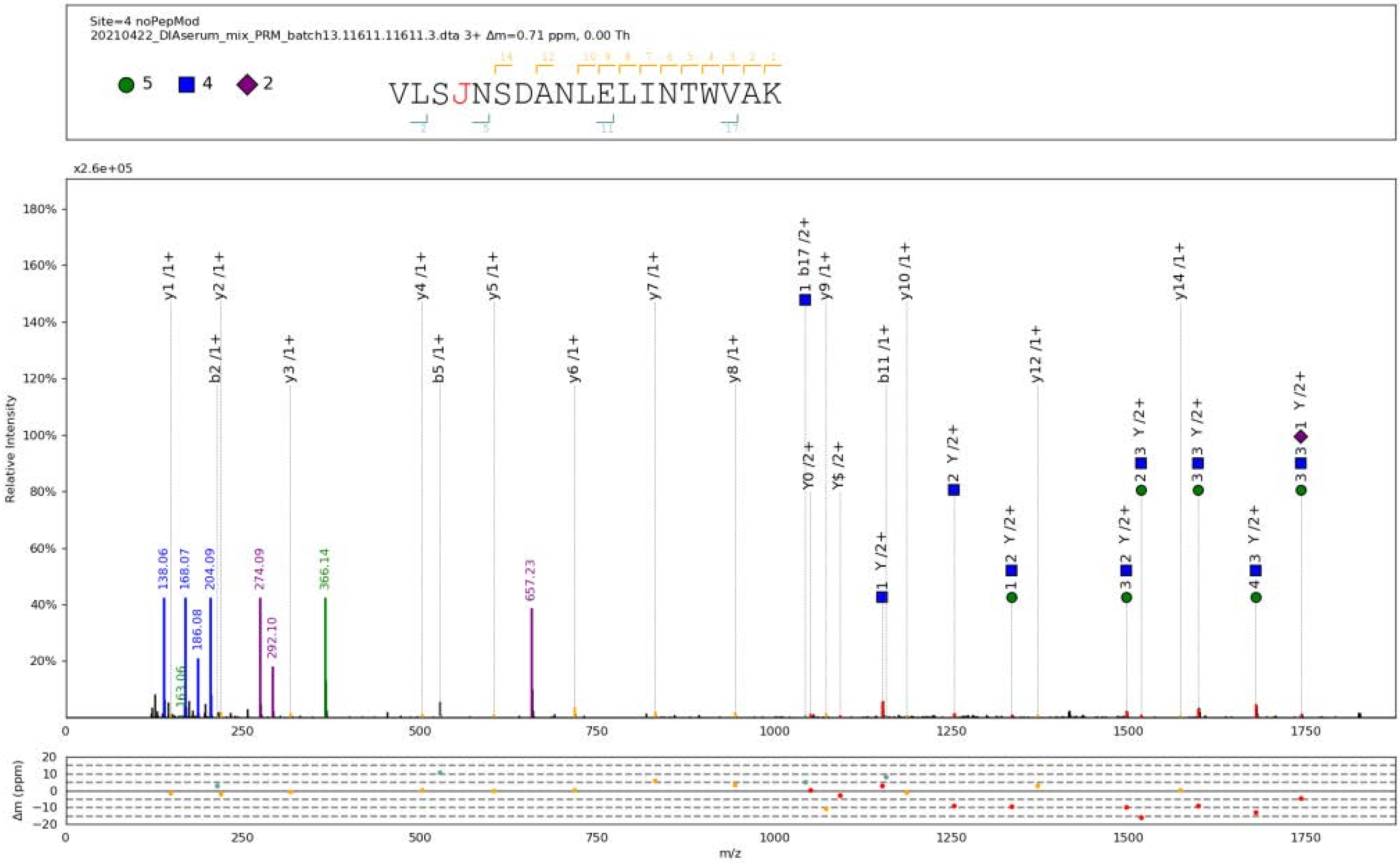

Site=4 noPepMod
20210422_DIAserum_mix_PRM_batch13.11611.11611.3.dta 3+ Δm=0.71 ppm, 0.00 Th
5 4 2
VLSJNSDANLELINTWVAK
x2.6e+05
Relative Intensity
y1 /1+
y2 /1+
b2 /1+
y3 /1+
y4 /1+
b5 /1+
y5 /1+
y6 /1+
y7 /1+
y8 /1+
b17 /2+
y9 /1+
Y0 /2+
Y$ /2+
b11 /1+
y10 /1+
y12 /1+
y14 /1+
138.06
168.07
204.09
186.08
163.06
274.09
292.10
366.14
657.23
m/z
Δm (ppm)

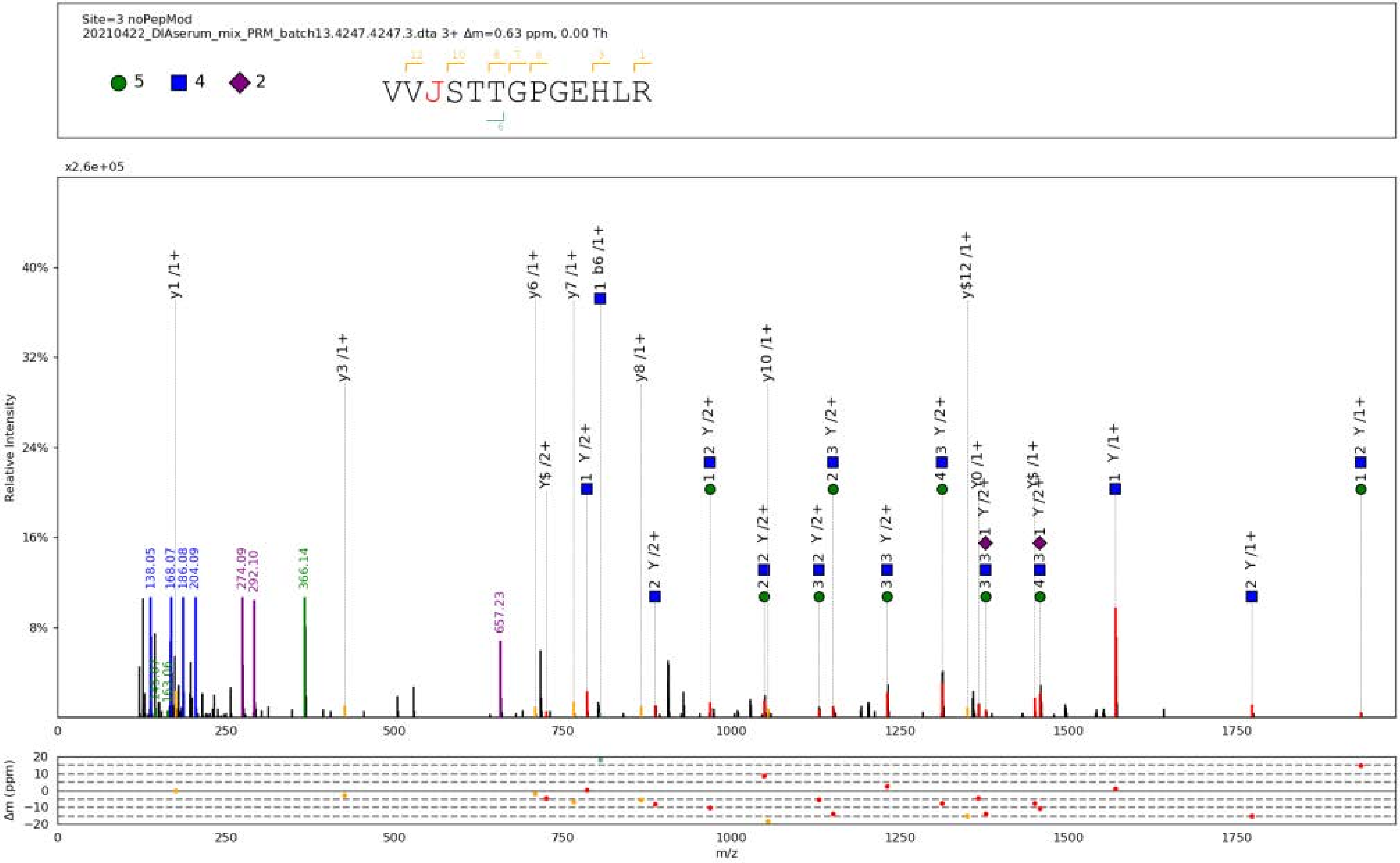

Site=3 noPepMod
20210422_DIAserum_mix_PRM_batch13.4247.4247.3.dta 3+ Δm=0.63 ppm, 0.00 Th
VVJSTTGPGEHLR
x2.6e+05
Relative Intensity
m/z
Δm (ppm)

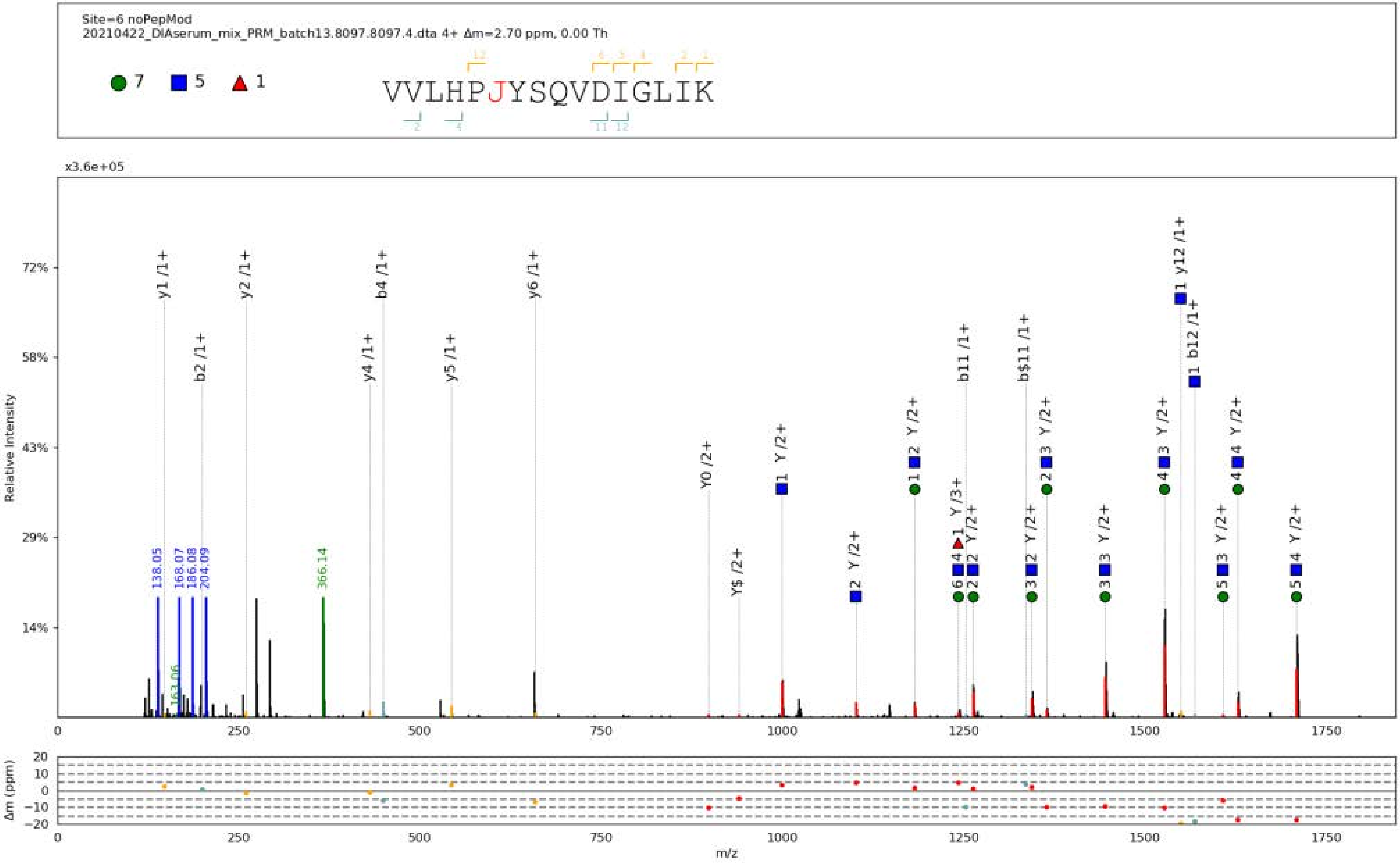
Site=6 noPepMod
20210422_DIAserum_mix_PRM_batch13.8097.8097.4.dta 4+ Δm=2.70 ppm, 0.00 Th
7
5
1
VVLHPJYSQVDIGLIK
x3.6e+05
Relative Intensity
72%
58%
43%
29%
14%
y1 /1+
b2 /1+
y2 /1+
y4 /1+
b4 /1+
y5 /1+
y6 /1+
Y0 /2+
Y$ /2+
Y /2+
Y /3+
b11 /1+
b$11 /1+
y12 /1+
b12 /1+
138.05
168.07
186.08
204.09
163.06
366.14
m/z
Δm (ppm)
0
250
500
750
1000
1250
1500
1750

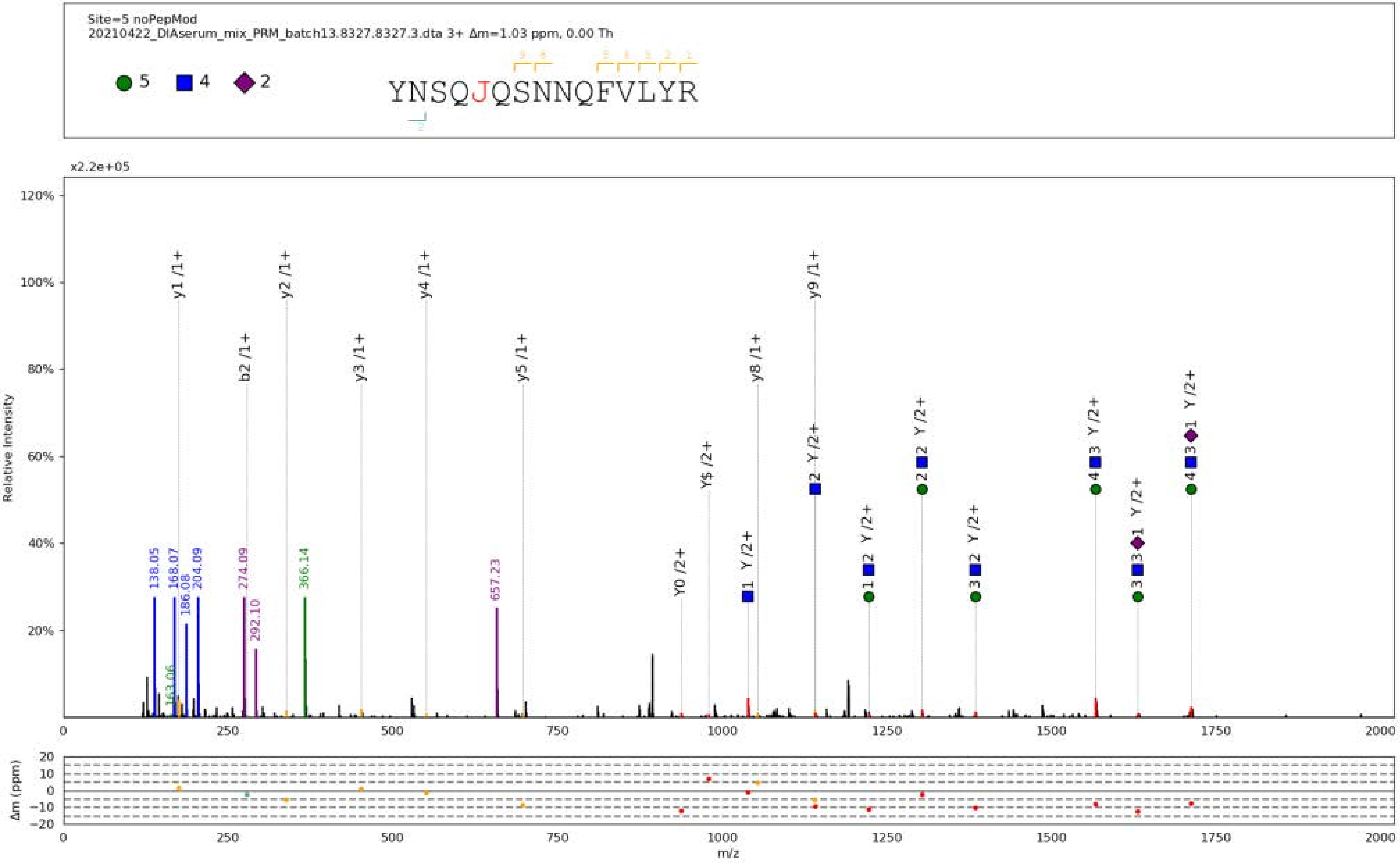
Site=5 noPepMod
20210422_DIAserum_mix_PRM_batch13.8327.8327.3.dta 3+ Δm=1.03 ppm, 0.00 Th
5
4
2
YNSQJQSNNQFVLYR
x2.2e+05
Relative Intensity
y1 /1+
y2 /1+
y4 /1+
b2 /1+
y3 /1+
y5 /1+
y8 /1+
y9 /1+
Y0 /2+
Y$ /2+
138.05
168.07
186.08
204.09
163.06
274.09
292.10
366.14
657.23
m/z
Δm (ppm)

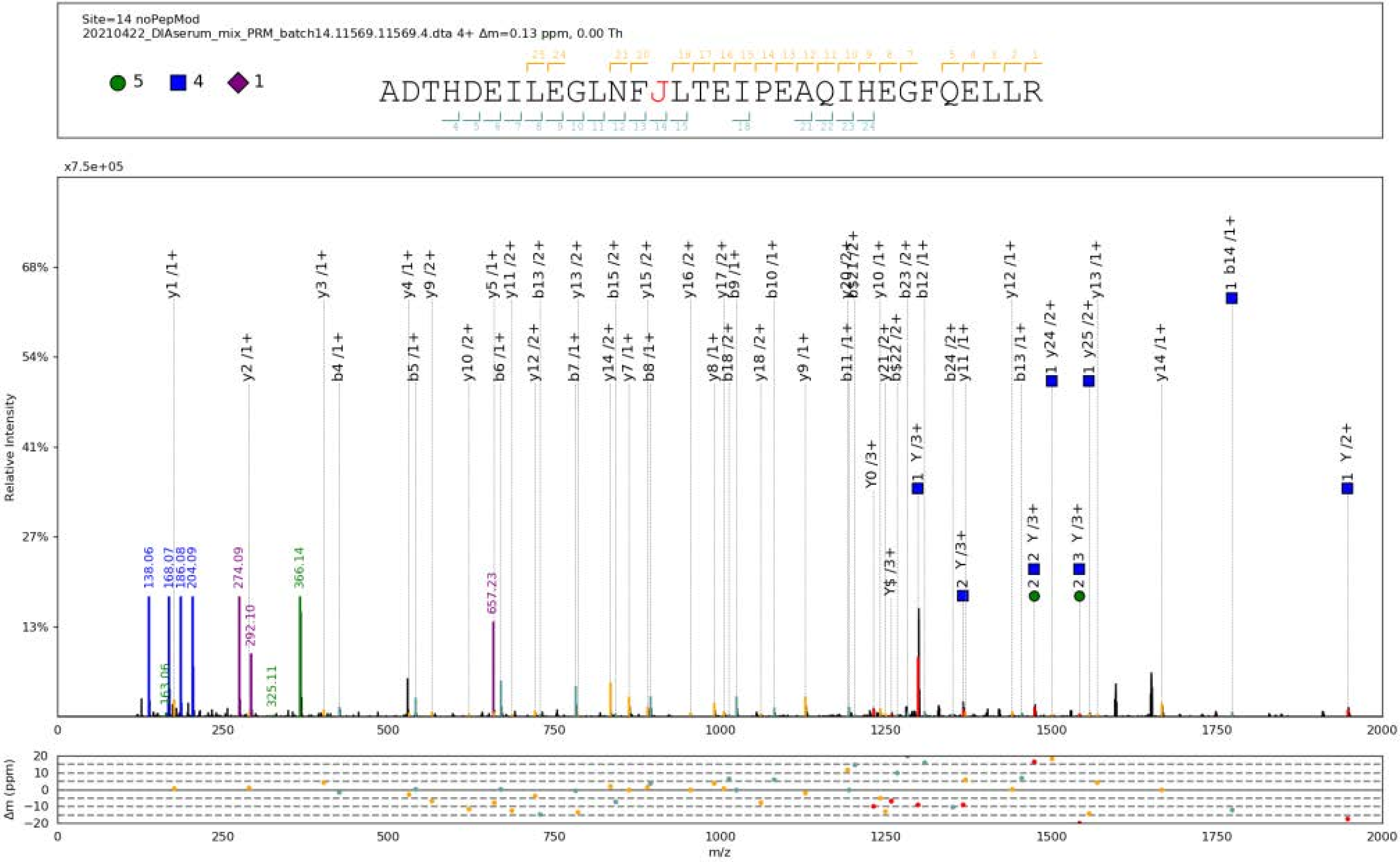
Site=14 noPepMod
20210422_DIAserum_mix_PRM_batch14.11569.11569.4.dta 4+ Δm=0.13 ppm, 0.00 Th
ADTHDEILEGLNFJLTEIPEAQIHEGFQELLR
x7.5e+05
Relative Intensity
m/z
Δm (ppm)

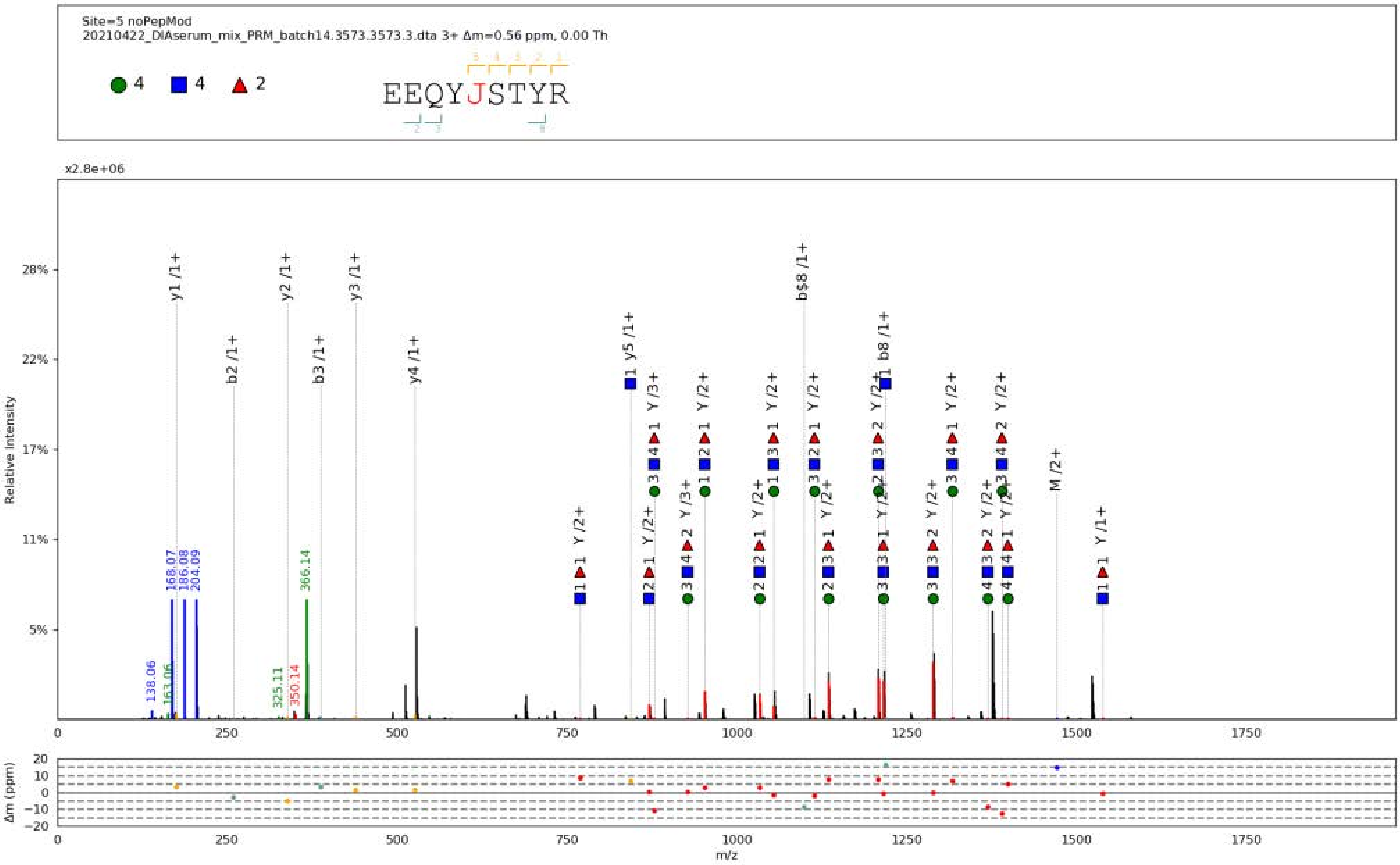

Site=5 noPepMod
20210422_DIAserum_mix_PRM_batch14.3573.3573.3.dta 3+ Δm=0.56 ppm, 0.00 Th
EEQYJSTYR
x2.8e+06
Relative Intensity
m/z
Δm (ppm)

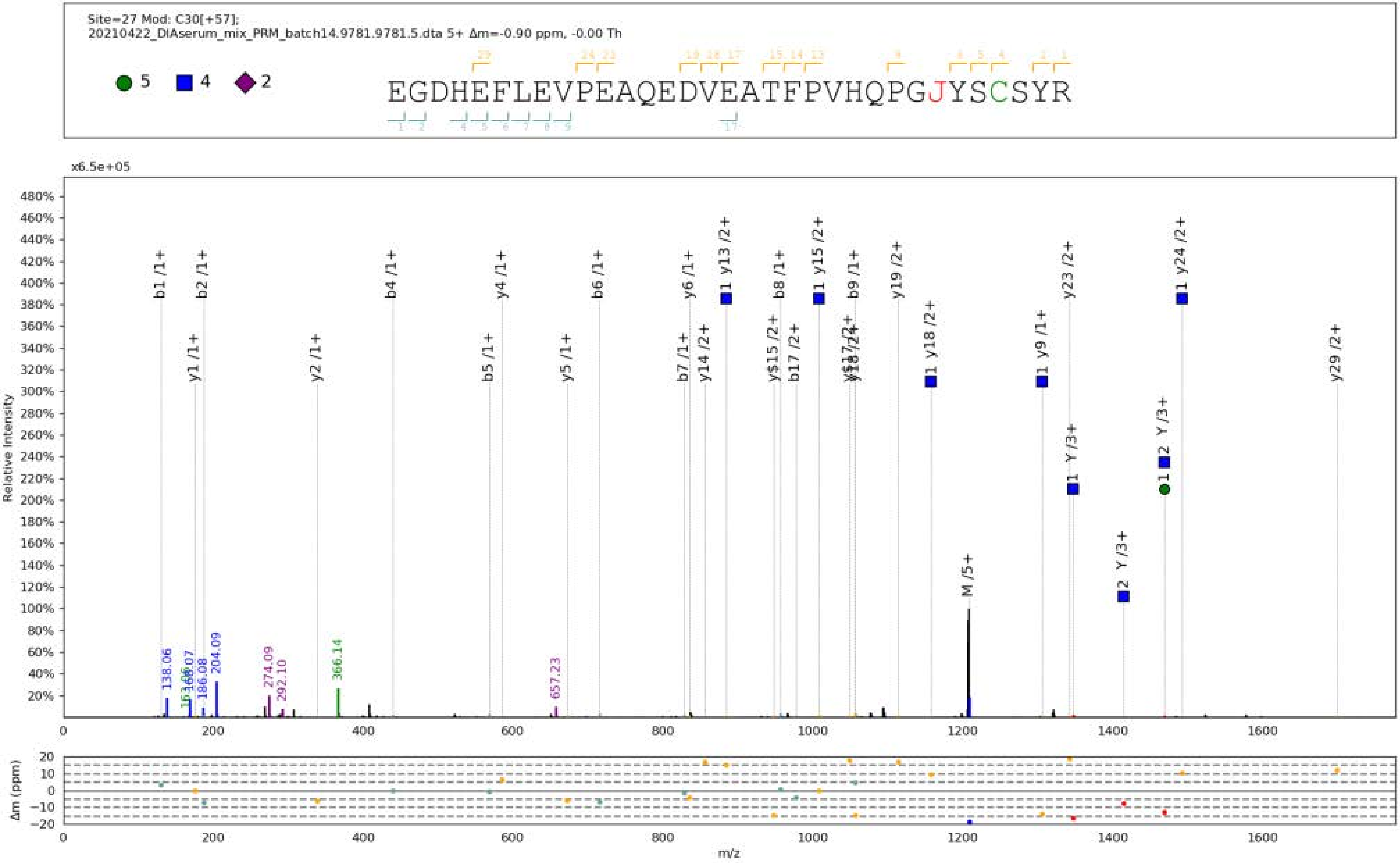

Site=27 Mod: C30[+57];
20210422_DIAserum_mix_PRM_batch14.9781.9781.5.dta 5+ Δm=-0.90 ppm, -0.00 Th
EGDHEFLEVPEAQEDVEATFPVHQPGJYSCSYR
x6.5e+05
Relative Intensity
m/z
Δm (ppm)

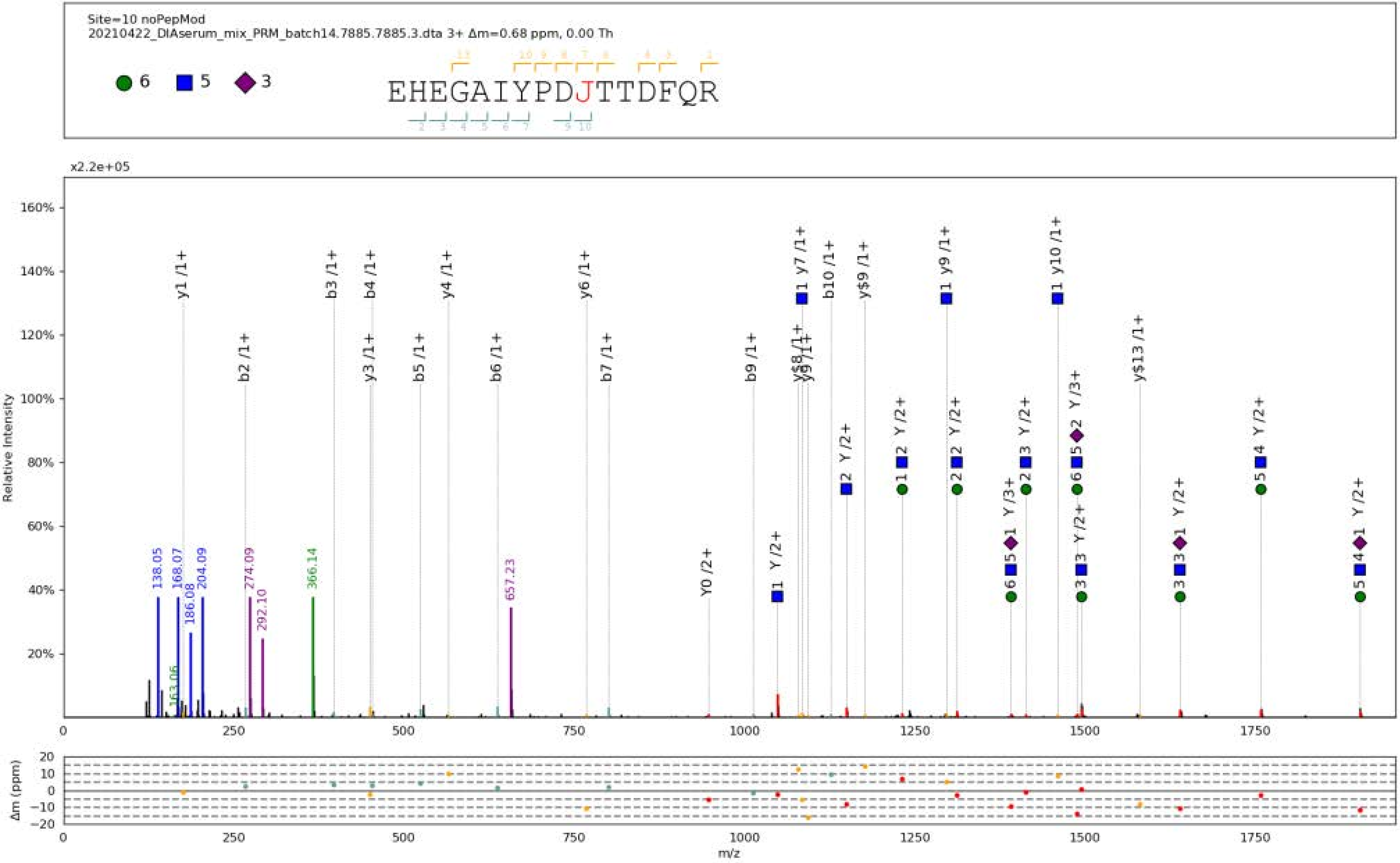

Site=10 noPepMod
20210422_DIAserum_mix_PRM_batch14.7885.7885.3.dta 3+ Δm=0.68 ppm, 0.00 Th
6
5
3
EHEGAIYPDJTTDFQR
x2.2e+05
Relative Intensity
y1 /1+
b2 /1+
b3 /1+
b4 /1+
y3 /1+
b5 /1+
y4 /1+
b6 /1+
y6 /1+
b7 /1+
Y0 /2+
b9 /1+
y7 /1+
b10 /1+
y$9 /1+
y9 /1+
y10 /1+
y$13 /1+
138.05
168.07
186.08
204.09
163.06
274.09
292.10
366.14
657.23
m/z
Δm (ppm)

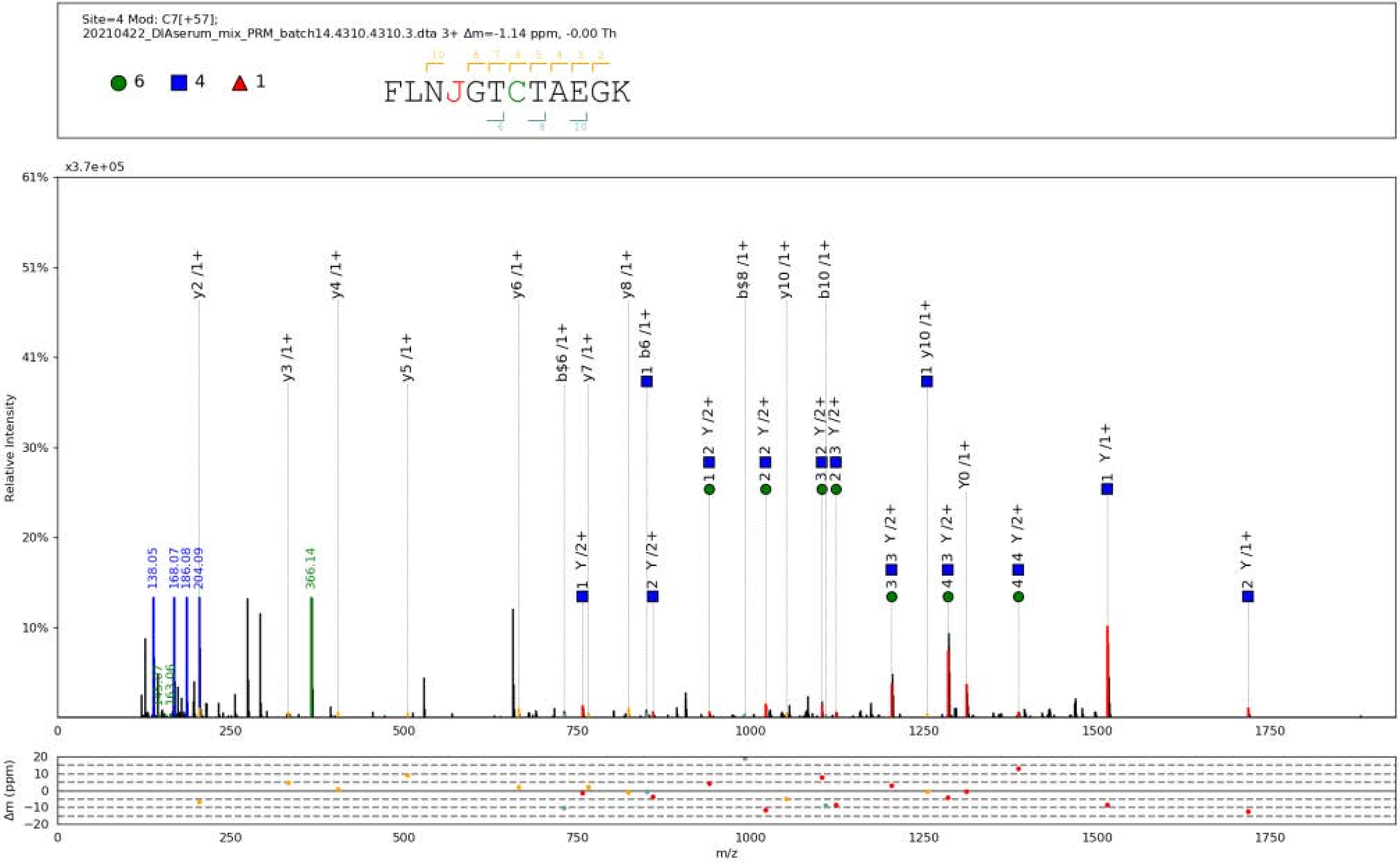
Site=4 Mod: C7[+57];
20210422_DIAserum_mix_PRM_batch14.4310.4310.3.dta 3+ Δm=-1.14 ppm, -0.00 Th
6
4
1
FLNJGTCTAEGK
x3.7e+05
Relative Intensity
y2 /1+
y3 /1+
y4 /1+
y5 /1+
y6 /1+
b$6 /1+
y7 /1+
y8 /1+
b6 /1+
b$8 /1+
y10 /1+
b10 /1+
y10 /1+
Y /2+
Y /1+
Y0 /1+
138.05
168.07
186.08
204.09
366.14
163.06
m/z
Δm (ppm)

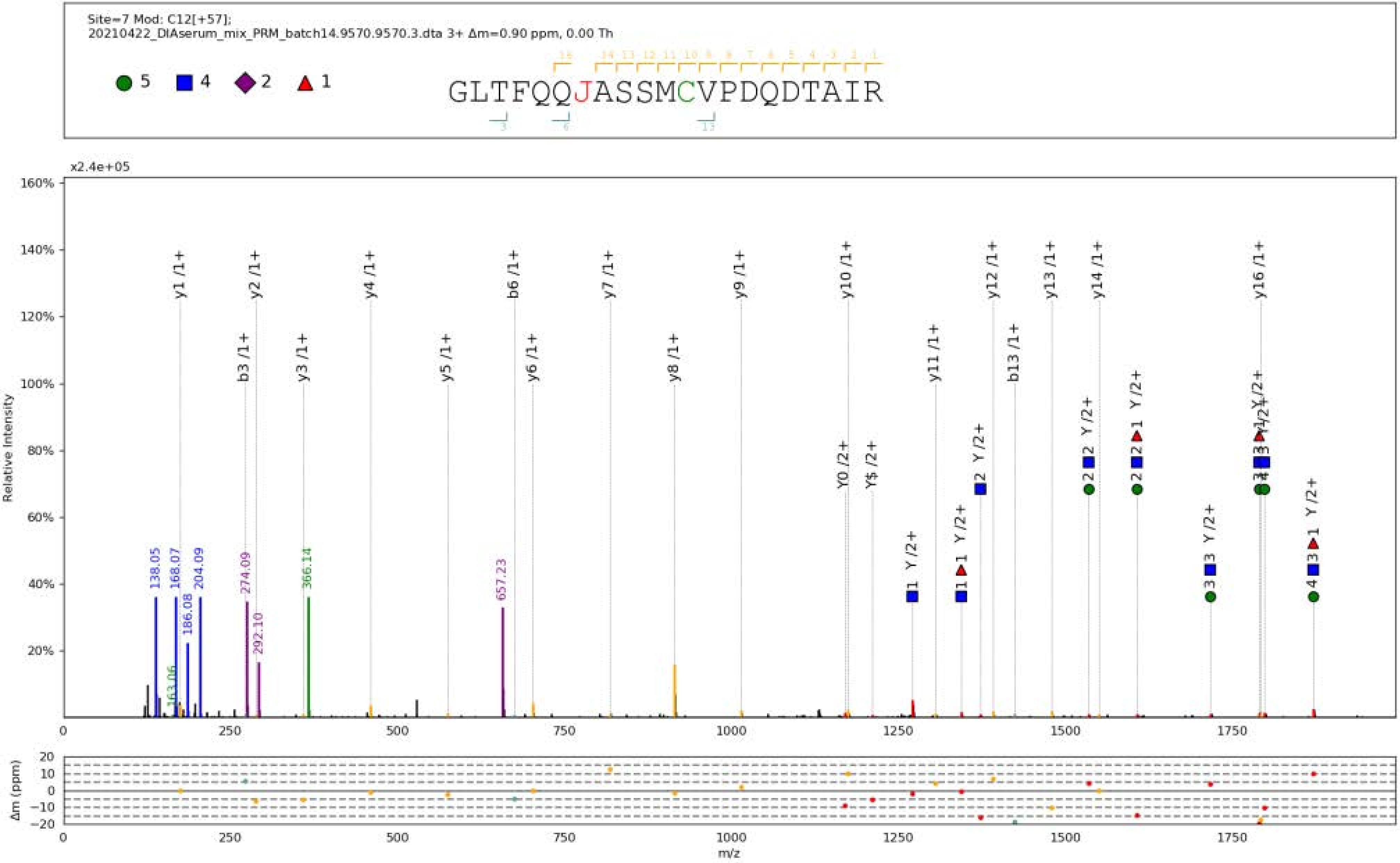

Site=7 Mod: C12[+57];
20210422_DIAserum_mix_PRM_batch14.9570.9570.3.dta 3+ Δm=0.90 ppm, 0.00 Th
GLTFQQJASSMCVPDQDTAIR
x2.4e+05
Relative Intensity
Δm (ppm)
m/z

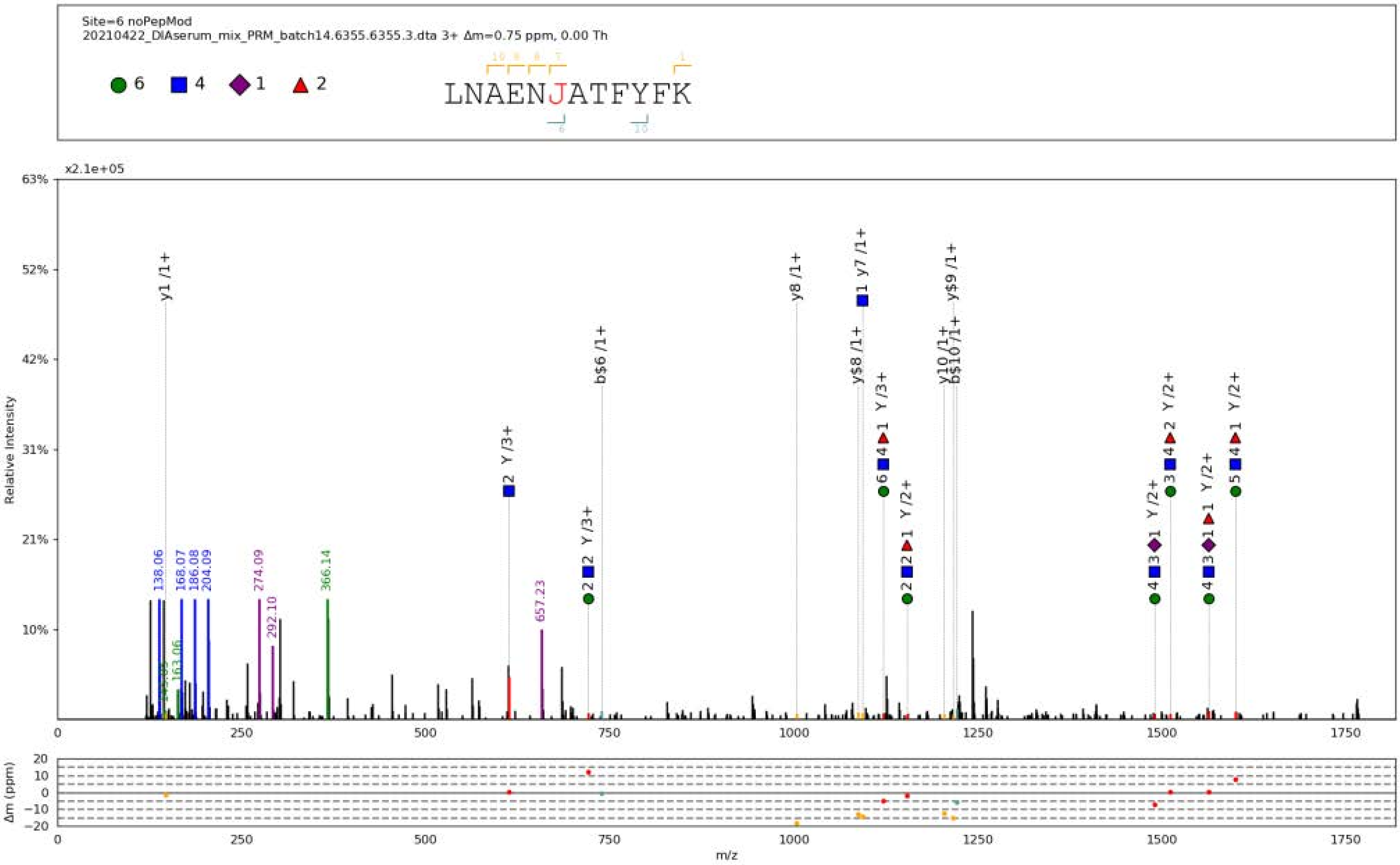

Site=6 noPepMod
20210422_DIAserum_mix_PRM_batch14.6355.6355.3.dta 3+ Δm=0.75 ppm, 0.00 Th
6 4 1 2
LNAENJATFYFK
x2.1e+05
Relative Intensity
y1 /1+
b$6 /1+
y8 /1+
y$8 /1+
1 y7 /1+
y$9 /1+
y10 /1+
b$10 /1+
138.06
168.07
186.08
204.09
274.09
292.10
366.14
163.06
657.23
m/z
Δm (ppm)

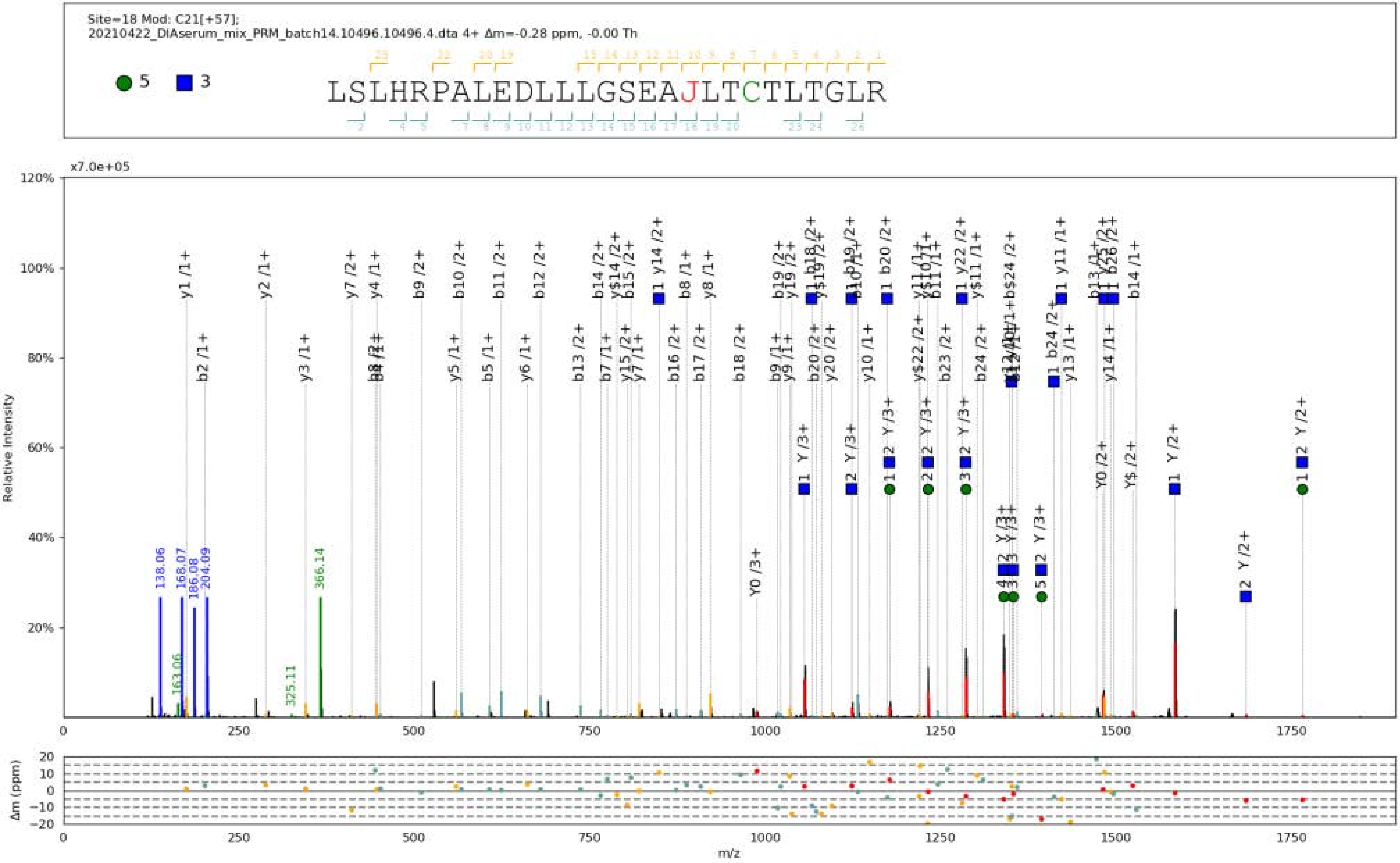
Site=18 Mod: C21[+57];
20210422_DIAserum_mix_PRM_batch14.10496.10496.4.dta 4+ Δm=-0.28 ppm, -0.00 Th
5
3
LSLHRPALEDLLLGSEAJLTCTLTGLR
x7.0e+05
Relative Intensity
Δm (ppm)
m/z
138.06
168.07
186.08
204.09
163.06
366.14
325.11

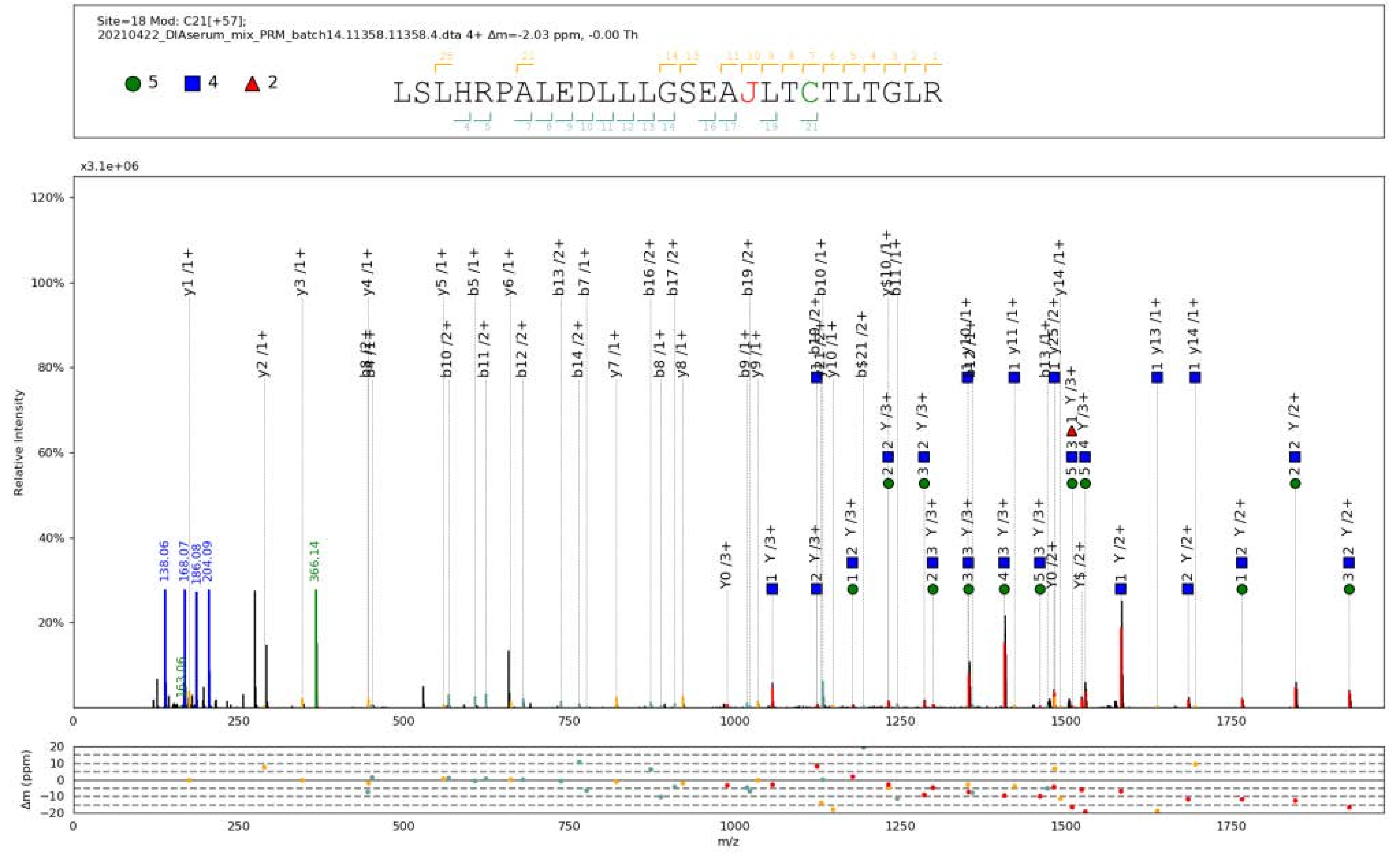

Site=18 Mod: C21[+57];
20210422_DIAserum_mix_PRM_batch14.11358.11358.4.dta 4+ Δm=-2.03 ppm, -0.00 Th
5
4
2
LSLHRPALEDLLLGSEAJLTCTLTGLR
x3.1e+06
Relative Intensity
m/z
Δm (ppm)
138.06
168.07
186.08
204.09
366.14
163.06
y1 /1+
y2 /1+
y3 /1+
y4 /1+
y5 /1+
b5 /1+
y6 /1+
b13 /2+
b7 /1+
b16 /2+
b17 /2+
b19 /2+
b10 /1+
y14 /1+
b10 /2+
b11 /2+
b12 /2+
b14 /2+
y7 /1+
b8 /1+
y8 /1+
y9 /1+
y10 /1+
b$21 /2+
y11 /1+
y13 /1+
Y0 /3+
Y /3+
Y /2+
Y0 /2+
Y$ /2+

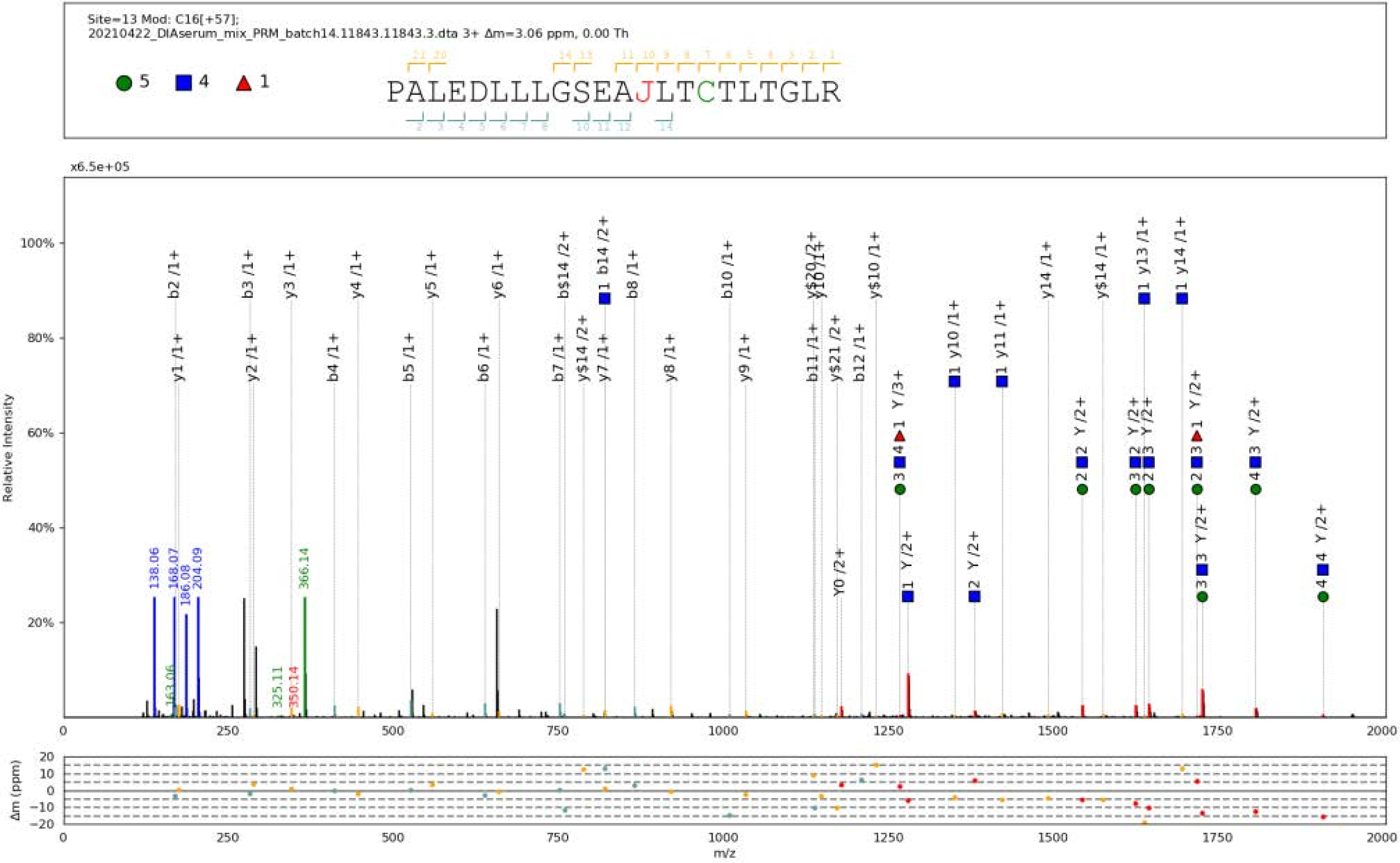

Site=13 Mod: C16[+57];
20210422_DIAserum_mix_PRM_batch14.11843.11843.3.dta 3+ Δm=3.06 ppm, 0.00 Th
PALEDLLLGSEAJLTCTLTGLR
Relative Intensity
m/z
Δm (ppm)

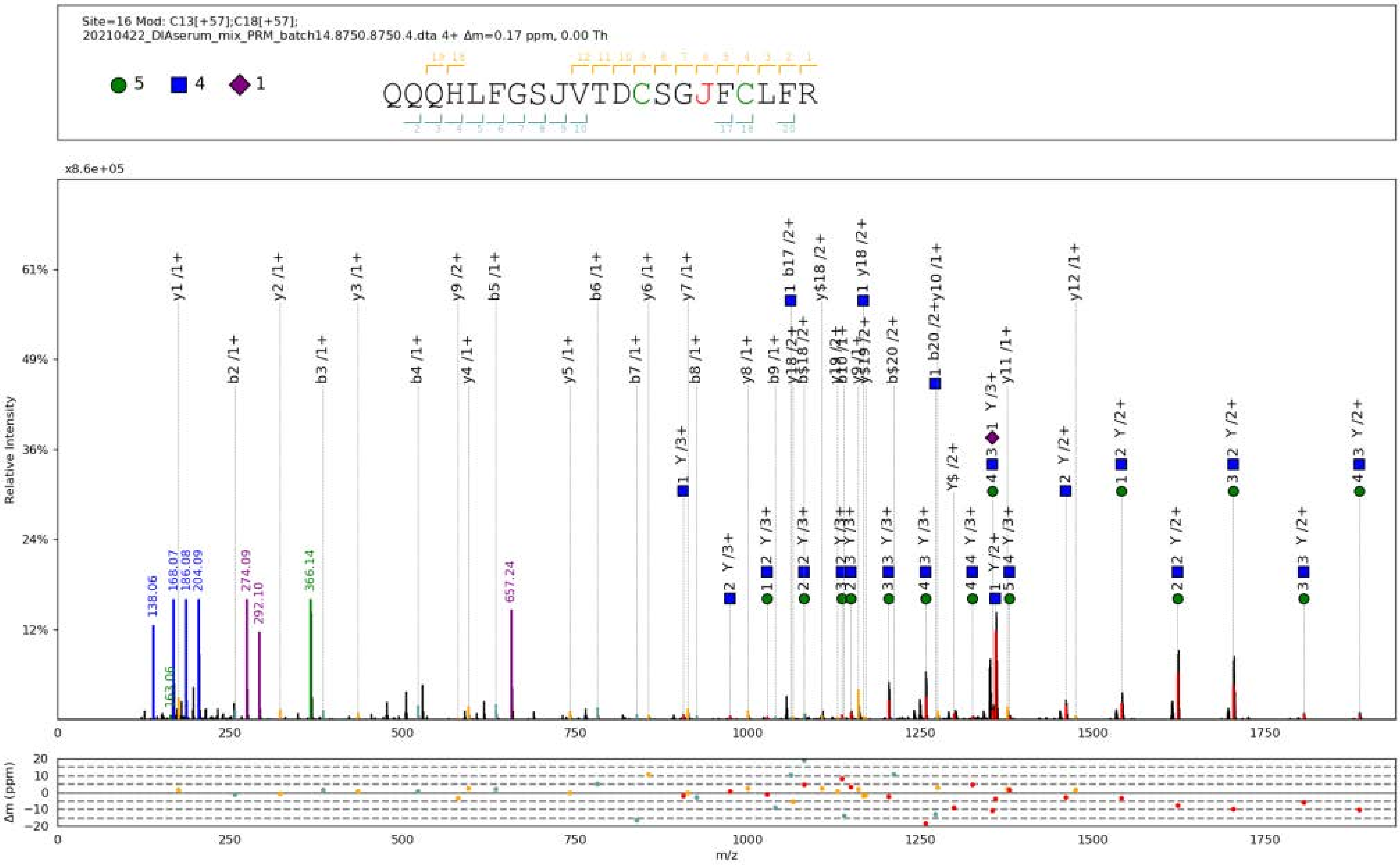

Site=16 Mod: C13[+57];C18[+57];
20210422_DIAserum_mix_PRM_batch14.8750.8750.4.dta 4+ Δm=0.17 ppm, 0.00 Th
5
4
1
QQQHLFGSJVTDCSGJFCLFR
x8.6e+05
Relative Intensity
61%
49%
36%
24%
12%
138.06
168.07
186.08
204.09
163.06
274.09
292.10
366.14
657.24
y1 /1+
b2 /1+
y2 /1+
b3 /1+
y3 /1+
b4 /1+
y9 /2+
y4 /1+
b5 /1+
y5 /1+
b6 /1+
b7 /1+
y6 /1+
y7 /1+
b8 /1+
y8 /1+
b9 /1+
1 b17 /2+
y$18 /2+
1 y18 /2+
1 b20 /2+y10 /1+
Y$ /2+
y11 /1+
y12 /1+
Y /3+
Y /2+
0
250
500
750
1000
1250
1500
1750
Δm (ppm)
20
10
0
−10
−20
m/z

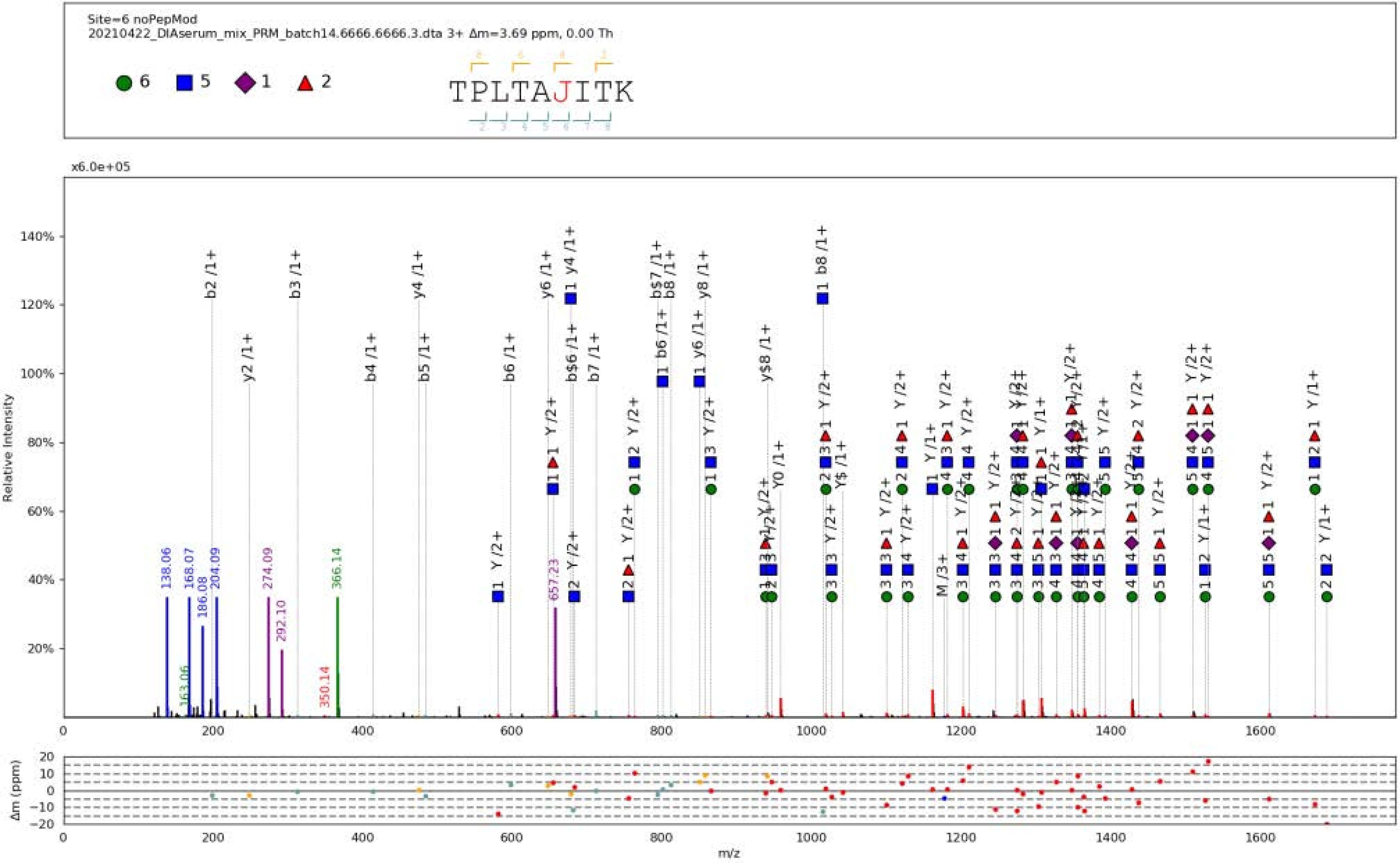

Site=6 noPepMod
20210422_DIAserum_mix_PRM_batch14.6666.6666.3.dta 3+ Δm=3.69 ppm, 0.00 Th
6
5
1
2
TPLTAJITK
x6.0e+05
Relative Intensity
m/z
Δm (ppm)
b2 /1+
y2 /1+
b3 /1+
b4 /1+
y4 /1+
b5 /1+
b6 /1+
y6 /1+
y4 /1+
b$6 /1+
b7 /1+
b$7 /1+
b8 /1+
b6 /1+
y6 /1+
y8 /1+
y$8 /1+
Y0 /1+
b8 /1+
Y$ /1+
M /3+
138.06
168.07
186.08
204.09
163.06
274.09
292.10
350.14
366.14
657.23

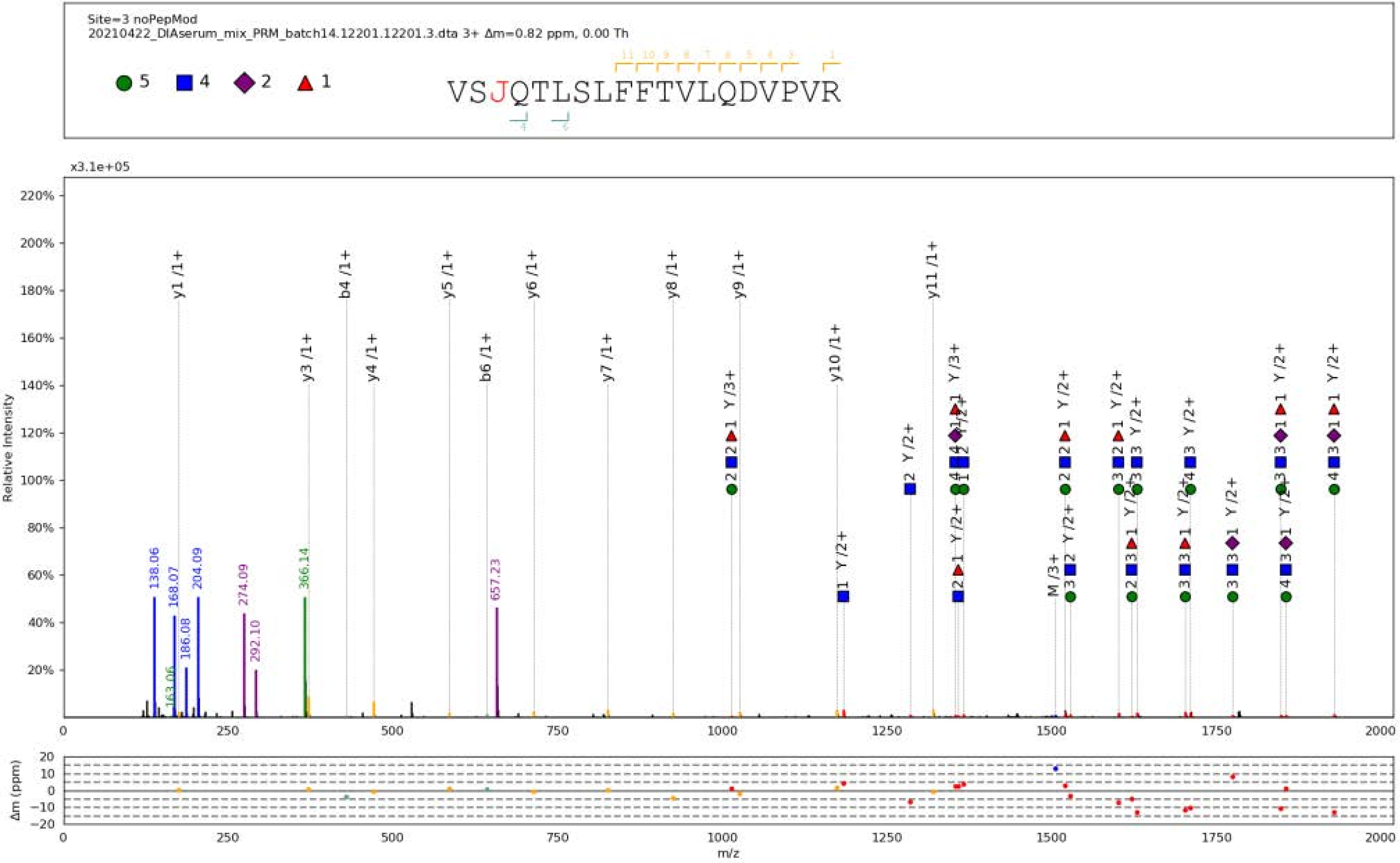

Site=3 noPepMod
20210422_DIAserum_mix_PRM_batch14.12201.12201.3.dta 3+ Δm=0.82 ppm, 0.00 Th
5
4
2
1
VSJQTLSLFFTVLQDVPVR
x3.1e+05
Relative Intensity
Δm (ppm)
m/z

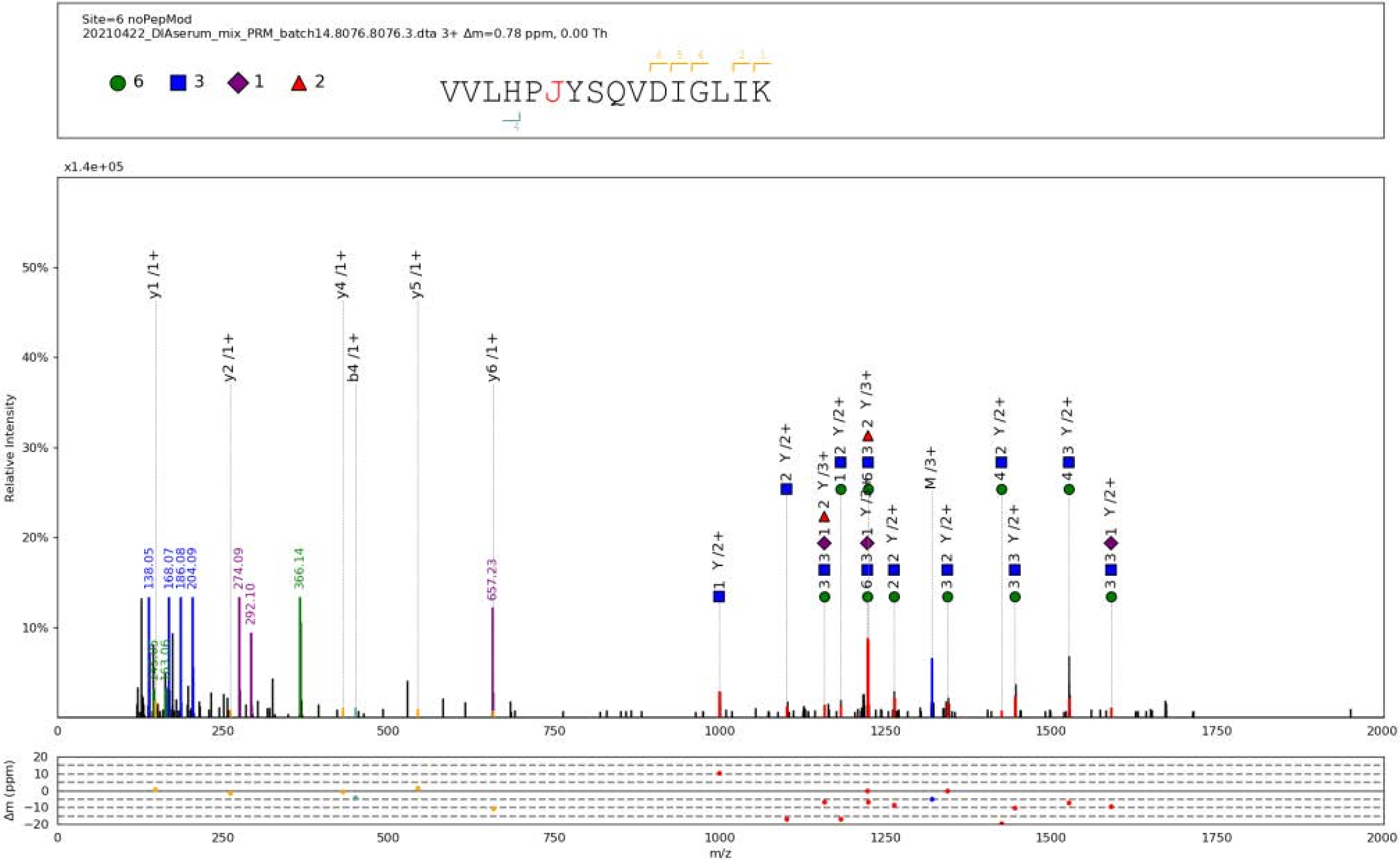
Site=6 noPepMod
20210422_DIAserum_mix_PRM_batch14.8076.8076.3.dta 3+ Δm=0.78 ppm, 0.00 Th
6 3 1 2
VVLHPJYSQVDIGLIK
x1.4e+05
Relative Intensity
y1 /1+
y2 /1+
y4 /1+
b4 /1+
y5 /1+
y6 /1+
138.05
168.07
186.08
204.09
274.09
292.10
366.14
657.23
M /3+
Δm (ppm)
m/z

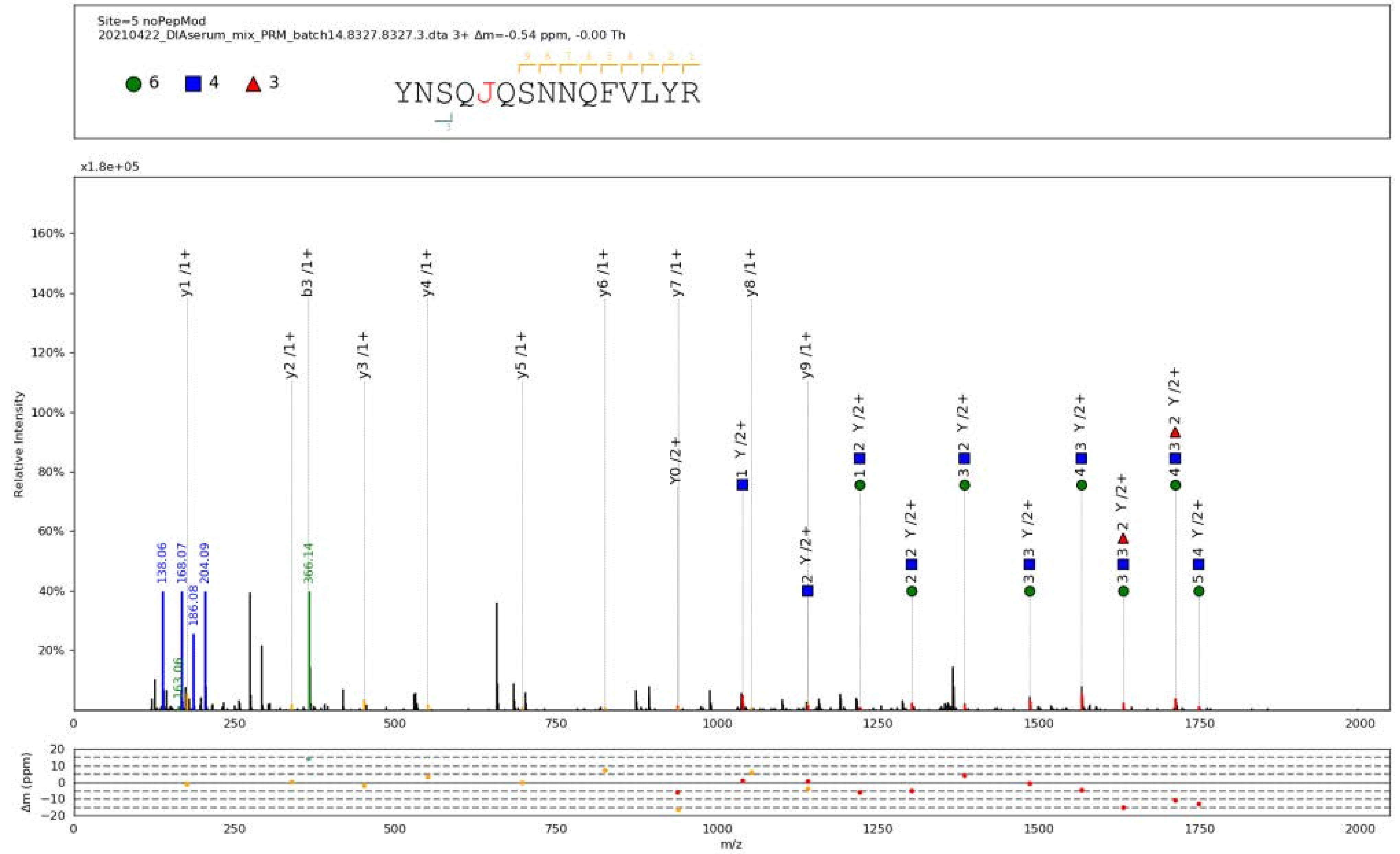
Site=5 noPepMod
20210422_DIAserum_mix_PRM_batch14.8327.8327.3.dta 3+ Δm=-0.54 ppm, -0.00 Th
6
4
3
YNSQJQSNNQFVLYR
x1.8e+05
Relative Intensity
m/z
Δm (ppm)
y1 /1+
b3 /1+
y2 /1+
y3 /1+
y4 /1+
y5 /1+
y6 /1+
y7 /1+
y8 /1+
y9 /1+
Y0 /2+
138.06
168.07
186.08
204.09
366.14
163.06

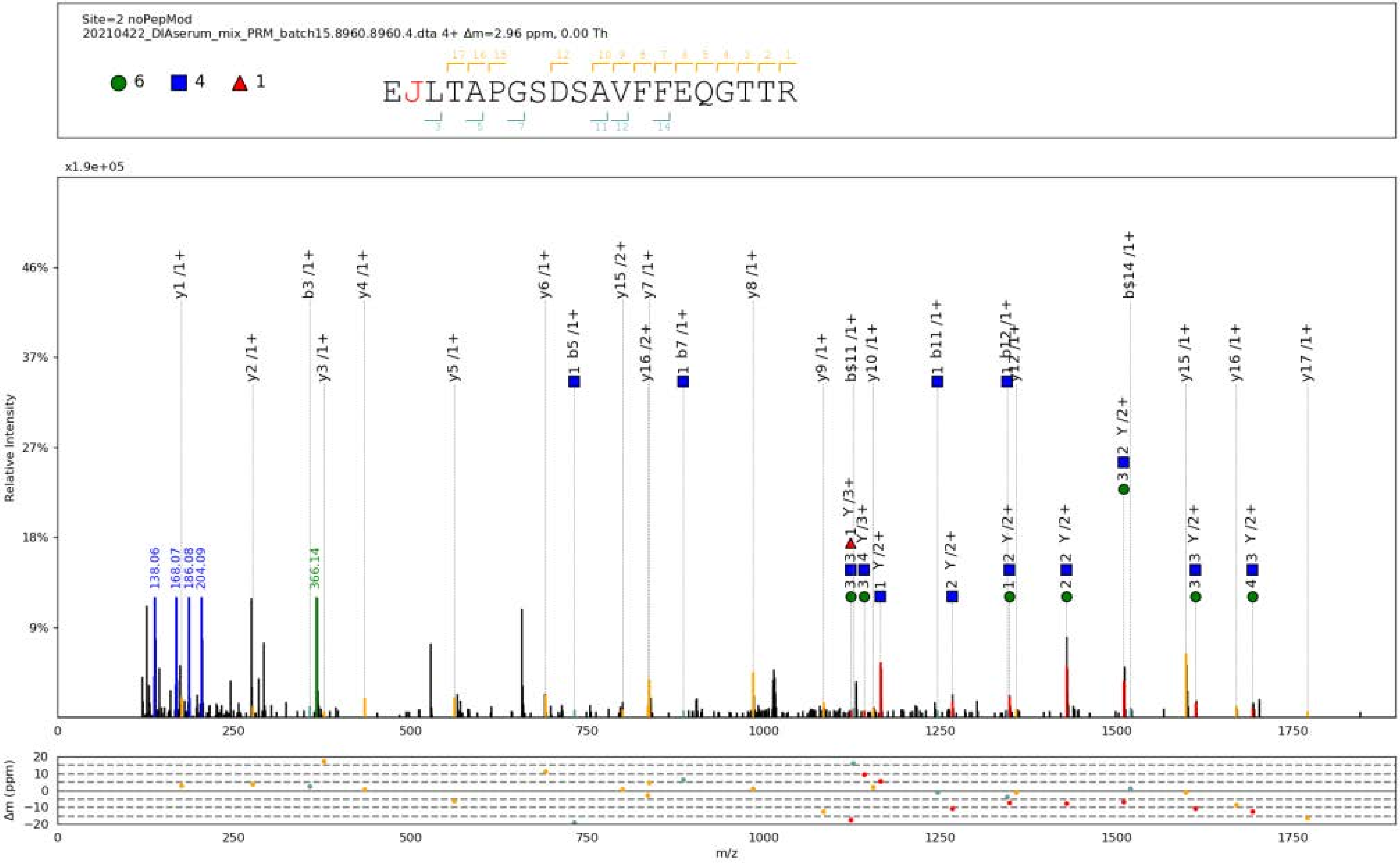

Site=2 noPepMod
20210422_DIAserum_mix_PRM_batch15.8960.8960.4.dta 4+ Δm=2.96 ppm, 0.00 Th
6
4
1
EJLTAPGSDSAVFFEQGTTR
x1.9e+05
Relative Intensity
m/z
Δm (ppm)

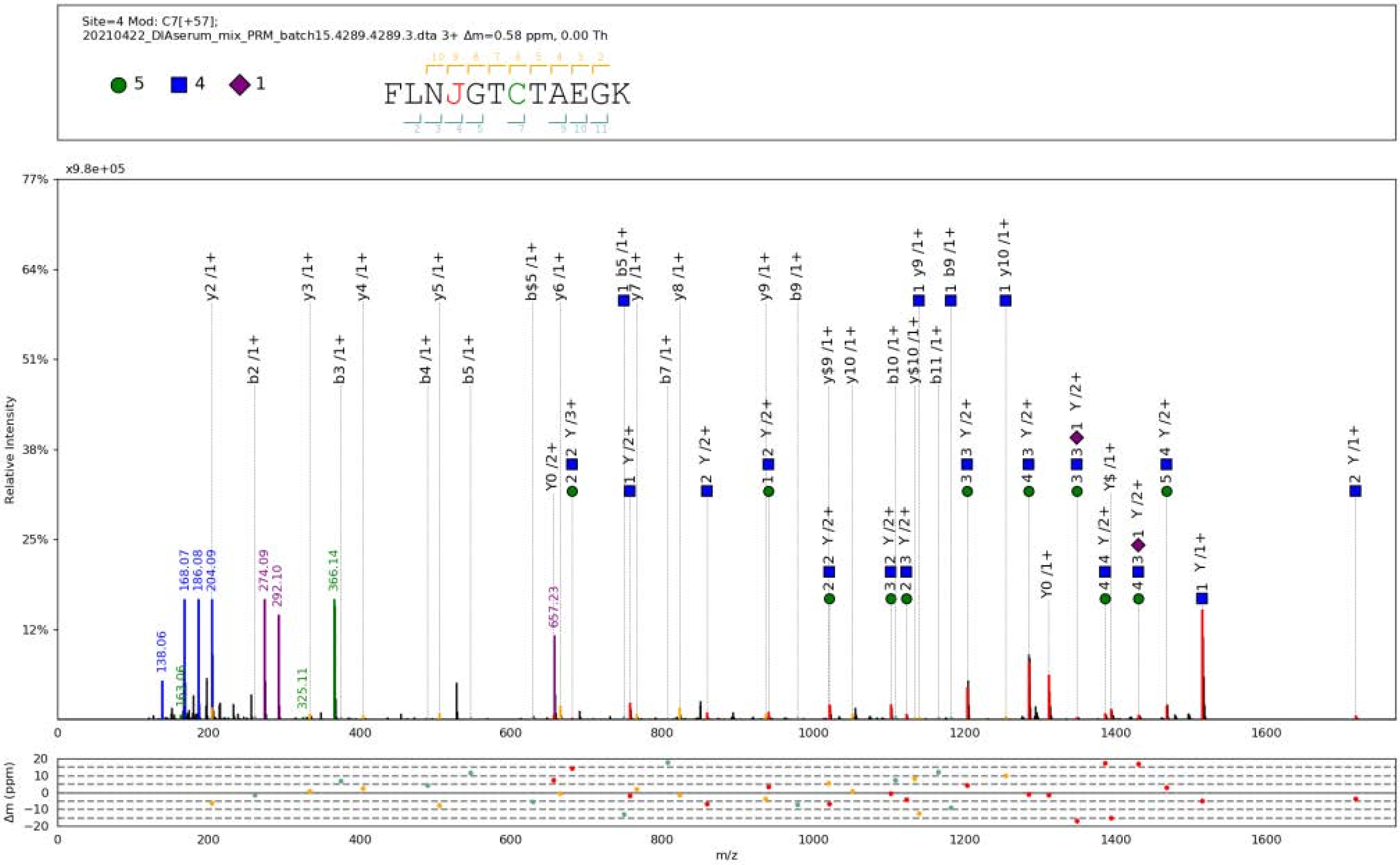

Site=4 Mod: C7[+57];
20210422_DIAserum_mix_PRM_batch15.4289.4289.3.dta 3+ Δm=0.58 ppm, 0.00 Th
5
4
1
FLNJGTCTAEGK
x9.8e+05
Relative Intensity
y2 /1+
b2 /1+
y3 /1+
b3 /1+
y4 /1+
b4 /1+
y5 /1+
b5 /1+
b$5 /1+
y6 /1+
Y0 /2+
Y /3+
1 b5 /1+
y7 /1+
Y /2+
b7 /1+
y8 /1+
y9 /1+
b9 /1+
y$9 /1+
y10 /1+
b10 /1+
y$10 /1+
1 y9 /1+
b11 /1+
1 b9 /1+
1 y10 /1+
Y0 /1+
Y$ /1+
Y /1+
138.06
163.06
168.07
186.08
204.09
274.09
292.10
325.11
366.14
657.23
m/z
Δm (ppm)

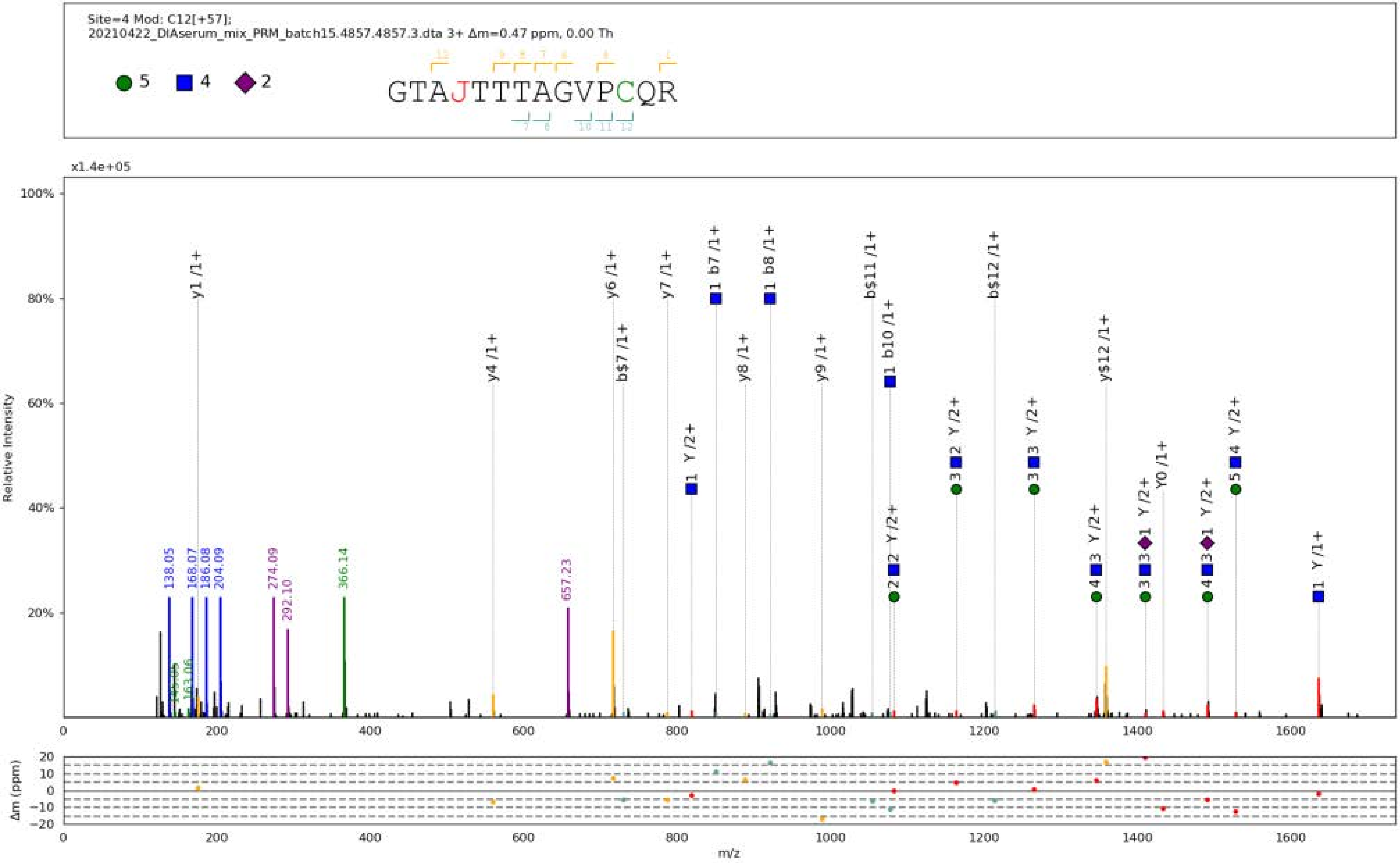

Site=4 Mod: C12[+57];
20210422_DIAserum_mix_PRM_batch15.4857.4857.3.dta 3+ Δm=0.47 ppm, 0.00 Th
5
4
2
GTAJTTTAGVPCQR
x1.4e+05
Relative Intensity
y1 /1+
y4 /1+
y6 /1+
b$7 /1+
y7 /1+
1 Y /2+
1 b7 /1+
y8 /1+
1 b8 /1+
y9 /1+
b$11 /1+
1 b10 /1+
2 2 Y /2+
3 2 Y /2+
b$12 /1+
3 3 Y /2+
4 3 Y /2+
y$12 /1+
3 3 1 Y /2+
Y0 /1+
4 3 1 Y /2+
5 4 Y /2+
1 Y /1+
138.05
168.07
186.08
204.09
274.09
292.10
366.14
657.23
163.06
Δm (ppm)
m/z

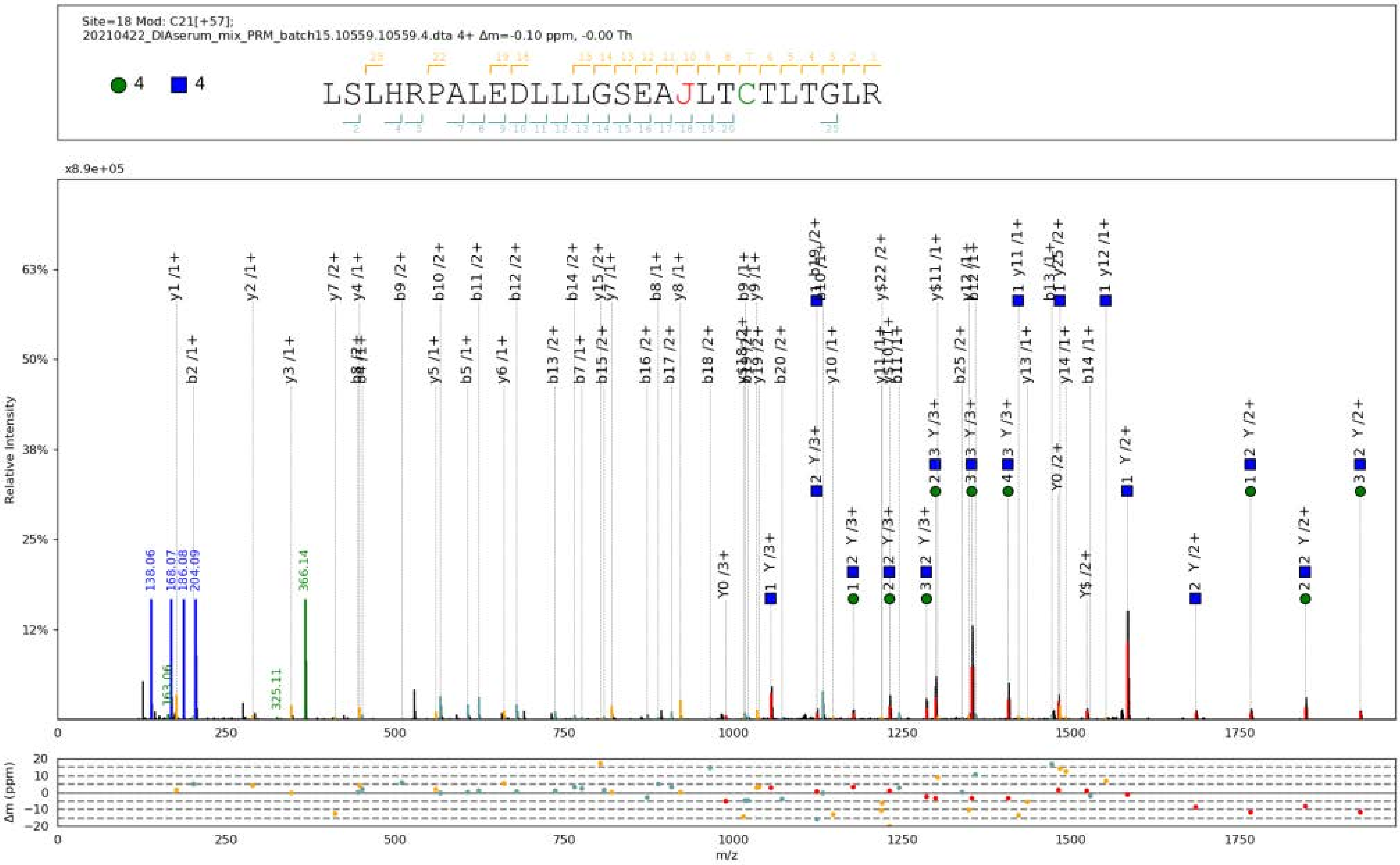

Site=18 Mod: C21[+57];
20210422_DIAserum_mix_PRM_batch15.10559.10559.4.dta 4+ Δm=-0.10 ppm, -0.00 Th
LSLHRPALEDLLLGSEAJLTCTLTGLR
x8.9e+05
Relative Intensity
Δm (ppm)
m/z

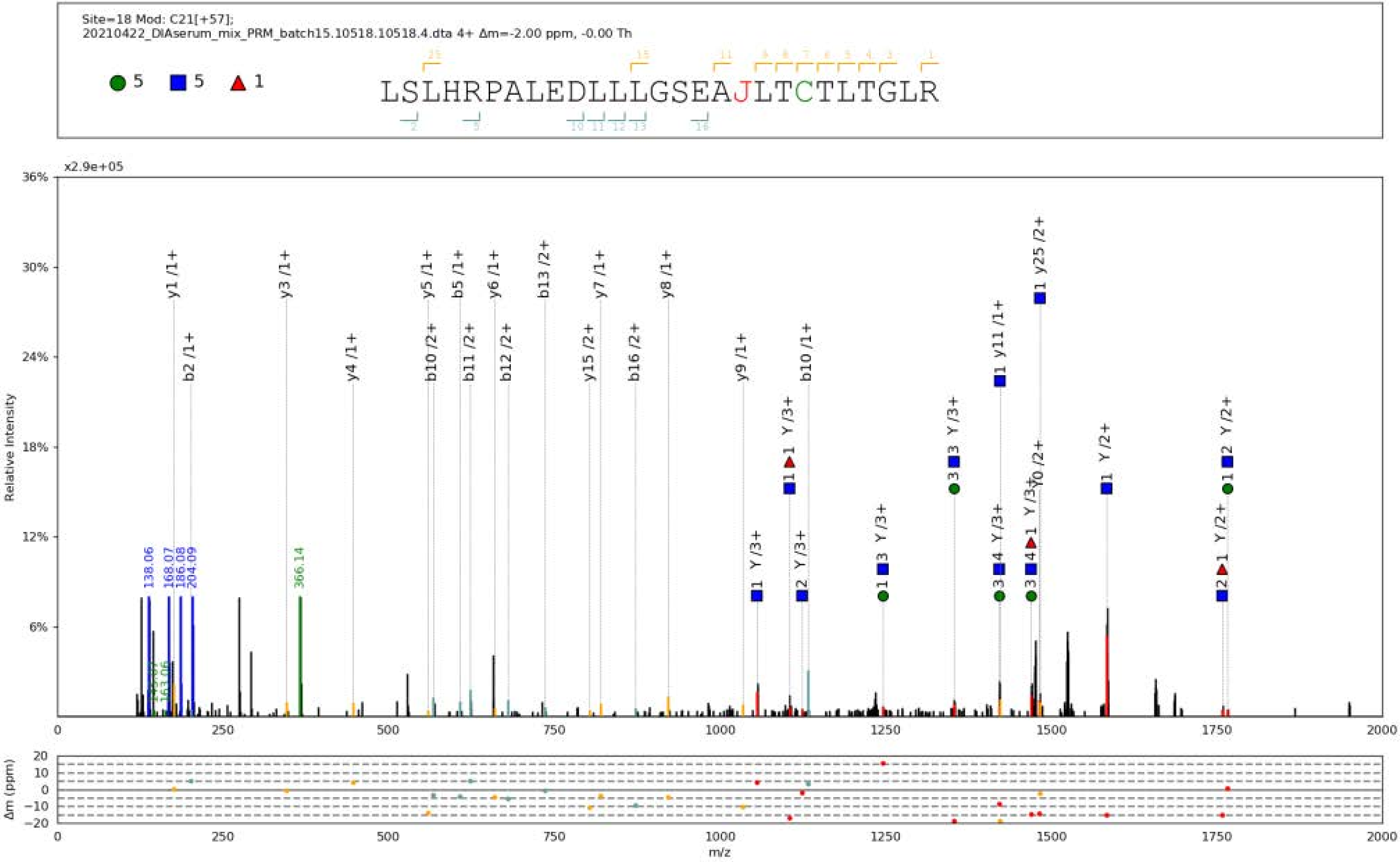

Site=18 Mod: C21[+57];
20210422_DIAserum_mix_PRM_batch15.10518.10518.4.dta 4+ Δm=-2.00 ppm, -0.00 Th
LSLHRPALEDLLLGSEAJLTCTLTGLR
x2.9e+05
Relative Intensity
m/z
Δm (ppm)

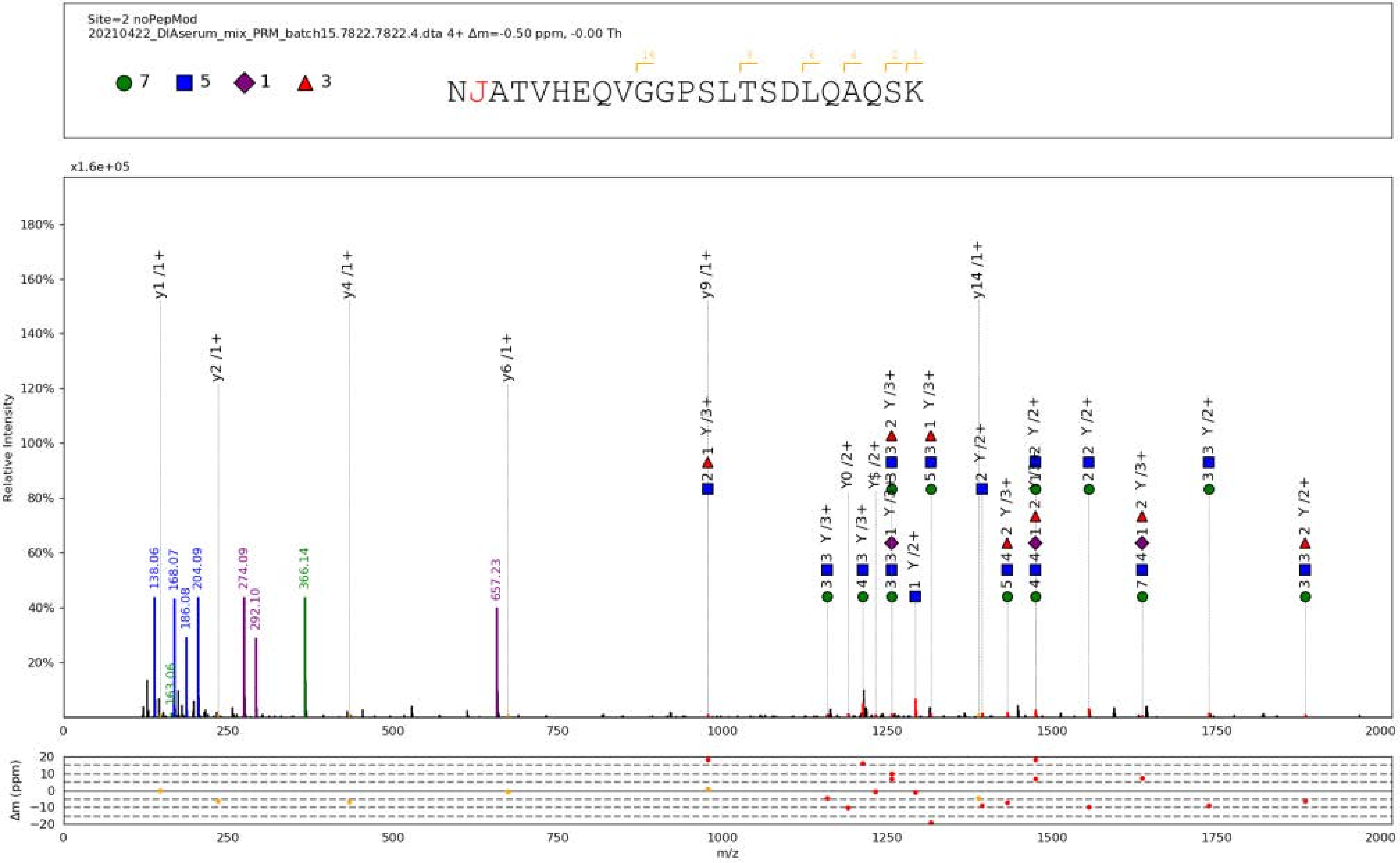
Site=2 noPepMod
20210422_DIAserum_mix_PRM_batch15.7822.7822.4.dta 4+ Δm=-0.50 ppm, -0.00 Th
7
5
1
3
NJATVHEQVGGPSLTSDLQAQSK
x1.6e+05
Relative Intensity
y1 /1+
y2 /1+
y4 /1+
y6 /1+
y9 /1+
y14 /1+
Y0 /2+
Y$ /2+
138.06
168.07
186.08
204.09
163.06
274.09
292.10
366.14
657.23
m/z
Δm (ppm)

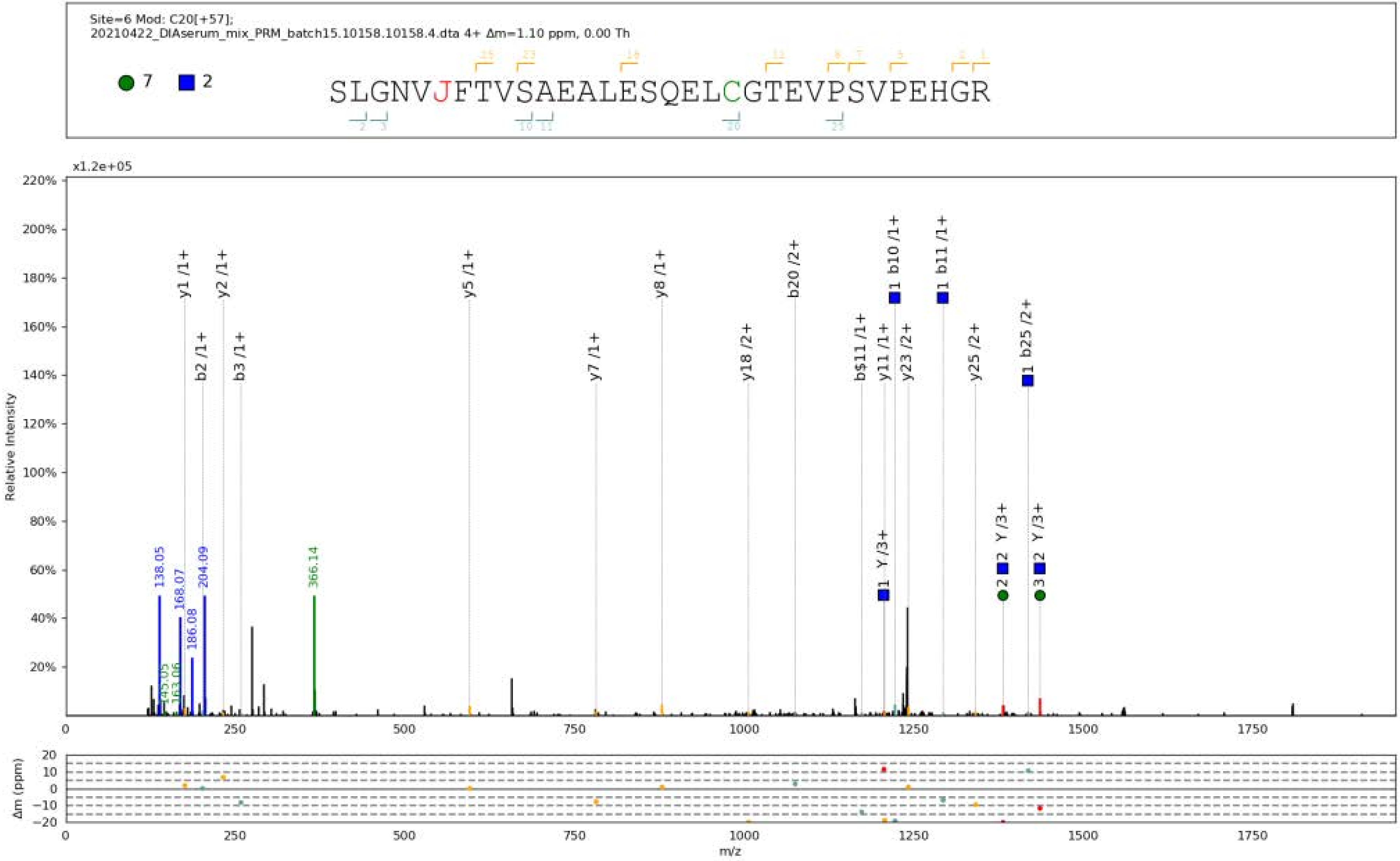
Site=6 Mod: C20[+57];
20210422_DIAserum_mix_PRM_batch15.10158.10158.4.dta 4+ Δm=1.10 ppm, 0.00 Th
SLGNVJFTVSAEALESQELCGTEVPSVPEHGR
x1.2e+05
Relative Intensity
y1 /1+
y2 /1+
b2 /1+
b3 /1+
y5 /1+
y7 /1+
y8 /1+
y18 /2+
b20 /2+
b$11 /1+
y11 /1+
y23 /2+
1 b10 /1+
1 b11 /1+
y25 /2+
1 b25 /2+
1 Y /3+
2 2 Y /3+
3 2 Y /3+
138.05
168.07
186.08
204.09
366.14
m/z
Δm (ppm)

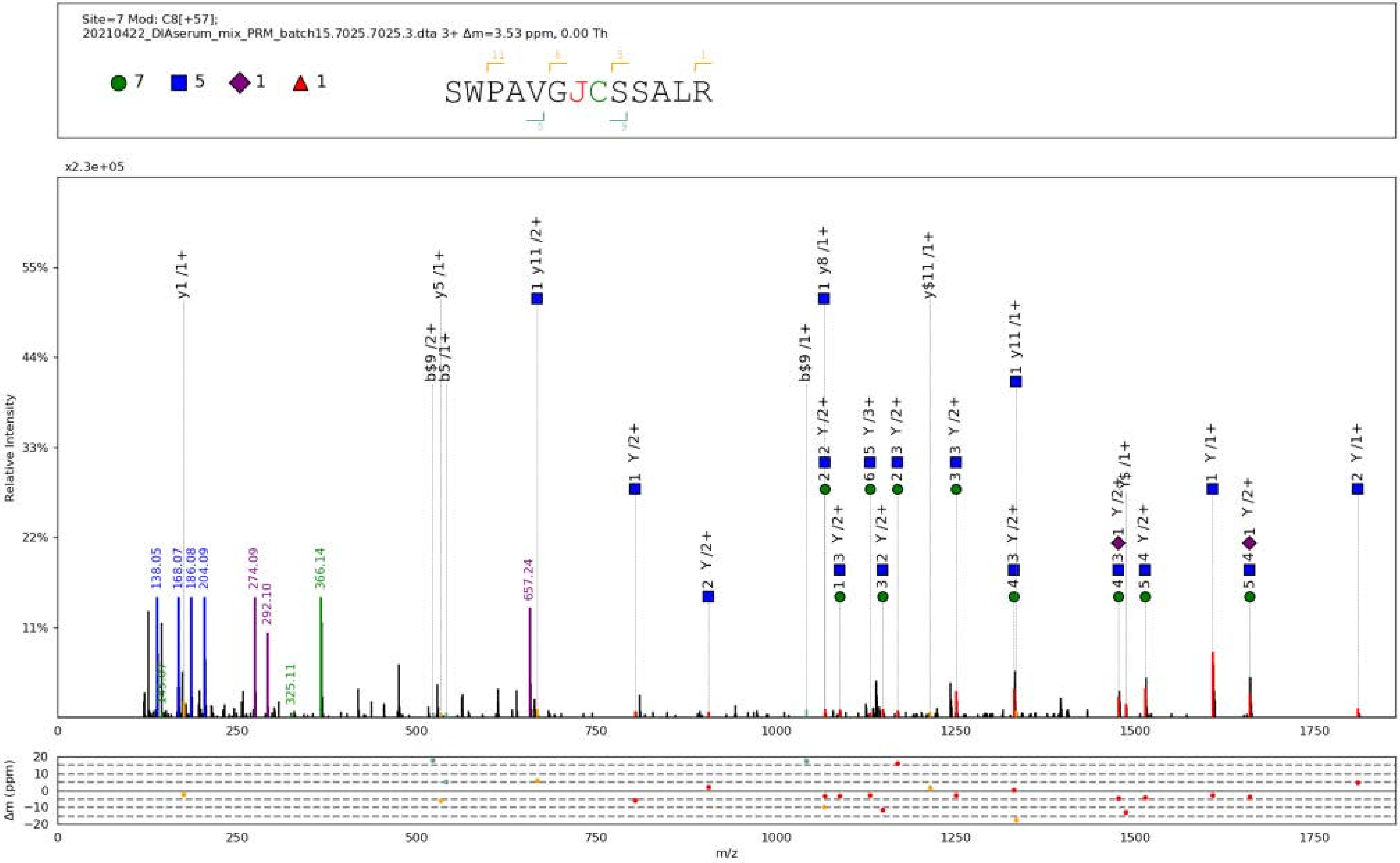

Site=7 Mod: C8[+57];
20210422_DIAserum_mix_PRM_batch15.7025.7025.3.dta 3+ Δm=3.53 ppm, 0.00 Th
7 5 1 1
SWPAVGJCSSALR
x2.3e+05
Relative Intensity
m/z
Δm (ppm)

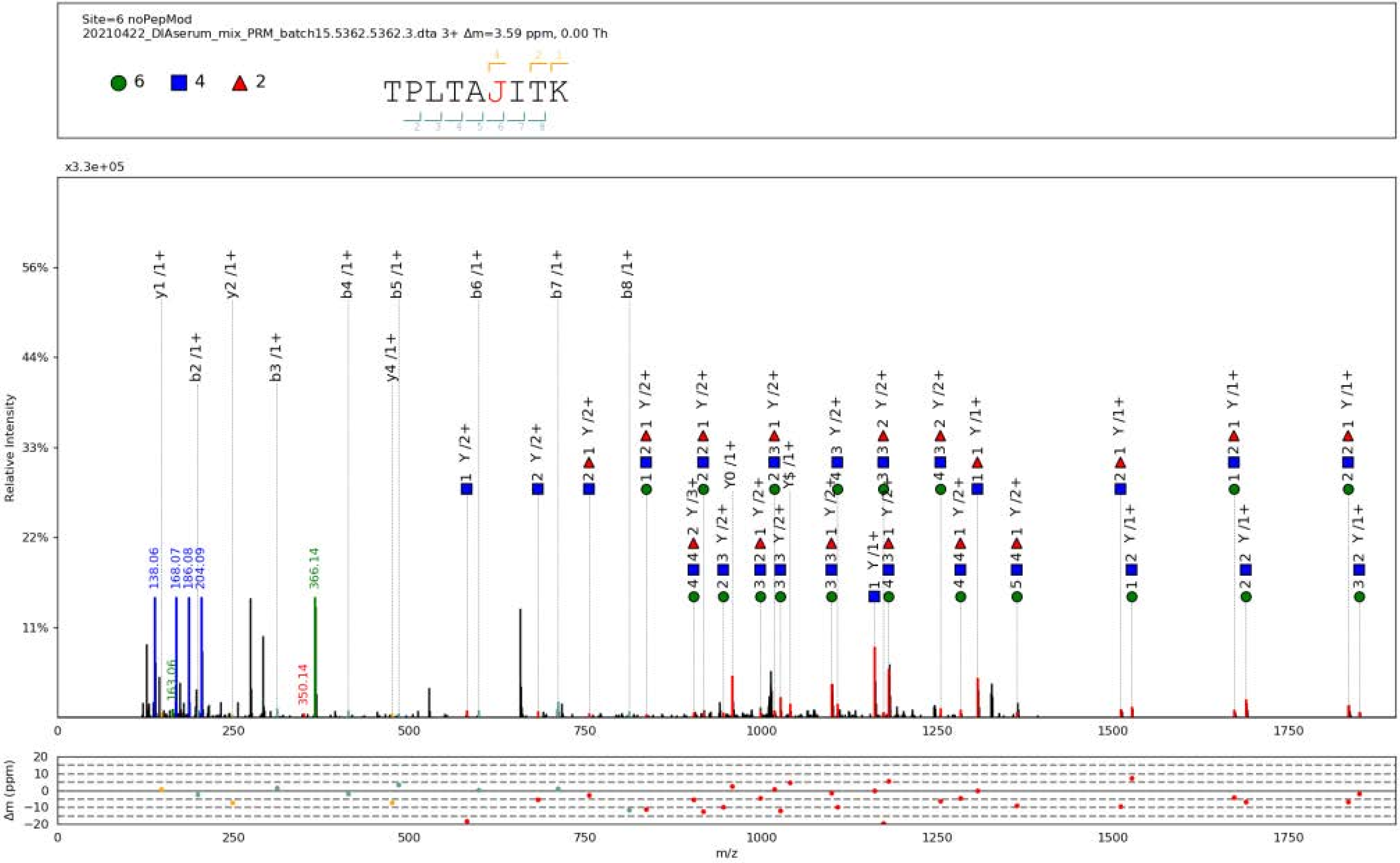

Site=6 noPepMod
20210422_DIAserum_mix_PRM_batch15.5362.5362.3.dta 3+ Δm=3.59 ppm, 0.00 Th
6 4 2
TPLTAJITK
x3.3e+05
Relative Intensity
56%
44%
33%
22%
11%
y1 /1+
b2 /1+
y2 /1+
b3 /1+
b4 /1+
y4 /1+
b5 /1+
b6 /1+
b7 /1+
b8 /1+
Y0 /1+
Y$ /1+
138.06
168.07
186.08
204.09
163.06
366.14
350.14
0
250
500
750
1000
1250
1500
1750
Δm (ppm)
m/z

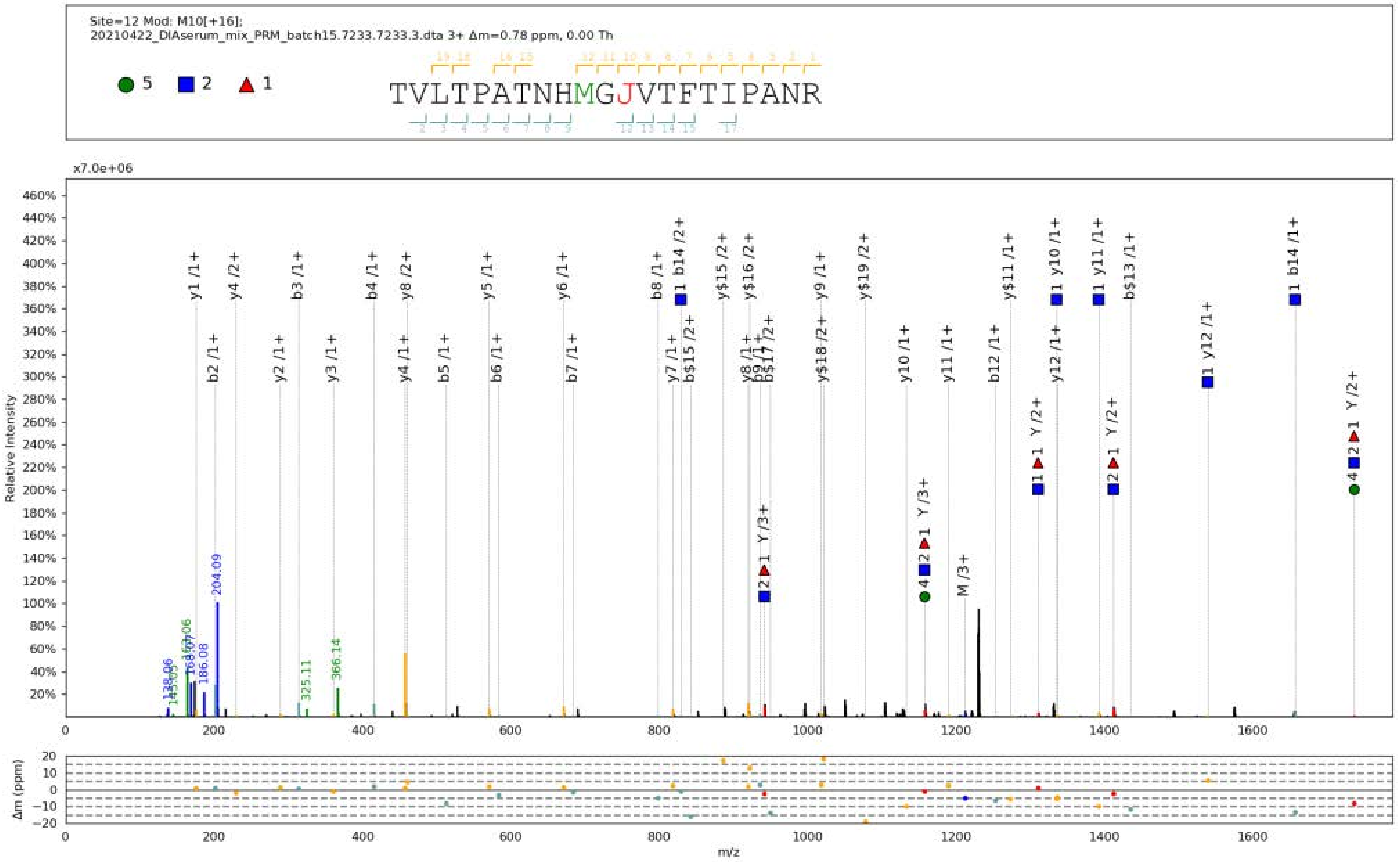

Site=12 Mod: M10[+16];
20210422_DIAserum_mix_PRM_batch15.7233.7233.3.dta 3+ Δm=0.78 ppm, 0.00 Th
5
2
1
TVLTPATNHMGJVTFTIPANR
x7.0e+06
Relative Intensity
Δm (ppm)
m/z

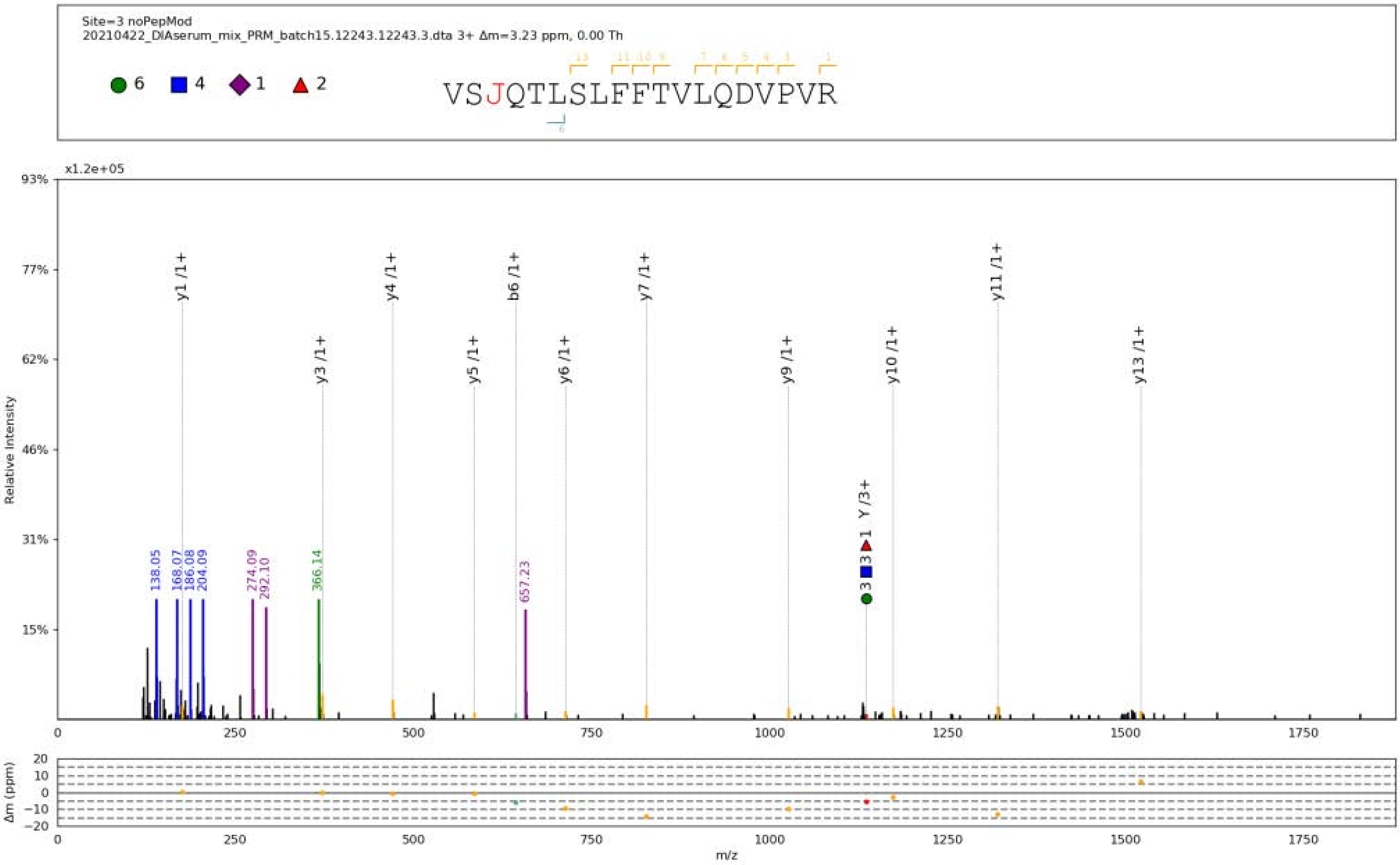
Site=3 noPepMod
20210422_DIAserum_mix_PRM_batch15.12243.12243.3.dta 3+ Δm=3.23 ppm, 0.00 Th
6
4
1
2
VSJQTLSLFFTVLQDVPVR
x1.2e+05
Relative Intensity
y1 /1+
y3 /1+
y4 /1+
y5 /1+
b6 /1+
y6 /1+
y7 /1+
y9 /1+
Y /3+
y10 /1+
y11 /1+
y13 /1+
138.05
168.07
186.08
204.09
274.09
292.10
366.14
657.23
m/z
Δm (ppm)

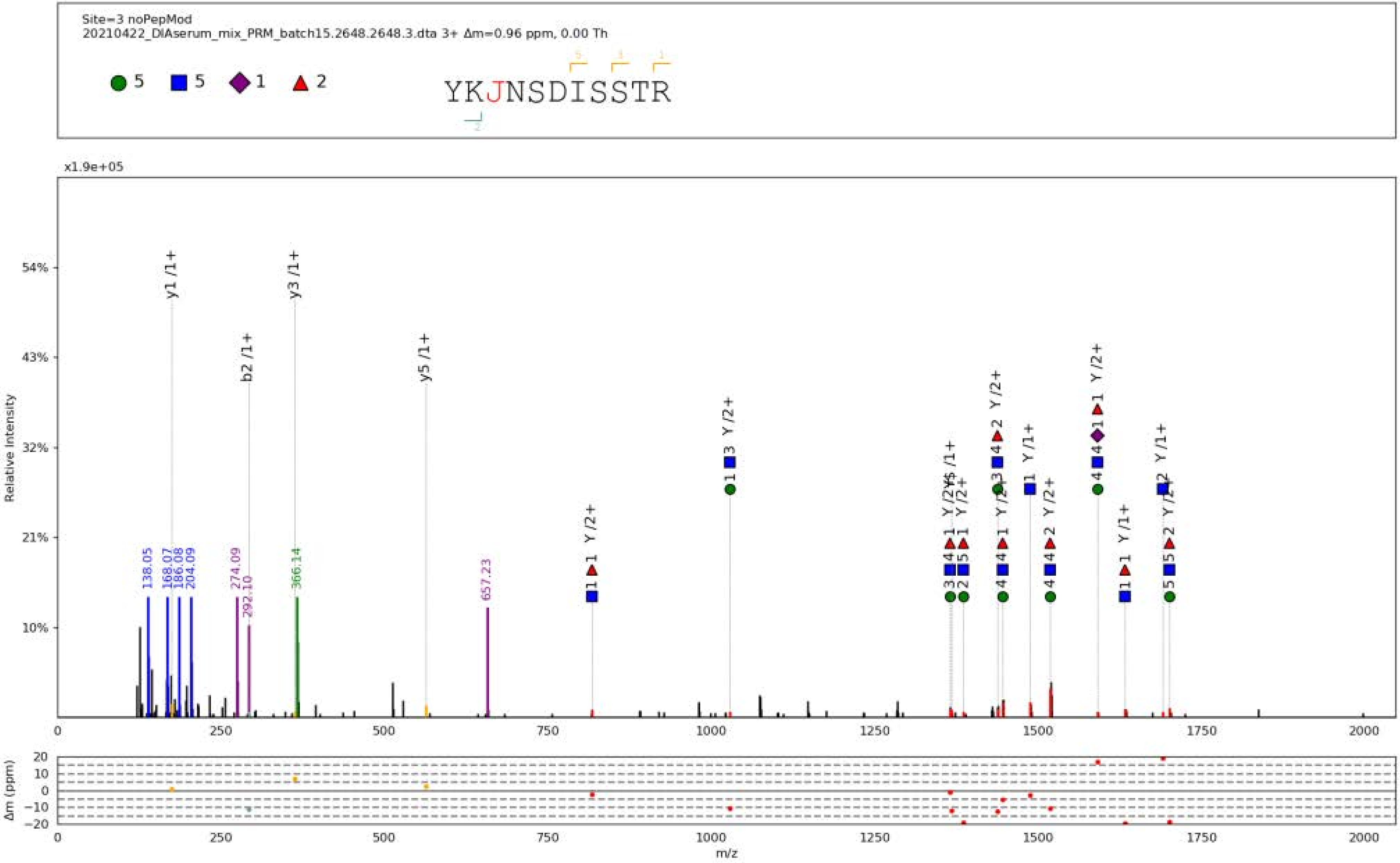
Site=3 noPepMod
20210422_DIAserum_mix_PRM_batch15.2648.2648.3.dta 3+ Δm=0.96 ppm, 0.00 Th
5 5 1 2
YKJNSDISSTR
x1.9e+05
Relative Intensity
54%
43%
32%
21%
10%
y1 /1+
y3 /1+
b2 /1+
y5 /1+
138.05
168.07
186.08
204.09
274.09
292.10
366.14
657.23
0
250
500
750
1000
1250
1500
1750
2000
Δm (ppm)
m/z

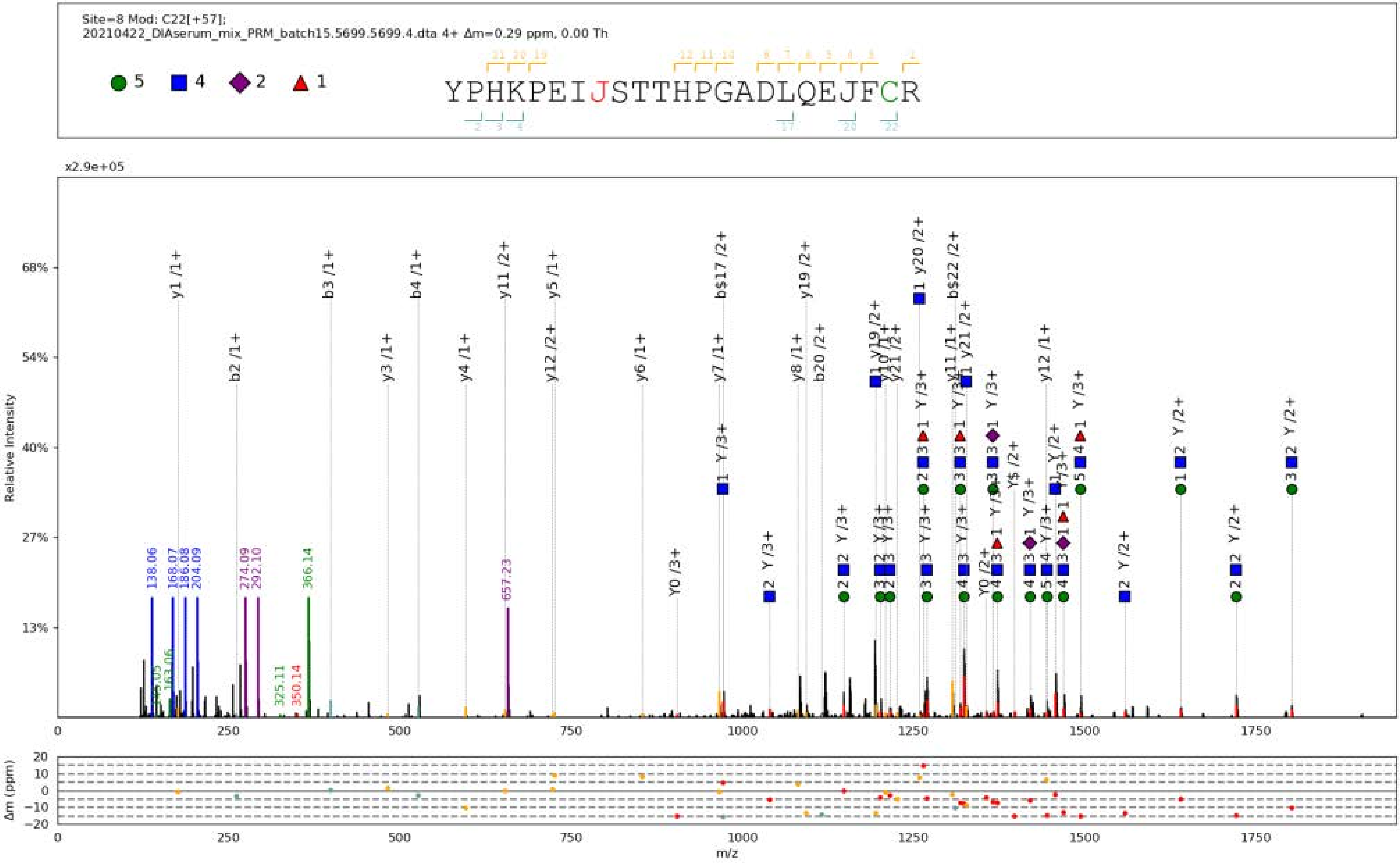
Site=8 Mod: C22[+57];
20210422_DIAserum_mix_PRM_batch15.5699.5699.4.dta 4+ Δm=0.29 ppm, 0.00 Th
5 4 2 1
YPHKPEIJSTTHPGADLQEJFCR
x2.9e+05
Relative Intensity
68%
54%
40%
27%
13%
y1 /1+
b2 /1+
b3 /1+
y3 /1+
b4 /1+
y4 /1+
y11 /2+
y5 /1+
y12 /2+
y6 /1+
Y0 /3+
b$17 /2+
y7 /1+
y8 /1+
y19 /2+
b20 /2+
y20 /2+
b$22 /2+
y21 /2+
y12 /1+
138.06
168.07
186.08
204.09
274.09
292.10
366.14
657.23
325.11
350.14
163.06
0
250
500
750
1000
1250
1500
1750
Δm (ppm)
20
10
0
−10
−20
m/z

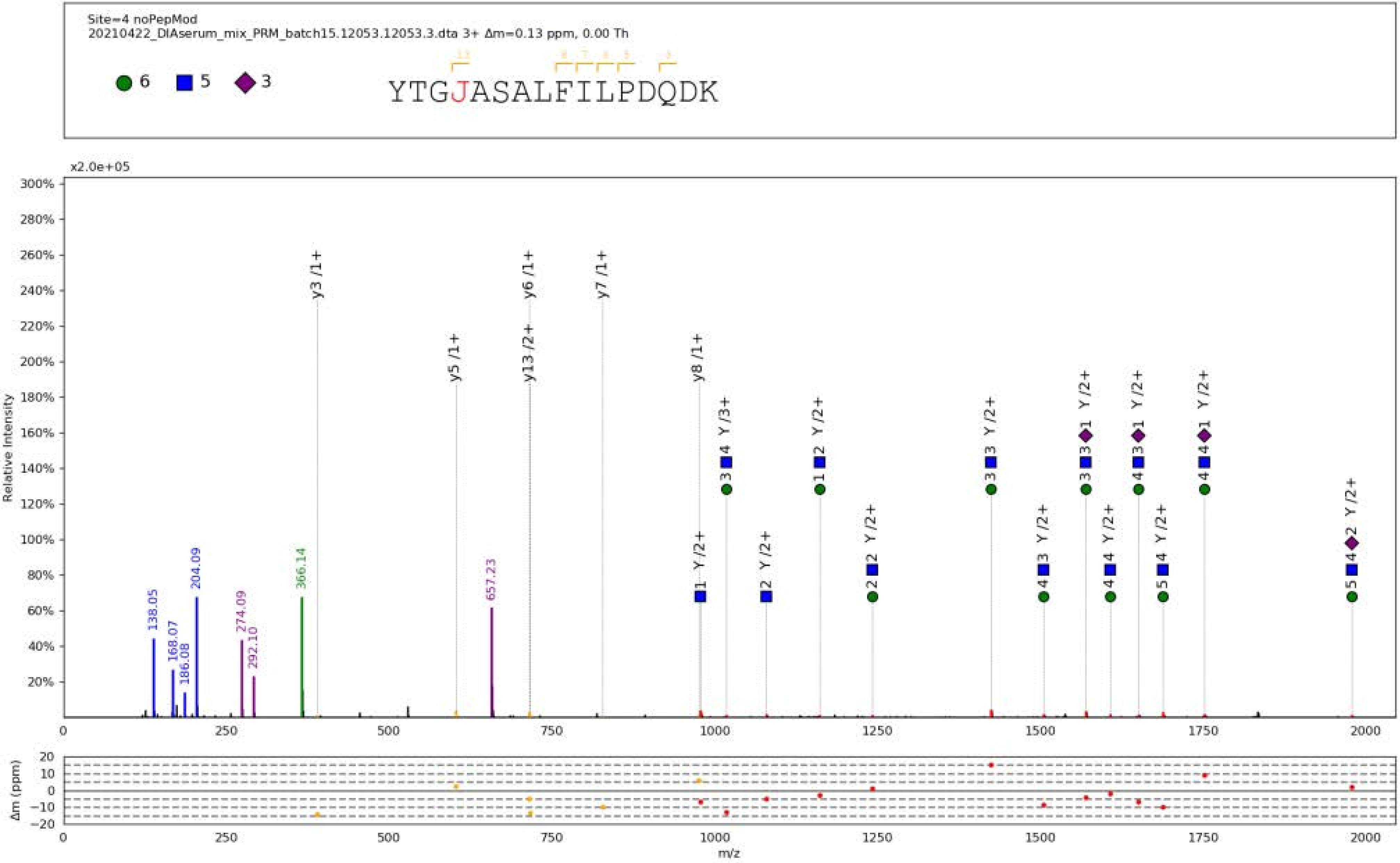
Site=4 noPepMod
20210422_DIAserum_mix_PRM_batch15.12053.12053.3.dta 3+ Δm=0.13 ppm, 0.00 Th
6
5
3
YTGJASALFILPDQDK
x2.0e+05
Relative Intensity
y3 /1+
y6 /1+
y7 /1+
y5 /1+
y13 /2+
y8 /1+
138.05
168.07
186.08
204.09
274.09
292.10
366.14
657.23
m/z
Δm (ppm)
