# Supplementary material for: GproDIA enables data-independent acquisition glycoproteomics with comprehensive statistical control
Source: Nat Commun. 2021 Oct 18;12:6073. doi: 10.1038/s41467-021-26246-3 (PMC8523693; doi:10.1038/s41467-021-26246-3)
Supplement: Supplementary file 23 — Reporting Summary [file 41467_2021_26246_MOESM23_ESM.pdf]

nature research

Corresponding author(s): Liang Qiao, Weiqian Cao

Last updated by author(s): Sep 10, 2021

Reporting Summary

Nature Research wishes to improve the reproducibility of the work that we publish. This form provides structure for consistency and transparency in reporting. For further information on Nature Research policies, see our Editorial Policies and the Editorial Policy Checklist.

Statistics

For all statistical analyses, confirm that the following items are present in the figure legend, table legend, main text, or Methods section.

n/a	Confirmed	
☐	☒	The exact sample size (n) for each experimental group/condition, given as a discrete number and unit of measurement
☐	☒	A statement on whether measurements were taken from distinct samples or whether the same sample was measured repeatedly
☒	☐	The statistical test(s) used AND whether they are one- or two-sided<br>*Only common tests should be described solely by name; describe more complex techniques in the Methods section.*
☒	☐	A description of all covariates tested
☒	☐	A description of any assumptions or corrections, such as tests of normality and adjustment for multiple comparisons
☐	☒	A full description of the statistical parameters including central tendency (e.g. means) or other basic estimates (e.g. regression coefficient) AND variation (e.g. standard deviation) or associated estimates of uncertainty (e.g. confidence intervals)
☒	☐	For null hypothesis testing, the test statistic (e.g. F, t, r) with confidence intervals, effect sizes, degrees of freedom and P value noted<br>*Give P values as exact values whenever suitable.*
☒	☐	For Bayesian analysis, information on the choice of priors and Markov chain Monte Carlo settings
☒	☐	For hierarchical and complex designs, identification of the appropriate level for tests and full reporting of outcomes
☐	☒	Estimates of effect sizes (e.g. Cohen's d, Pearson's r), indicating how they were calculated

Our web collection on statistics for biologists contains articles on many of the points above.

Software and code

Policy information about availability of computer code

Data collection	Data collection was performed by Thermo Xcalibur (version 3.0.63).
Data analysis	DDA and targeted MS/MS data were analyzed by pGlyco3 (version 3.0.rc1). The raw DIA data files were converted to mzML format using MSConvert from ProteoWizard (version 3.0.11537). DIA mzML data were analyzed by OpenSWATH (version 2.6.0), PyProphet (version 2.1.5), TRIC from msproteomicstools (version 0.11.0), and our software GproDIA. GproDIA was implemented in Python (version 3.5.6, Anaconda distribution version 4.2.0), and source code has been deposited on GitHub at https://github.com/lmsac/GproDIA. Post-analysis statistics was conducted using R (version 3.5.1, Microsoft R Open). The Python package “matplotlib” (version 3.0.3), as well as the R packages “ggplot2” (version 3.0.0) and “VennDiagram” (version 1.6.20) were used for data visualization.

For manuscripts utilizing custom algorithms or software that are central to the research but not yet described in published literature, software must be made available to editors and reviewers. We strongly encourage code deposition in a community repository (e.g. GitHub). See the Nature Research guidelines for submitting code & software for further information.

Data

Policy information about availability of data

All manuscripts must include a data availability statement. This statement should provide the following information, where applicable:

- Accession codes, unique identifiers, or web links for publicly available datasets
- A list of figures that have associated raw data
- A description of any restrictions on data availability

All raw mass spectrometry data, spectral libraries and search results have been deposited to ProteomeXchange via the iProX partner repository with the dataset identifiers PXD023980 (http://proteomecentral.proteomexchange.org/cgi/GetDataset?ID=PXD023980) or IPX0002792000 (https://www.iprox.cn/page/project.html?id=IPX0002792000). Swiss-Prot protein databases used in this study are available at UniProt (https://www.uniprot.org), and have also been deposited

to the ProteomeXchange/iProX repository. Source data are provided with this paper.

Field-specific reporting

Please select the one below that is the best fit for your research. If you are not sure, read the appropriate sections before making your selection.

- [x] Life sciences
- [ ] Behavioural & social sciences
- [ ] Ecological, evolutionary & environmental sciences

For a reference copy of the document with all sections, see nature.com/documents/nr-reporting-summary-flat.pdf

Life sciences study design

All studies must disclose on these points even when the disclosure is negative.

Sample size	Each sample was analyzed by LC-MS/MS with 3 or 4 technical replicates. These sample sizes are usually used in DIA proteomics study to validate the technical variance. These sample sizes are sufficient to calculate the coefficients of variation for quantification and evaluate the level of missing values.
Data exclusions	No data were excluded.
Replication	Each sample was analyzed by LC-MS/MS with 3 or 4 technical replicates to verify the quantification results obtained by DDA and DIA. All attempts at replication were successful.
Randomization	Not applicable. In this study, performance of glycopeptide identification/quantification by DDA and DIA was benchmarked using technical replicates of each sample. No randomization was carried out.
Blinding	Not applicable. This study focuses on technical strength of our proposed method and does not report any biological findings. The samples used in this study are ordinary model specimens without labeling, grouping or classification. Therefore, no blinding was carried out.

Reporting for specific materials, systems and methods

We require information from authors about some types of materials, experimental systems and methods used in many studies. Here, indicate whether each material, system or method listed is relevant to your study. If you are not sure if a list item applies to your research, read the appropriate section before selecting a response.

Materials & experimental systems

n/a	Involved in the study	
x		Antibodies
x		Eukaryotic cell lines
x		Palaeontology and archaeology
x		Animals and other organisms
	x	Human research participants
x		Clinical data
x		Dual use research of concern

Methods

n/a	Involved in the study	
x		ChIP-seq
x		Flow cytometry
x		MRI-based neuroimaging

Human research participants

Policy information about studies involving human research participants

Population characteristics	Human serum samples were collected from 40 volunteers. A pooled serum specimen mixed from the samples was used in this study. All the study protocols and results are unrelated to characteristics of individual participant.
Recruitment	This study focuses on technical strength of our proposed method, instead of any biological findings. A pooled specimen was used as a model sample in this study. As all the study protocols and results are unrelated to characteristics of the recruited participants, no self-selection bias is introduced and all the results are not affected by recruitment.
Ethics oversight	The research protocol was approved by the Ethics Committee of the Fudan University, and complied with all relevant laws and regulations of China.

Note that full information on the approval of the study protocol must also be provided in the manuscript.
